# Iron-catalyzed stereoselective C–H alkylation for simultaneous construction of C–N axial and *C*-central chirality

**DOI:** 10.1038/s41467-024-47589-7

**Published:** 2024-04-25

**Authors:** Zi-Jing Zhang, Nicolas Jacob, Shilpa Bhatia, Philipp Boos, Xinran Chen, Joshua C. DeMuth, Antonis M. Messinis, Becky Bongsuiru Jei, João C. A. Oliveira, Aleksa Radović, Michael L. Neidig, Joanna Wencel-Delord, Lutz Ackermann

**Affiliations:** 1 Institut für Organische und Biomolekulare Chemie, Georg-August-Universität Göttingen, 37077 Göttingen, Germany; 2 Laboratoire d’Innovation Moléculaire et Applications (UMR CNRS 7042), Université de Strasbourg/Université de Haute-Alsace, ECPM, 67087 Strasbourg, France; 3 Department of Chemistry, University of Rochester, Rochester, NY 14627 USA; 4 Department of Chemistry, Zhejiang University, 310027 Hangzhou, China; 5 Inorganic Chemistry Laboratory, Department of Chemistry, University of Oxford, South Parks Road, Oxford, OX1 3QR UK; 6 Institut für Organische Chemie, Universität Würzburg, 97074 Würzburg, Germany; 7 Wöhler Research Institute for Sustainable Chemistry (WISCh), Georg-August-Universität Göttingen, 37077 Göttingen, Germany

**Keywords:** Asymmetric catalysis, Homogeneous catalysis

## Abstract

The assembly of chiral molecules with multiple stereogenic elements is challenging, and, despite of indisputable advances, largely limited to toxic, cost-intensive and precious metal catalysts. In sharp contrast, we herein disclose a versatile C–H alkylation using a non-toxic, low-cost iron catalyst for the synthesis of substituted indoles with two chiral elements. The key for achieving excellent diastereo- and enantioselectivity was substitution on a chiral *N*-heterocyclic carbene ligand providing steric hindrance and extra represented by noncovalent interaction for the concomitant generation of C–N axial chirality and *C*-stereogenic center. Experimental and computational mechanistic studies have unraveled the origin of the catalytic efficacy and stereoselectivity.

## Abstract (web summary)

A highly efficient stereoselective C−H alkylation of indoles with aryl alkenes is achieved by sustainable iron catalysis, leading to atropoenriched and enantioenriched substituted indoles with high structural diversity. Detailed mechanistic studies by experiment, Mössbauer spectroscopy and computation reveal the origin of the catalytic efficacy and stereoselectivity.

## Introduction

**Fig. 1:**
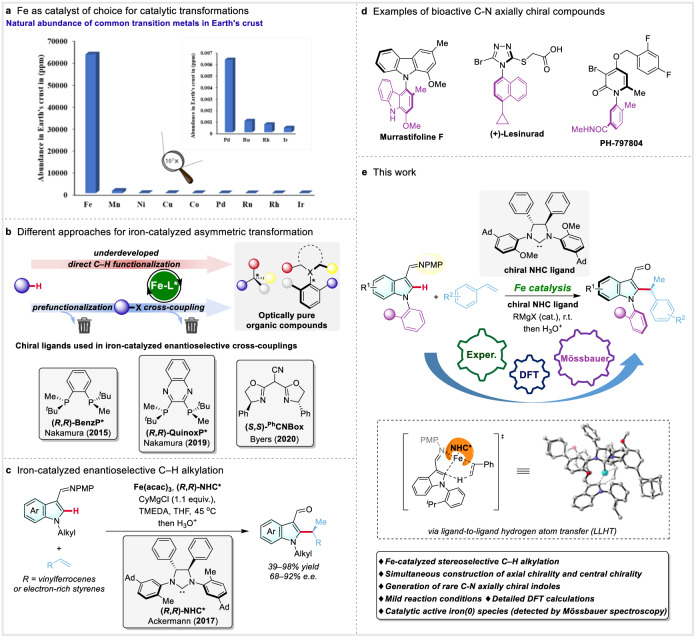
Toward new sustainable assembly of chiral indoles bearing two chiral elements by iron-catalyzed stereoselective C–H alkylation. **a** Iron as catalyst of choice for catalytic transformations. **b** Different approaches for iron-catalyzed asymmetric transformation. **c** Iron-catalyzed enantioselective C−H alkylation. **d** Examples of bioactive C−N axially chiral compounds. **e** This work: iron-catalyzed diastereo- and enantioselective C−H alkylation.

With the expanding importance of chiral molecules in various fields of chemistry, ranging from the pharmaceutical industry to agrochemistry, fragrances, and material science, the development of sustainable and highly selective methodologies allowing to rapidly access enantiopure complex molecules is amongst the most vivid fields of organic synthesis and catalysis^1–3^. While aiming for more sustainable synthesis, the expansion of the C–H activation field rapidly affording molecular complexity from simple, non-prefunctionalized substrates is certainly a key achievement^4^. However, the majority of stereoselective C–H functionalization strongly rely on the use of noble metal-based catalysts^5–10^, including palladium, iridium, or rhodium. In clear contrast, such transformation catalyzed by 3d-metals^11,12^, and in particular from the most abundant, low cost, and non-toxic iron^13–16^ (Fig. 1a), continues to be challenging. Indeed, various oxidation states of iron, combined with a diversity of reaction scenarios conceivable in a presence of such a catalyst and difficulty in isolating well-defined iron-complexes^17–20^, renders the chiral iron-catalysis still in its infancy.

Although a few asymmetric cross-coupling reactions catalyzed by chiral iron-phosphine^21,22^ and iron-oxazoline^23,24^ complexes have been achieved, the need to use prefunctionalized substrates and equivalent organometallic reagents limits their applications^25–27^ (Fig. 1b). And yet, while considering the complementary reactivity of iron-complexes and noble 4d- and 5d-metal catalyst, combined with often high reactivity of such complexes translating into mild reaction conditions^28–30^, stereoselective iron-catalyzed C–H activation holds great promise to expand the diversity of easily accessible enantiopure molecules. Building upon previous reports^31^, our group developed a highly enantioselective C–H alkylation of indoles with vinylferrocenes and electron-rich styrenes enabled by a newly designed chiral *N*-heterocyclic carbene (NHC)^32^ catalyst^15^ (Fig. 1c). However, it is worth noting that there has been limited subsequent research on iron-catalyzed asymmetric C–H activation.

Over the decades, *C*-stereogenic molecules have focused major scientific interest to escape from flatness^6^. However, the diversity of chiral molecules spreads far beyond, englobing *Si*-, *P*- and *S*-stereogenic molecules^33,34^, or atropisomeric biaryls, heterobiaryls and styrenes^35–38^. Besides, restricted rotation around an N–Ar bond has also been attracting considerable attention from the scientific community^39,40^, progressively evolving from purely fundamental curiosity to an appealing tool for the design of highly selective drug candidates^41–44^ (Fig. 1d). Synthetic routes affording such compounds are yet rare and almost exclusively based on the use of noble metals^45–52^. Moreover, further increasing molecular complexity and the three-dimensional structure of the molecules by introducing simultaneously two chirality elements via a one-step process remains challenging^53,54^. In particular, the possibility of simultaneously controlling C–N atropoisomerism and *C*-central chirality has rarely been reported^55–58^. Despite the originality of these examples, the need for expensive and rare rhodium, iridium or palladium-catalysts seriously limits the synthetic potential of these transformations.

Inspired by previous research^15,31,59^, we were wondering if a more challenging C–N atroposelective transformation by iron catalysis could also be designed. Indeed, the introduction of a suitable, sufficiently sterically congested aromatic substituent on the N-atom could translate into the generation of an atropisomeric compound via the introduction of a substituent at the C2-position. Moreover, the possibility of generating an additional element of chirality by trapping the chiral metalacyclic intermediate with a prochiral coupling partner, such as olefin, appears as an ultimate challenge. Such an unprecedented transformation would involve not only the stereoselective olefin insertion but also simultaneously impose the configuration of the N–Ar bond thus promoting the formation of the indole product with two chiral elements.

Herein, we report an original, iron–NHC complex catalyzed asymmetric transformation delivering substituted indoles bearing both, C–N axial and *C*-central chirality, which is a unique and more challenging achievement to realize simultaneous stereocontrol of two chiral elements within one elementary step^60^ compared to single chiral center construction^15^ (Fig. 1e). The salient features of this reaction are: 1) the first example of iron-catalyzed C–N atroposelective reaction; 2) the first example of the use of iron-catalyst to build up complex molecules bearing both, axial and central chirality; 3) synthesis of various  substituted indoles exhibiting complex three-dimensional structure. In addition to these synthetic values, detailed experimental and theoretical mechanistic studies allowed shedding light on the asymmetric iron-catalysis from a broader perspective.

## Results and discussion

### Optimization of reaction conditions

**Fig. 2:**
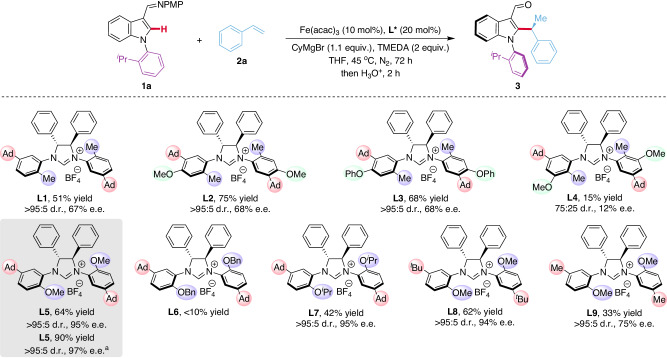
Condition optimization for the iron-catalyzed asymmetric C–H alkylation. Reaction conditions: **1a** (0.1 mmol), **2a** (0.15 mmol), Fe(acac)_3_ (10 mol%), L* (20 mol%), CyMgBr (1 M in THF, 0.11 mmol) and TMEDA (0.2 mmol) were stirred in THF (0.2 mL) at 45 °C for 72 h under N_2_, then added HCl aq. (1 M, 1.0 mL) and stirred for 2 h. The yield was determined by ^1^H NMR spectroscopy using 1,3,5-trimethoxybenzene as the internal standard. The diastereomeric ratio (d.r.) was determined by ^1^H NMR spectroscopy. The enantiomeric excess (e.e.) was determined by HPLC. ^a^**1a** (0.1 mmol), **2a** (0.15 mmol), Fe(acac)_3_ (10 mol%), **L5** (10 mol%), CyMgBr (1 M in THF, 50 mol%) and TMEDA (0.2 mmol) were stirred in Et_2_O (0.2 mL) at room temperature for 72 h under N_2_. TMEDA, *N,N,N’,N’*-tetramethylethylenediamine; THF, tetrahydrofuran.

We initiated our studies into the stereoselective C–H alkylation of indole derivative **1a** with styrene **2a** using the Fe/NHC catalytic system (Fig. 2). Pleasingly, the desired alkylation product **3** was obtained in 51% yield with >95:5 diastereomeric ratio (d.r.) and 67% enantiomeric excess (e.e.) when using Fe(acac)_3_ as the metal catalyst and **L1** as chiral ligand. Subsequently, the structure of the chiral NHC ligand was further probed. The perfect diastereocontrol have been achieved through careful ligand design. The introduction of a methoxy or phenoxy group at the *para*-position of the aromatic ring (**L2,** **L3**) had a minor effect on the stereoselectivity of the alkylation reaction, while the presence of an additional methoxy group at the *meta*-position (**L4**) translated into a severe decrease in both reaction efficiency and stereoselectivity. In contrast, a remarkably 95% e.e. was realized when a more electron-donating and less sterically demanding methoxy motif is present at the *ortho*-position of the ligand (**L5**). Further investigations into the role of the *ortho*-substituent of the aromatic ring revealed that the benzyloxy-substituted ligand (**L6**) is inefficient while isopropoxy-derived **L7** furnished the desired product in the same 95% e.e., but a lower yield. The substitution pattern at the *meta*-position had a dramatic impact on the stereoinduction. While a *tert*-butyl derived ligand (**L8**) performed equally well as the adamantyl congener (**L5**), a significant drop in the stereoinduction to 75% e.e. was observed with a methyl-substituted **L9**.

Next, the reaction conditions were further optimized. The tuning of the reaction parameters, including solvent and temperature illustrated that high yield and enantioselectivity could be obtained when the reaction was performed in Et_2_O at a relatively low temperature. Remarkably, the optimal results in terms of isolated yield (90%), diastereo- (>95:5) and enantioselectivity (97%) were realized using a reduced amount of the chiral NHC ligand (10 mol%), reflecting the efficiency and potential of this new catalytic system (see Supplementary Table 2 for details).

### Substrate scope

**Fig. 3:**
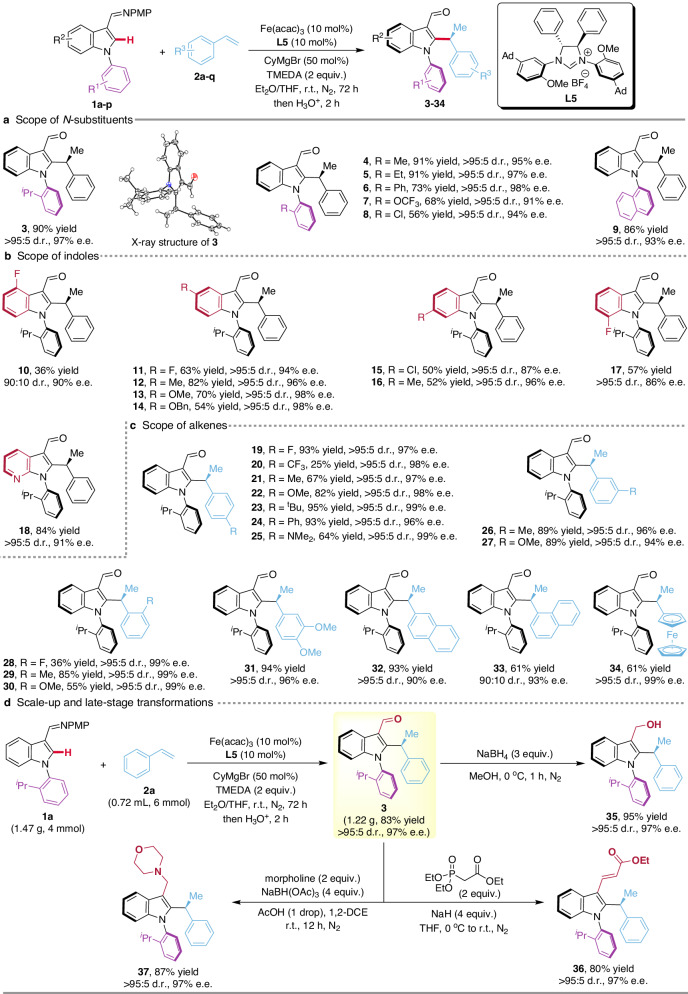
Substrate scope and synthetic transformations for the iron-catalyzed asymmetric C–H alkylation. **a** *N*-substituent pattern. **b** Robustness with respect to indoles. **c** Scope of alkenes. **d** Scale-up and late-stage transformations. Reaction conditions: **1** (0.1 mmol), **2** (0.15 mmol), Fe(acac)_3_ (10 mol%), **L5** (10 mol%), CyMgBr (1 M in THF, 50 mol%) and TMEDA (0.2 mmol) were stirred in Et_2_O (0.2 mL) at room temperature for 72 h under N_2_, then added HCl aq. (1 M, 1.0 mL) and stirred for 2 h. Yields are those of the isolated products. The diastereomeric ratio (d.r.) was determined by ^1^H NMR spectroscopy. The enantiomeric excess (e.e.) was determined by HPLC.

To delineate the robustness of the diastereo- and enantioselective iron catalysis, indoles bearing a variety of *N*-aryl substituents were explored (Fig. 3a). Electronic changes and the steric hindrance of the *ortho*-substituents of the *N*-aromatic ring had a minor impact on the stereoselectivity of this reaction (**3**–**9**, all >95:5 d.r., up to 98% e.e.) and the atropostability of the products was guaranteed even for the less hindered methyl-substituted product **4**. Further studies showed that both electron-withdrawing and electron-donating groups on the indole ring were well tolerated, delivering the desired products **10**–**17** in moderate to good yields with excellent diastereo- and enantioselectivities (up to >95:5 d.r., 98% e.e.) (Fig. 3b). Notably, azaindole **1p** was also a potent substrate, furnishing product **18** in a good yield with a high level of stereoselectivity (>95:5 d.r., 91% e.e.). The reaction scope regarding the alkene was also very broad (Fig. 3c). A variety of differently substituted alkenes bearing electron-withdrawing or electron-donating substituents on the *para*-, *meta*- or *ortho*-position of aromatic ring furnished the desired products **19**–**31** in excellent stereoselectivities (all >95:5 d.r., 94-99% e.e.). 2-Vinylnaphthalene, 1-vinylnaphthalene, and vinylferrocene were compatible and afforded the target products **32**–**34** in good yields and enantioselectivities (90-99% e.e.). The absolute configuration of the alkylation products was confirmed unambiguously by a single-crystal X-ray diffraction analysis of indole product **3**.

### Scale-up and late-stage transformations

A gram-scale reaction of indole derivative **1a** and styrene **2a** was successfully performed, delivering the desired product **3** in 83% yield without deterioration of the stereoselectivity (Fig. 3d), thereby demonstrating the utility of the stereoselective iron catalysis in preparative-scale organic synthesis. The presence of the formyl group on the product provided also important opportunities for expanding the molecular diversity. Accordingly, the corresponding alcohol **35**, olefin **36**, and amino **37** were accessed without loss of optical purity, as shown in Fig. 3d.

### Mechanistic studies

**Fig. 4:**
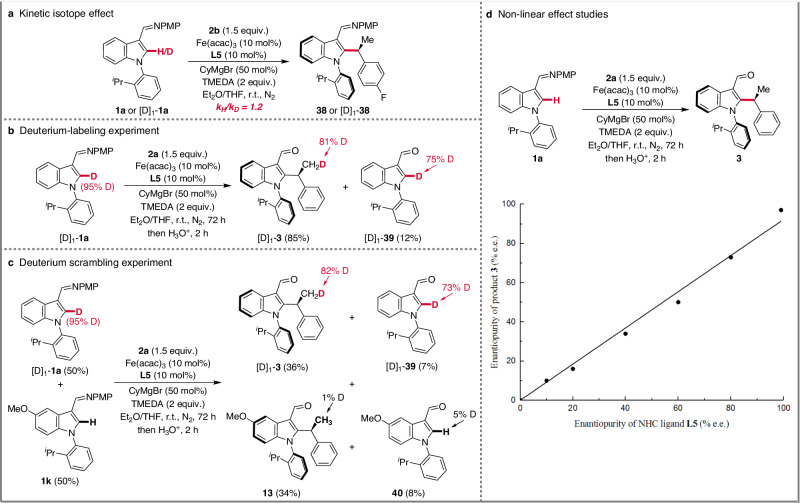
Mechanistic studies. **a** Kinetic isotope effect. **b** Deuterium-labeling experiment. **c** Deuterium scrambling experiment. **d** Non-linear effect studies.

To gain insights into the reaction mechanism, a series of experiments were carried out (Fig. 4). First, the kinetic isotope effect (KIE) study suggested that the C–H cleavage is facile (Fig. 4a). The reaction of the C2-deuterated indole substrate [D]_1_-**1a** with styrene **2a** afforded the product [D]_1_-**3** together with 12% of the unreacted substrate, isolated as a 75:25 mixture of [D]_1_-**39** and **39**. As to the alkylation product [D]_1_-**3**, the significant transfer of the C2-deuterium from the indole into the methyl position (81% D) indicates that the C−H activation occurs by ligand-to-ligand hydrogen transfer (LLHT) or C−H oxidative addition (Fig. 4b). In addition, a competition experiment between two different substrates ([D]_1_-**1a** and **1k**) suggested that there was no deuterium scrambling distribution (Fig. 4c). Non-linear effect studies revealed a linear relation between the enantiopurity of the ligand and the e.e. of the product formed (Fig. 4d). This result is suggestive of only one NHC ligand being involved in the control of the reaction stereoselectivity.

**Fig. 5:**
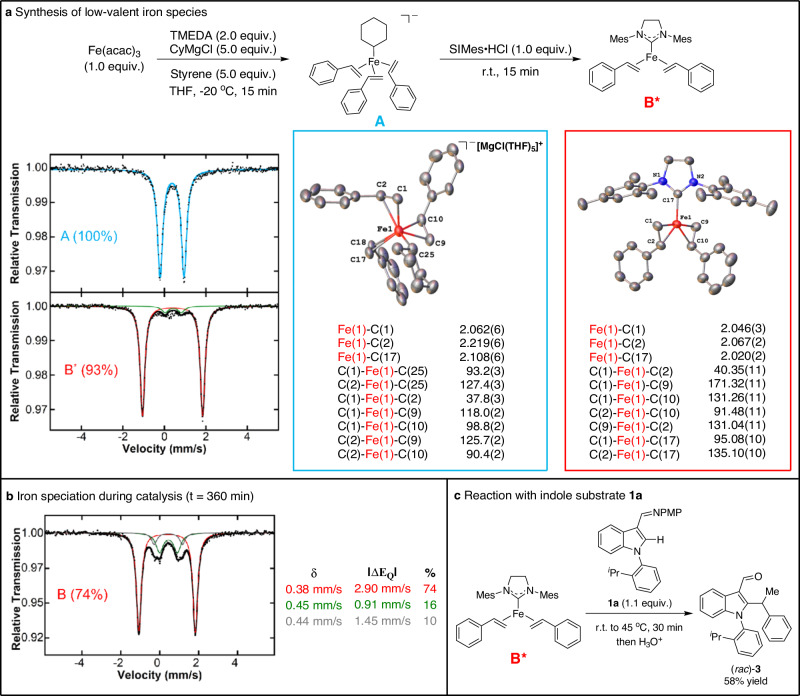
Active catalyst studies. **a** Reaction pathway for generating **A** and B^*^, respectively. 80 K ^57^Fe Mössbauer spectra of their solid crystals with parameters δ = 0.37 mm/s and |ΔE_Q_ | = 1.16 mm/s (**A**) and δ = 0.39 mm/s and |ΔE_Q_ | = 2.92 mm/s (B^*^), and their crystal structures with selected bond lengths and angles. **b** 80 K ^57^Fe Mössbauer spectrum of the catalytic reaction with chiral NHC **L5** at t = 360 min. **c** Reaction of in situ generated B^*^ with indole substrate **1a** to generate C−H alkylated product.

Further studies were pursued to 1) define the potential reactive iron-NHC species responsible for C–H activation and 2) obtain molecular-level insight into the role of the TMEDA additive in catalysis. For the latter, a recent report demonstrated the generation of a low-valent Fe-styrene species under similar reaction conditions which could be a precursor to a low-valent Fe-NHC complex for catalysis^61^. Consistent with this hypothesis, the synthesis of the Fe(0) species [Fe(Cy)(η^2^-styrene)_3_][MgCl(THF)_5_] (**A**) with the cyclohexyl Grignard reagent (CyMgCl) (Fig. 5a) and subsequent reaction with ligand **L5** in the presence of excess CyMgCl led to the formation of a new iron species as identified by in situ freeze-trapped 80 K ^57^Fe Mössbauer spectroscopy (see Supplementary Information for details). This species was postulated to be a low-valent Fe(**L5**)(η^2^-styrene)_2_ complex (**B**) as related compounds have been reported in the literature^62,63^. These studies were further extended to evaluation of the in situ iron speciation during catalysis, where freeze-trapped ^57^Fe Mössbauer spectroscopy at 360 minutes into the C–H alkylation reaction revealed the presence of a single major iron species with parameters similar to **B** (Fig. 5b).

While **B** was not readily amenable to crystallographic analysis, utilizing the achiral surrogate SIMes•HCl (possessing a saturated backbone like **L5**) enabled access to the *S* = 1 iron(0) complex Fe(SIMes)(η^2^-styrene)_2_ (B^*^) (Fig. 5a) which, combined with Mössbauer parameter calculations (see Supplementary Information for details), further supported the assignment of **B** as Fe(**L5**)(η^2^-styrene)_2_. To evaluate the catalytic relevance of such low coordinate Fe-NHC species, the reaction of B^*^ with indole substrate **1a** resulted in the generation of the C–H alkylated product (*rac*)-**3** (Fig. 5c). Overall, these results support Fe(**L**)(η^2^-styrene)_2_ species as the likely active iron species in the current system, accessed from the low-valent catalytic precursor [Fe(Cy)(η^2^-styrene)_3_]^-^.

### Computational studies

**Fig. 6:**
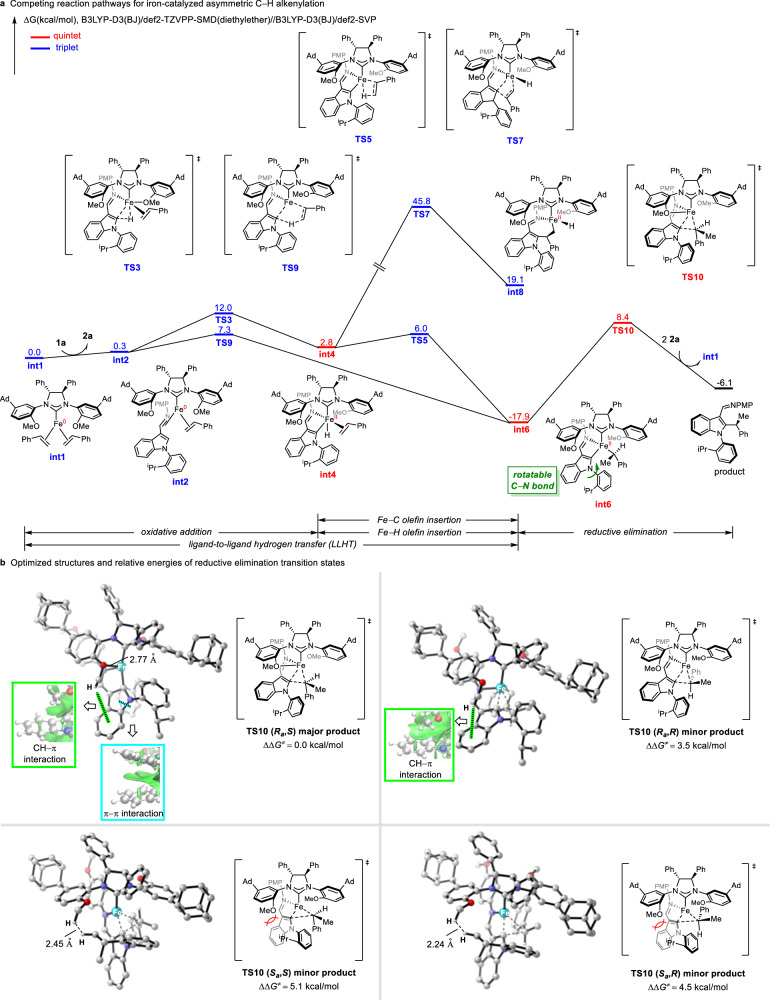
DFT calculations on the reaction mechanism and origins of the stereoselectivity. **a** DFT-computed free energy profile of the competing reaction pathways for iron-catalyzed asymmetric C−H alkylation for major product. **b** Optimized structures and relative energies of reductive elimination transition states (trivial hydrogens are omitted for clarity).

To understand the mechanistic details and to account for the origins of stereoselectivity, we furthermore performed density functional theory (DFT) calculations^64^. The competing reaction pathways for the iron-catalyzed asymmetric C−H alkylation for (*R*_*a*_,*S*)-conformer are shown in Fig. 6a. Initiating from the triplet iron(0) complex **int1** ligated by chiral NHC and two styrenes, whose reactivity already had been verified experimentally (Fig. 5c), ligand exchange occurs forming substrate coordinated intermediate **int2**. Classic oxidative addition of the indole C−H bond to iron center via triplet three-membered transition state **TS3** is viable, generating quintet iron(II) hydride **int4**. Subsequent olefin insertion into Fe−H bond is facile leading to quintet alkyl iron(II) intermediate **int6**. However, the competing LLHT pathway from **int2** directly to **int6** is more favorable by 4.7 kcal/mol than the oxidative addition with sequential olefin insertion into Fe−H bond. We also considered the possibility of olefin insertion into the Fe−C bond via four-membered transition state **TS7**, while this insertion is proven to be unfeasible. Therefore, LLHT is most likely the preferred pathway for the generation of the alkyl iron(II) intermediate **int6**, where free rotation over the C−N axis is still achievable (see Supplementary Fig. 19). **Int6** then undergoes irreversible reductive elimination which is the rate- and enantio-determining step to release the product and meanwhile regenerate the catalytic active iron(0) species **int1**. It is worth mentioning that only the most stable spin state of each species is presented in Fig. 6a, for the free energy profile of all possible spin states see Supplementary Fig. 18.

Based on these experimental and computational findings, we became interested in the controlling factors of stereoselectivity. The four reductive elimination transition states are shown in Fig. 6b with respective optimized structures and energies. **TS10(*R***_**a**_,***S*)** is at least 3.5 kcal/mol more favorable than the other three transition states **TS10(*R***_**a**_,***R*),** **TS10(*S***_**a**_,***S*)** and **TS10(*S***_**a**_,***R***). The coordination of methoxy group to the iron center stabilizes the transition state **TS10(*R***_**a**_,***S*)** for major product. Moreover, noncovalent interactions play an important role in the stereoselectivity control. The CH–π interaction in **TS10(*R***_**a**_,***S*)** and **TS10(*R***_**a**_,***R*)** between the methoxyl group of the NHC ligand and indole determines the axial chirality. This CH–π interaction is confirmed by the independent gradient model (IGM) analysis^65^. The same CH–π interaction is not present in **TS10(*S***_**a**_,***S*)** or **TS10(*S***_**a**_,***R*)**. On the contrary, the steric hindrance between the methoxy group and the isopropyl group of the substrate is instead dominant. Another stereoselectivity-controlling factor is π-π stacking interaction in transition state **TS10(*R***_**a**_,***S*)** between the indole and phenyl group, which determines the central chirality. The energy decomposition analysis of **TS10(*R***_**a**_,***S*)** also reflects the impact of the noncovalent interactions (Supplementary Fig. 20). The coordination in combination with noncovalent interaction in transition state **TS10(*R***_**a**_,***S*)** highlights the key role of methoxy group of chiral NHC.

A highly efficient selective C−H alkylation of indoles with aryl alkenes was achieved by sustainable iron catalysis, leading to rare atropoenriched and enantioenriched substituted indoles with high structural diversity. This iron-catalyzed asymmetric C−H alkylation was viable at room temperature, demonstrating the great potential of iron catalysis in the field of stereoselective C−H activation. Detailed mechanistic studies by experiment, Mössbauer spectroscopy and computation revealed an iron(0) complex as catalytic active species and this iron(0) complex undergoes a LLHT process instead of classic C−H bond oxidative addition. Computational studies revealed that the involvement of the methoxy group in the side arm of the chiral NHC ligand is vital for providing both covalent and noncovalent interaction in the rate- and enantio-determining transition state. This insight emphasizes the necessity for the de novo design of chiral ligands in asymmetric synthesis. We envisioned that the present approach and the mechanistic findings will promote the development in related challenging iron-catalyzed C−H functionalization constructing multiple chiral centers.

## Methods

### General procedure for iron-catalyzed stereoselective C–H alkylation

To a flame-dried and N_2_-purged Schlenk tube were added indole substrate (0.1 mmol), Fe(acac)_3_ (0.01 mmol, 10 mol%) and chiral NHC ligand **L5** (0.01 mmol, 10 mol%). The Schlenk tube was then sealed, purged and backfilled with N_2_ three times. Et_2_O (0.2 mL), TMEDA (0.2 mmol) and alkene substrate (0.15 mmol) were added via syringe. CyMgBr (1 M in THF, 0.05 mmol, 0.05 mL) was then added dropwise and the resulting mixture was stirred at room temperature for 72 hours. Then, the reaction mixture was diluted with THF (2.0 mL) and quenched with HCl aqueous solution (1 M, 1.0 mL). The resulting mixture was stirred at room temperature for 2 hours. The phases were then separated, the aqueous layer was extracted with ethyl acetate (5.0 mL ×3). The combined organic layer was washed with brine, dried over Na_2_SO_4_, filtered and concentrated *in vacuo*. The diastereomeric ratio was determined by ^1^H NMR analysis of the crude reaction mixture. The residue was purified by column chromatography on silica gel (*n*-hexane: ethyl acetate = 10:1) to afford the desired product.

### Supplementary information

Supplementary Information

Peer Review File

Description of Additional Supplementary Files

Supplementary Data 1

## Data Availability

The data that support the findings of this study are available within the main text, the Supplementary Information and the Supplementary Data. Details about materials, methods, experimental procedures, characterization data, NMR and HPLC spectra are available in the Supplementary Information, cartesian coordinates and atomic coordinates are available in the Supplementary Data and all other data are available from the corresponding author upon request. Crystallographic data for the structures reported in this article have been deposited at the Cambridge Crystallographic Data Centre, under deposition numbers CCDC 2176328 (**3**), CCDC 2296422 (**A**), CCDC 2296423 (**B**^*****^). Copies of the data can be obtained free of charge via https://www.ccdc.cam.ac.uk/structures/.
