# Supplementary material for: Iron-catalyzed stereoselective C–H alkylation for simultaneous construction of C–N axial and C-central chirality
Source: Nat Commun. 2024 Apr 25;15:3503. doi: 10.1038/s41467-024-47589-7 (PMC11045758; doi:10.1038/s41467-024-47589-7)
Supplement: Supplementary file 1 — Supplementary Information [file 41467_2024_47589_MOESM1_ESM.pdf]

Supplementary Information

Iron-catalyzed stereoselective C–H alkylation for simultaneous construction of C–N axial and *C*-central chirality

Zi-Jing Zhang, Nicolas Jacob, Shilpa Bhatia, Philipp Boos, Xinran Chen, Joshua C. DeMuth, Antonis M. Messinis, Becky Bongsuiru Jei, João C. A. Oliveira, Aleksa Radović, Michael L. Neidig*, Joanna Wencel-Delord* and Lutz Ackermann*

**Email: Lutz.Ackermann@chemie.unigoettingen.de; wenceldelord@unistra.fr; michael.neidig@chem.ox.ac.uk.*

Table of Contents

1. General information..........2
2. Details for condition optimization..........3
3. Synthesis of chiral NHC ligands..........6
4. Synthesis of indole substrates..........13
5. General procedure for iron-catalyzed stereoselective C–H alkylation..........23
6. Characterization data of products..........24
7. Scale-up reaction..........64
8. Late-stage transformations..........65
9. Mechanistic investigations..........70
10. DFT calculations..........89
11. X-ray single crystal data..........96
12. NMR spectra..........108
13. References..........188

1. General information

General data:

NMR spectra were recorded on Bruker-300 MHz spectrometer or Bruker-400 MHz spectrometer. Chemical shifts (δ) are given in ppm relative to TMS. The residual solvent signals were used as references and the chemical shifts converted to the TMS scale ($CDCl_3$: δH = 7.26 ppm, δC = 77.16 ppm).

High resolution mass spectra were recorded on a Thermo LTQ Orbitrap XL (ESI+) or a P-SIMS-Gly of Bruker Daltonics Inc (EI+).

Infrared spectra were recorded on a Nicolet MX-1E FT-IR spectrometer.

Enantiomeric excesses were measured on Agilent 1290 Infinity HPLC (2487 Dual λ Absorbance Detector and 1525 Binary HPLC Pump, UV detection monitored at 250 nm). Chiralpak IC-3, ID-3, IE-3 and OD-3 columns were purchased from Daicel Chemical Industries, Ltd.

Optical rotations were measured at 589 nm (sodium D line) by using a Perkin-Elmer 343 polarimeter.

Melting point ranges are measured with a Stuart Melting Point Apparatus SMP3 (Barloworld Scientific, Ltd.).

Materials:

All starting materials, reagents and solvents were purchased from commercial suppliers (Aldrich, Alfa, TCI, Daicel, etc.) and used as supplied unless otherwise stated. Chiral *N*-heterocyclic carbene (NHC) ligands were synthesized in accordance with the similar procedures in literature[1-4]. Diethyl ether and tetrahydrofuran were dried over Na and distilled prior to use.

2. Details for condition optimization

Supplementary Table 1. Screening of chiral NHC ligands[a]

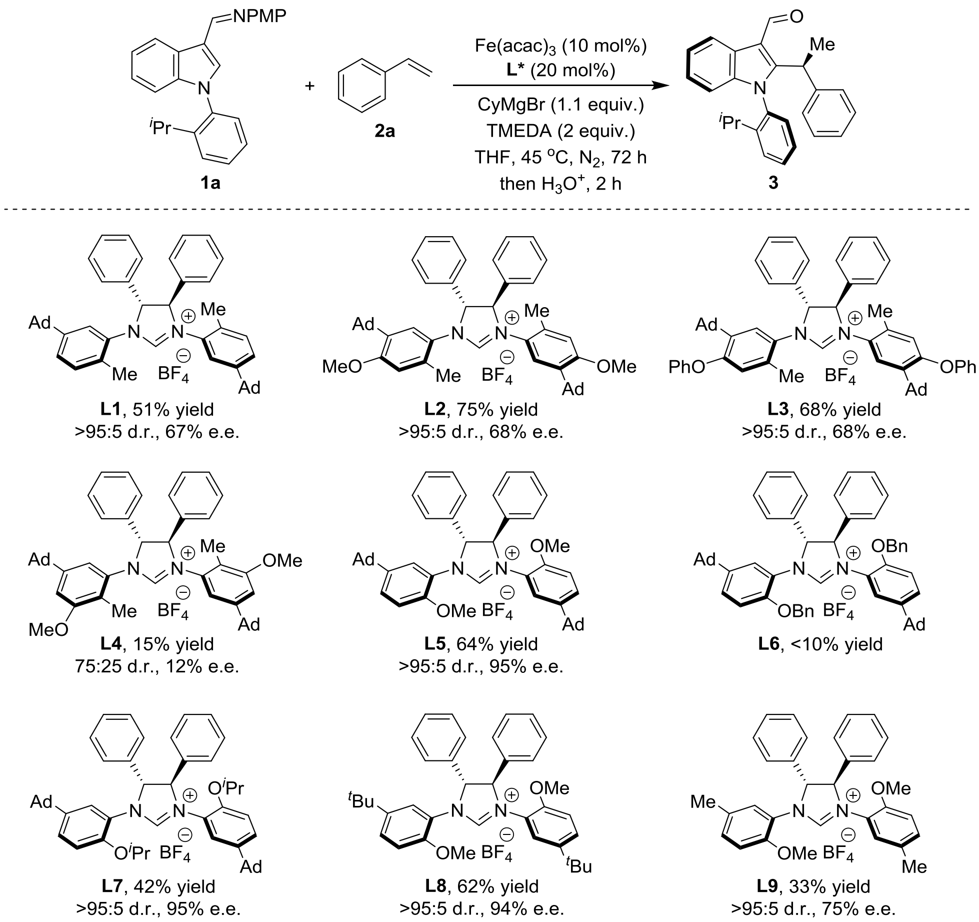

[a]Reaction conditions: **1a** (0.1 mmol), **2a** (0.15 mmol), Fe(acac)$_3$ (10 mol%), **L*** (20 mol%), CyMgBr (1 M in THF, 0.11 mmol) and TMEDA (0.2 mmol) were stirred in THF (0.2 mL) at 45 °C for 72 h under N_2, then added HCl aq. (1 M, 1.0 mL) and stirred for 2 h. The yield was determined by ^{1}H NMR spectroscopy using 1,3,5-trimethoxybenzene as the internal standard. The diastereomeric ratio (d.r.) was determined by ^{1}H NMR spectroscopy. The enantiomeric excess (e.e.) was determined by HPLC. TMEDA, *N,N,N',N'*-tetramethylethylenediamine; THF, tetrahydrofuran.

Supplementary Table 2. Optimization of reaction conditions

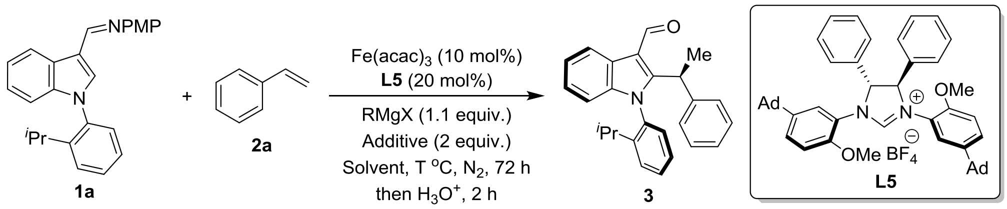

Entry[a]	RMgX	Additive	Solvent	T (°C)	Yield (%)	d.r.	e.e. (%)
1	CyMgBr	TMEDA	THF	45	64	>95:5	95
2	MeMgBr	TMEDA	THF	45	n.d.	-	-
3	EtMgBr	TMEDA	THF	45	<10	-	-
4	^{t}BuMgCl	TMEDA	THF	45	<10	-	-
5	PhMgBr	TMEDA	THF	45	n.d.	-	-
6	^{i}PrCH$_2$MgBr	TMEDA	THF	45	11	>95:5	93
7	Me$_3$SiCH$_2$MgCl	TMEDA	THF	45	n.d.	-	-
8	CyMgCl	TMEDA	THF	45	63	>95:5	95
9	CyMgBr	DMEDA	THF	45	n.d.	-	-
10	CyMgBr	NEt$_3$	THF	45	54	>95:5	92
11	CyMgBr	TMEDA	2-MeTHF	45	63	>95:5	94
12	CyMgBr	TMEDA	Toluene	45	68	>95:5	95
13	CyMgBr	TMEDA	Et$_2$O	45	74	>95:5	96
14[b]	CyMgBr	TMEDA	Et$_2$O	45	78	>95:5	96
15[c]	CyMgBr	TMEDA	Et$_2$O	45	84	>95:5	96
16[d]	CyMgBr	TMEDA	Et$_2$O	45	21	>95:5	95
17	CyMgBr	TMEDA	Et$_2$O	60	80	89:11	96
18	CyMgBr	TMEDA	Et$_2$O	r.t.	87	>95:5	97
19[c]	CyMgBr	TMEDA	Et$_2$O	r.t.	92	>95:5	97
20[e]	CyMgBr	TMEDA	Et$_2$O	r.t.	80	>95:5	97
21[f]	CyMgBr	TMEDA	Et$_2$O	r.t.	68	>95:5	96
22[g]	CyMgBr	TMEDA	Et$_2$O	r.t.	46	>95:5	96
23[h]	CyMgBr	TMEDA	Et$_2$O	r.t.	n.d.	-	-
24[i]	CyMgBr	TMEDA	Et$_2$O	r.t.	n.d.	-	-
25[j]	CyMgBr	TMEDA	Et$_2$O	r.t.	n.d.	-	-
26[k]	CyMgBr	TMEDA	Et$_2$O	r.t.	n.d.	-	-
27[l]	CyMgBr	TMEDA	Et$_2$O	r.t.	n.d.	-	-
28[m]	CyMgBr	TMEDA	Et$_2$O	r.t.	n.d.	-	-
29	-	TMEDA	Et$_2$O	r.t.	n.d.	-	-
30	CyMgBr	-	Et$_2$O	r.t.	42	>95:5	96
31[c,n]	CyMgBr	TMEDA	Et$_2$O	r.t.	92	>95:5	97
32[c,o]	CyMgBr	TMEDA	Et$_2$O	r.t.	91(90)	>95:5	97
33[c,p]	CyMgBr	TMEDA	Et$_2$O	r.t.	82	>95:5	96
34[c,q]	CyMgBr	TMEDA	Et$_2$O	r.t.	45	>95:5	93

[a]Reaction conditions: **1a** (0.1 mmol), **2a** (0.15 mmol), Fe(acac)$_3$ (10 mol%), **L5** (20 mol%), RMgX (1 M in THF, 0.11 mmol) and additive (0.2 mmol) were stirred in solvent (0.2 mL) at T °C for 72 h under N$_2$, then added HCl aq. (1 M, 1.0 mL) and stirred for 2 h. The yield was determined by ^{1}H NMR spectroscopy using 1,3,5-trimethoxybenzene as the internal standard (yield of isolated product

given within parentheses). The diastereomeric ratio (d.r.) was determined by ^{1}H NMR spectroscopy. The enantiomeric excess (e.e.) was determined by HPLC. [b]Use **2a** (0.2 mmol). [c]Use CyMgBr (1 M in THF, 0.05 mmol). [d]Use CyMgBr (1 M in THF, 0.2 mmol). [e]$Fe(acac)_2$ was used instead of $Fe(acac)_3$. [f]$FeCl_3$ was used instead of $Fe(acac)_3$. [g]$FeCl_2$ was used instead of $Fe(acac)_3$. [h]$Co(acac)_2$ was used instead of $Fe(acac)_3$. [i]$Ni(acac)_2$ was used instead of $Fe(acac)_3$. [j]CuI was used instead of $Fe(acac)_3$. [k]$Pd_2(dba)_3$ was used instead of $Fe(acac)_3$. [l]In the absence of $Fe(acac)_3$. [m]In the absence of **L5**. [n]$Fe(acac)_3$ (10 mol%), **L5** (15 mol%). [o]$Fe(acac)_3$ (10 mol%), **L5** (10 mol%). [p]$Fe(acac)_3$ (5 mol%), **L5** (10 mol%). [q]$Fe(acac)_3$ (5 mol%), **L5** (5 mol%). DMEDA, *N,N'*-dimethyl-1,2-ethanediamine; n.d., not detected; r.t., room temperature.

3. Synthesis of chiral NHC ligands

General Procedure 1: synthesis of chiral NHC ligands[1,2]

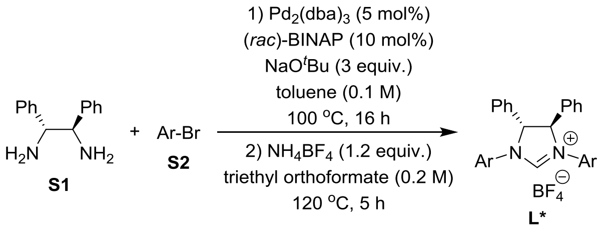

In a round-bottomed flask under nitrogen, were added the (1*R*,2*R*)-1,2-diphenylethane-1,2-diamine **S1** (1.0 equiv., 2.0 mmol, 424.3 mg), aryl bromide derivative **S2** (2.1 equiv., 4.2 mmol), $Pd_2(dba)_3$ (5 mol%, 0.1 mmol, 91.6 mg), (*rac*)-BINAP (10 mol%, 0.2 mmol, 124.5 mg), NaO^{t}Bu (3.0 equiv., 6.0 mmol, 576.3 mg) and toluene (0.1 M, 20.0 mL). The reaction mixture was degassed and stirred at 100 °C for 16 hours. After 16 hours, the reaction mixture was filtered through a pad of Celite (eluted with CH_2Cl_2), then the solvent was removed *in vacuo*. The crude product was purified quickly by flash chromatography on silica gel (*n*-hexane: dichloromethane = 10:1).

The crude *N,N'*-diarylated diamine was then treated with ammonium tetrafluoroborate (1.2 equiv., 2.4 mmol, 252.1 mg) and triethyl orthoformate (0.2 M, 10.0 mL) for 5 hours at 120 °C. After concentration *in vacuo*, the crude mixture was purified *via* flash chromatography (dichloromethane 100% to dichloromethane: acetone = 10:1) to afford the desired compound **L***.

General Procedure 2: synthesis of chiral NHC ligands[3,4]

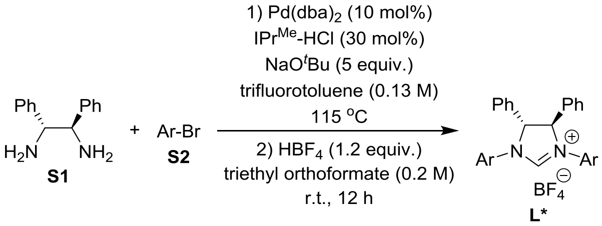

In a round-bottomed flask under nitrogen, were added the (1*R*,2*R*)-1,2-diphenylethane-1,2-diamine **S1** (1.0 equiv., 2.0 mmol, 424.3 mg), aryl bromide derivative **S2** (2.1 equiv., 4.2 mmol), $Pd(dba)_2$ (10 mol%, 0.2 mmol, 115.0 mg), IPr^{Me}·HCl (30 mol%, 0.6 mmol, 271.4 mg), NaO^{t}Bu (5.0 equiv., 10.0 mmol, 960.6 mg) and trifluorotoluene (0.13 M, 15.0 mL). The reaction mixture was degassed and stirred at 115 °C until full conversion of the starting material. Then, the reaction mixture was allowed to cool to room temperature and was filtered through a pad of Celite (eluted with CH_2Cl_2), then the solvents were removed *in vacuo*. The crude product was washed with methanol or purified by chromatography on silica gel (*n*-hexane: dichloromethane = 10:1).

The crude *N,N'*-diarylated diamine was then treated with aqueous HBF_4 (48%w in water, 1.2 equiv., 2.4 mmol, 440.1 mg) and triethyl orthoformate (0.2 M, 10.0 mL) for 12 hours at room temperature. After concentration *in vacuo*, the crude mixture was purified *via* flash chromatography (dichloromethane 100% to dichloromethane: acetone = 10:1) to afford the desired compound **L***.

(4*R*,5*R*)-1,3-Bis(5-(adamantan-1-yl)-2-methylphenyl)-4,5-diphenyl-4,5-dihydro-1*H*-imidazol-3-ium tetrafluoroborate (L1)

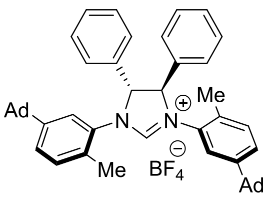

The **general procedure 1** was followed using 1-(3-bromo-4-methylphenyl)adamantane (4.2 mmol, 1.28 g) to afford **L1** (1.13 g, 74% yield over 2 steps) as a pale yellow solid. **M.p.**: 198-201 °C. **^{1}H NMR (400 MHz, $CDCl_3$)** δ 8.57 (s, 1H), 7.51 – 7.35 (m, 10H), 7.26 (d, *J* = 1.7 Hz, 2H), 7.21 (dd, *J* = 8.1, 2.0 Hz, 2H), 7.14 (d, *J* = 8.1 Hz, 2H), 5.80 (s, 2H), 2.46 (s, 6H), 2.13 – 1.98 (m, 6H), 1.77 – 1.67 (m, 24H). **^{13}C NMR (101 MHz, $CDCl_3$)** δ 157.2 (CH), 151.5 (C_q), 133.9 (C_q), 132.5 (C_q), 131.4 (CH), 130.4 (CH), 130.0 (C_q), 129.8 (CH), 128.5 (CH), 126.7 (CH), 124.7 (CH), 76.2 (CH), 42.7 (CH_2), 36.7 (CH_2), 36.2 (C_q), 28.9 (CH), 18.0 (CH_3). **^{19}F NMR (377 MHz, $CDCl_3$)** δ -151.06, -151.11. **IR** (ATR) $\tilde{\nu}$ = 3062, 3035, 2900, 2847, 1624, 1604, 1566, 1454, 1269, 1213, 1055, 757, 700 cm^{-1}. **HRMS** (ESI) m/z $(M–BF_4)^+$: calculated for $(C_{49}H_{55}N_2)^+$: 671.4360, found: 671.4360. **$[\alpha]_D^{20}$** = +298.2 (c = 0.67, $CHCl_3$).

(4*R*,5*R*)-1,3-Bis(5-(adamantan-1-yl)-4-methoxy-2-methylphenyl)-4,5-diphenyl-4,5-dihydro-1*H*-imidazol-3-ium tetrafluoroborate (L2)

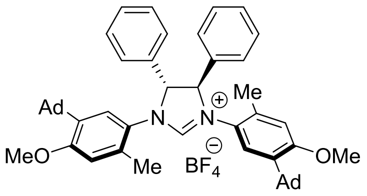

The **general procedure 2** was followed using 1-(5-bromo-2-methoxy-4-methylphenyl)adamantane (4.2 mmol, 1.40 g) to afford **L2** (1.39 g, 85% yield over 2 steps) as a pale yellow solid. **M.p.**: 196-198 °C. **^{1}H NMR (400 MHz, $CDCl_3$)** δ 8.62 (s, 1H), 7.50 – 7.35 (m, 10H), 6.80 (s, 2H), 6.66 (s, 2H), 5.57 (s, 2H), 3.78 (s, 6H), 2.46 (s, 6H), 2.05 – 1.94 (m, 6H), 1.91 – 1.78 (m, 12H),

1.76 – 1.64 (m, 12H). **^{13}C NMR (101 MHz, $CDCl_3$)** δ 159.5 (C_q), 157.7 (CH), 137.8 (C_q), 134.8 (C_q), 132.5 (C_q), 130.4 (CH), 129.9 (CH), 128.4 (CH), 126.1 (CH), 125.0 (C_q), 114.2 (CH), 76.4 (CH), 55.3 (CH_3), 40.2 (CH_2), 37.1 (CH_2), 36.8 (C_q), 29.0 (CH), 18.1 (CH_3). **^{19}F NMR (377 MHz, $CDCl_3$)** δ -151.89, -151.94. **IR** (ATR) $\tilde{\nu}$ = 3060, 3039, 2903, 2847, 1621, 1503, 1454, 1393, 1247, 1215, 1035, 757, 701 cm^{-1}. **HRMS** (ESI) m/z $(M–BF_4)^+$: calculated for $(C_{51}H_{59}N_2O_2)^+$: 731.4571, found: 731.4567. $[\alpha]_D^{20}$ = +163.0 (c = 0.66, $CHCl_3$).

(4*R*,5*R*)-1,3-Bis(5-(adamantan-1-yl)-2-methyl-4-phenoxyphenyl)-4,5-diphenyl-4,5-dihydro-1*H*-imidazol-3-ium tetrafluoroborate (L3)

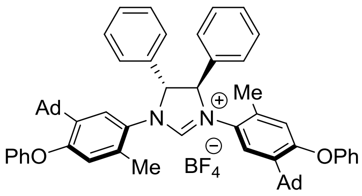

The **general procedure 2** was followed using 1-(5-bromo-4-methyl-2-phenoxyphenyl)adamantane (4.2 mmol, 1.66 g) to afford **L3** (0.76 g, 40% yield over 2 steps) as a pale yellow solid. **M.p.**: 204-205 °C. **^{1}H NMR (400 MHz, $CDCl_3$)** δ 8.73 (s, 1H), 7.52 – 7.38 (m, 10H), 7.34 (t, *J* = 7.8 Hz, 4H), 7.11 (t, *J* = 7.4 Hz, 2H), 7.03 (s, 2H), 6.95 (d, *J* = 8.0 Hz, 4H), 6.59 (s, 2H), 5.65 (s, 2H), 2.34 (s, 6H), 2.05 – 1.94 (m, 6H), 1.95 – 1.82 (m, 12H), 1.75 – 1.63 (m, 12H). **^{13}C NMR (101 MHz, $CDCl_3$)** δ 157.7 (CH), 157.4 (C_q), 156.9 (C_q), 140.3 (C_q), 134.4 (C_q), 132.4 (C_q), 130.5 (CH), 130.1 (CH), 129.9 (CH), 128.4 (CH), 127.5 (C_q), 127.1 (CH), 123.6 (CH), 122.0 (CH), 119.5 (CH), 76.3 (CH), 40.5 (CH_2), 36.9 (CH_2), 36.8 (C_q), 28.9 (CH), 17.8 (CH_3). **^{19}F NMR (377 MHz, $CDCl_3$)** δ -151.52, -151.57. **IR** (ATR) $\tilde{\nu}$ = 3060, 3039, 2904, 2848, 1624, 1597, 1488, 1454, 1214, 1054, 1020, 757, 702 cm^{-1}. **HRMS** (ESI) m/z $(M–BF_4)^+$: calculated for $(C_{61}H_{63}N_2O_2)^+$: 855.4884, found: 855.4881. $[\alpha]_D^{20}$ = +158.8 (c = 0.17, $CHCl_3$).

(4*R*,5*R*)-1,3-Bis(5-(adamantan-1-yl)-3-methoxy-2-methylphenyl)-4,5-diphenyl-4,5-dihydro-1*H*-imidazol-3-ium tetrafluoroborate (L4)

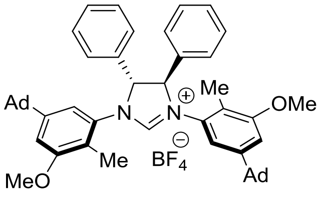

The **general procedure 2** was followed using 1-(3-bromo-5-methoxy-4-methylphenyl)adamantane (4.2 mmol, 1.40 g) to afford **L4** (0.64 g, 39% yield over 2 steps) as a yellow solid. **M.p.**: 155-158 °C. **^{1}H NMR (400 MHz, CDCl$_3$)** δ 9.49 (s, 1H), 7.56 – 7.40 (m, 8H), 7.37 (dd, *J* = 7.8, 1.8 Hz, 4H), 6.66 (d, *J* = 3.0 Hz, 2H), 5.56 (s, 2H), 3.72 (s, 6H), 2.35 (s, 6H), 2.05 – 1.92 (m, 6H), 1.86 – 1.74 (m, 12H), 1.75 – 1.61 (m, 12H). **^{13}C NMR (101 MHz, CDCl$_3$)** δ 159.0 (C$_q$), 152.5 (CH), 144.2 (C$_q$), 135.6 (C$_q$), 134.5 (C$_q$), 130.3 (CH), 130.3 (CH), 129.4 (C$_q$), 126.9 (CH), 122.6 (CH), 117.2 (CH), 75.1 (CH), 61.1 (CH$_3$), 41.3 (CH$_2$), 37.6 (C$_q$), 36.9 (CH$_2$), 29.1 (CH), 17.5 (CH$_3$). **^{19}F NMR (377 MHz, CDCl$_3$)** δ -150.58, -150.63. **IR** (ATR) $\tilde{\nu}$ = 3055, 3035, 2902, 2848, 1615, 1595, 1454, 1269, 1227, 1053, 1006, 755, 700 cm^{-1}. **HRMS** (ESI) m/z (M–BF$_4$)$^+$: calculated for $(C_{51}H_{59}N_2O_2)^+$: 731.4571, found: 731.4574. **$[\alpha]_D^{20}$** = +119.6 (c = 0.68, CHCl$_3$).

(4*R*,5*R*)-1,3-Bis(5-(adamantan-1-yl)-2-methoxyphenyl)-4,5-diphenyl-4,5-dihydro-1*H*-imidazol-3-ium tetrafluoroborate (L5)

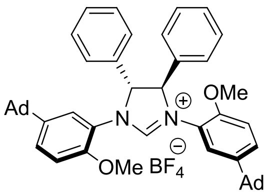

The **general procedure 1** was followed using 1-(3-bromo-4-methoxyphenyl)adamantane (4.2 mmol, 1.34 g) to afford **L5** (1.01 g, 64% yield over 2 steps) as a pale yellow solid. **M.p.**: 158-160 °C. **^{1}H NMR (400 MHz, CDCl$_3$)** δ 8.91 (s, 1H), 7.45 – 7.34 (m, 10H), 7.23 (dd, *J* = 8.7, 2.3 Hz, 2H), 7.12 (d, *J* = 2.4 Hz, 2H), 6.90 (d, *J* = 8.7 Hz, 2H), 5.76 (s, 2H), 3.96 (s, 6H), 2.08 – 1.95 (m, 6H), 1.75 – 1.62 (m, 24H). **^{13}C NMR (101 MHz, CDCl$_3$)** δ 158.1 (CH), 151.0 (C$_q$), 145.0 (C$_q$), 135.4 (C$_q$), 130.0 (CH), 129.7 (CH), 128.0 (CH), 126.8 (CH), 123.5 (CH), 122.5 (C$_q$), 111.8 (CH), 75.4 (CH), 56.4 (CH$_3$), 42.9 (CH$_2$), 36.7 (CH$_2$), 35.8 (C$_q$), 28.9 (CH). **^{19}F NMR (377 MHz, CDCl$_3$)** δ -152.38, -152.43. **IR** (ATR) $\tilde{\nu}$ = 3059, 3036, 2901, 2847, 1625, 1601, 1514, 1455, 1275, 1058, 1018, 758, 701 cm^{-1}. **HRMS** (ESI) m/z (M–BF$_4$)$^+$: calculated for $(C_{49}H_{55}N_2O_2)^+$: 703.4258, found: 703.4259. **$[\alpha]_D^{20}$** = +257.3 (c = 0.62, CHCl$_3$).

(4*R*,5*R*)-1,3-Bis(5-(adamantan-1-yl)-2-(benzyloxy)phenyl)-4,5-diphenyl-4,5-dihydro-1*H*-imidazol-3-ium tetrafluoroborate (L6)

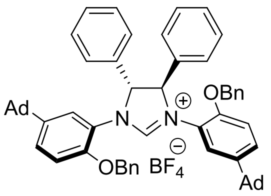

The **general procedure 1** was followed using 1-(4-(benzyloxy)-3-bromophenyl)adamantane (4.2 mmol, 1.66 g) to afford **L6** (0.60 g, 32% yield over 2 steps) as a white solid. **M.p.**: 128-130 °C. **^{1}H NMR (400 MHz, CDCl$_3$)** δ 8.88 (s, 1H), 7.51 – 7.43 (m, 4H), 7.43 – 7.33 (m, 6H), 7.31 – 7.26 (m, 2H), 7.24 (dd, *J* = 8.8, 2.5 Hz, 2H), 7.17 (t, *J* = 7.7 Hz, 4H), 7.12 – 7.07 (m, 4H), 6.98 (d, *J* = 8.8 Hz, 2H), 6.92 (d, *J* = 2.3 Hz, 2H), 5.55 (s, 2H), 5.15 (d, *J* = 10.7 Hz, 2H), 5.08 (d, *J* = 10.8 Hz, 2H), 2.07 – 1.97 (m, 6H), 1.81 – 1.63 (m, 24H). **^{13}C NMR (101 MHz, CDCl$_3$)** δ 158.8 (CH), 150.8 (C_q), 145.1 (C_q), 136.2 (C_q), 135.3 (C_q), 129.8 (CH), 129.4 (CH), 129.0 (CH), 128.6 (CH), 128.6 (CH), 128.2 (CH), 127.2 (CH), 124.7 (CH), 122.6 (C_q), 112.7 (CH), 75.5 (CH), 71.4 (CH_2), 42.9 (CH_2), 36.7 (CH_2), 35.7 (C_q), 28.9 (CH). **^{19}F NMR (377 MHz, CDCl$_3$)** δ -151.97, -152.02. **IR** (ATR) $\tilde{\nu}$ = 3064, 3034, 2901, 2847, 1626, 1602, 1513, 1454, 1276, 1059, 756, 699 cm^{-1}. **HRMS** (ESI) m/z $(M–BF_4)^+$: calculated for $(C_{61}H_{63}N_2O_2)^+$: 855.4884, found: 855.4880. **$[\alpha]_D^{20}$** = +133.2 (c = 0.28, $CHCl_3$).

(4*R*,5*R*)-1,3-Bis(5-(adamantan-1-yl)-2-isopropoxyphenyl)-4,5-diphenyl-4,5-dihydro-1*H*-imidazol-3-ium tetrafluoroborate (L7)

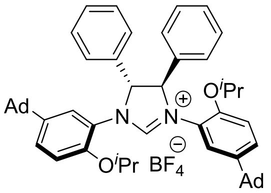

The **general procedure 1** was followed using 1-(3-bromo-4-isopropoxyphenyl)adamantane (4.2 mmol, 1.46 g) to afford **L7** (1.02 g, 60% yield over 2 steps) as a white solid. **M.p.**: 139-141 °C. **^{1}H NMR (400 MHz, CDCl$_3$)** δ 8.78 (s, 1H), 7.47 – 7.36 (m, 10H), 7.21 (dd, *J* = 8.7, 2.4 Hz, 2H), 7.13 (d, *J* = 2.4 Hz, 2H), 6.87 (d, *J* = 8.8 Hz, 2H), 5.81 (s, 2H), 4.72 (hept, *J* = 6.1 Hz, 2H), 2.06 – 1.98 (m, 6H), 1.76 – 1.62 (m, 24H), 1.48 (d, *J* = 6.0 Hz, 6H), 1.41 (d, *J* = 6.0 Hz, 6H). **^{13}C NMR (101 MHz, CDCl$_3$)** δ 157.7 (CH), 149.4 (C_q), 144.7 (C_q), 136.0 (C_q), 130.0 (CH), 129.6 (CH), 128.1 (CH), 127.0 (CH), 124.8 (CH), 122.8 (C_q), 113.0 (CH), 75.2 (CH), 71.1 (CH), 42.9 (CH_2), 36.7 (CH_2),

35.7 (C_q), 28.9 (CH), 22.5 (CH_3), 22.4 (CH_3). **^{19}F NMR (377 MHz, $CDCl_3$)** δ -152.07, -152.12. **IR** (ATR) $\tilde{\nu}$ = 3065, 3033, 2974, 2847, 1626, 1602, 1511, 1454, 1275, 1057, 757, 700 cm^{-1}. **HRMS** (ESI) m/z $(M–BF_4)^+$: calculated for $(C_{53}H_{63}N_2O_2)^+$: 759.4884, found: 759.4882. **$[\alpha]_D^{20}$** = +271.2 (c = 0.81, $CHCl_3$).

(4*R*,5*R*)-1,3-Bis(5-(*tert*-butyl)-2-methoxyphenyl)-4,5-diphenyl-4,5-dihydro-1*H*-imidazol-3-ium tetrafluoroborate (L8)

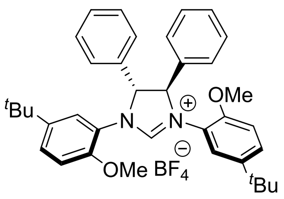

The **general procedure 1** was followed using 2-bromo-4-(*tert*-butyl)-1-methoxybenzene (4.2 mmol, 1.02 g) to afford **L8** (0.99 g, 78% yield over 2 steps) as a white solid. **M.p.**: 90-93 °C. **^{1}H NMR (400 MHz, $CDCl_3$)** δ 8.88 (s, 1H), 7.44 – 7.34 (m, 10H), 7.26 (dd, *J* = 8.7, 2.4 Hz, 2H), 7.16 (d, *J* = 2.4 Hz, 2H), 6.89 (d, *J* = 8.7 Hz, 2H), 5.75 (s, 2H), 3.96 (s, 6H), 1.14 (s, 18H). **^{13}C NMR (101 MHz, $CDCl_3$)** δ 158.1 (CH), 151.0 (C_q), 144.7 (C_q), 135.2 (C_q), 130.1 (CH), 129.6 (CH), 128.0 (CH), 127.2 (CH), 124.1 (CH), 122.4 (C_q), 111.7 (CH), 75.5 (CH), 56.4 (CH_3), 34.3 (C_q), 31.2 (CH_3). **^{19}F NMR (377 MHz, $CDCl_3$)** δ -152.38, -152.43. **IR** (ATR) $\tilde{\nu}$ = 3062, 3035, 1625, 1603, 1511, 1276, 1057, 758, 702 cm^{-1}. **HRMS** (ESI) m/z $(M–BF_4)^+$: calculated for $(C_{37}H_{43}N_2O_2)^+$: 547.3319, found: 547.3320. **$[\alpha]_D^{20}$** = +311.8 (c = 0.73, $CHCl_3$).

(4*R*,5*R*)-1,3-Bis(2-methoxy-5-methylphenyl)-4,5-diphenyl-4,5-dihydro-1*H*-imidazol-3-ium tetrafluoroborate (L9)

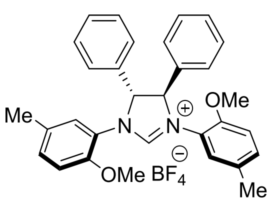

The **general procedure 1** was followed using 2-bromo-1-methoxy-4-methylbenzene (4.2 mmol, 0.84 g) to afford **L9** (0.80 g, 73% yield over 2 steps) as a pale yellow solid. **M.p.**: 89-91 °C. **^{1}H NMR (400 MHz, $CDCl_3$)** δ 8.90 (s, 1H), 7.45 – 7.33 (m, 10H), 7.12 (d, *J* = 2.0 Hz, 2H), 7.05 (dd, *J* = 8.4, 1.6 Hz, 2H), 6.82 (d, *J* = 8.5 Hz, 2H), 5.77 (s, 2H), 3.91 (s, 6H), 2.18 (s, 6H). **^{13}C NMR (101 MHz, $CDCl_3$)** δ 158.2 (CH), 151.1 (C_q), 135.5 (C_q), 131.5 (C_q), 131.2 (CH), 130.0 (CH), 129.7 (CH),

127.7 (CH), 127.1 (CH), 122.5 (C_q), 112.0 (CH), 75.3 (CH), 56.3 (CH_3), 20.4 (CH_3). **^{19}F NMR (377 MHz, $CDCl_3$)** δ -152.40, -152.46. **IR** (ATR) $\tilde{\nu}$ = 3065, 3034, 1626, 1605, 1512, 1274, 1059, 760, 700 cm^{-1}. **HRMS** (ESI) m/z $(M–BF_4)^+$: calculated for $(C_{31}H_{31}N_2O_2)^+$: 463.2380, found: 463.2380. $[\alpha]_D^{20}$ = +367.7 (c = 0.68, $CHCl_3$).

4. Synthesis of indole substrates

General Procedure 3: synthesis of indole substrates

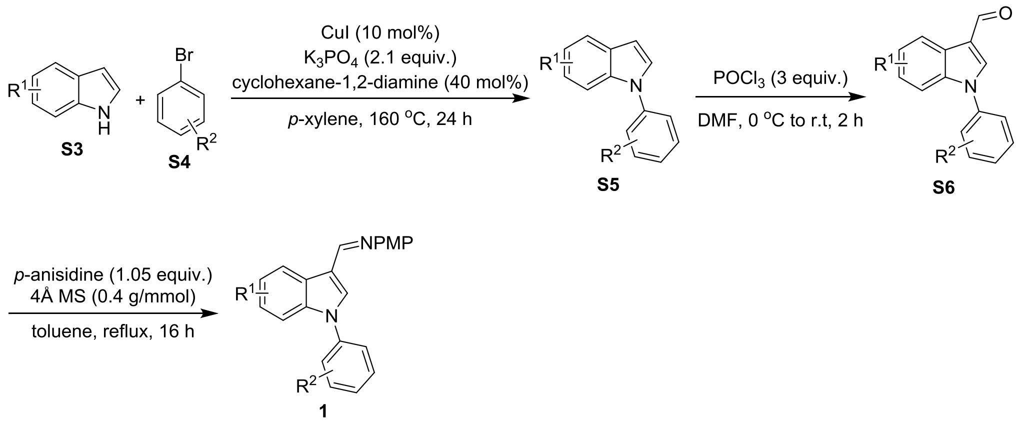

To a nitrogen purged round bottom flask equipped with a magnetic stirring bar, indole derivative **S3** (1.0 equiv., 10.0 mmol), CuI (10 mol%., 1.0 mmol, 189.8 mg) and potassium phosphate (2.1 equiv., 21.0 mmol, 4.45 g) were added. The flask was evacuated and refilled with nitrogen three times. Subsequently, corresponding aryl bromide **S4** (1.5 equiv., 15.0 mmol), cyclohexane-1,2-diamine (40 mol%, 4.0 mmol, 490 μL) and *p*-xylene (0.8 M, 12.5 mL) were added. The mixture was heated to 160 °C and stirred for 24 hours. After cooling to room temperature, the mixture was quenched with saturated NH_4Cl aqueous solution. The resulting solution was extracted with ethyl acetate. The combined organic layers were dried over Na_2SO_4, filtered and concentrated *in vacuo*. The residue was purified by column chromatography on silica gel (*n*-hexane 100% to *n*-hexane: dichloromethane = 20:1) to afford the desired product **S5**.

To a stirred solution of indole derivative **S5** in DMF (0.1 M, 100.0 mL) was added dropwise $POCl_3$ (3.0 equiv., 30.0 mmol, 2.80 mL) at 0 °C. The resulting mixture was stirred for 2 hours at room temperature. After 2 hours, the reaction mixture was carefully quenched with a mixture of ice and water. Then, NaOH pellets were added until reaching pH 8-9. Et_2O was added and the phases were separated. The aqueous phase was extracted 3 times with Et_2O and the combined organic phase was washed 5 times with water, then with brine, dried over Na_2SO_4, filtered and concentrated *in vacuo* to give the expected aldehyde **S6** which was used without further purification.

In a flame-dried round bottom flask under nitrogen, were added aldehyde **S6**, *p*-anisidine (1.05 equiv., 10.5 mmol, 1.29 g) and 4Å molecular sieves (0.4 g/mmol, 4.0 g). Then, dry toluene (0.5 M, 20.0 mL) was added and the reaction mixture was refluxed for 16 hours. After completion of the reaction, the reaction mixture was cooled to room temperature, filtered through a pad of Celite

(eluted with EtOAc) and concentrated *in vacuo*. The residue was purified by recrystallization (*n*-hexane/ethyl acetate) or flash chromatography on silica gel (*n*-hexane: ethyl acetate = 10:1 containing 0.5 v% NEt_3) to afford desired product **1**.

(*E*)-1-(1-(2-Isopropylphenyl)-1*H*-indol-3-yl)-*N*-(4-methoxyphenyl)methanimine (1a)

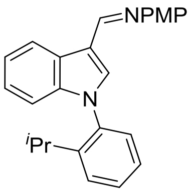

The **general procedure 3** was followed using 1*H*-indole (10.0 mmol, 1.17 g) and 1-bromo-2-isopropylbenzene (15.0 mmol, 2.97 g) to afford **1a** (2.14 g, 58% yield over 3 steps) as a white solid. **M.p.**: 120-122 °C. **^{1}H NMR (300 MHz, $CDCl_3$)** δ 8.73 (s, 1H), 8.59 (d, *J* = 7.7 Hz, 1H), 7.59 (s, 1H), 7.56 – 7.45 (m, 2H), 7.39 – 7.21 (m, 6H), 7.02 (d, *J* = 7.9 Hz, 1H), 6.95 (d, *J* = 8.8 Hz, 2H), 3.85 (s, 3H), 2.68 (hept, *J* = 6.9 Hz, 1H), 1.15 (d, *J* = 6.9 Hz, 3H), 1.09 (d, *J* = 6.9 Hz, 3H). **^{13}C NMR (75 MHz, $CDCl_3$)** δ 157.6 (C_q), 153.0 (CH), 147.0 (C_q), 146.8 (C_q), 139.4 (C_q), 136.0 (C_q), 134.2 (CH), 129.7 (CH), 128.6 (CH), 127.1 (CH), 126.9 (CH), 125.6 (C_q), 123.9 (CH), 122.4 (CH), 122.1 (CH), 122.0 (CH), 116.5 (C_q), 114.5 (CH), 110.8 (CH), 55.7 (CH_3), 28.0 (CH), 24.7 (CH_3), 23.7 (CH_3). **IR** (ATR) $\tilde{\nu}$ = 2963, 2870, 2834, 1620, 1501, 1461, 1311, 1242, 741 cm^{-1}. **HRMS** (ESI) m/z $(M+H)^+$: calculated for $(C_{25}H_{25}N_2O)^+$: 369.1961, found: 369.1959.

(*E*)-*N*-(4-Methoxyphenyl)-1-(1-(*o*-tolyl)-1*H*-indol-3-yl)methanimine (1b)

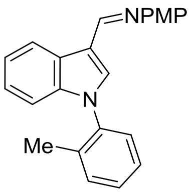

The **general procedure 3** was followed using 1*H*-indole (10.0 mmol, 1.17 g) and 1-bromo-2-methylbenzene (15.0 mmol, 2.55 g) to afford **1b** (1.56 g, 46% yield over 3 steps) as a white solid. **M.p.**: 93-95 °C. **^{1}H NMR (400 MHz, $CDCl_3$)** δ 8.73 (s, 1H), 8.59 (d, *J* = 7.7 Hz, 1H), 7.61 (s, 1H), 7.45 – 7.40 (m, 2H), 7.39 – 7.24 (m, 6H), 7.04 (d, *J* = 8.0 Hz, 1H), 6.95 (d, *J* = 8.8 Hz, 2H), 3.84 (s, 3H), 2.10 (s, 3H). **^{13}C NMR (101 MHz, $CDCl_3$)** δ 157.6 (C_q), 152.9 (CH), 146.7 (C_q), 138.4 (C_q), 137.5 (C_q), 135.8 (C_q), 133.6 (CH), 131.5 (CH), 129.0 (CH), 128.1 (CH), 127.1 (CH), 125.8 (C_q), 123.8 (CH), 122.5 (CH), 122.0 (CH), 122.0 (CH), 116.6 (C_q), 114.5 (CH), 110.9 (CH), 55.7 (CH_3),

17.7 (CH_3). **IR** (ATR) $\tilde{\nu}$ = 2954, 2834, 1620, 1501, 1462, 1312, 1241, 747 cm^{-1}. **HRMS** (ESI) m/z $(M+H)^+$: calculated for $(C_{23}H_{21}N_2O)^+$: 341.1648, found: 341.1654.

(*E*)-1-(1-(2-Ethylphenyl)-1*H*-indol-3-yl)-*N*-(4-methoxyphenyl)methanimine (1c)

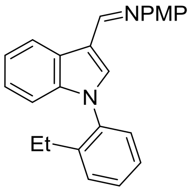

The **general procedure 3** was followed using 1*H*-indole (10.0 mmol, 1.17 g) and 1-bromo-2-ethylbenzene (15.0 mmol, 2.76 g) to afford **1c** (1.84 g, 52% yield over 3 steps) as a white solid. **M.p.**: 111-113 °C. **^{1}H NMR (400 MHz, $CDCl_3$)** δ 8.72 (s, 1H), 8.60 (d, *J* = 7.6 Hz, 1H), 7.59 (s, 1H), 7.50 – 7.41 (m, 2H), 7.38 – 7.21 (m, 6H), 7.03 (d, *J* = 7.9 Hz, 1H), 6.95 (d, *J* = 8.8 Hz, 2H), 3.83 (s, 3H), 2.42 (qd, *J* = 7.5, 5.2 Hz, 2H), 1.04 (t, *J* = 7.5 Hz, 3H). **^{13}C NMR (101 MHz, $CDCl_3$)** δ 157.6 (C_q), 152.9 (CH), 146.7 (C_q), 142.0 (C_q), 138.9 (C_q), 136.8 (C_q), 134.0 (CH), 129.8 (CH), 129.3 (CH), 128.4 (CH), 127.1 (CH), 125.7 (C_q), 123.8 (CH), 122.5 (CH), 122.0 (CH), 122.0 (CH), 116.6 (C_q), 114.4 (CH), 110.9 (CH), 55.6 (CH_3), 24.2 (CH_2), 15.1 (CH_3). **IR** (ATR) $\tilde{\nu}$ = 2967, 2834, 1621, 1501, 1461, 1312, 1242, 747 cm^{-1}. **HRMS** (ESI) m/z $(M+H)^+$: calculated for $(C_{24}H_{23}N_2O)^+$: 355.1805, found: 355.1807.

(*E*)-1-(1-([1,1'-Biphenyl]-2-yl)-1*H*-indol-3-yl)-*N*-(4-methoxyphenyl)methanimine (1d)

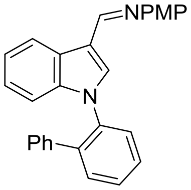

The **general procedure 3** was followed using 1*H*-indole (10.0 mmol, 1.17 g) and 2-bromo-1,1'-biphenyl (15.0 mmol, 3.48 g) to afford **1d** (0.88 g, 22% yield over 3 steps) as a white solid. **M.p.**: 116-118 °C. **^{1}H NMR (400 MHz, $CDCl_3$)** δ 8.64 (d, *J* = 7.9 Hz, 1H), 8.55 (s, 1H), 7.70 – 7.64 (m, 1H), 7.64 – 7.49 (m, 3H), 7.39 – 7.30 (m, 1H), 7.31 – 7.27 (m, 4H), 7.27 – 7.25 (m, 1H), 7.24 – 7.20 (m, 3H), 7.17 – 7.09 (m, 2H), 6.97 (d, *J* = 8.8 Hz, 2H), 3.86 (s, 3H). **^{13}C NMR (101 MHz, $CDCl_3$)** δ 157.5 (C_q), 152.8 (CH), 146.4 (C_q), 139.1 (C_q), 138.3 (C_q), 138.1 (C_q), 135.9 (C_q), 134.5 (CH), 131.7 (CH), 128.8 (CH), 128.6 (CH), 128.5 (CH), 128.2 (CH), 128.1 (CH), 127.6 (CH), 125.8 (C_q), 123.8 (CH), 122.6 (CH), 122.0 (CH), 122.0 (CH), 116.8 (C_q), 114.3 (CH), 110.9 (CH),

55.5 (CH_3). **IR** (ATR) $\tilde{\nu}$ = 3055, 2834, 1620, 1501, 1482, 1241, 744 cm^{-1}. **HRMS** (ESI) m/z $(M+H)^+$: calculated for $(C_{28}H_{23}N_2O)^+$: 403.1805, found: 403.1800.

(*E*)-*N*-(4-Methoxyphenyl)-1-(1-(2-(trifluoromethoxy)phenyl)-1*H*-indol-3-yl)methanimine (1e)

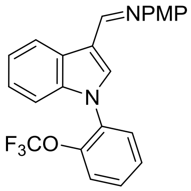

The **general procedure 3** was followed using 1*H*-indole (10.0 mmol, 1.17 g) and 1-bromo-2-(trifluoromethoxy)benzene (15.0 mmol, 3.60 g) to afford **1e** (0.62 g, 15% yield over 3 steps) as a yellow solid. **M.p.**: 80-82 °C. **^{1}H NMR (400 MHz, $CDCl_3$)** δ 8.73 (s, 1H), 8.67 – 8.60 (m, 1H), 7.66 (s, 1H), 7.61 – 7.45 (m, 4H), 7.40 – 7.20 (m, 5H), 6.96 (d, *J* = 8.8 Hz, 2H), 3.85 (s, 3H). **^{13}C NMR (101 MHz, $CDCl_3$)** δ 157.7 (C_q), 152.7 (CH), 146.5 (C_q), 144.2 (q, *J* = 1.6 Hz, C_q), 138.1 (C_q), 133.7 (CH), 131.5 (C_q), 129.5 (CH), 129.0 (CH), 128.0 (CH), 126.1 (C_q), 124.2 (CH), 122.8 (CH), 122.6 (q, *J* = 1.2 Hz, CH), 122.4 (CH), 122.1 (CH), 120.3 (q, *J* = 259.4 Hz, C_q), 117.7 (C_q), 114.5 (CH), 110.6 (CH), 55.6 (CH_3). **^{19}F NMR (377 MHz, $CDCl_3$)** δ -57.97. **IR** (ATR) $\tilde{\nu}$ = 1622, 1502, 1242, 1208, 1188, 1165, 747 cm^{-1}. **HRMS** (ESI) m/z $(M+H)^+$: calculated for $(C_{23}H_{18}F_3N_2O_2)^+$: 411.1315, found: 411.1309.

(*E*)-1-(1-(2-Chlorophenyl)-1*H*-indol-3-yl)-*N*-(4-methoxyphenyl)methanimine (1f)

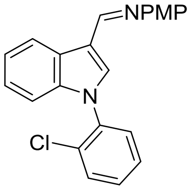

The **general procedure 3** was followed using 1*H*-indole (10.0 mmol, 1.17 g) and 1-bromo-2-chlorobenzene (15.0 mmol, 2.85 g) to afford **1f** (1.86 g, 52% yield over 3 steps) as a white solid. **M.p.**: 115-117 °C. **^{1}H NMR (400 MHz, $CDCl_3$)** δ 8.73 (s, 1H), 8.65 – 8.55 (m, 1H), 7.69 – 7.58 (m, 2H), 7.52 – 7.42 (m, 3H), 7.38 – 7.22 (m, 4H), 7.16 – 7.10 (m, 1H), 7.00 – 6.91 (m, 2H), 3.85 (s, 3H). **^{13}C NMR (101 MHz, $CDCl_3$)** δ 157.7 (C_q), 152.7 (CH), 146.6 (C_q), 138.2 (C_q), 136.1 (C_q), 133.7 (CH), 131.9 (C_q), 131.1 (CH), 129.9 (CH), 129.5 (CH), 128.0 (CH), 125.9 (C_q), 124.0 (CH), 122.7 (CH), 122.3 (CH), 122.1 (CH), 117.3 (C_q), 114.5 (CH), 110.9 (CH), 55.7 (CH_3). **IR** (ATR) $\tilde{\nu}$ =

1620, 1502, 1241, 1034, 833, 749 cm^{-1}. **HRMS** (ESI) m/z $(M+H)^+$: calculated for $(C_{22}H_{18}ClN_2O)^+$: 361.1102, found: 361.1109.

(*E*)-*N*-(4-Methoxyphenyl)-1-(1-(naphthalen-1-yl)-1*H*-indol-3-yl)methanimine (1g)

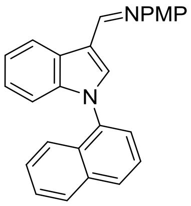

The **general procedure 3** was followed using 1*H*-indole (10.0 mmol, 1.17 g) and 1-bromonaphthalene (15.0 mmol, 3.09 g) to afford **1g** (2.82 g, 75% yield over 3 steps) as a white solid. **M.p.**: 140-142 °C. **^{1}H NMR (400 MHz, $CDCl_3$)** δ 8.73 (s, 1H), 8.65 (d, *J* = 7.9 Hz, 1H), 7.96 (t, *J* = 7.3 Hz, 2H), 7.71 (s, 1H), 7.61 – 7.49 (m, 3H), 7.46 – 7.36 (m, 2H), 7.32 (ddd, *J* = 8.1, 7.0, 1.0 Hz, 1H), 7.27 (d, *J* = 8.8 Hz, 2H), 7.24 – 7.15 (m, 1H), 6.99 (d, *J* = 8.2 Hz, 1H), 6.94 (d, *J* = 8.8 Hz, 2H), 3.81 (s, 3H). **^{13}C NMR (101 MHz, $CDCl_3$)** δ 157.6 (C_q), 152.8 (CH), 146.6 (C_q), 139.4 (C_q), 135.1 (C_q), 134.7 (CH), 134.5 (C_q), 130.3 (C_q), 129.3 (CH), 128.5 (CH), 127.4 (CH), 127.0 (CH), 125.9 (C_q), 125.6 (CH), 125.3 (CH), 123.9 (CH), 123.1 (CH), 122.6 (CH), 122.2 (CH), 122.1 (CH), 116.9 (C_q), 114.4 (CH), 111.2 (CH), 55.6 (CH_3). **IR** (ATR) $\tilde{\nu}$ = 3055, 2833, 1620, 1500, 1242, 776, 748 cm^{-1}. **HRMS** (ESI) m/z $(M+H)^+$: calculated for $(C_{26}H_{21}N_2O)^+$: 377.1648, found: 377.1646.

(*E*)-1-(4-Fluoro-1-(2-isopropylphenyl)-1*H*-indol-3-yl)-*N*-(4-methoxyphenyl)methanimine (1h)

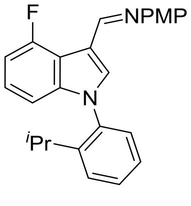

The **general procedure 3** was followed using 4-fluoro-1*H*-indole (10.0 mmol, 1.35 g) and 1-bromo-2-isopropylbenzene (15.0 mmol, 2.97 g) to afford **1h** (1.39 g, 36% yield over 3 steps) as a yellow solid. **M.p.**: 115-117 °C. **^{1}H NMR (400 MHz, $CDCl_3$)** δ 9.02 (s, 1H), 7.96 (s, 1H), 7.56 – 7.49 (m, 2H), 7.38 – 7.29 (m, 1H), 7.29 – 7.23 (m, 3H), 7.12 (td, *J* = 8.1, 5.0 Hz, 1H), 6.99 – 6.90 (m, 3H), 6.80 (d, *J* = 8.2 Hz, 1H), 3.84 (s, 3H), 2.66 (hept, *J* = 6.9 Hz, 1H), 1.16 (d, *J* = 6.9 Hz, 3H), 1.09 (d, *J* = 6.8 Hz, 3H). **^{13}C NMR (101 MHz, $CDCl_3$)** δ 157.9 (C_q), 157.2 (d, *J* = 247.5 Hz, C_q), 153.1 (d, *J* = 3.1 Hz, CH), 146.7 (C_q), 145.9 (C_q), 141.1 (d, *J* = 11.4 Hz, C_q), 135.7 (C_q), 130.0 (CH), 129.6 (CH), 128.5 (CH), 127.1 (CH), 127.0 (CH), 123.4 (d, *J* = 7.8 Hz, CH), 122.2 (CH), 115.8 (d, *J* = 20.6 Hz,

C_q), 114.9 (d, J = 4.3 Hz, C_q), 114.5 (CH), 107.6 (d, J = 3.6 Hz, CH), 106.9 (d, J = 19.5 Hz, CH), 55.6 (CH_3), 28.0 (CH), 24.7 (CH_3), 23.7 (CH_3). **^{19}F NMR (377 MHz, $CDCl_3$)** δ -119.58. **IR** (ATR) $\tilde{\nu}$ = 2963, 2927, 2869, 2834, 1615, 1502, 1298, 1240, 1166, 1037, 830, 780, 757, 735 cm^{-1}. **HRMS** (ESI) m/z $(M+H)^+$: calculated for $(C_{25}H_{24}FN_2O)^+$: 387.1867, found: 387.1868.

(*E*)-1-(5-Fluoro-1-(2-isopropylphenyl)-1*H*-indol-3-yl)-*N*-(4-methoxyphenyl)methanimine (1i)

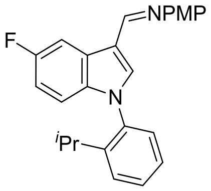

The **general procedure 3** was followed using 5-fluoro-1*H*-indole (10.0 mmol, 1.35 g) and 1-bromo-2-isopropylbenzene (15.0 mmol, 2.97 g) to afford **1i** (1.41 g, 37% yield over 3 steps) as a yellow solid. **M.p.**: 142-144 °C. **^{1}H NMR (400 MHz, $CDCl_3$)** δ 8.69 (s, 1H), 8.30 (d, J = 9.6 Hz, 1H), 7.58 (s, 1H), 7.52 (d, J = 4.1 Hz, 2H), 7.39 – 7.30 (m, 1H), 7.27 (d, J = 8.4 Hz, 3H), 7.04 – 6.87 (m, 4H), 3.84 (s, 3H), 2.63 (hept, J = 7.0 Hz, 1H), 1.15 (d, J = 6.8 Hz, 3H), 1.09 (d, J = 6.8 Hz, 3H). **^{13}C NMR (101 MHz, $CDCl_3$)** δ 159.4 (d, J = 237.2 Hz, C_q), 157.7 (C_q), 152.5 (CH), 147.0 (C_q), 146.4 (C_q), 136.0 (C_q), 135.8 (C_q), 135.4 (CH), 129.9 (CH), 128.5 (CH), 127.2 (CH), 127.0 (CH), 126.0 (d, J = 11.1 Hz, C_q), 122.0 (CH), 116.5 (d, J = 4.7 Hz, C_q), 114.5 (CH), 112.3 (d, J = 26.5 Hz, CH), 111.6 (d, J = 9.7 Hz, CH), 107.9 (d, J = 24.8 Hz, CH), 55.7 (CH_3), 28.0 (CH), 24.7 (CH_3), 23.7 (CH_3). **^{19}F NMR (377 MHz, $CDCl_3$)** δ -121.90. **IR** (ATR) $\tilde{\nu}$ = 2964, 2869, 1621, 1501, 1461, 1242, 1176, 831, 761 cm^{-1}. **HRMS** (ESI) m/z $(M+H)^+$: calculated for $(C_{25}H_{24}FN_2O)^+$: 387.1867, found: 387.1868.

(*E*)-1-(1-(2-Isopropylphenyl)-5-Methyl-1*H*-indol-3-yl)-*N*-(4-methoxyphenyl)methanimine (1j)

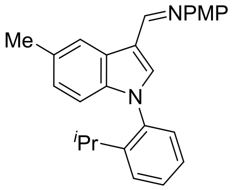

The **general procedure 3** was followed using 5-methyl-1*H*-indole (10.0 mmol, 1.31 g) and 1-bromo-2-isopropylbenzene (15.0 mmol, 2.97 g) to afford **1j** (2.36 g, 62% yield over 3 steps) as a pale yellow solid. **M.p.**: 153-155 °C. **^{1}H NMR (400 MHz, $CDCl_3$)** δ 8.70 (s, 1H), 8.37 (s, 1H), 7.56 – 7.46 (m, 3H), 7.37 – 7.21 (m, 4H), 7.09 (d, J = 8.3 Hz, 1H), 6.95 (d, J = 8.7 Hz, 2H), 6.91 (d, J = 8.4 Hz, 1H), 3.85 (s, 3H), 2.69 (hept, J = 6.8 Hz, 1H), 2.53 (s, 3H), 1.15 (d, J = 6.8 Hz, 3H), 1.09 (d, J = 6.8 Hz,

3H). **^{13}C NMR (101 MHz, $CDCl_3$)** δ 157.5 (C_q), 153.3 (CH), 146.9 (C_q), 146.9 (C_q), 137.8 (C_q), 136.2 (C_q), 134.4 (CH), 131.6 (C_q), 129.6 (CH), 128.6 (CH), 127.0 (CH), 126.8 (CH), 125.8 (C_q), 125.4 (CH), 122.0 (CH), 122.0 (CH), 116.1 (C_q), 114.4 (CH), 110.5 (CH), 55.7 (CH_3), 28.0 (CH), 24.7 (CH_3), 23.7 (CH_3), 21.7 (CH_3). **IR** (ATR) $\tilde{\nu}$ = 2965, 2868, 2833, 1620, 1500, 1242, 1182, 833, 796, 762 cm^{-1}. **HRMS** (ESI) m/z $(M+H)^+$: calculated for $(C_{26}H_{27}N_2O)^+$: 383.2118, found: 383.2122.

(*E*)-1-(1-(2-Isopropylphenyl)-5-methoxy-1*H*-indol-3-yl)-*N*-(4-methoxyphenyl)methanimine (1k)

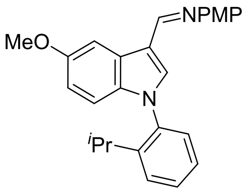

The **general procedure 3** was followed using 5-methoxy-1*H*-indole (10.0 mmol, 1.47 g) and 1-bromo-2-isopropylbenzene (15.0 mmol, 2.97 g) to afford **1k** (2.88 g, 72% yield over 3 steps) as a pale yellow solid. **M.p.**: 56-58 °C. **^{1}H NMR (400 MHz, $CDCl_3$)** δ 8.69 (s, 1H), 8.12 (s, 1H), 7.57 – 7.44 (m, 3H), 7.37 – 7.16 (m, 4H), 6.98 – 6.87 (m, 4H), 3.93 (s, 3H), 3.81 (s, 3H), 2.66 (hept, *J* = 6.9 Hz, 1H), 1.13 (d, *J* = 6.8 Hz, 3H), 1.08 (d, *J* = 6.8 Hz, 3H). **^{13}C NMR (101 MHz, $CDCl_3$)** δ 157.5 (C_q), 156.0 (C_q), 153.1 (CH), 146.8 (C_q), 146.7 (C_q), 136.0 (C_q), 134.6 (CH), 134.5 (C_q), 129.6 (CH), 128.5 (CH), 127.0 (CH), 126.8 (CH), 126.1 (C_q), 122.0 (CH), 116.1 (C_q), 114.4 (CH), 114.1 (CH), 111.6 (CH), 103.9 (CH), 55.9 (CH_3), 55.6 (CH_3), 27.9 (CH), 24.7 (CH_3), 23.6 (CH_3). **IR** (ATR) $\tilde{\nu}$ = 2962, 2869, 2833, 1618, 1500, 1462, 1243, 1176, 1033, 831, 794, 761 cm^{-1}. **HRMS** (ESI) m/z $(M+H)^+$: calculated for $(C_{26}H_{27}N_2O_2)^+$: 399.2067, found: 399.2074.

(*E*)-1-(5-(Benzyloxy)-1-(2-isopropylphenyl)-1*H*-indol-3-yl)-*N*-(4-methoxyphenyl)methanimine (1l)

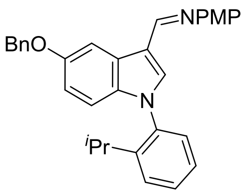

The **general procedure 3** was followed using 5-(benzyloxy)-1*H*-indole (10.0 mmol, 2.23 g) and 1-bromo-2-isopropylbenzene (15.0 mmol, 2.97 g) to afford **1l** (1.23 g, 26% yield over 3 steps) as a yellow solid. **M.p.**: 124-126 °C. **^{1}H NMR (400 MHz, $CDCl_3$)** δ 8.70 (s, 1H), 8.22 (s, 1H), 7.57 – 7.45 (m, 5H), 7.40 (t, *J* = 7.4 Hz, 2H), 7.33 (t, *J* = 6.9 Hz, 2H), 7.27 (d, *J* = 7.5 Hz, 3H), 7.02 – 6.86 (m,

4H), 5.20 (s, 2H), 3.85 (s, 3H), 2.68 (hept, *J* = 6.9 Hz, 1H), 1.15 (d, *J* = 6.8 Hz, 3H), 1.09 (d, *J* = 6.8 Hz, 3H). **^{13}C NMR (101 MHz, CDCl$_3$)** δ 157.5 (C_q), 155.3 (C_q), 153.1 (CH), 147.0 (C_q), 146.8 (C_q), 137.7 (C_q), 136.1 (C_q), 134.7 (C_q), 134.7 (CH), 129.7 (CH), 128.7 (CH), 128.5 (CH), 128.0 (CH), 127.9 (CH), 127.1 (CH), 126.9 (CH), 126.1 (C_q), 122.1 (CH), 116.3 (C_q), 114.8 (CH), 114.5 (CH), 111.7 (CH), 105.4 (CH), 70.9 (CH_2), 55.7 (CH_3), 28.0 (CH), 24.8 (CH_3), 23.7 (CH_3). **IR** (ATR) $\tilde{\nu}$ = 2963, 2868, 2834, 1618, 1500, 1462, 1243, 1180, 1033, 831, 795, 761 cm^{-1}. **HRMS** (ESI) m/z $(M+H)^+$: calculated for $(C_{32}H_{31}N_2O_2)^+$: 475.2380, found: 475.2375.

(*E*)-1-(6-Chloro-1-(2-isopropylphenyl)-1*H*-indol-3-yl)-*N*-(4-methoxyphenyl)methanimine (1m)

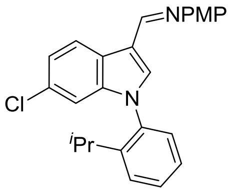

The **general procedure 3** was followed using 6-chloro-1*H*-indole (10.0 mmol, 1.51 g) and 1-bromo-2-isopropylbenzene (15.0 mmol, 2.97 g) to afford **1m** (2.10 g, 52% yield over 3 steps) as a pale yellow solid. **M.p.**: 118-120 °C. **^{1}H NMR (400 MHz, CDCl$_3$)** δ 8.68 (s, 1H), 8.53 (d, *J* = 8.5 Hz, 1H), 7.55 (s, 1H), 7.53 (dd, *J* = 3.9, 0.9 Hz, 2H), 7.38 – 7.33 (m, 1H), 7.30 – 7.24 (m, 4H), 7.00 (d, *J* = 1.8 Hz, 1H), 6.95 (d, *J* = 8.9 Hz, 2H), 3.84 (s, 3H), 2.63 (hept, *J* = 6.9 Hz, 1H), 1.16 (d, *J* = 6.9 Hz, 3H), 1.10 (d, *J* = 6.9 Hz, 3H). **^{13}C NMR (101 MHz, CDCl$_3$)** δ 157.8 (C_q), 152.5 (CH), 146.9 (C_q), 146.4 (C_q), 139.8 (C_q), 135.5 (C_q), 134.8 (CH), 130.0 (CH), 129.9 (C_q), 128.5 (CH), 127.3 (CH), 127.1 (CH), 124.1 (C_q), 123.7 (CH), 122.7 (CH), 122.1 (CH), 116.6 (C_q), 114.5 (CH), 110.8 (CH), 55.7 (CH_3), 28.0 (CH), 24.7 (CH_3), 23.8 (CH_3). **IR** (ATR) $\tilde{\nu}$ = 2964, 2868, 2834, 1620, 1501, 1243, 1158, 832, 761 cm^{-1}. **HRMS** (ESI) m/z $(M+H)^+$: calculated for $(C_{25}H_{24}ClN_2O)^+$: 403.1572, found: 403.1581.

(*E*)-1-(1-(2-Isopropylphenyl)-6-methyl-1*H*-indol-3-yl)-*N*-(4-methoxyphenyl)methanimine (1n)

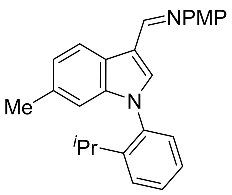

The **general procedure 3** was followed using 6-methyl-1*H*-indole (10.0 mmol, 1.31 g) and 1-bromo-2-isopropylbenzene (15.0 mmol, 2.97 g) to afford **1n** (2.18 g, 57% yield over 3 steps) as a pale yellow solid. **M.p.**: 52-54 °C. **^{1}H NMR (400 MHz, CDCl$_3$)** δ 8.70 (s, 1H), 8.45 (d, *J* = 8.1 Hz, 1H),

7.57 – 7.47 (m, 3H), 7.35 (t, *J* = 7.1 Hz, 1H), 7.31 – 7.24 (m, 3H), 7.15 (d, *J* = 8.1 Hz, 1H), 6.95 (d, *J* = 8.2 Hz, 2H), 6.80 (s, 1H), 3.85 (s, 3H), 2.70 (hept, *J* = 6.9 Hz, 1H), 2.43 (s, 3H), 1.16 (d, *J* = 6.8 Hz, 3H), 1.11 (d, *J* = 6.8 Hz, 3H). **^{13}C NMR (101 MHz, $CDCl_3$)** δ 157.5 (C_q), 153.1 (CH), 147.0 (C_q), 146.8 (C_q), 139.8 (C_q), 136.2 (C_q), 133.9 (C_q), 133.8 (CH), 129.6 (CH), 128.6 (CH), 127.1 (CH), 126.9 (CH), 123.7 (CH), 123.4 (C_q), 122.0 (CH), 116.5 (C_q), 114.4 (CH), 110.7 (CH), 55.7 (CH_3), 28.0 (CH), 24.7 (CH_3), 23.7 (CH_3), 22.0 (CH_3). **IR** (ATR) $\tilde{\nu}$ = 2964, 2867, 2833, 1620, 1501, 1453, 1295, 1241, 1182, 1032, 754 cm^{-1}. **HRMS** (ESI) m/z $(M+H)^+$: calculated for $(C_{26}H_{27}N_2O)^+$: 383.2118, found: 383.2117.

(*E*)-1-(7-Fluoro-1-(2-isopropylphenyl)-1*H*-indol-3-yl)-*N*-(4-methoxyphenyl)methanimine (1o)

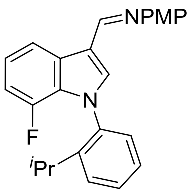

The **general procedure 3** was followed using 7-fluoro-1*H*-indole (10.0 mmol, 1.35 g) and 1-bromo-2-isopropylbenzene (15.0 mmol, 2.97 g) to afford **1o** (1.60 g, 41% yield over 3 steps) as a yellow oil. **^{1}H NMR (400 MHz, $CDCl_3$)** δ 8.71 (s, 1H), 8.39 (d, *J* = 8.0 Hz, 1H), 7.55 – 7.44 (m, 3H), 7.36 – 7.17 (m, 5H), 7.02 – 6.89 (m, 3H), 3.86 (s, 3H), 2.72 (hept, *J* = 7.0 Hz, 1H), 1.15 (d, *J* = 6.9 Hz, 6H). **^{13}C NMR (101 MHz, $CDCl_3$)** δ 157.8 (C_q), 152.5 (CH), 149.9 (d, *J* = 247.2 Hz, C_q), 146.6 (C_q), 146.4 (C_q), 137.6 (C_q), 135.4 (CH), 129.7 (CH), 129.5 (d, *J* = 3.8 Hz, C_q), 128.2 (d, *J* = 1.2 Hz, CH), 127.0 (d, *J* = 10.2 Hz, C_q), 126.5 (CH), 126.3 (CH), 122.4 (d, *J* = 6.2 Hz, CH), 122.1 (CH), 118.4 (d, *J* = 3.9 Hz, CH), 117.0 (C_q), 114.5 (CH), 109.7 (d, *J* = 16.9 Hz, CH), 55.7 (CH_3), 28.1 (CH), 24.7 (CH_3), 23.2 (CH_3). **^{19}F NMR (377 MHz, $CDCl_3$)** δ -133.54. **IR** (ATR) $\tilde{\nu}$ = 2963, 2869, 2834, 1621, 1494, 1290, 1238, 1183, 1033, 832, 787, 760, 736 cm^{-1}. **HRMS** (ESI) m/z $(M+H)^+$: calculated for $(C_{25}H_{24}FN_2O)^+$: 387.1867, found: 387.1861.

(*E*)-1-(1-(2-Isopropylphenyl)-1*H*-pyrrolo[2,3-*b*]pyridin-3-yl)-*N*-(4-methoxyphenyl)methanimine (1p)

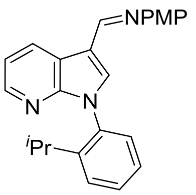

The **general procedure 3** was followed using 1*H*-pyrrolo[2,3-*b*]pyridine (10.0 mmol, 1.18 g) and 1-bromo-2-isopropylbenzene (15.0 mmol, 2.97 g) to afford **1p** (0.66 g, 18% yield over 3 steps) as a yellow solid. **M.p.**: 112-114 °C. **^{1}H NMR (400 MHz, CDCl$_3$)** δ 8.88 (d, *J* = 7.8 Hz, 1H), 8.68 (s, 1H), 8.39 (d, *J* = 4.5 Hz, 1H), 7.67 (s, 1H), 7.51 (d, *J* = 6.1 Hz, 2H), 7.39 – 7.22 (m, 5H), 6.96 (d, *J* = 8.2 Hz, 2H), 3.85 (s, 3H), 2.64 (hept, *J* = 6.9 Hz, 1H), 1.14 (d, *J* = 6.8 Hz, 6H). **^{13}C NMR (101 MHz, CDCl$_3$)** δ 157.9 (C_q), 152.5 (CH), 150.0 (C_q), 146.8 (C_q), 146.2 (C_q), 145.5 (CH), 135.2 (C_q), 134.4 (CH), 131.4 (CH), 129.8 (CH), 128.7 (CH), 127.0 (CH), 126.9 (CH), 122.1 (CH), 118.1 (CH), 118.1 (C_q), 114.9 (C_q), 114.5 (CH), 55.7 (CH_3), 28.4 (CH), 24.4 (CH_3), 23.6 (CH_3). **IR** (ATR) $\tilde{\nu}$ = 2963, 2868, 2834, 1620, 1502, 1428, 1288, 1238, 778, 757 cm^{-1}. **HRMS** (ESI) m/z (M+H)$^+$: calculated for $(C_{24}H_{24}N_3O)^+$: 370.1914, found: 370.1910.

5. General procedure for iron-catalyzed stereoselective C–H alkylation

General Procedure 4: iron-catalyzed stereoselective C–H alkylation

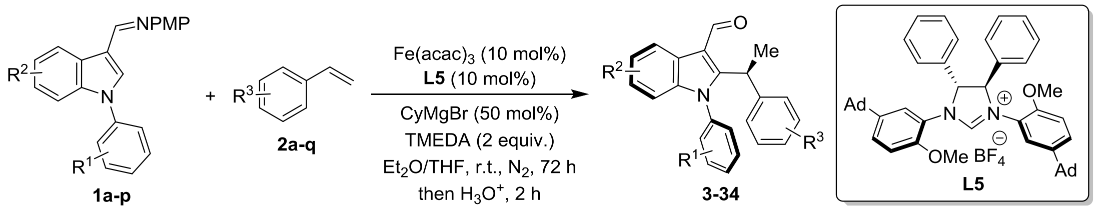

To a flame-dried and N_2-purged Schlenk tube were added indole substrate (0.1 mmol), $Fe(acac)_3$ (10 mol%, 0.01 mmol, 3.5 mg) and chiral NHC ligand **L5** (10 mol%, 0.01 mmol, 7.9 mg). The Schlenk tube was then sealed, purged and backfilled with N_2 three times. Ethyl ether (0.2 mL), TMEDA (0.2 mmol, 30 μL) and alkene substrate (0.15 mmol) were added *via* syringe. CyMgBr (1 M in THF, 0.05 mmol, 0.05 mL) was then added dropwise and the resulting mixture was stirred at room temperature for 72 hours. Then, the reaction mixture was diluted with tetrahydrofuran (2.0 mL) and quenched with HCl aqueous solution (1 M, 1.0 mL). The resulting mixture was stirred at room temperature for 2 hours. The phases were then separated, the aqueous layer was extracted with ethyl acetate (5.0 mL ×3). The combined organic layer was washed with brine, dried over Na_2SO_4, filtered and concentrated *in vacuo*. The diastereomeric ratio was determined by ^{1}H NMR analysis of the crude reaction mixture. The residue was purified by column chromatography on silica gel (*n*-hexane: ethyl acetate = 10:1) to afford the desired product.

6. Characterization data of products

(*R*$_a$, *S*)-1-(2-Isopropylphenyl)-2-(1-phenylethyl)-1*H*-indole-3-carbaldehyde (3)

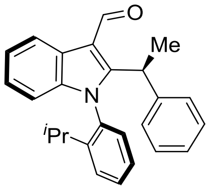

The **general procedure 4** was followed using indole substrate **1a** (0.1 mmol, 36.8 mg) and styrene (**2a**) (0.15 mmol, 17 μL) to afford **3** (33.1 mg, 90% yield, >95:5 d.r.) as a white solid. **M.p.**: 137-139 °C. **^{1}H NMR (400 MHz, CDCl$_3$)** δ 10.25 (s, 1H), 8.43 (d, *J* = 7.9 Hz, 1H), 7.66 – 7.49 (m, 2H), 7.39 – 7.14 (m, 8H), 7.06 (d, *J* = 7.8 Hz, 1H), 6.80 (d, *J* = 8.1 Hz, 1H), 4.20 (q, *J* = 7.4 Hz, 1H), 2.47 (hept, *J* = 6.9 Hz, 1H), 1.88 (d, *J* = 7.4 Hz, 3H), 1.21 (d, *J* = 6.9 Hz, 3H), 1.06 (d, *J* = 6.8 Hz, 3H). **^{13}C NMR (101 MHz, CDCl$_3$)** δ 186.4 (CH), 154.9 (C$_q$), 148.0 (C$_q$), 142.9 (C$_q$), 138.4 (C$_q$), 133.3 (C$_q$), 130.6 (CH), 129.5 (CH), 128.8 (CH), 127.5 (CH), 127.3 (CH), 127.2 (CH), 126.9 (CH), 126.0 (C$_q$), 123.8 (CH), 123.6 (CH), 122.2 (CH), 114.9 (C$_q$), 111.3 (CH), 37.1 (CH), 28.0 (CH), 24.8 (CH$_3$), 23.8 (CH$_3$), 22.7 (CH$_3$). **IR** (ATR) $\tilde{v}$ = 3056, 3027, 2964, 2926, 2869, 2826, 1650, 1494, 1458, 1398, 1234, 750, 697 cm^{-1}. **HRMS** (ESI) m/z (M+H)$^+$: calculated for $(C_{26}H_{26}NO)^+$: 368.2009, found: 368.2014; **$[\alpha]_D^{20}$** = +30.2 (c = 0.92, CHCl$_3$); The product was analyzed by HPLC to determine the enantiomeric excess: 97% e.e. (CHIRALPAK IC-3, *n*-hexane/*i*-PrOH = 95/5, flow rate: 1.0 mL/min, T = 20 °C, 250 nm), t$_R$ (major) = 22.18 min, t$_R$ (minor) = 24.78 min.

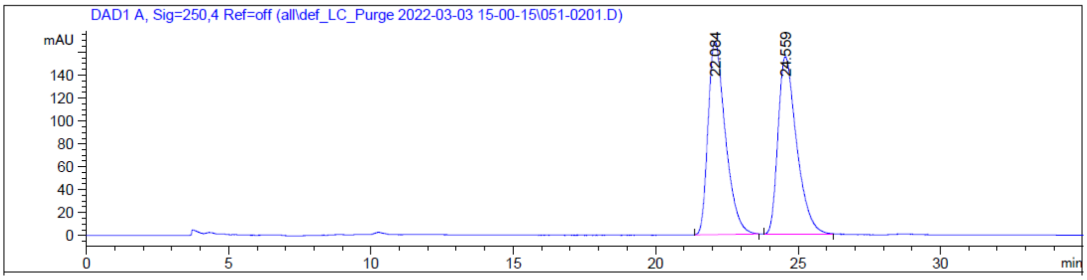

Peak #	RetTime [min]	Type	Width [min]	Area [mAU*s]	Height [mAU]	Area %
1	22.084	BB	0.5710	7134.90723	168.83073	50.3502
2	24.559	BB	0.5589	7035.65479	154.89104	49.6498

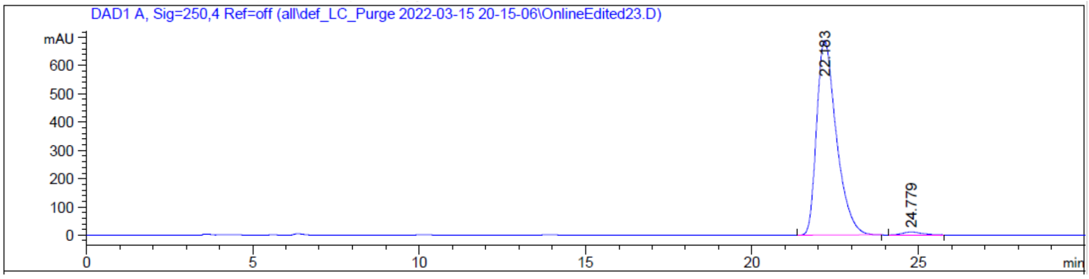

Peak #	RetTime [min]	Type	Width [min]	Area [mAU*s]	Height [mAU]	Area %
1	22.183	BB	0.5743	2.84091e4	688.65875	98.4927
2	24.779	BV R	0.4851	434.76352	10.54317	1.5073

(R_a, *S*)-2-(1-Phenylethyl)-1-(*o*-tolyl)-1*H*-indole-3-carbaldehyde (4)

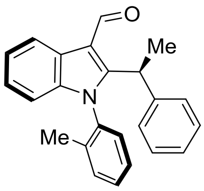

The **general procedure 4** was followed using indole substrate **1b** (0.1 mmol, 34.0 mg) and styrene (**2a**) (0.15 mmol, 17 µL) to afford **4** (30.9 mg, 91% yield, >95:5 d.r.) as a pale yellow oil. **^{1}H NMR (400 MHz, CDCl$_3$)** δ 10.25 (s, 1H), 8.43 (d, *J* = 7.9 Hz, 1H), 7.46 (td, *J* = 7.6, 1.4 Hz, 1H), 7.39 – 7.29 (m, 3H), 7.25 – 7.16 (m, 5H), 7.15 – 7.10 (m, 2H), 6.74 (d, *J* = 8.2 Hz, 1H), 4.47 (q, *J* = 7.4 Hz, 1H), 1.83 (d, *J* = 7.4 Hz, 3H), 1.79 (s, 3H). **^{13}C NMR (101 MHz, CDCl$_3$)** δ 186.1 (CH), 154.6 (C_q), 142.1 (C_q), 137.6 (C_q), 137.5 (C_q), 135.0 (C_q), 131.6 (CH), 130.0 (CH), 129.6 (CH), 128.6 (CH), 127.4 (CH), 127.3 (CH), 126.9 (CH), 125.9 (C_q), 123.9 (CH), 123.5 (CH), 122.0 (CH), 114.7 (C_q), 110.8 (CH), 36.6 (CH), 21.7 (CH_3), 17.3 (CH_3). **IR** (ATR) $\tilde{v}$ = 3056, 3027, 2960, 2935, 2825, 1650, 1460, 1394, 1234, 751, 697 cm^{-1}. **HRMS** (ESI) m/z $(M+H)^+$: calculated for $(C_{24}H_{22}NO)^+$: 340.1696, found: 340.1692. $[\alpha]_D^{20}$ = –25.9 (c = 0.75, CHCl$_3$). The product was analyzed by HPLC to determine the enantiomeric excess: 95% e.e. (CHIRALPAK IC-3, *n*-hexane/*i*-PrOH = 90/10, flow rate: 1.0 mL/min, T = 20 °C, 254 nm), t_R (minor) = 20.97 min, t_R (major) = 23.27 min.

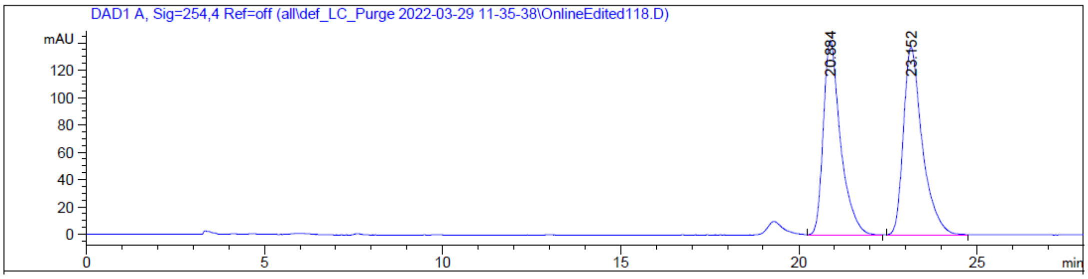

Peak #	RetTime [min]	Type	Width [min]	Area [mAU*s]	Height [mAU]	Area %
1	20.884	BB	0.5000	4754.23975	142.20517	48.5183
2	23.152	BB	0.5454	5044.61572	137.05435	51.4817

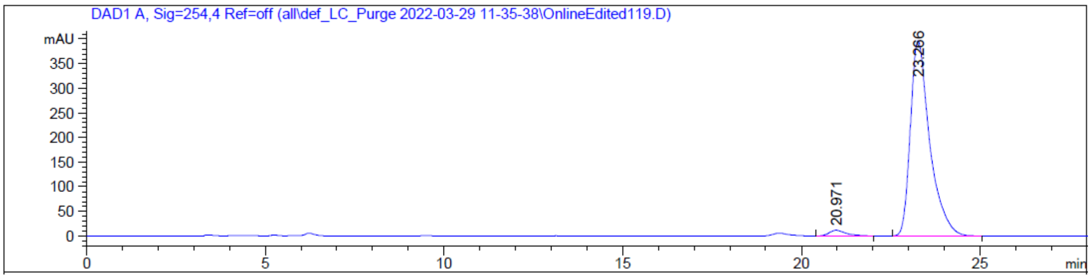

Peak #	RetTime [min]	Type	Width [min]	Area [mAU*s]	Height [mAU]	Area %
1	20.971	BB	0.4518	381.13614	11.64785	2.4920
2	23.266	BB	0.5545	1.49134e4	396.78305	97.5080

(*R*$_a$, *S*)-1-(2-Ethylphenyl)-2-(1-phenylethyl)-1*H*-indole-3-carbaldehyde (5)

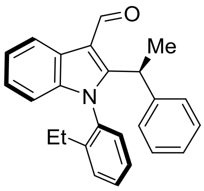

The **general procedure 4** was followed using indole substrate **1c** (0.1 mmol, 35.4 mg) and styrene (**2a**) (0.15 mmol, 17 µL) to afford **5** (32.2 mg, 91% yield, >95:5 d.r.) as a yellow oil. **^{1}H NMR (400 MHz, $CDCl_3$)** δ 10.27 (s, 1H), 8.43 (d, *J* = 7.9 Hz, 1H), 7.55 – 7.41 (m, 2H), 7.32 (t, *J* = 7.4 Hz, 2H), 7.27 – 7.15 (m, 4H), 7.12 (d, *J* = 8.0 Hz, 3H), 6.76 (d, *J* = 8.2 Hz, 1H), 4.39 (q, *J* = 7.4 Hz, 1H), 2.10 (q, *J* = 7.7 Hz, 2H), 1.83 (d, *J* = 7.4 Hz, 3H), 1.02 (t, *J* = 7.6 Hz, 3H). **^{13}C NMR (101 MHz, $CDCl_3$)** δ 186.2 (CH), 154.6 (C_q), 143.0 (C_q), 142.3 (C_q), 138.1 (C_q), 134.4 (C_q), 130.2 (CH), 129.6 (CH), 129.5 (CH), 128.7 (CH), 127.4 (CH), 127.2 (CH), 126.9 (CH), 125.9 (C_q), 123.8 (CH), 123.5 (CH), 122.0 (CH), 114.9 (C_q), 111.1 (CH), 36.9 (CH), 23.3 (CH_2), 22.0 (CH_3), 14.3 (CH_3). **IR** (ATR) $\tilde{\nu}$ =

3057, 3026, 2971, 2935, 2876, 2828, 1650, 1494, 1459, 1395, 1233, 749, 697 cm^{-1}. **HRMS** (ESI) m/z $(M+Na)^+$: calculated for $(C_{25}H_{23}NONa)^+$: 376.1672, found: 376.1686; $[\alpha]_D^{20}$ = –15.4 (c = 0.63, $CHCl_3$); The product was analyzed by HPLC to determine the enantiomeric excess: 97% e.e. (CHIRALPAK IC-3, *n*-hexane/*i*-PrOH = 95/5, flow rate: 0.7 mL/min, T = 20 °C, 250 nm), t_R (minor) = 44.21 min, t_R (major) = 46.09 min.

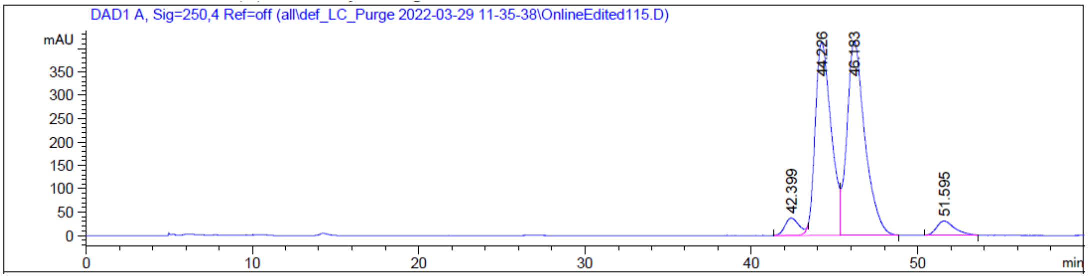

Peak #	RetTime [min]	Type	Width [min]	Area [mAU*s]	Height [mAU]	Area %
1	42.399	BV E	0.6782	2131.36279	37.04669	3.3771
2	44.226	VV R	0.8018	2.76115e4	412.58896	43.7499
3	46.183	VV R	0.8876	3.10869e4	415.61838	49.2567
4	51.595	VB R	0.8801	2282.31396	30.46076	3.6163

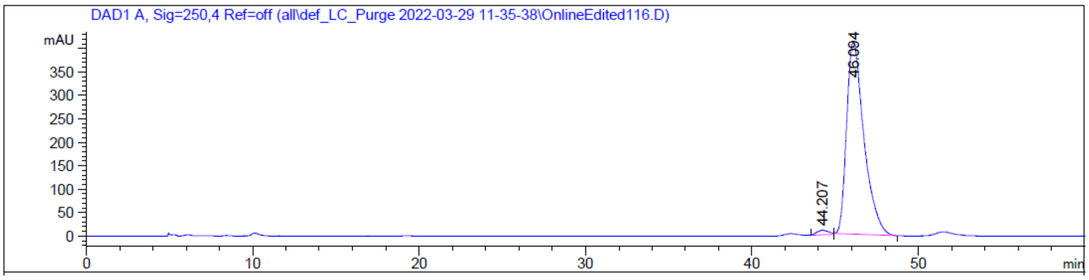

Peak #	RetTime [min]	Type	Width [min]	Area [mAU*s]	Height [mAU]	Area %
1	44.207	MM R	0.8259	470.90173	9.50314	1.5629
2	46.094	BV R	0.8530	2.96595e4	410.48755	98.4371

(*R_a*, *S*)-1-([1,1'-Biphenyl]-2-yl)-2-(1-phenylethyl)-1*H*-indole-3-carbaldehyde (6)

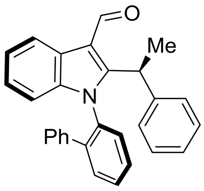

The **general procedure 4** was followed using indole substrate **1d** (0.1 mmol, 40.2 mg) and styrene (**2a**) (0.15 mmol, 17 μL) to afford **6** (29.2 mg, 73% yield, >95:5 d.r.) as a pale yellow solid.

M.p.: 176-178 °C. **^{1}H NMR (400 MHz, CDCl$_3$)** δ 10.10 (s, 1H), 8.44 (d, J = 7.7 Hz, 1H), 7.75 (dd, J = 7.8, 1.6 Hz, 1H), 7.68 (td, J = 7.5, 1.3 Hz, 1H), 7.56 (td, J = 7.6, 1.7 Hz, 1H), 7.38 (td, J = 7.8, 1.2 Hz, 2H), 7.36 – 7.27 (m, 2H), 7.28 – 7.19 (m, 4H), 7.20 – 7.10 (m, 2H), 7.09 – 7.02 (m, 4H), 4.04 (q, J = 7.3 Hz, 1H), 1.10 (d, J = 7.4 Hz, 3H). **^{13}C NMR (101 MHz, CDCl$_3$)** δ 186.3 (CH), 155.0 (C$_q$), 142.9 (C$_q$), 141.2 (C$_q$), 138.8 (C$_q$), 137.6 (C$_q$), 133.7 (C$_q$), 131.6 (CH), 130.5 (CH), 130.3 (CH), 129.0 (CH), 129.0 (CH), 128.6 (CH), 128.5 (CH), 128.2 (CH), 126.9 (CH), 126.7 (CH), 126.2 (C$_q$), 124.0 (CH), 123.6 (CH), 122.3 (CH), 114.7 (C$_q$), 111.3 (CH), 36.8 (CH), 20.9 (CH$_3$). **IR** (ATR) $\tilde{\nu}$ = 3057, 3027, 2936, 2834, 1650, 1478, 1455, 1397, 1230, 744, 698 cm^{-1}. **HRMS** (ESI) m/z (M+H)$^+$: calculated for $(C_{29}H_{24}NO)^+$: 402.1852, found: 402.1846; **$[\alpha]_D^{20}$** = +42.2 (c = 0.78, CHCl$_3$); The product was analyzed by HPLC to determine the enantiomeric excess: 98% e.e. (CHIRALPAK IC-3, *n*-hexane/*i*-PrOH = 95/5, flow rate: 1.0 mL/min, T = 20 °C, 250 nm), t_R (major) = 27.55 min, t_R (minor) = 32.97 min.

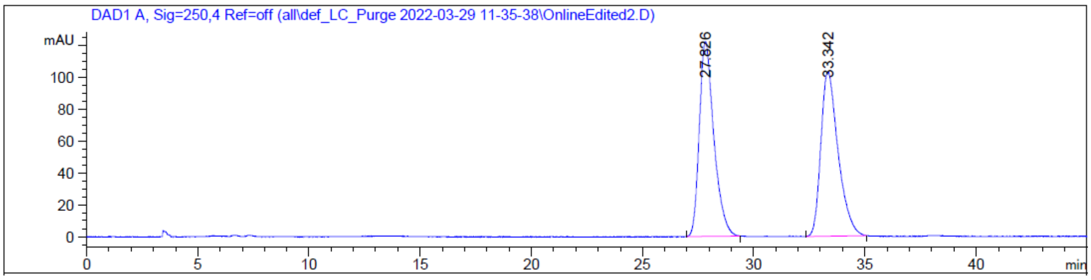

Peak #	RetTime [min]	Type	Width [min]	Area [mAU*s]	Height [mAU]	Area %
1	27.826	BB	0.5352	5554.23828	121.99103	49.9284
2	33.342	BV R	0.6364	5570.16895	103.08459	50.0716

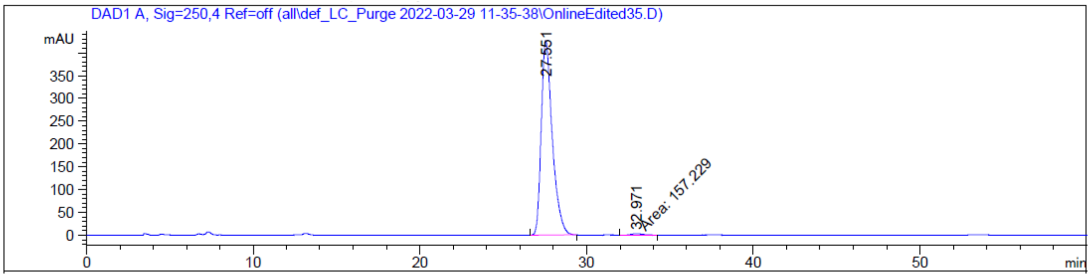

Peak #	RetTime [min]	Type	Width [min]	Area [mAU*s]	Height [mAU]	Area %
1	27.551	BV R	0.6243	1.97383e4	427.02179	99.2097
2	32.971	MM T	0.9082	157.22931	2.99555	0.7903

(R_a, S)-2-(1-Phenylethyl)-1-(2-(trifluoromethoxy)phenyl)-1*H*-indole-3-carbaldehyde (7)

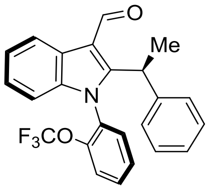

The **general procedure 4** was followed using indole substrate **1e** (0.1 mmol, 41.0 mg) and styrene (**2a**) (0.15 mmol, 17 μL) to afford **7** (27.8 mg, 68% yield, >95:5 d.r.) as a colorless oil. **^{1}H NMR (400 MHz, $CDCl_3$)** δ 10.25 (s, 1H), 8.42 (dt, J = 8.0, 0.9 Hz, 1H), 7.61 (ddd, J = 8.4, 7.4, 1.7 Hz, 1H), 7.52 (dt, J = 8.4, 1.6 Hz, 1H), 7.44 (td, J = 7.7, 1.4 Hz, 1H), 7.38 – 7.28 (m, 2H), 7.26 – 7.15 (m, 4H), 7.14 – 7.08 (m, 2H), 6.83 (d, J = 8.2 Hz, 1H), 4.28 (q, J = 7.4 Hz, 1H), 1.91 (d, J = 7.4 Hz, 3H). **^{13}C NMR (101 MHz, $CDCl_3$)** δ 186.4 (CH), 154.9 (C_q), 146.2 (q, J = 1.3 Hz, C_q), 142.5 (C_q), 138.0 (C_q), 131.5 (CH), 131.4 (CH), 128.8 (CH), 128.5 (C_q), 127.8 (CH), 127.1 (CH), 126.9 (CH), 126.1 (C_q), 124.1 (CH), 123.7 (CH), 122.2 (CH), 121.3 (q, J = 1.2 Hz, CH), 120.2 (q, J = 260.4 Hz, C_q), 115.4 (C_q), 110.5 (CH), 37.2 (CH), 21.8 (CH_3). **^{19}F NMR (377 MHz, $CDCl_3$)** δ -57.38. **IR** (ATR) $\tilde{\nu}$ = 3057, 3026, 2980, 2937, 2836, 1651, 1504, 1458, 1398, 1249, 1213, 1180, 748, 696 cm^{-1}. **HRMS** (ESI) m/z $(M+H)^+$: calculated for $(C_{24}H_{19}F_3NO_2)^+$: 410.1362, found: 410.1368; **$[\alpha]_D^{20}$** = –36.5 (c = 0.34, $CHCl_3$); The product was analyzed by HPLC to determine the enantiomeric excess: 91% e.e. (CHIRALPAK IC-3, *n*-hexane/*i*-PrOH = 95/5, flow rate: 1.0 mL/min, T = 20 °C, 250 nm), t_R (minor) = 22.08 min, t_R (major) = 23.89 min.

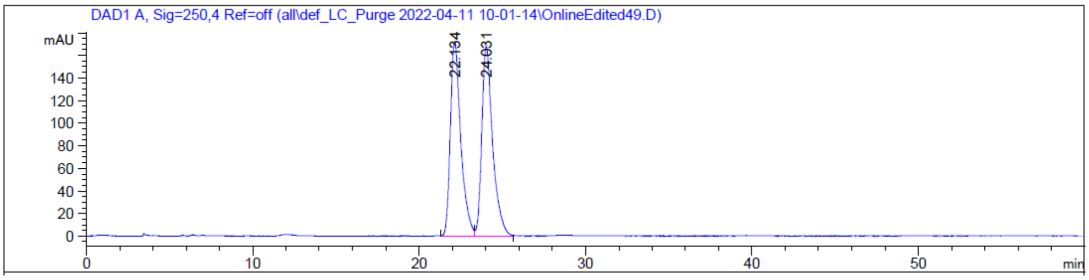

Peak #	RetTime [min]	Type	Width [min]	Area [mAU*s]	Height [mAU]	Area %
1	22.134	BV	0.5493	7479.38770	172.02431	49.7788
2	24.031	VV R	0.5323	7545.86914	167.58139	50.2212

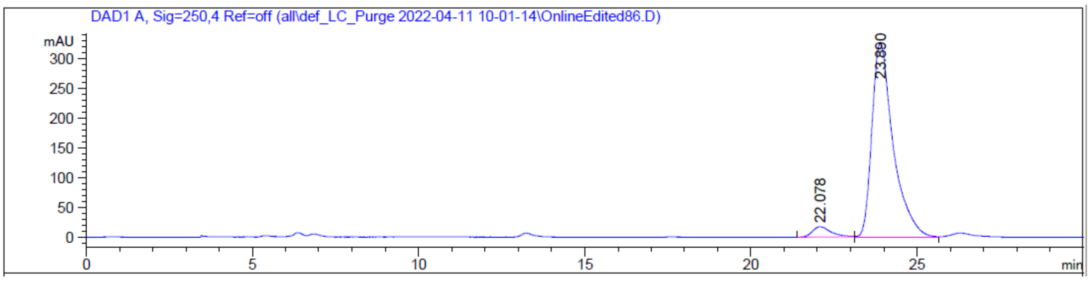

Peak #	RetTime [min]	Type	Width [min]	Area [mAU*s]	Height [mAU]	Area %
1	22.078	VB R	0.4442	654.26764	17.45888	4.3540
2	23.890	BV R	0.5954	1.43727e4	325.33093	95.6460

(*R*$_a$, *S*)-1-(2-Chlorophenyl)-2-(1-phenylethyl)-1*H*-indole-3-carbaldehyde (8)

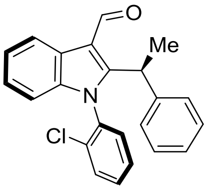

The **general procedure 4** was followed using indole substrate **1f** (0.1 mmol, 36.0 mg) and styrene (**2a**) (0.15 mmol, 17 μL) to afford **8** (20.2 mg, 56% yield, >95:5 d.r.) as a pale yellow oil. **^{1}H NMR (400 MHz, CDCl$_3$)** δ 10.23 (s, 1H), 8.42 (d, *J* = 7.7 Hz, 1H), 7.62 (dd, *J* = 8.0, 1.4 Hz, 1H), 7.52 (td, *J* = 7.8, 1.7 Hz, 1H), 7.42 (td, *J* = 7.7, 1.5 Hz, 1H), 7.36 – 7.29 (m, 2H), 7.27 – 7.17 (m, 4H), 7.14 (dd, *J* = 7.9, 1.3 Hz, 2H), 6.78 (d, *J* = 8.2 Hz, 1H), 4.30 (q, *J* = 7.3 Hz, 1H), 1.90 (d, *J* = 7.4 Hz, 3H). **^{13}C NMR (101 MHz, CDCl$_3$)** δ 186.3 (CH), 154.7 (C$_q$), 142.3 (C$_q$), 137.5 (C$_q$), 134.2 (C$_q$), 134.0 (C$_q$), 131.3 (CH), 131.3 (CH), 131.1 (CH), 128.7 (CH), 128.2 (CH), 127.2 (CH), 126.9 (CH), 126.0 (C$_q$), 124.1 (CH), 123.7 (CH), 122.2 (CH), 115.2 (C$_q$), 110.6 (CH), 37.2 (CH), 22.0 (CH$_3$). **IR** (ATR) $\tilde{\nu}$ = 3056, 3027, 2980, 2936, 1651, 1490, 1476, 1396, 1250, 1058, 746, 699 cm^{-1}. **HRMS** (ESI) m/z (M+H)$^+$: calculated for (C$_{23}$H$_{19}$ClNO)$^+$: 360.1150, found: 360.1147; **$[\alpha]_D^{20}$** = –20.9 (c = 0.33, CHCl$_3$); The product was analyzed by HPLC to determine the enantiomeric excess: 94% e.e. (CHIRALPAK IC-3, *n*-hexane/*i*-PrOH = 95/5, flow rate: 1.0 mL/min, T = 20 °C, 250 nm), t$_R$ (minor) = 35.11 min, t$_R$ (major) = 40.83 min.

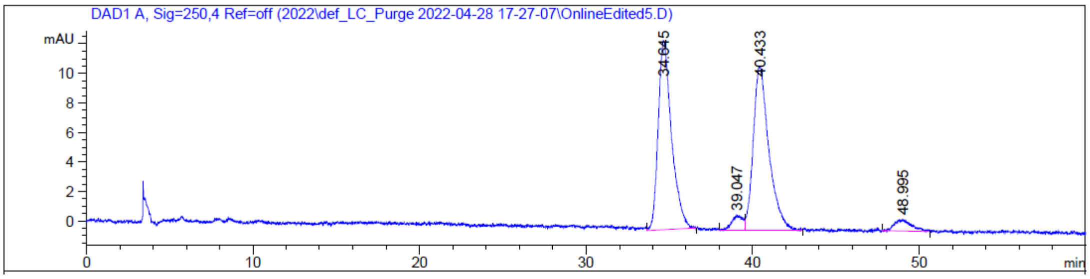

Peak #	RetTime [min]	Type	Width [min]	Area [mAU*s]	Height [mAU]	Area %
1	34.645	MM R	0.8430	714.06165	12.82436	46.2309
2	39.047	MF R	0.7670	48.98929	1.06456	3.1717
3	40.433	FM R	1.0849	728.62756	11.19397	47.1739
4	48.995	MM R	1.1008	52.87735	8.32915e-1	3.4235

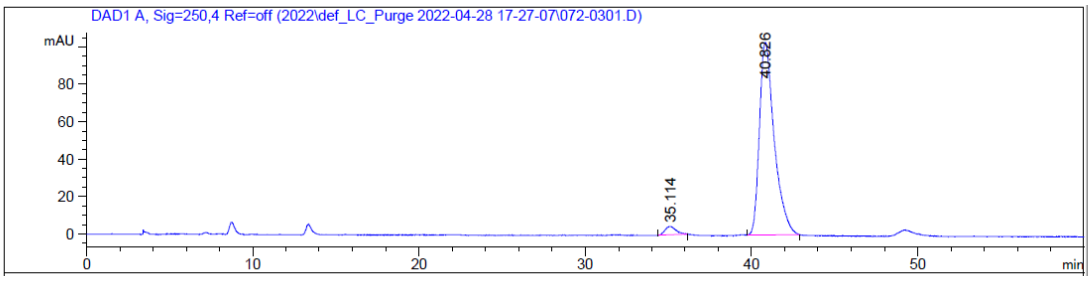

Peak #	RetTime [min]	Type	Width [min]	Area [mAU*s]	Height [mAU]	Area %
1	35.114	MM R	0.7250	195.16791	4.48679	2.9710
2	40.826	VV R	0.7288	6373.96191	103.17062	97.0290

(*R*$_a$, *S*)-1-(Naphthalen-1-yl)-2-(1-phenylethyl)-1*H*-indole-3-carbaldehyde (9)

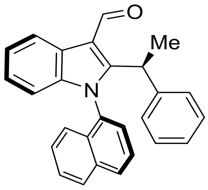

The **general procedure 4** was followed using indole substrate **1g** (0.1 mmol, 37.6 mg) and styrene (**2a**) (0.15 mmol, 17 µL) to afford **9** (32.4 mg, 86% yield, >95:5 d.r.) as a colorless oil. **^{1}H NMR (400 MHz, CDCl$_3$)** δ 10.26 (s, 1H), 8.48 (d, *J* = 8.0 Hz, 1H), 8.06 (d, *J* = 8.3 Hz, 1H), 7.99 (d, *J* = 8.5 Hz, 1H), 7.63 – 7.53 (m, 2H), 7.48 (dd, *J* = 7.3, 1.2 Hz, 1H), 7.42 – 7.28 (m, 2H), 7.22 – 7.06 (m, 6H), 7.04 (dd, *J* = 8.5, 1.0 Hz, 1H), 6.68 (d, *J* = 8.2 Hz, 1H), 4.34 (q, *J* = 7.3 Hz, 1H), 1.76 (d, *J* = 7.4 Hz, 3H). **^{13}C NMR (101 MHz, CDCl$_3$)** δ 186.3 (CH), 155.9 (C$_q$), 142.3 (C$_q$), 138.7 (C$_q$), 134.5 (C$_q$), 132.7 (C$_q$), 131.0 (C$_q$), 130.4 (CH), 128.6 (CH), 127.9 (CH), 127.4 (CH), 127.2 (CH), 127.2 (CH), 126.7 (CH),

125.9 (C_q), 125.5 (CH), 124.0 (CH), 123.6 (CH), 122.7 (CH), 122.1 (CH), 114.9 (C_q), 111.2 (CH), 36.8 (CH), 22.0 (CH_3). **IR** (ATR) $\tilde{v}$ = 3057, 3008, 2972, 2936, 2827, 1650, 1454, 1407, 1233, 1217, 1070, 748, 696 cm^{-1}. **HRMS** (ESI) m/z $(M+H)^+$: calculated for $(C_{27}H_{22}NO)^+$: 376.1696, found: 376.1694; $[\alpha]_D^{20}$ = –71.7 (c = 0.70, $CHCl_3$); The product was analyzed by HPLC to determine the enantiomeric excess: 93% e.e. (CHIRALPAK IC-3, *n*-hexane/*i*-PrOH = 95/5, flow rate: 1.0 mL/min, T = 20 °C, 250 nm), t_R (minor) = 35.32 min, t_R (major) = 43.42 min.

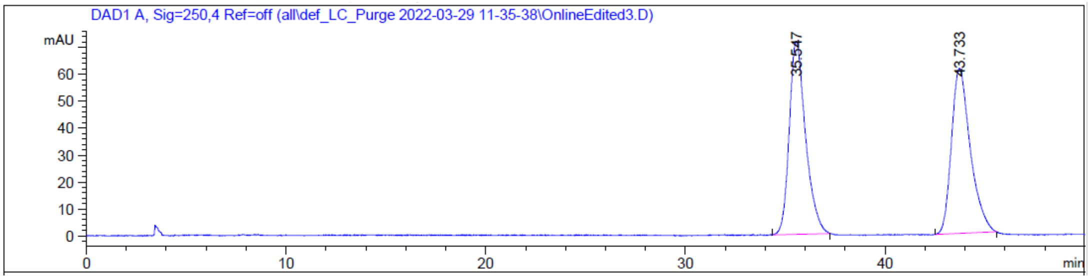

Peak #	RetTime [min]	Type	Width [min]	Area [mAU*s]	Height [mAU]	Area %
1	35.547	VV R	0.6670	4070.81079	71.85060	50.1846
2	43.733	VB R	0.7729	4040.86597	61.46972	49.8154

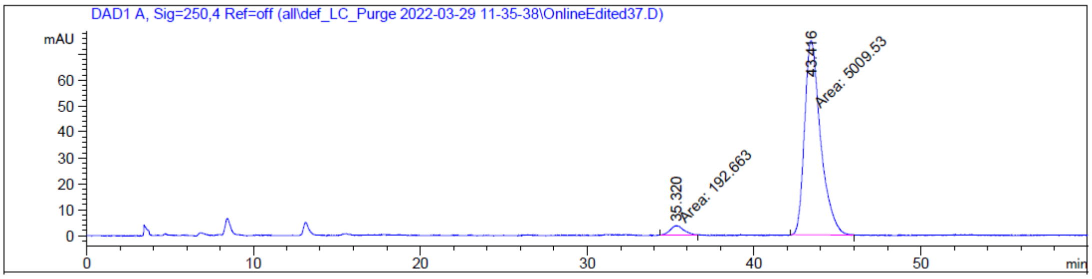

Peak #	RetTime [min]	Type	Width [min]	Area [mAU*s]	Height [mAU]	Area %
1	35.320	MM T	0.9004	192.66292	3.56614	3.7035
2	43.416	MM T	1.1151	5009.52539	74.87661	96.2965

(S_a, *S*)-4-Fluoro-1-(2-isopropylphenyl)-2-(1-phenylethyl)-1*H*-indole-3-carbaldehyde (10)

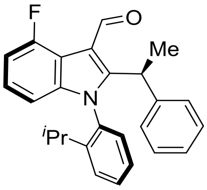

The **general procedure 4** was followed using indole substrate **1h** (0.1 mmol, 38.6 mg) and styrene (**2a**) (0.15 mmol, 17 µL) to afford **10** (13.9 mg, 36% yield, 90:10 d.r.) as a colorless oil. **^{1}H NMR (400 MHz, $CDCl_3$)** δ 10.42 (d, *J* = 1.6 Hz, 1H), 7.60 – 7.52 (m, 2H), 7.31 (ddd, *J* = 7.9,

5.5, 3.4 Hz, 1H), 7.24 – 7.14 (m, 5H), 7.13 – 7.04 (m, 2H), 6.99 (ddd, J = 10.8, 8.0, 0.9 Hz, 1H), 6.59 (d, J = 7.8 Hz, 1H), 4.25 (q, J = 7.3 Hz, 1H), 2.35 (hept, J = 6.8 Hz, 1H), 1.85 (d, J = 7.3 Hz, 3H), 1.14 (d, J = 6.9 Hz, 3H), 1.01 (d, J = 6.8 Hz, 3H). **^{13}C NMR (101 MHz, $CDCl_3$)** δ 185.6 (CH), 156.8 (d, J = 252.8 Hz, C_q), 152.2 (C_q), 147.8 (C_q), 142.0 (C_q), 140.5 (d, J = 11.7 Hz, C_q), 133.2 (C_q), 130.8 (CH), 129.4 (CH), 128.4 (CH), 128.3 (C_q), 127.5 (CH), 127.4 (CH), 127.3 (CH), 126.6 (CH), 123.7 (d, J = 7.9 Hz, CH), 115.0 (d, J = 3.0 Hz, C_q), 108.6 (d, J = 20.9 Hz, CH), 107.9 (d, J = 3.8 Hz, CH), 37.1 (CH), 28.0 (CH), 24.8 (CH_3), 23.5 (CH_3), 18.8 (CH_3). **^{19}F NMR (377 MHz, $CDCl_3$)** δ -114.60. **IR** (ATR) ṽ = 3056, 3027, 2965, 2926, 2870, 1672, 1489, 1453, 1286, 1200, 781, 759, 697 cm^{-1}. **HRMS** (ESI) m/z $(M+H)^+$: calculated for $(C_{26}H_{25}FNO)^+$: 386.1915, found: 386.1916; **$[\alpha]_D^{20}$** = +18.8 (c = 0.16, $CHCl_3$); The product was analyzed by HPLC to determine the enantiomeric excess: 90% e.e. (CHIRALPAK IE-3, *n*-hexane/*i*-PrOH = 95/5, flow rate: 1.0 mL/min, T = 20 °C, 250 nm), t_R (minor) = 13.57 min, t_R (major) = 15.16 min.

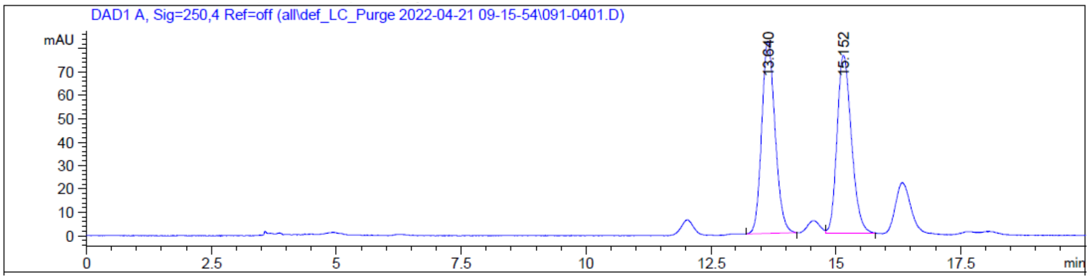

Peak #	RetTime [min]	Type	Width [min]	Area [mAU*s]	Height [mAU]	Area %
1	13.640	BB	0.2765	1533.24231	82.23599	50.2293
2	15.152	FM R	0.3035	1519.24414	75.99797	49.7707

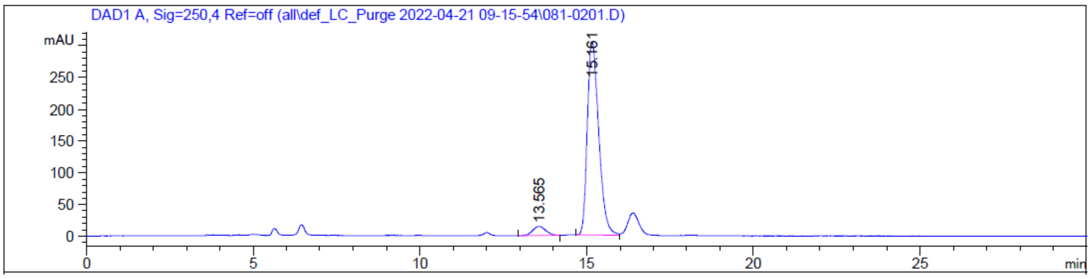

Peak #	RetTime [min]	Type	Width [min]	Area [mAU*s]	Height [mAU]	Area %
1	13.565	VV R	0.3218	392.54254	14.36671	5.1374
2	15.161	MF R	0.3870	7248.35596	304.94968	94.8626

(R_a, S)-5-Fluoro-1-(2-isopropylphenyl)-2-(1-phenylethyl)-1*H*-indole-3-carbaldehyde (11)

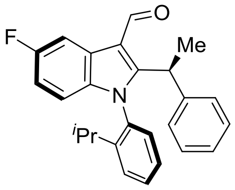

The **general procedure 4** was followed using indole substrate **1i** (0.1 mmol, 38.6 mg) and styrene (**2a**) (0.15 mmol, 17 μL) to afford **11** (24.3 mg, 63% yield, >95:5 d.r.) as a colorless oil. **^{1}H NMR (400 MHz, CDCl$_3$)** δ 10.20 (s, 1H), 8.11 (dd, J = 9.4, 2.5 Hz, 1H), 7.60 – 7.50 (m, 2H), 7.33 – 7.19 (m, 4H), 7.19 – 7.13 (m, 2H), 7.08 – 7.02 (m, 1H), 6.92 (td, J = 9.0, 2.6 Hz, 1H), 6.71 (dd, J = 8.9, 4.3 Hz, 1H), 4.16 (q, J = 7.4 Hz, 1H), 2.43 (hept, J = 6.9 Hz, 1H), 1.87 (d, J = 7.4 Hz, 3H), 1.21 (d, J = 6.9 Hz, 3H), 1.06 (d, J = 6.8 Hz, 3H). **^{13}C NMR (101 MHz, CDCl$_3$)** δ 186.1 (CH), 160.4 (d, J = 238.8 Hz, C$_q$), 155.9 (C$_q$), 147.9 (C$_q$), 142.6 (C$_q$), 134.8 (C$_q$), 133.1 (C$_q$), 130.8 (CH), 129.4 (CH), 128.9 (CH), 127.6 (CH), 127.3 (CH), 127.1 (CH), 127.0 (CH), 126.6 (d, J = 11.1 Hz, C$_q$), 114.9 (d, J = 4.4 Hz, C$_q$), 112.2 (d, J = 9.1 Hz, CH), 111.9 (d, J = 7.5 Hz, CH), 107.8 (d, J = 25.1 Hz, CH), 37.3 (CH), 28.0 (CH), 24.8 (CH$_3$), 23.8 (CH$_3$), 22.6 (CH$_3$). **^{19}F NMR (377 MHz, CDCl$_3$)** δ -120.00. **IR** (ATR) $\tilde{v}$ = 3018, 2968, 1649, 1475, 1460, 1215, 744, 668 cm^{-1}. **HRMS** (ESI) m/z (M+H)$^+$: calculated for $(C_{26}H_{25}FNO)^+$: 386.1915, found: 386.1920; **[α]$^{20}_{D}$** = +17.6 (c = 0.25, CHCl$_3$); The product was analyzed by HPLC to determine the enantiomeric excess: 94% e.e. (CHIRALPAK ID-3, *n*-hexane/*i*-PrOH = 95/5, flow rate: 1.0 mL/min, T = 20 °C, 250 nm), t$_R$ (minor) = 13.14 min, t$_R$ (major) = 14.49 min.

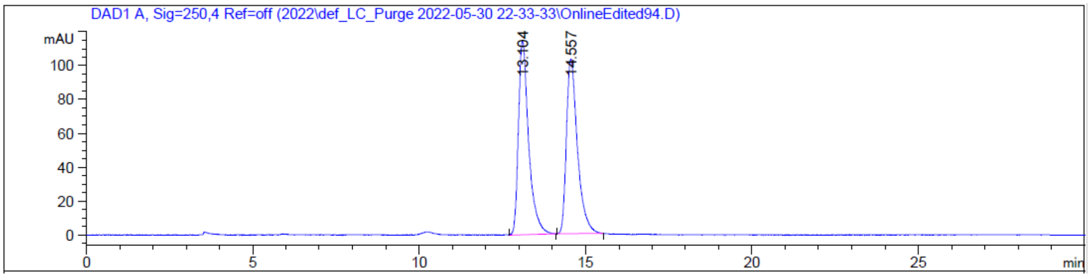

Peak #	RetTime [min]	Type	Width [min]	Area [mAU*s]	Height [mAU]	Area %
1	13.104	BV R	0.3060	2435.56421	114.55635	50.6242
2	14.557	BV R	0.3270	2375.50635	103.04195	49.3758

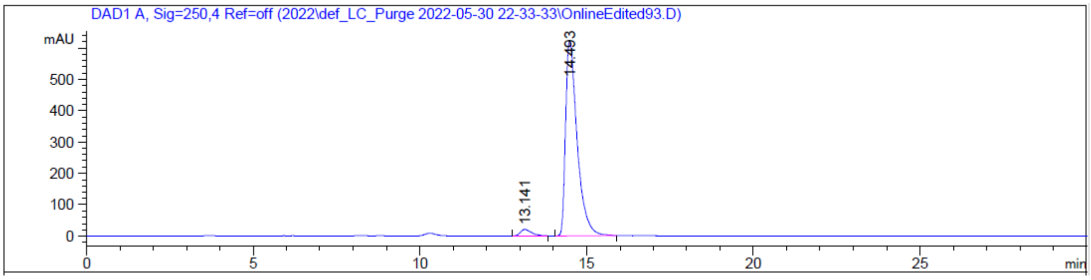

Peak #	RetTime [min]	Type	Width [min]	Area [mAU*s]	Height [mAU]	Area %
1	13.141	BV R	0.2786	476.70236	20.85794	3.0892
2	14.493	VV R	0.3552	1.49545e4	622.56830	96.9108

(*R*$_a$, *S*)-1-(2-Isopropylphenyl)-5-methyl-2-(1-phenylethyl)-1*H*-indole-3-carbaldehyde (12)

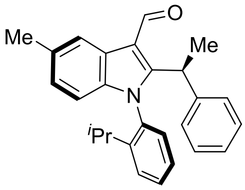

The **general procedure 4** was followed using indole substrate **1j** (0.1 mmol, 38.2 mg) and styrene (**2a**) (0.15 mmol, 17 μL) to afford **12** (31.3 mg, 82% yield, >95:5 d.r.) as a yellow oil. **^{1}H NMR (400 MHz, $CDCl_3$)** δ 10.21 (s, 1H), 8.26 (s, 1H), 7.63 – 7.49 (m, 2H), 7.33 – 7.23 (m, 3H), 7.23 – 7.14 (m, 3H), 7.08 – 6.99 (m, 2H), 6.69 (d, *J* = 8.3 Hz, 1H), 4.18 (q, *J* = 7.4 Hz, 1H), 2.53 – 2.44 (m, 4H), 1.87 (d, *J* = 7.4 Hz, 3H), 1.21 (d, *J* = 6.9 Hz, 3H), 1.07 (d, *J* = 6.8 Hz, 3H). **^{13}C NMR (101 MHz, $CDCl_3$)** δ 186.4 (CH), 155.0 (C_q), 147.9 (C_q), 142.9 (C_q), 136.8 (C_q), 133.4 (C_q), 133.3 (C_q), 130.5 (CH), 129.4 (CH), 128.7 (CH), 127.4 (CH), 127.2 (CH), 127.2 (CH), 126.8 (CH), 126.2 (C_q), 125.2 (CH), 121.9 (CH), 114.5 (C_q), 111.0 (CH), 37.0 (CH), 27.9 (CH), 24.8 (CH_3), 23.8 (CH_3), 22.6 (CH_3), 21.6 (CH_3). **IR** (ATR) $\tilde{v}$ = 3059, 3027, 2964, 2924, 2869, 1650, 1493, 1462, 1398, 1218, 800, 759, 712, 697 cm^{-1}. **HRMS** (ESI) m/z $(M+H)^+$: calculated for $(C_{27}H_{28}NO)^+$: 382.2165, found: 382.2166; **$[\alpha]_D^{20}$** = +36.8 (c = 0.78, $CHCl_3$); The product was analyzed by HPLC to determine the enantiomeric excess: 96% e.e. (CHIRALPAK IC-3, *n*-hexane/*i*-PrOH = 95/5, flow rate: 1.0 mL/min, T = 20 °C, 250 nm), t_R (major) = 18.74 min, t_R (minor) = 22.01 min.

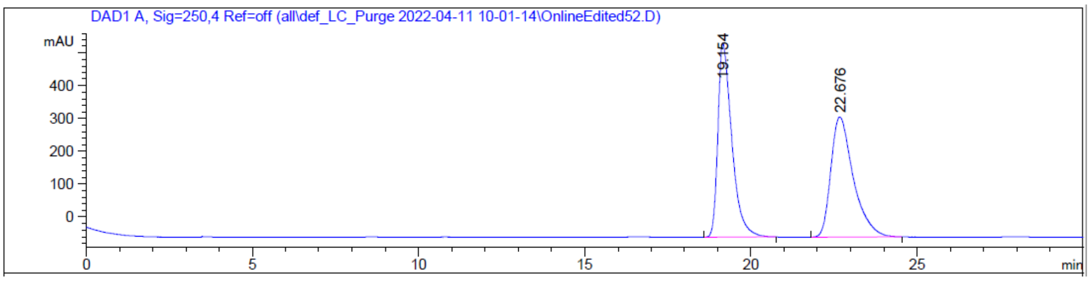

Peak #	RetTime [min]	Type	Width [min]	Area [mAU*s]	Height [mAU]	Area %
1	19.154	BB	0.4043	1.72201e4	592.15924	50.0180
2	22.676	BB	0.6178	1.72077e4	366.44458	49.9820

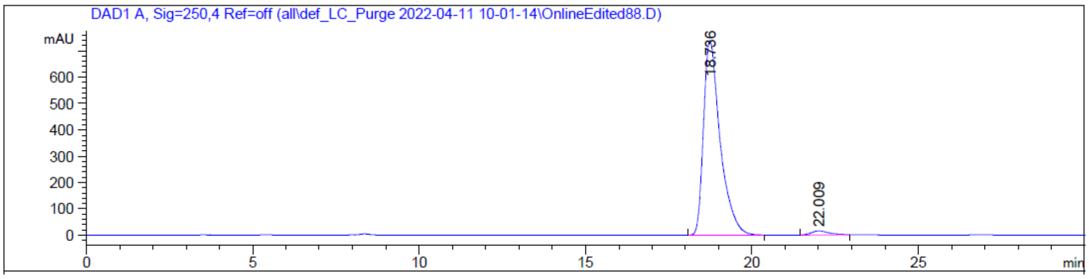

Peak #	RetTime [min]	Type	Width [min]	Area [mAU*s]	Height [mAU]	Area %
1	18.736	VV R	0.4998	2.56476e4	739.18561	97.8787
2	22.009	BV R	0.4382	555.85669	14.90170	2.1213

(*R*$_a$, *S*)-1-(2-Isopropylphenyl)-5-methoxy-2-(1-phenylethyl)-1*H*-indole-3-carbaldehyde (13)

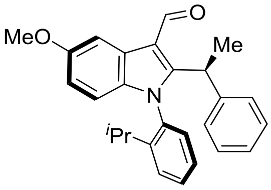

The **general procedure 4** was followed using indole substrate **1k** (0.1 mmol, 39.8 mg) and styrene (**2a**) (0.15 mmol, 17 μL) to afford **13** (27.8 mg, 70% yield, >95:5 d.r.) as a yellow oil. **^{1}H NMR (400 MHz, CDCl$_3$)** δ 10.26 (s, 1H), 8.01 (d, *J* = 2.5 Hz, 1H), 7.68 – 7.54 (m, 2H), 7.39 – 7.22 (m, 6H), 7.13 (d, *J* = 7.8 Hz, 1H), 6.89 (dd, *J* = 8.8, 2.5 Hz, 1H), 6.75 (d, *J* = 8.8 Hz, 1H), 4.23 (q, *J* = 7.4 Hz, 1H), 3.97 (s, 3H), 2.53 (hept, *J* = 6.8 Hz, 1H), 1.93 (d, *J* = 7.4 Hz, 3H), 1.27 (d, *J* = 6.9 Hz, 3H), 1.13 (d, *J* = 6.8 Hz, 3H). **^{13}C NMR (101 MHz, CDCl$_3$)** δ 186.4 (CH), 157.2 (C$_q$), 155.0 (C$_q$), 147.9 (C$_q$), 142.9 (C$_q$), 133.4 (C$_q$), 133.3 (C$_q$), 130.5 (CH), 129.4 (CH), 128.8 (CH), 127.5 (CH), 127.2 (CH), 127.1 (CH), 126.9 (CH), 126.6 (C$_q$), 114.7 (C$_q$), 114.1 (CH), 112.1 (CH),

103.4 (CH), 56.0 (CH_3), 37.1 (CH), 27.9 (CH), 24.8 (CH_3), 23.8 (CH_3), 22.6 (CH_3). **IR** (ATR) $\tilde{\nu}$ = 2964, 2935, 2869, 2831, 1650, 1478, 1414, 1260, 1078, 1032, 758 cm^{-1}. **HRMS** (ESI) m/z $(M+H)^+$: calculated for $(C_{27}H_{28}NO_2)^+$: 398.2115, found: 398.2114; $[\alpha]_D^{20}$ = +42.1 (c = 0.52, $CHCl_3$); The product was analyzed by HPLC to determine the enantiomeric excess: 98% e.e. (CHIRALPAK IC-3, *n*-hexane/*i*-PrOH = 95/5, flow rate: 1.0 mL/min, T = 20 °C, 250 nm), t_R (major) = 31.29 min, t_R (minor) = 33.98 min.

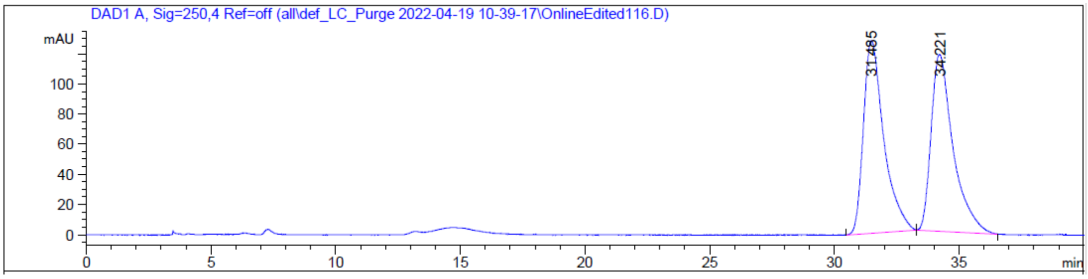

Peak #	RetTime [min]	Type	Width [min]	Area [mAU*s]	Height [mAU]	Area %
1	31.485	BB	0.6682	7256.68799	127.84484	50.2625
2	34.221	VV R	0.7203	7180.90430	117.29235	49.7375

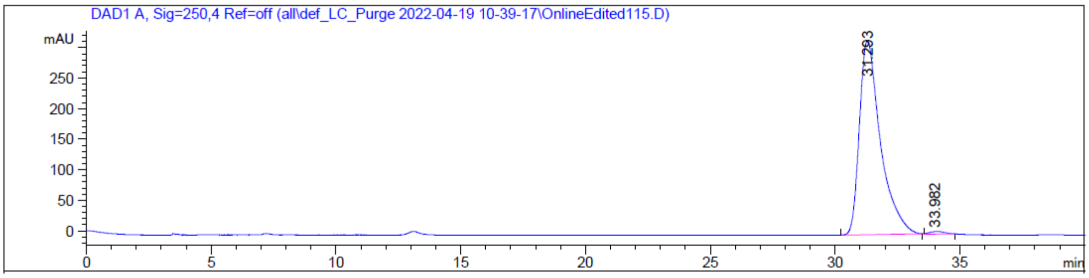

Peak #	RetTime [min]	Type	Width [min]	Area [mAU*s]	Height [mAU]	Area %
1	31.293	BV R	0.6923	1.85379e4	317.44739	98.9517
2	33.982	MM R	0.7535	196.39200	4.34372	1.0483

(*R*$_a$, *S*)-5-(Benzyloxy)-1-(2-isopropylphenyl)-2-(1-phenylethyl)-1*H*-indole-3-carbaldehyde (14)

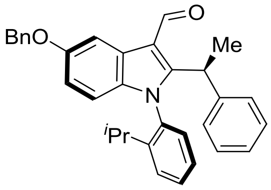

The **general procedure 4** was followed using indole substrate **1l** (0.1 mmol, 47.4 mg) and styrene (**2a**) (0.15 mmol, 17 μL) to afford **14** (25.4 mg, 54% yield, >95:5 d.r.) as a pale yellow oil. **^{1}H NMR**

(400 MHz, $CDCl_3$) δ 10.20 (s, 1H), 8.08 (s, 1H), 7.61 – 7.47 (m, 4H), 7.41 (t, *J* = 7.4 Hz, 2H), 7.34 (d, *J* = 7.4 Hz, 1H), 7.33 – 7.24 (m, 3H), 7.25 – 7.16 (m, 3H), 7.08 (d, *J* = 7.8 Hz, 1H), 6.93 (d, *J* = 8.8 Hz, 1H), 6.71 (d, *J* = 8.8 Hz, 1H), 5.18 (s, 2H), 4.18 (q, *J* = 7.4 Hz, 1H), 2.49 (hept, *J* = 7.0 Hz, 1H), 1.88 (d, *J* = 7.3 Hz, 3H), 1.22 (d, *J* = 6.8 Hz, 3H), 1.08 (d, *J* = 6.7 Hz, 3H). **^{13}C NMR (101 MHz, $CDCl_3$)** δ 186.4 (CH), 156.4 (C_q), 155.0 (C_q), 148.0 (C_q), 142.9 (C_q), 137.5 (C_q), 133.4 (C_q), 133.4 (C_q), 130.5 (CH), 129.4 (CH), 128.8 (CH), 128.7 (CH), 128.0 (CH), 127.8 (CH), 127.5 (CH), 127.2 (CH), 127.2 (CH), 126.9 (CH), 126.6 (C_q), 114.8 (C_q), 114.7 (CH), 112.2 (CH), 104.7 (CH), 70.8 (CH_2), 37.1 (CH), 27.9 (CH), 24.8 (CH_3), 23.8 (CH_3), 22.6 (CH_3). **IR** (ATR) $\tilde{\nu}$ = 3056, 3028, 2964, 2927, 2869, 1650, 1460, 1414, 1258, 1178, 759, 696 cm^{-1}. **HRMS** (ESI) m/z $(M+H)^+$: calculated for $(C_{33}H_{32}NO_2)^+$: 474.2428, found: 474.2435; **$[\alpha]_D^{20}$** = +43.0 (c = 0.37, $CHCl_3$); The product was analyzed by HPLC to determine the enantiomeric excess: 98% e.e. (CHIRALPAK ID-3, *n*-hexane/*i*-PrOH = 95/5, flow rate: 1.0 mL/min, T = 20 °C, 250 nm), t_R (minor) = 21.90 min, t_R (major) = 27.16 min.

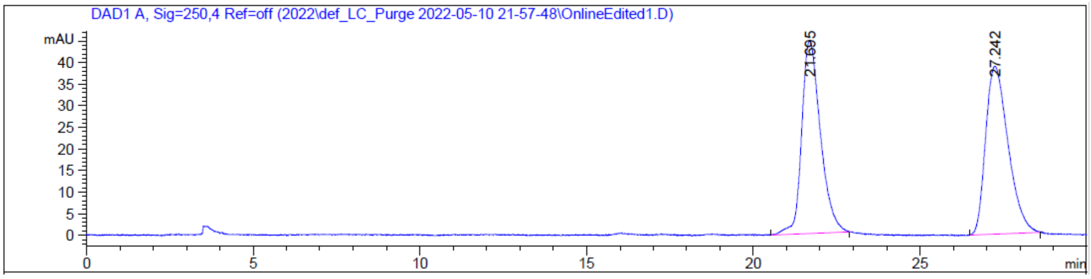

Peak #	RetTime [min]	Type	Width [min]	Area [mAU*s]	Height [mAU]	Area %
1	21.695	VV R	0.4603	1749.56641	44.73755	48.8891
2	27.242	BV R	0.5520	1829.07715	38.93844	51.1109

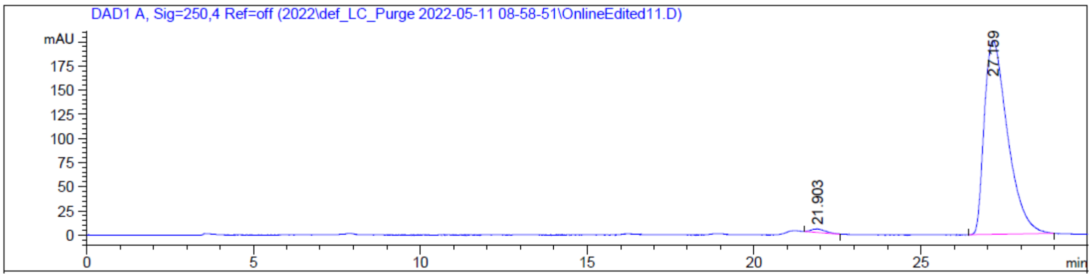

Peak #	RetTime [min]	Type	Width [min]	Area [mAU*s]	Height [mAU]	Area %
1	21.903	VB R	0.3421	107.97691	3.71520	1.0982
2	27.159	BV R	0.5734	9723.81641	200.64670	98.9018

(S_a, S)-6-Chloro-1-(2-isopropylphenyl)-2-(1-phenylethyl)-1*H*-indole-3-carbaldehyde (15)

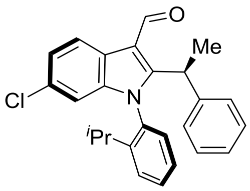

The **general procedure 4** was followed using indole substrate **1m** (0.1 mmol, 40.2 mg) and styrene (**2a**) (0.15 mmol, 17 µL) to afford **15** (20.1 mg, 50% yield, >95:5 d.r.) as a pale yellow oil. **^{1}H NMR (400 MHz, CDCl$_3$)** δ 10.22 (s, 1H), 8.34 (d, J = 8.5 Hz, 1H), 7.63 – 7.51 (m, 2H), 7.33 – 7.19 (m, 5H), 7.14 (d, J = 7.0 Hz, 2H), 7.03 (d, J = 7.8 Hz, 1H), 6.77 (d, J = 1.9 Hz, 1H), 4.17 (q, J = 7.4 Hz, 1H), 2.43 (hept, J = 6.8 Hz, 1H), 1.86 (d, J = 7.4 Hz, 3H), 1.22 (d, J = 6.9 Hz, 3H), 1.08 (d, J = 6.8 Hz, 3H). **^{13}C NMR (101 MHz, CDCl$_3$)** δ 186.2 (CH), 155.5 (C_q), 147.9 (C_q), 142.6 (C_q), 138.8 (C_q), 132.8 (C_q), 130.9 (CH), 129.7 (C_q), 129.4 (CH), 128.9 (CH), 127.7 (CH), 127.4 (CH), 127.1 (CH), 127.0 (CH), 124.5 (C_q), 124.1 (CH), 123.3 (CH), 114.9 (C_q), 111.2 (CH), 37.2 (CH), 28.0 (CH), 24.9 (CH$_3$), 23.8 (CH$_3$), 22.7 (CH$_3$). **IR** (ATR) $\tilde{\nu}$ = 3057, 3026, 2966, 2936, 2869, 1655, 1493, 1472, 1450, 1397, 761 cm^{-1}. **HRMS** (ESI) m/z (M+H)$^+$: calculated for $(C_{26}H_{25}ClNO)^+$: 402.1619, found: 402.1621; $[\alpha]_D^{20}$ = +4.9 (c = 0.33, CHCl$_3$); The product was analyzed by HPLC to determine the enantiomeric excess: 87% e.e. (CHIRALPAK IE-3, *n*-hexane/*i*-PrOH = 95/5, flow rate: 1.0 mL/min, T = 20 °C, 250 nm), t_R (minor) = 12.32 min, t_R (major) = 15.12 min.

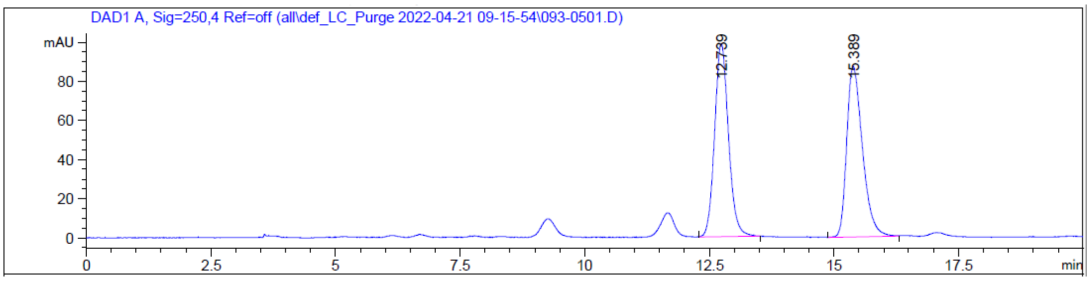

Peak #	RetTime [min]	Type	Width [min]	Area [mAU*s]	Height [mAU]	Area %
1	12.739	BV R	0.2850	1902.31519	98.62481	49.9555
2	15.389	VV R	0.3223	1905.70276	87.07484	50.0445

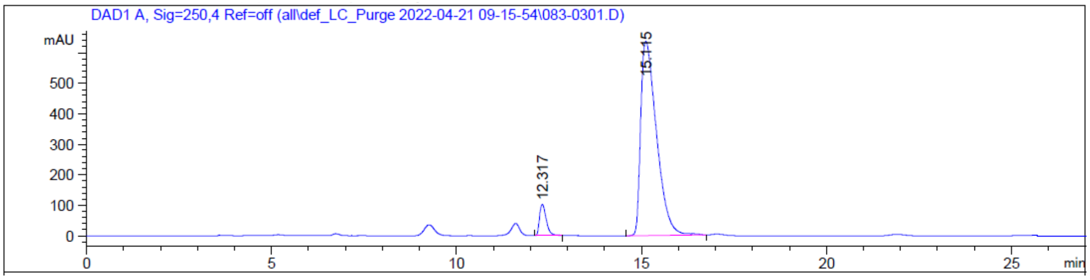

Peak #	RetTime [min]	Type	Width [min]	Area [mAU*s]	Height [mAU]	Area %
1	12.317	BB	0.1959	1275.23938	101.97403	6.3093
2	15.115	BV R	0.4332	1.89369e4	638.14557	93.6907

(*Ra*, *S*)-1-(2-Isopropylphenyl)-6-methyl-2-(1-phenylethyl)-1*H*-indole-3-carbaldehyde (16)**

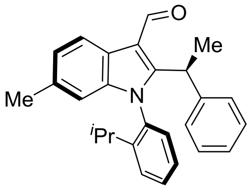

The **general procedure 4** was followed using indole substrate **1n** (0.1 mmol, 38.2 mg) and styrene (**2a**) (0.15 mmol, 17 µL) to afford **16** (19.8 mg, 52% yield, >95:5 d.r.) as a white solid. **M.p.**: 169-171 °C. **^{1}H NMR (400 MHz, $CDCl_3$)** δ 10.21 (s, 1H), 8.29 (d, *J* = 8.1 Hz, 1H), 7.62 – 7.50 (m, 2H), 7.32 – 7.10 (m, 7H), 7.03 (d, *J* = 8.3 Hz, 1H), 6.56 (s, 1H), 4.17 (q, *J* = 7.4 Hz, 1H), 2.47 (hept, *J* = 6.8 Hz, 1H), 2.36 (s, 3H), 1.86 (d, *J* = 7.4 Hz, 3H), 1.20 (d, *J* = 6.9 Hz, 3H), 1.06 (d, *J* = 6.8 Hz, 3H). **^{13}C NMR (101 MHz, $CDCl_3$)** δ 186.4 (CH), 154.5 (C_q), 148.0 (C_q), 143.0 (C_q), 138.8 (C_q), 133.9 (C_q), 133.5 (C_q), 130.5 (CH), 129.5 (CH), 128.7 (CH), 127.5 (CH), 127.2 (CH), 127.2 (CH), 126.8 (CH), 125.2 (CH), 123.7 (C_q), 121.8 (CH), 114.9 (C_q), 111.2 (CH), 37.1 (CH), 27.9 (CH), 24.8 (CH_3), 23.8 (CH_3), 22.7 (CH_3), 21.8 (CH_3). **IR** (ATR) $\tilde{\nu}$ = 3057, 3028, 2964, 2924, 2869, 1651, 1520, 1491, 1451, 1398, 760, 701 cm^{-1}. **HRMS** (ESI) m/z $(M+Na)^+$: calculated for $(C_{27}H_{27}NONa)^+$: 404.1985, found: 404.1982; $[\alpha]_D^{20}$ = +13.5 (c = 0.17, $CHCl_3$); The product was analyzed by HPLC to determine the enantiomeric excess: 96% e.e. (CHIRALPAK IC-3, *n*-hexane/*i*-PrOH = 95/5, flow rate: 1.0 mL/min, T = 20 °C, 250 nm), t_R (major) = 24.32 min, t_R (minor) = 27.22 min.

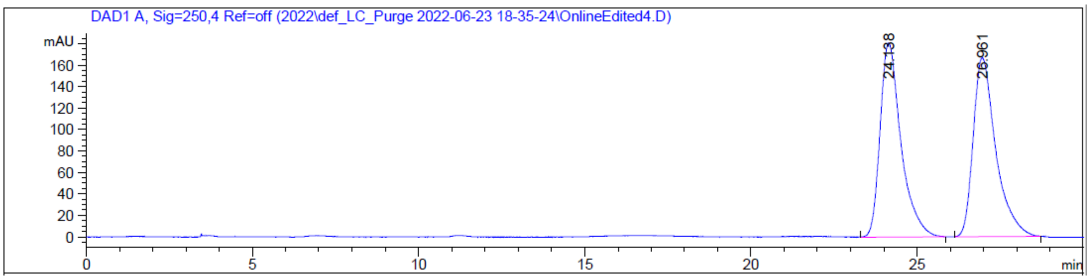

Peak #	RetTime [min]	Type	Width [min]	Area [mAU*s]	Height [mAU]	Area %
1	24.138	VV R	0.5547	7875.31250	180.59898	49.9603
2	26.961	VV R	0.5589	7887.83643	166.74387	50.0397

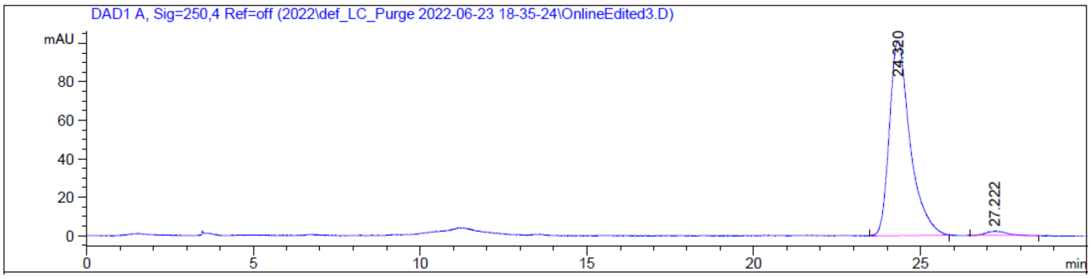

Peak #	RetTime [min]	Type	Width [min]	Area [mAU*s]	Height [mAU]	Area %
1	24.320	VV R	0.5212	4358.52588	101.46518	97.9374
2	27.222	MM R	0.6695	91.79020	2.28513	2.0626

(*S*a, *S*)-7-Fluoro-1-(2-isopropylphenyl)-2-(1-phenylethyl)-1H-indole-3-carbaldehyde (17)

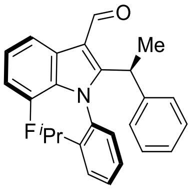

The **general procedure 4** was followed using indole substrate **1o** (0.1 mmol, 38.6 mg) and styrene (**2a**) (0.15 mmol, 17 µL) to afford **17** (22.0 mg, 57% yield, >95:5 d.r.) as a pale yellow oil. **^{1}H NMR (400 MHz, CDCl$_3$)** δ 10.21 (d, *J* = 1.0 Hz, 1H), 8.22 (dd, *J* = 8.0, 0.9 Hz, 1H), 7.57 – 7.48 (m, 2H), 7.33 – 7.08 (m, 8H), 6.89 (ddd, *J* = 12.1, 8.0, 1.0 Hz, 1H), 4.14 (q, *J* = 7.4 Hz, 1H), 2.53 (hept, *J* = 6.9 Hz, 1H), 1.86 (d, *J* = 7.4 Hz, 3H), 1.23 (d, *J* = 6.9 Hz, 3H), 1.10 (d, *J* = 6.8 Hz, 3H). **^{13}C NMR (101 MHz, CDCl$_3$)** δ 186.4 (CH), 155.9 (C$_q$), 149.4 (d, *J* = 248.1 Hz, C$_q$), 147.4 (C$_q$), 142.6 (C$_q$), 134.9 (C$_q$), 130.5 (CH), 129.4 (d, *J* = 3.3 Hz, C$_q$), 128.9 (d, *J* = 1.5 Hz, CH), 128.8 (CH), 127.1 (CH), 127.0 (CH), 127.0 (CH), 126.7 (CH), 125.7 (d, *J* = 8.1 Hz, C$_q$), 123.9 (d, *J* = 6.2 Hz, CH), 118.0 (d,

J = 4.0 Hz, CH), 115.2 (C_q), 109.9 (d, J = 17.2 Hz, CH), 36.5 (CH), 28.1 (CH), 24.2 (CH_3), 23.8 (CH_3), 22.7 (CH_3). **^{19}F NMR (377 MHz, $CDCl_3$)** δ -133.04. **IR** (ATR) ṽ = 3057, 3027, 2966, 2924, 2871, 1656, 1627, 1492, 1453, 1397, 1235, 783, 760, 734 cm^{-1}. **HRMS** (ESI) m/z $(M+H)^+$: calculated for $(C_{26}H_{25}FNO)^+$: 386.1915, found: 386.1911; **$[\alpha]^{20}_{D}$** = +12.5 (c = 0.12, $CHCl_3$); The product was analyzed by HPLC to determine the enantiomeric excess: 86% e.e. (CHIRALPAK OD-3, *n*-hexane/*i*-PrOH = 90/10, flow rate: 1.0 mL/min, T = 20 °C, 250 nm), t_R (major) = 5.76 min, t_R (minor) = 7.09 min.

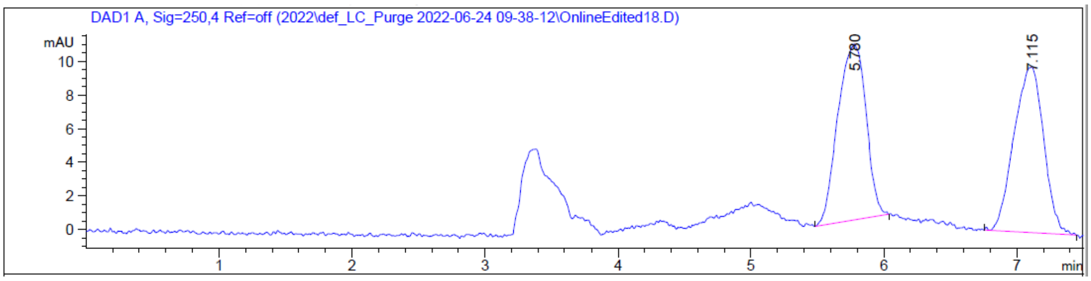

Peak #	RetTime [min]	Type	Width [min]	Area [mAU*s]	Height [mAU]	Area %
1	5.780	BB	0.1781	154.54388	10.47402	50.1552
2	7.115	VV R	0.2021	153.58714	9.89531	49.8448

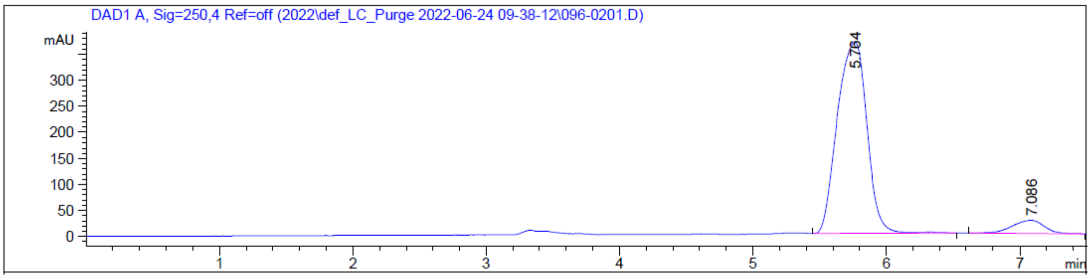

Peak #	RetTime [min]	Type	Width [min]	Area [mAU*s]	Height [mAU]	Area %
1	5.764	BV R	0.2517	5666.40674	368.49020	93.0627
2	7.086	BBA	0.2303	422.39938	25.36348	6.9373

(*S*$_a$, *S*)-1-(2-Isopropylphenyl)-2-(1-phenylethyl)-1*H*-pyrrolo[2,3-b]pyridine-3-carbaldehyde (18)

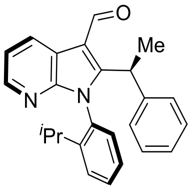

The **general procedure 4** was followed using indole substrate **1p** (0.1 mmol, 36.9 mg) and styrene (**2a**) (0.15 mmol, 17 µL) to afford **18** (30.9 mg, 84% yield, >95:5 d.r.) as a pale yellow solid. **M.p.**: 164-166 °C. **^{1}H NMR (400 MHz, CDCl$_3$)** δ 10.16 (s, 1H), 8.67 (dd, J = 7.8, 1.7 Hz, 1H), 8.32 (dd, J = 4.8, 1.6 Hz, 1H), 7.61 – 7.51 (m, 2H), 7.34 – 7.17 (m, 7H), 7.12 – 7.08 (m, 1H), 4.28 (q, J = 7.4 Hz, 1H), 2.39 (hept, J = 6.9 Hz, 1H), 1.86 (d, J = 7.4 Hz, 3H), 1.21 (d, J = 6.9 Hz, 3H), 1.09 (d, J = 6.8 Hz, 3H). **^{13}C NMR (101 MHz, CDCl$_3$)** δ 186.3 (CH), 155.6 (C$_q$), 149.4 (C$_q$), 147.9 (C$_q$), 145.3 (CH), 142.5 (C$_q$), 132.5 (C$_q$), 130.7 (CH), 130.7 (CH), 129.4 (CH), 128.9 (CH), 127.4 (CH), 127.2 (CH), 127.2 (CH), 127.1 (CH), 119.6 (CH), 118.7 (C$_q$), 113.3 (C$_q$), 37.1 (CH), 28.4 (CH), 24.5 (CH$_3$), 23.5 (CH$_3$), 22.6 (CH$_3$). **IR** (ATR) $\tilde{\nu}$ = 3057, 3028, 2963, 2926, 2869, 1652, 1493, 1424, 1408, 1269, 777, 758, 697 cm^{-1}. **HRMS** (ESI) m/z (M+H)$^{+}$: calculated for $(C_{25}H_{25}N_2O)^{+}$: 369.1961, found: 369.1962; **$[\alpha]_D^{20}$** = +9.2 (c = 0.36, CHCl$_3$); The product was analyzed by HPLC to determine the enantiomeric excess: 91% e.e. (CHIRALPAK IC-3, *n*-hexane/*i*-PrOH = 95/5, flow rate: 1.0 mL/min, T = 20 °C, 250 nm), t_R (major) = 18.31 min, t_R (minor) = 20.06 min.

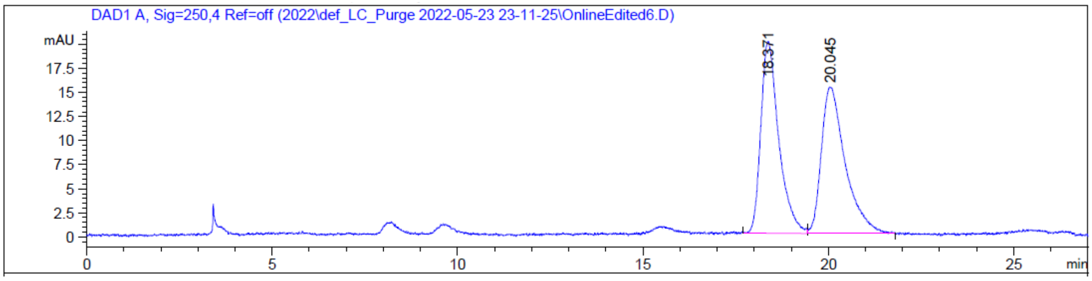

Peak #	RetTime [min]	Type	Width [min]	Area [mAU*s]	Height [mAU]	Area %
1	18.371	MF R	0.5606	669.63342	19.90737	49.6428
2	20.045	FM R	0.7086	679.26874	15.16825	50.3572

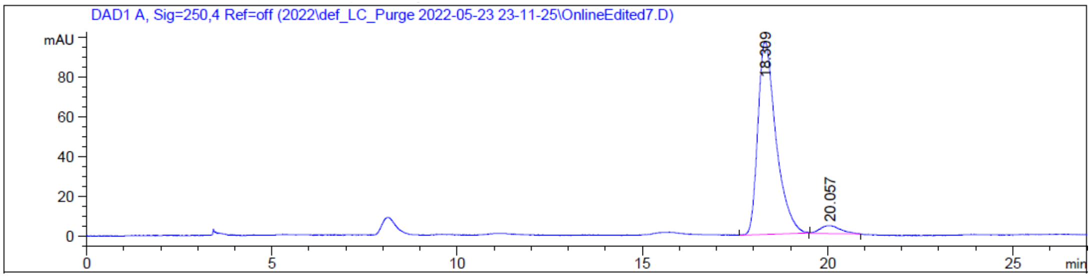

Peak #	RetTime [min]	Type	Width [min]	Area [mAU*s]	Height [mAU]	Area %
1	18.309	VB R	0.4218	3306.48901	97.21434	95.5147
2	20.057	MM R	0.6076	155.27171	4.07997	4.4853

(R_a, *S*)-2-(1-(4-Fluorophenyl)ethyl)-1-(2-isopropylphenyl)-1*H*-indole-3-carbaldehyde (19)

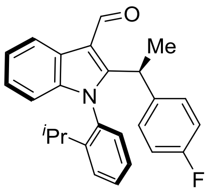

The **general procedure 4** was followed using indole substrate **1a** (0.1 mmol, 36.8 mg) and 1-fluoro-4-vinylbenzene (**2b**) (0.15 mmol, 18 μL) to afford **19** (35.9 mg, 93% yield, >95:5 d.r.) as a pale yellow oil. **^{1}H NMR (400 MHz, CDCl$_3$)** δ 10.27 (s, 1H), 8.43 (d, *J* = 7.9 Hz, 1H), 7.64 – 7.52 (m, 2H), 7.35 – 7.27 (m, 2H), 7.20 (ddd, *J* = 8.4, 7.1, 1.2 Hz, 1H), 7.16 – 7.10 (m, 2H), 7.05 – 7.00 (m, 1H), 6.98 – 6.91 (m, 2H), 6.80 (d, *J* = 8.2 Hz, 1H), 4.18 (q, *J* = 7.4 Hz, 1H), 2.45 (hept, *J* = 6.9 Hz, 1H), 1.87 (d, *J* = 7.3 Hz, 3H), 1.21 (d, *J* = 6.9 Hz, 3H), 1.06 (d, *J* = 6.8 Hz, 3H). **^{13}C NMR (101 MHz, CDCl$_3$)** δ 186.0 (CH), 161.7 (d, *J* = 245.8 Hz, C$_q$), 154.3 (C$_q$), 148.0 (C$_q$), 138.6 (d, *J* = 3.3 Hz, C$_q$), 138.3 (C$_q$), 133.2 (C$_q$), 130.7 (CH), 129.3 (CH), 128.8 (d, *J* = 7.9 Hz, CH), 127.4 (d, *J* = 23.1 Hz, CH), 126.0 (C$_q$), 123.9 (CH), 123.6 (CH), 122.0 (CH), 115.7 (CH), 115.4 (CH), 114.8 (C$_q$), 111.3 (CH), 36.6 (CH), 28.0 (CH), 24.8 (CH$_3$), 23.7 (CH$_3$), 22.7 (CH$_3$). **^{19}F NMR (377 MHz, CDCl$_3$)** δ -115.99. **IR** (ATR) $\tilde{\nu}$ = 2966, 2935, 2869, 1650, 1509, 1458, 1400, 1234, 1079, 835, 758 cm^{-1}. **HRMS** (ESI) m/z (M+H)$^+$: calculated for $(C_{26}H_{25}FNO)^+$: 386.1915, found: 386.1911; **[α]$_D^{20}$** = +35.3 (c = 1.09, CHCl$_3$); The product was analyzed by HPLC to determine the enantiomeric excess: 97% e.e. (CHIRALPAK IC-3, *n*-hexane/*i*-PrOH = 95/5, flow rate: 1.0 mL/min, T = 20 °C, 250 nm), t_R (minor) = 23.47 min, t_R (major) = 25.13 min.

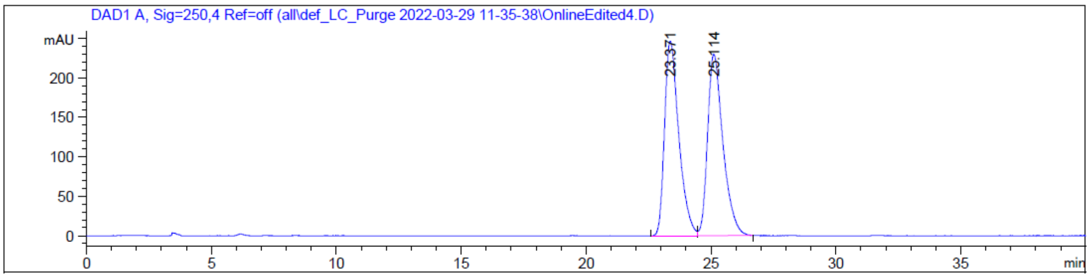

Peak #	RetTime [min]	Type	Width [min]	Area [mAU*s]	Height [mAU]	Area %
1	23.371	VV R	0.5518	9807.91504	246.70218	49.9443
2	25.114	VV R	0.5826	9829.77930	230.01192	50.0557

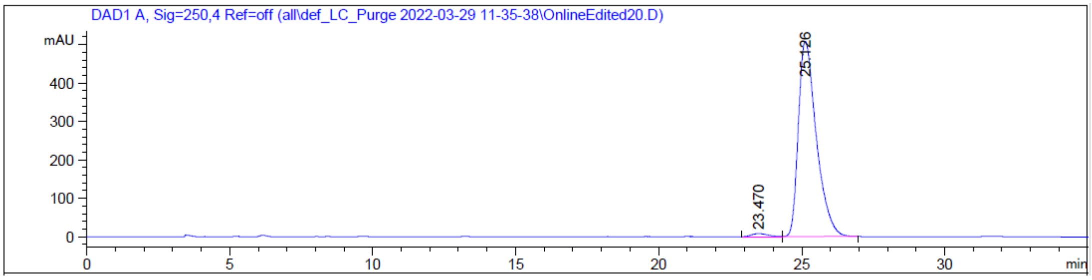

Peak #	RetTime [min]	Type	Width [min]	Area [mAU*s]	Height [mAU]	Area %
1	23.470	VV R	0.4412	310.42465	8.30221	1.3817
2	25.126	VV R	0.6129	2.21570e4	507.91214	98.6183

(R_a, *S*)-1-(2-Isopropylphenyl)-2-(1-(4-(trifluoromethyl)phenyl)ethyl)-1*H*-indole-3-carbaldehyde (20)

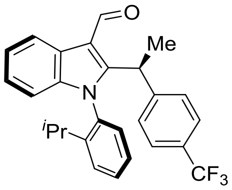

The **general procedure 4** was followed using indole substrate **1a** (0.1 mmol, 36.8 mg) and 1-(trifluoromethyl)-4-vinylbenzene (**2c**) (0.15 mmol, 22 μL) to afford **20** (11.0 mg, 25% yield, >95:5 d.r.) as a colorless oil. **^{1}H NMR (400 MHz, CDCl$_3$)** δ 10.24 (s, 1H), 8.41 (d, *J* = 7.9 Hz, 1H), 7.63 – 7.53 (m, 2H), 7.52 (d, *J* = 8.2 Hz, 2H), 7.39 – 7.25 (m, 4H), 7.21 (t, *J* = 7.7 Hz, 1H), 7.02 (d, *J* = 7.8 Hz, 1H), 6.81 (d, *J* = 8.2 Hz, 1H), 4.24 (q, *J* = 7.4 Hz, 1H), 2.44 (hept, *J* = 6.8 Hz, 1H), 1.90 (d, *J* = 7.4 Hz, 3H), 1.20 (d, *J* = 6.9 Hz, 3H), 1.06 (d, *J* = 6.8 Hz, 3H). **^{13}C NMR (101 MHz, CDCl$_3$)** δ 185.7 (CH), 153.2 (C$_q$), 148.0 (C$_q$), 146.9 (C$_q$), 138.4 (C$_q$), 133.2 (C$_q$), 130.8 (CH), 129.3 (CH), 129.3 (q, *J* = 32.63 Hz, C$_q$), 127.7 (CH), 127.6 (CH), 127.4 (CH), 126.1 (C$_q$), 125.7 (q, *J* = 3.9 Hz, CH), 125.7 (q, *J* = 35.6 Hz, C$_q$), 124.1 (CH), 123.7 (CH), 121.9 (CH), 114.9 (C$_q$), 111.4 (CH), 37.1 (CH), 28.0 (CH), 24.8 (CH$_3$), 23.8 (CH$_3$), 22.2 (CH$_3$). **^{19}F NMR (377 MHz, CDCl$_3$)** δ -62.53. **IR** (ATR) $\tilde{\nu}$ = 2965, 2924, 2870, 1734, 1651, 1618, 1458, 1404, 1326, 1166, 1121, 1071, 1018, 747 cm^{-1}. **HRMS** (ESI) m/z (M+H)$^+$: calculated for $(C_{27}H_{25}F_3NO)^+$: 436.1883, found: 436.1883; **$[\alpha]_D^{20}$** = +10.0 (c = 0.12, CHCl$_3$); The product was analyzed by HPLC to determine the enantiomeric excess: 98% e.e. (CHIRALPAK IC-3, *n*-hexane/*i*-PrOH = 95/5, flow rate: 1.0 mL/min, T = 20 °C, 250 nm), t_R (major) = 14.19 min, t_R (minor) = 17.27 min.

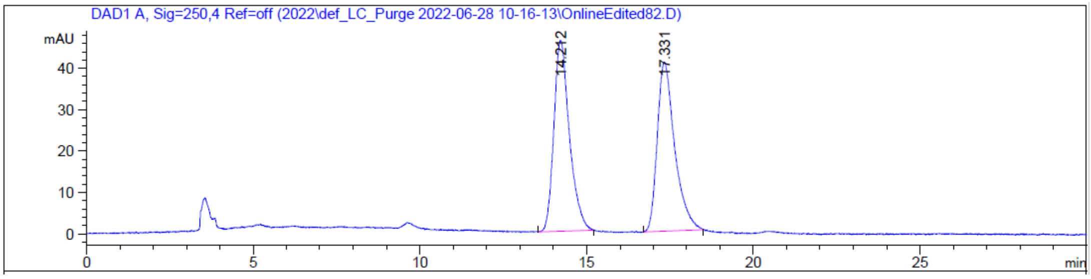

Peak #	RetTime [min]	Type	Width [min]	Area [mAU*s]	Height [mAU]	Area %
1	14.212	VV R	0.3842	1495.28015	46.12429	50.2314
2	17.331	VV R	0.4422	1481.50366	40.84553	49.7686

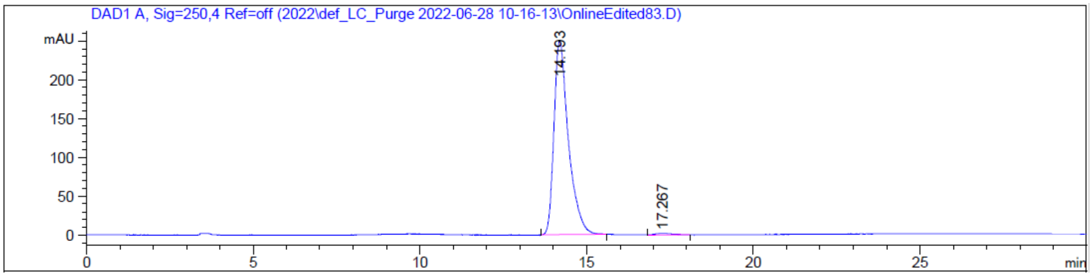

Peak #	RetTime [min]	Type	Width [min]	Area [mAU*s]	Height [mAU]	Area %
1	14.193	BV R	0.4138	7518.99854	250.06334	99.0295
2	17.267	MM R	0.5549	73.68989	2.21334	0.9705

(*R*$_a$, *S*)-1-(2-Isopropylphenyl)-2-(1-(*p*-tolyl)ethyl)-1*H*-indole-3-carbaldehyde (21)

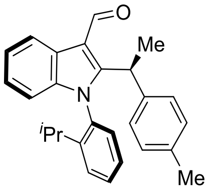

The **general procedure 4** was followed using indole substrate **1a** (0.1 mmol, 36.8 mg) and 1-methyl-4-vinylbenzene (**2d**) (0.15 mmol, 20 μL) to afford **21** (25.6 mg, 67% yield, >95:5 d.r.) as a colorless oil. **^{1}H NMR (400 MHz, CDCl$_3$)** δ 10.24 (s, 1H), 8.43 (d, *J* = 8.0 Hz, 1H), 7.60 – 7.51 (m, 2H), 7.34 – 7.26 (m, 2H), 7.19 (ddd, *J* = 8.3, 7.1, 1.2 Hz, 1H), 7.10 – 7.04 (m, 5H), 6.79 (d, *J* = 8.2 Hz, 1H), 4.16 (q, *J* = 7.4 Hz, 1H), 2.47 (hept, *J* = 6.8 Hz, 1H), 2.30 (s, 3H), 1.85 (d, *J* = 7.4 Hz, 3H), 1.21 (d, *J* = 6.9 Hz, 3H), 1.06 (d, *J* = 6.8 Hz, 3H). **^{13}C NMR (101 MHz, CDCl$_3$)** δ 186.5 (CH), 155.3 (C$_q$), 148.0 (C$_q$), 139.9 (C$_q$), 138.4 (C$_q$), 136.5 (C$_q$), 133.4 (C$_q$), 130.5 (CH), 129.5 (CH), 129.4 (CH), 127.5 (CH), 127.2 (CH), 127.1 (CH), 126.0 (C$_q$), 123.7 (CH), 123.5 (CH), 122.2 (CH), 114.8 (C$_q$),

111.3 (CH), 36.7 (CH), 27.9 (CH), 24.8 (CH_3), 23.8 (CH_3), 22.8 (CH_3), 21.1 (CH_3). **IR** (ATR) $\tilde{\nu}$ = 3055, 3026, 2965, 2925, 2869, 2829, 1650, 1513, 1458, 1398, 1234, 1079, 756, 743 cm^{-1}. **HRMS** (ESI) m/z $(M+H)^+$: calculated for $(C_{27}H_{28}NO)^+$: 382.2165, found: 382.2165; $[\alpha]_D^{20}$ = +12.2 (c = 0.50, $CHCl_3$); The product was analyzed by HPLC to determine the enantiomeric excess: 97% e.e. (CHIRALPAK IC-3, *n*-hexane/*i*-PrOH = 95/5, flow rate: 1.0 mL/min, T = 20 °C, 250 nm), t_R (major) = 19.31 min, t_R (minor) = 28.04 min.

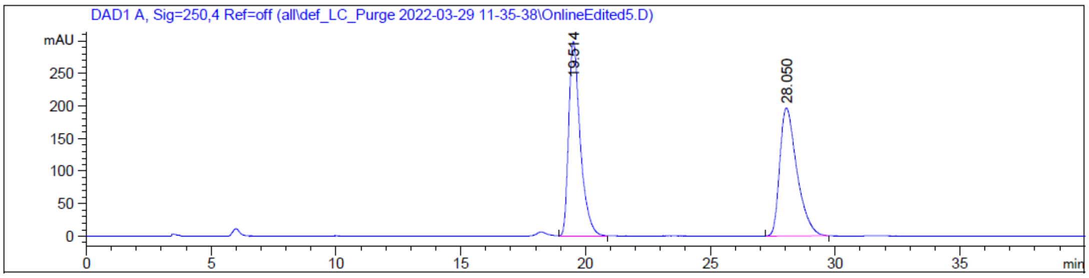

Peak #	RetTime [min]	Type	Width [min]	Area [mAU*s]	Height [mAU]	Area %
1	19.514	BB	0.4673	9602.81348	298.54993	50.4905
2	28.050	BB	0.5652	9416.22461	196.81165	49.5095

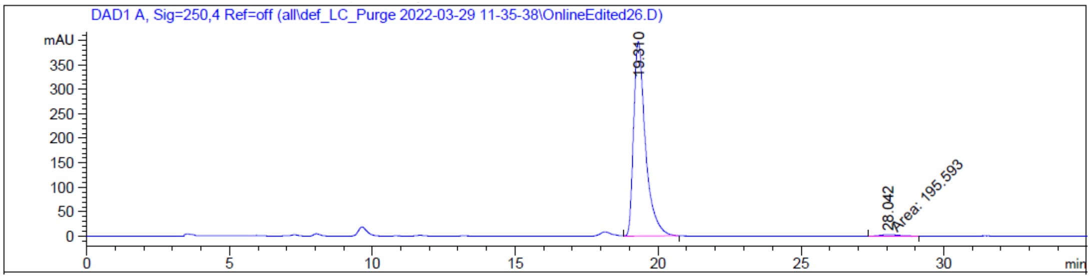

Peak #	RetTime [min]	Type	Width [min]	Area [mAU*s]	Height [mAU]	Area %
1	19.310	BB	0.4214	1.14235e4	397.37830	98.3166
2	28.042	MM T	0.9072	195.59253	3.59345	1.6834

(*R*$_a$, *S*)-1-(2-Isopropylphenyl)-2-(1-(4-methoxyphenyl)ethyl)-1*H*-indole-3-carbaldehyde (22)

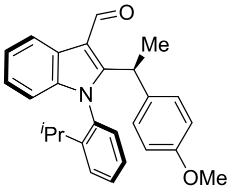

The **general procedure 4** was followed using indole substrate **1a** (0.1 mmol, 36.8 mg) and 1-methoxy-4-vinylbenzene (**2e**) (0.15 mmol, 20 µL) to afford **22** (32.6 mg, 82% yield, >95:5 d.r.) as a

pale yellow solid. **M.p.**: 112-114 °C. **^{1}H NMR (400 MHz, CDCl$_3$)** δ 10.26 (s, 1H), 8.43 (d, *J* = 7.9 Hz, 1H), 7.61 – 7.50 (m, 2H), 7.34 – 7.27 (m, 2H), 7.19 (ddd, *J* = 8.3, 7.2, 1.2 Hz, 1H), 7.11 – 7.04 (m, 3H), 6.79 (d, *J* = 8.7 Hz, 3H), 4.15 (q, *J* = 7.4 Hz, 1H), 3.77 (s, 3H), 2.47 (hept, *J* = 6.9 Hz, 1H), 1.85 (d, *J* = 7.4 Hz, 3H), 1.21 (d, *J* = 6.9 Hz, 3H), 1.06 (d, *J* = 6.8 Hz, 3H). **^{13}C NMR (101 MHz, CDCl$_3$)** δ 186.4 (CH), 158.4 (C_q), 155.3 (C_q), 147.9 (C_q), 138.3 (C_q), 135.0 (C_q), 133.4 (C_q), 130.5 (CH), 129.5 (CH), 128.2 (CH), 127.5 (CH), 127.2 (CH), 126.0 (C_q), 123.7 (CH), 123.5 (CH), 122.1 (CH), 114.8 (C_q), 114.1 (CH), 111.3 (CH), 55.4 (CH_3), 36.4 (CH), 27.9 (CH), 24.8 (CH_3), 23.8 (CH_3), 22.9 (CH_3). **IR** (ATR) $\tilde{v}$ = 2965, 2934, 2869, 2835, 1650, 1511, 1458, 1397, 1249, 1180, 1032, 757 cm^{-1}. **HRMS** (ESI) m/z $(M+H)^+$: calculated for $(C_{27}H_{28}NO_2)^+$: 398.2115, found: 398.2116; **$[\alpha]_D^{20}$** = +2.4 (c = 0.72, CHCl$_3$); The product was analyzed by HPLC to determine the enantiomeric excess: 98% e.e. (CHIRALPAK IC-3, *n*-hexane/*i*-PrOH = 85/15, flow rate: 1.0 mL/min, T = 20 °C, 250 nm), t_R (major) = 17.29 min, t_R (minor) = 25.97 min.

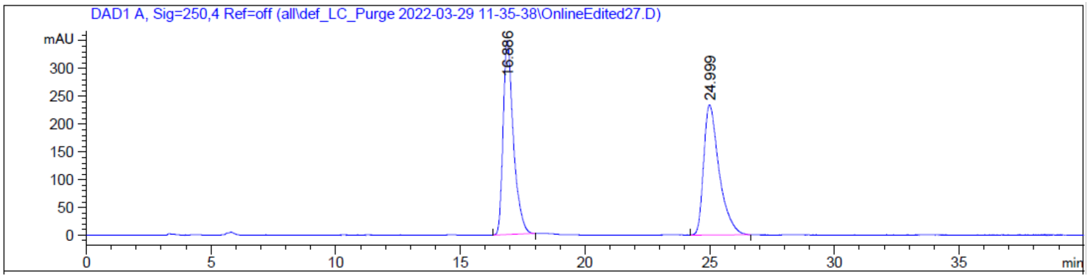

Peak #	RetTime [min]	Type	Width [min]	Area [mAU*s]	Height [mAU]	Area %
1	16.886	BV R	0.4236	9987.18066	349.23053	49.8071
2	24.999	BB	0.5737	1.00645e4	234.03767	50.1929

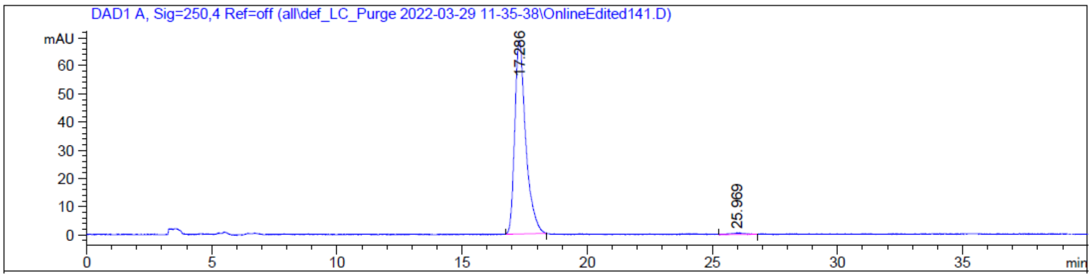

Peak #	RetTime [min]	Type	Width [min]	Area [mAU*s]	Height [mAU]	Area %
1	17.286	BB	0.4165	2087.96631	68.72413	98.9250
2	25.969	MM R	0.6810	22.68983	5.55293e-1	1.0750

(R_a, S)-2-(1-(4-(*tert*-Butyl)phenyl)ethyl)-1-(2-isopropylphenyl)-1*H*-indole-3-carbaldehyde (23)

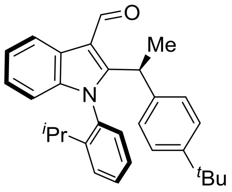

The **general procedure 4** was followed using indole substrate **1a** (0.1 mmol, 36.8 mg) and 1-(*tert*-butyl)-4-vinylbenzene (**2f**) (0.15 mmol, 27 µL) to afford **23** (40.0 mg, 95% yield, >95:5 d.r.) as a colorless oil. **^{1}H NMR (400 MHz, CDCl$_3$)** δ 10.22 (s, 1H), 8.42 (d, J = 7.8 Hz, 1H), 7.60 – 7.50 (m, 2H), 7.35 – 7.24 (m, 4H), 7.19 (t, J = 7.7 Hz, 1H), 7.17 – 7.07 (m, 3H), 6.80 (d, J = 8.1 Hz, 1H), 4.19 (q, J = 7.4 Hz, 1H), 2.47 (hept, J = 6.9 Hz, 1H), 1.86 (d, J = 7.4 Hz, 3H), 1.28 (s, 9H), 1.20 (d, J = 6.9 Hz, 3H), 1.06 (d, J = 6.8 Hz, 3H). **^{13}C NMR (101 MHz, CDCl$_3$)** δ 186.5 (CH), 155.6 (C$_q$), 149.7 (C$_q$), 148.0 (C$_q$), 139.7 (C$_q$), 138.4 (C$_q$), 133.4 (C$_q$), 130.5 (CH), 129.5 (CH), 127.5 (CH), 127.2 (CH), 126.8 (CH), 126.0 (C$_q$), 125.6 (CH), 123.7 (CH), 123.5 (CH), 122.1 (CH), 114.6 (C$_q$), 111.3 (CH), 36.4 (CH), 34.5 (C$_q$), 31.4 (CH$_3$), 27.9 (CH), 24.9 (CH$_3$), 23.7 (CH$_3$), 22.5 (CH$_3$). **IR** (ATR) $\tilde{\nu}$ = 3055, 2964, 2868, 2828, 1650, 1458, 1399, 1234, 1079, 753 cm^{-1}. **HRMS** (ESI) m/z (M+H)$^+$: calculated for $(C_{30}H_{34}NO)^+$: 424.2635, found: 424.2632; **$[\alpha]_D^{20}$** = –11.5 (c = 0.95, CHCl$_3$); The product was analyzed by HPLC to determine the enantiomeric excess: 99% e.e. (CHIRALPAK IC-3, *n*-hexane/*i*-PrOH = 95/5, flow rate: 1.0 mL/min, T = 20 °C, 250 nm), t$_R$ (major) = 13.65 min, t$_R$ (minor) = 16.29 min.

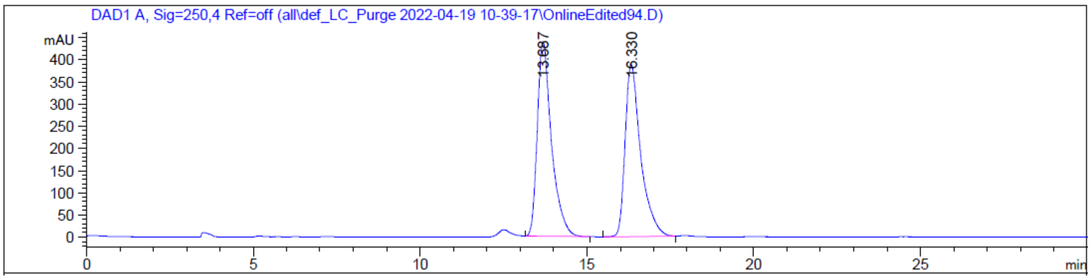

Peak #	RetTime [min]	Type	Width [min]	Area [mAU*s]	Height [mAU]	Area %
1	13.687	BV R	0.4251	1.30137e4	438.73117	50.7901
2	16.330	VV R	0.4660	1.26088e4	388.13202	49.2099

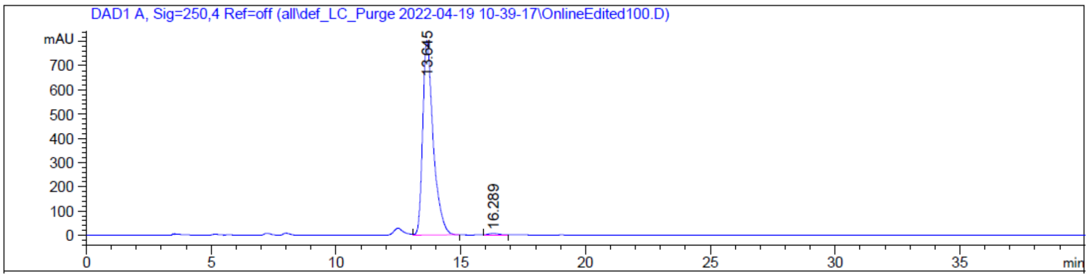

Peak #	RetTime [min]	Type	Width [min]	Area [mAU*s]	Height [mAU]	Area %
1	13.645	FM R	0.4880	2.35261e4	803.42334	99.3879
2	16.289	BB	0.2996	144.88678	5.73754	0.6121

(*R*$_a$, *S*)-2-(1-([1,1'-Biphenyl]-4-yl)ethyl)-1-(2-isopropylphenyl)-1*H*-indole-3-carbaldehyde (24)

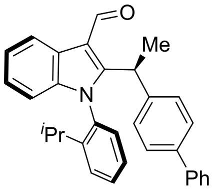

The **general procedure 4** was followed using indole substrate **1a** (0.1 mmol, 36.8 mg) and 4-vinyl-1,1'-biphenyl (**2g**) (0.15 mmol, 27.0 mg) to afford **24** (41.2 mg, 93% yield, >95:5 d.r.) as a pale yellow oil. **^{1}H NMR (400 MHz, CDCl$_3$)** δ 10.27 (s, 1H), 8.41 (d, J = 7.9 Hz, 1H), 7.57 – 7.48 (m, 4H), 7.45 (d, J = 8.3 Hz, 2H), 7.37 (dd, J = 8.4, 6.6 Hz, 2H), 7.33 – 7.25 (m, 2H), 7.24 – 7.11 (m, 4H), 7.06 (d, J = 7.8 Hz, 1H), 6.77 (d, J = 8.2 Hz, 1H), 4.21 (q, J = 7.4 Hz, 1H), 2.44 (hept, J = 6.9 Hz, 1H), 1.87 (d, J = 7.4 Hz, 3H), 1.18 (d, J = 6.9 Hz, 3H), 1.03 (d, J = 6.8 Hz, 3H). **^{13}C NMR (101 MHz, CDCl$_3$)** δ 186.3 (CH), 154.8 (C$_q$), 148.0 (C$_q$), 141.9 (C$_q$), 140.6 (C$_q$), 139.8 (C$_q$), 138.4 (C$_q$), 133.3 (C$_q$), 130.6 (CH), 129.5 (CH), 128.9 (CH), 127.6 (CH), 127.5 (CH), 127.4 (CH), 127.4 (CH), 127.3 (CH), 127.1 (CH), 126.0 (C$_q$), 123.8 (CH), 123.6 (CH), 122.1 (CH), 114.9 (C$_q$), 111.3 (CH), 36.8 (CH), 28.0 (CH), 24.8 (CH$_3$), 23.8 (CH$_3$), 22.6 (CH$_3$). **IR** (ATR) $\tilde{\nu}$ = 3056, 3028, 2965, 2935, 2869, 2829, 1650, 1492, 1458, 1399, 1234, 1079, 752, 697 cm^{-1}. **HRMS** (ESI) m/z (M+H)$^+$: calculated for $(C_{32}H_{30}NO)^+$: 444.2322, found: 444.2325; **$[\alpha]_D^{20}$** = –41.4 (c = 1.26, CHCl$_3$); The product was analyzed by HPLC to determine the enantiomeric excess: 96% e.e. (CHIRALPAK ID-3, *n*-hexane/*i*-PrOH = 95/5, flow rate: 1.0 mL/min, T = 20 °C, 250 nm), t$_R$ (minor) = 18.84 min, t$_R$ (major) = 23.78 min.

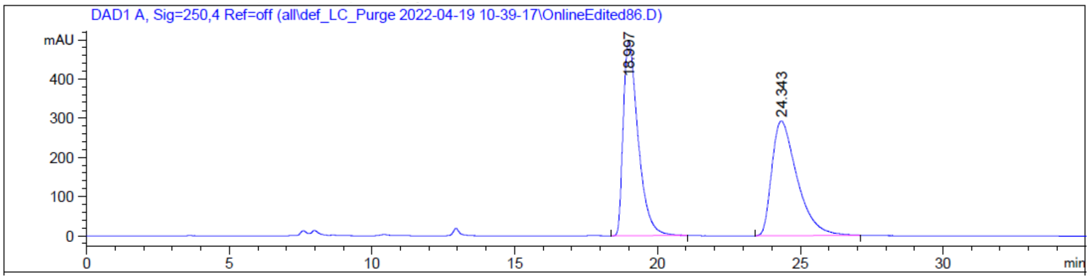

Peak #	RetTime [min]	Type	Width [min]	Area [mAU*s]	Height [mAU]	Area %
1	18.997	VV R	0.5181	1.79894e4	496.04370	50.0347
2	24.343	BV R	0.7241	1.79644e4	292.26779	49.9653

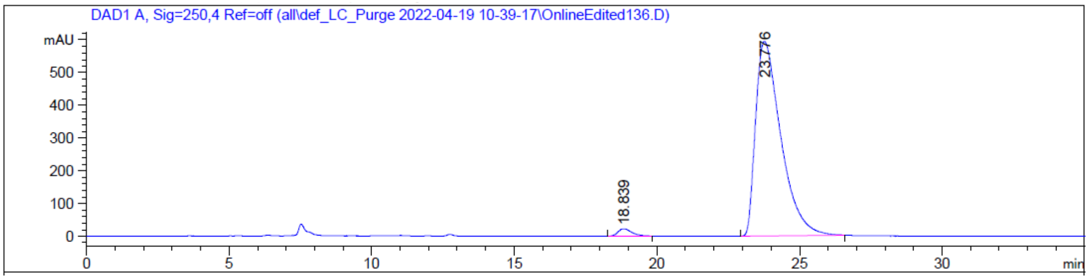

Peak #	RetTime [min]	Type	Width [min]	Area [mAU*s]	Height [mAU]	Area %
1	18.839	VV R	0.3904	749.79285	22.75704	2.0047
2	23.776	BV R	0.7292	3.66525e4	594.53192	97.9953

(*R*$_a$, *S*)-2-(1-(4-(Dimethylamino)phenyl)ethyl)-1-(2-isopropylphenyl)-1*H*-indole-3-carbaldehyde (25)

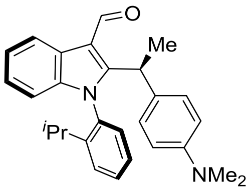

The **general procedure 4** was followed using indole substrate **1a** (0.1 mmol, 36.8 mg) and *N*,*N*-dimethyl-4-vinylaniline (**2h**) (0.15 mmol, 22.0 mg) to afford **25** (26.1 mg, 64% yield, >95:5 d.r.) as a colorless oil. **^{1}H NMR (400 MHz, CDCl$_3$)** δ 10.26 (s, 1H), 8.43 (d, *J* = 7.9 Hz, 1H), 7.60 – 7.50 (m, 2H), 7.35 – 7.25 (m, 2H), 7.18 (t, *J* = 7.7 Hz, 1H), 7.08 (d, *J* = 7.8 Hz, 1H), 7.03 (d, *J* = 8.3 Hz, 2H), 6.78 (d, *J* = 8.2 Hz, 1H), 6.62 (d, *J* = 8.3 Hz, 2H), 4.11 (q, *J* = 7.4 Hz, 1H), 2.90 (s, 6H), 2.47 (hept, *J* = 6.9 Hz, 1H), 1.82 (d, *J* = 7.4 Hz, 3H), 1.20 (d, *J* = 6.8 Hz, 3H), 1.06 (d, *J* = 6.7 Hz, 3H). **^{13}C NMR (101 MHz, CDCl$_3$)** δ 186.7 (CH), 156.1 (C$_q$), 149.5 (C$_q$), 147.9 (C$_q$), 138.3 (C$_q$), 133.5 (C$_q$),

130.6 (C_q), 130.4 (CH), 129.6 (CH), 127.8 (CH), 127.4 (CH), 127.2 (CH), 126.1 (C_q), 123.6 (CH), 123.4 (CH), 122.2 (CH), 114.7 (C_q), 112.8 (CH), 111.2 (CH), 40.7 (CH_3), 36.2 (CH), 27.9 (CH), 24.8 (CH_3), 23.8 (CH_3), 22.9 (CH_3). **IR** (ATR) $\tilde{v}$ = 2964, 2926, 2869, 1650, 1613, 1521, 1458, 1398, 1347, 1235, 754 cm^{-1}. **HRMS** (ESI) m/z $(M+H)^+$: calculated for $(C_{28}H_{31}N_2O)^+$: 411.2431, found: 411.2435; $[\alpha]_D^{20}$ = –9.6 (c = 0.44, $CHCl_3$); The product was analyzed by HPLC to determine the enantiomeric excess: 99% e.e. (CHIRALPAK ID-3, *n*-hexane/*i*-PrOH = 90/10, flow rate: 1.0 mL/min, T = 20 °C, 250 nm), t_R (minor) = 14.36 min, t_R (major) = 15.50 min.

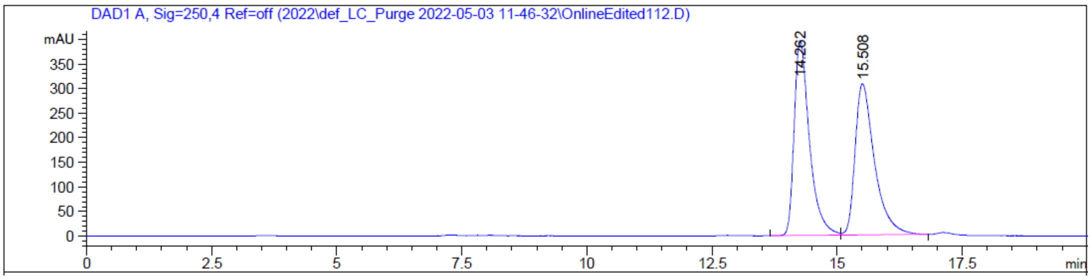

Peak #	RetTime [min]	Type	Width [min]	Area [mAU*s]	Height [mAU]	Area %
1	14.262	VV R	0.3177	8414.94238	396.30756	50.3247
2	15.508	VV R	0.3912	8306.35059	307.48627	49.6753

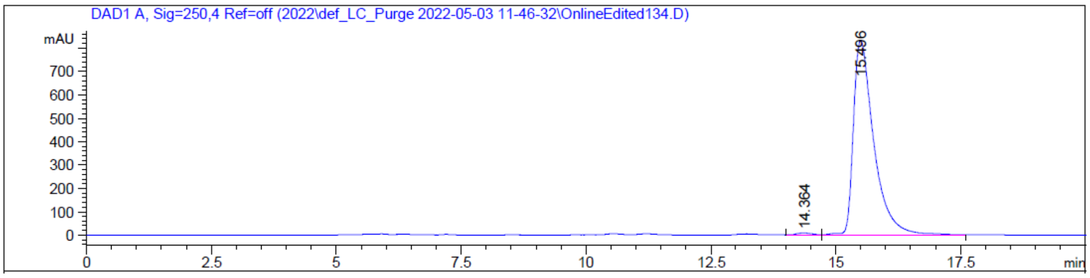

Peak #	RetTime [min]	Type	Width [min]	Area [mAU*s]	Height [mAU]	Area %
1	14.364	BB	0.2153	158.12140	8.81514	0.6659
2	15.496	VV R	0.4048	2.35885e4	831.61981	99.3341

(*Ra*, *S*)-1-(2-Isopropylphenyl)-2-(1-(*m*-tolyl)ethyl)-1*H*-indole-3-carbaldehyde (26)

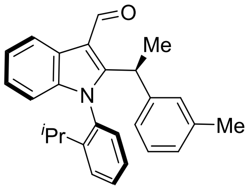

The **general procedure 4** was followed using indole substrate **1a** (0.1 mmol, 36.8 mg) and 1-methyl-3-vinylbenzene (**2i**) (0.15 mmol, 20 µL) to afford **26** (34.0 mg, 89% yield, >95:5 d.r.) as a colorless

oil. **^{1}H NMR (400 MHz, $CDCl_3$)** δ 10.26 (s, 1H), 8.44 (d, *J* = 7.9 Hz, 1H), 7.62 – 7.47 (m, 2H), 7.36 – 7.23 (m, 2H), 7.25 – 7.08 (m, 2H), 7.10 – 6.91 (m, 4H), 6.80 (d, *J* = 8.1 Hz, 1H), 4.17 (q, *J* = 7.4 Hz, 1H), 2.45 (hept, *J* = 6.8 Hz, 1H), 2.27 (s, 3H), 1.86 (d, *J* = 7.4 Hz, 3H), 1.20 (d, *J* = 6.9 Hz, 3H), 1.06 (d, *J* = 6.8 Hz, 3H). **^{13}C NMR (101 MHz, $CDCl_3$)** δ 186.5 (CH), 155.1 (C_q), 147.9 (C_q), 142.7 (C_q), 138.4 (C_q), 133.3 (C_q), 130.5 (CH), 129.5 (CH), 128.6 (CH), 127.9 (CH), 127.6 (CH), 127.5 (CH), 127.2 (CH), 126.0 (C_q), 124.3 (CH), 123.7 (CH), 123.5 (CH), 122.2 (CH), 114.9 (C_q), 111.3 (CH), 37.1 (CH), 27.9 (CH), 24.8 (CH_3), 23.8 (CH_3), 22.8 (CH_3), 21.7 (CH_3). **IR** (ATR) $\tilde{\nu}$ = 3055, 2964, 2926, 2869, 2828, 1650, 1458, 1398, 1230, 1077, 755 cm^{-1}. **HRMS** (ESI) m/z $(M+H)^+$: calculated for $(C_{27}H_{28}NO)^+$: 382.2165, found: 382.2164; **$[\alpha]_D^{20}$** = +24.4 (c = 1.04, $CHCl_3$); The product was analyzed by HPLC to determine the enantiomeric excess: 96% e.e. (CHIRALPAK ID-3, *n*-hexane/*i*-PrOH = 95/5, flow rate: 1.0 mL/min, T = 20 °C, 250 nm), t_R (minor) = 10.90 min, t_R (major) = 12.55 min.

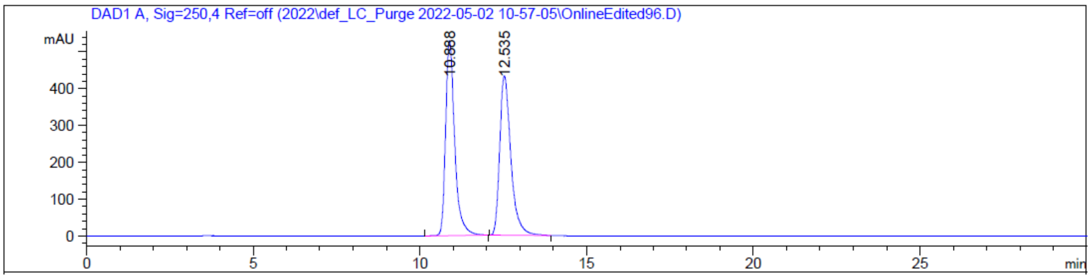

Peak #	RetTime [min]	Type	Width [min]	Area [mAU*s]	Height [mAU]	Area %
1	10.888	VB R	0.2870	1.00294e4	529.38129	49.9323
2	12.535	BB	0.3340	1.00566e4	432.96744	50.0677

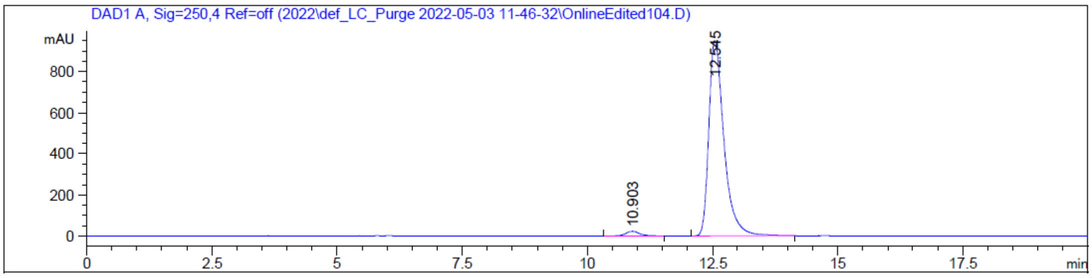

Peak #	RetTime [min]	Type	Width [min]	Area [mAU*s]	Height [mAU]	Area %
1	10.903	VV R	0.2356	446.99133	23.11295	2.1882
2	12.545	BV R	0.3133	1.99805e4	950.36420	97.8118

(R_a, S)-1-(2-Isopropylphenyl)-2-(1-(3-methoxyphenyl)ethyl)-1*H*-indole-3-carbaldehyde (27)

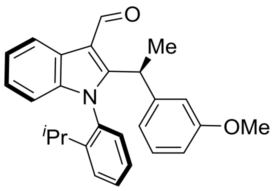

The **general procedure 4** was followed using indole substrate **1a** (0.1 mmol, 36.8 mg) and 1-methoxy-3-vinylbenzene (**2j**) (0.15 mmol, 21 μL) to afford **27** (35.5 mg, 89% yield, >95:5 d.r.) as a white solid. **M.p.**: 120-122 °C. **^{1}H NMR (400 MHz, $CDCl_3$)** δ 10.26 (s, 1H), 8.43 (d, *J* = 7.9 Hz, 1H), 7.61 – 7.51 (m, 2H), 7.31 (q, *J* = 7.2 Hz, 2H), 7.19 (q, *J* = 7.4 Hz, 2H), 7.09 (d, *J* = 7.8 Hz, 1H), 6.83 – 6.68 (m, 4H), 4.17 (q, *J* = 7.4 Hz, 1H), 3.74 (s, 3H), 2.47 (hept, *J* = 6.9 Hz, 1H), 1.86 (d, *J* = 7.4 Hz, 3H), 1.21 (d, *J* = 6.8 Hz, 3H), 1.06 (d, *J* = 6.7 Hz, 3H). **^{13}C NMR (101 MHz, $CDCl_3$)** δ 186.3 (CH), 159.9 (C_q), 154.8 (C_q), 148.0 (C_q), 144.5 (C_q), 138.4 (C_q), 133.3 (C_q), 130.6 (CH), 129.7 (CH), 129.5 (CH), 127.5 (CH), 127.2 (CH), 126.0 (C_q), 123.8 (CH), 123.5 (CH), 122.2 (CH), 119.6 (CH), 114.9 (C_q), 113.7 (CH), 111.6 (CH), 111.3 (CH), 55.3 (CH_3), 37.0 (CH), 27.9 (CH), 24.8 (CH_3), 23.8 (CH_3), 22.56 (CH_3). **IR** (ATR) $\tilde{\nu}$ = 3055, 2964, 2936, 2869, 2835, 1650, 1608, 1493, 1457, 1398, 1234, 1044, 756, 742 cm^{-1}. **HRMS** (ESI) m/z $(M+H)^+$: calculated for $(C_{27}H_{28}NO_2)^+$: 398.2115, found: 398.2118; $[\alpha]_D^{20}$ = +15.1 (c = 0.88, $CHCl_3$); The product was analyzed by HPLC to determine the enantiomeric excess: 94% e.e. (CHIRALPAK ID-3, *n*-hexane/*i*-PrOH = 95/5, flow rate: 1.0 mL/min, T = 20 °C, 250 nm), t_R (minor) = 19.36 min, t_R (major) = 23.05 min.

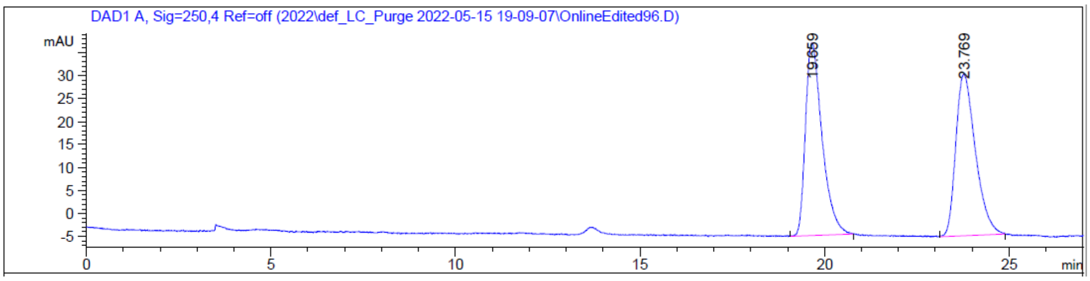

Peak #	RetTime [min]	Type	Width [min]	Area [mAU*s]	Height [mAU]	Area %
1	19.659	VV R	0.3723	1312.69592	41.92653	50.2375
2	23.769	BB	0.4412	1300.28625	35.17894	49.7625

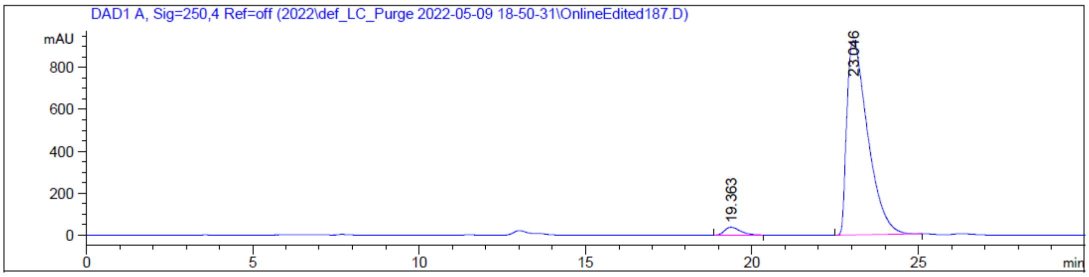

Peak #	RetTime [min]	Type	Width [min]	Area [mAU*s]	Height [mAU]	Area %
1	19.363	BV R	0.3898	1231.28174	37.81913	2.9668
2	23.046	VV R	0.5844	4.02702e4	925.91632	97.0332

(R_a, *S*)-2-(1-(2-Fluorophenyl)ethyl)-1-(2-isopropylphenyl)-1*H*-indole-3-carbaldehyde (28)

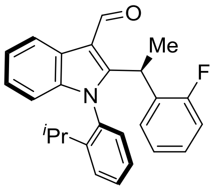

The **general procedure 4** was followed using indole substrate **1a** (0.1 mmol, 36.8 mg) and 1-fluoro-2-vinylbenzene (**2k**) (0.15 mmol, 18 μL) to afford **28** (13.9 mg, 36% yield, >95:5 d.r.) as a colorless oil. **^{1}H NMR (400 MHz, $CDCl_3$)** δ 10.30 (s, 1H), 8.44 (d, *J* = 7.9 Hz, 1H), 7.59 – 7.49 (m, 2H), 7.38 – 7.26 (m, 2H), 7.27 – 7.14 (m, 3H), 7.09 (t, *J* = 7.6 Hz, 1H), 6.98 (d, *J* = 7.8 Hz, 1H), 6.96 – 6.90 (m, 1H), 6.77 (d, *J* = 8.2 Hz, 1H), 4.42 (q, *J* = 7.4 Hz, 1H), 2.43 (hept, *J* = 6.9, 6.5 Hz, 1H), 1.84 (d, *J* = 7.3 Hz, 3H), 1.22 (d, *J* = 6.8 Hz, 3H), 1.04 (d, *J* = 6.8 Hz, 3H). **^{13}C NMR (101 MHz, $CDCl_3$)** δ 186.2 (CH), 160.6 (d, *J* = 247.0 Hz, C_q), 153.4 (C_q), 147.8 (C_q), 138.4 (C_q), 133.2 (C_q), 130.5 (CH), 129.8 (d, *J* = 14.1 Hz, C_q), 129.5 (d, *J* = 4.0 Hz, CH), 128.9 (d, *J* = 8.4 Hz, CH), 128.3 (d, *J* = 3.8 Hz, CH), 127.4 (CH), 127.1 (CH), 125.9 (C_q), 124.5 (d, *J* = 3.6 Hz, CH), 123.9 (CH), 123.5 (CH), 122.2 (CH), 115.7 (d, *J* = 22.0 Hz, CH), 115.7 (C_q), 111.3 (CH), 32.0 (d, *J* = 2.4 Hz, CH), 28.0 (CH), 24.9 (CH_3), 23.7 (CH_3), 21.6 (CH_3). **^{19}F NMR (377 MHz, $CDCl_3$)** δ -115.67. **IR** (ATR) $\tilde{\nu}$ = 2967, 2936, 2925, 1653, 1491, 1458, 1398, 1228, 754 cm^{-1}. **HRMS** (ESI) m/z $(M+H)^+$: calculated for $(C_{26}H_{25}FNO)^+$: 386.1915, found: 386.1912; **$[\alpha]_D^{20}$** = +21.3 (c = 0.15, $CHCl_3$); The product was analyzed by HPLC to determine the enantiomeric excess: 99% e.e. (CHIRALPAK IC-3, *n*-hexane/*i*-PrOH = 95/5, flow rate: 1.0 mL/min, T = 20 °C, 250 nm), t_R (major) = 18.21 min, t_R (minor) = 19.06 min.

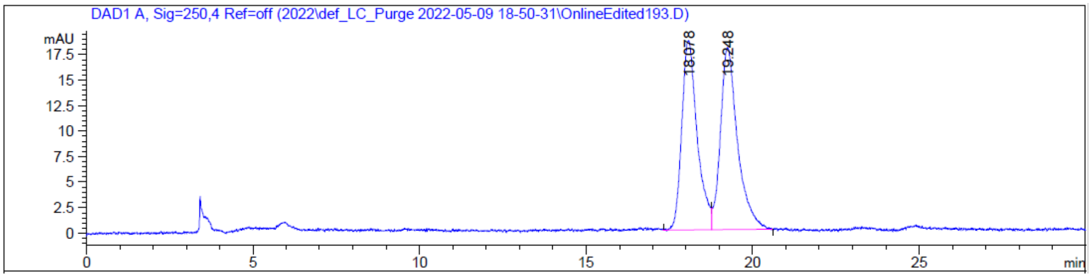

Peak #	RetTime [min]	Type	Width [min]	Area [mAU*s]	Height [mAU]	Area %
1	18.078	MF R	0.5290	600.65942	18.58783	48.3040
2	19.248	FM R	0.5728	642.83777	17.88596	51.6960

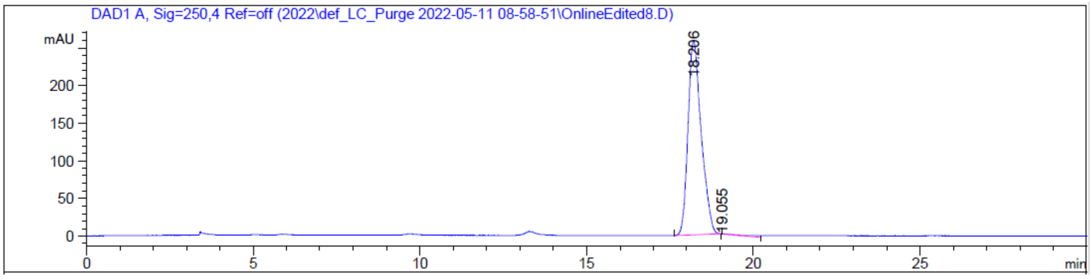

Peak #	RetTime [min]	Type	Width [min]	Area [mAU*s]	Height [mAU]	Area %
1	18.206	BV R	0.3961	7105.26563	258.12714	99.4992
2	19.055	MM R	0.2764	35.76384	1.25239e-1	0.5008

(*R*$_a$, *S*)-1-(2-Isopropylphenyl)-2-(1-(*o*-tolyl)ethyl)-1*H*-indole-3-carbaldehyde (29)

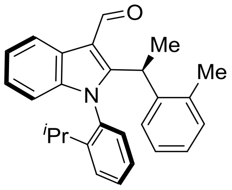

The **general procedure 4** was followed using indole substrate **1a** (0.1 mmol, 36.8 mg) and 1-methyl-2-vinylbenzene (**2l**) (0.15 mmol, 19 µL) to afford **29** (32.5 mg, 85% yield, >95:5 d.r.) as a pale yellow solid. **M.p.**: 123-125 °C. **^{1}H NMR (400 MHz, CDCl$_3$)** δ 10.69 (s, 1H), 8.55 (d, *J* = 7.9 Hz, 1H), 7.57 – 7.46 (m, 2H), 7.32 (t, *J* = 7.5 Hz, 1H), 7.27 – 7.22 (m, 1H), 7.17 (t, *J* = 7.7 Hz, 1H), 7.13 – 6.95 (m, 4H), 6.71 (d, *J* = 8.2 Hz, 1H), 6.39 (d, *J* = 8.3 Hz, 1H), 4.38 (q, *J* = 7.4 Hz, 1H), 2.37 (hept, *J* = 7.2 Hz, 1H), 1.82 (d, *J* = 7.5 Hz, 3H), 1.68 (s, 3H), 1.21 (d, *J* = 6.9 Hz, 3H), 1.03 (d, *J* = 6.8 Hz, 3H). **^{13}C NMR (101 MHz, CDCl$_3$)** δ 187.0 (CH), 153.5 (C$_q$), 147.6 (C$_q$), 141.1 (C$_q$), 138.4 (C$_q$), 135.2 (C$_q$), 133.4 (C$_q$), 130.5 (CH), 130.3 (CH), 129.5 (CH), 127.5 (CH), 127.2 (CH), 126.9 (CH), 126.9 (CH),

126.8 (CH), 126.0 (C_q), 123.9 (CH), 123.4 (CH), 122.3 (CH), 117.5 (C_q), 110.8 (CH), 36.4 (CH), 27.9 (CH), 24.5 (CH_3), 23.6 (CH_3), 22.4 (CH_3), 18.4 (CH_3). **IR** (ATR) $\tilde{\nu}$ = 3052, 3016, 2965, 2927, 2869, 1650, 1492, 1461, 1398, 1236, 1077, 758 cm^{-1}. **HRMS** (ESI) m/z $(M+H)^+$: calculated for $(C_{27}H_{28}NO)^+$: 382.2165, found: 382.2170; **$[\alpha]_D^{20}$** = +278.1 (c = 0.75, $CHCl_3$); The product was analyzed by HPLC to determine the enantiomeric excess: 99% e.e. (CHIRALPAK ID-3, *n*-hexane/*i*-PrOH = 95/5, flow rate: 1.0 mL/min, T = 20 °C, 250 nm), t_R (minor) = 10.98 min, t_R (major) = 14.90 min.

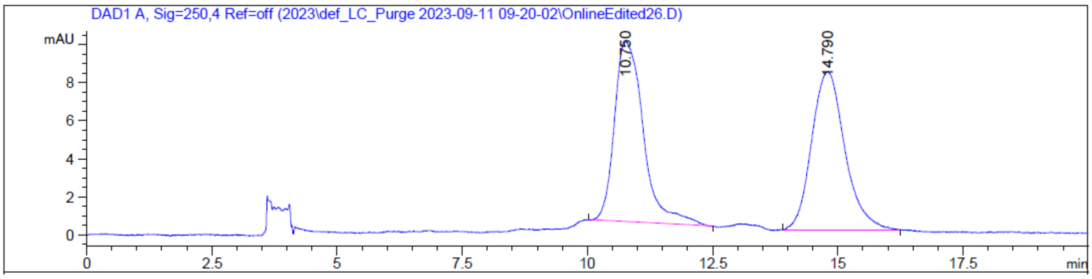

Peak #	RetTime [min]	Type	Width [min]	Area [mAU*s]	Height [mAU]	Area %
1	10.750	MM R	0.6830	381.91635	9.51910	49.8688
2	14.790	MM R	0.7690	383.92520	8.32140	50.1312

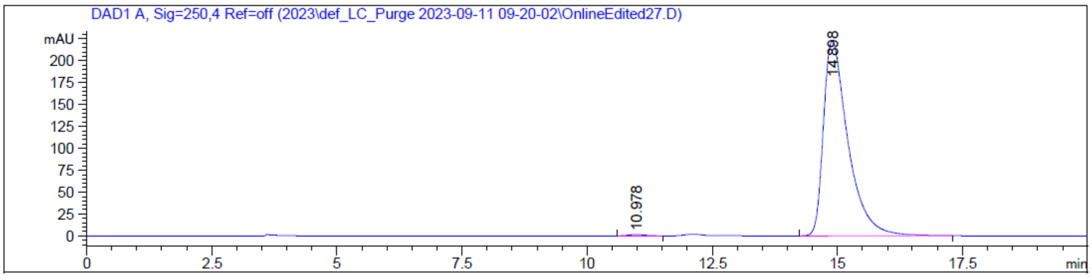

Peak #	RetTime [min]	Type	Width [min]	Area [mAU*s]	Height [mAU]	Area %
1	10.978	BB	0.2618	35.50762	1.61307	0.4645
2	14.898	BB	0.5043	7608.73682	222.86345	99.5355

(*R*$_a$, *S*)-1-(2-Isopropylphenyl)-2-(1-(2-methoxyphenyl)ethyl)-1*H*-indole-3-carbaldehyde (30)

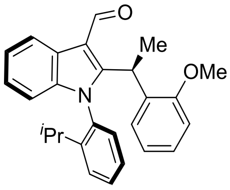

The **general procedure 4** was followed using indole substrate **1a** (0.1 mmol, 36.8 mg) and

1-methoxy-2-vinylbenzene (**2m**) (0.15 mmol, 20 μL) to afford **30** (21.8 mg, 55% yield, >95:5 d.r.) as a pale yellow oil. **^{1}H NMR (400 MHz, CDCl$_3$)** δ 10.36 (s, 1H), 8.47 (d, *J* = 8.1 Hz, 1H), 7.57 – 7.45 (m, 2H), 7.29 (ddd, *J* = 8.0, 7.1, 1.0 Hz, 1H), 7.23 – 7.12 (m, 4H), 6.92 – 6.81 (m, 2H), 6.73 (dd, *J* = 8.2, 1.3 Hz, 2H), 4.54 (q, *J* = 7.4 Hz, 1H), 3.53 (s, 3H), 2.41 (hept, *J* = 6.9 Hz, 1H), 1.76 (d, *J* = 7.4 Hz, 3H), 1.20 (d, *J* = 6.9 Hz, 3H), 1.03 (d, *J* = 6.8 Hz, 3H). **^{13}C NMR (101 MHz, CDCl$_3$)** δ 186.9 (CH), 156.7 (C$_q$), 155.3 (C$_q$), 147.8 (C$_q$), 138.5 (C$_q$), 133.6 (C$_q$), 131.1 (C$_q$), 130.1 (CH), 129.7 (CH), 128.3 (CH), 128.0 (CH), 127.2 (CH), 126.6 (CH), 125.9 (C$_q$), 123.6 (CH), 123.2 (CH), 122.2 (CH), 120.9 (CH), 116.1 (C$_q$), 111.0 (CH), 110.8 (CH), 55.4 (CH$_3$), 32.6 (CH), 28.0 (CH), 24.9 (CH$_3$), 23.6 (CH$_3$), 21.5 (CH$_3$). **IR** (ATR) $\tilde{\nu}$ = 3055, 2965, 2935, 2870, 2836, 1650, 1492, 1459, 1397, 1246, 1078, 1029, 752 cm^{-1}. **HRMS** (ESI) m/z (M+H)$^+$: calculated for $(C_{27}H_{28}NO_2)^+$: 398.2115, found: 398.2110; **$[\alpha]_D^{20}$** = +106.5 (c = 0.34, CHCl$_3$); The product was analyzed by HPLC to determine the enantiomeric excess: 99% e.e. (CHIRALPAK IC-3, *n*-hexane/*i*-PrOH = 95/5, flow rate: 1.0 mL/min, T = 20 °C, 250 nm), t_R (major) = 24.28 min, t_R (minor) = 27.07 min.

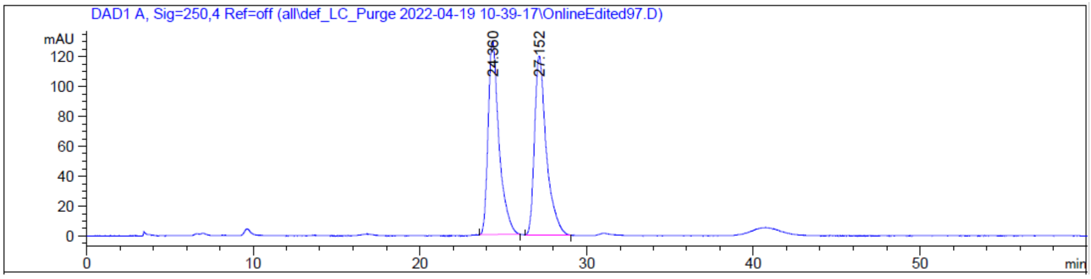

Peak #	RetTime [min]	Type	Width [min]	Area [mAU*s]	Height [mAU]	Area %
1	24.360	BB	0.5445	5880.65283	129.81349	49.8400
2	27.152	VV R	0.5906	5918.39844	119.75993	50.1600

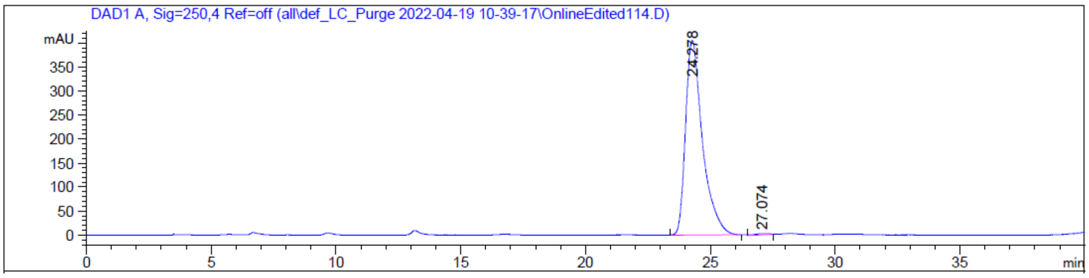

Peak #	RetTime [min]	Type	Width [min]	Area [mAU*s]	Height [mAU]	Area %
1	24.278	VV R	0.5804	1.90974e4	404.79926	99.4180
2	27.074	MM R	0.6548	111.78906	2.71600	0.5820

(R_a, S)-2-(1-(3,4-Dimethoxyphenyl)ethyl)-1-(2-isopropylphenyl)-1*H*-indole-3-carbaldehyde (31)

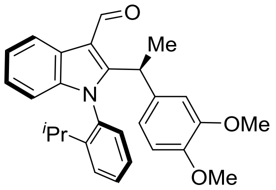

The **general procedure 4** was followed using indole substrate **1a** (0.1 mmol, 36.8 mg) and 1,2-dimethoxy-4-vinylbenzene (**2n**) (0.15 mmol, 22 µL) to afford **31** (40.0 mg, 94% yield, >95:5 d.r.) as a pale yellow oil. **^{1}H NMR (400 MHz, $CDCl_3$)** δ 10.35 (s, 1H), 8.43 (d, *J* = 7.9 Hz, 1H), 7.61 – 7.51 (m, 2H), 7.36 – 7.24 (m, 2H), 7.19 (t, *J* = 7.7 Hz, 1H), 7.02 (d, *J* = 7.8 Hz, 1H), 6.79 (d, *J* = 8.2 Hz, 1H), 6.79 – 6.68 (m, 2H), 6.65 (s, 1H), 4.14 (q, *J* = 7.4 Hz, 1H), 3.83 (s, 3H), 3.77 (s, 3H), 2.46 (hept, *J* = 7.0 Hz, 1H), 1.86 (d, *J* = 7.4 Hz, 3H), 1.21 (d, *J* = 6.9 Hz, 3H), 1.06 (d, *J* = 6.7 Hz, 3H). **^{13}C NMR (101 MHz, $CDCl_3$)** δ 186.3 (CH), 154.8 (C_q), 149.0 (C_q), 148.0 (C_q), 138.3 (C_q), 135.4 (C_q), 133.4 (C_q), 130.5 (CH), 129.5 (CH), 127.5 (CH), 127.1 (CH), 126.1 (C_q), 123.8 (CH), 123.5 (CH), 122.1 (CH), 119.4 (CH), 114.9 (C_q), 111.3 (CH), 111.3 (CH), 110.8 (CH), 56.0 (CH_3), 56.0 (CH_3), 37.0 (CH), 28.0 (CH), 24.8 (CH_3), 23.7 (CH_3), 22.9 (CH_3). **IR** (ATR) $\tilde{\nu}$ = 2966, 2935, 2869, 2834, 1650, 1517, 1459, 1406, 1254, 1234, 1146, 1028, 757 cm^{-1}. **HRMS** (ESI) m/z $(M+H)^+$: calculated for $(C_{28}H_{30}NO_3)^+$: 428.2220, found: 428.2223; **$[\alpha]_D^{20}$** = +53.5 (c = 0.65, $CHCl_3$); The product was analyzed by HPLC to determine the enantiomeric excess: 96% e.e. (CHIRALPAK ID-3, *n*-hexane/*i*-PrOH = 90/10, flow rate: 1.0 mL/min, T = 20 °C, 250 nm), t_R (minor) = 34.37 min, t_R (major) = 39.20 min.

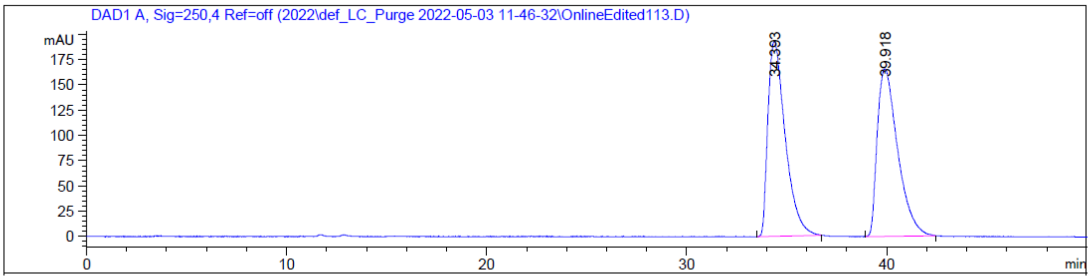

Peak #	RetTime [min]	Type	Width [min]	Area [mAU*s]	Height [mAU]	Area %
1	34.393	VV R	0.7101	1.16478e4	192.99297	49.7852
2	39.918	BV R	0.8323	1.17483e4	165.87726	50.2148

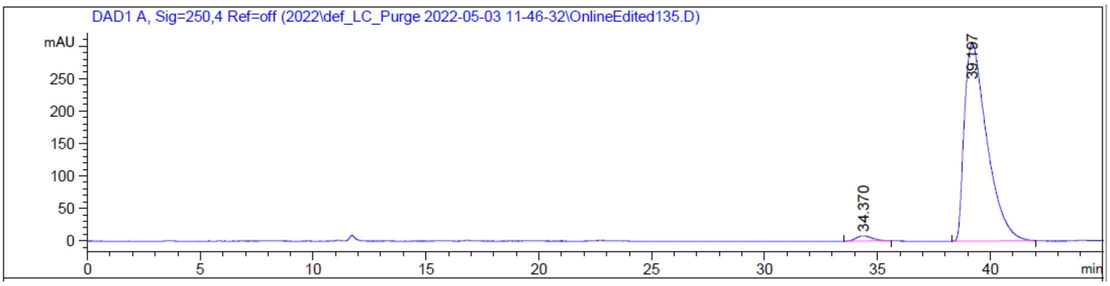

Peak #	RetTime [min]	Type	Width [min]	Area [mAU*s]	Height [mAU]	Area %
1	34.370	MM R	0.8405	409.96179	8.12966	1.9007
2	39.197	VV R	0.8235	2.11590e4	304.95441	98.0993

(R_a, *S*)-1-(2-Isopropylphenyl)-2-(1-(naphthalen-2-yl)ethyl)-1*H*-indole-3-carbaldehyde (32)

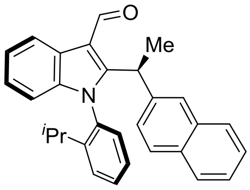

The **general procedure 4** was followed using indole substrate **1a** (0.1 mmol, 36.8 mg) and 2-vinylnaphthalene (**2o**) (0.15 mmol, 23.1 mg) to afford **32** (38.7 mg, 93% yield, >95:5 d.r.) as a colorless oil. **^{1}H NMR (400 MHz, $CDCl_3$)** δ 10.32 (s, 1H), 8.48 (d, *J* = 7.9 Hz, 1H), 7.81 – 7.77 (m, 1H), 7.74 (d, *J* = 8.1 Hz, 2H), 7.65 – 7.53 (m, 3H), 7.51 – 7.41 (m, 2H), 7.35 (t, *J* = 7.5 Hz, 1H), 7.31 – 7.18 (m, 3H), 7.07 (d, *J* = 7.8 Hz, 1H), 6.83 (d, *J* = 8.2 Hz, 1H), 4.37 (q, *J* = 7.4 Hz, 1H), 2.51 (hept, *J* = 6.8 Hz, 1H), 1.99 (d, *J* = 7.3 Hz, 3H), 1.23 (d, *J* = 6.8 Hz, 3H), 1.09 (d, *J* = 6.7 Hz, 3H). **^{13}C NMR (101 MHz, $CDCl_3$)** δ 186.4 (CH), 154.5 (C_q), 148.0 (C_q), 140.2 (C_q), 138.4 (C_q), 133.4 (C_q), 133.3 (C_q), 132.3 (C_q), 130.6 (CH), 129.5 (CH), 128.5 (CH), 128.0 (CH), 127.6 (CH), 127.5 (CH), 127.2 (CH), 126.4 (CH), 126.1 (CH), 126.0 (C_q), 125.9 (CH), 125.3 (CH), 123.9 (CH), 123.6 (CH), 122.2 (CH), 115.1 (C_q), 111.3 (CH), 37.4 (CH), 28.0 (CH), 24.8 (CH_3), 23.8 (CH_3), 22.7 (CH_3). **IR** (ATR) $\tilde{\nu}$ = 3056, 2965, 2935, 2869, 2829, 1650, 1458, 1397, 1233, 752 cm^{-1}. **HRMS** (ESI) m/z $(M+H)^+$: calculated for $(C_{30}H_{28}NO)^+$: 418.2165, found: 418.2168; **$[\alpha]_D^{20}$** = –48.3 (c = 1.08, $CHCl_3$); The product was analyzed by HPLC to determine the enantiomeric excess: 90% e.e. (CHIRALPAK ID-3, *n*-hexane/*i*-PrOH = 95/5, flow rate: 1.0 mL/min, T = 20 °C, 250 nm), t_R (minor) = 16.36 min, t_R (major) = 18.86 min.

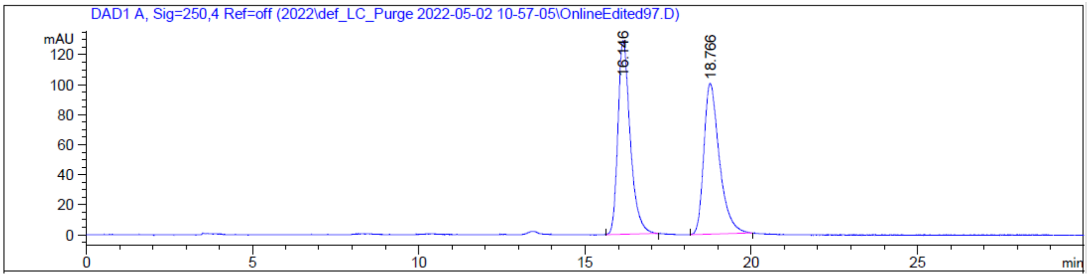

Peak #	RetTime [min]	Type	Width [min]	Area [mAU*s]	Height [mAU]	Area %
1	16.146	BV R	0.3591	3322.35010	129.17831	50.3999
2	18.766	BV R	0.4164	3269.63013	100.55051	49.6001

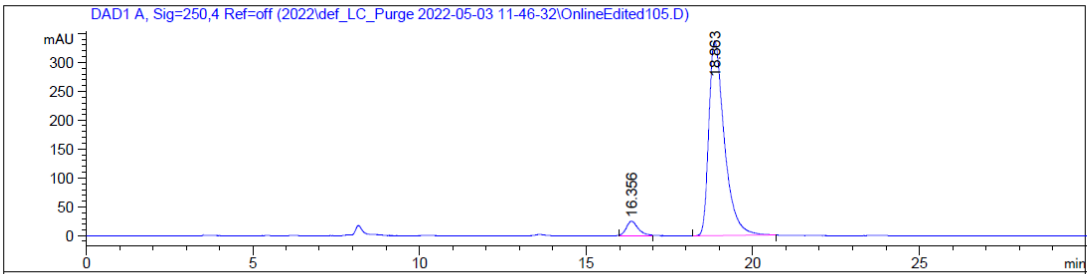

Peak #	RetTime [min]	Type	Width [min]	Area [mAU*s]	Height [mAU]	Area %
1	16.356	MM R	0.4021	592.24432	24.54876	5.1433
2	18.863	BV R	0.4726	1.09226e4	337.46854	94.8567

(*R*$_a$, *S*)-1-(2-Isopropylphenyl)-2-(1-(naphthalen-1-yl)ethyl)-1*H*-indole-3-carbaldehyde (33)

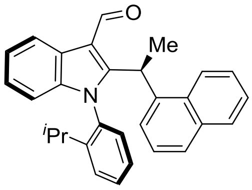

The **general procedure 4** was followed using indole substrate **1a** (0.1 mmol, 36.8 mg) and 1-vinylnaphthalene (**2p**) (0.15 mmol, 23.1 mg) to afford **33** (25.5 mg, 61% yield, 90:10 d.r.) as a colorless oil. **^{1}H NMR (400 MHz, CDCl$_3$)** δ 10.69 (s, 1H), 8.55 (d, *J* = 8.0 Hz, 1H), 7.81 (d, *J* = 8.1 Hz, 1H), 7.70 (d, *J* = 8.1 Hz, 1H), 7.50 – 7.39 (m, 4H), 7.38 – 7.25 (m, 4H), 7.19 (t, *J* = 7.7 Hz, 1H), 6.81 – 6.64 (m, 2H), 6.40 (d, *J* = 7.8 Hz, 1H), 5.06 (q, *J* = 7.5 Hz, 1H), 2.36 (hept, *J* = 6.8 Hz, 1H), 1.97 (d, *J* = 7.4 Hz, 3H), 1.15 (d, *J* = 6.8 Hz, 3H), 1.02 (d, *J* = 6.8 Hz, 3H). **^{13}C NMR (101 MHz, CDCl$_3$)** δ 186.9 (CH), 153.4 (C$_q$), 147.3 (C$_q$), 138.7 (C$_q$), 138.5 (C$_q$), 134.0 (C$_q$), 133.3 (C$_q$), 130.8 (C$_q$), 130.2 (CH), 129.1 (CH), 129.0 (CH), 127.8 (CH), 127.1 (CH), 126.8 (CH), 126.4 (CH),

126.1 (C_q), 125.7 (CH), 125.6 (CH), 125.5 (CH), 124.0 (CH), 123.5 (CH), 122.2 (CH), 122.1 (CH), 117.2 (C_q), 111.1 (CH), 35.2 (CH), 28.0 (CH), 24.5 (CH_3), 23.7 (CH_3), 23.2 (CH_3). **IR** (ATR) $\tilde{\nu}$ = 3056, 2966, 2936, 2925, 1651, 1459, 1396, 757 cm^{-1}. **HRMS** (ESI) m/z $(M+H)^+$: calculated for $(C_{30}H_{28}NO)^+$: 418.2165, found: 418.2169; **$[\alpha]_D^{20}$** = +255.3 (c = 0.30, $CHCl_3$); The product was analyzed by HPLC to determine the enantiomeric excess: 93% e.e. (CHIRALPAK IC-3, *n*-hexane/*i*-PrOH = 95/5, flow rate: 1.0 mL/min, T = 20 °C, 250 nm), t_R (minor) = 19.67 min, t_R (major) = 21.72 min.

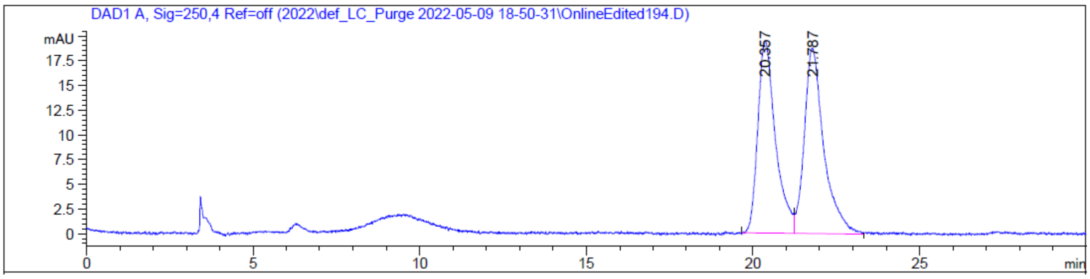

Peak #	RetTime [min]	Type	Width [min]	Area [mAU*s]	Height [mAU]	Area %
1	20.357	MF R	0.5902	720.93054	19.47984	48.5480
2	21.787	FM R	0.6406	764.05444	18.80372	51.4520

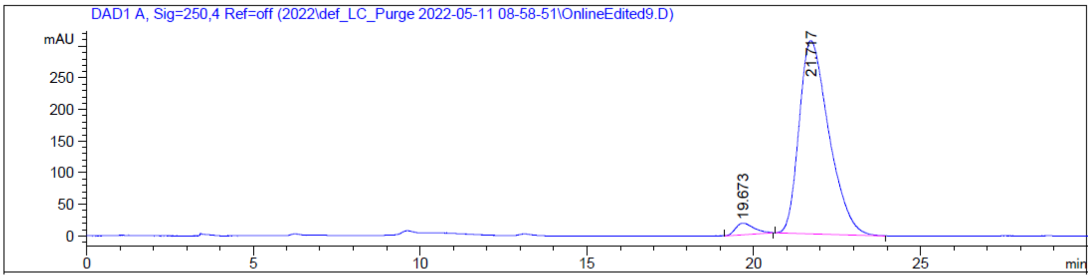

Peak #	RetTime [min]	Type	Width [min]	Area [mAU*s]	Height [mAU]	Area %
1	19.673	BV R	0.4440	686.79803	18.21240	3.4945
2	21.717	VV R	0.7348	1.89671e4	305.73471	96.5055

(*R*$_a$, *R*)-2-(1-Ferrocenylethyl)-1-(2-isopropylphenyl)-1*H*-indole-3-carbaldehyde (34)

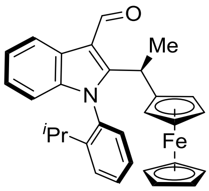

The **general procedure 4** was followed using indole substrate **1a** (0.1 mmol, 36.8 mg) and vinyl

ferrocene (**2q**) (0.15 mmol, 31.8 mg) to afford **34** (29.0 mg, 61% yield, >95:5 d.r.) as a brown oil. **^{1}H NMR (400 MHz, CDCl$_3$)** δ 10.22 (s, 1H), 8.35 (d, *J* = 7.9 Hz, 1H), 7.69 – 7.58 (m, 2H), 7.49 (t, *J* = 6.8 Hz, 1H), 7.33 (d, *J* = 7.7 Hz, 1H), 7.32 – 7.24 (m, 1H), 7.17 (s, 1H), 6.79 (d, *J* = 8.2 Hz, 1H), 4.34 (s, 1H), 4.12 – 4.02 (m, 3H), 4.01 – 3.90 (m, 6H), 2.49 (hept, *J* = 6.9 Hz, 1H), 1.78 (d, *J* = 7.4 Hz, 3H), 1.25 (d, *J* = 6.9 Hz, 3H), 1.06 (d, *J* = 6.8 Hz, 3H). **^{13}C NMR (101 MHz, CDCl$_3$)** δ 186.0 (CH), 156.5 (C$_q$), 148.3 (C$_q$), 138.1 (C$_q$), 133.5 (C$_q$), 130.6 (CH), 129.6 (CH), 127.6 (CH), 127.1 (CH), 125.9 (C$_q$), 123.6 (CH), 123.5 (CH), 122.1 (CH), 113.6 (C$_q$), 111.3 (CH), 91.7 (C$_q$), 69.0 (CH), 68.6 (CH), 68.4 (CH), 67.2 (CH), 66.5 (CH), 32.1 (CH), 27.9 (CH), 25.1 (CH$_3$), 23.8 (CH$_3$), 22.0 (CH$_3$). **IR** (ATR) $\tilde{\nu}$ = 2964, 2926, 2869, 2827, 1649, 1458, 1410, 1072, 756 cm^{-1}. **HRMS** (ESI) m/z (M+H)$^+$: calculated for $(C_{30}H_{30}FeNO)^+$: 476.1671, found: 476.1669; **$[\alpha]_D^{20}$** = +330.0 (c = 0.61, CHCl$_3$); The product was analyzed by HPLC to determine the enantiomeric excess: 99% e.e. (CHIRALPAK ID-3, *n*-hexane/*i*-PrOH = 90/10, flow rate: 1.0 mL/min, T = 20 °C, 250 nm), t$_R$ (minor) = 14.55 min, t$_R$ (major) = 21.93 min.

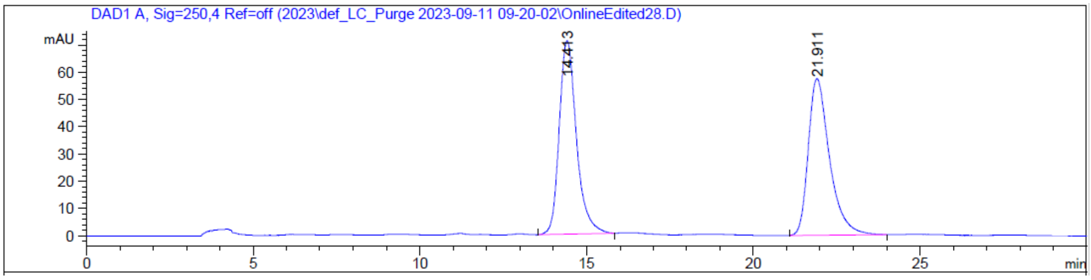

Peak #	RetTime [min]	Type	Width [min]	Area [mAU*s]	Height [mAU]	Area %
1	14.413	BB	0.5413	2545.47754	70.99490	49.9916
2	21.911	BB	0.6028	2546.32886	57.48416	50.0084

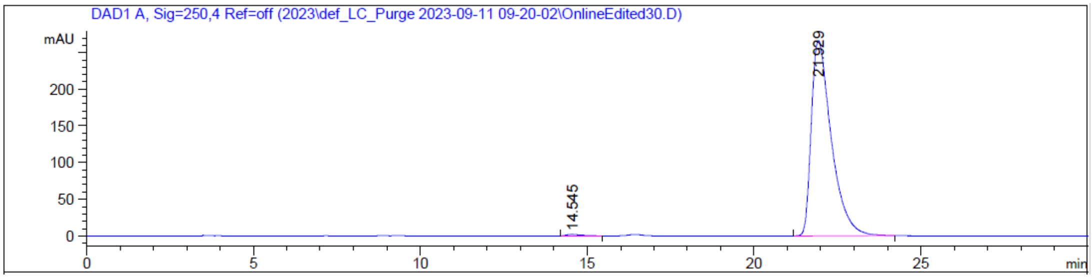

Peak #	RetTime [min]	Type	Width [min]	Area [mAU*s]	Height [mAU]	Area %
1	14.545	BB	0.3635	65.69038	2.14371	0.5887
2	21.929	BB	0.6144	1.10923e4	266.06561	99.4113

7. Scale-up reaction

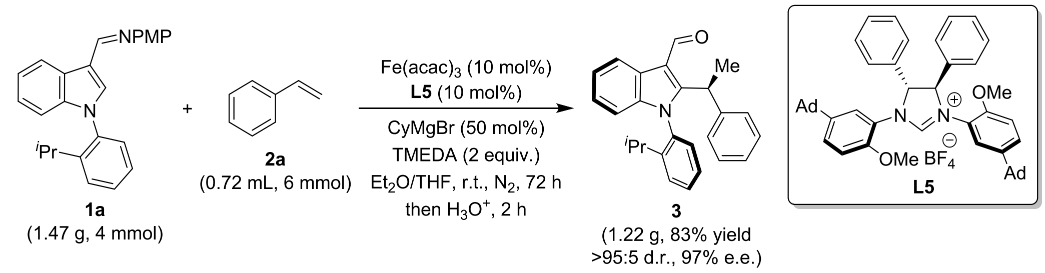

To a flame-dried and N_2-purged Schlenk tube were added indole substrate **1a** (4.0 mmol, 1.47 g), $Fe(acac)_3$ (0.4 mmol, 141.3 mg) and chiral NHC ligand **L5** (0.4 mmol, 316.2 mg). The Schlenk tube was then sealed, purged and backfilled with N_2 three times. Ethyl ether (8.0 mL), TMEDA (8.0 mmol, 1.20 mL) and styrene **2a** (6.0 mmol, 0.72 mL) were added *via* syringe. CyMgBr (1 M in THF, 2.0 mmol, 2.0 mL) was then added dropwise and the resulting mixture was stirred at room temperature for 72 hours. Then, the reaction mixture was diluted with tetrahydrofuran (40.0 mL) and quenched with HCl aqueous solution (1 M, 20.0 mL). The resulting mixture was stirred at room temperature for 2 hours. The phases were then separated, the aqueous layer was extracted with ethyl acetate (50.0 mL ×3). The combined organic layer was washed with brine, dried over Na_2SO_4, filtered and concentrated *in vacuo*. The diastereomeric ratio was determined by ^{1}H NMR analysis of the crude reaction mixture. The residue was purified by column chromatography on silica gel (*n*-hexane: ethyl acetate = 10:1) to afford the desired product **3** (1.22 g, 83% yield, >95:5 d.r., 97% e.e.).

8. Late-stage transformations

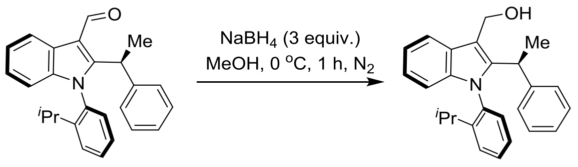

3
(>95:5 d.r., 97% e.e.)

35, 95% yield
>95:5 d.r., 97% e.e.

Under the optimal conditions, **3** was obtained in 90% yield, >95:5 d.r. and 97% e.e. To a solution of **3** (0.1 mmol, 36.7 mg) in MeOH (1.0 mL) under nitrogen, was added portionwise $NaBH_4$ (0.3 mmol, 11.3 mg) at 0 °C. The resulting mixture was stirred for 1 hours and was then quenched by NaOH aqueous solution (2 M, 1.0 mL). The solution was stirred for 10 min and then CH_2Cl_2 (5.0 mL) were added. The phases were separated, the aqueous layer was extracted with CH_2Cl_2 (5.0 mL ×3) and the combined organic layer was washed with brine, dried over Na_2SO_4 and concentrated to give **35** (35.1 mg, 95% yield, >95:5 d.r., 97% e.e.).

(*R*_a, *S*)-(1-(2-Isopropylphenyl)-2-(1-phenylethyl)-1*H*-indol-3-yl)methanol (35)

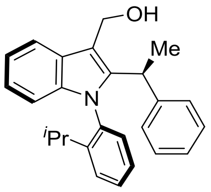

White solid; 95% yield; >95:5 d.r.; **m.p.**: 128-130 °C. **^{1}H NMR (400 MHz, $CDCl_3$)** δ 7.85 (d, *J* = 7.1 Hz, 1H), 7.65 – 7.53 (m, 2H), 7.38 – 7.16 (m, 8H), 7.08 (dd, *J* = 7.9, 1.3 Hz, 1H), 6.89 (d, *J* = 7.4 Hz, 1H), 4.98 (d, *J* = 12.3 Hz, 1H), 4.88 (d, *J* = 12.2 Hz, 1H), 4.13 (q, *J* = 7.4 Hz, 1H), 2.59 (hept, *J* = 6.9 Hz, 1H), 1.87 (d, *J* = 7.4 Hz, 3H), 1.28 (d, *J* = 6.9 Hz, 3H), 1.14 (d, *J* = 6.8 Hz, 3H). **^{13}C NMR (101 MHz, $CDCl_3$)** δ 148.6 (C_q), 144.3 (C_q), 143.2 (C_q), 138.0 (C_q), 134.8 (C_q), 130.2 (CH), 129.7 (CH), 128.5 (CH), 128.1 (C_q), 127.3 (CH), 127.1 (CH), 126.8 (CH), 126.5 (CH), 122.0 (CH), 120.3 (CH), 118.2 (CH), 111.8 (C_q), 111.0 (CH), 56.1 (CH_2), 36.5 (CH), 27.8 (CH), 25.0 (CH_3), 23.8 (CH_3), 21.1 (CH_3). **IR** (ATR) $\tilde{\nu}$ = 3358, 3057, 3027, 2964, 2925, 2869, 1493, 1458, 1363, 992, 744, 699 cm^{-1}. **HRMS** (ESI) m/z $(M+H)^+$: calculated for $(C_{26}H_{28}NO)^+$: 370.2165, found: 370.2170; $[\alpha]_D^{20}$ = –17.3 (c = 0.51, $CHCl_3$); The product was analyzed by HPLC to determine the enantiomeric excess: 97% e.e. (CHIRALPAK IC-3, *n*-hexane/*i*-PrOH = 95/5, flow rate: 1.0 mL/min, T = 20 °C, 250 nm), t_R (major) = 7.12 min, t_R (minor) = 11.42 min.

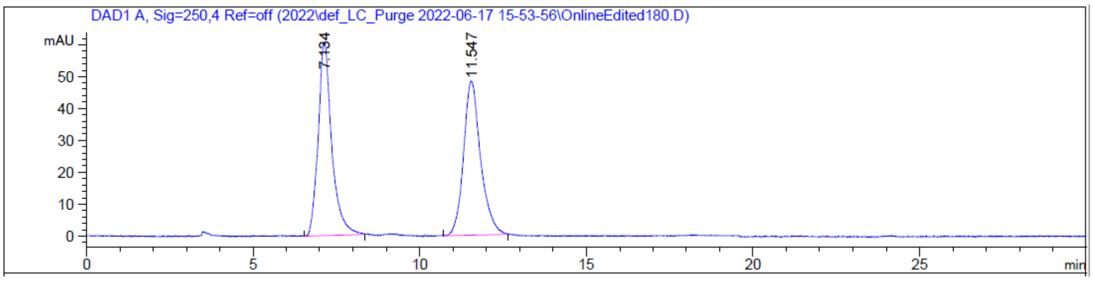

Peak #	RetTime [min]	Type	Width [min]	Area [mAU*s]	Height [mAU]	Area %
1	7.134	VV R	0.3603	1713.55603	60.71355	49.9146
2	11.547	VB R	0.4203	1719.41833	48.41754	50.0854

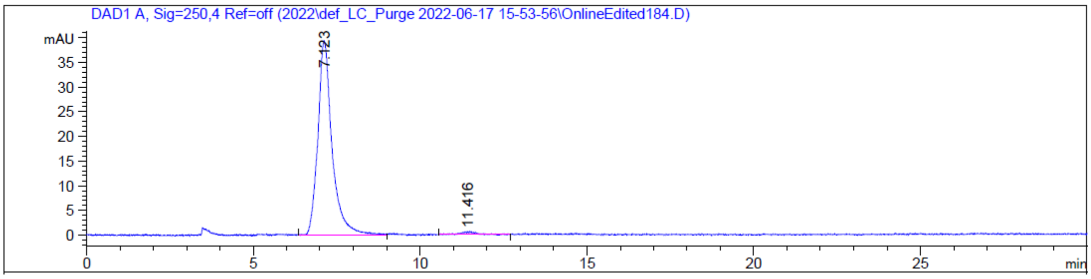

Peak #	RetTime [min]	Type	Width [min]	Area [mAU*s]	Height [mAU]	Area %
1	7.123	MM R	0.4685	1155.43262	39.35905	98.7286
2	11.416	MM R	0.4223	14.87967	5.87179e-1	1.2714

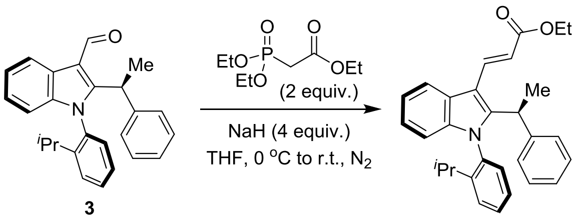

3
(>95:5 d.r., 97% e.e.)

36, 80% yield
>95:5 d.r., 97% e.e.

Under the optimal conditions, **3** was obtained in 90% yield, >95:5 d.r. and 97% e.e. To a solution of ethyl 2-(diethoxyphosphoryl)acetate (0.2 mmol, 44.8 mg) in anhydrous THF (1.0 mL) under nitrogen, was added portionwise NaH (60% dispersion in mineral oil, 0.4 mmol, 16.0 mg) at 0 °C. After stirring for 1 hours, **3** (0.1 mmol, 36.7 mg) was added and the resulting mixture was allowed to warm up to room temperature and stirred overnight. The reaction mixture was quenched by saturated NH_4Cl aqueous solution (1.0 mL). Then, diethyl ether (5 mL) and water (5 mL) were added. The phases were separated and the aqueous layer was extracted with diethyl ether (5.0 mL ×3). The combined organic layer was washed with brine, dried over Na_2SO_4, filtered and concentrated *in*

vacuum. The crude product was purified by column chromatography on silica gel (*n*-hexane: ethyl acetate = 10:1) to afford the desired product **36** (35.0 mg, 80% yield, >95:5 d.r., 97% e.e.).

Ethyl (R_a, *S*, *E*)-3-(1-(2-isopropylphenyl)-2-(1-phenylethyl)-1*H*-indol-3-yl)acrylate (36)

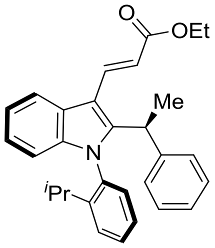

Colorless oil; 80% yield; >95:5 d.r.; **^{1}H NMR (400 MHz, $CDCl_3$)** δ 8.04 (d, *J* = 15.9 Hz, 1H), 7.98 (d, *J* = 8.0 Hz, 1H), 7.58 – 7.47 (m, 2H), 7.30 – 7.22 (m, 4H), 7.23 – 7.11 (m, 4H), 7.03 (d, *J* = 7.8 Hz, 1H), 6.80 (d, *J* = 8.1 Hz, 1H), 6.48 (d, *J* = 15.9 Hz, 1H), 4.23 (q, *J* = 7.2 Hz, 2H), 4.14 (q, *J* = 7.5 Hz, 1H), 2.48 (hept, *J* = 6.8 Hz, 1H), 1.82 (d, *J* = 7.4 Hz, 3H), 1.33 (t, *J* = 7.1 Hz, 3H), 1.18 (d, *J* = 6.8 Hz, 3H), 1.06 (d, *J* = 6.7 Hz, 3H). **^{13}C NMR (101 MHz, $CDCl_3$)** δ 168.3 (C_q), 148.2 (C_q), 143.0 (C_q), 138.9 (C_q), 138.5 (CH), 134.0 (C_q), 130.2 (CH), 129.9 (CH), 128.6 (CH), 127.3 (CH), 127.3 (CH), 127.0 (CH), 126.6 (CH), 126.2 (C_q), 122.8 (CH), 121.9 (CH), 120.6 (CH), 113.8 (CH), 111.6 (CH), 109.6 (C_q), 60.0 (CH_2), 36.8 (CH), 27.9 (CH), 24.8 (CH_3), 23.7 (CH_3), 21.4 (CH_3), 14.6 (CH_3). **IR** (ATR) $\tilde{\nu}$ = 3057, 3027, 2967, 2870, 1699, 1620, 1493, 1460, 1375, 1280, 1172, 1079, 746 cm^{-1}. **HRMS** (ESI) m/z $(M+H)^+$: calculated for $(C_{30}H_{32}NO_2)^+$: 438.2428, found: 438.2426; **$[\alpha]_D^{20}$** = +164.3 (c = 0.65, $CHCl_3$); The product was analyzed by HPLC to determine the enantiomeric excess: 97% e.e. (CHIRALPAK IC-3, *n*-hexane/*i*-PrOH = 95/5, flow rate: 1.0 mL/min, T = 20 °C, 250 nm), t_R (minor) = 7.08 min, t_R (major) = 8.09 min.

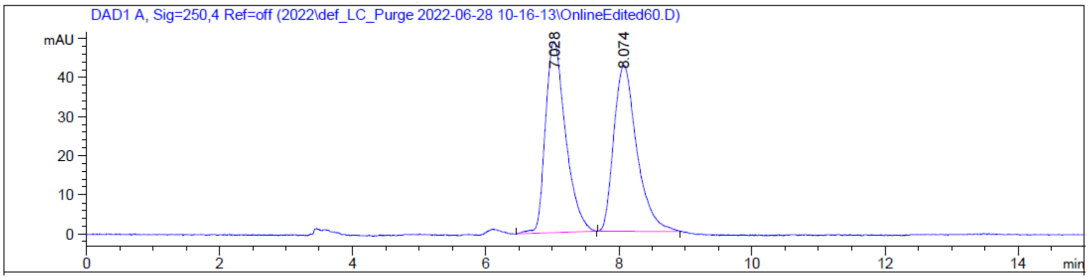

Peak #	RetTime [min]	Type	Width [min]	Area [mAU*s]	Height [mAU]	Area %
1	7.028	VB R	0.2982	1031.37463	48.88088	50.2470
2	8.074	BV R	0.2944	1021.23663	42.48494	49.7530

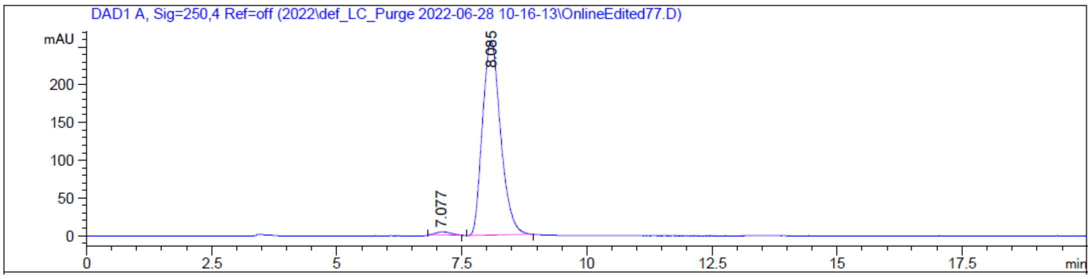

Peak #	RetTime [min]	Type	Width [min]	Area [mAU*s]	Height [mAU]	Area %
1	7.077	MM R	0.3611	84.66371	3.90819	1.2890
2	8.085	BV R	0.3847	6483.71045	257.55426	98.7110

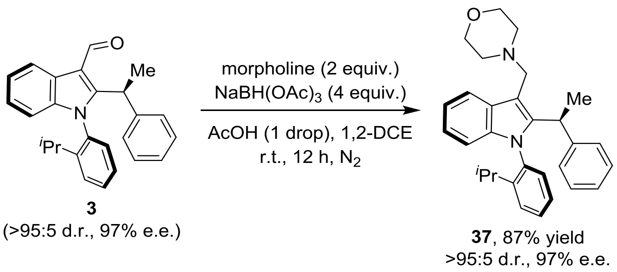

Under the optimal conditions, **3** was obtained in 90% yield, >95:5 d.r. and 97% e.e. A solution of **3** (36.7 mg, 0.1 mmol), morpholine (18 μL, 0.2 mmol), $NaBH(OAc)_3$ (84.8 mg, 0.4 mmol) and AcOH (1 drop) in 1,2-dichloroethane (1.0 mL) was stirred at room temperature for 12 hours. The reaction mixture was diluted with CH_2Cl_2 (5.0 mL), quenched by saturated $NaHCO_3$ aqueous solution (5.0 mL). The phases were separated and the aqueous layer was extracted with CH_2Cl_2 (5.0 mL ×3). The combined organic layer was washed with brine, dried over Na_2SO_4, filtered and concentrated *in vacuum*. The residue was purified by column chromatography on silica gel (*n*-hexane: ethyl acetate = 10:1) to afford the desired product **37** (38.2 mg, 87% yield, >95:5 d.r., 97% e.e.).

(R_a, *S*)-4-((1-(2-Isopropylphenyl)-2-(1-phenylethyl)-1H-indol-3-yl)methyl)morpholine (37)

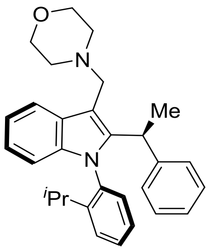

White solid; 87% yield; >95:5 d.r.; **m.p.**: 158-160 °C. **^{1}H NMR (400 MHz, $CDCl_3$)** δ 7.85 (d, *J* = 7.9 Hz, 1H), 7.54 – 7.42 (m, 2H), 7.23 – 7.03 (m, 8H), 6.93 (d, *J* = 7.4 Hz, 1H), 6.76 (d, *J* = 7.1 Hz,

1H), 4.02 (q, J = 7.3 Hz, 1H), 3.75 – 3.46 (m, 6H), 2.54 – 2.24 (m, 5H), 1.77 (d, J = 7.4 Hz, 3H), 1.17 (d, J = 6.9 Hz, 3H), 1.03 (d, J = 6.8 Hz, 3H). **^{13}C NMR (101 MHz, $CDCl_3$)** δ 148.5 (C_q), 144.3 (C_q), 142.8 (C_q), 137.8 (C_q), 135.1 (C_q), 130.5 (CH), 129.5 (CH), 129.4 (C_q), 128.1 (CH), 127.7 (CH), 126.9 (CH), 126.6 (CH), 126.1 (CH), 121.5 (CH), 119.6 (CH), 119.2 (CH), 110.6 (CH), 108.7 (C_q), 67.4 (CH_2), 53.7 (CH_2), 53.2 (CH_2), 37.0 (CH), 27.8 (CH), 25.0 (CH_3), 23.6 (CH_3), 20.2 (CH_3). **IR** (ATR) $\tilde{v}$ = 3057, 3027, 2963, 2925, 2851, 1493, 1460, 1364, 1117, 1003, 864, 742, 698 cm^{-1}. **HRMS** (ESI) m/z $(M+H)^+$: calculated for $(C_{30}H_{35}N_2O)^+$: 439.2744, found: 439.2750; $[\alpha]_D^{20}$ = +6.8 (c = 0.40, $CHCl_3$); The product was analyzed by HPLC to determine the enantiomeric excess: 97% e.e. (CHIRALPAK OD-3, *n*-hexane/*i*-PrOH = 90/10, flow rate: 1.0 mL/min, T = 20 °C, 250 nm), t_R (major) = 4.72 min, t_R (minor) = 5.05 min.

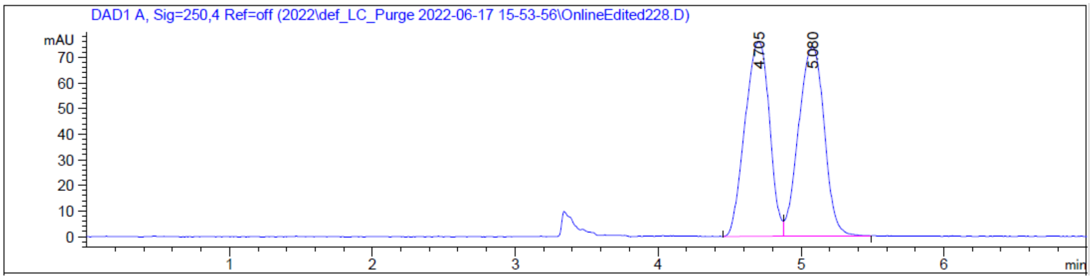

Peak #	RetTime [min]	Type	Width [min]	Area [mAU*s]	Height [mAU]	Area %
1	4.705	BV	0.1849	905.59497	76.05986	49.5260
2	5.080	VV R	0.2030	922.92853	73.75670	50.4740

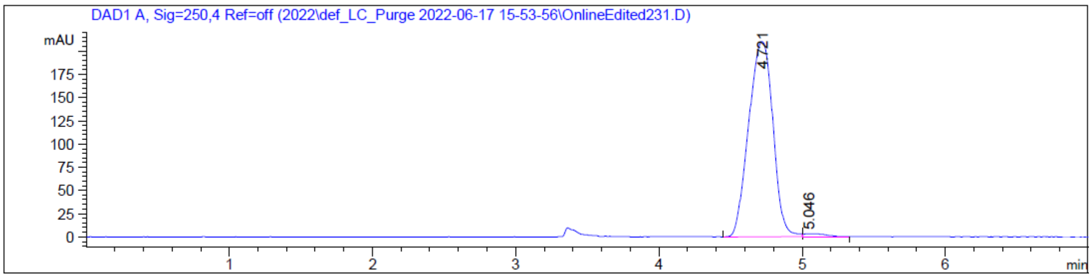

Peak #	RetTime [min]	Type	Width [min]	Area [mAU*s]	Height [mAU]	Area %
1	4.721	MF R	0.1964	2477.01318	210.22374	98.6156
2	5.046	FM R	0.1816	34.77426	3.19128	1.3844

9. Mechanistic investigations

9.1 Deuterium-labeling experiment

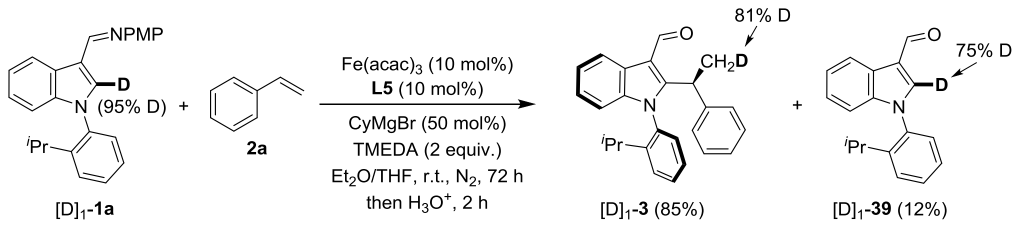

To a flame-dried and N_2-purged Schlenk tube were added indole substrate [D]$_1$-**1a** (0.1 mmol, 36.9 mg), Fe(acac)$_3$ (0.01 mmol, 3.5 mg) and chiral NHC ligand **L5** (0.01 mmol, 7.9 mg). The Schlenk tube was then sealed, purged and backfilled with N_2 three times. Ethyl ether (0.2 mL), TMEDA (0.2 mmol, 30 μL) and styrene **2a** (0.15 mmol, 17 μL) were added *via* syringe. CyMgBr (1 M in THF, 0.05 mmol, 0.05 mL) was then added dropwise and the resulting mixture was stirred at room temperature for 72 hours. Then, the reaction mixture was diluted with tetrahydrofuran (2.0 mL) and quenched with HCl aqueous solution (1 M, 1.0 mL). The resulting mixture was stirred at room temperature for 2 hours. The phases were then separated, the aqueous layer was extracted with ethyl acetate (5.0 mL ×3). The combined organic layer was washed with brine, dried over Na_2SO_4, filtered and concentrated *in vacuo*. The residue was purified by column chromatography on silica gel (*n*-hexane: ethyl acetate = 10:1) to afford the product and hydrolyzed starting material. Deuterium contents were determined by ^{1}H NMR spectroscopic analysis.

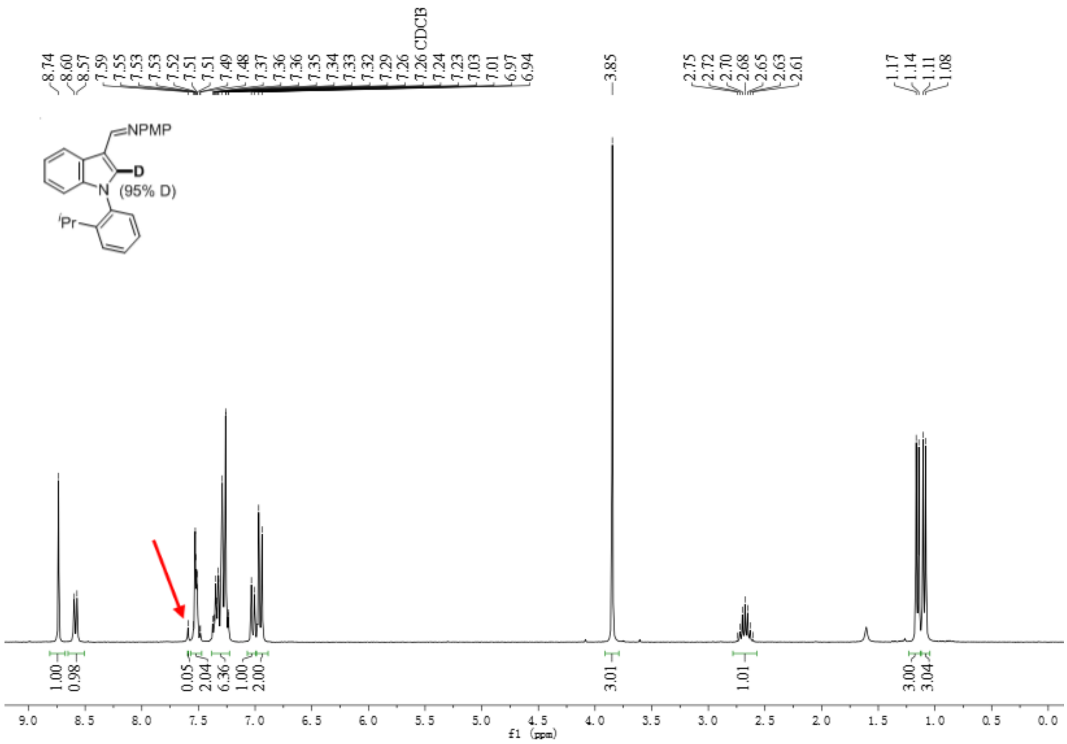

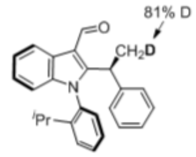
81% D
CH2D
N
iPr

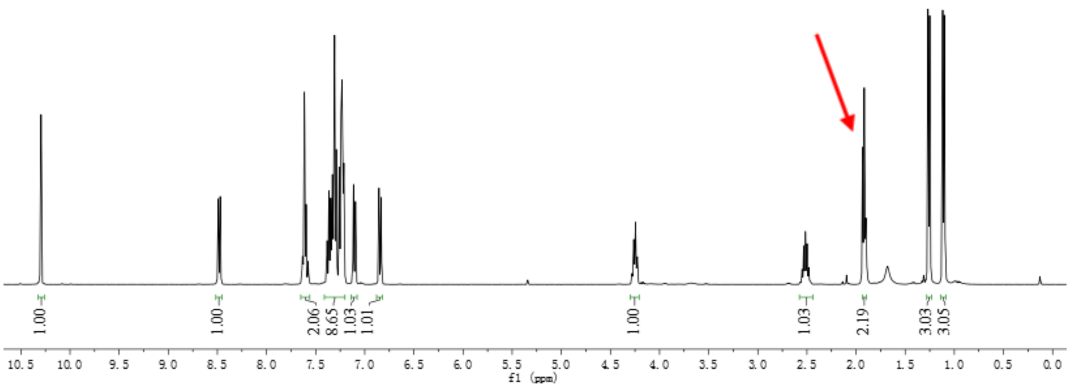
1.00
1.00
2.06
8.65
1.03
1.01
1.00
1.03
2.19
3.03
3.05
f1 (ppm)

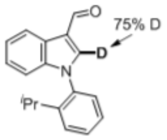
75% D
D
N
iPr

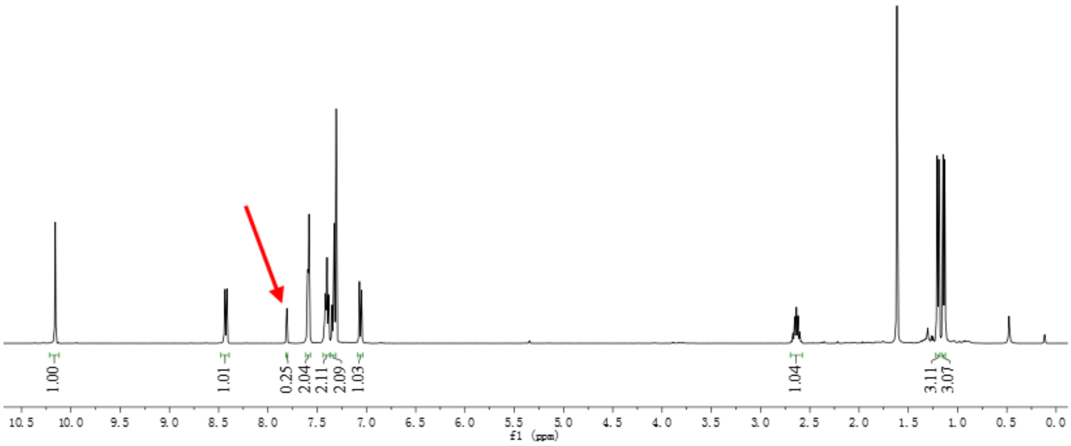
1.00
1.01
0.25
2.04
2.11
2.09
1.03
1.04
3.11
3.07
f1 (ppm)

9.2 Deuterium scrambling experiment

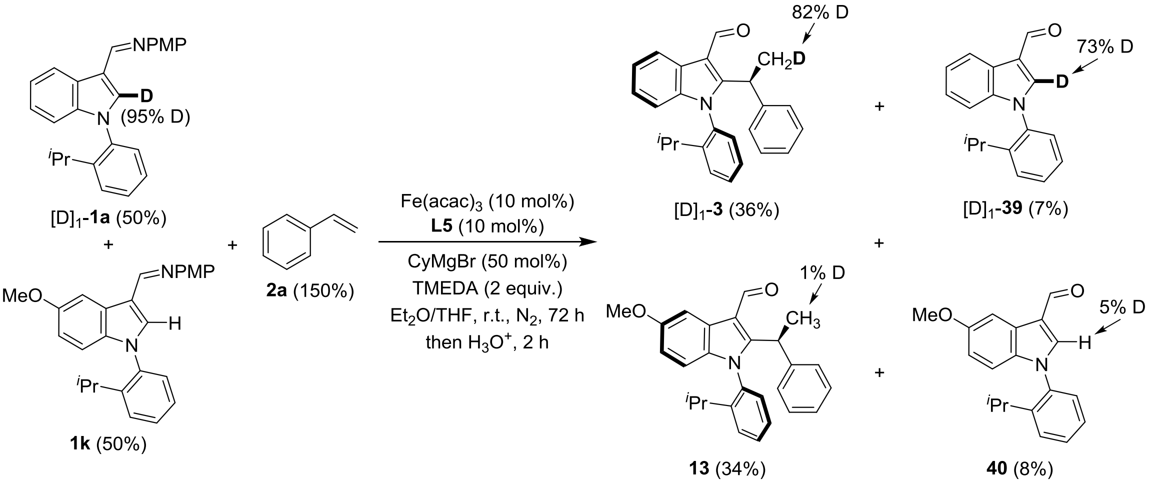

To a flame-dried and N_2-purged Schlenk tube were added indole substrate $[D]_1$-**1a** (0.05 mmol, 18.5 mg), **1k** (0.05 mmol, 19.9 mg), $Fe(acac)_3$ (0.01 mmol, 3.5 mg) and chiral NHC ligand **L5** (0.01 mmol, 7.9 mg). The Schlenk tube was then sealed, purged and backfilled with N_2 three times. Ethyl ether (0.2 mL), TMEDA (0.2 mmol, 30 μL) and styrene **2a** (0.15 mmol, 17 μL) were added *via* syringe. CyMgBr (1 M in THF, 0.05 mmol, 0.05 mL) was then added dropwise and the resulting mixture was stirred at room temperature for 72 hours. Then, the reaction mixture was diluted with tetrahydrofuran (2.0 mL) and quenched with HCl aqueous solution (1 M, 1.0 mL). The resulting mixture was stirred at room temperature for 2 hours. The phases were then separated, the aqueous layer was extracted with ethyl acetate (5.0 mL ×3). The combined organic layer was washed with brine, dried over Na_2SO_4, filtered and concentrated *in vacuo*. The residue was purified by column chromatography on silica gel (*n*-hexane: ethyl acetate = 20:1) to afford products and hydrolyzed starting materials. Deuterium contents were determined by ^{1}H NMR spectroscopic analysis.

9.3 Kinetic isotope effect experiment

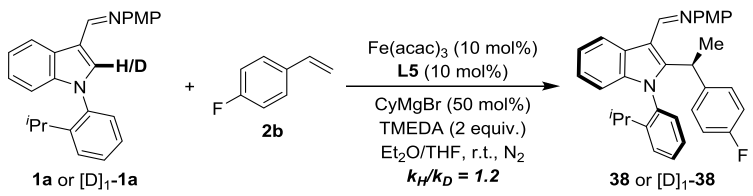

The kinetic isotope effect (KIE) was examined by applying the initial rate method. To a flame-dried and N_2-purged Schlenk tube were added indole substrate **1a** (0.2 mmol, 73.6 mg), $Fe(acac)_3$ (0.02 mmol, 7.0 mg) and chiral NHC ligand **L5** (0.02 mmol, 15.8 mg). The Schlenk tube was then

sealed, purged and backfilled with N_2 three times. Ethyl ether (0.5 mL), TMEDA (0.4 mmol, 60 µL), 4-fluorostyrene **2b** (0.3 mmol, 36 µL) and fluorobenzene (0.2 mmol, 19 µL) were added *via* syringe. CyMgBr (1 M in THF, 0.1 mmol, 0.1 mL) was then added dropwise and the resulting mixture was stirred at room temperature (t = 0 min). Aliquots (50 µL) were removed periodically every 20 min. The conversion was determined by ^{19}F NMR using fluorobenzene as the internal standard.

The same procedure was applied with indole substrate [D]$_1$-**1a** (Note: the last point was not taken into account due to reduction of the reaction rate).

The following results were obtained.

Supplementary Table 3. Kinetic isotope effect

Entry	Time (min)	Yield (%) (with **1a**)	Yield (%) (with [D]$_1$-**1a**)
1	20	1	0
2	40	3	2
3	60	5	3.5
4	80	6.5	5
5	100	9	7
6	120	11	7.5

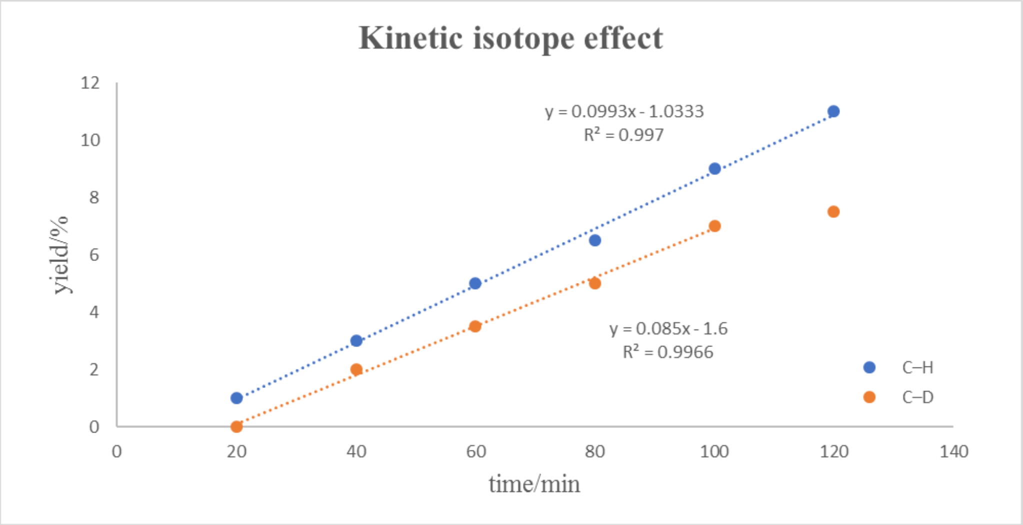

Supplementary Fig. 1 | Kinetic isotope effect

9.4 Nonlinear effect studies

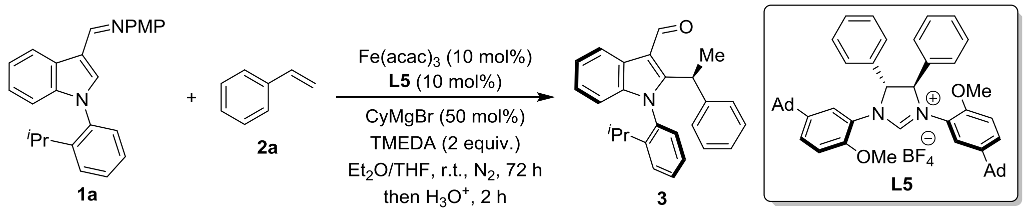

The nonlinear effect study was conducted by building a relationship between the e.e. value of the NHC ligand **L5** and that of the product **3**[5-6] (Supplementary Table 4). The specified e.e. values of NHC ligand **L5** was made by mixing certain amounts of optically pure NHC ligand **L5** with optically pure NHC ligand *ent*-**L5**. Seven reactions containing NHC ligand of racemic, 10%, 20%, 40%, 60%, 80%, and 99% optical purity were run in parallel.

To a flame-dried and N_2-purged Schlenk tube were added indole substrate **1a** (0.1 mmol, 36.8 mg), $Fe(acac)_3$ (0.01 mmol, 3.5 mg) and NHC ligand **L5** with different e.e. values (0.01 mmol, 8.0 mg). The Schlenk tube was then sealed, purged and backfilled with N_2 three times. Ethyl ether (0.2 mL), TMEDA (0.2 mmol, 30 μL) and styrene **2a** (0.15 mmol, 17 μL) were added *via* syringe. CyMgBr (1 M in THF, 0.05 mmol, 0.05 mL) was then added dropwise and the resulting mixture was stirred at room temperature for 72 hours. Then, the reaction mixture was diluted with tetrahydrofuran (2.0 mL) and quenched with HCl aqueous solution (1 M, 1.0 mL). The resulting mixture was stirred at room temperature for 2 hours. The phases were then separated, the aqueous layer was extracted with ethyl acetate (5.0 mL ×3). The combined organic layer was washed with brine, dried over Na_2SO_4, filtered and concentrated *in vacuo*. After purification by column chromatography on silica gel (*n*-hexane: ethyl acetate = 10:1) the product **3** was analyzed by HPLC to determine the enantiomeric excess (CHIRALPAK IC-3, *n*-hexane/*i*-PrOH = 95/5, flow rate: 1.0 mL/min, T = 20 °C, 250 nm, t_R (major) = 22.18 min, t_R (minor) = 24.78 min). A graph of e.e. of product vs. e.e. of NHC ligand was then plotted (Supplementary Fig. 2).

Supplementary Table 4. Nonlinear effect studies

Entry	e.e. of NHC ligand **L5** (%)	Yield (%)	d.r.	e.e. of product **3** (%)
1	0	86	>95:5	0
2	10	76	>95:5	10
3	20	68	>95:5	16
4	40	78	>95:5	34
5	60	76	>95:5	50
6	80	87	>95:5	73
7	99	90	>95:5	97

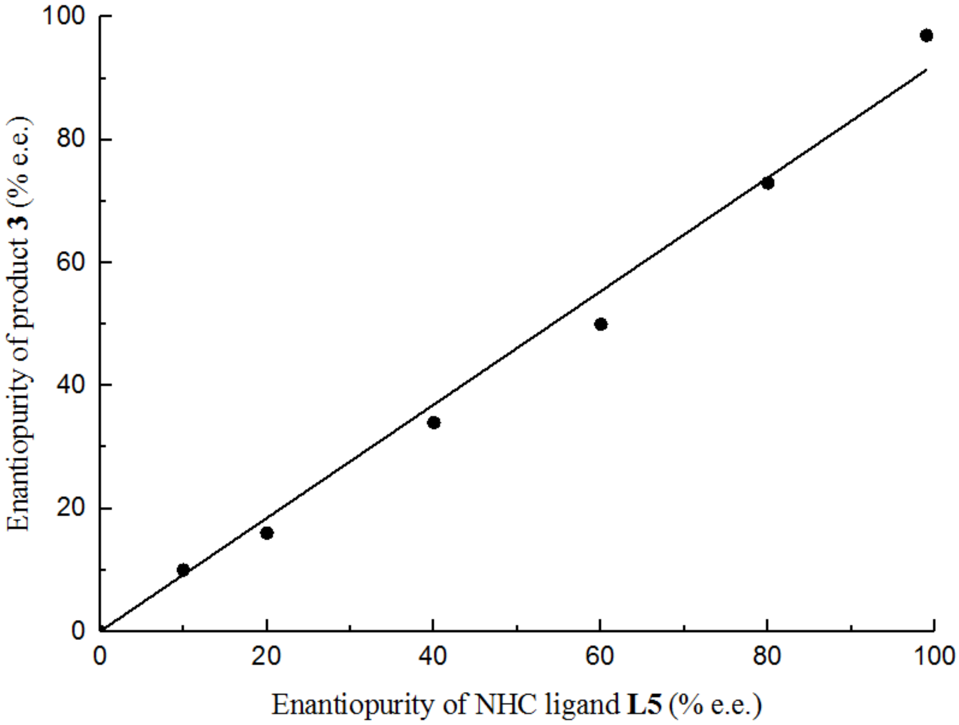

Supplementary Fig. 2 | Nonlinear effect studies

9.5 Synthesis of complex Fe(IMes)(η^2-styrene)$_2$ (41)

Fe(IMes)(η^2-styrene)$_2$ (**41**) was synthesized according to a previously reported procedure[7]. To a colorless solution of IMes (1.0 mmol, 304.4 mg) in THF (5.0 mL) was added $FeCl_2$ (1.0 mmol, 125.9 mg) at room temperature. The reaction mixture was stirring at room temperature for 4 hours, and then styrene (2.5 mmol, 286 μL) and KC_8 (2.0 mmol, 269.9 mg) were added subsequently. After stirring at room temperature for 16 hours, the mixture was filtered to give a yellow green solution. The solution was subjected to vacuum to remove the volatiles, and the resultant green residue was washed with *n*-hexane (10 mL) and dried under vacuum, which gave **41** as a deep green solid (273.1 mg, 48% yield).

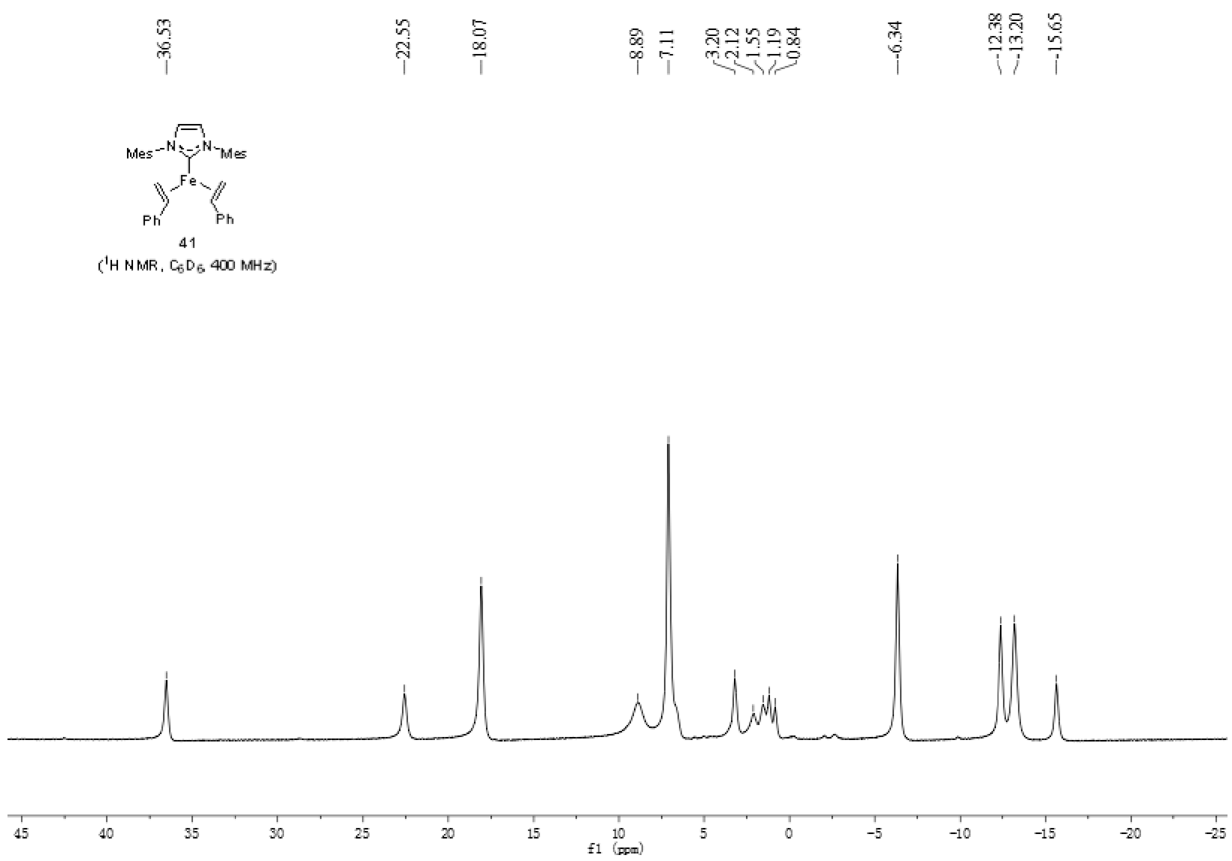

Supplementary Fig. 3 | ^{1}H NMR spectrum of Fe(IMes)(η^2-styrene)$_2$ (**41**).

9.6 Monitoring of the reaction between 1a and 2a catalyzed by Fe(acac)$_3$ in presence of IMes•HCl and CyMgBr with *in situ* ^{1}H NMR spectroscopy

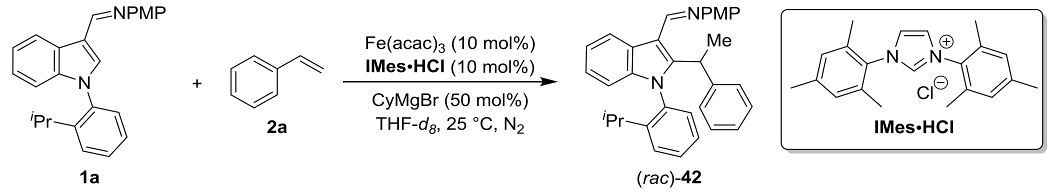

In a nitrogen filled glove box, a screw-top NMR tube was loaded with indole substrate **1a** (0.1 mmol, 36.8 mg), 1,3,5-trimethoxybenzene (0.1 mmol, 16.8 mg), styrene **2a** (0.15 mmol, 17 μL), IMes•HCl (100 mM fine suspension in THF-d_8, 0.01 mmol, 100 μL), Fe(acac)$_3$ (100 mM solution in THF-d_8, 0.01 mmol, 100 μL) and THF-d_8 (150 μL). Subsequently, CyMgBr (1.07 mM solution in THF, 0.050 mmol, 46.9 μL) was added and the NMR tube was placed in a Dewar filled with liquid nitrogen. It was then transferred to the NMR spectrometer where it was thawed and vigorously shaken before being inserted in the spectrometer at 25 °C where ^{1}H NMR spectra were collected every 5 minutes with a relaxation delay of 10 seconds (Supplementary Fig. 4-7).

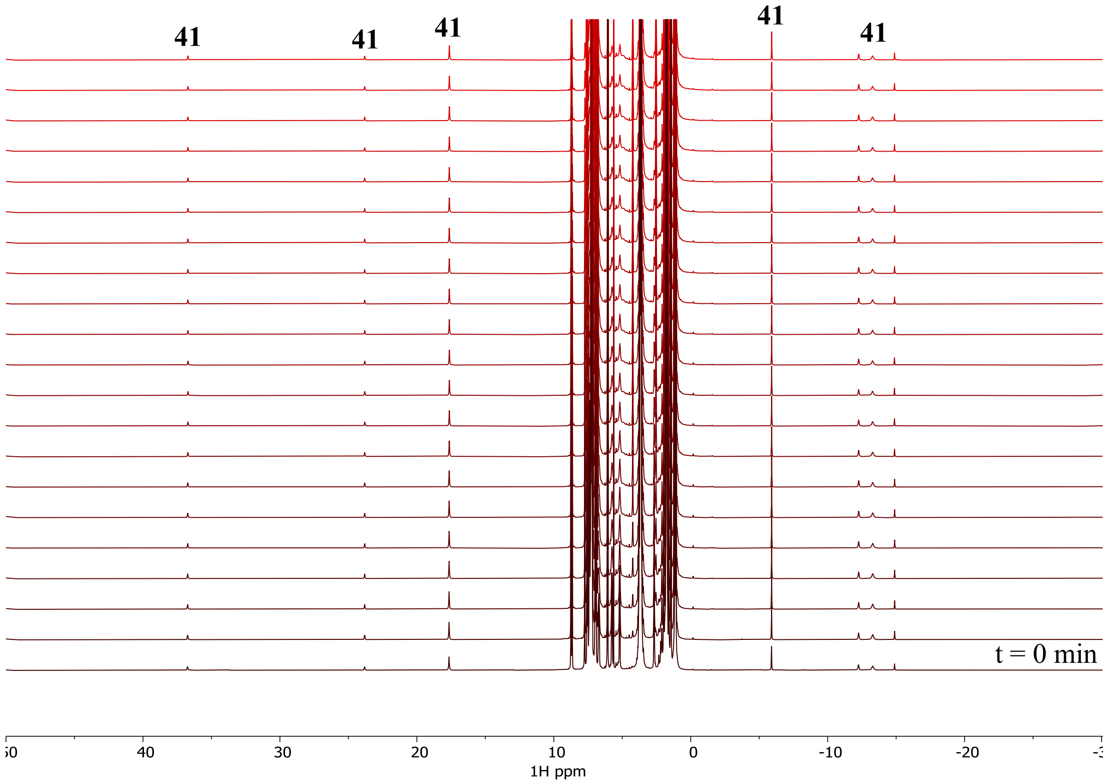

Supplementary Fig. 4 | ^{1}H NMR (400 MHz, THF-d_8, 25 °C) spectra collected with a time interval of 5 min (only 21 spectra are presented here for clarity) in the reaction of **1a** (250 mM) and **2a** (395 mM) catalyzed by a mixture of $Fe(acac)_3$ (25 mM) and IMes•HCl (25 mM) after being activated with CyMgBr (125 mM) in THF-d_8 (400 µL).

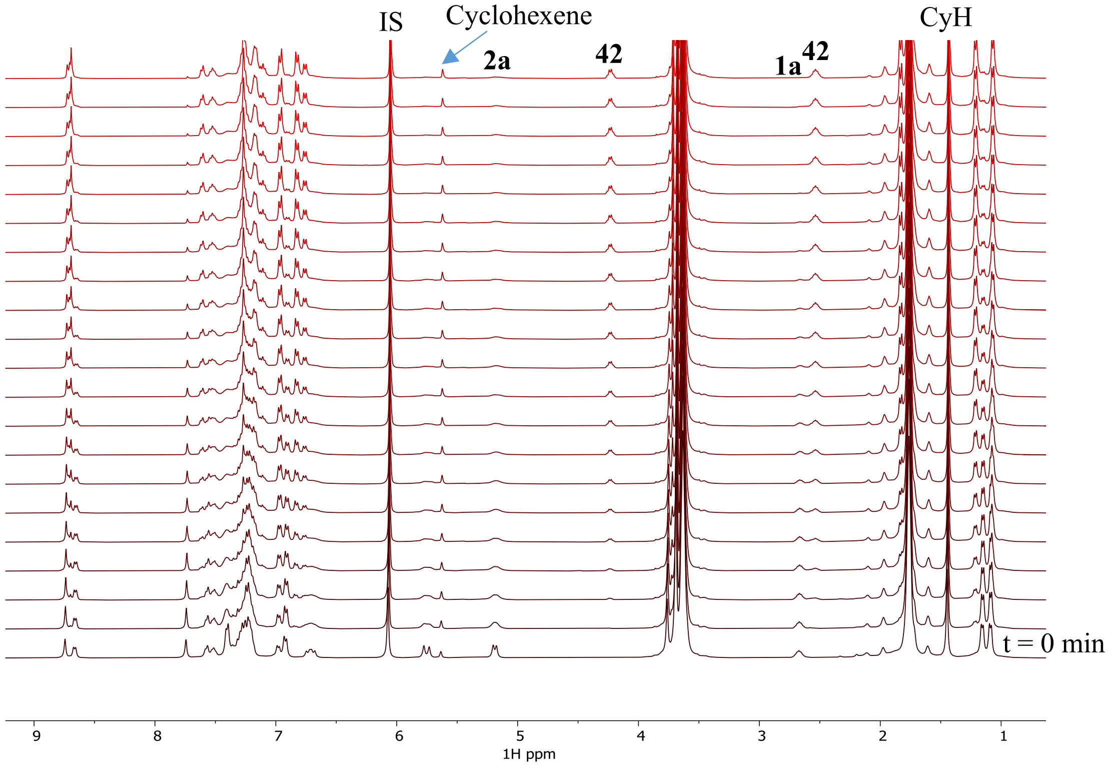

Supplementary Fig. 5 | Expansion of **Supplementary Fig. 4**.

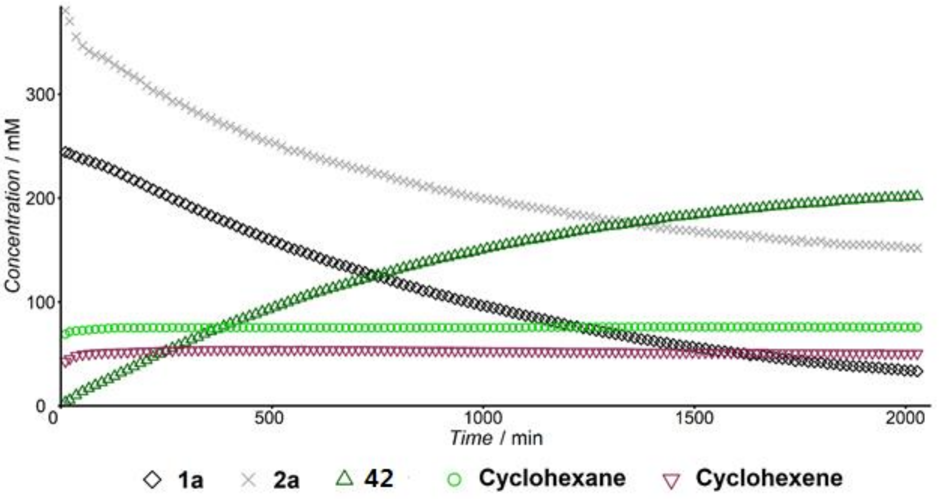

Supplementary Fig. 6 | Reaction between **1a** (250 mM) and **2a** (370 mM) catalyzed by a mixture of Fe(acac)$_3$ (25 mM) and IMes•HCl (25 mM) after being activated with CyMgBr (125 mM) in THF-d_8 (400 μL) at 25 °C.

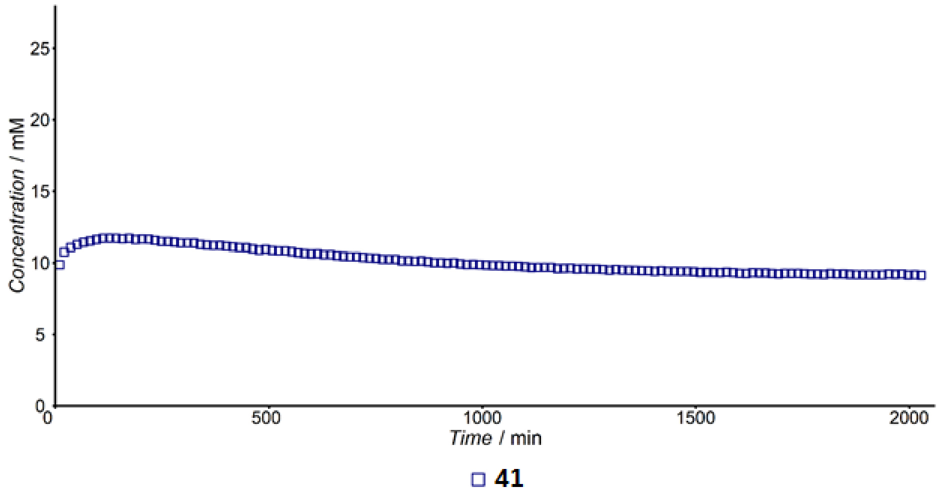

Supplementary Fig. 7 | Reaction between **1a** (250 mM) and **2a** (370 mM) catalyzed by a mixture of Fe(acac)$_3$ (25 mM) and IMes•HCl (25 mM) after being activated with CyMgBr (125 mM) in THF-d_8 (400 μL) at 25 °C.

9.7 Monitoring of the reaction between 1a and 2a catalyzed by Fe(IMes)(η^2-styrene)$_2$ with *in situ* ^{1}H NMR spectroscopy

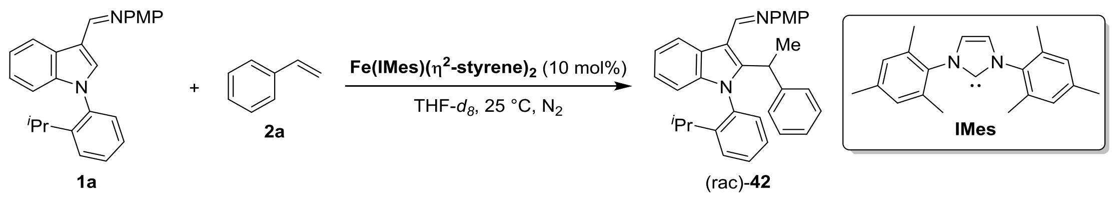

In a nitrogen filled glove box, a screw-top NMR tube was loaded with indole substrate **1a** (0.125 mmol, 46.1 mg), 1,3,5-trimethoxybenzene (0.125 mmol, 21.0 mg), styrene **2a** (0.188 mmol, 22.5 μL) and THF-d_8 (375 μL). Subsequently, Fe(IMes)(η^2-styrene)$_2$ (**41**) (100 mM solution in THF-d_8, 0.0125 mmol, 125 μL,) was added and the NMR tube was placed in a Dewar filled with liquid nitrogen. It was then transferred to the NMR spectrometer where it was thawed and vigorously

shaken before being inserted in the spectrometer at 25 °C. Subsequently, ^{1}H NMR spectra were collected every 5 minutes with a relaxation delay of 10 seconds (Supplementary Fig. 8-11).

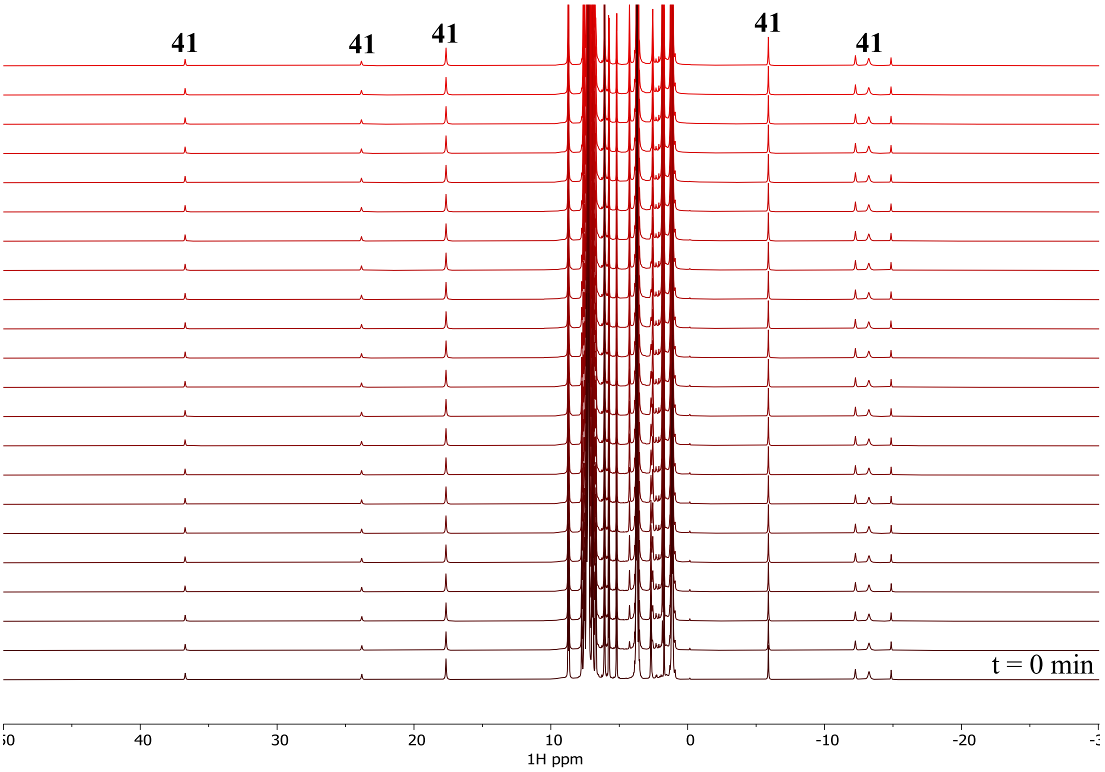

Supplementary Fig. 8 | ^{1}H NMR (400 MHz, THF-d_8, 25 °C) spectra collected with a time interval of 5 min (only 21 spectra are presented here for clarity) in the reaction between **1a** (250 mM) and **2a** (370 mM) catalyzed by Fe(IMes)(η^2-styrene)$_2$ (**41**) (25 mM) in THF-d_8 (500 μL).

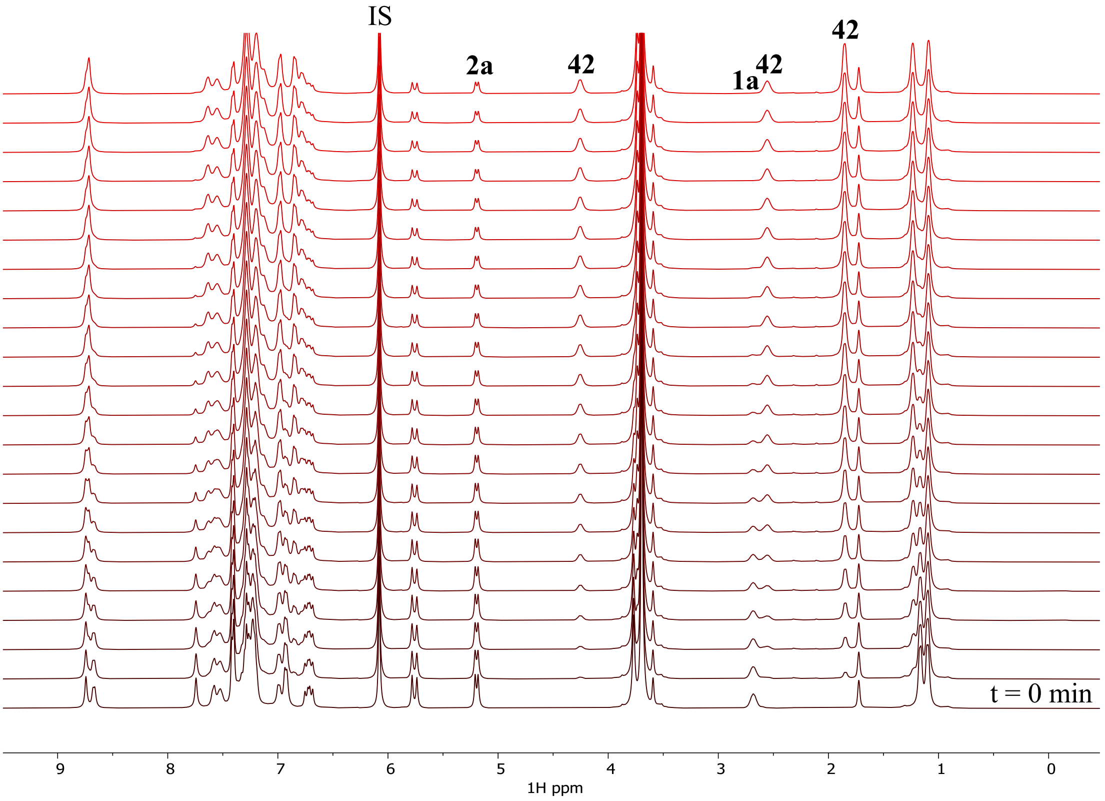

Supplementary Fig. 9 | Expansion of **Supplementary Fig. 8**.

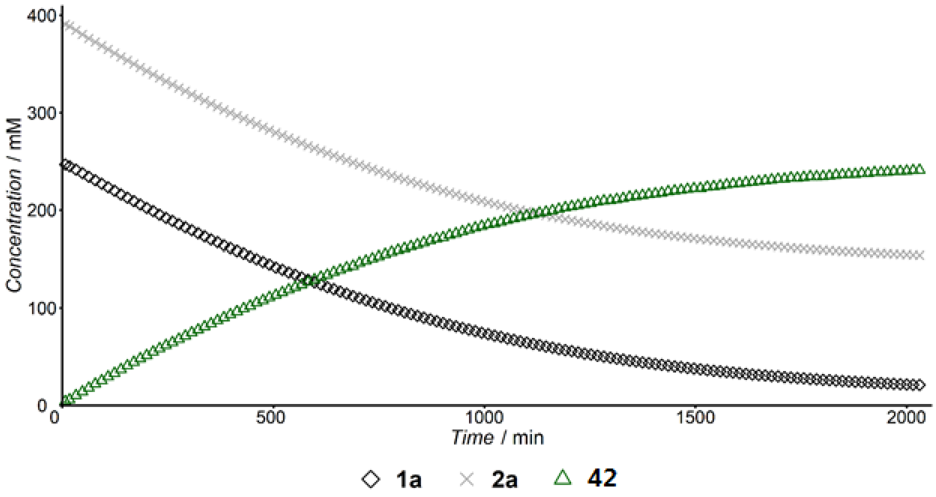

Supplementary Fig. 10 | Reaction between **1a** (250 mM) and **2a** (370 mM) catalyzed by Fe(IMes)(η^2-styrene)$_2$ (**41**) (25 mM) in THF-d_8 (500 µL) at 25 °C.

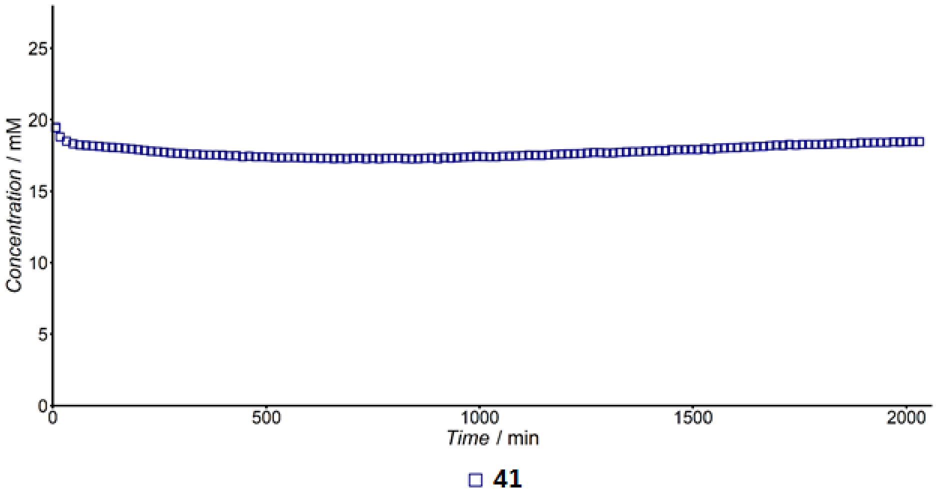

Supplementary Fig. 11 | Reaction between **1a** (250 mM) and **2a** (370 mM) catalyzed by Fe(IMes)(η^2-styrene)$_2$ (**41**) (25 mM) in THF-d_8 (500 µL) at 25 °C.

9.8 Comparison of the catalytic performance between the *in situ* generated system and [Fe(IMes)(η^2-styrene)$_2$]

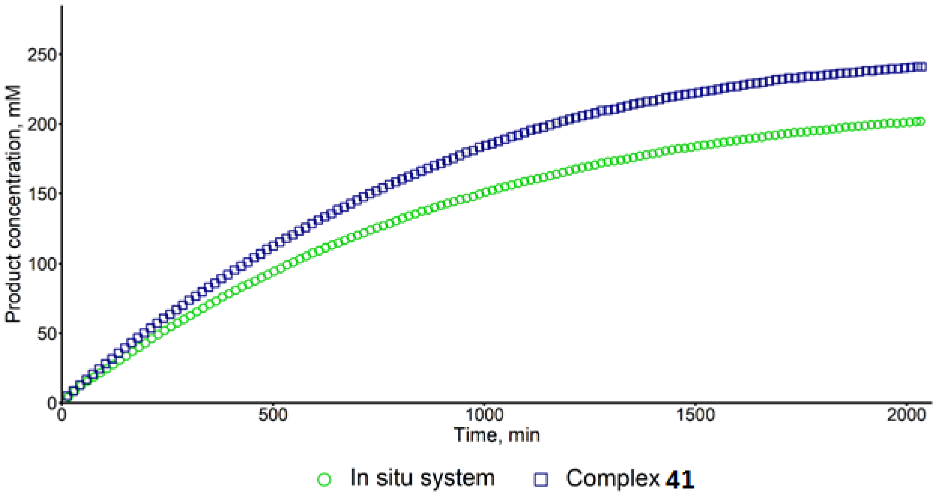

Supplementary Fig. 12 | Reaction between indole substrate **1a** and styrene **2a** catalyzed by Fe(acac)$_3$/IMes•HCl/CyMgBr (green circle) or by the well-defined iron/carbene complex Fe(IMes)(η^2-styrene)$_2$ (**41**) (navy square) at 10 mol% loading in iron and 25 °C.

9.9 Preparation of [Fe(Cy)(η^2-styrene)$_3$][MgCl(THF)$_5$] (A)

Fe(acac)$_3$ (142.0 mg, 0.4 mmol), TMEDA (120 µL, 0.8 mmol), and THF (1.0 mL) was added to a 20 mL scintillation vial and stirred for 15 minutes at room temperature and then transferred to a pre-cooled aluminum pi-block at -25 °C for 30 minutes. Next, styrene (230 µL, 2.0 mmol) was added to the scintillation chilled vial and stirred for 2 minutes followed by addition of CyMgCl (1.54 mL, 1.3 M in THF/toluene). Afterwards, 5 mL of chilled THF was added to the vial and stirred for 2 minutes followed by addition of 5 mL of chilled (-25 °C) hexane while stirring. After stirring for 2 minutes, the solution was allowed to settle for 5 minutes at -25 °C. The vial of solution was then filtered through a 1 cm Celite pad into clean 20 mL scintillation vials and stored in a -30 °C freezer. Dark red/maroon crystals.

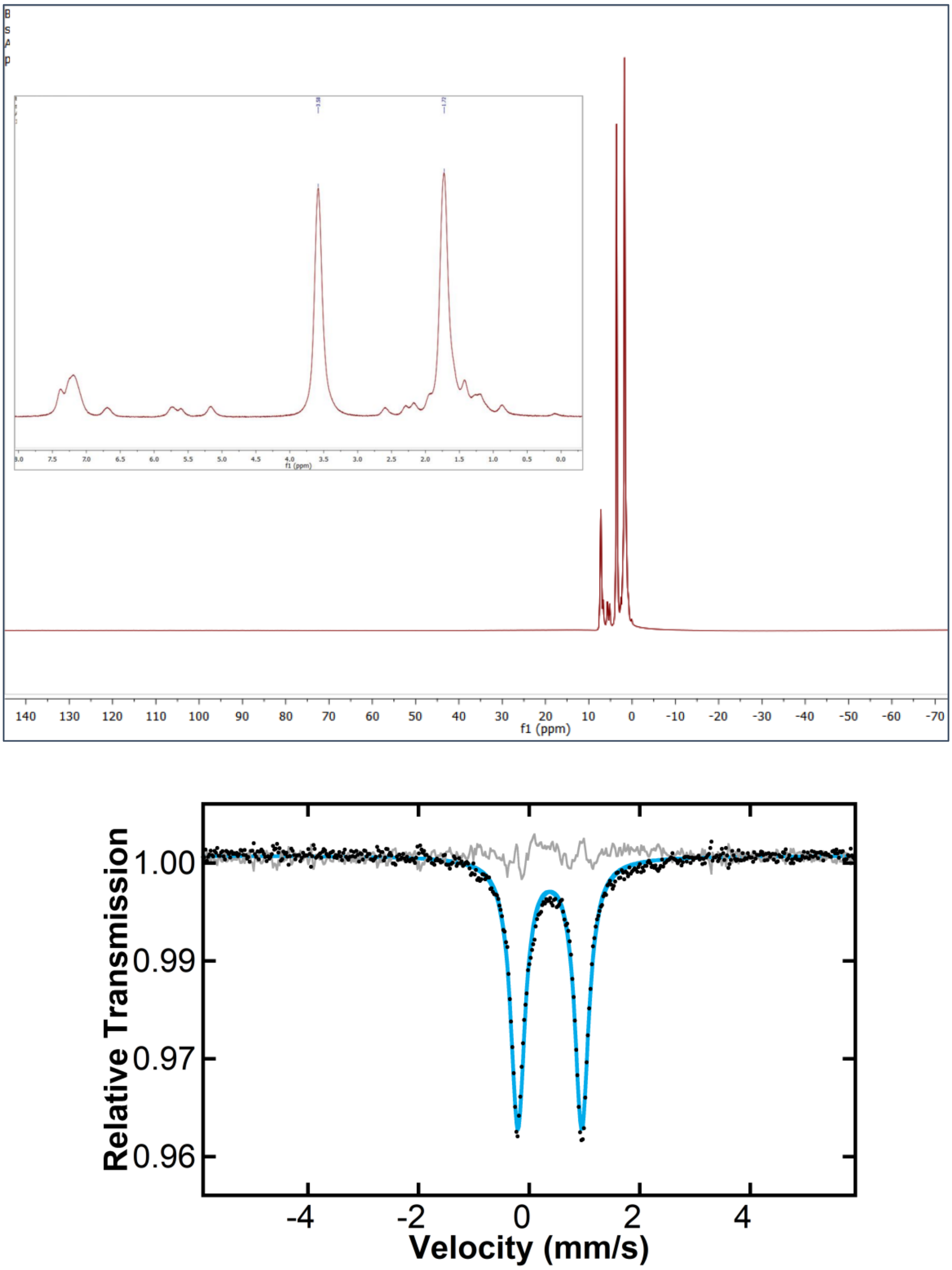

Supplementary Fig. 13 | ^{1}H NMR spectrum of [Fe(Cy)(η^2-styrene)$_3$][MgCl(THF)$_5$] (**A**) in THF-d_8. Evans method measurements were not consistent, attributed to the high instability of the complex for sample weighing and preparation (top). 80 K ^{57}Fe Mössbauer spectra of **A** with δ = 0.37 mm/s and $|\Delta E_Q|$ = 1.16 mm/s (100%) with the residual plot (bottom). Note: The residual shown in grey was calculated using the formula: $N_{calculated}$ - $N_{experimental}$ / $(N_{experimental})^{1/2}$ where $N_{calculated}$ = the number of counts of the calculated spectra and $N_{experimental}$ = the number of counts of the experimental spectra. The result was then converted to relative transmission for comparison.

9.10 Preparation of Fe(SIMes)(η^2-styrene)$_2$ (B*)

^{57}Fe(acac)$_3$ (9.3 mg, 0.026 mmol), Fe(acac)$_3$ (19.0 mg, 0.054 mmol), and THF (0.5 mL) was added to a 20 mL scintillation vial and stirred for 15 minutes at room temperature. In a separate 20 mL scintillation vial, SIMes•HCl (27.4 mg, 0.08 mmol) was dissolved in 0.5 mL of THF followed by addition of Phenethylmagnesiumchloride (400 µL, 1 M in THF). Both Fe(acac)$_3$ and SIMes solution were then transferred to a pre-cooled aluminum pi-block at -20 °C for 30 minutes. Next, the solution of SIMes was added dropwise to the solution of Fe(acac)$_3$ and stirred for 10 minutes at -20 °C. Afterwards, the vial was warmed up to room temperature and stirred for 30 minutes and then transferred to an oil recirculating bath cooled aluminum pie-block at -20 °C for 30 minutes. To the scintillation vial, 8 mL of chilled (-20 °C) pentane was added while stirring. After stirring for 5 minutes, the solution was allowed to settle for 5 minutes at -20 °C. The vial of solution was then filtered through a 1 cm Celite pad into clean 20 mL scintillation vials and stored in a -30 °C freezer. Dark green crystals were observed in each vial after 2-4 days. Evans method NMR was performed in THF-d_8, and the magnetic moment was determined to be 3.8(2) B.M, consistent with a high-spin (S = 1) ground state.

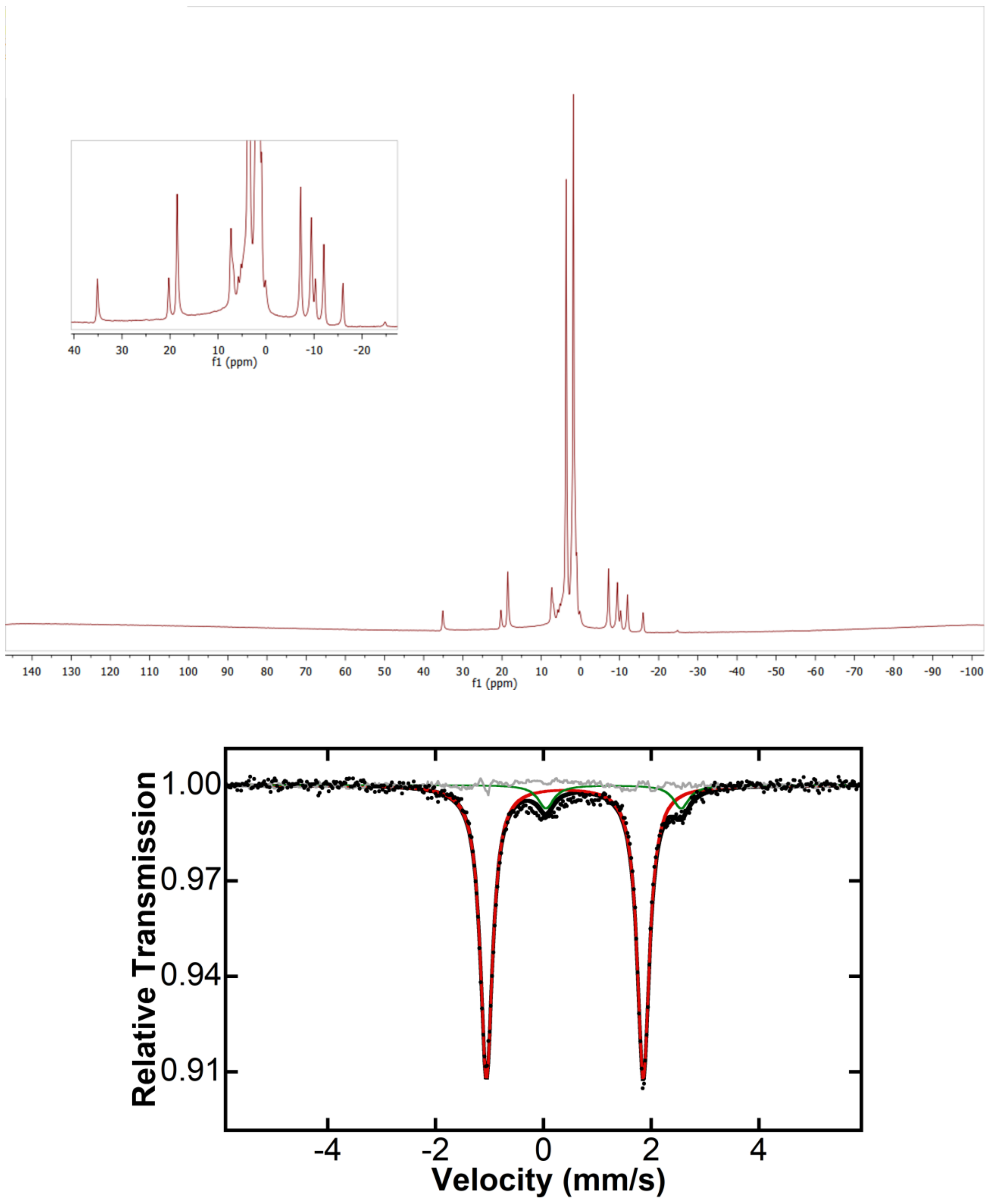

Supplementary Fig. 14 | ^{1}H NMR spectrum of Fe(SIMes)(η^2-styrene)$_2$ (**B***) in THF-d_8 (top). 80 K ^{57}Fe Mössbauer spectra of **B*** with δ = 0.39 mm/s and $|\Delta E_Q|$ = 2.92 mm/s (93%) with the residual plot shown in grey (bottom).

9.11 Stoichiometric reaction of A with L5

A solution of crystals of **A** (11.3 mg) and **L5** (17.0 mg) was prepared in 0.4 mL THF at -25 °C and stirred for 15 minutes and warmed up to room temperature for 10 minutes and then transferred to 45 °C. The solution was then used for sample preparation by freeze-trapped method in liquid nitrogen at 30 seconds and then 2 minutes for Mössbauer analysis (Supplementary Fig. 15). Note: Reaction of **A** with achiral ligand SIMes•HCl also resulted in generation of species **B*** up to 50% after 10 minutes at room temperature (Supplementary Fig. 16).

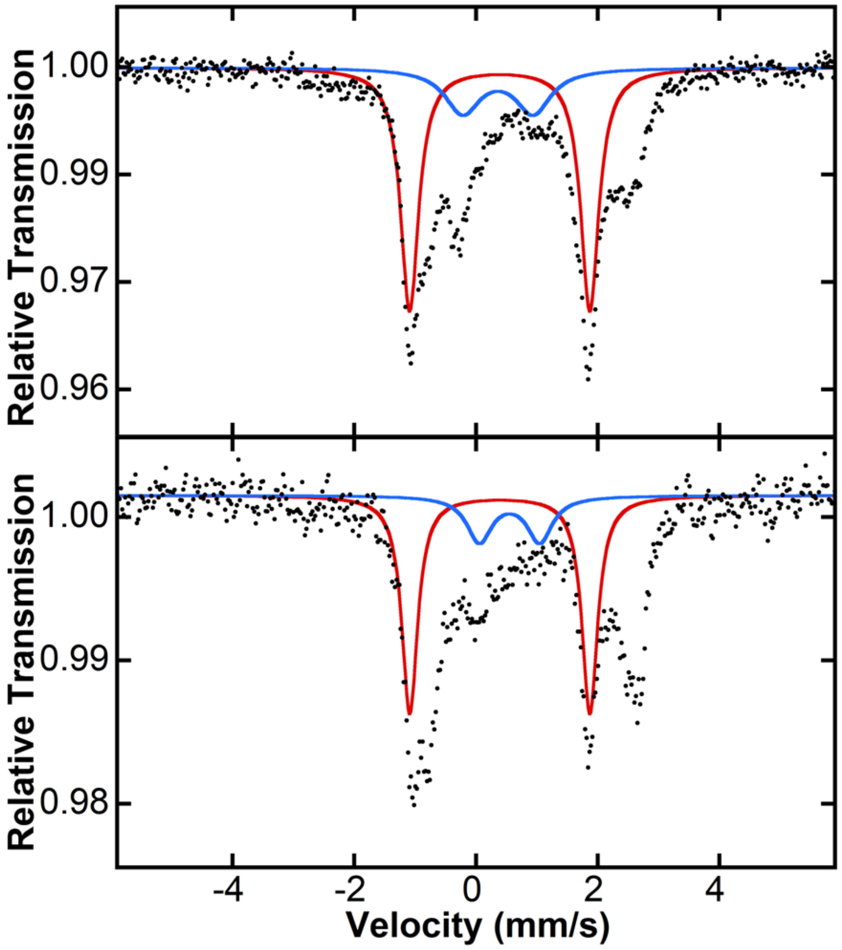

Supplementary Fig. 15 | Mössbauer analysis of the reaction of Fe(Cy)(η^2-styrene)$_3$ (**A**) with a solution of **L5** (1.5 equiv) for 30 seconds (top) and 2 minutes (bottom). The red species with δ = 0.39 mm/s and $|E_Q|$ = 2.84 mm/s is the dominant species (44% in top, 37% in bottom) likely corresponding to **B**. While a small amount of unreacted **A** (blue species, 16% of total iron) species appears to be present, it was not possible with the current data and isolated species parameters to fit any further components with confidence. Therefore, no residual plot is given. The presence of a much larger amount of **B** during catalysis may indicate that the presence of excess styrene favors the formation of **B** in solution and limits its decomposition at 45 °C.

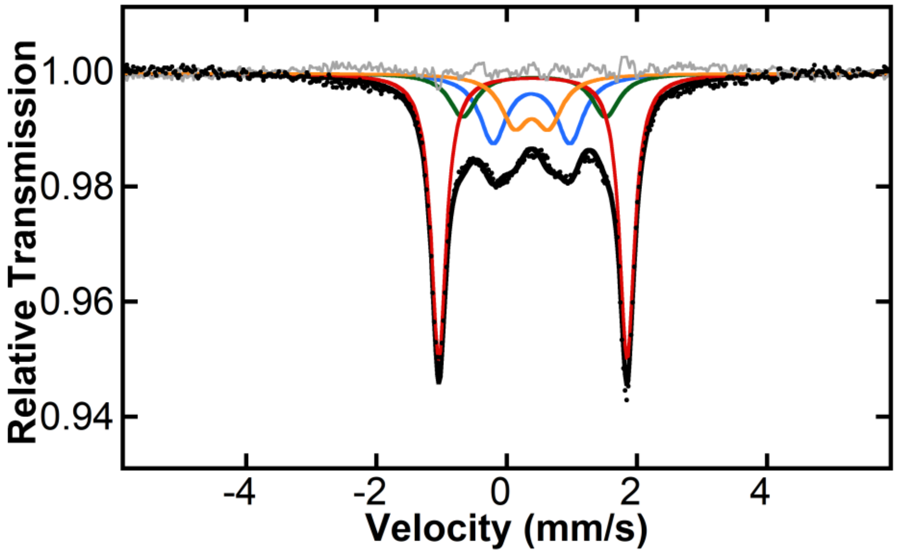

Supplementary Fig. 16 | Mössbauer analysis of the reaction of Fe(Cy)(η^2-styrene)$_3$ (**A**) with a solution of SIMes•HCl (1.5 equiv.). The major red component has parameters corresponding to **B*** and the minor blue component has parameters corresponding to unreacted **A** (21 % of total iron). Unfortunately, the remaining species could not be assigned and likely result from the thermal instability of **A**. The residual plot is shown in grey.

9.12 Catalytic C−H alkylation reaction

A solution was prepared with ^{57}Fe(acac)$_3$ (2.2 mg, 0.006 mmol), Fe(acac)$_3$ (8.5 mg, 0.024 mmol), **L5** (47.6 mg, 0.06 mmol), **1a** (106.5 mg, 0.3 mmol), TMEDA (71.0 mg, 0.6 mmol), styrene (48.1 mg, 0.45 mmol), CyMgBr (330 µL, 1M in THF) and THF (0.6 mL) at room temperature followed by transfer to a pre-heated aluminum pie-block and stirred at 45 °C. Samples were freeze-trapped in liquid nitrogen at 120 minutes and 360 minutes for Mössbauer and EPR analysis.

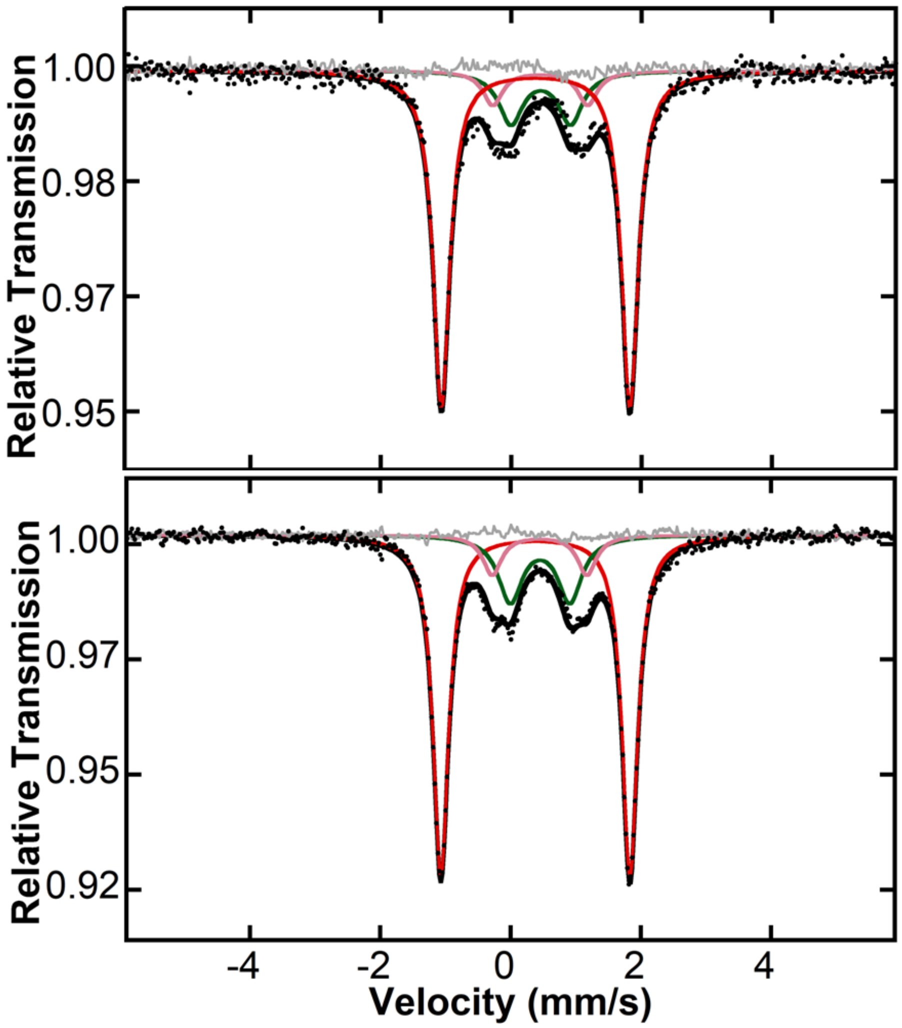

Supplementary Fig. 17 | Catalytic reaction between **1a** and styrene freeze-trapped for 80 K ^{57}Fe Mössbauer spectroscopy at 120 minutes (top) and 360 minutes (below), showing one dominant species **B** with parameters δ = 0.39 mm/s and $|\Delta E_Q|$ = 2.90 mm/s (red component). The residual plots are shown in grey. Note: Less than 0.5% species observed to be EPR active.

9.13 Stoichiometric reaction of B* with 1a

In a 20 mL scintillation vial $Fe(acac)_3$ (35.3 mg, 0.1 mmol), SIMes•HCl (34.3 mg, 0.1 mmol), and THF (2.0 mL) were added and stirred at room temperature for 10 minutes. The vial was then cooled to -20 °C for 30 minutes. Then Phenethylmagnesiumchloride (0.6 mL, 1.0 M, 0.6 mmol) was quickly added to the vial followed by stirring at -20 °C for 15 minutes. The vial was then stirred at room temperature for 15 minutes. For the reaction with **1a**, the solution was added to a new vial containing **1a** (35.7 mg, 0.11 mmol) at room temperature, and allowed to stir for 1 minute prior to placing on an aluminum pie-block preheated to 45 °C and stirred for 30 minutes. The sample was then transferred to clean 20 mL scintillation vials with 1 mL deionized H_2O and stirred for 5 minutes at room temperature. Then 3 mL of dichloromethane was added followed by 2 mL HCl (6 M). The solution was then stirred at 45 °C for 4 hours. Then the solution was allowed to cool to room temperature. The organic phase was extracted in dichloromethane. The extracts were then dried under vacuum followed by addition of the internal standard (1,3,5-trimethoxybenzene). The solids were dissolved in $CDCl_3$ and the solution was filtered through Celite into an NMR tube. ^1H NMR analysis of generated product using 1,2,3-trimethoxybenzene as internal standard revealed 58% product generation with respect to the generated **B*** species.

10. DFT calculations

Computational Methods

All DFT calculations were carried out with Gaussian 16 program[8]. The geometry optimizations were conducted using B3LYP functional[9,10] including Grimme's dispersion corrections[11] with Becke-Johnson damping, Def2-SVP basis set[12,13] for iron and other atoms. To confirm whether each optimized stationary point is an energy minimum or a transition state as well as evaluate the zero-point vibrational energy and thermal corrections at 298 K, the vibrational frequencies were computed at the same level of theory as for the geometry optimizations. On the basis of the gas-phase optimized structures, the single-point energies and solvent effects were evaluated with the B3LYP-D3(BJ) functional Def2-TZVPP basis set[12,13] for iron and other atoms. The solvation energies were calculated using the self-consistent reaction field with the SMD implicit solvent model[14]. The 3D diagrams of computed species were generated using CYLView[15] or VMD[16].

Energy decomposition analysis was performed based on the classical molecular force field[17] (EDA-FF) using Multiwfn software[18]. The universal force field (UFF) was used for iron and AMBER force field for other atoms. Atomic charges were generated by Merz-Kollman (MK) method.

Calculations of Mössbauer parameters were performed with ORCA package[19], using optimized structure for **B** (from the method above) and those for **A** and **B*** using the B3LYP functional and def2-TZVP[12] basis set for all atoms. Calculations were performed using BP86 functional[20,21] with core polarized CP(PPP)[22,23] basis set for iron atom and def2-TZVP basis set for all other atoms. Isomer shifts were calculated from electron densities at the iron nucleus using a previously reported procedure[24]. The observed deviations in isomer shift values between experiment and theory were considering the errors commonly observed in calculating isomer shifts for organoiron species[25].

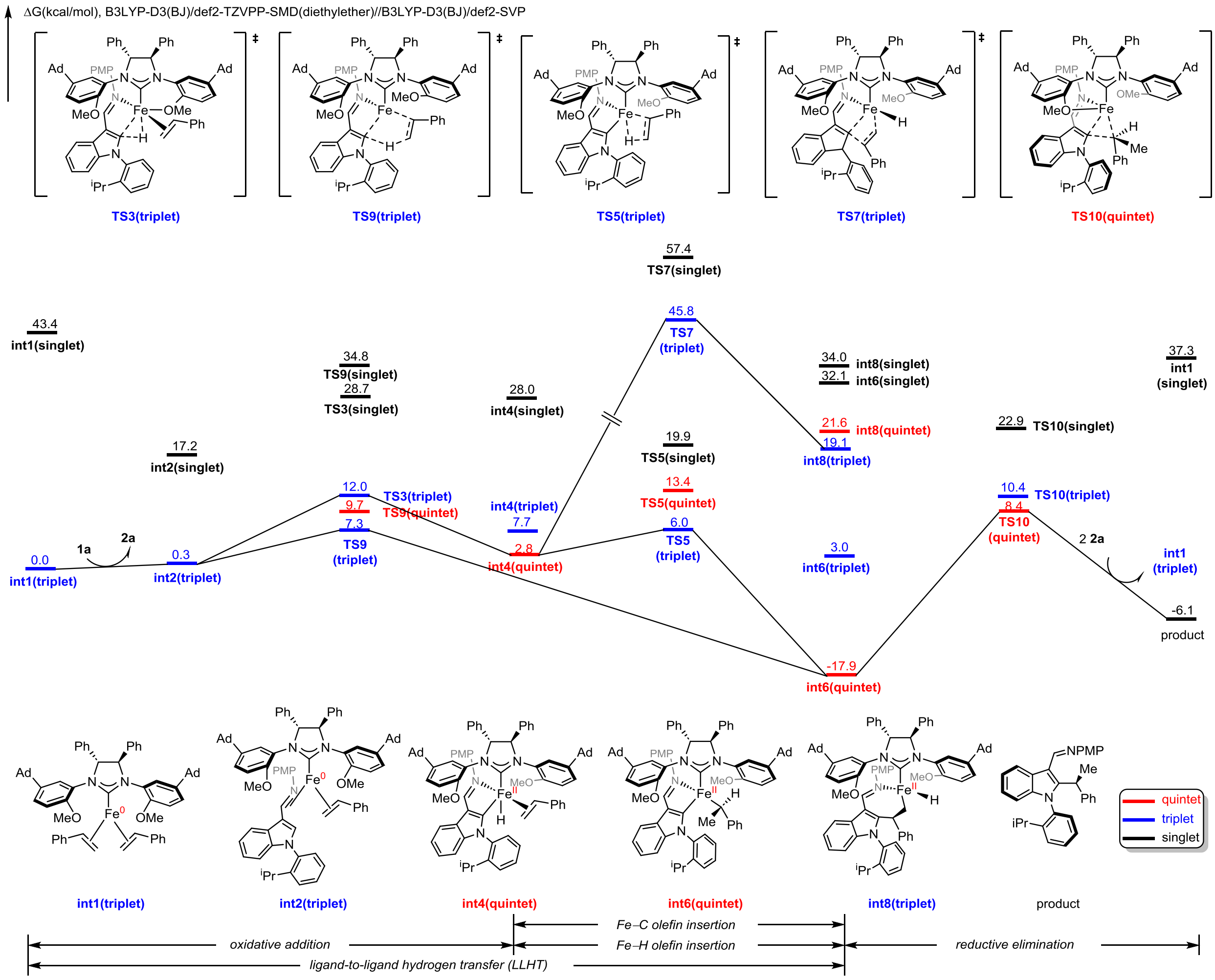

Supplementary Fig. 18 | DFT-computed free energy profile of the competing reaction pathways for iron-catalyzed asymmetric C−H alkenylation for major product including all possible spin states.

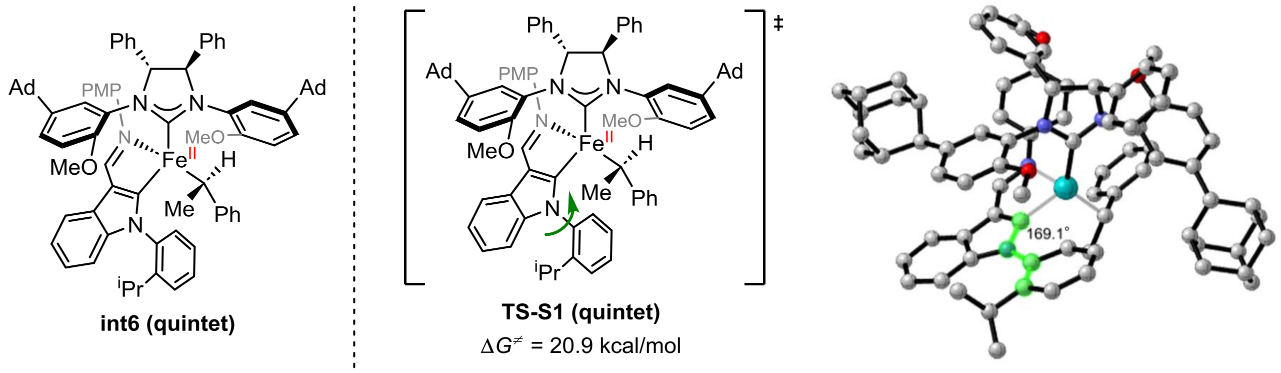

Supplementary Fig. 19 | Racemization of axial chirality in alkyl iron(II) intermediate **int6**.

a) Energy decomposition analysis

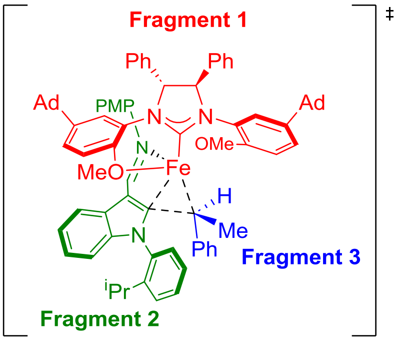

TS10 (quintet)

	Electrostatic (kcal/mol)	Repulsion (kcal/mol)	Dispersion (kcal/mol)
**Frag1** and **Frag2**	-177.1	52.5	-43.3
**Frag1** and **Frag3**	-128.9	6.2	-9.1
**Frag2** and **Frag3**	84.7	111.2	-32.0

b) Atom contribution to electrostatic interaction *c) Atom contribution to dispersion interaction*

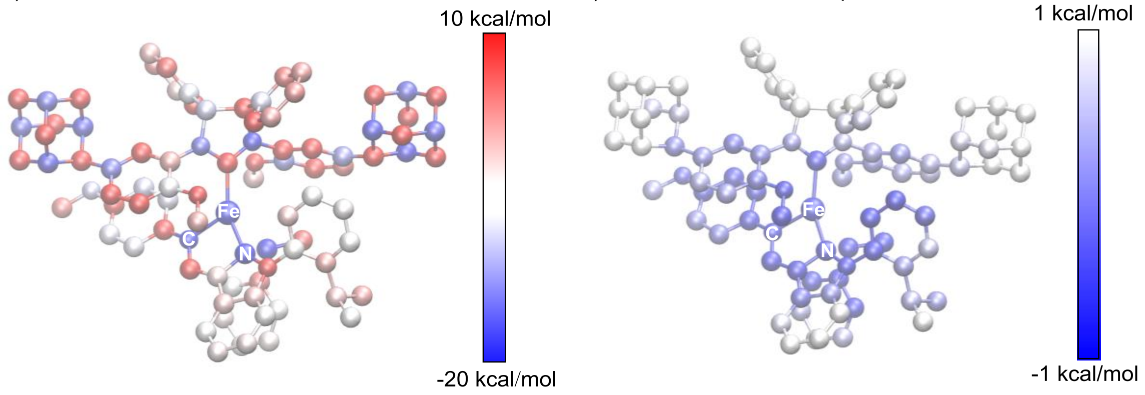

Supplementary Fig. 20 | Energy decomposition analysis of the reductive elimination transition state **TS10 (*R*$_a$, *S*)**. **a**, Interaction energy components between fragments in the **TS10 (*R*$_a$, *S*)**. **b**, Atom contribution map to the electrostatic interaction component in **TS10 (*R*$_a$, *S*)**. **c**, Atom contribution map to the dispersion interaction in **TS10 (*R*$_a$, *S*)**. Non-relevant hydrogens were omitted for clarity.

To access the nature as well as to quantify the noncovalent interactions in the quintet **TS10 (*R*$_a$, *S*)**, we have performed energy decomposition analysis (EDA). The reductive elimination transition state was divided into three fragments (Supplementary Fig. 20a), namely iron catalyst part (fragment 1), indole part (fragment 2) and alkyl part (fragment 3). The EDA results show that electrostatics is the major component to the noncovalent interactions followed by dispersion forces. The binding of fragment 1 and fragment 2 is the strongest among other combinations, which is also reflected in both atom-level electrostatic and dispersion interaction maps. The atoms that contribute the most to the electrostatic interaction are the iron from fragment 1 as well as the C and N connected to the metal center from fragment 2 (Supplementary Fig. 20b). The dispersion interaction is also present in larger extent between fragment 1 and fragment 2 (Supplementary Fig. 20c). The electrostatic in combination with dispersion force strongly contributes for the stabilization of the reductive elimination transition state **TS10 (*R*$_a$, *S*).**

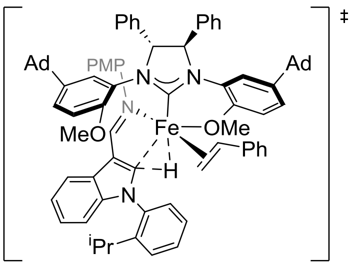

TS9(triplet)

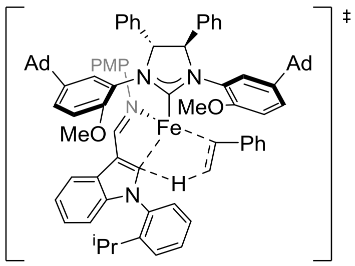

TS3(triplet)

Methods	ΔΔG≠ (kcal/mol)	ΔΔG≠ (kcal/mol)
A	0.0	4.7
B	0.0	5.9
C	0.0	7.5
D	0.0	7.7

Method A: B3LYP-D3(BJ)/def2-TZVPP-SMD(diethylether)//B3LYP-D3(BJ)/def2-SVP
Method B: TPSSh-D3(BJ)/def2-TZVPP-SMD(diethylether)//B3LYP-D3(BJ)/def2-SVP
Method C: ω-B97XD/def2-TZVPP-SMD(diethylether)//B3LYP-D3(BJ)/def2-SVP
Method D: PBE0-D3(BJ)/def2-TZVPP-SMD(diethylether)//B3LYP-D3(BJ)/def2-SVP

Supplementary Fig. 21 | Relative free energies of the competing ligand-to-ligand hydrogen transfer transition state **TS9** and oxidative addition transition state **TS3** using various functionals.

Table of Energies

Supplementary Table 5. Zero-point correction (*ZPE*), thermal correction to enthalpy (*TCH*), thermal correction to Gibbs free energy (*TCG*), electronic energies (*E*), enthalpies (*H*) and Gibbs free energy (*G*) (in Hartree) of the structures calculated at the B3LYP-D3(BJ)/def2-TZVPP-SMD(diethylether)//B3LYP-D3(BJ)/def2-SVP level of theory.

Structures	*ZPE*	*TCH*	*TCG*	*E*	*H*	*G*	Imaginary Frequency
**int1(singlet)**	1.206837	1.266628	1.110598	− 4044.157692	− 4042.891064	− 4043.047094	
**int1(triplet)**	1.203335	1.263333	1.106500	− 4044.222814	− 4042.959481	−4043.116314	
**int2(singlet)**	1.502260	1.579681	1.385370	− 4886.808312	−4885.228631	−4885.422942	
**int2(triplet)**	1.499283	1.577703	1.378677	− 4886.828609	−4885.250906	−4885.449932	
**TS3(singlet)**	1.500858	1.577729	1.386060	− 4886.79062	−4885.212891	−4885.40456	331.63*i*
**TS3(triplet)**	1.495263	1.573445	1.376739	− 4886.807917	−4885.234472	−4885.431178	860.39*i*
**int4(singlet)**	1.500140	1.577469	1.385170	− 4886.790861	−4885.213392	−4885.405691	
**int4(triplet)**	1.497904	1.575938	1.379914	− 4886.817949	−4885.242011	−4885.438035	

**int4(quintet)**	1.495436	1.573745	1.378825	−4886.824752	—4885.251007	—4885.445927	
**TS5(singlet)**	1.499975	1.577394	1.384198	−4886.802906	—4885.225512	—4885.418708	623.64*i*
**TS5(triplet)**	1.499420	1.577069	1.380860	−4886.821701	—4885.244632	—4885.440841	742.48*i*
**TS5(quintet)**	1.493797	1.572525	1.373271	−4886.802293	—4885.229768	—4885.429022	665.25*i*
**int6(singlet)**	1.503931	1.580520	1.392086	−4886.791255	—4885.210735	—4885.399169	
**int6(triplet)**	1.502605	1.580047	1.387224	−4886.832717	—4885.252670	—4885.445493	
**int6(quintet)**	1.499793	1.578714	1.379023	−4886.857835	—4885.279121	—4885.478812	
**TS7(singlet)**	1.499940	1.575812	1.389564	−4886.748393	—4885.172581	—4885.358829	212.55*i*
**TS7(triplet)**	1.497203	1.573792	1.384679	−4886.762005	—4885.188213	—4885.377326	261.89*i*
**int8(singlet)**	1.501209	1.577501	1.389660	−4886.785785	—4885.208284	—4885.396125	
**int8(triplet)**	1.499135	1.576181	1.383656	−4886.803622	—4885.227441	—4885.419966	
**int8(quintet)**	1.497426	1.575011	1.380164	−4886.79602	—4885.221009	—4885.415856	
**TS9(singlet)**	1.441125	1.515156	1.331334	−4886.779522	—4885.204940	—4885.394927	362.14*i*
**TS9(triplet)**	1.499204	1.576247	1.385531	−4886.824176	—4885.247929	—4885.438645	764.30*i*
**TS9(quintet)**	1.493789	1.571822	1.378053	−4886.812961	—4885.241139	—4885.434908	1246.18*i*
**TS10(singlet)**	1.501808	1.578426	1.389566	−4886.803359	—4885.224933	—4885.413793	370.51*i*
**TS10(triplet)**	1.500619	1.577728	1.386362	−4886.820113	—4885.242385	—4885.433751	353.09*i*
**TS10(quintet)**	1.499100	1.577031	1.381164	—4886.818066	—4885.241035	—4885.436902	290.61*i*
**TS10(R_a,R)**	1.499886	1.577320	1.385315	−4886.821839	—4885.244519	—4885.436524	345.47*i*
**TS10(S_a,S)**	1.499416	1.577031	1.382981	−4886.811891	—4885.23486	—4885.42891	275.69*i*
**TS10(S_a,R)**	1.499022	1.576959	1.382180	−4886.81683	—4885.239871	—4885.43465	177.40*i*

**1a**	0.429905	0.455443	0.372380	−1152.408991	−1151.953548	−1152.036611	
**2a**	0.133411	0.141068	0.102195	−309.804769	−309.663701	−309.702574	
**product**	0.567950	0.600878	0.501680	−1462.247553	−1461.646675	−1461.745873	
**TS-S1(quintet)**	1.500493	1.577990	1.385180	−4886.830752	−4885.252762	−4885.445572	49.47 *i*

Supplementary Table 6. Mössbauer parameter calculations for complexes **A**, **B** and **B***.

[Fe(cyclohexyl)(η^2-styrene)$_3$]$^-$ **(A)**

	Experimental*	$S = 0$	$S = 1$
Isomer shift (mm/s)	0.37	0.35	0.38
Quadrupole splitting (mm/s)	1.16	3.57	-0.82

[Fe(SIMes)(η^2-styrene)$_2$] **(B*)**

	Experimental*	$S = 0$	$S = 1$
Isomer shift (mm/s)	0.39	0.65	0.30
Quadrupole splitting (mm/s)	2.92	3.33	2.26

[Fe(**L5**)(η^2-styrene)$_2$] **(B)**

	Experimental (assigned)*	$S = 0$	$S = 1$
Isomer shift (mm/s)	0.38	0.49	0.38
Quadrupole splitting (mm/s)	2.90	-3.08	3.05

**Note: all experimental* $|\Delta E_Q|$ *are absolute values*

11. X-ray single crystal data

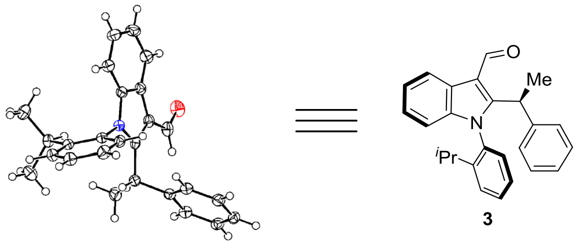

3

CCDC Number	CCDC 2176328
Empirical formula	$C_{26}H_{25}NO$
Formula weight	367.47
Temperature/K	100.00
Wavelength/Å	1.54178
Crystal system	Orthorhombic
Space group (number)	$P2_12_12_1$ (19)
a/Å	7.6785(2)
b/Å	14.6182(3)
c/Å	36.6327(8)
α/°	90
β/°	90
γ/°	90
Volume/Å^3	4111.87(16)
Z	8
ρ_{calc}/gcm^{-3}	1.187
μ/mm^{-1}	0.551
F(000)	1568
Crystal size/mm^3	0.32×0.146×0.078
Crystal color	colorless
Crystal shape	block
Radiation	CuK_α (λ=1.54178 Å)
2Θ range for data collection/°	4.82 to 158.16 (0.79 Å)
Index ranges	$-9 \leq h \leq 9$, $-18 \leq k \leq 18$, $-46 \leq l \leq 46$
Reflections collected	125052
Independent reflections	8828, R(int) = 0.0396, R(sigma) = 0.0132
Data/restraints/parameters	8828/0/511
Goodness-of-fit on F^2	1.033
Final R indexes [I>=2σ (I)]	R_1 = 0.0272, wR_2 = 0.0732
Final R indexes [all data]	R_1 = 0.0281, wR_2 = 0.0739
Largest diff. peak/hole/eÅ^{-3}	0.20/-0.15
Flack X parameter	-0.02(4)

Reference Number: neijd09

CCDC Number: CCDC 2296422

Crystal Structure Report:

$C_{57} H_{83} Cl Fe Mg O_5$

or

$[MgCl(THF)_5][Fe(Cy)(\eta^2\text{-styrene})_3] \cdot C_7H_8$

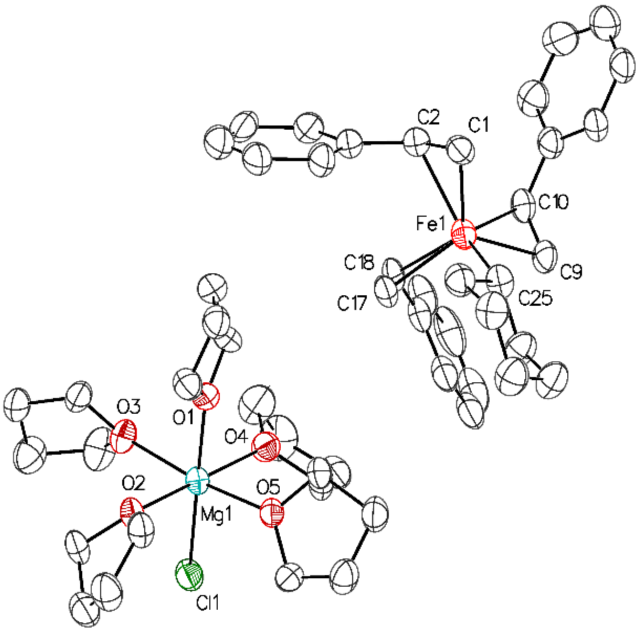

William W. Brennessel

X-ray Crystallographic Facility

Department of Chemistry, University of Rochester

120 Trustee Road

Rochester, NY 14627

Data collection

A crystal (0.609 x 0.169 x 0.009 mm^3) was placed onto a thin glass optical fiber or a nylon loop and mounted on a Rigaku XtaLAB Synergy-S Dualflex diffractometer equipped with a HyPix-6000HE HPC area detector for data collection at 100.01(10) K. A preliminary set of cell constants and an orientation matrix were calculated from a small sampling of reflections[26]. A short pre-experiment was run, from which an optimal data collection strategy was determined. The full data collection was carried out using a PhotonJet (Cu) X-ray source with frame times of 2.00 and 8.00 seconds and a detector distance of 34.0 mm. Series of frames were collected in 0.50º steps in *w* at different 2*q*, *k*, and *f* settings. After the intensity data were corrected for absorption, the final cell constants were calculated from the xyz centroids of 15247 strong reflections from the actual data collection after integration[26]. See Supplementary Table 7 for additional crystal and refinement information.

Structure solution and refinement

The structure was solved using SHELXT[27] and refined using SHELXL[28]. The space group $P2_1$ was determined based on systematic absences and intensity statistics. Most or all non-hydrogen atoms were assigned from the solution. Full-matrix least squares/difference Fourier cycles were performed which located any remaining non-hydrogen atoms. All non-hydrogen atoms were refined with anisotropic displacement parameters. The hydrogen atoms on the metal-coordinating carbon atoms of the styrene ligands were found from the difference Fourier map and refined freely. All other hydrogen atoms were placed in ideal positions and refined as riding atoms with relative isotropic displacement parameters. The final full matrix least squares refinement converged to $R1 = 0.0582$ (F^2, $I > 2s(I)$) and $wR2 = 0.1663$ (F^2, all data).

Structure description

The structure is the one suggested. The asymmetric unit contains one cation, one anion, and one toluene solvent molecule, all in general positions.

Structure manipulation and figure generation were performed using Olex2[29]. Unless noted otherwise all structural diagrams containing anisotropic displacement ellipsoids are drawn at the 50% probability level.

Data collection, structure solution, and structure refinement were conducted at the X-ray

Crystallographic Facility, B04 Hutchison Hall, Department of Chemistry, University of Rochester. The instrument was purchased with funding from NSF MRI program grant CHE-1725028. All publications arising from this report MUST either 1) include William W. Brennessel as a coauthor or 2) acknowledge William W. Brennessel and the X-ray Crystallographic Facility of the Department of Chemistry at the University of Rochester.

Some equations of interest:

$$R_{int} = S\ |F_o^2 - <F_o^2>|\ /\ S|F_o^2|$$

$$R1 = S||F_o|-|F_c||\ /\ S|F_o|$$

$$wR2 = [S[w(F_o^2-F_c^2)^2]\ /\ S\ [w(F_o^2)^2]]^{1/2}$$

where $w = 1\ /\ [s^2\ (F_o^2) + (aP)^2 + bP]$ and

$$P = 1/3\ \max\ (0, F_o^2) + 2/3\ F_c^2$$

$$GOF = S = [S[w(F_o^2-F_c^2)^2]\ /\ (m-n)]^{1/2}$$

where m = number of reflections and n = number of parameters

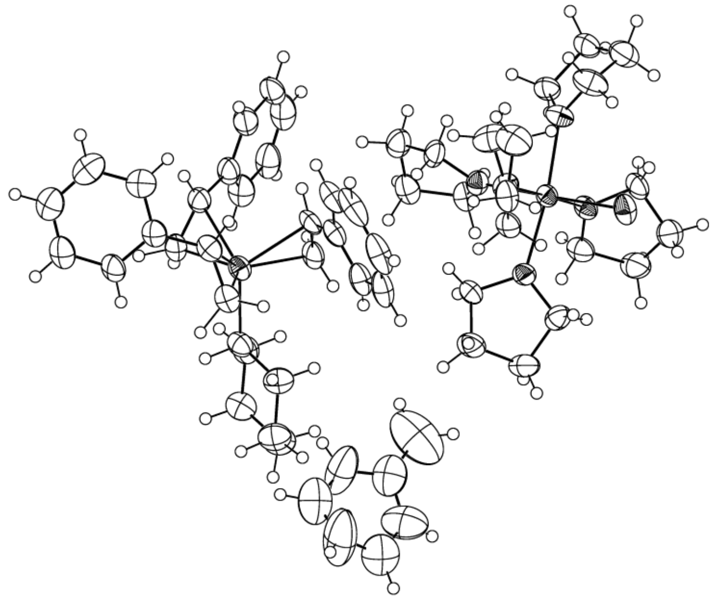

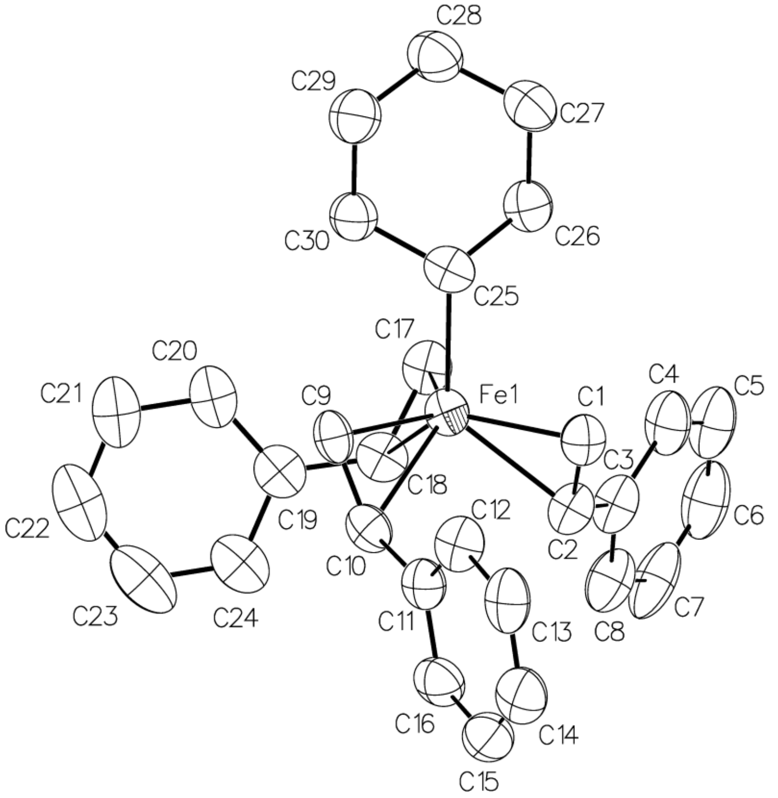
C28
C29
C27
C30
C26
C25
C17
C20
C21
C9
Fe1
C1
C4
C5
C3
C18
C6
C22
C19
C12
C2
C10
C11
C13
C8
C7
C23
C24
C16
C14
C15

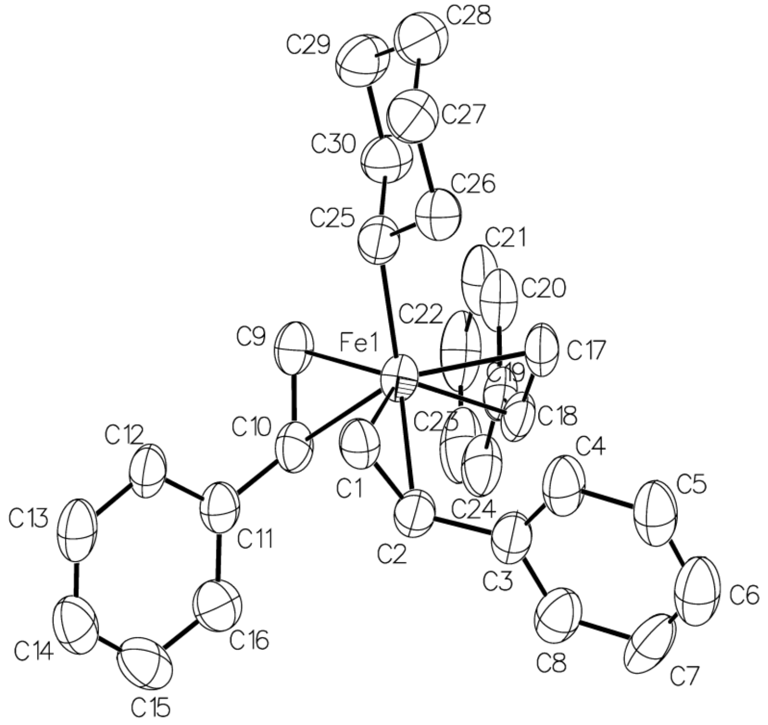
C28
C29
C27
C30
C26
C25
C21
C20
C22
C9
Fe1
C17
C19
C10
C23
C18
C12
C4
C1
C24
C5
C13
C11
C2
C3
C6
C16
C14
C8
C7
C15

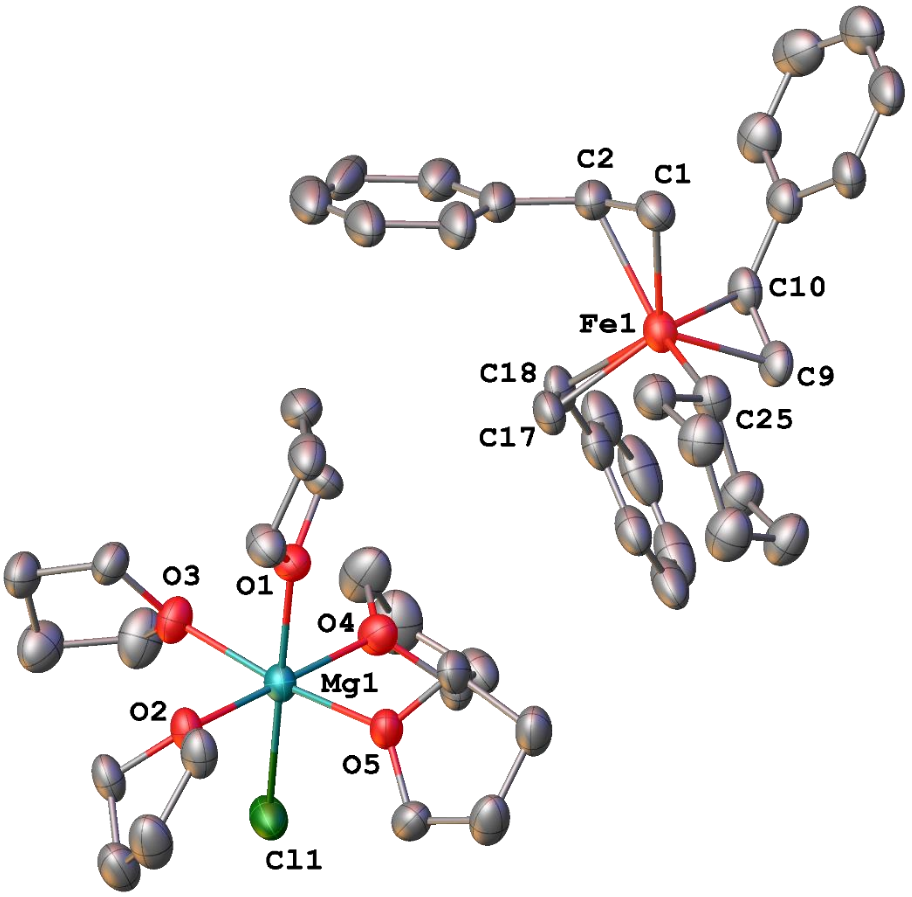
C2
C1
C10
Fe1
C18
C9
C17
C25
O3
O1
O4
Mg1
O2
O5
Cl1

Supplementary Table 7. Crystal data and structure refinement for neijd09.

Identification code	neijd09	
Empirical formula	C57 H83 Cl Fe Mg O5	
Formula weight	963.84	
Temperature	100.01(10) K	
Wavelength	1.54184 Å	
Crystal system	monoclinic	
Space group	$P2_1$	
Unit cell dimensions	*a* = 12.8448(3) Å	α = 90°
	b = 12.6485(3) Å	β = 106.897(3)°
	c = 16.7692(5) Å	γ = 90°
Volume	2606.83(12) $Å^3$	
Z	2	
Density (calculated)	1.228 Mg/m^3	
Absorption coefficient	3.264 mm^{-1}	
F(000)	1040	
Crystal color, morphology	yellow-orange, plate	
Crystal size	0.609 x 0.169 x 0.009 mm^3	
Theta range for data collection	2.754 to 80.213°	
Index ranges	$-16 \le h \le 15$, $-15 \le k \le 16$, $-21 \le l \le 21$	
Reflections collected	47003	
Independent reflections	10936 [*R*(int) = 0.0791]	
Observed reflections	8753	
Completeness to theta = 67.684°	100.0%	
Absorption correction	Multi-scan	
Max. and min. transmission	1.00000 and 0.48102	
Refinement method	Full-matrix least-squares on F^2	
Data / restraints / parameters	10936 / 52 / 623	
Goodness-of-fit on F^2	1.071	
Final *R* indices [*I*>2sigma(*I*)]	*R*1 = 0.0582, *wR*2 = 0.1460	
R indices (all data)	*R*1 = 0.0773, *wR*2 = 0.1663	
Absolute structure parameter	-0.003(4)	
Largest diff. peak and hole	0.580 and -0.528 e.$Å^{-3}$	

Reference Number: neijd10

CCDC Number: CCDC 2296423

Crystal Structure Report:

$C_{45.77}H_{61.08}FeN_2O_{1.23}$

or

(SIMes)Fe(η^2-styrene)$_2$ · 1.23(thf) · 0.77(pentane)

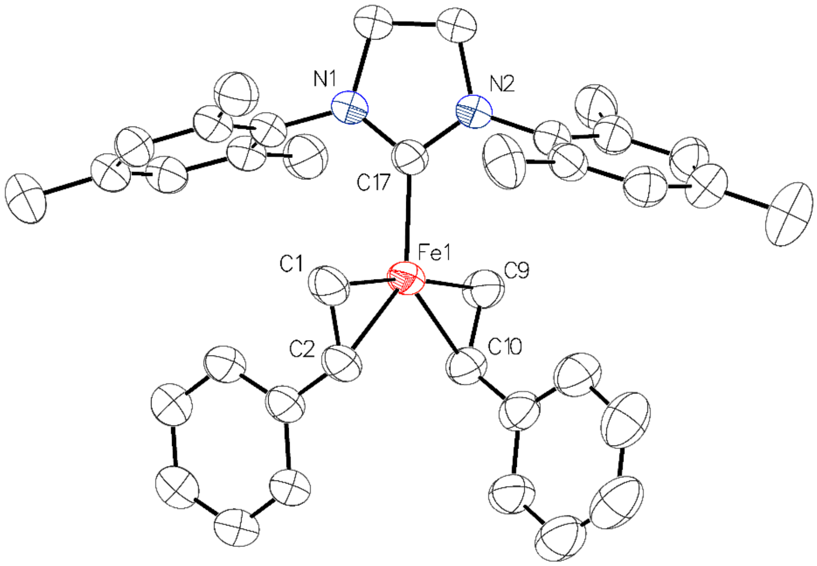

William W. Brennessel

X-ray Crystallographic Facility

Department of Chemistry, University of Rochester

120 Trustee Road

Rochester, NY 14627

Data collection

A crystal (0.605 x 0.227 x 0.033 mm^3) was placed onto a thin glass optical fiber or a nylon loop and mounted on a Rigaku XtaLAB Synergy-S Dualflex diffractometer equipped with a HyPix-6000HE HPC area detector for data collection at 100.00(10) K. A preliminary set of cell constants and an orientation matrix were calculated from a small sampling of reflections[26]. A short pre-experiment was run, from which an optimal data collection strategy was determined. The full data collection was carried out using a PhotonJet (Cu) X-ray source with frame times of 2.29 and 13.75 seconds and a detector distance of 34.0 mm. Series of frames were collected in 0.50° steps in ω at different 2θ, κ, and ϕ settings. After the intensity data were corrected for absorption, the final cell constants were calculated from the xyz centroids of 18890 strong reflections from the actual data collection after integration[26]. See Supplementary Table 8 for additional crystal and refinement information.

Structure solution and refinement

The structure was solved using SHELXT[27] and refined using SHELXL[28]. The space group $P2_1/n$ was determined based on systematic absences. Most or all non-hydrogen atoms were assigned from the solution. Full-matrix least squares/difference Fourier cycles were performed which located any remaining non-hydrogen atoms. All non-hydrogen atoms were refined with anisotropic displacement parameters. The hydrogen atoms on the metal-coordinating carbons atoms were found from the difference Fourier map and refined freely. All other hydrogen atoms were placed in ideal positions and refined as riding atoms with relative isotropic displacement parameters. The final full matrix least squares refinement converged to $R1 = 0.0581$ (F^2, $I > 2\sigma(I)$) and $wR2 = 0.1736$ (F^2, all data).

Structure description

The structure is the one suggested. The asymmetric unit contains one iron complex and solvent of crystallization in general positions. The solvent is a disordered mixture of thf and pentane (0.77:0.23).

Structure manipulation and figure generation were performed using Olex2[29]. Unless noted otherwise all structural diagrams containing anisotropic displacement ellipsoids are drawn at the 50 % probability level.

Data collection, structure solution, and structure refinement were conducted at the X-ray Crystallographic Facility, B04 Hutchison Hall, Department of Chemistry, University of Rochester.

The instrument was purchased with funding from NSF MRI program grant CHE-1725028. All publications arising from this report MUST either 1) include William W. Brennessel as a coauthor or 2) acknowledge William W. Brennessel and the X-ray Crystallographic Facility of the Department of Chemistry at the University of Rochester.

Some equations of interest:

$$R_{int} = \Sigma |F_o^2 - <F_o^2>| / \Sigma|F_o^2|$$

$$R1 = \Sigma||F_o|-|F_c|| / \Sigma|F_o|$$

$$wR2 = [\Sigma[w(F_o^2-F_c^2)^2] / \Sigma [w(F_o^2)^2]]^{1/2}$$

where $w = 1 / [\sigma^2 (F_o^2) + (aP)^2 + bP]$ and

$$P = 1/3 \max (0, F_o^2) + 2/3 F_c^2$$

$$GOF = S = [\Sigma[w(F_o^2-F_c^2)^2] / (m-n)]^{1/2}$$

where m = number of reflections and n = number of parameters

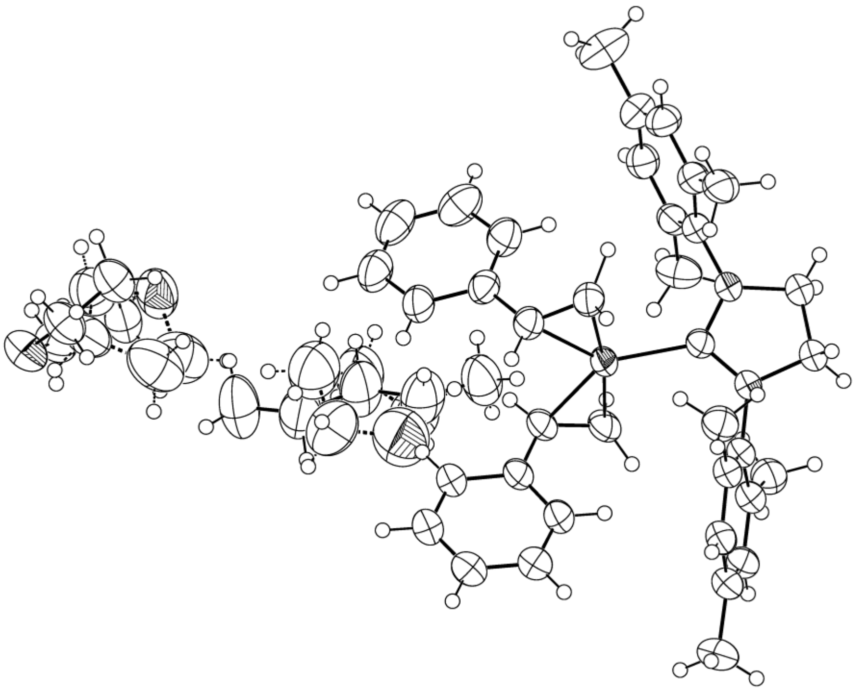

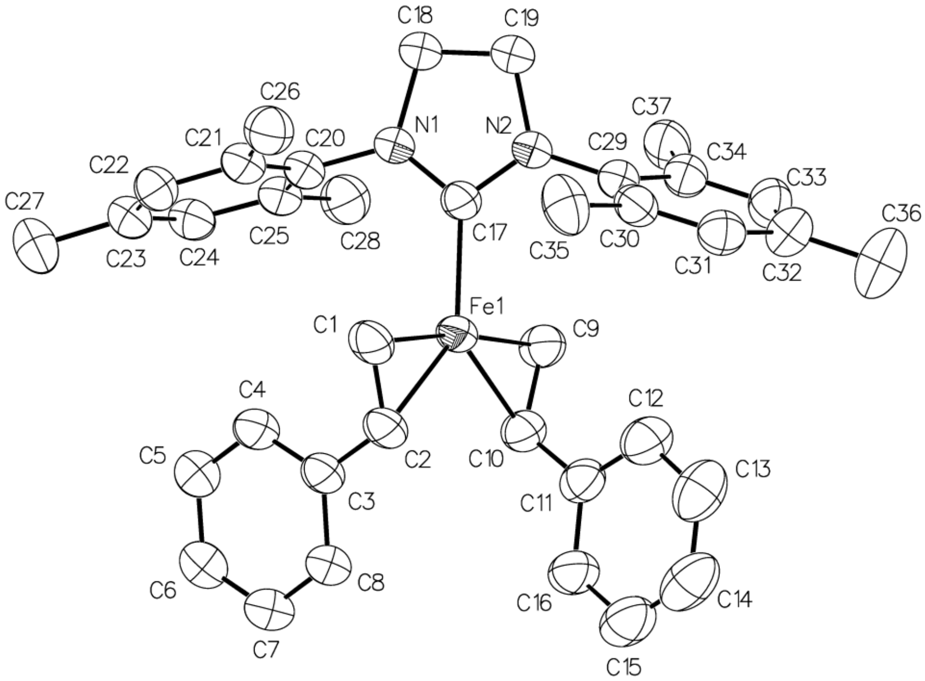
C18
C19
C26
C37
C21
C20
N1
N2
C29
C34
C22
C33
C27
C36
C23
C24
C25
C28
C17
C35
C30
C31
C32
Fe1
C1
C9
C4
C12
C5
C2
C10
C13
C3
C11
C6
C8
C16
C14
C7
C15

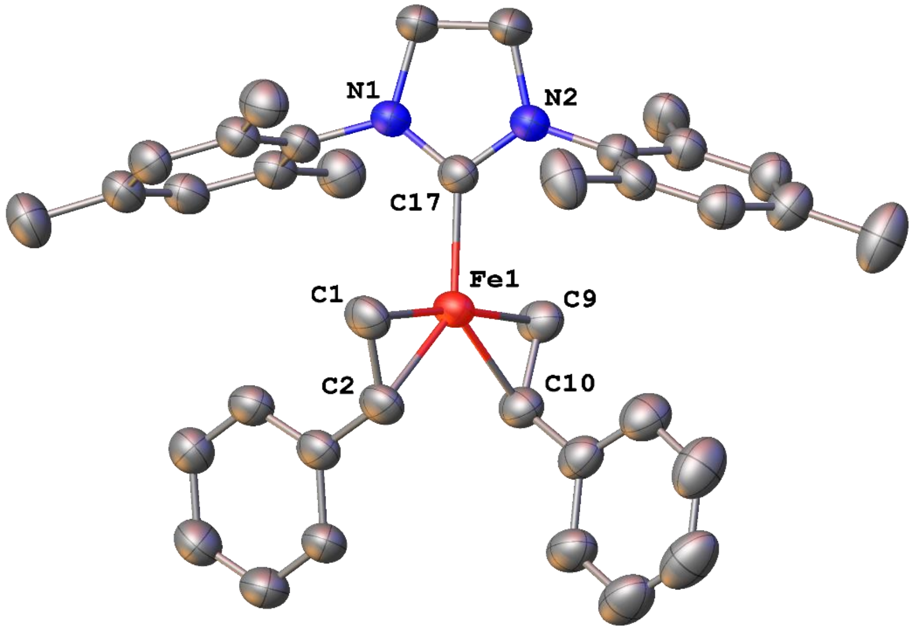
N1
N2
C17
Fe1
C1
C9
C2
C10

Supplementary Table 8. Crystal data and structure refinement for neijd10.

Identification code	neijd10	
Empirical formula	C45.77 H61.08 Fe N2 O1.23	
Formula weight	714.81	
Temperature	100.00(10) K	
Wavelength	1.54184 Å	
Crystal system	monoclinic	
Space group	$P2_1/n$	
Unit cell dimensions	*a* = 12.4806(3) Å	α = 90°
	b = 15.1966(2) Å	β = 96.910(2)°
	c = 21.5141(4) Å	γ = 90°
Volume	4050.78(14) $Å^3$	
Z	4	
Density (calculated)	1.172 Mg/m^3	
Absorption coefficient	3.246 mm^{-1}	
F(000)	1542	
Crystal color, morphology	green, plate	
Crystal size	0.605 x 0.227 x 0.033 mm^3	
Theta range for data collection	3.570 to 80.856°	
Index ranges	$-15 \leq h \leq 15$, $-17 \leq k \leq 19$, $-27 \leq l \leq 27$	
Reflections collected	52251	
Independent reflections	8740 [*R*(int) = 0.0595]	
Observed reflections	7069	
Completeness to theta = 74.504°	100.0%	
Absorption correction	Multi-scan	
Max. and min. transmission	1.00000 and 0.34391	
Refinement method	Full-matrix least-squares on F^2	
Data / restraints / parameters	8740 / 144 / 573	
Goodness-of-fit on F^2	1.083	
Final *R* indices [*I*>2sigma(*I*)]	*R*1 = 0.0581, *wR*2 = 0.1644	
R indices (all data)	*R*1 = 0.0695, *wR*2 = 0.1736	
Largest diff. peak and hole	0.456 and -0.476 e.$Å^{-3}$	

12. NMR spectra

Supplementary Fig. 22. ^{1}H NMR spectrum of **L1**

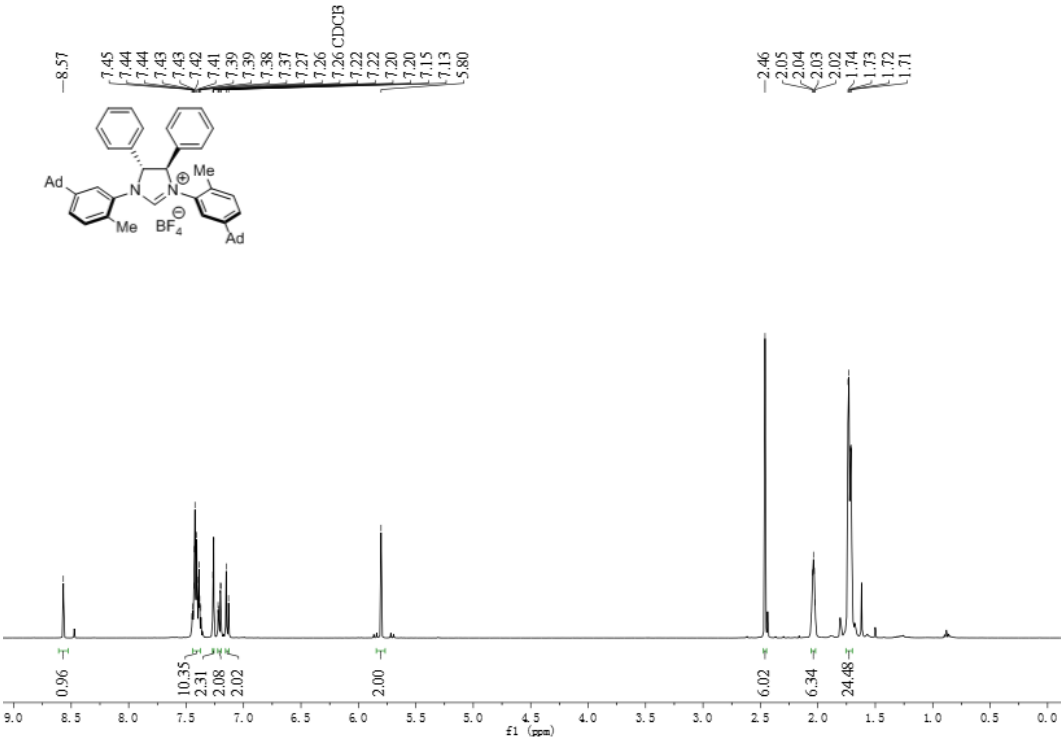

Supplementary Fig. 23. ^{13}C NMR spectrum of **L1**

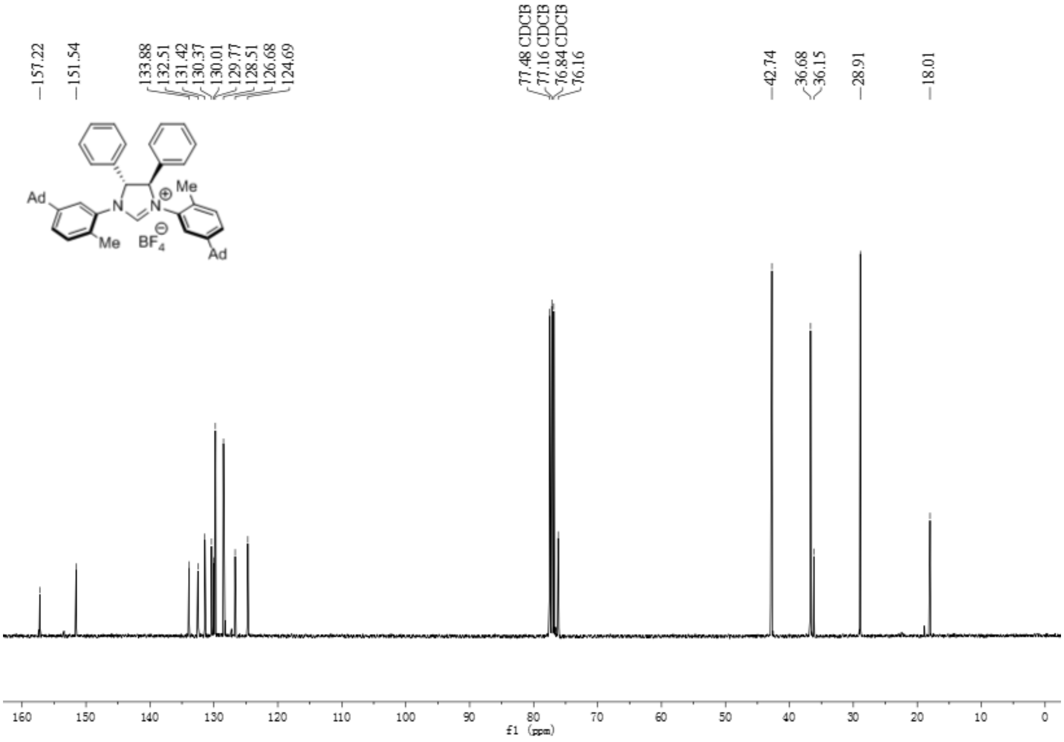

Supplementary Fig. 24. ^{19}F NMR spectrum of **L1**

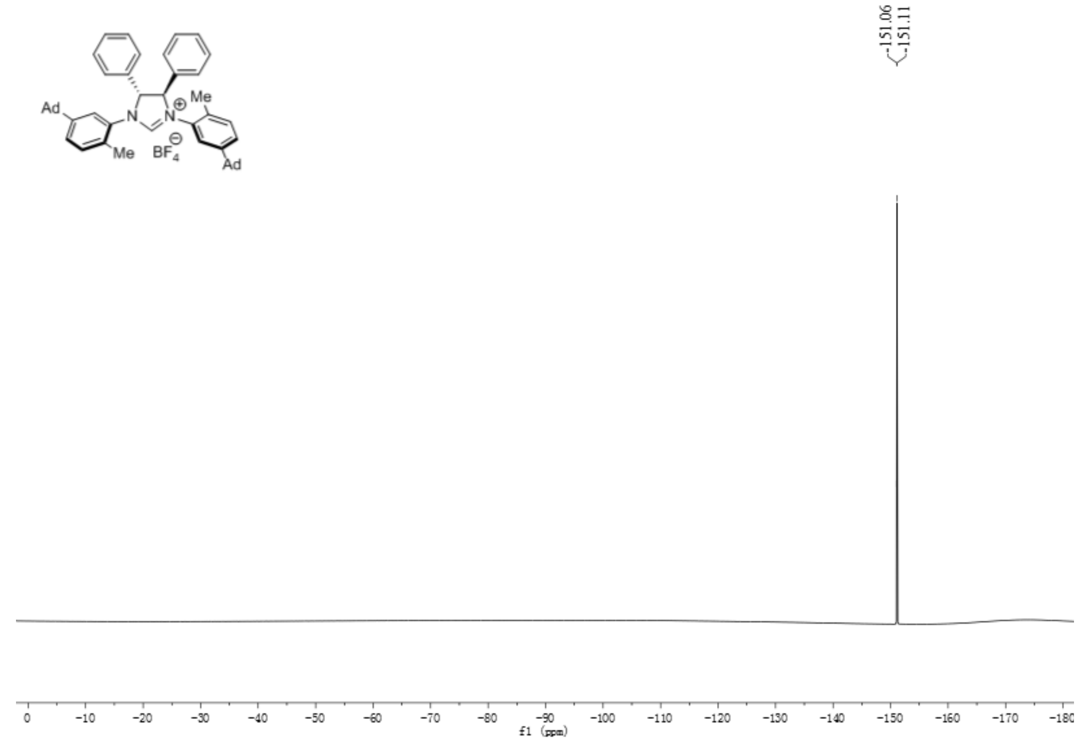

Supplementary Fig. 25. ^{1}H NMR spectrum of **L2**

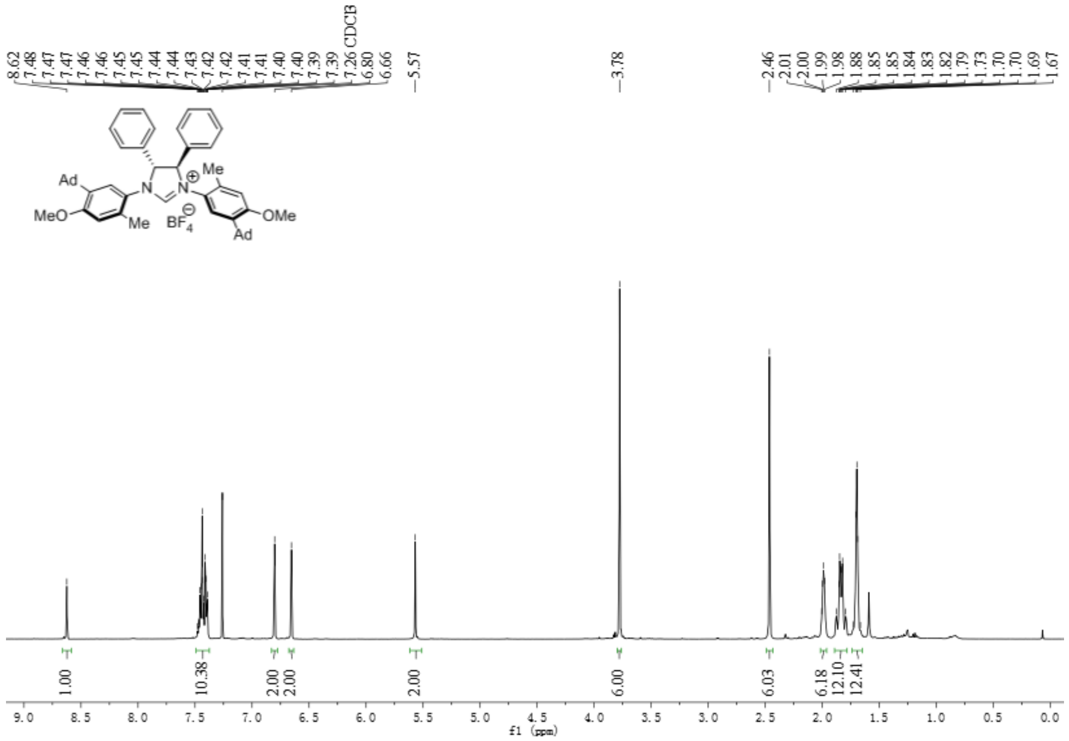

Supplementary Fig. 26. ^{13}C NMR spectrum of **L2**

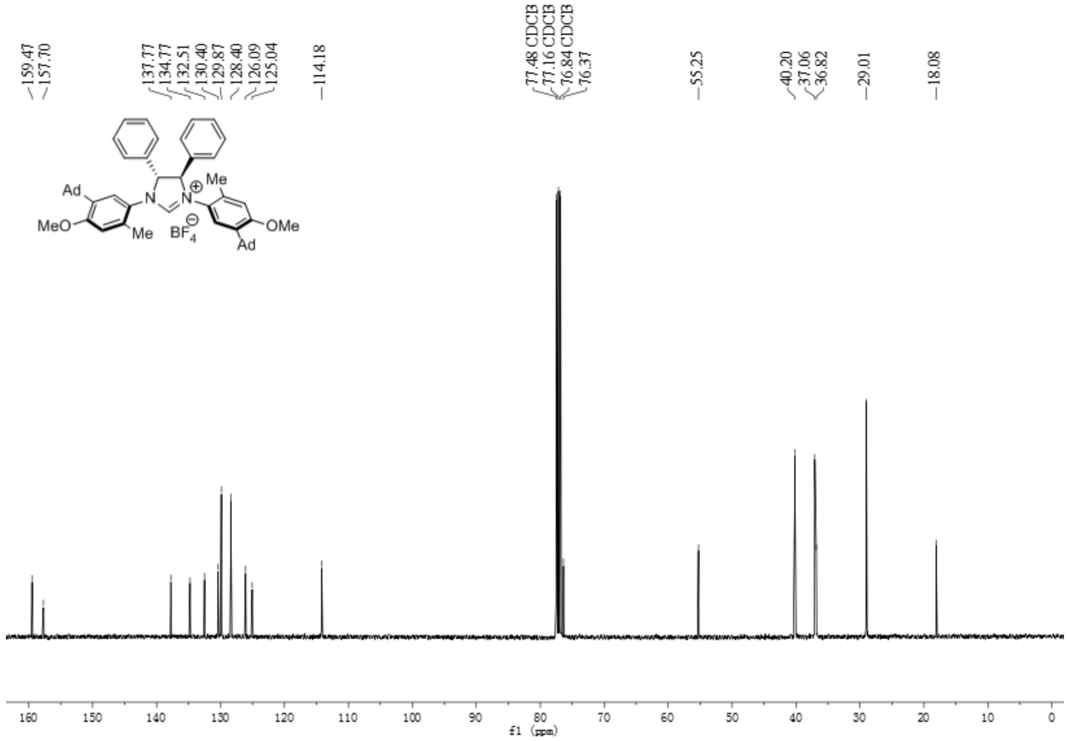

Supplementary Fig. 27. ^{19}F NMR spectrum of **L2**

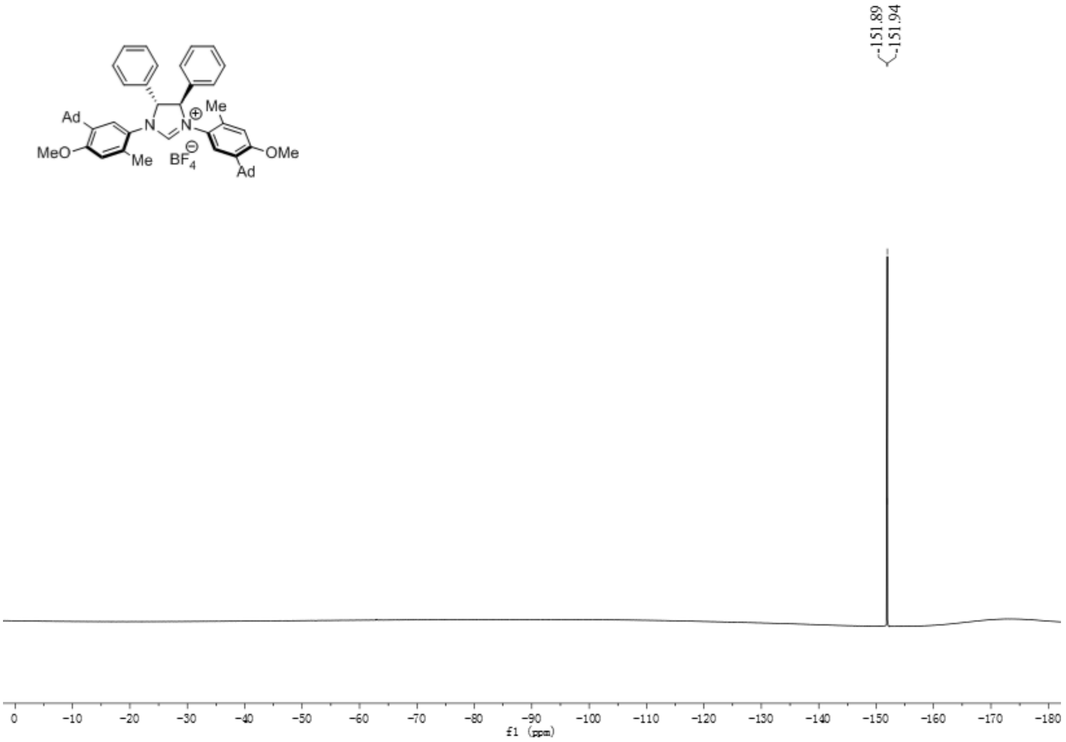

Supplementary Fig. 28. ^{1}H NMR spectrum of **L3**

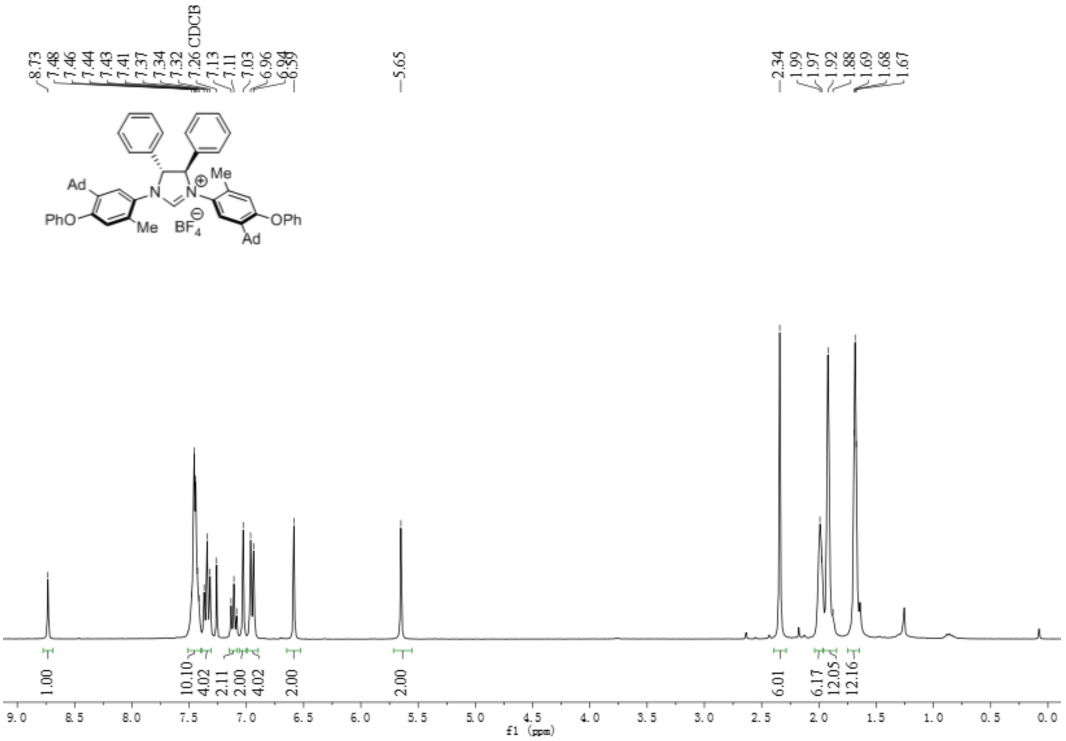

Supplementary Fig. 29. ^{13}C NMR spectrum of **L3**

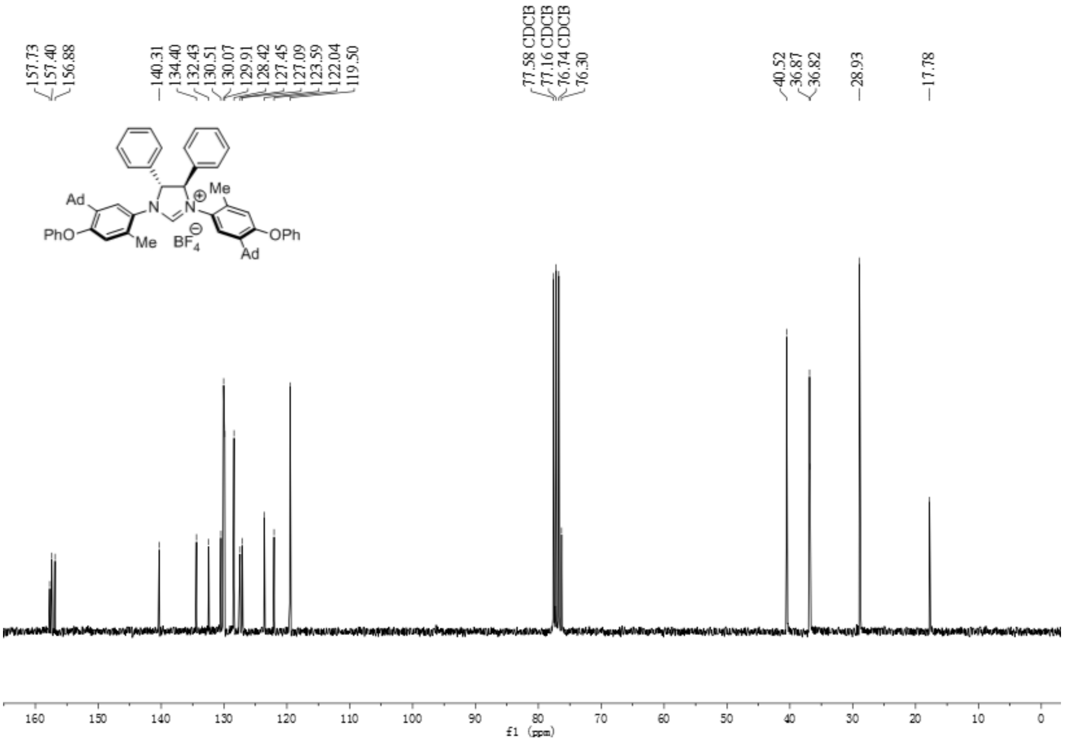

Supplementary Fig. 30. ^{19}F NMR spectrum of **L3**

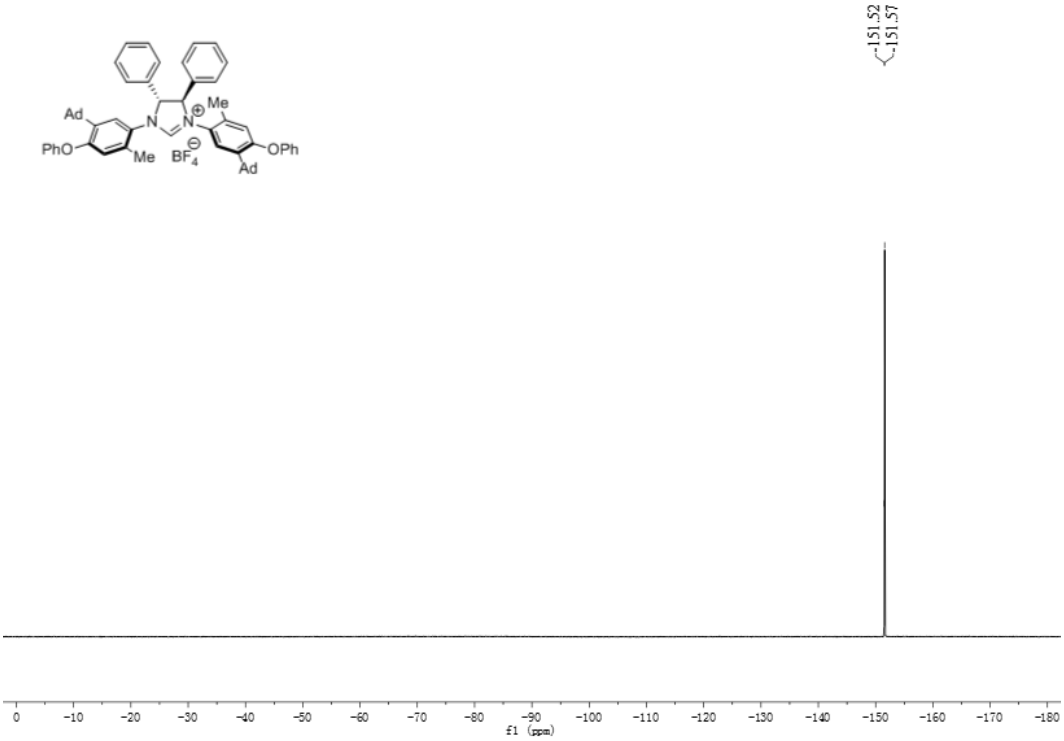

Supplementary Fig. 31. ^{1}H NMR spectrum of **L4**

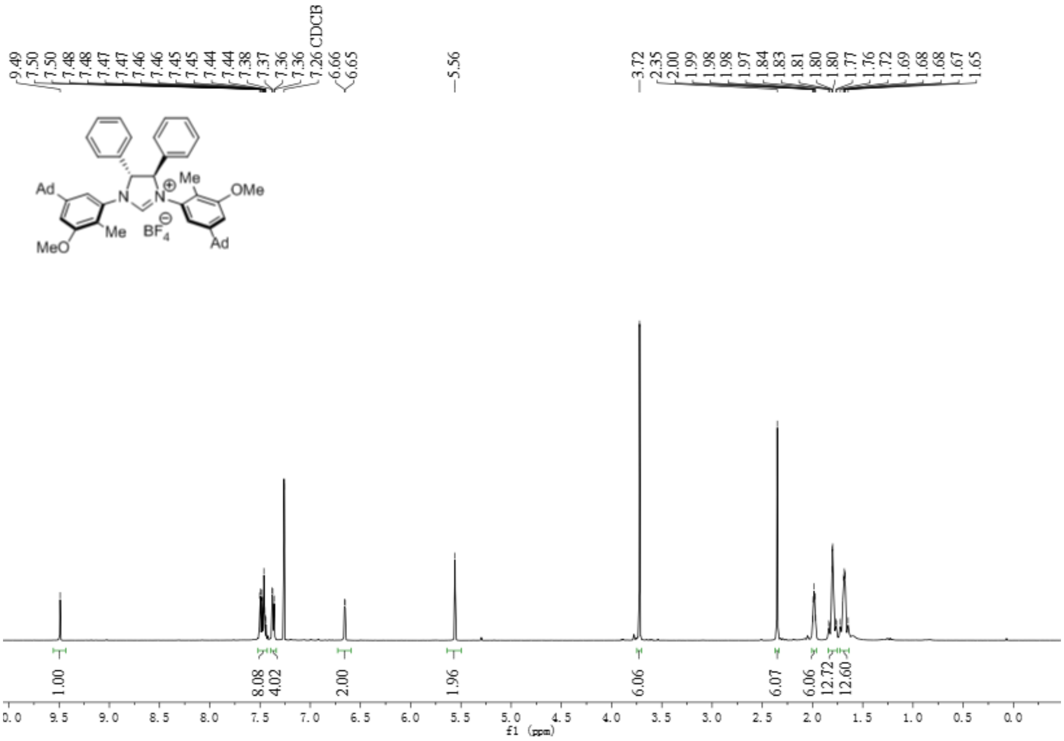

Supplementary Fig. 32. ^{13}C NMR spectrum of **L4**

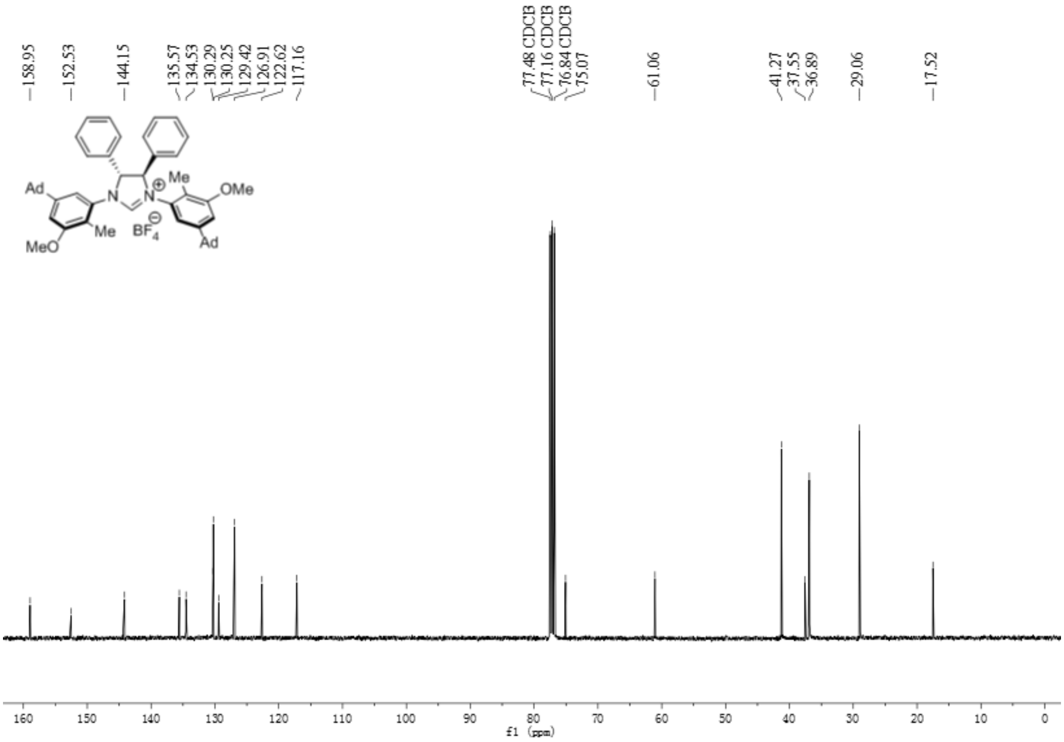

Supplementary Fig. 33. ^{19}F NMR spectrum of **L4**

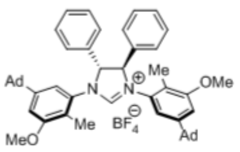

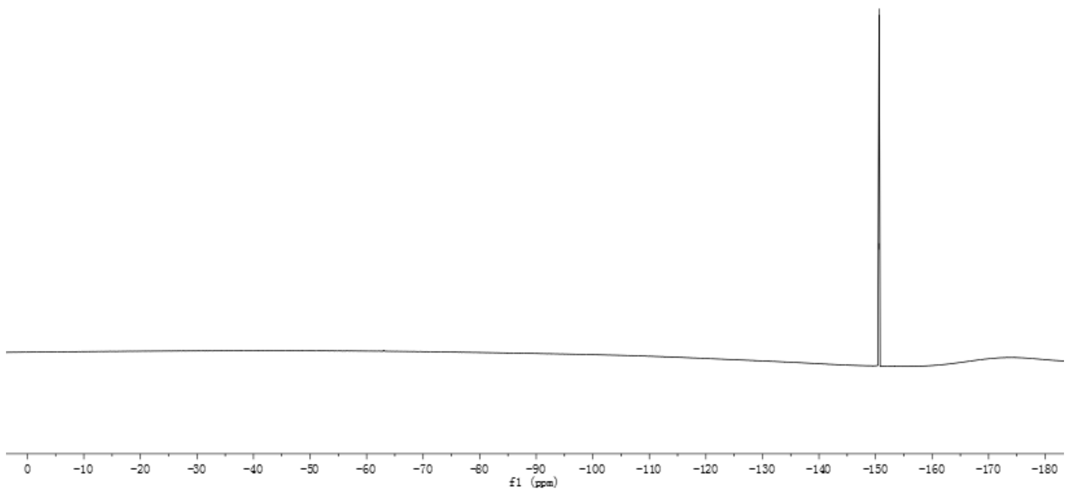

Supplementary Fig. 34. ^{1}H NMR spectrum of **L5**

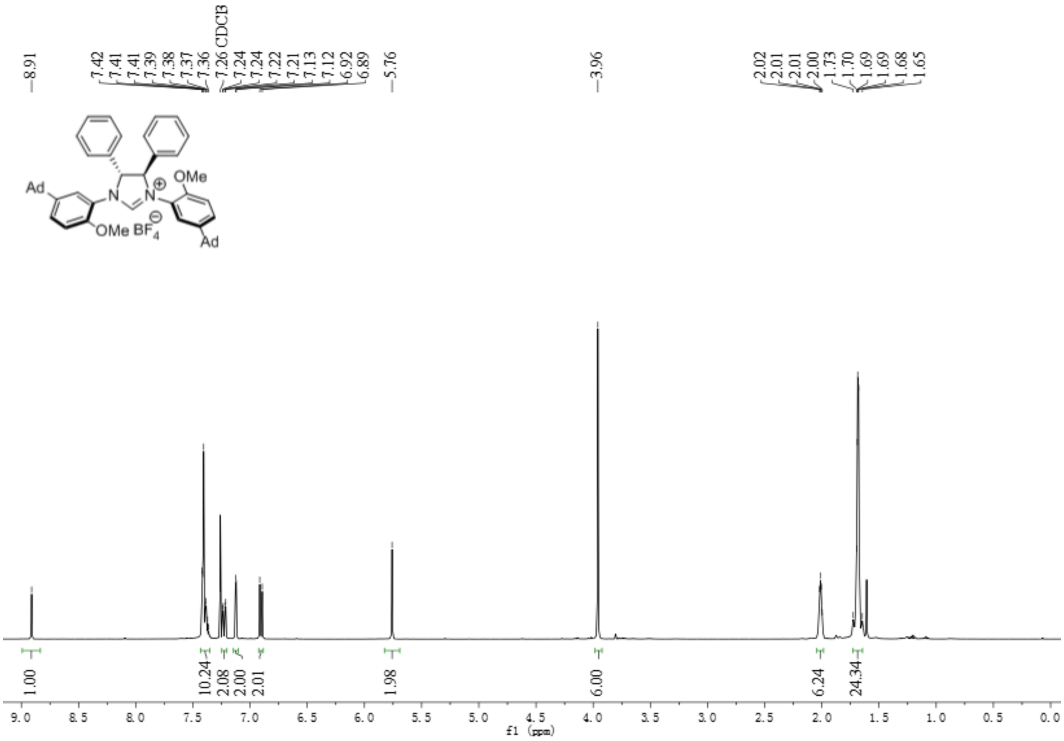

Supplementary Fig. 35. ^{13}C NMR spectrum of **L5**

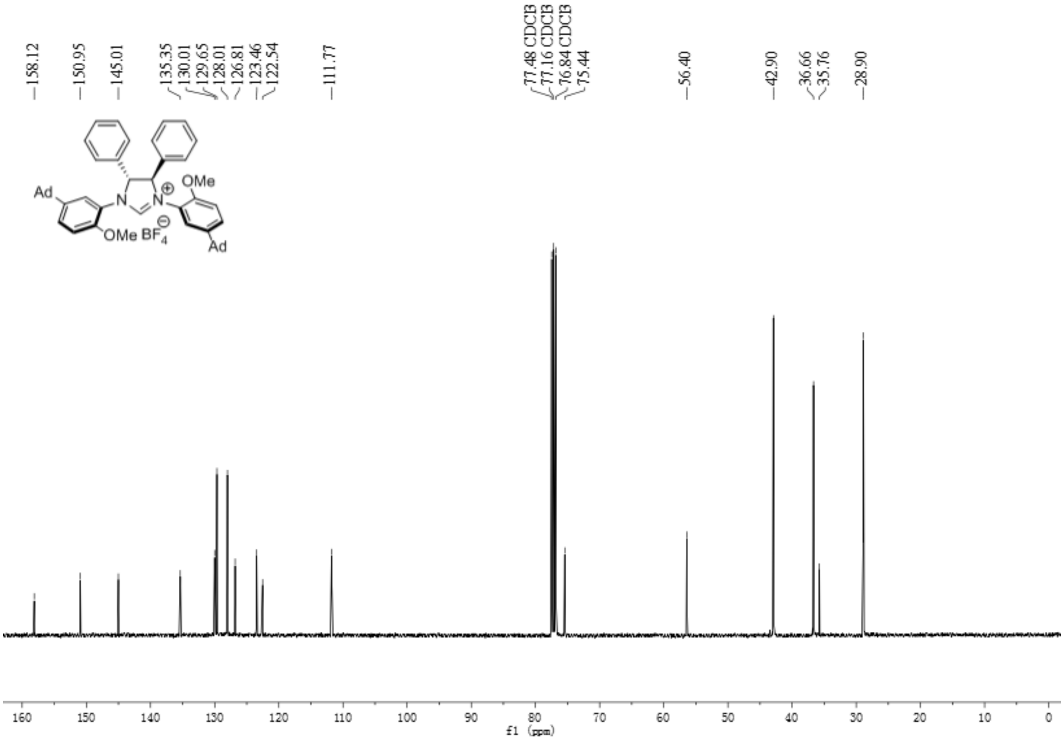

Supplementary Fig. 36. ^{19}F NMR spectrum of **L5**

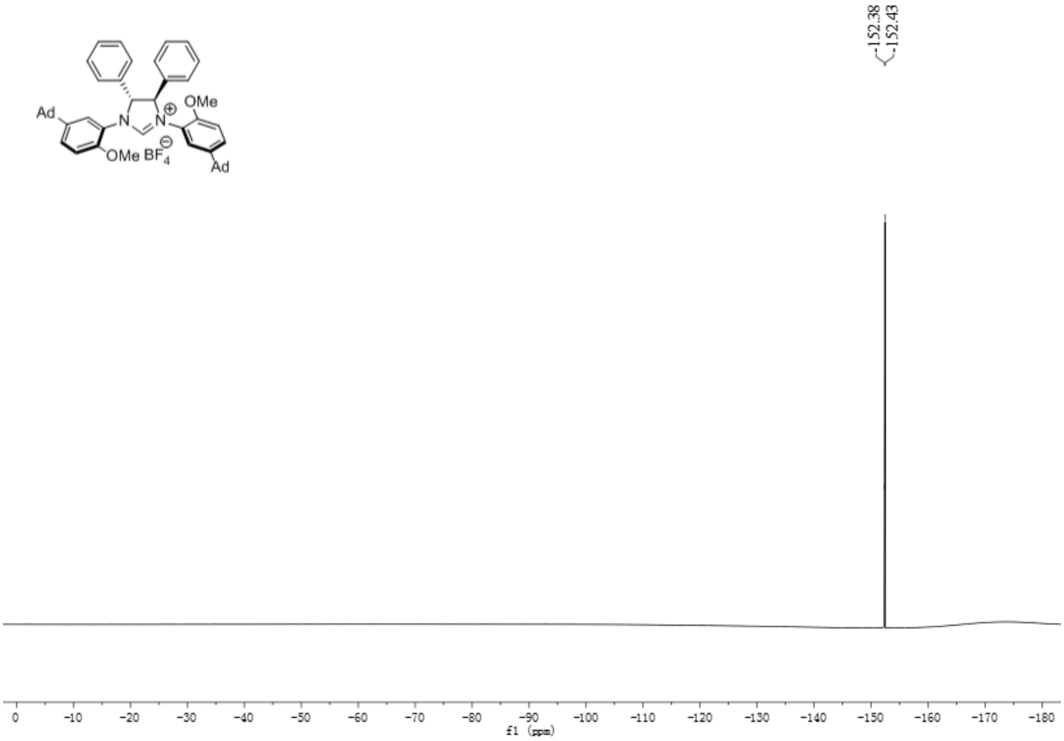

Supplementary Fig. 37. ^{1}H NMR spectrum of **L6**

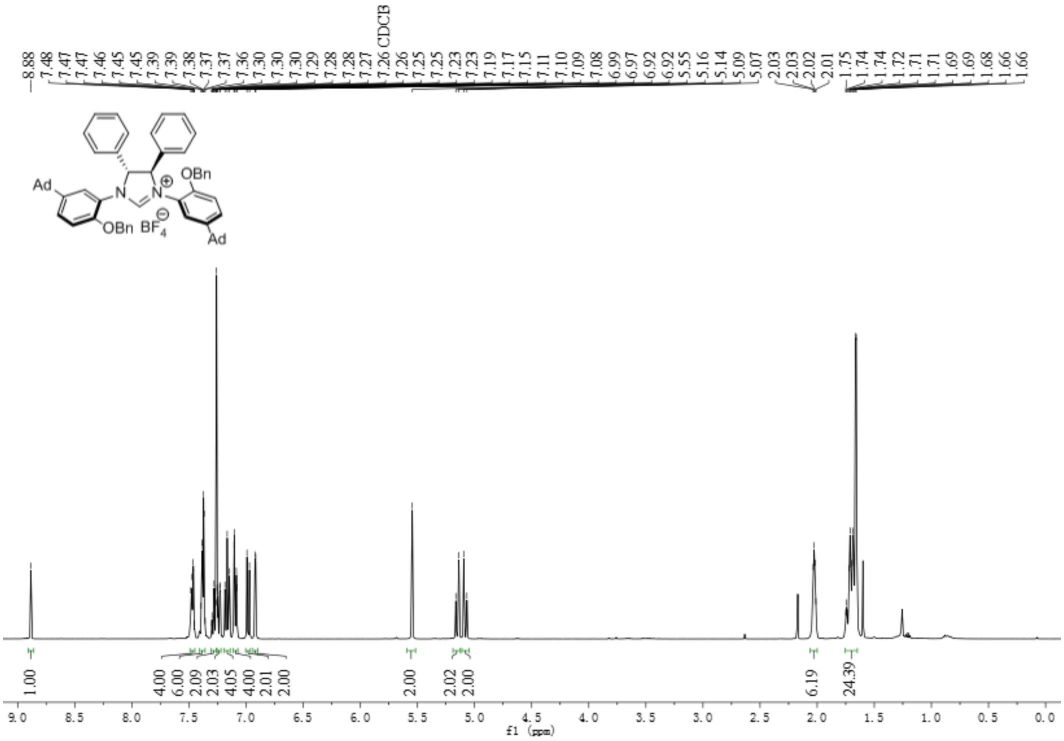

Supplementary Fig. 38. ^{13}C NMR spectrum of **L6**

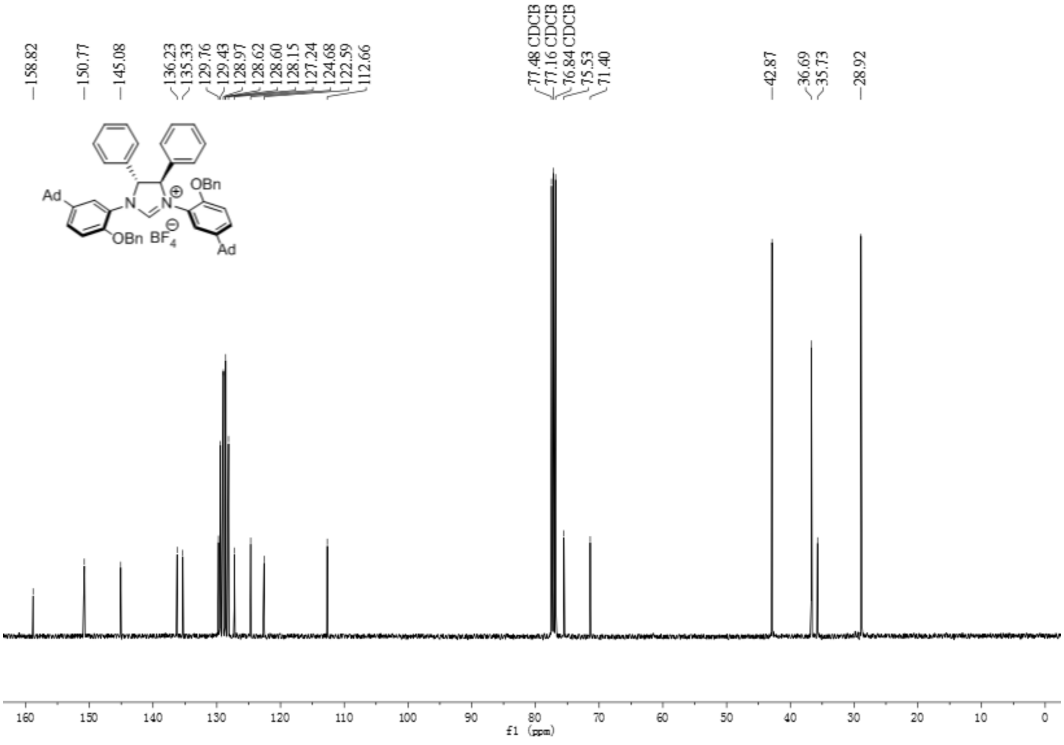

Supplementary Fig. 39. ^{19}F NMR spectrum of **L6**

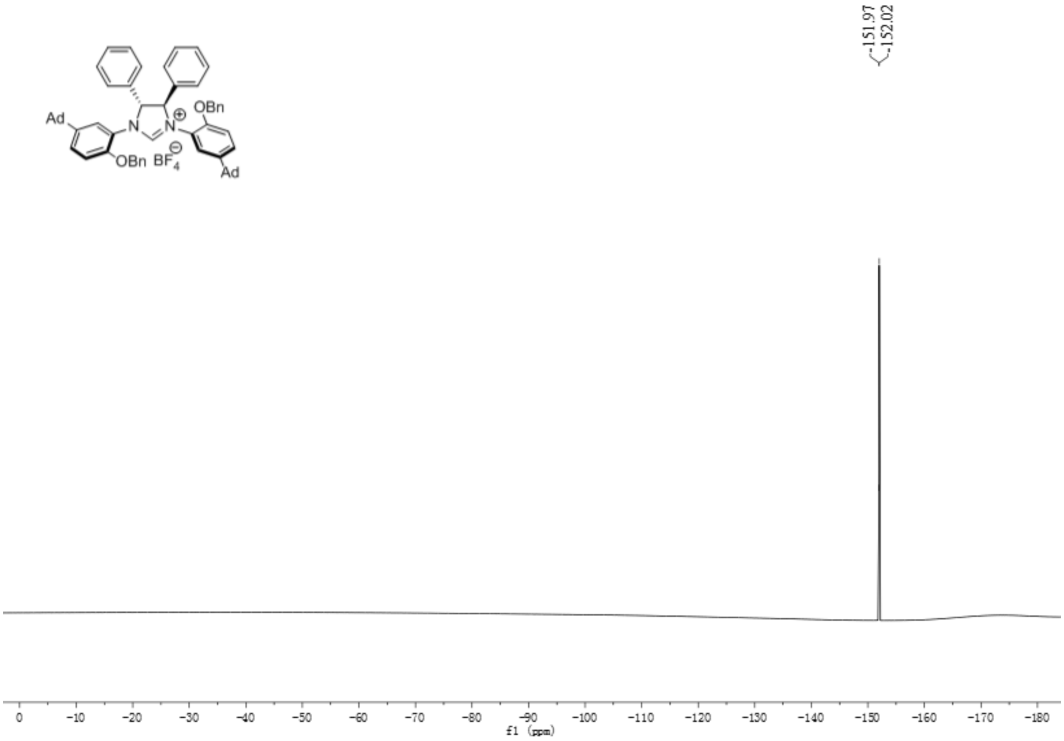

Supplementary Fig. 40. ^{1}H NMR spectrum of **L7**

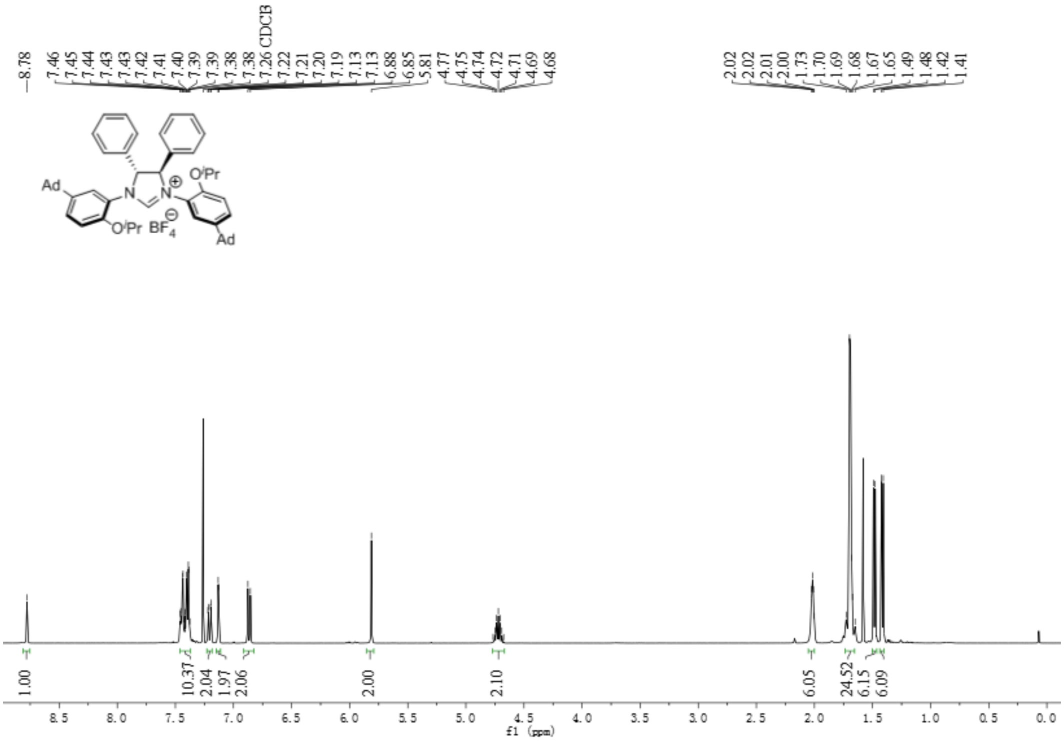

Supplementary Fig. 41. ^{13}C NMR spectrum of **L7**

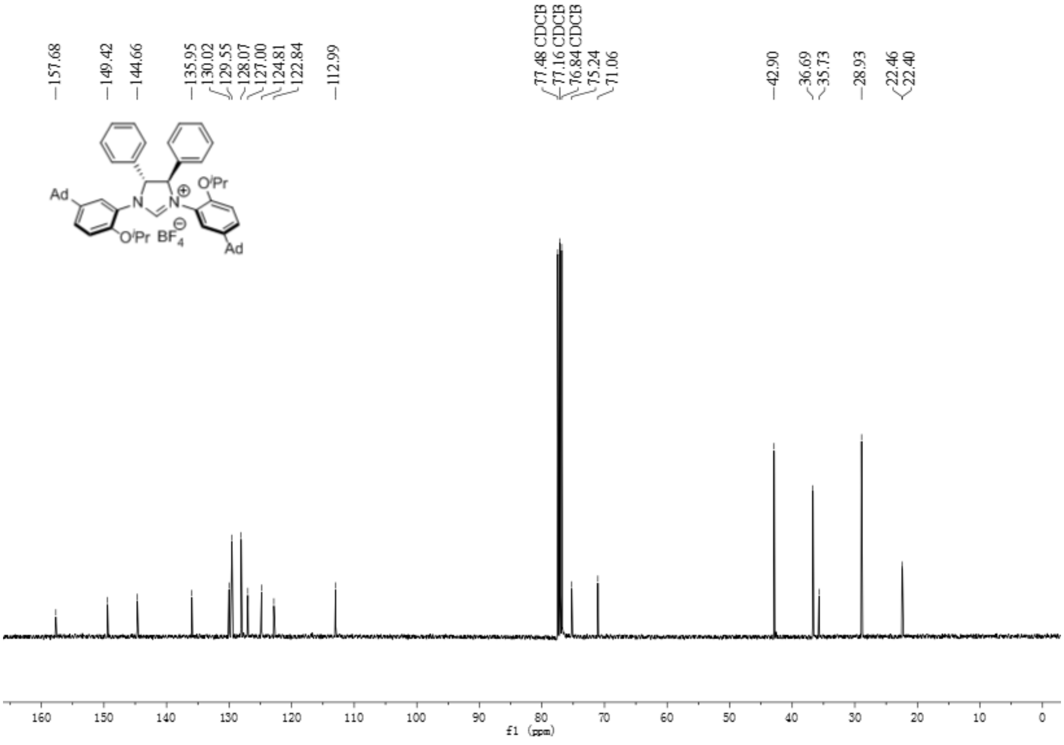

Supplementary Fig. 42. ^{19}F NMR spectrum of **L7**

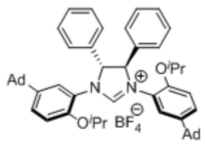

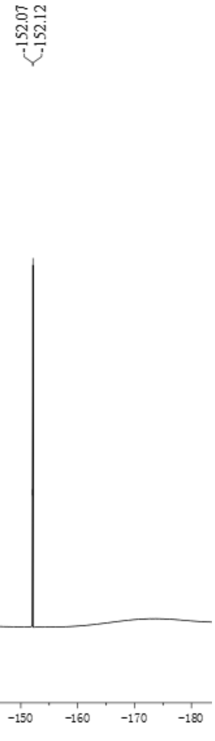

Supplementary Fig. 43. ^{1}H NMR spectrum of **L8**

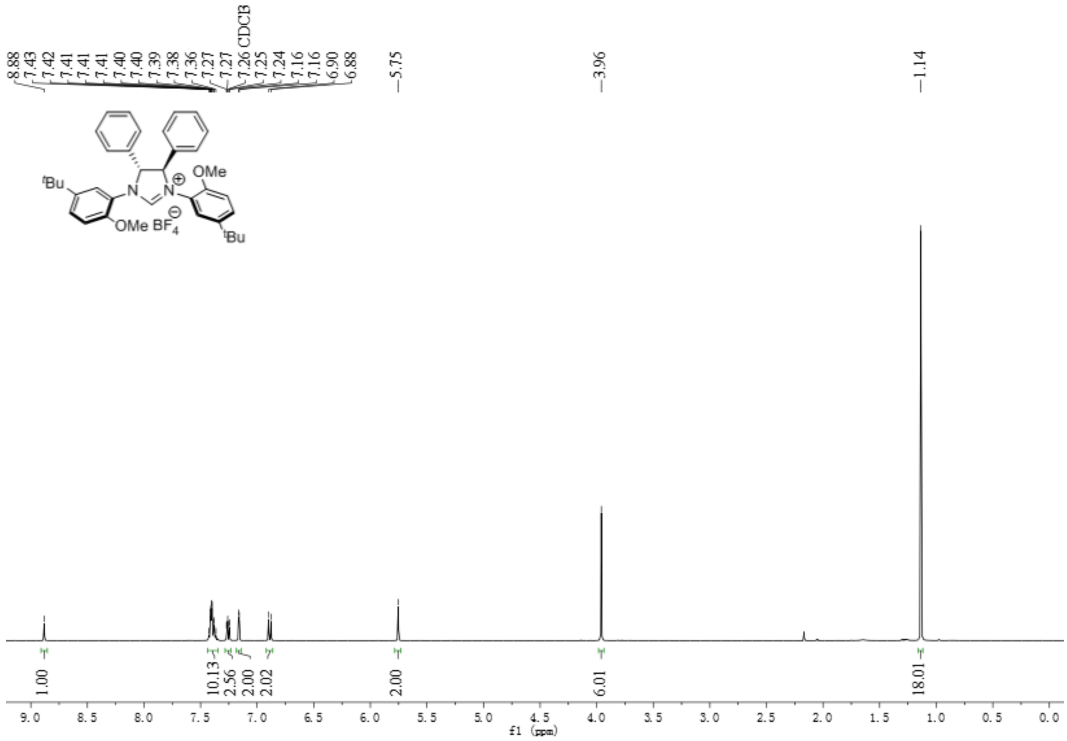

Supplementary Fig. 44. ^{13}C NMR spectrum of **L8**

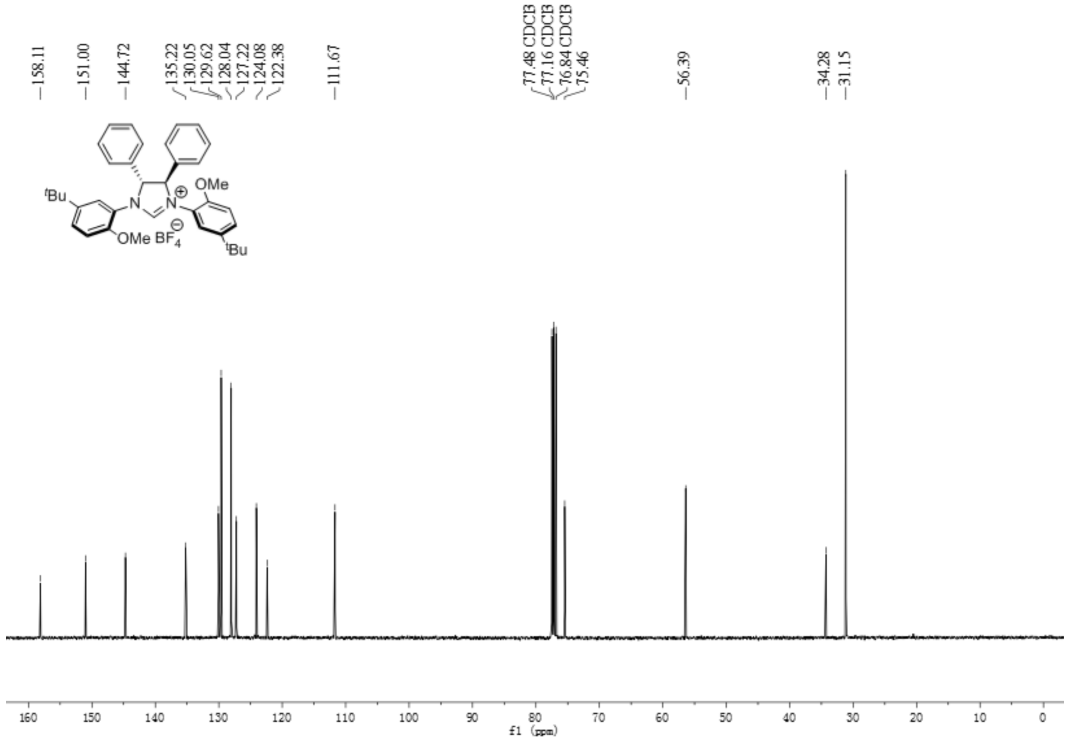

Supplementary Fig. 45. ^{19}F NMR spectrum of **L8**

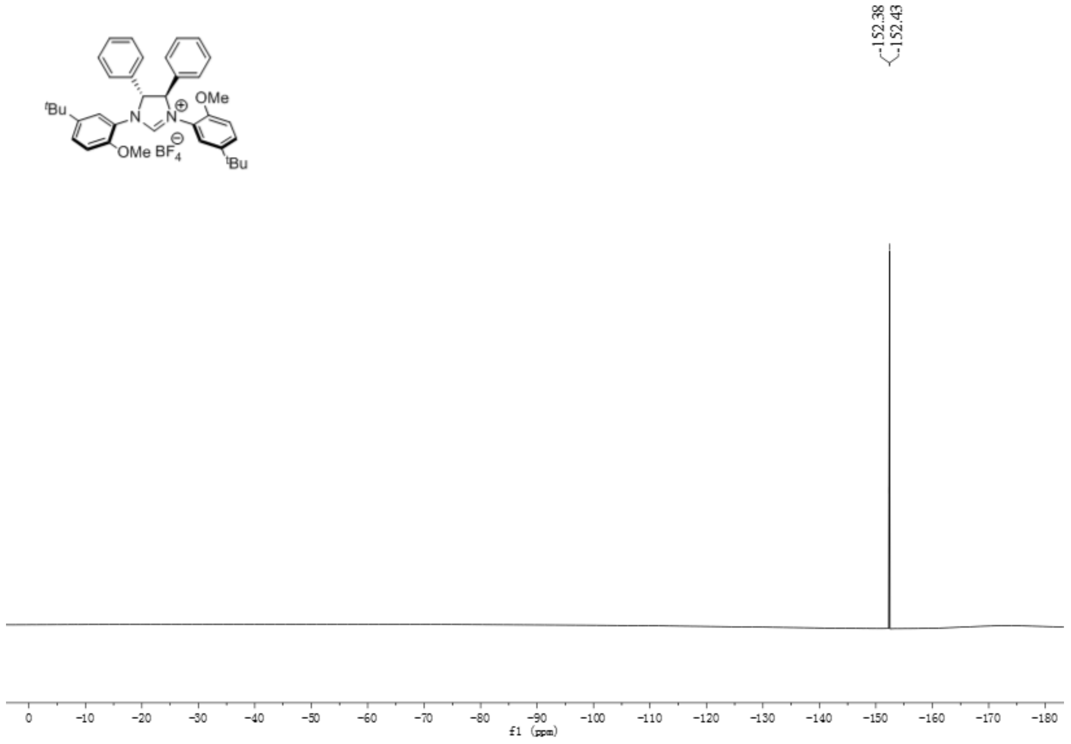

Supplementary Fig. 46. ^{1}H NMR spectrum of **L9**

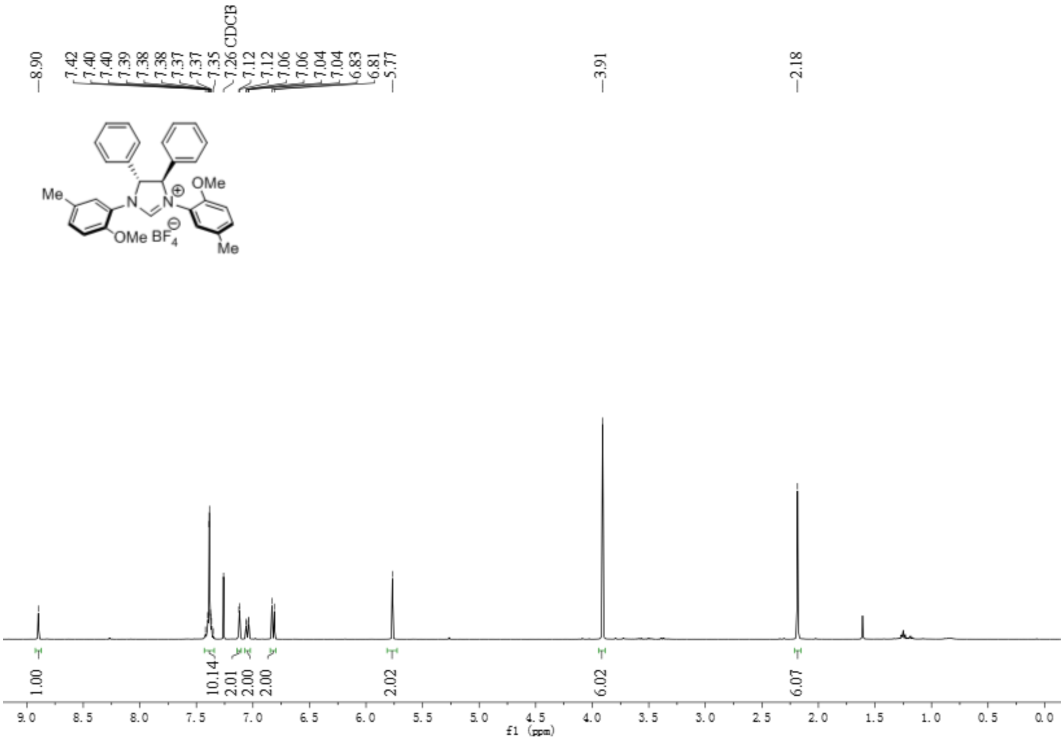

Supplementary Fig. 47. ^{13}C NMR spectrum of **L9**

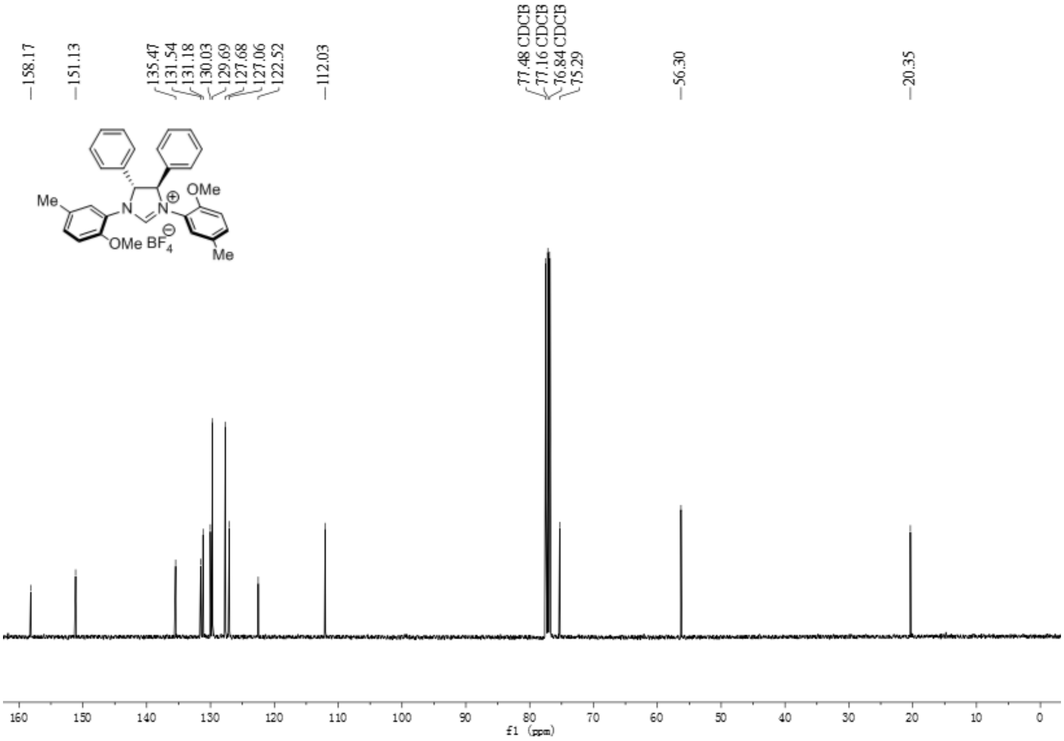

Supplementary Fig. 48. ^{19}F NMR spectrum of **L9**

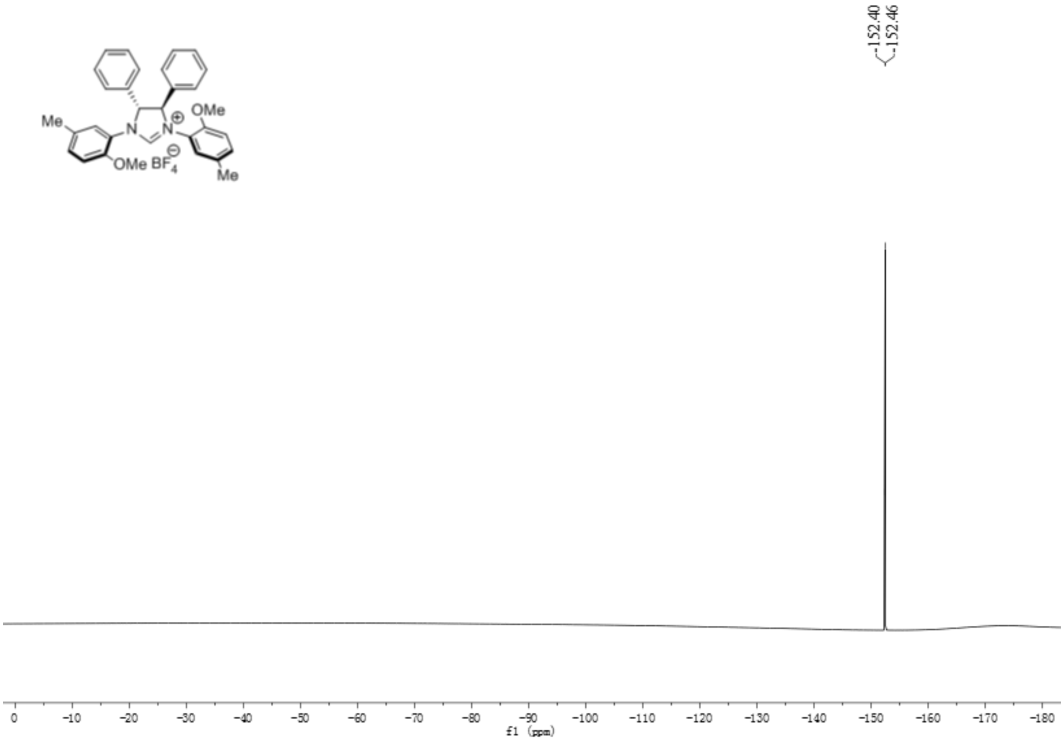

Supplementary Fig. 49. ^{1}H NMR spectrum of **1a**

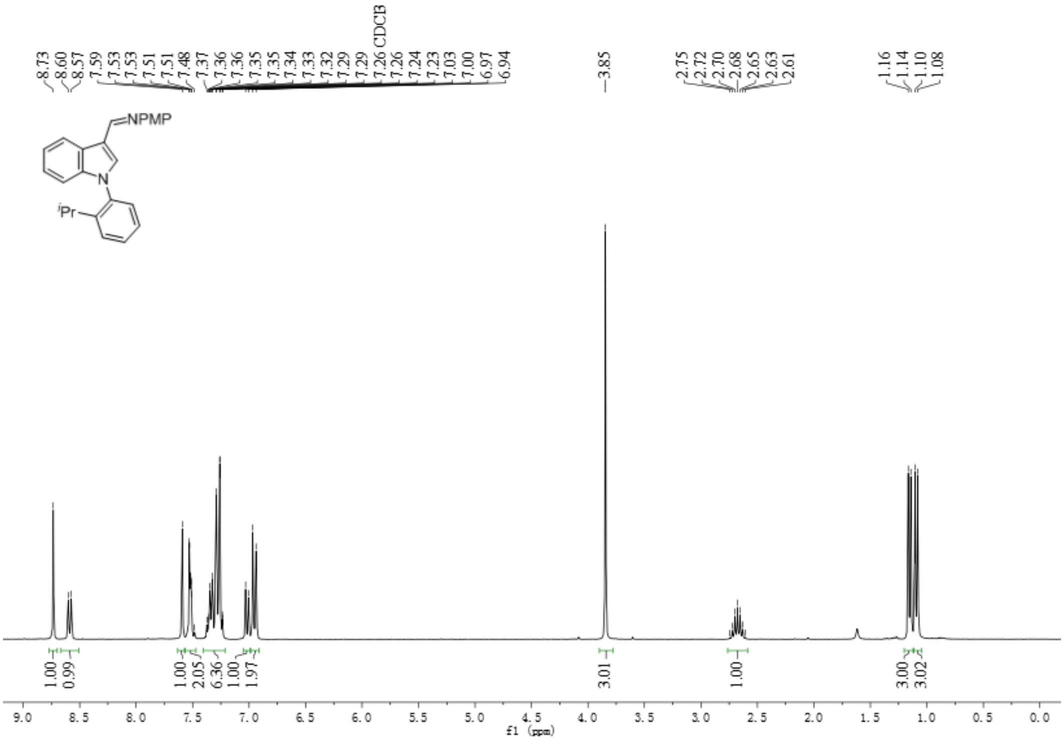

Supplementary Fig. 50. ^{13}C NMR spectrum of **1a**

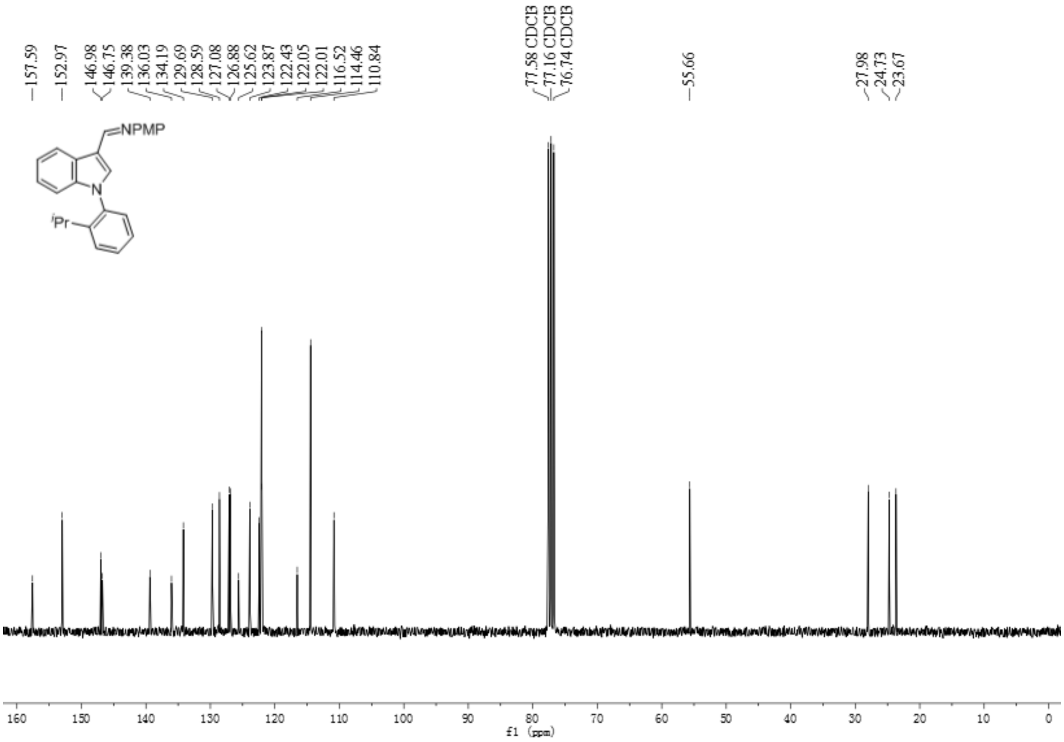

Supplementary Fig. 51. ^{1}H NMR spectrum of **1b**

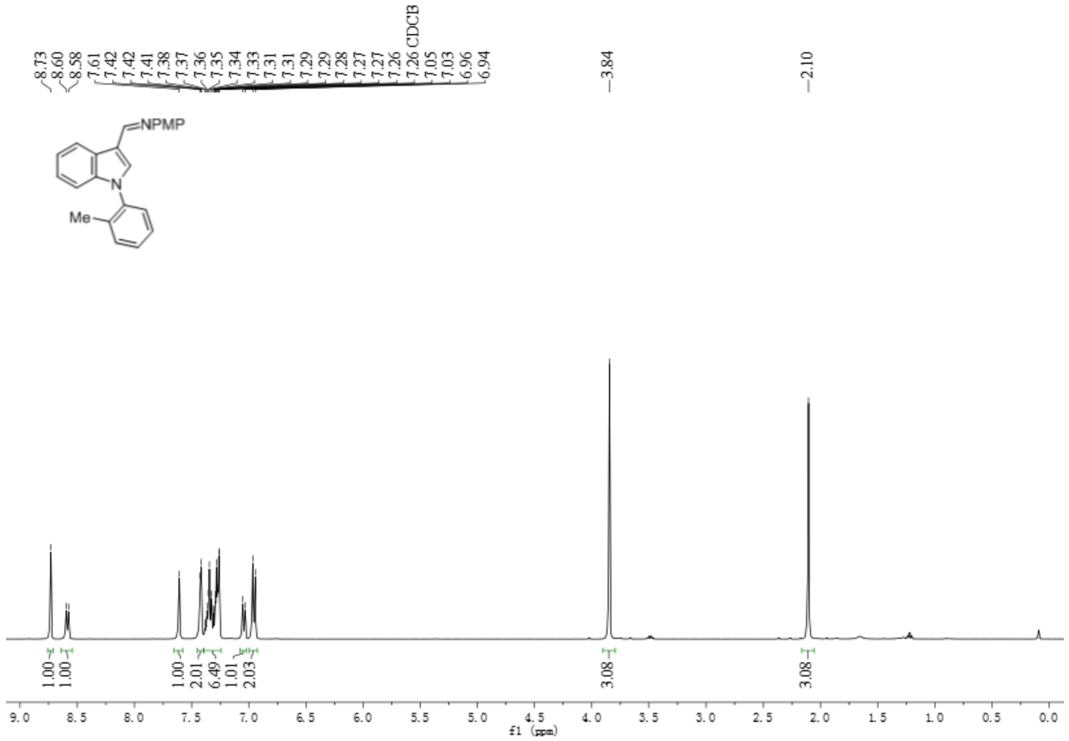

Supplementary Fig. 52. ^{13}C NMR spectrum of **1b**

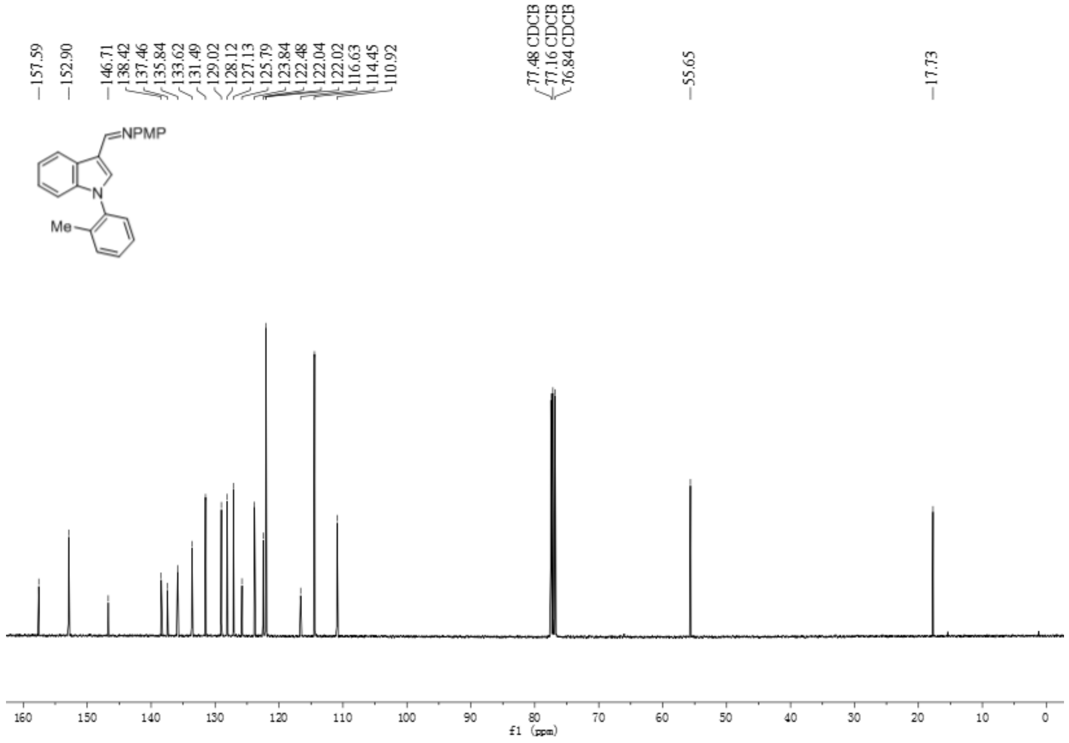

Supplementary Fig. 53. ^{1}H NMR spectrum of **1c**

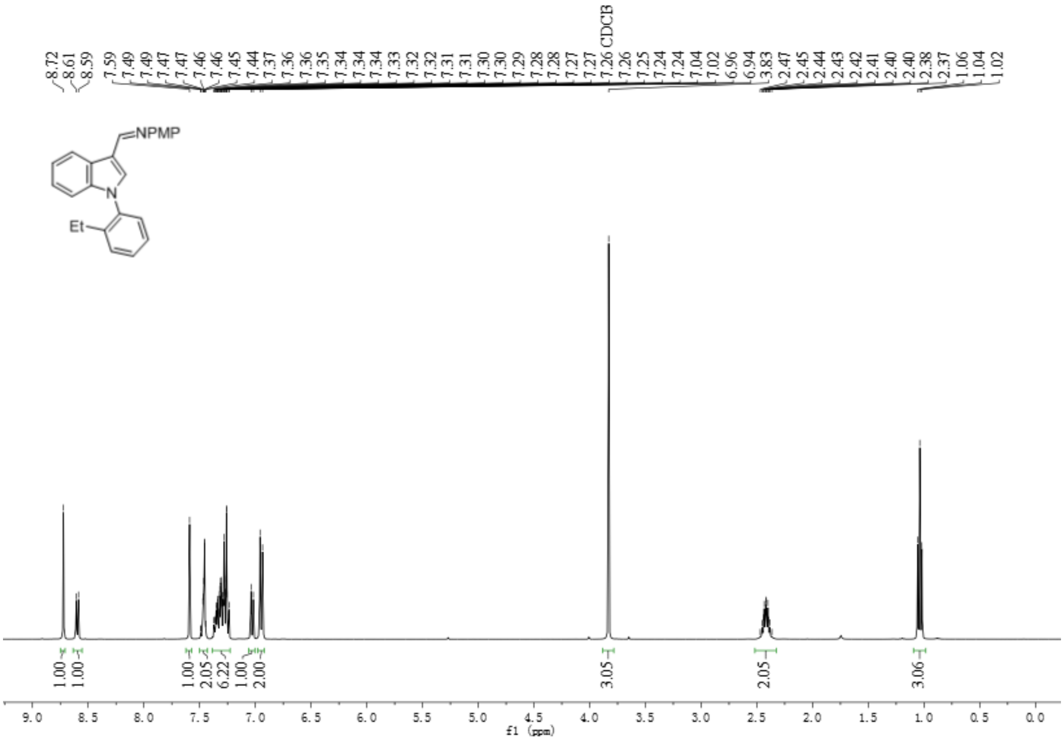

Supplementary Fig. 54. ^{13}C NMR spectrum of **1c**

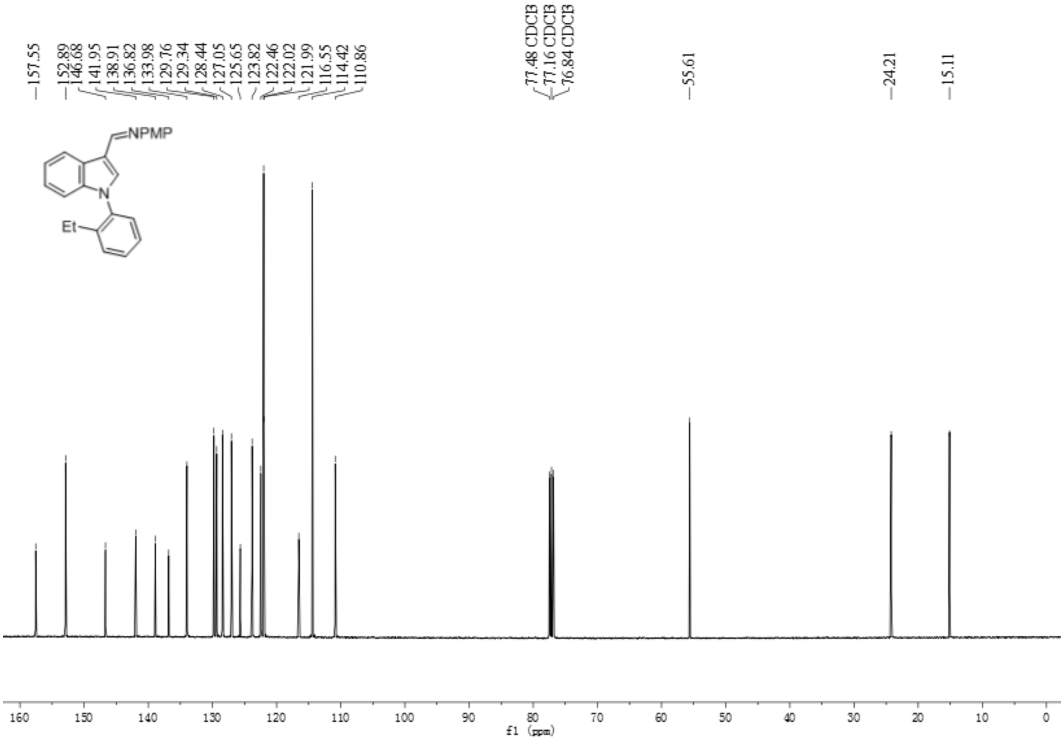

Supplementary Fig. 55. ^{1}H NMR spectrum of **1d**

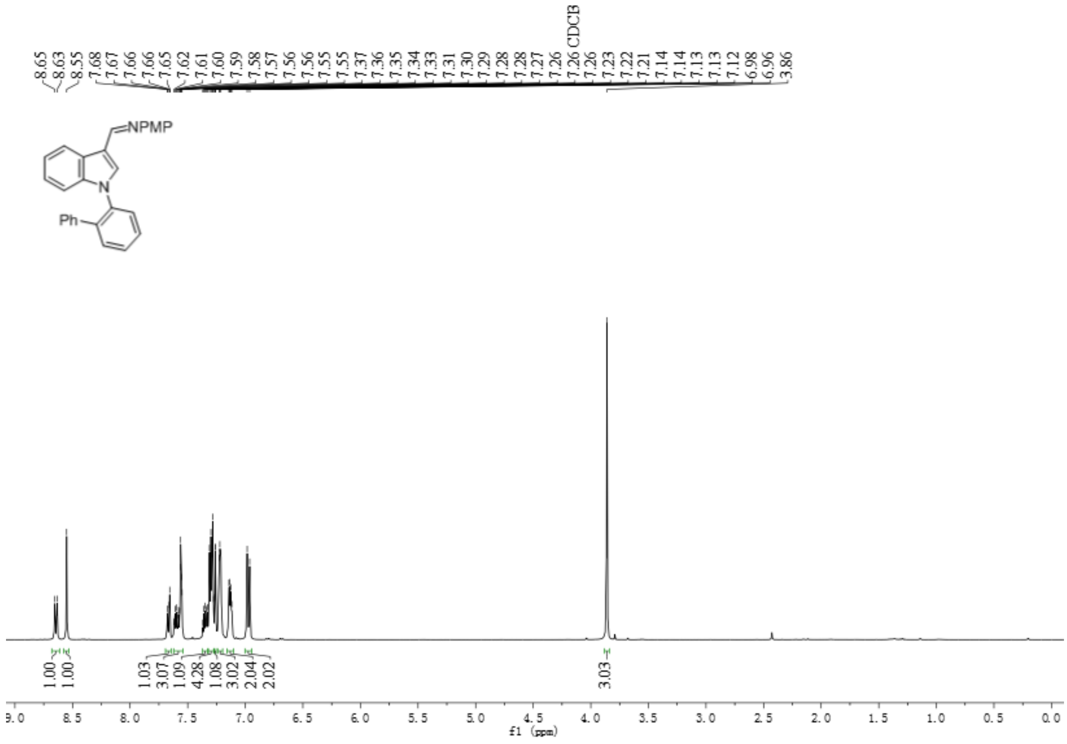

Supplementary Fig. 56. ^{13}C NMR spectrum of **1d**

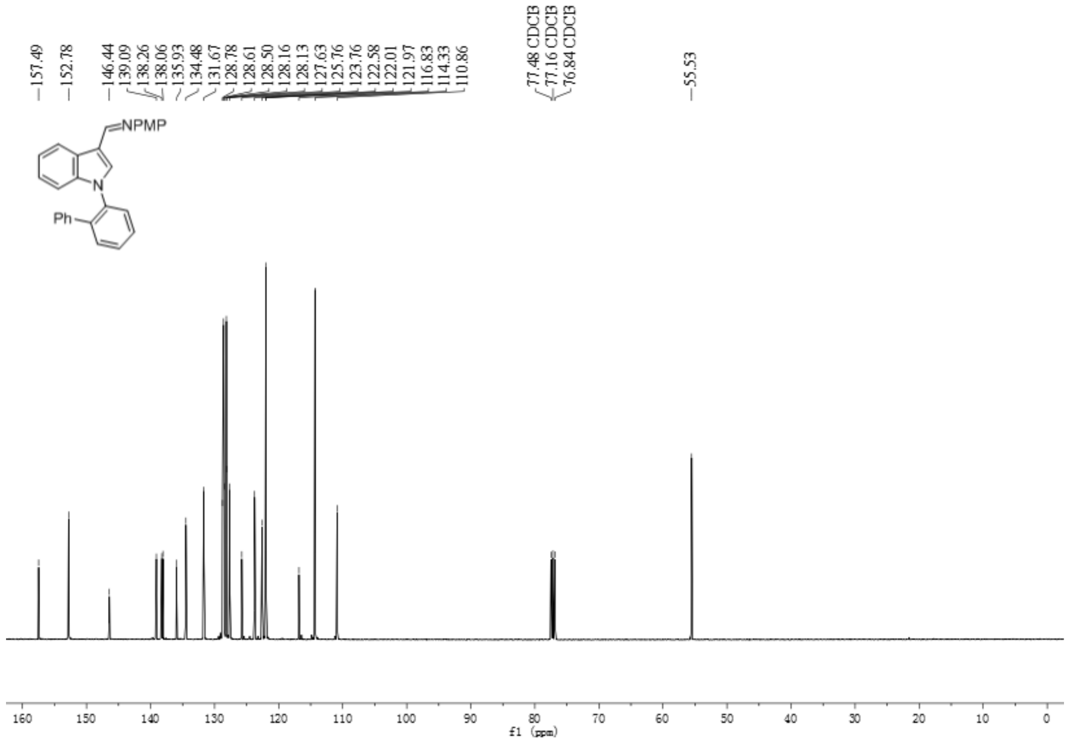

Supplementary Fig. 57. ^{1}H NMR spectrum of **1e**

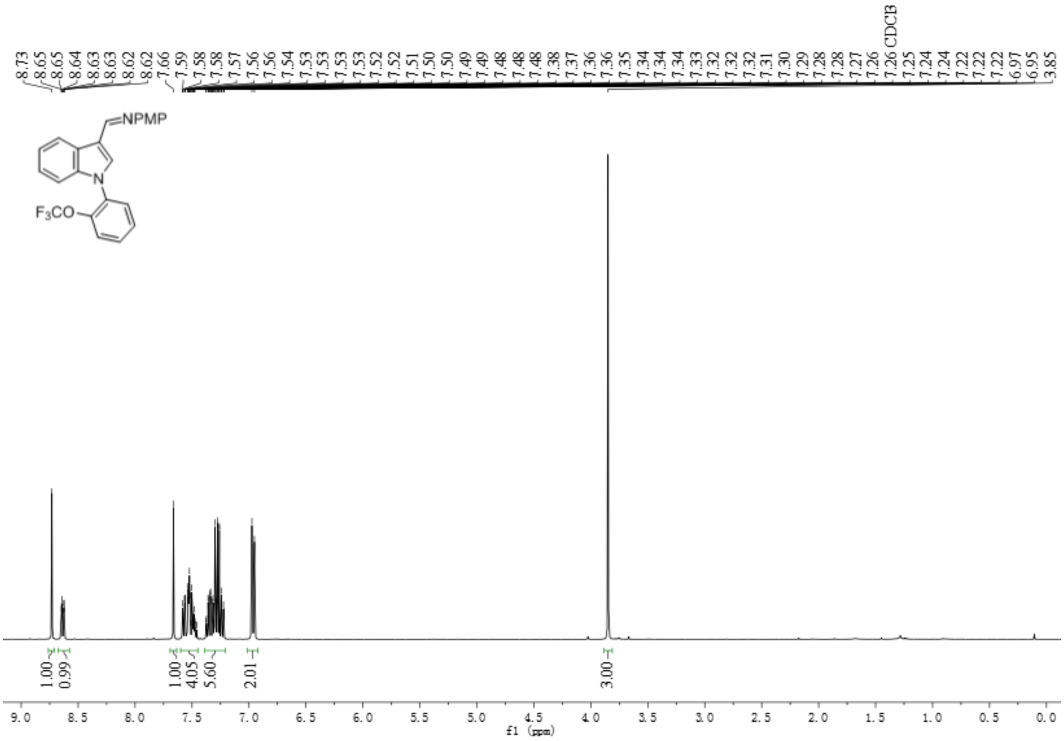

Supplementary Fig. 58. ^{13}C NMR spectrum of **1e**

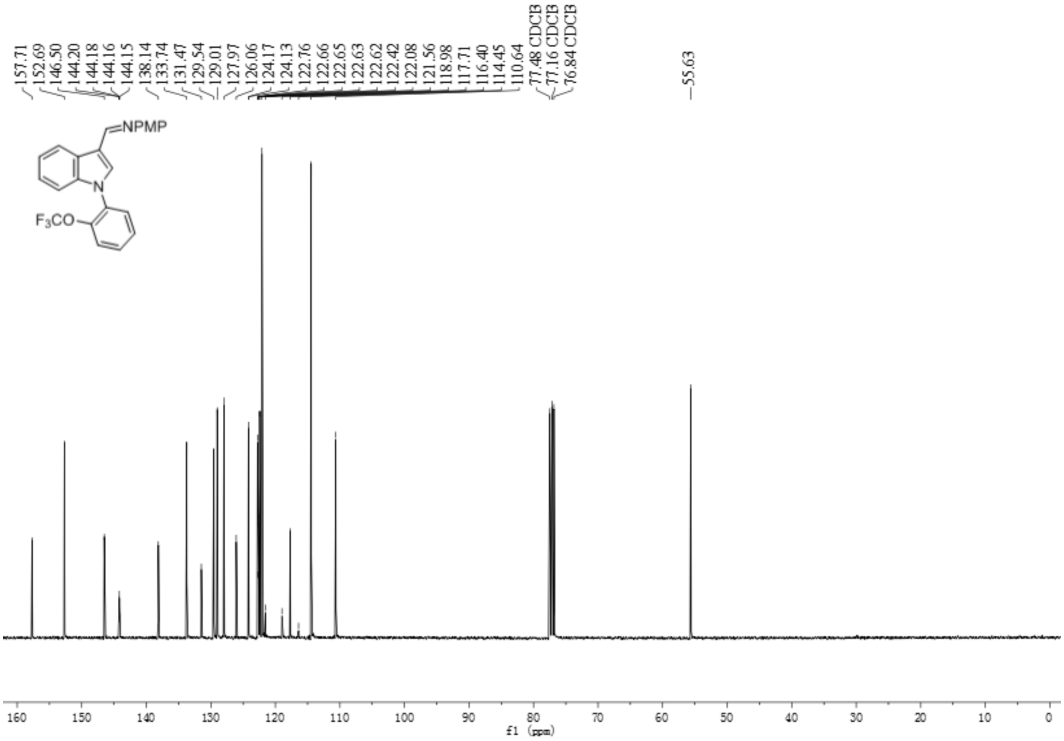

Supplementary Fig. 59. ^{19}F NMR spectrum of **1e**

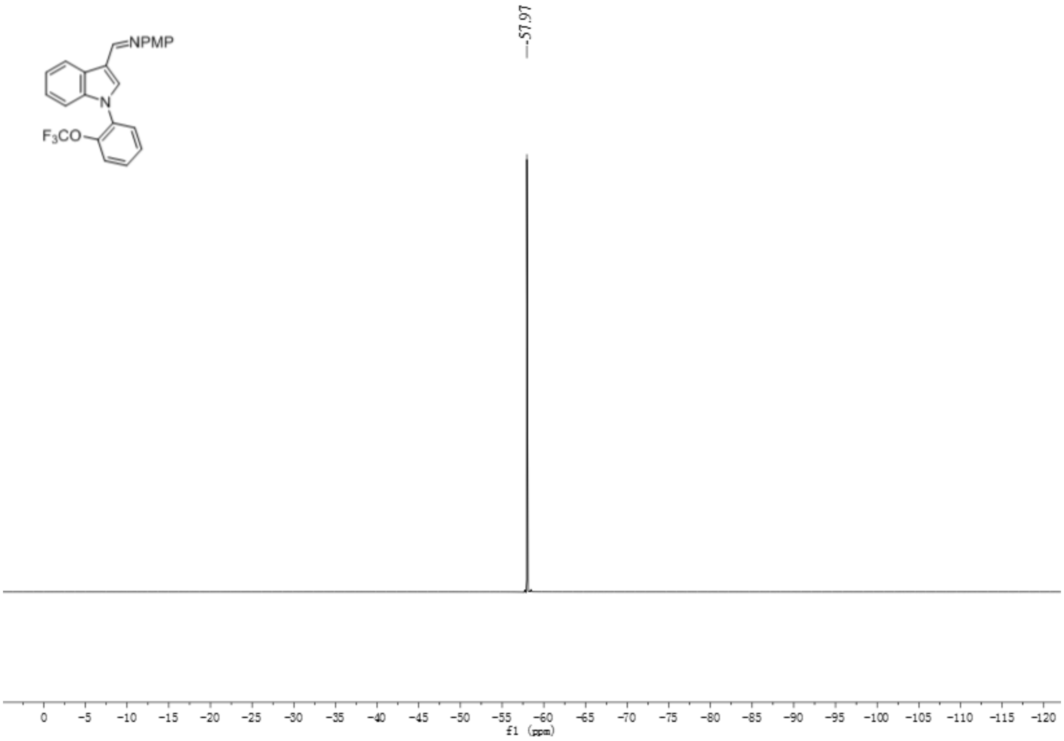

Supplementary Fig. 60. ^{1}H NMR spectrum of **1f**

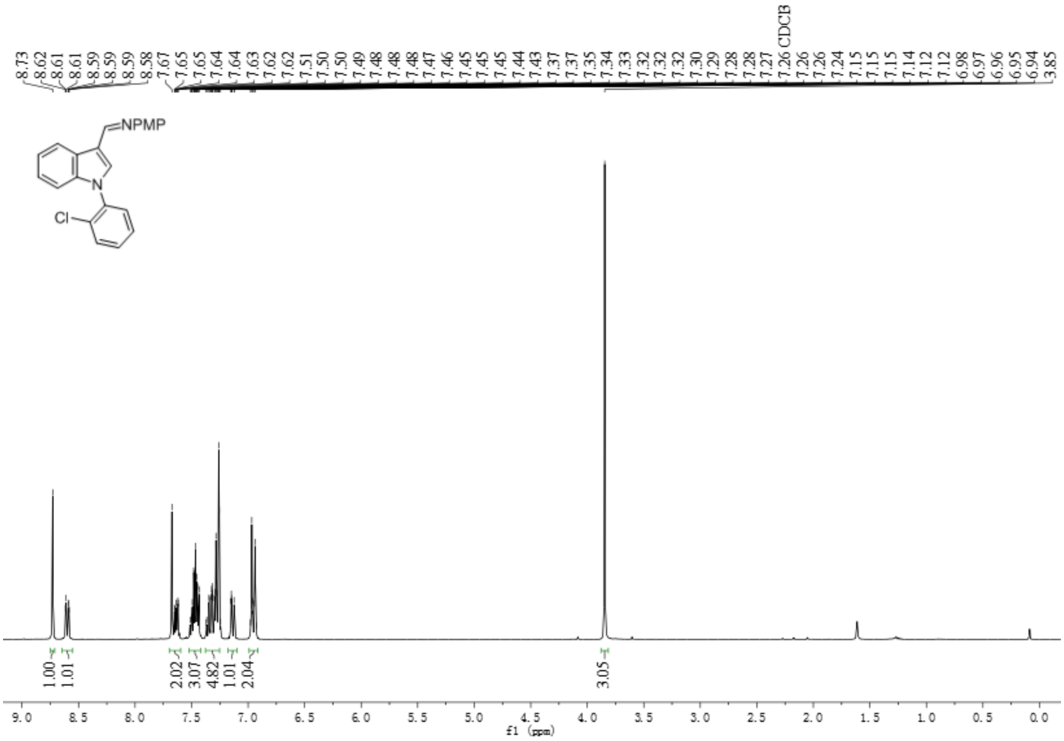

Supplementary Fig. 61. ^{13}C NMR spectrum of **1f**

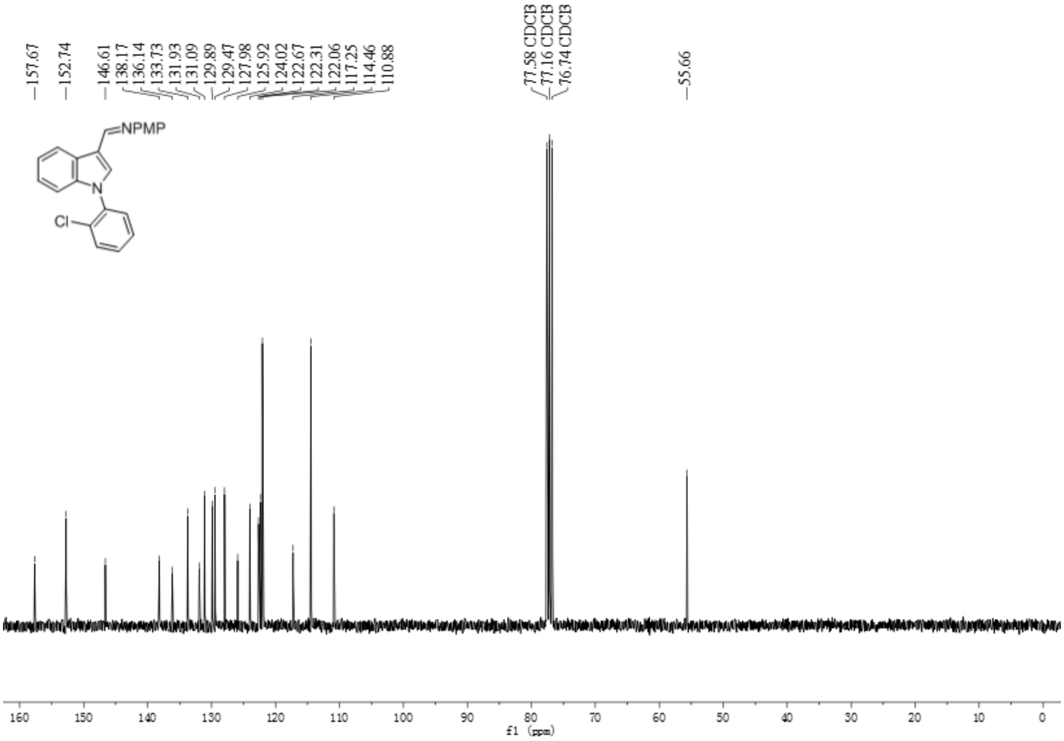

Supplementary Fig. 62. ^{1}H NMR spectrum of **1g**

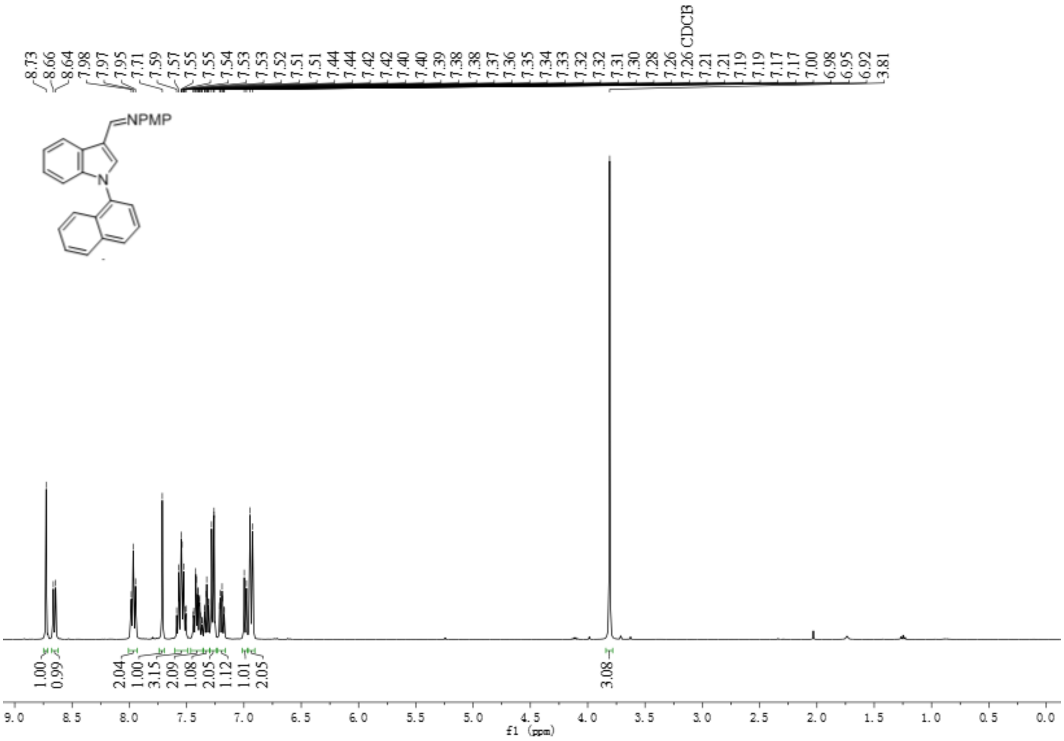

Supplementary Fig. 63. ^{13}C NMR spectrum of **1g**

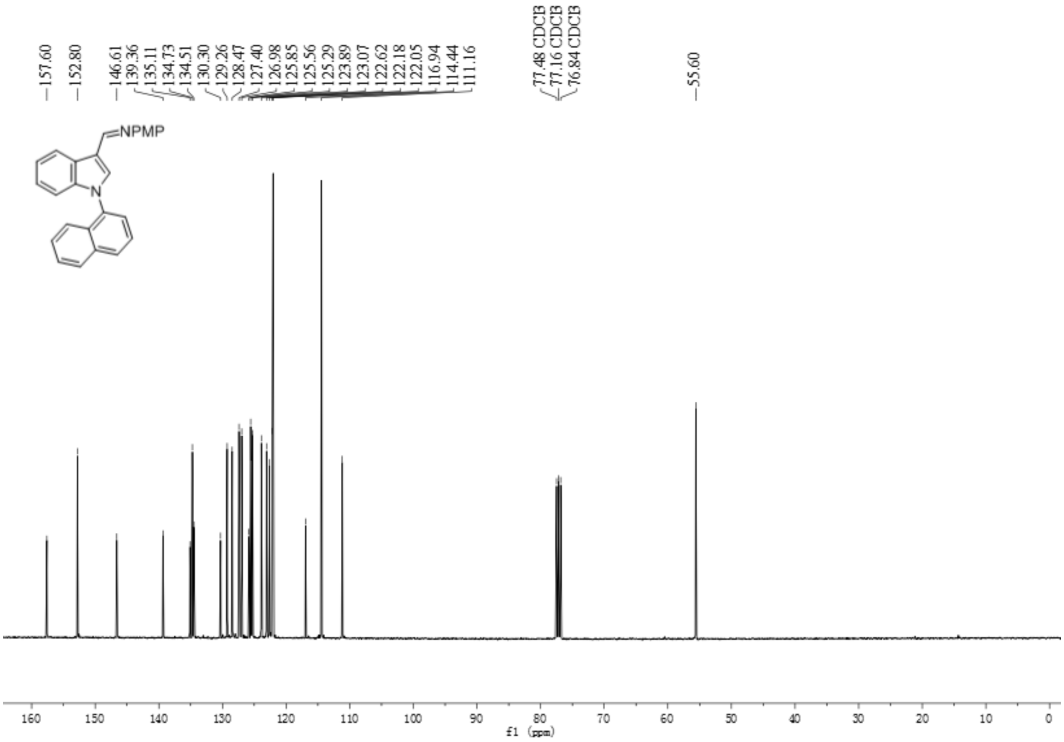

Supplementary Fig. 64. ^{1}H NMR spectrum of **1h**

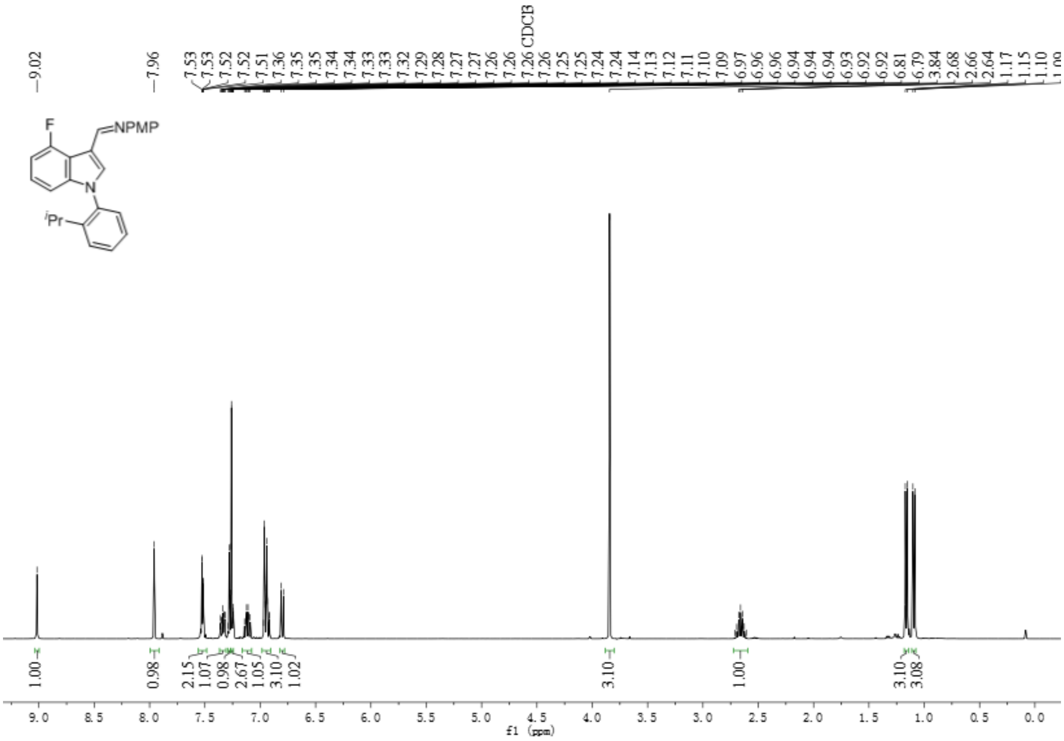

Supplementary Fig. 65. ^{13}C NMR spectrum of **1h**

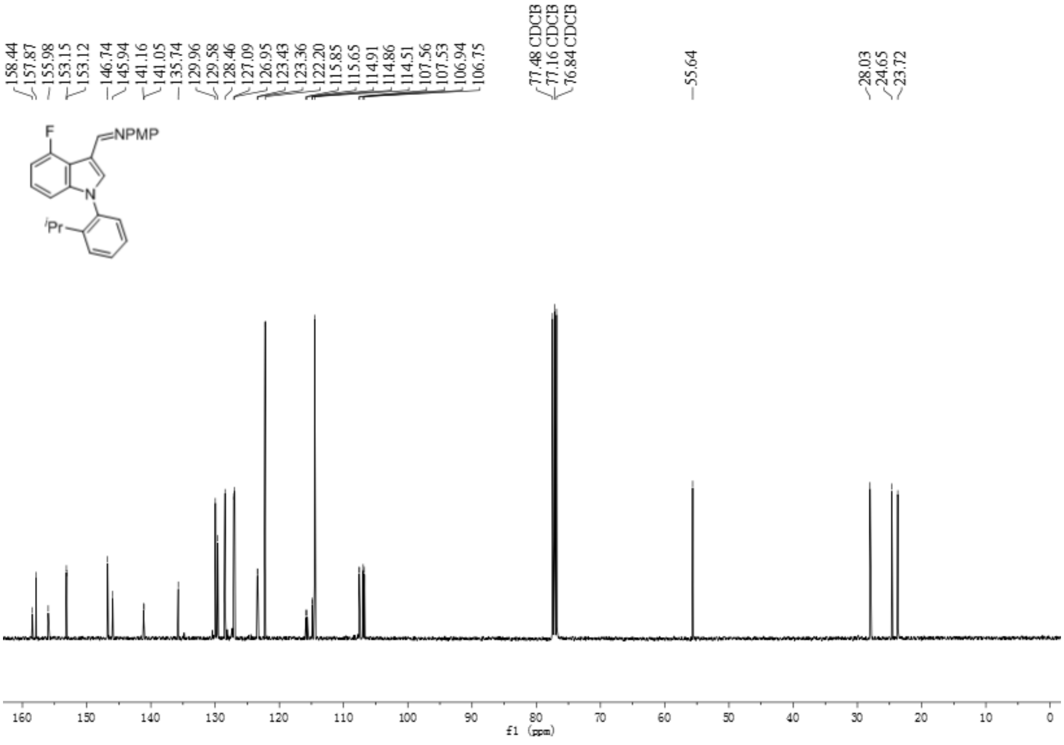

Supplementary Fig. 66. ^{19}F NMR spectrum of **1h**

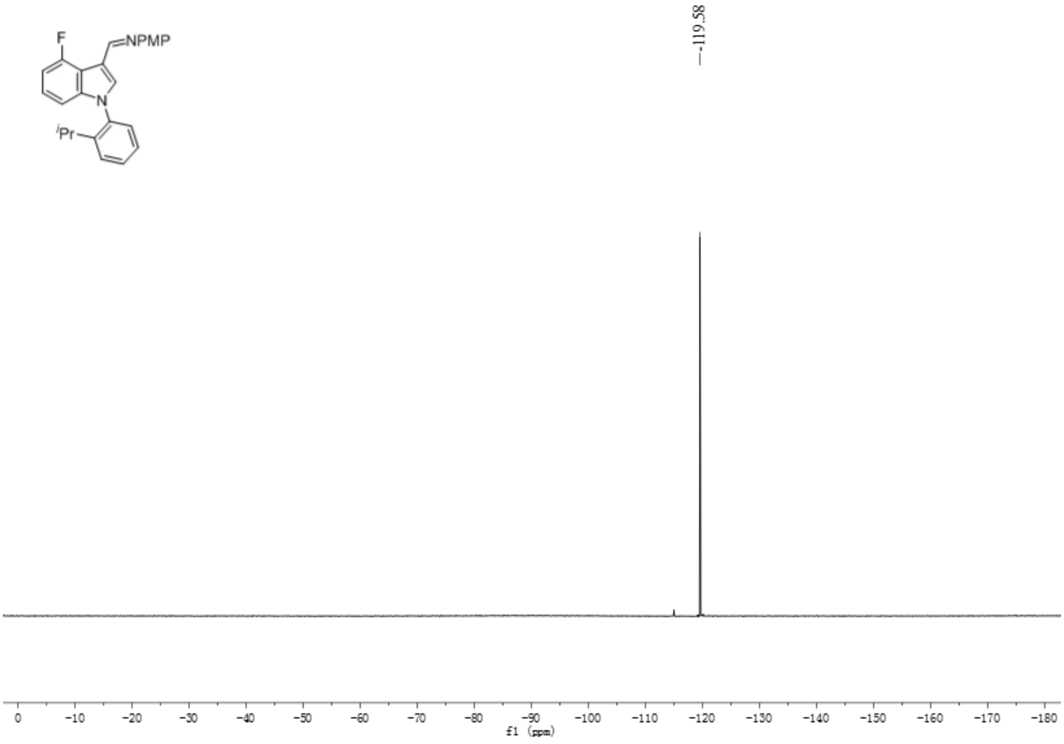

Supplementary Fig. 67. ^{1}H NMR spectrum of **1i**

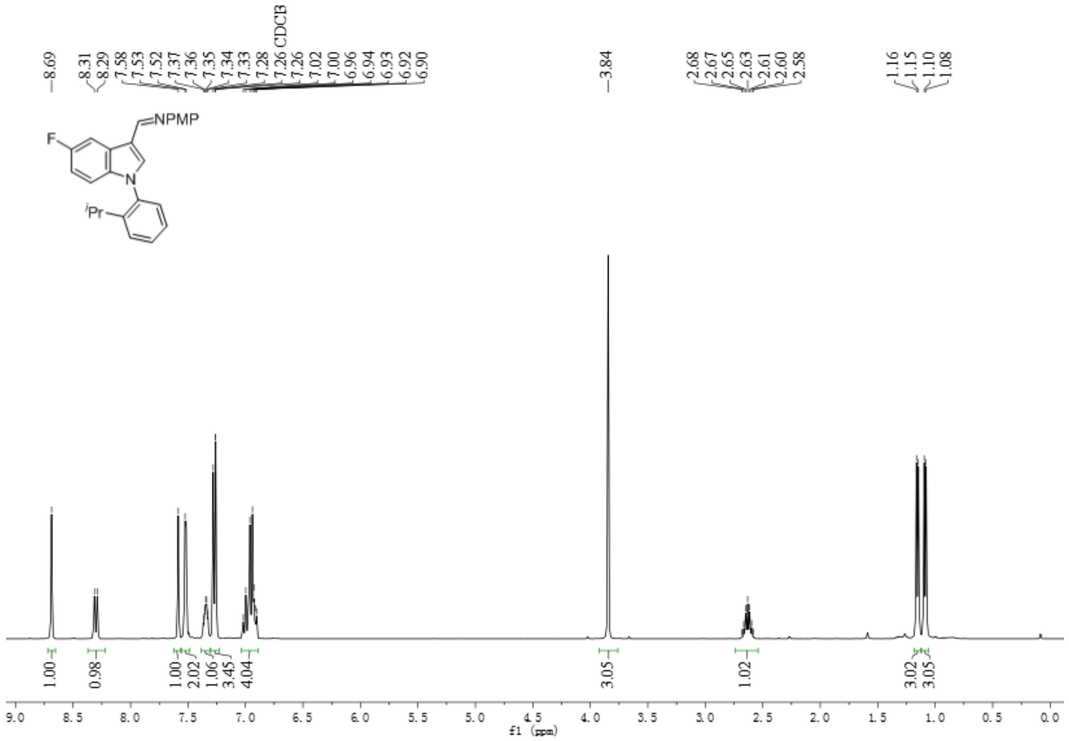

Supplementary Fig. 68. ^{13}C NMR spectrum of **1i**

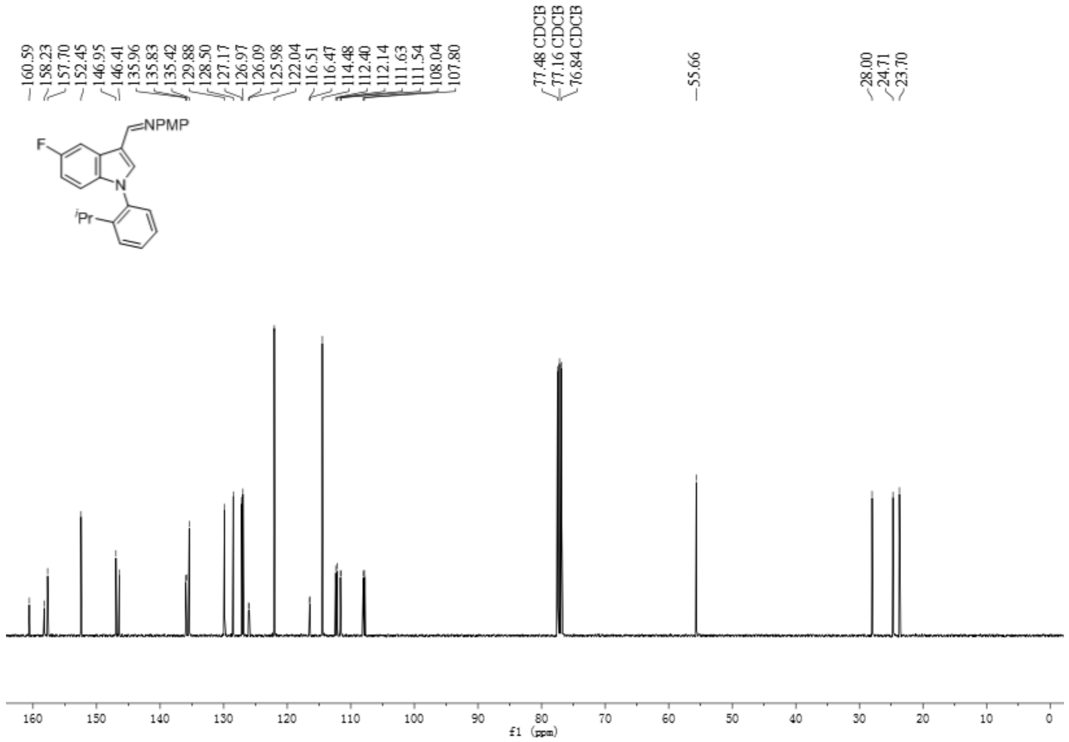

Supplementary Fig. 69. ^{19}F NMR spectrum of **1i**

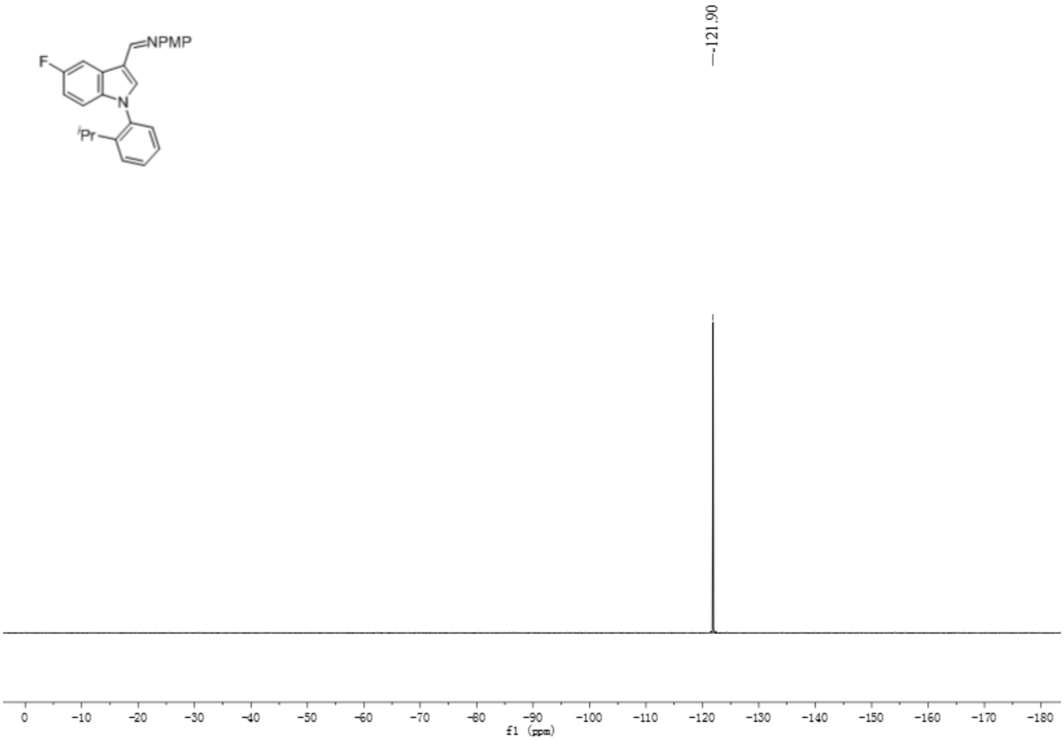

Supplementary Fig. 70. ^{1}H NMR spectrum of **1j**

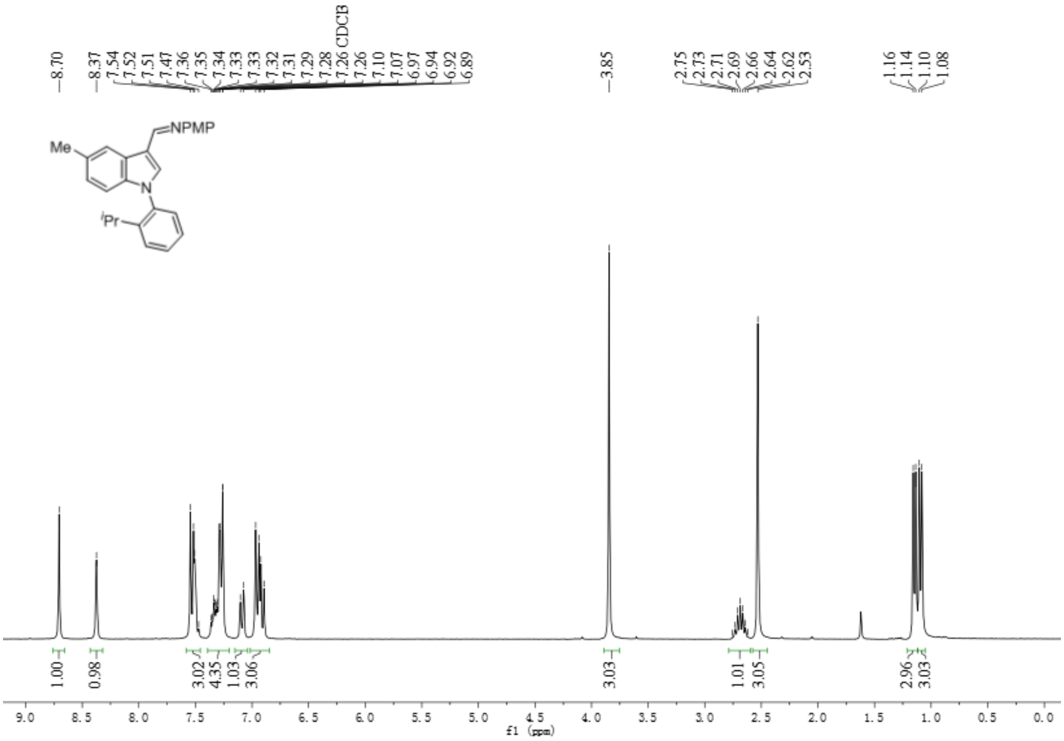

Supplementary Fig. 71. ^{13}C NMR spectrum of **1j**

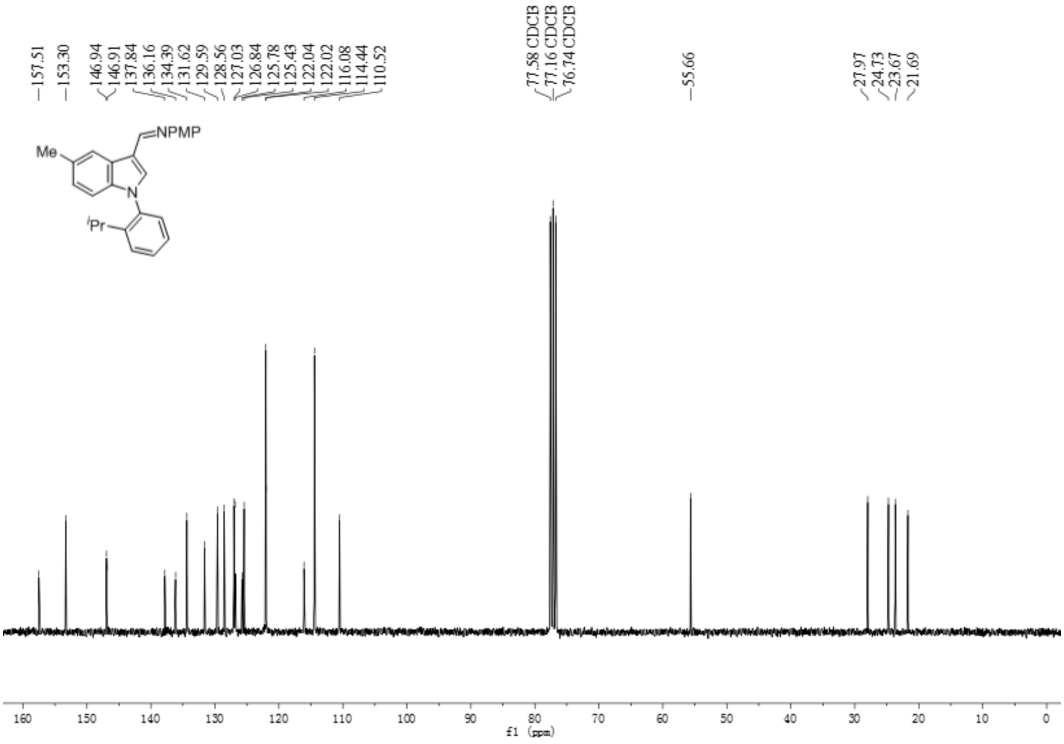

Supplementary Fig. 72. ^{1}H NMR spectrum of **1k**

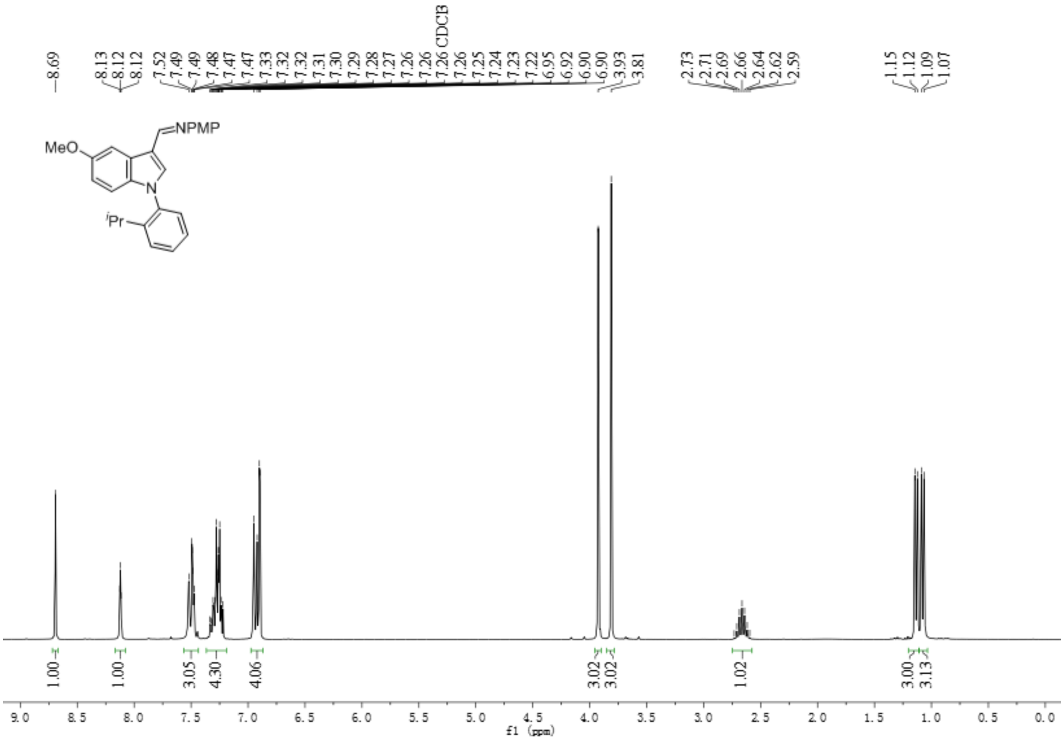

Supplementary Fig. 73. ^{13}C NMR spectrum of **1k**

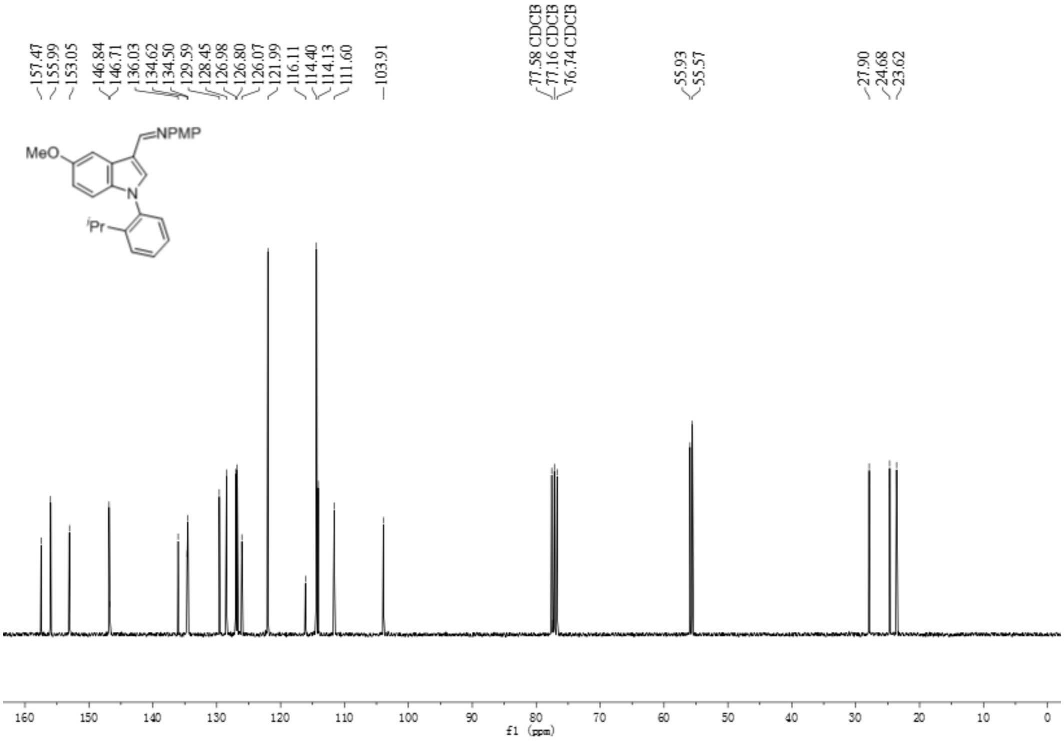

Supplementary Fig. 74. ^{1}H NMR spectrum of **1l**

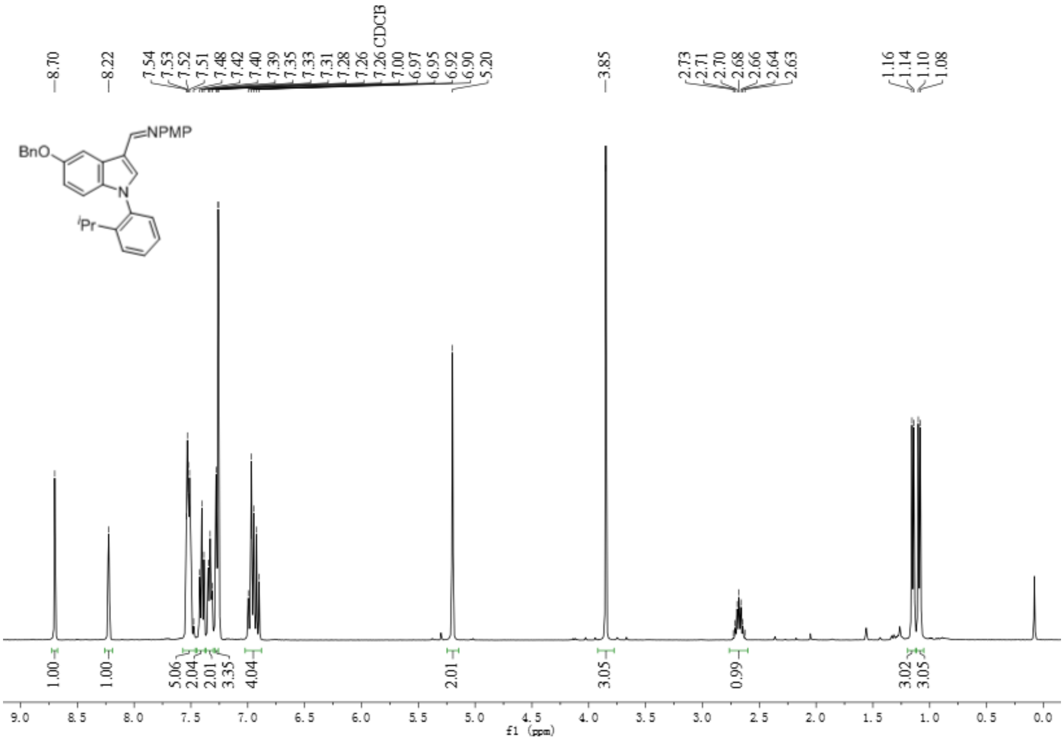

Supplementary Fig. 75. ^{13}C NMR spectrum of **1l**

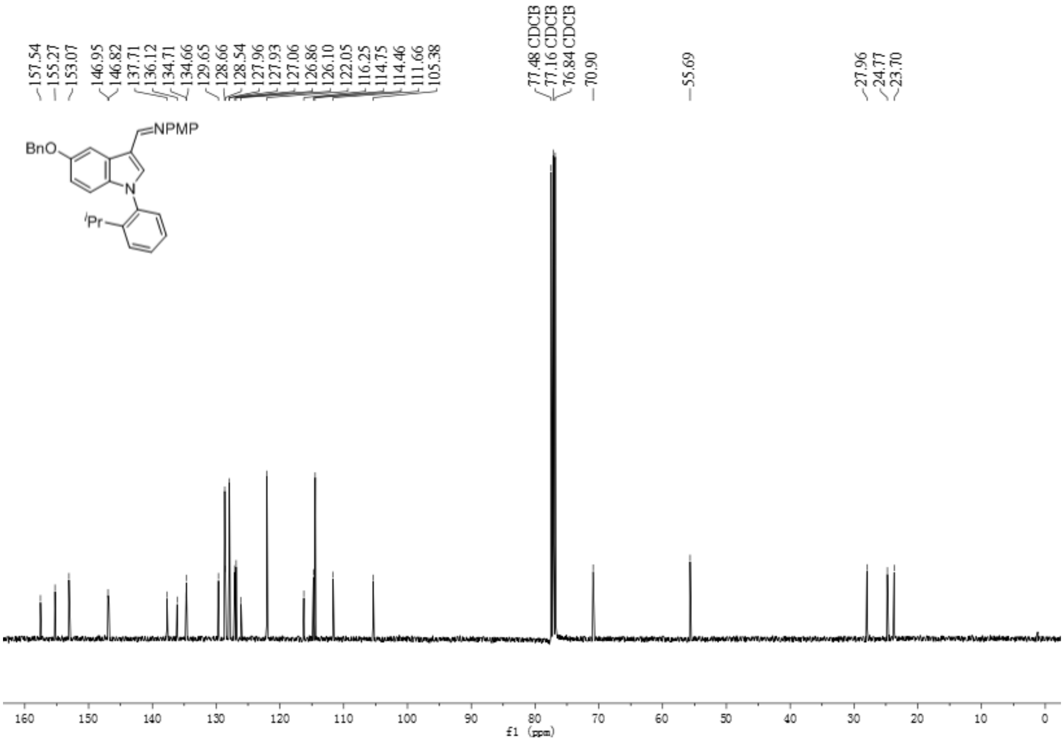

Supplementary Fig. 76. ^{1}H NMR spectrum of **1m**

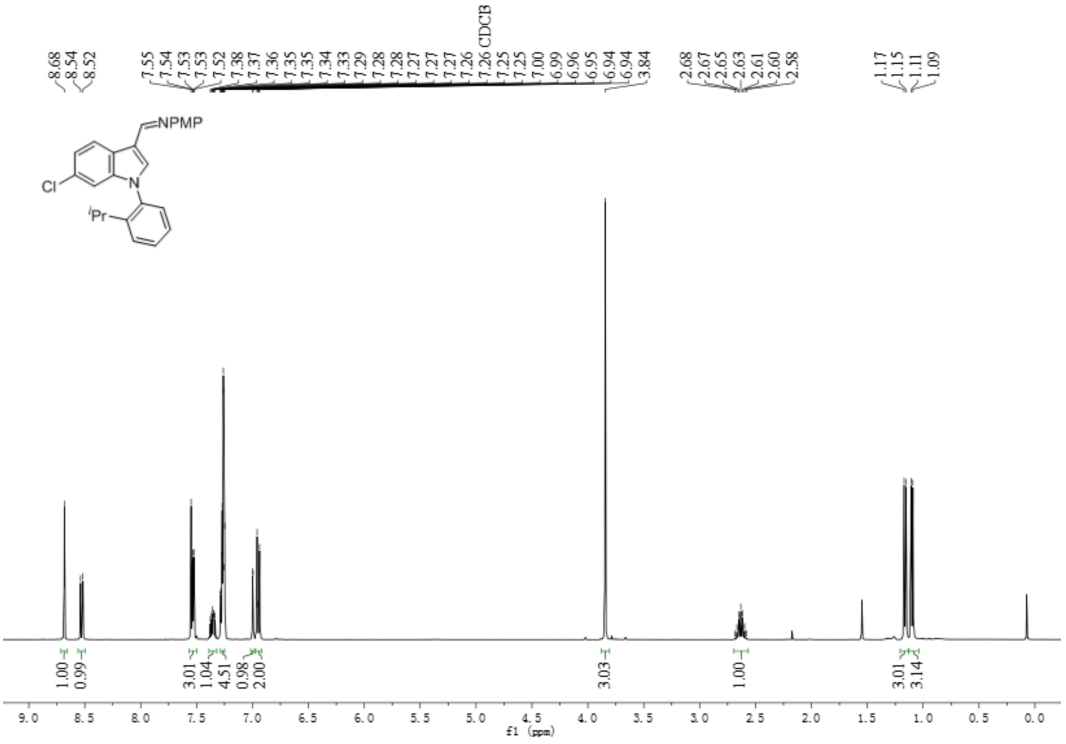

Supplementary Fig. 77. ^{13}C NMR spectrum of **1m**

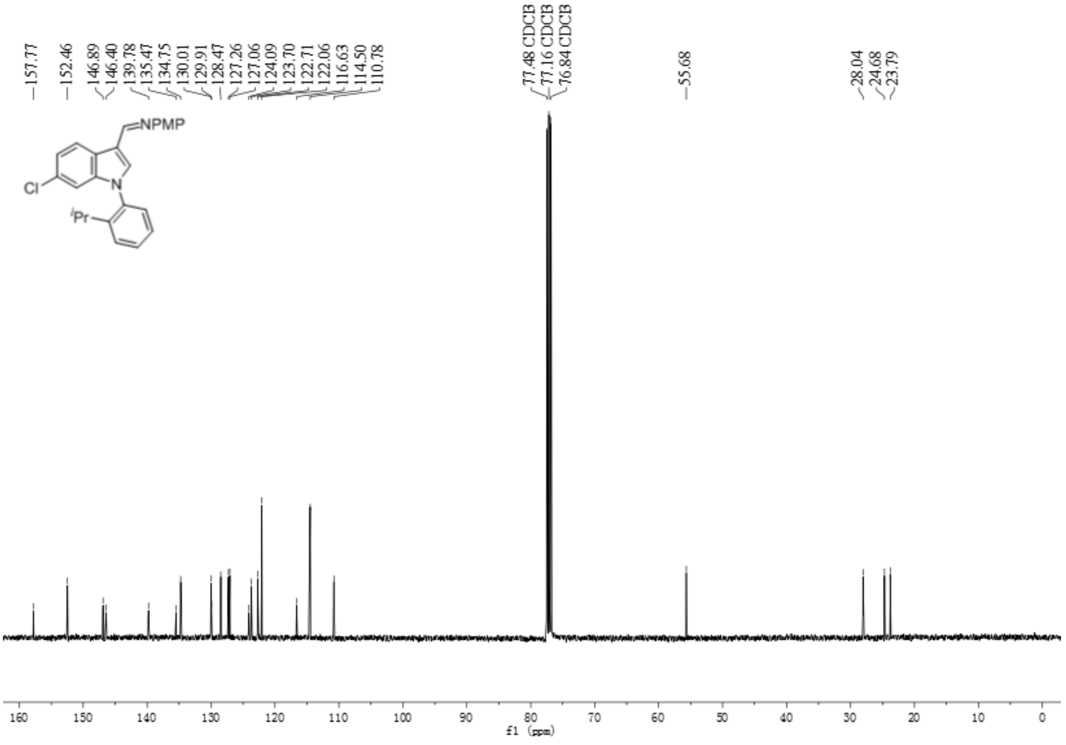

Supplementary Fig. 78. ^{1}H NMR spectrum of **1n**

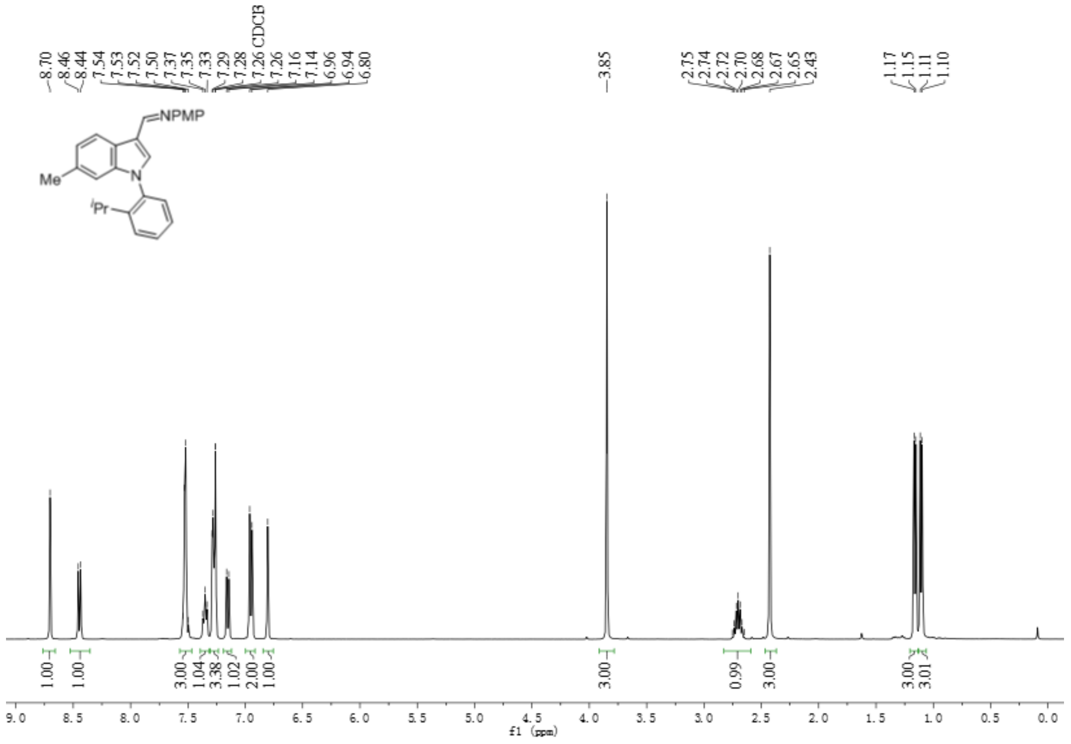

Supplementary Fig. 79. ^{13}C NMR spectrum of **1n**

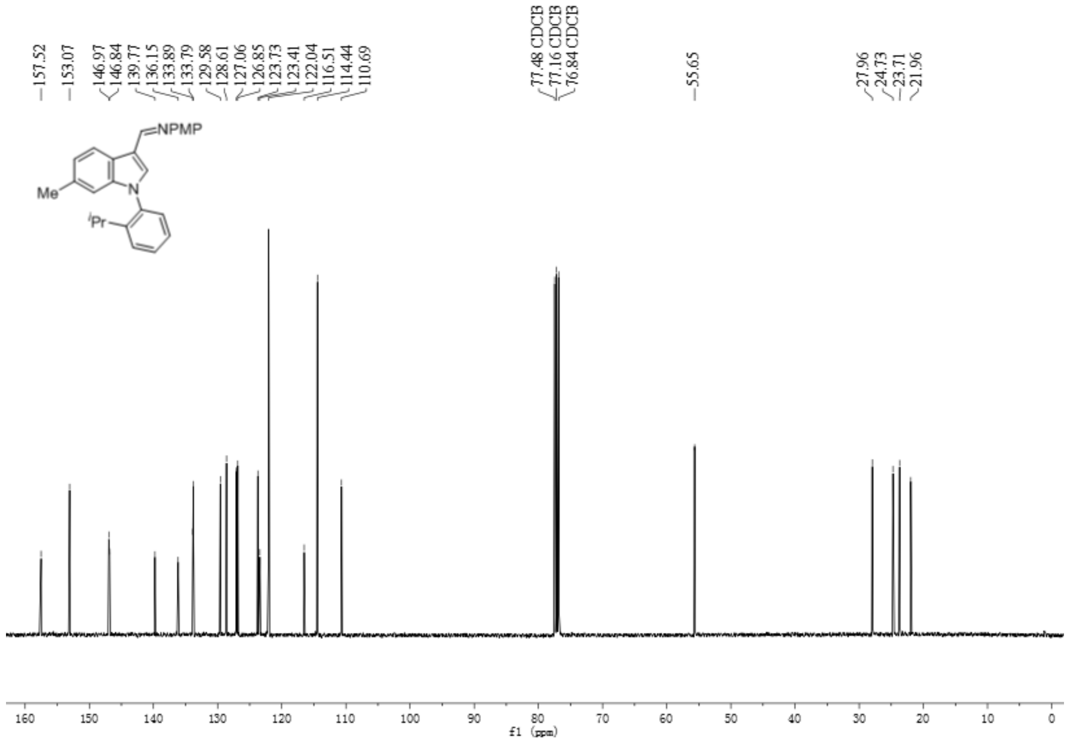

Supplementary Fig. 80. ^{1}H NMR spectrum of **1o**

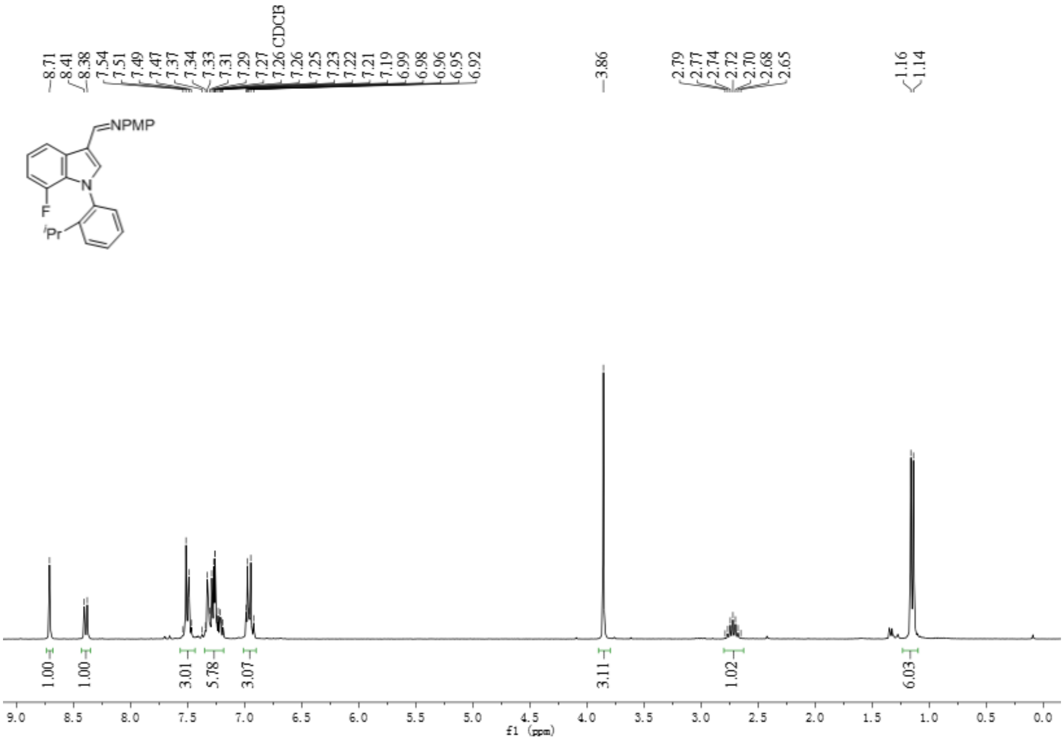

Supplementary Fig. 81. ^{13}C NMR spectrum of **1o**

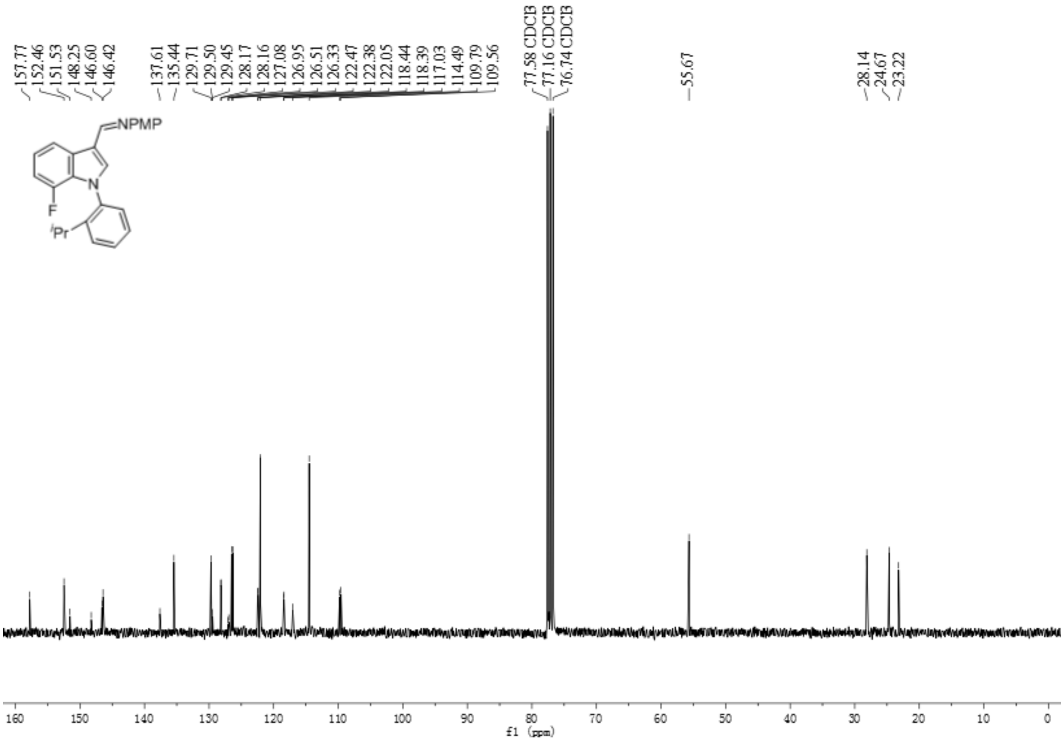

Supplementary Fig. 82. ^{19}F NMR spectrum of **1o**

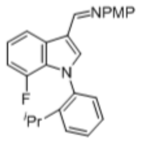

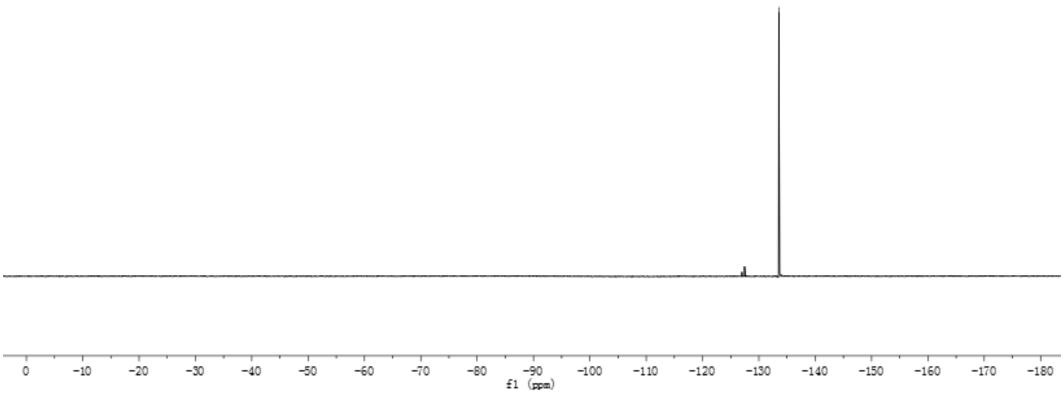

Supplementary Fig. 83. ^{1}H NMR spectrum of **1p**

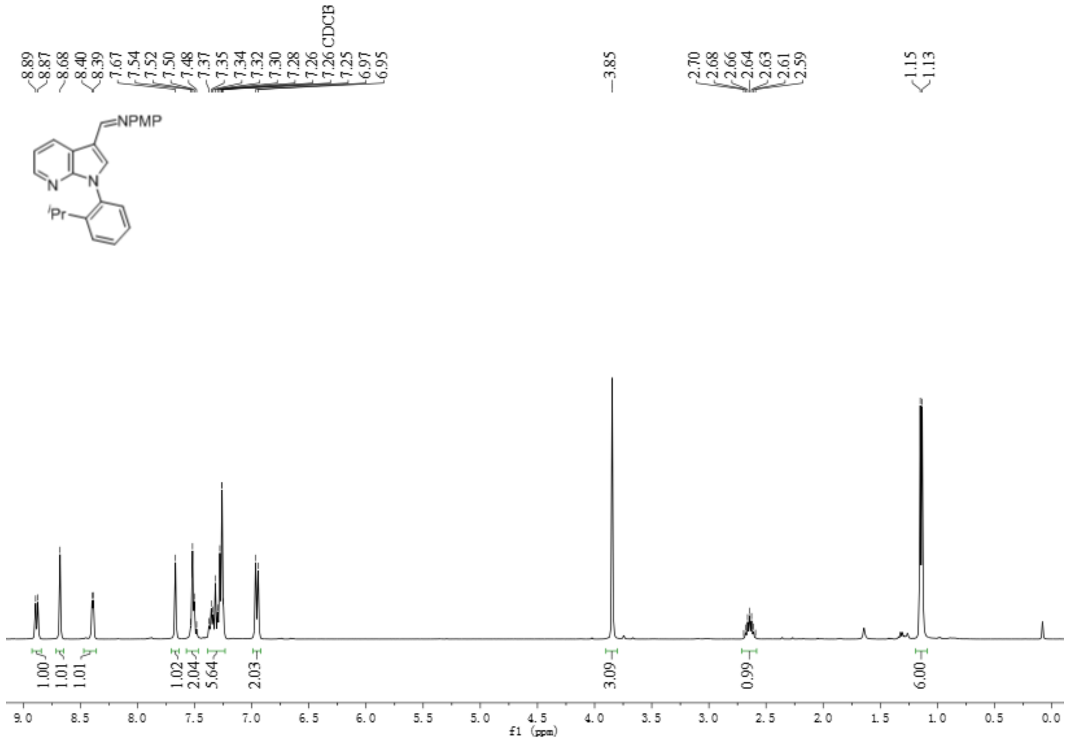

Supplementary Fig. 84. ^{13}C NMR spectrum of **1p**

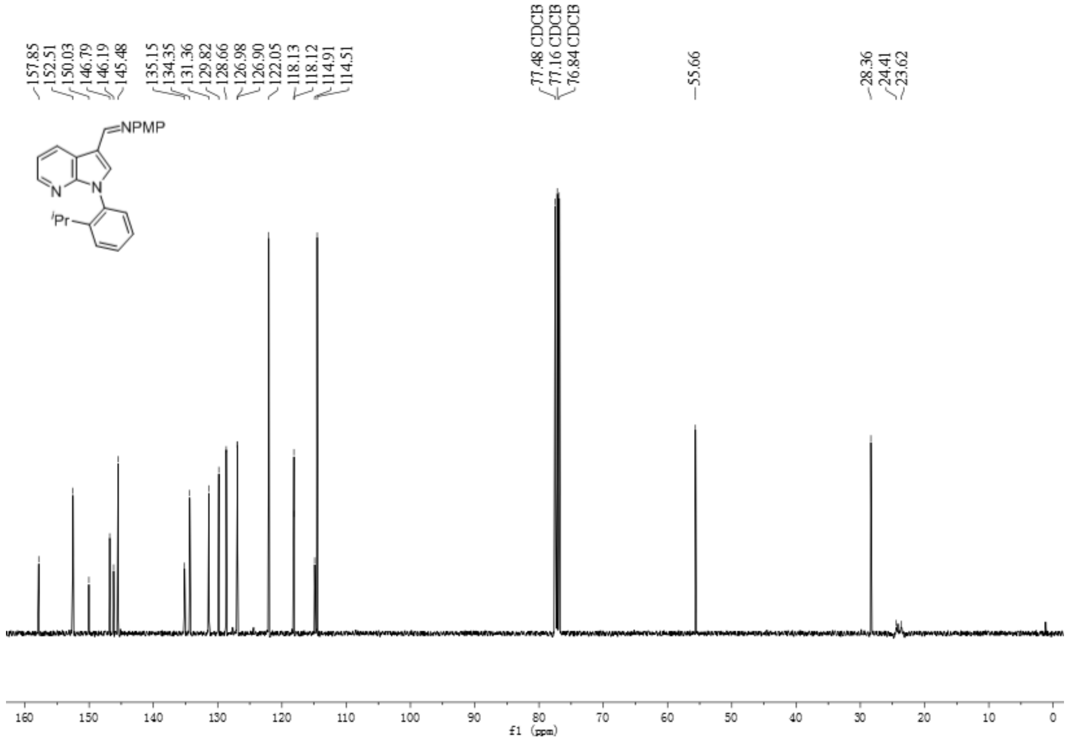

Supplementary Fig. 85. ^{1}H NMR spectrum of **3**

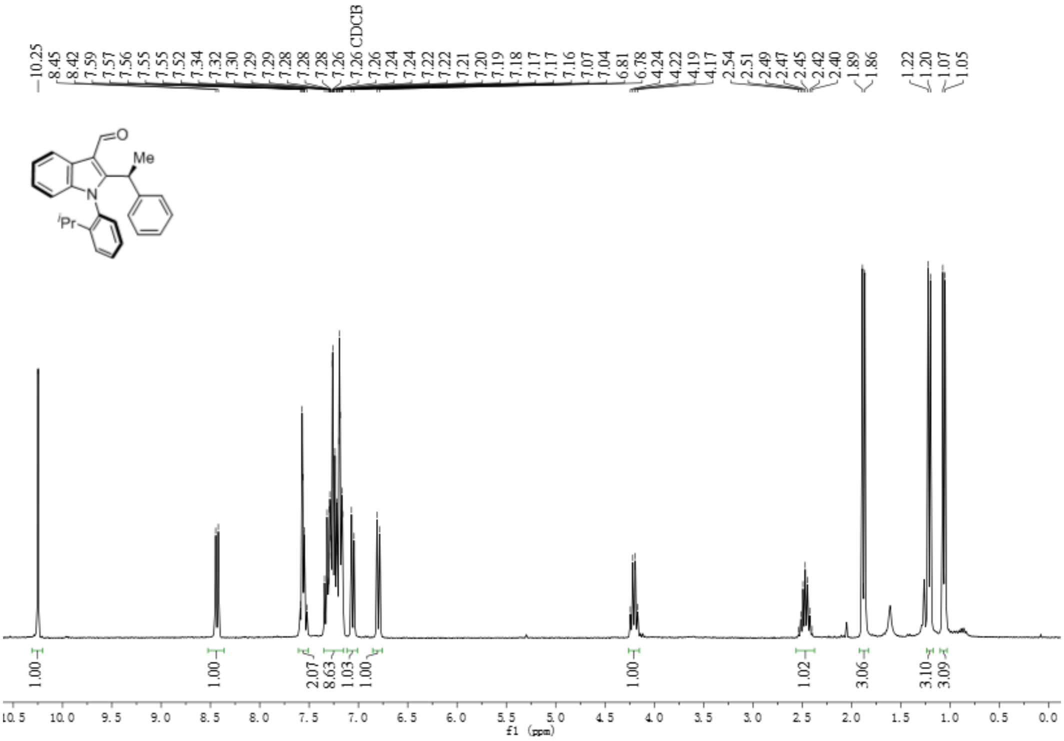

Supplementary Fig. 86. ^{13}C NMR spectrum of **3**

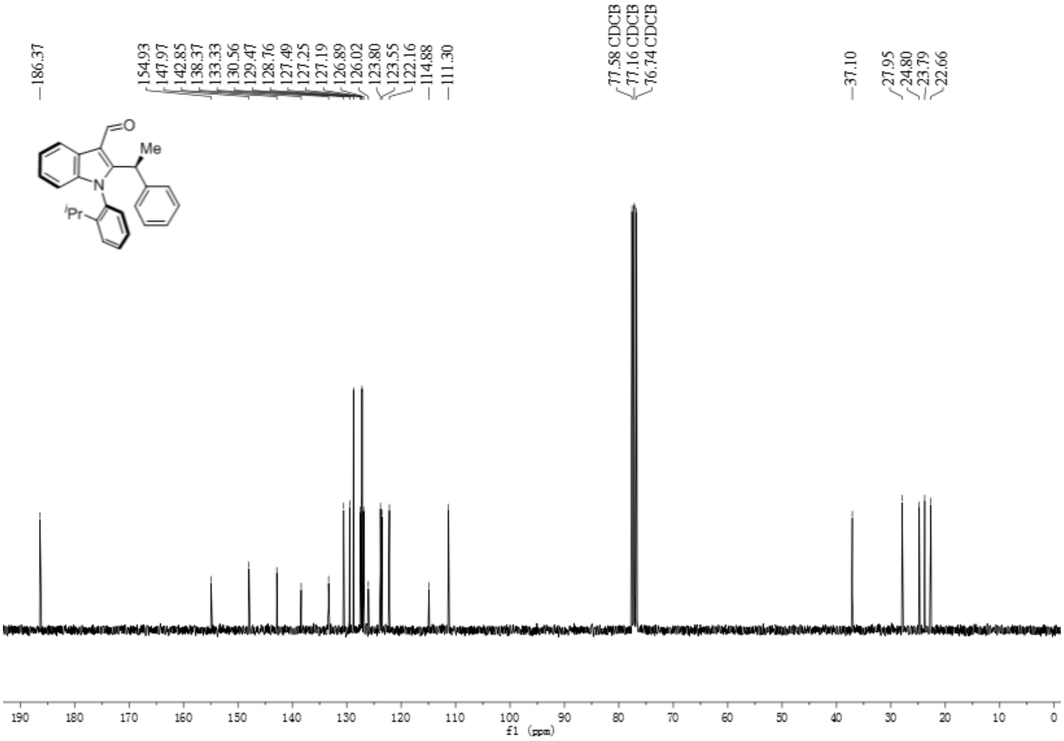

Supplementary Fig. 87. ^{1}H NMR spectrum of **4**

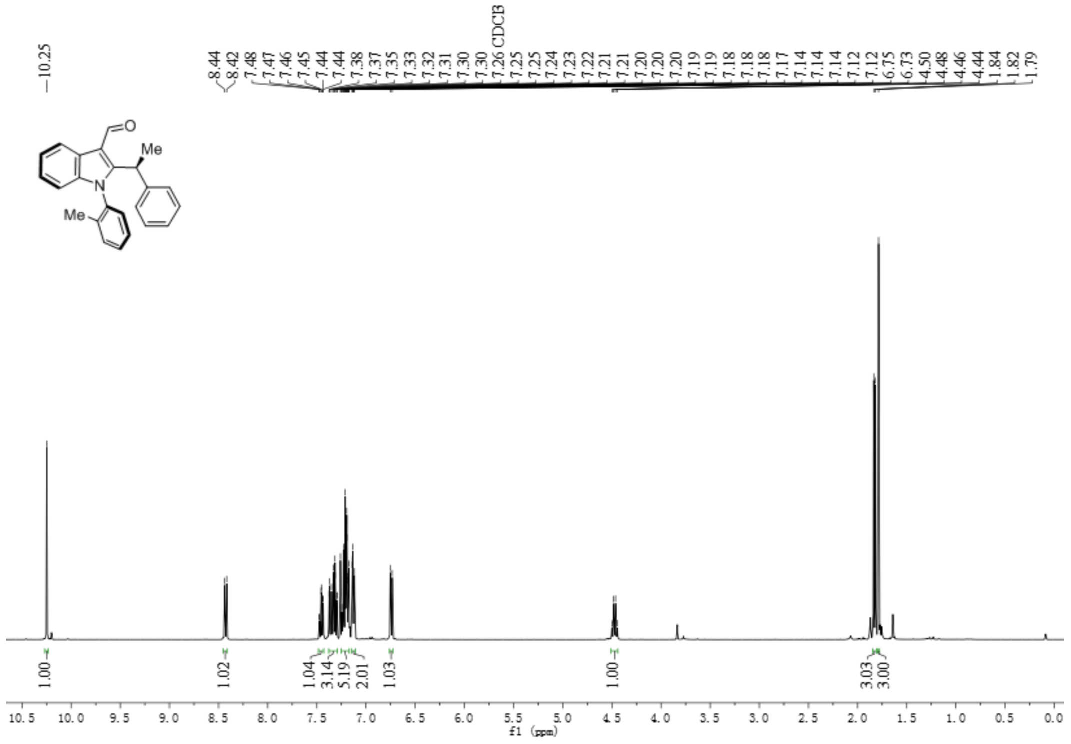

Supplementary Fig. 88. ^{13}C NMR spectrum of **4**

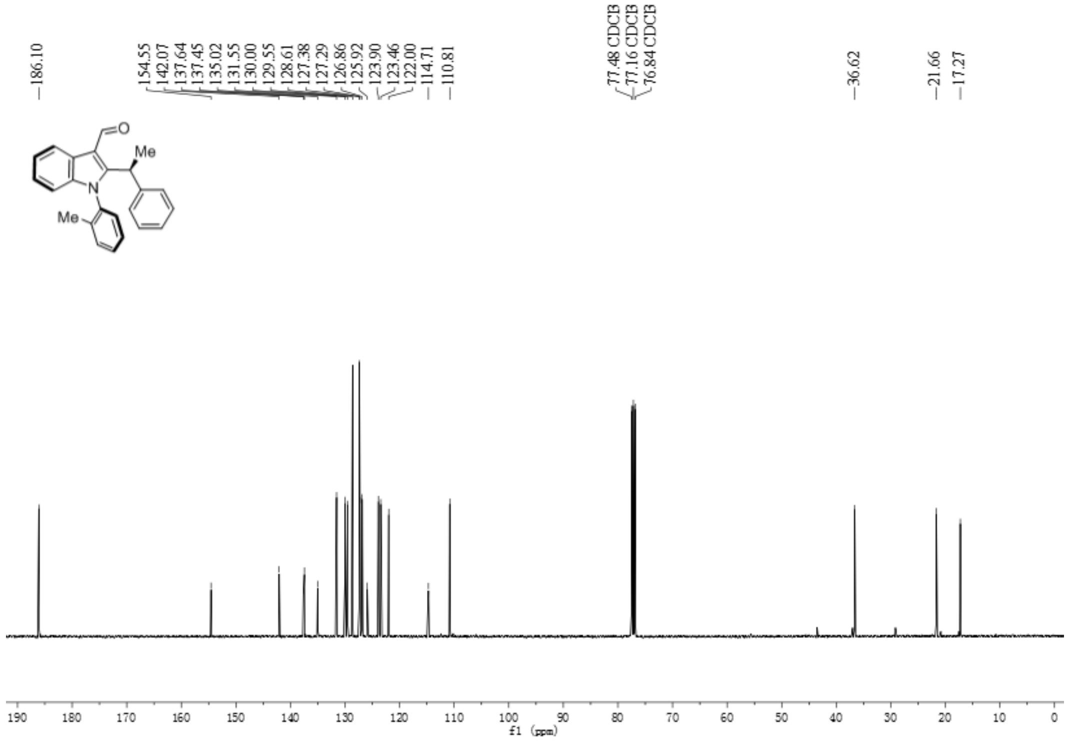

Supplementary Fig. 89. ^{1}H NMR spectrum of **5**

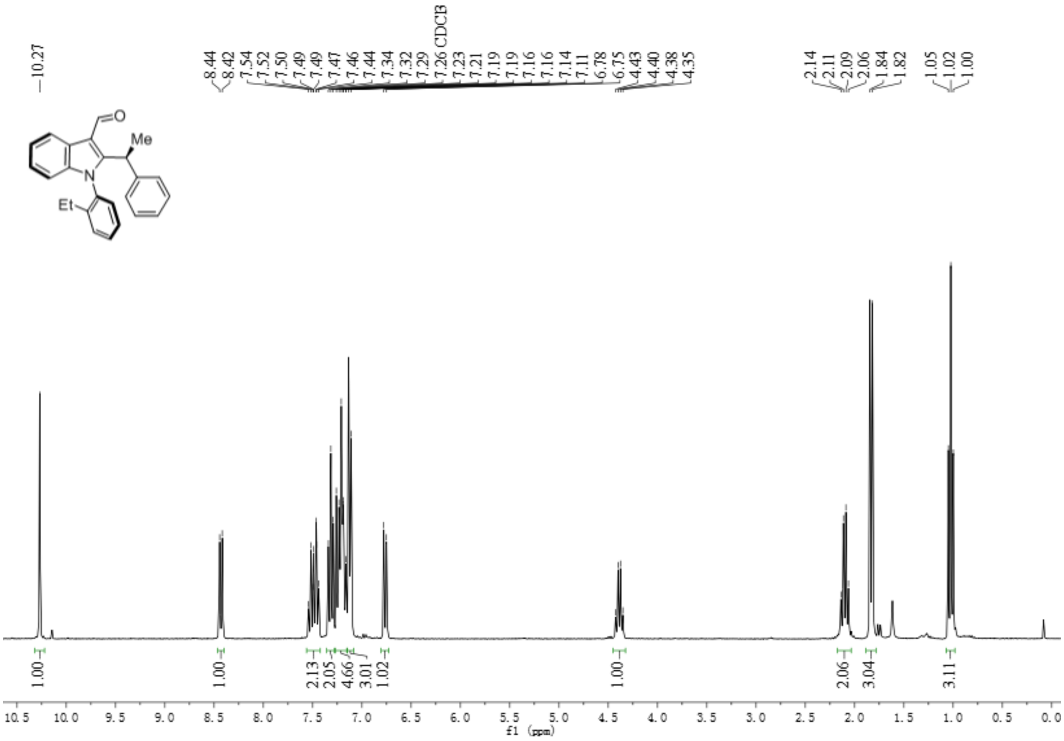

Supplementary Fig. 90. ^{13}C NMR spectrum of **5**

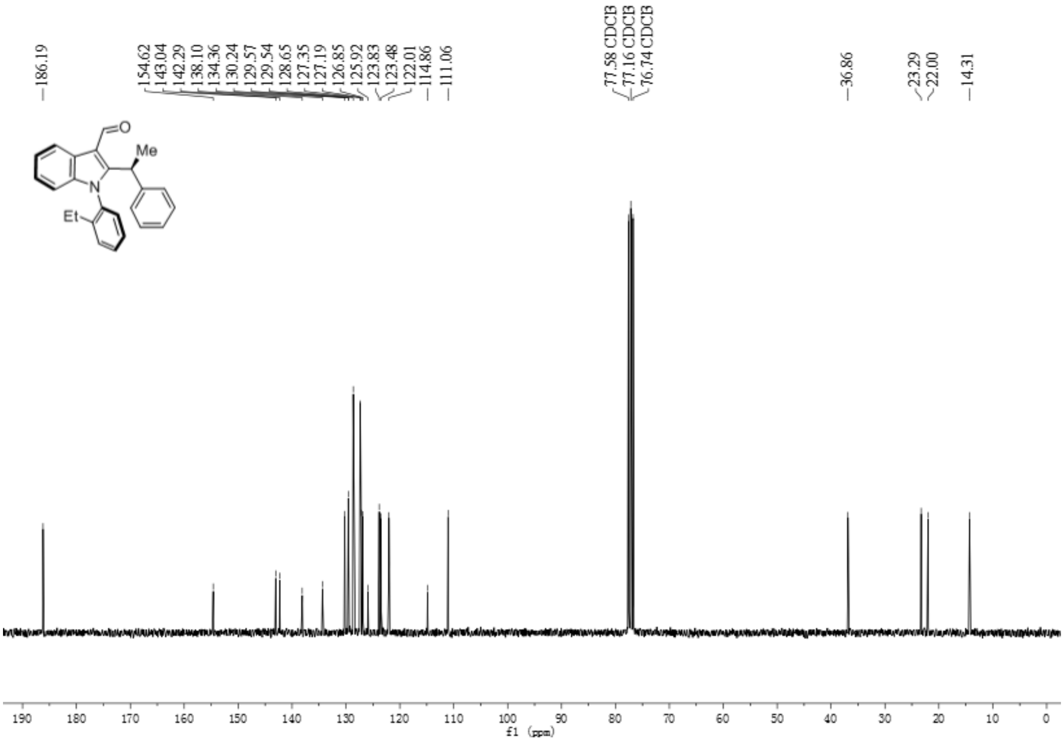

Supplementary Fig. 91. ^{1}H NMR spectrum of **6**

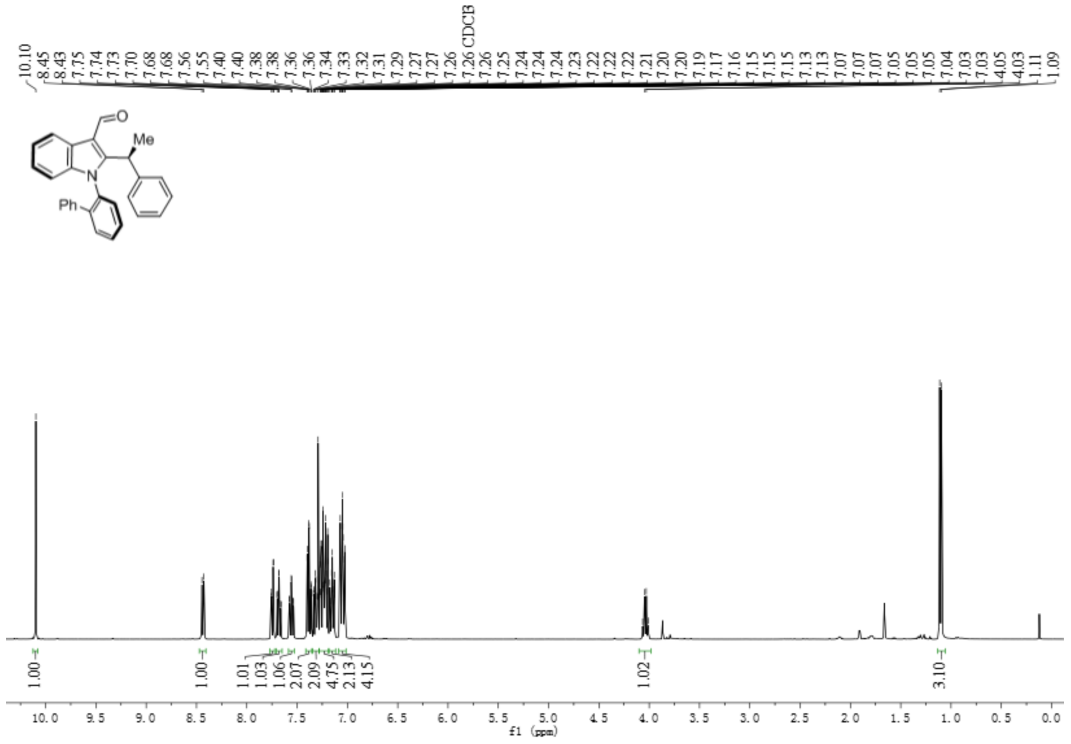

Supplementary Fig. 92. ^{13}C NMR spectrum of **6**

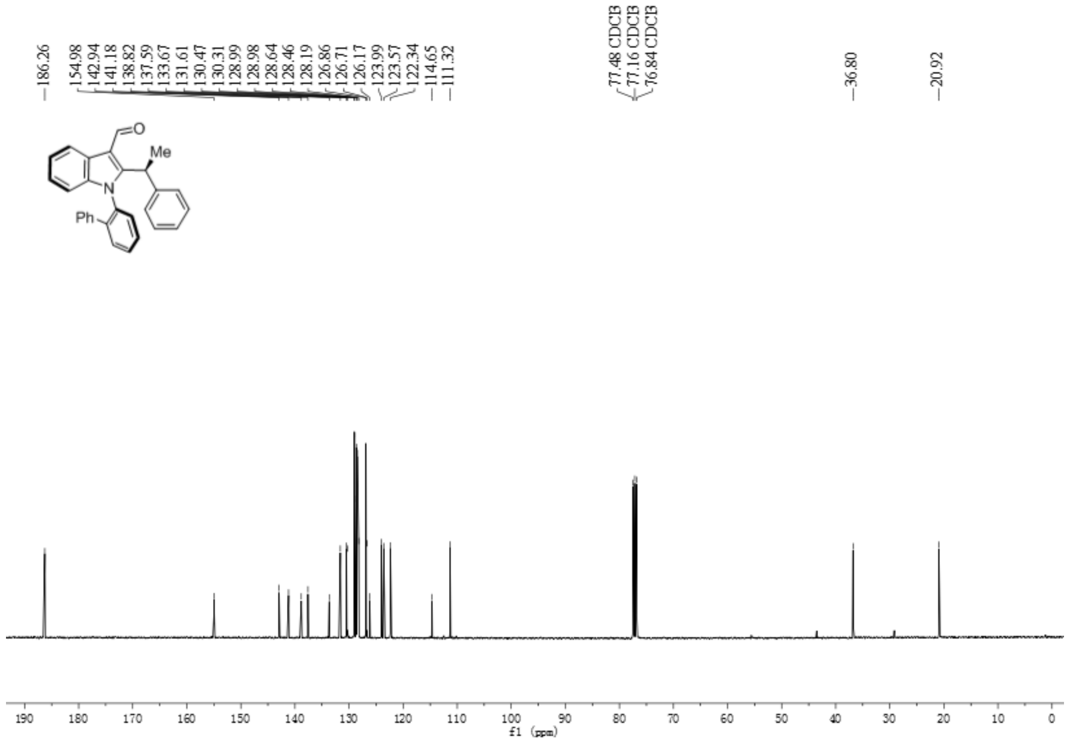

Supplementary Fig. 93. ^{1}H NMR spectrum of **7**

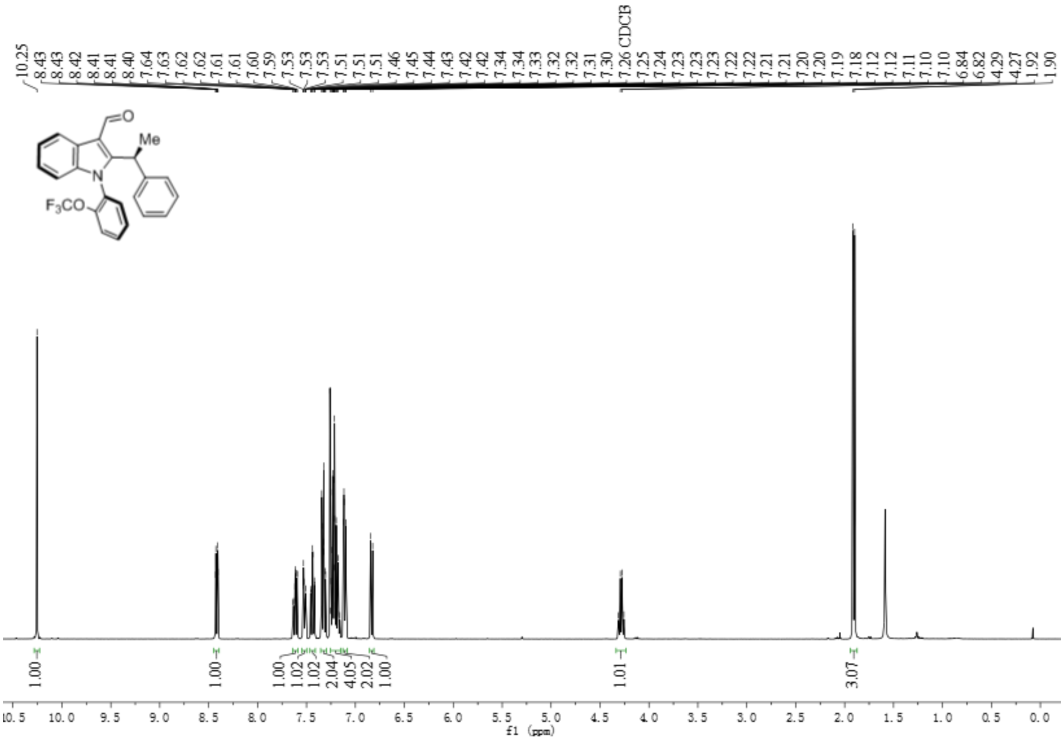

Supplementary Fig. 94. ^{13}C NMR spectrum of **7**

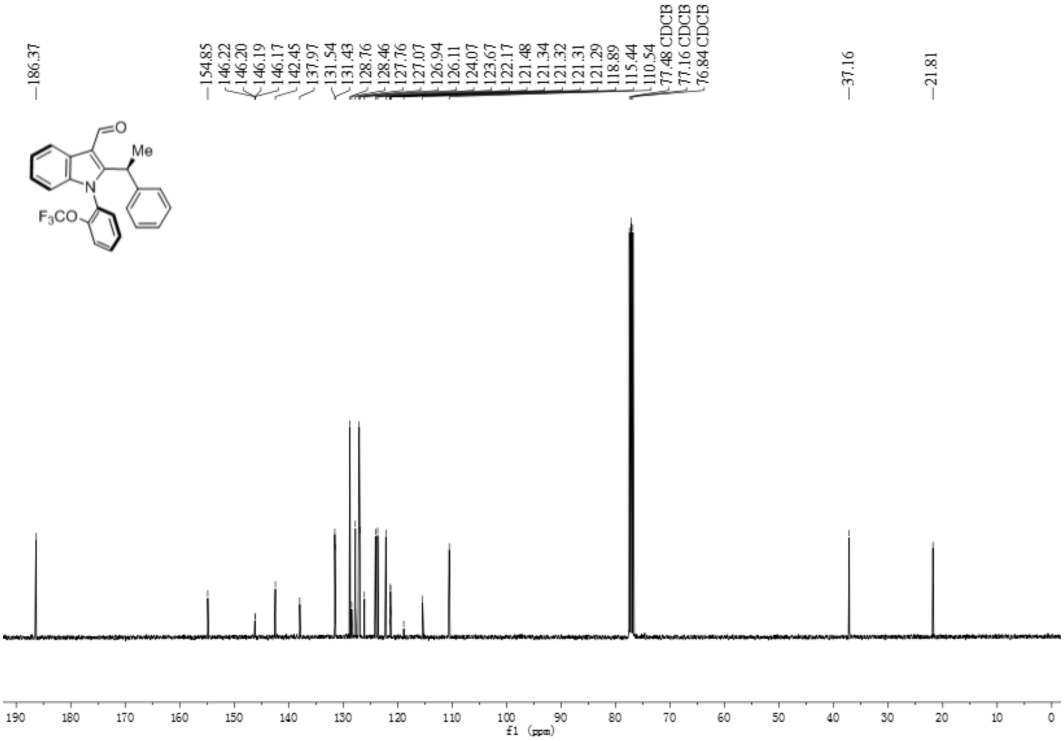

Supplementary Fig. 95. ^{19}F NMR spectrum of **7**

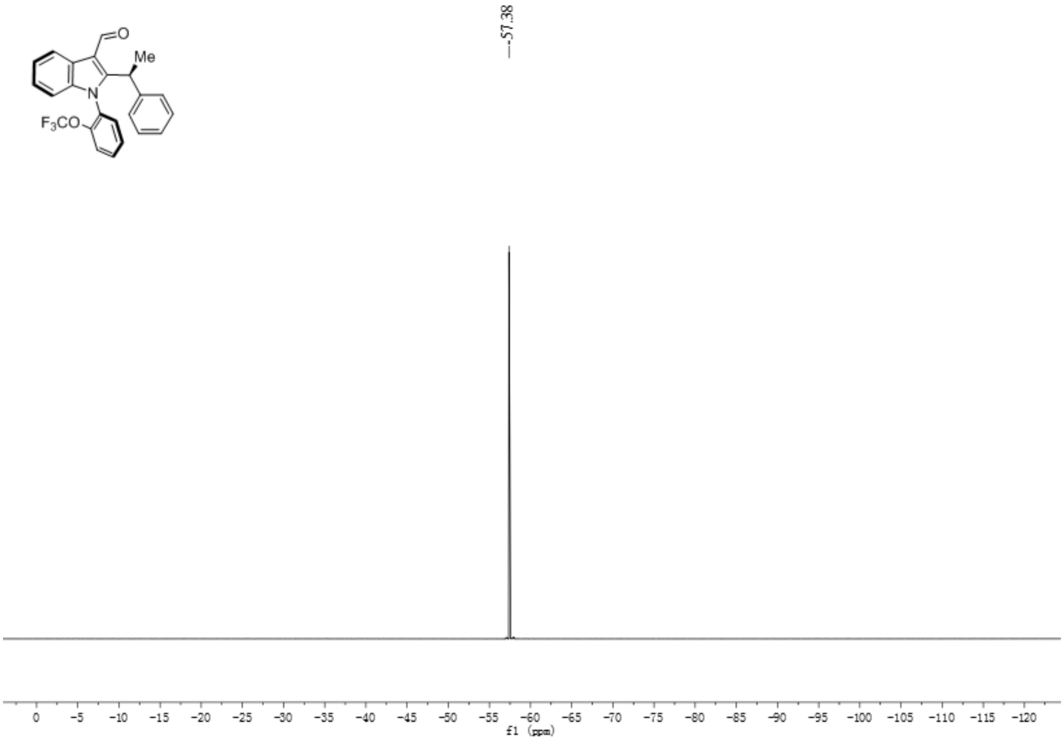

Supplementary Fig. 96. ^{1}H NMR spectrum of **8**

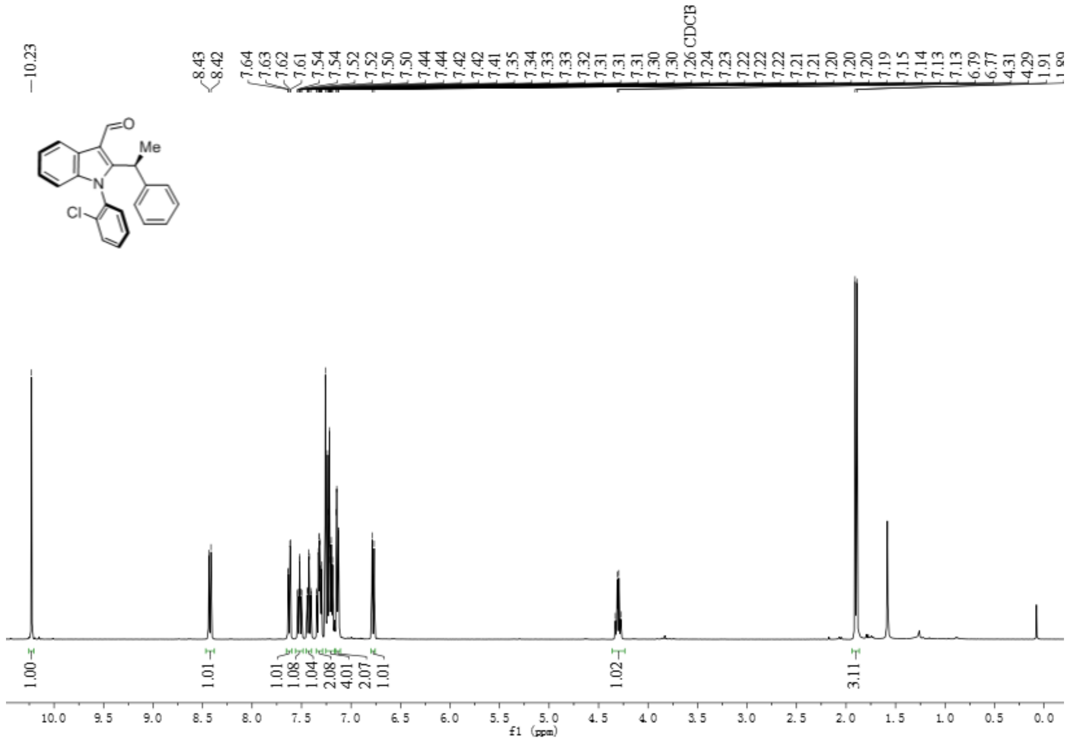

Supplementary Fig. 97. ^{13}C NMR spectrum of **8**

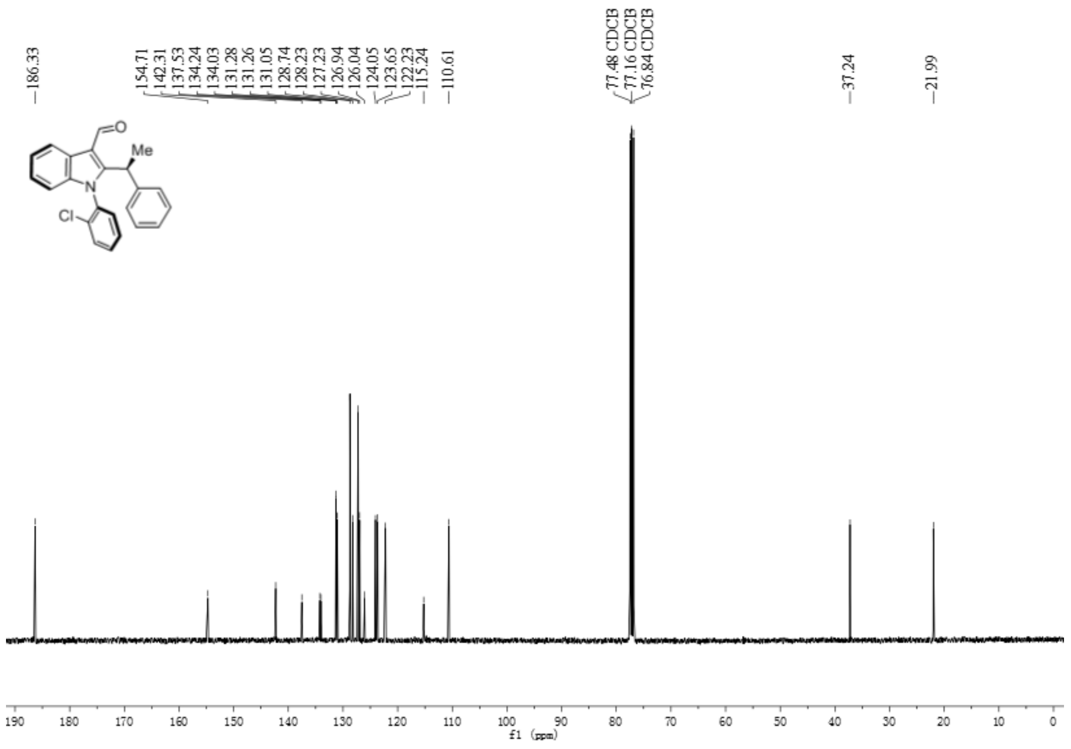

Supplementary Fig. 98. ^{1}H NMR spectrum of **9**

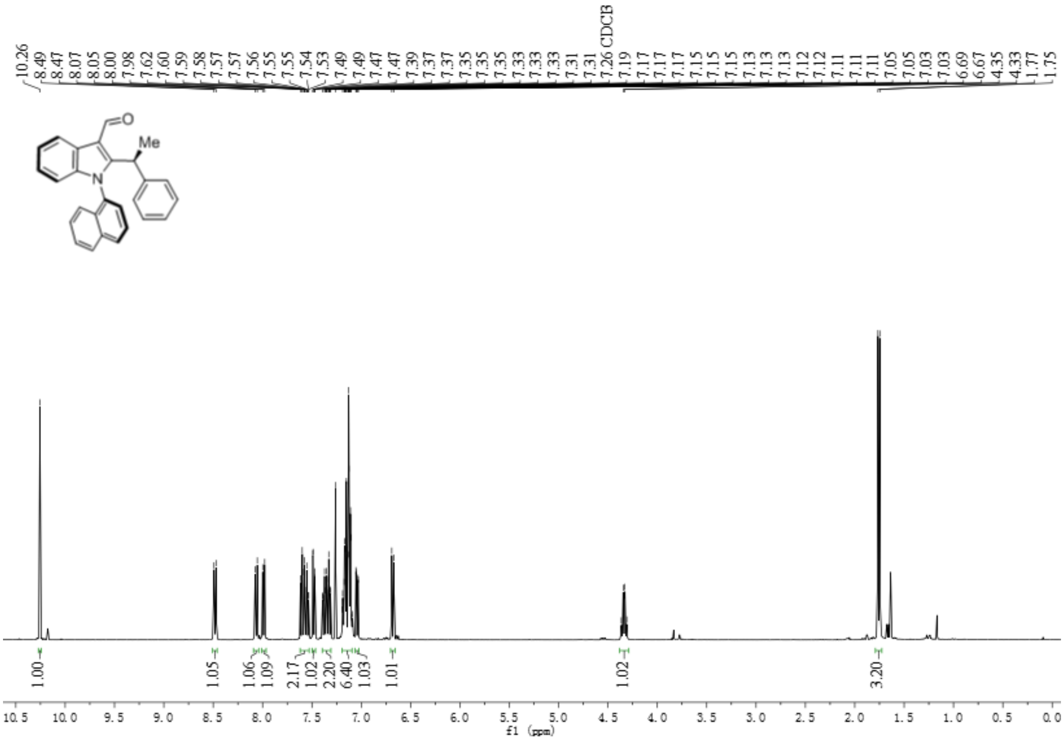

Supplementary Fig. 99. ^{13}C NMR spectrum of **9**

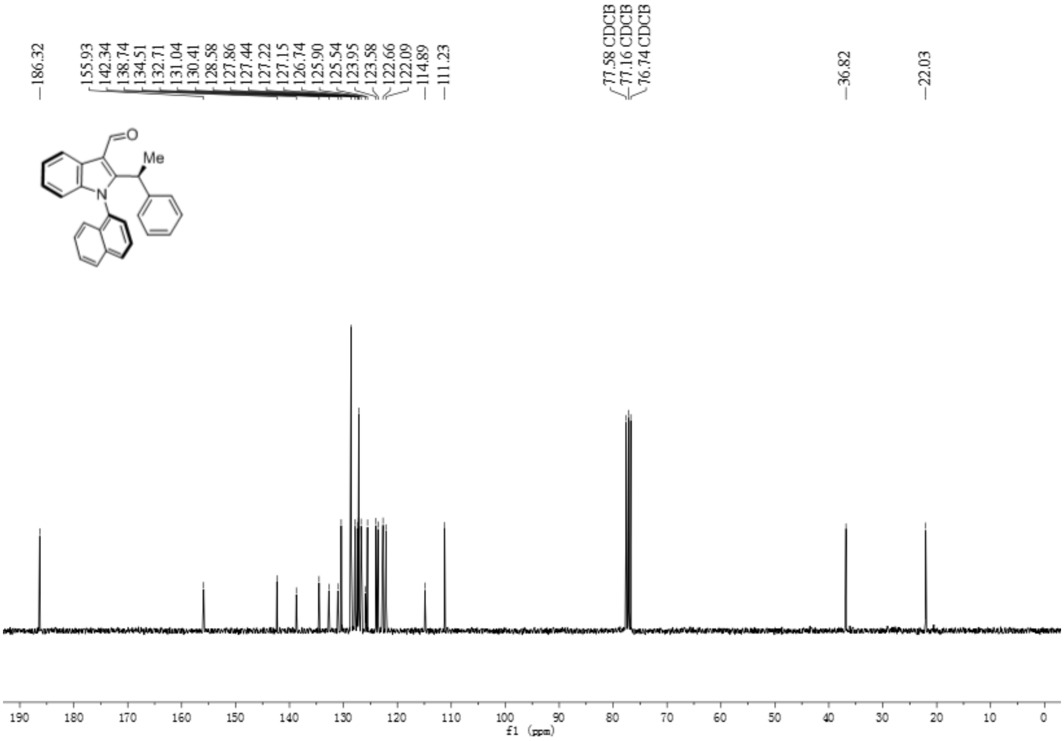

Supplementary Fig. 100. ^{1}H NMR spectrum of **10**

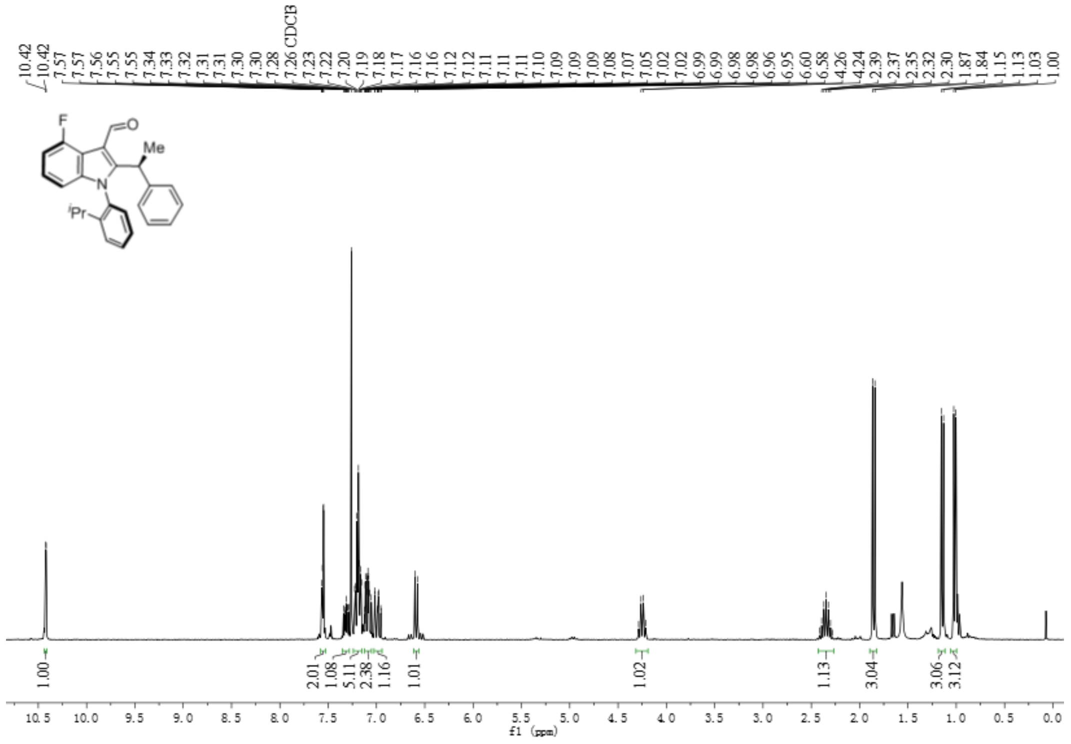

Supplementary Fig. 101. ^{13}C NMR spectrum of **10**

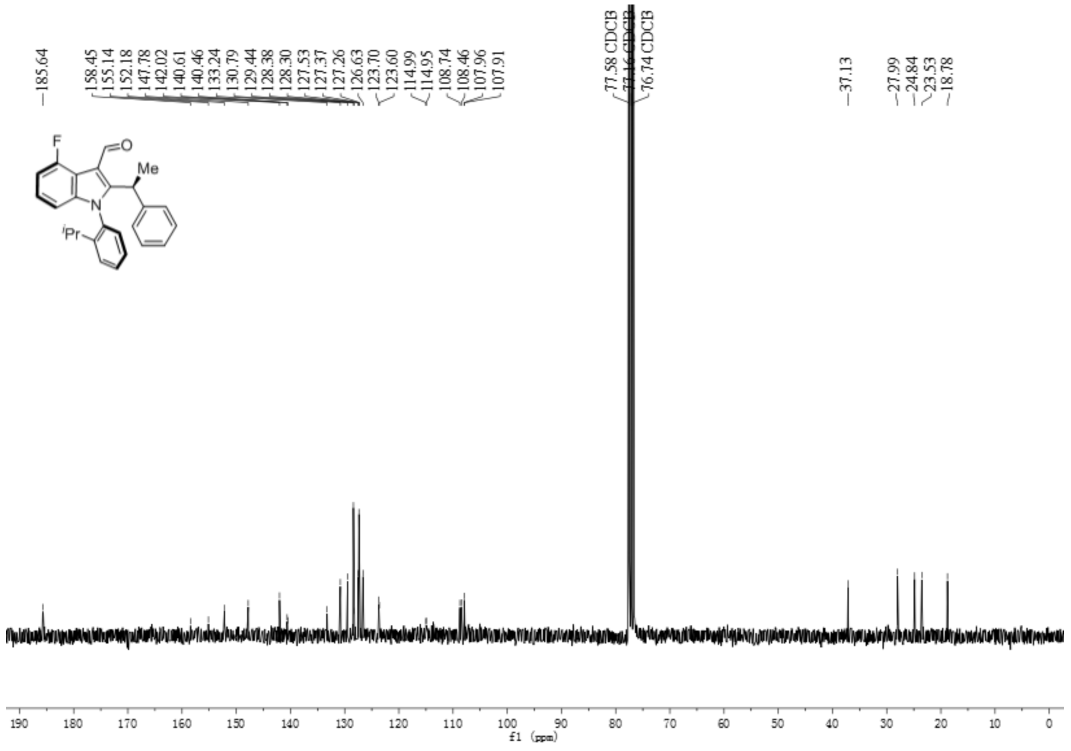

Supplementary Fig. 102. ^{19}F NMR spectrum of **10**

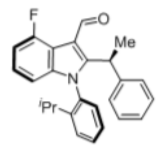

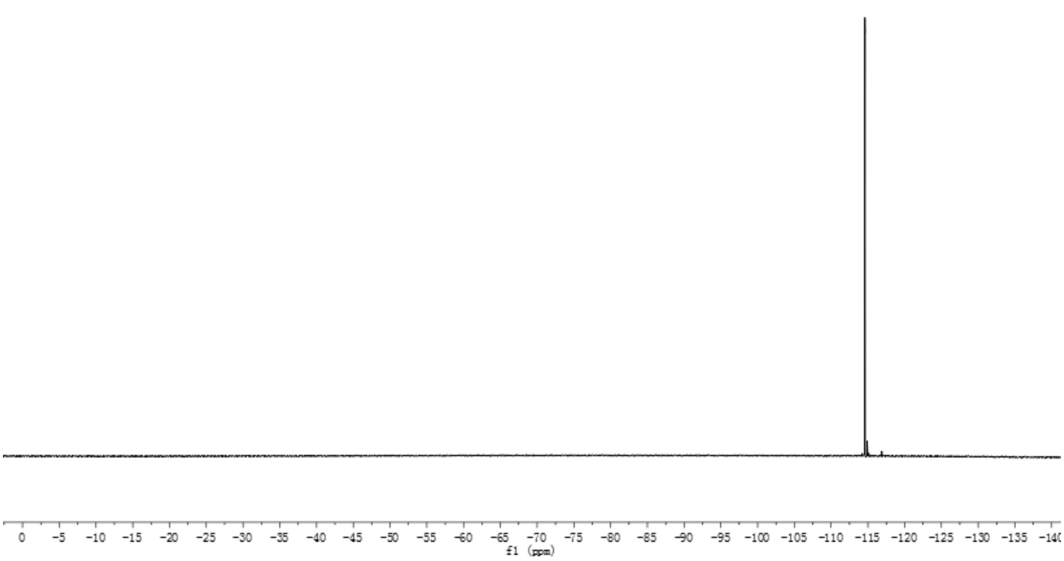

Supplementary Fig. 103. ^{1}H NMR spectrum of **11**

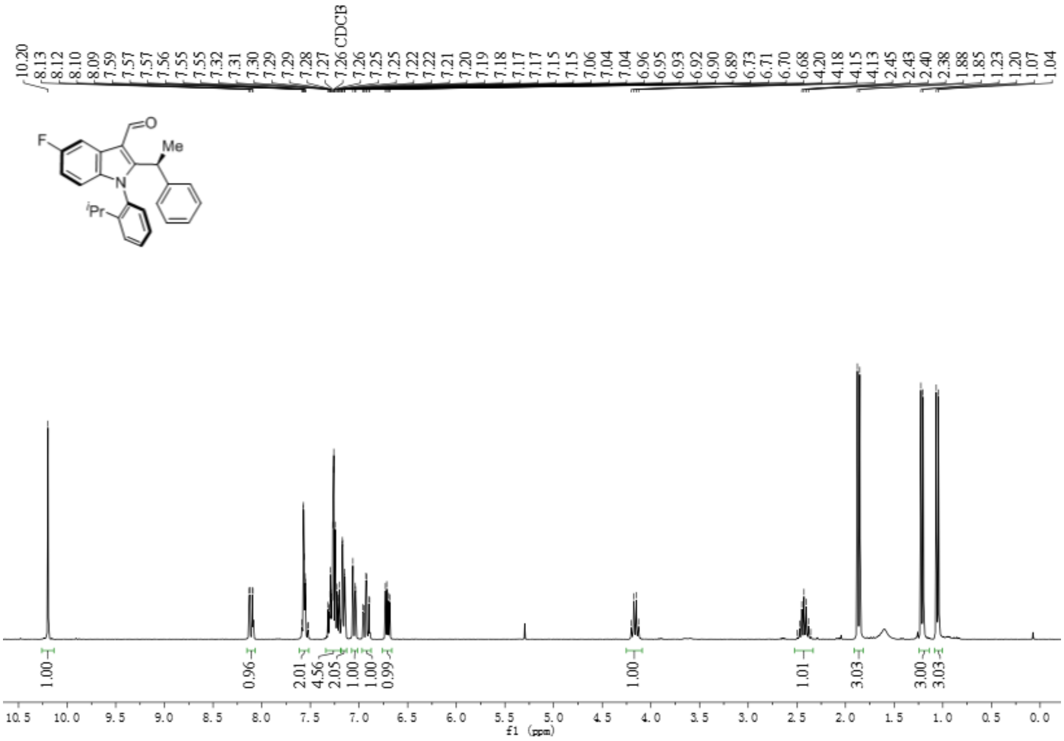

Supplementary Fig. 104. ^{13}C NMR spectrum of **11**

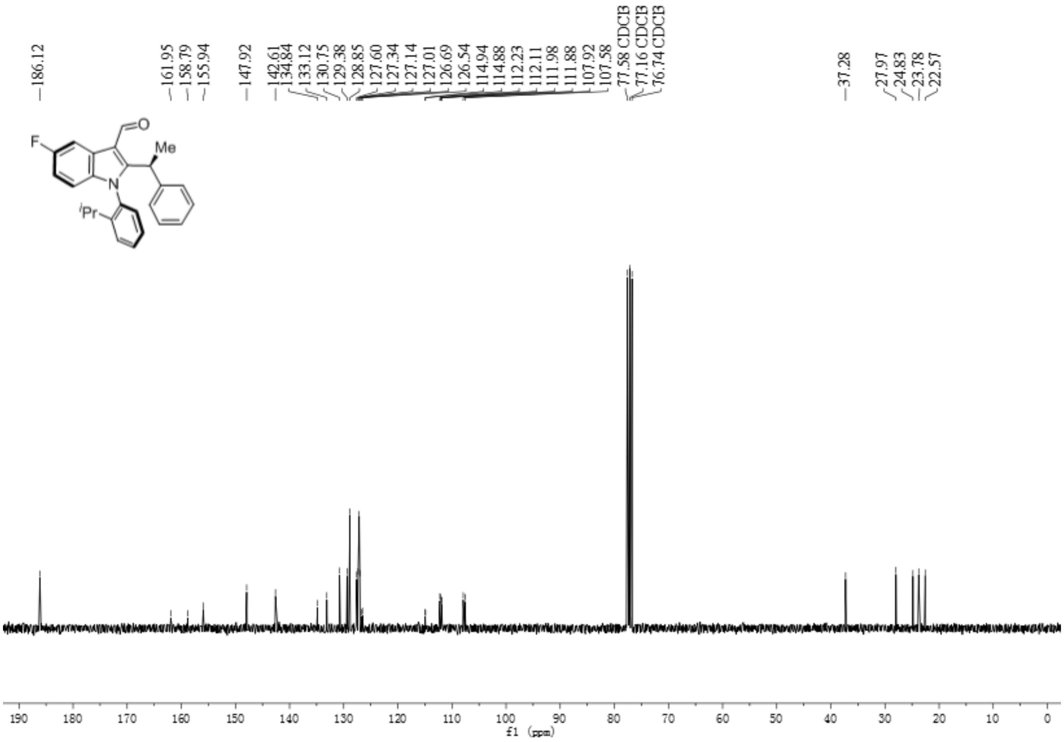

Supplementary Fig. 105. ^{19}F NMR spectrum of **11**

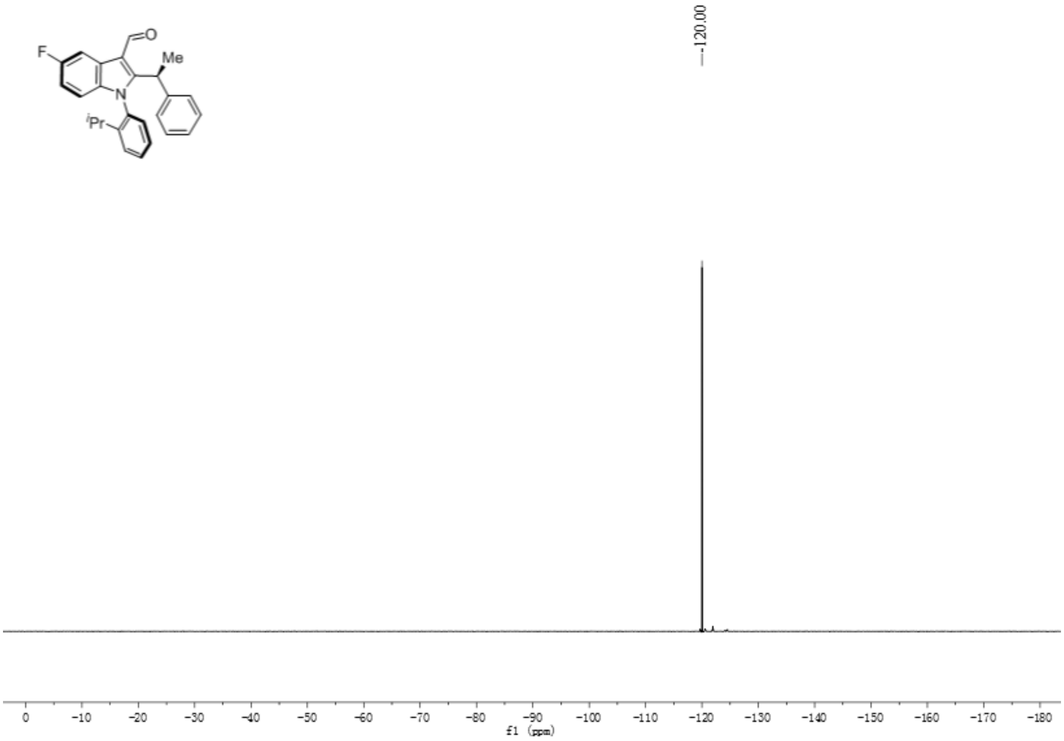

Supplementary Fig. 106. ^{1}H NMR spectrum of **12**

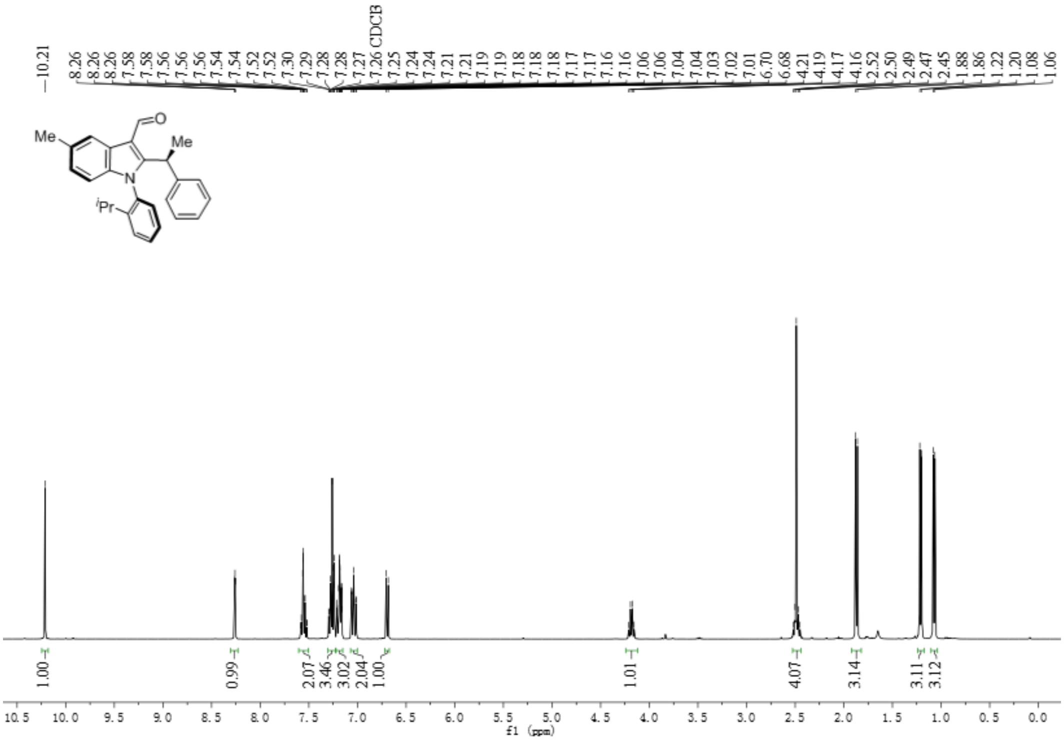

Supplementary Fig. 107. ^{13}C NMR spectrum of **12**

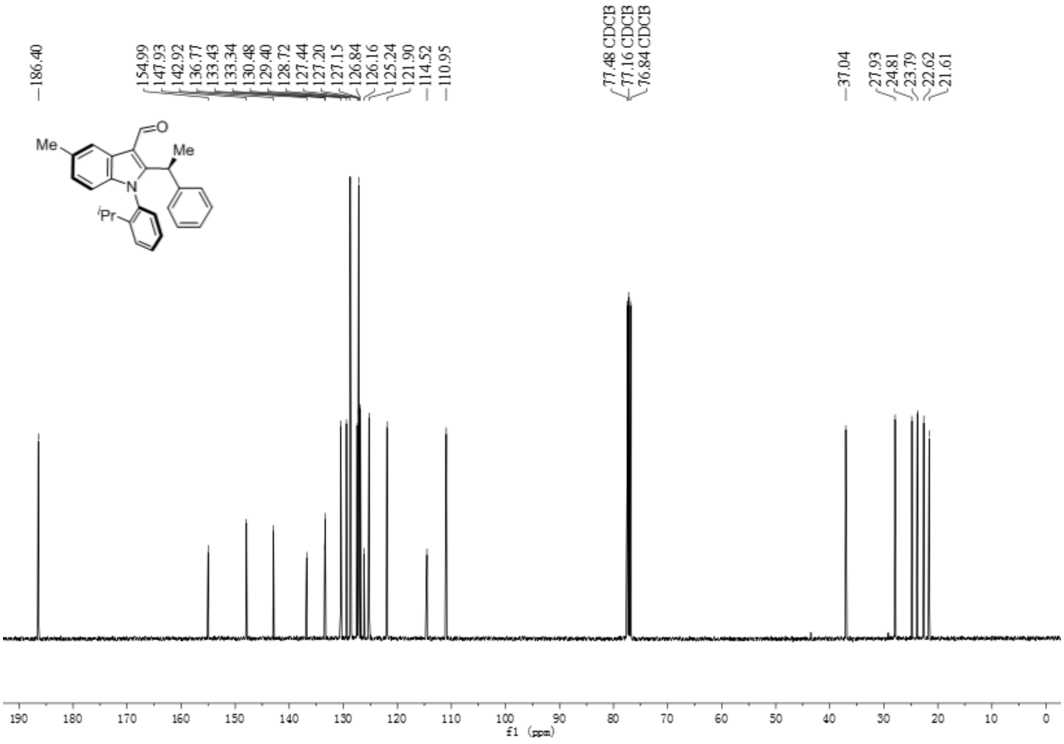

Supplementary Fig. 108. ^{1}H NMR spectrum of **13**

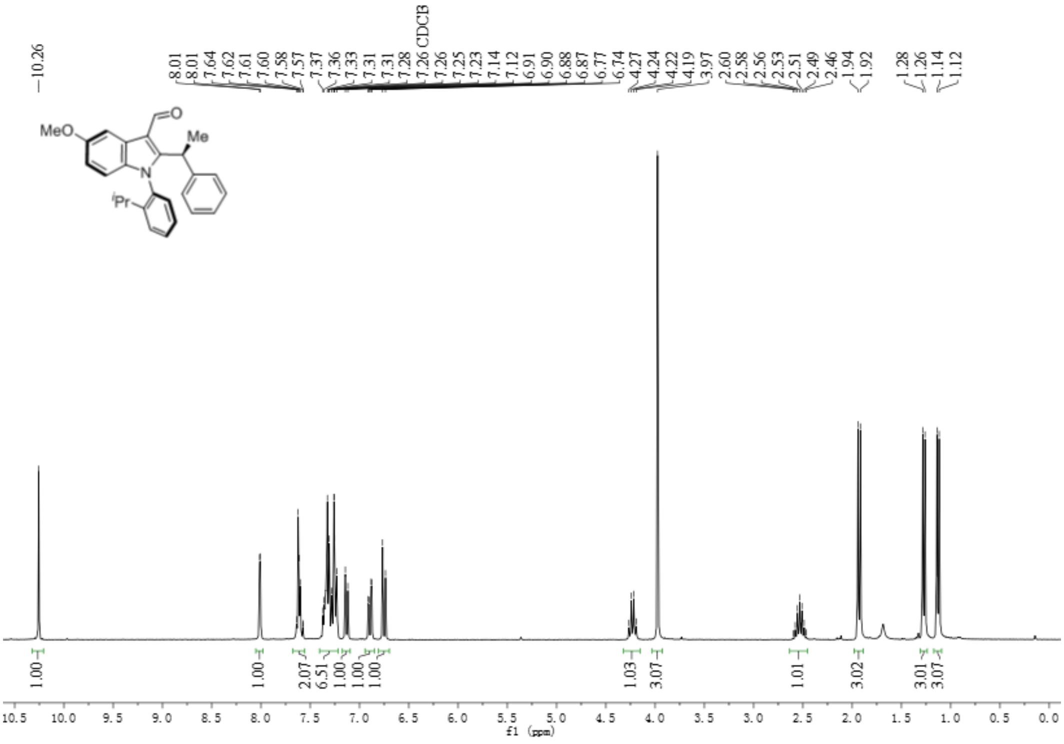

Supplementary Fig. 109. ^{13}C NMR spectrum of **13**

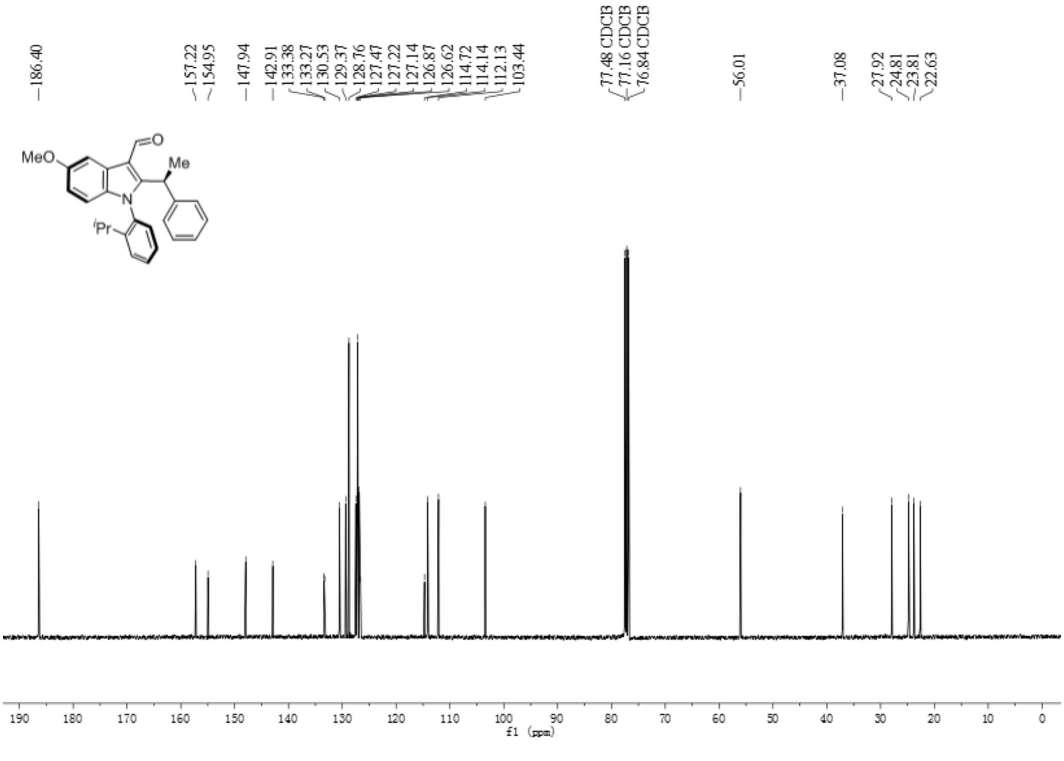

Supplementary Fig. 110. ^{1}H NMR spectrum of **14**

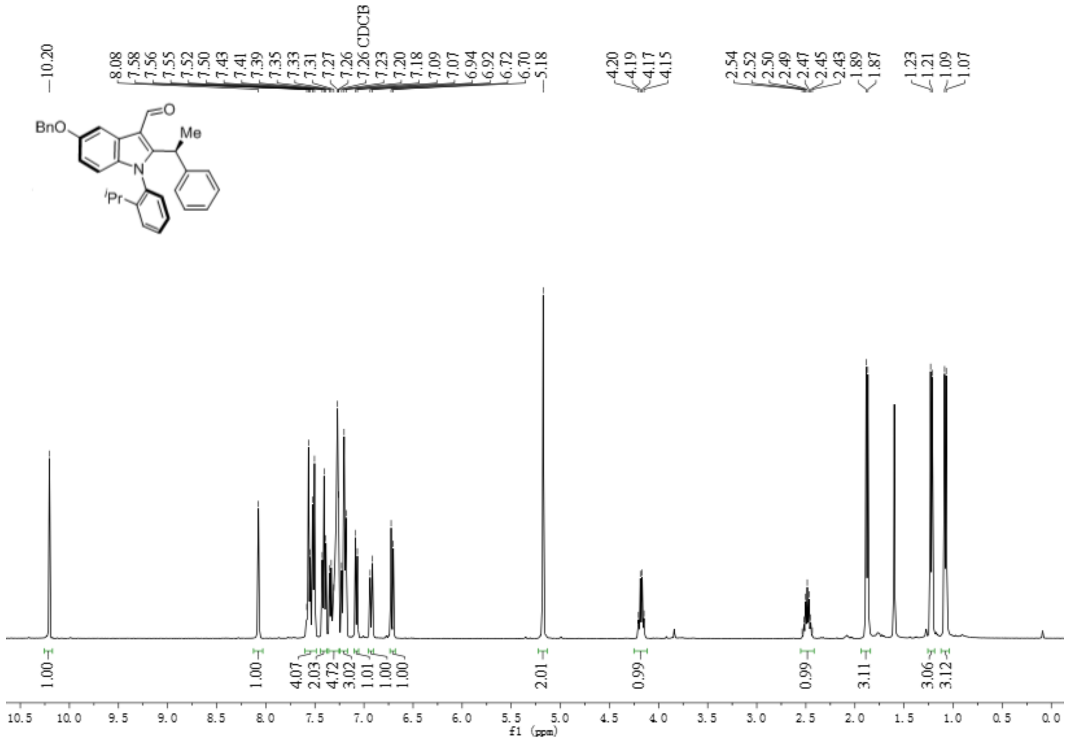

Supplementary Fig. 111. ^{13}C NMR spectrum of **14**

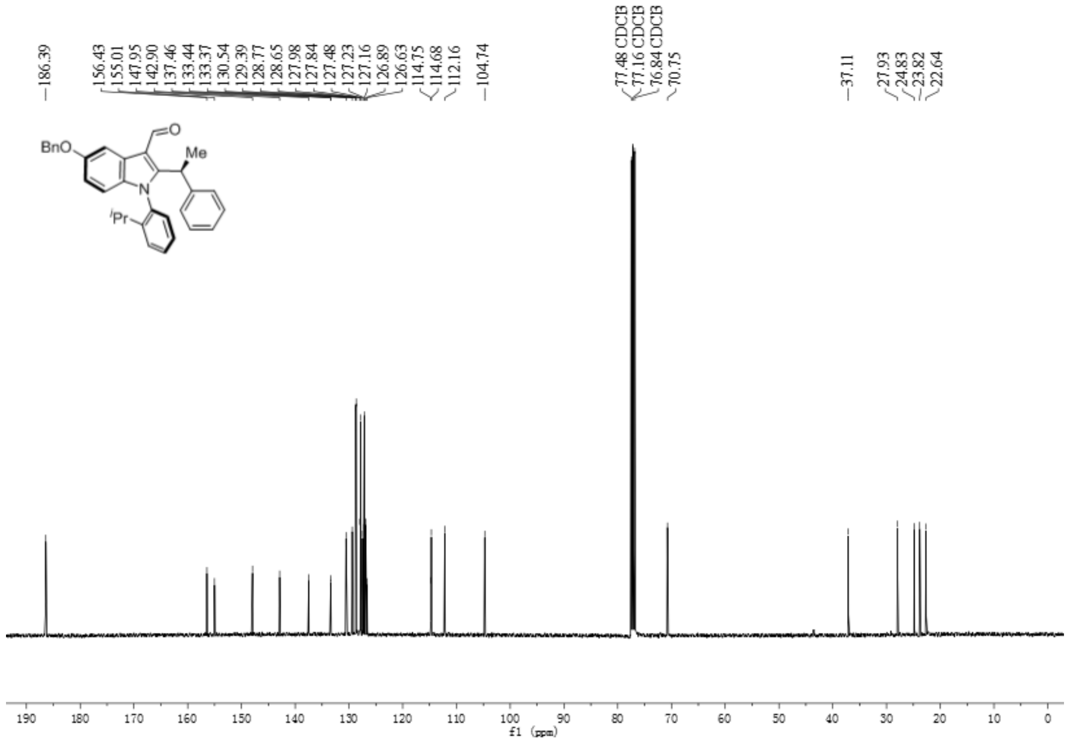

Supplementary Fig. 112. ^{1}H NMR spectrum of **15**

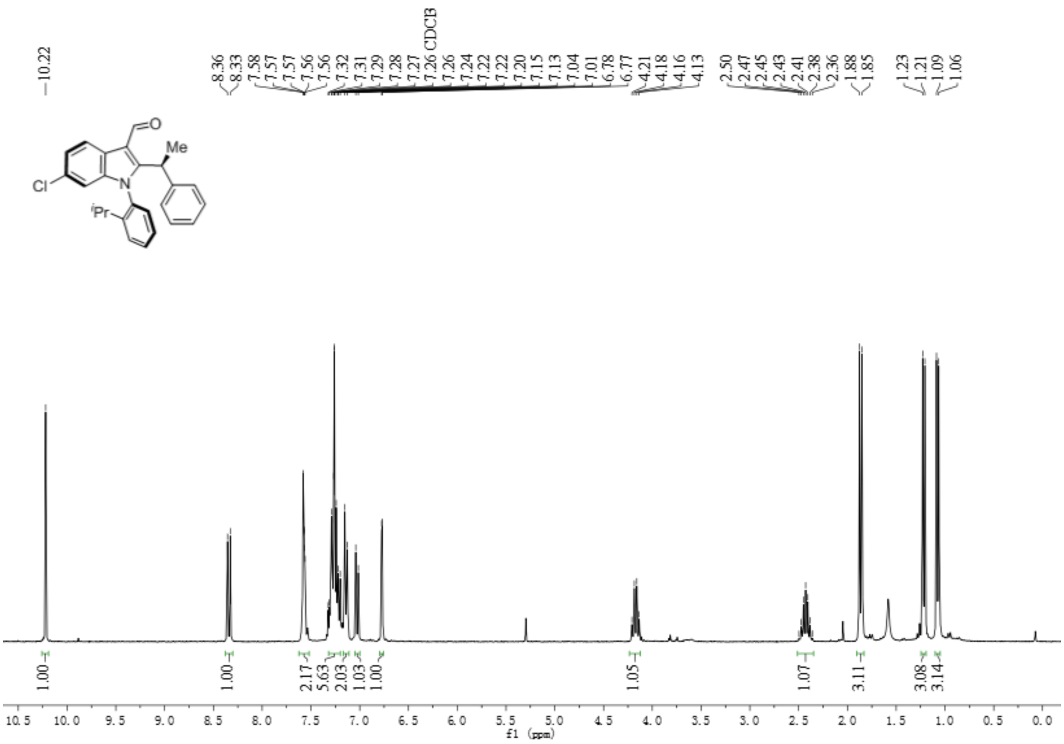

Supplementary Fig. 113. ^{13}C NMR spectrum of **15**

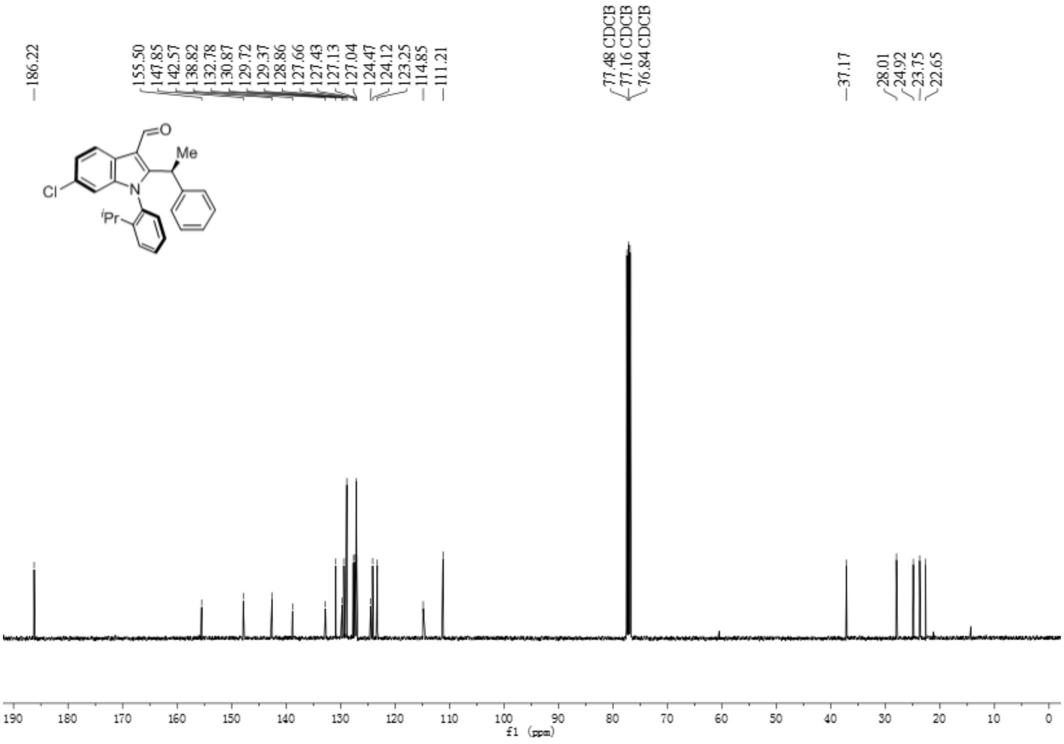

Supplementary Fig. 114. ^{1}H NMR spectrum of **16**

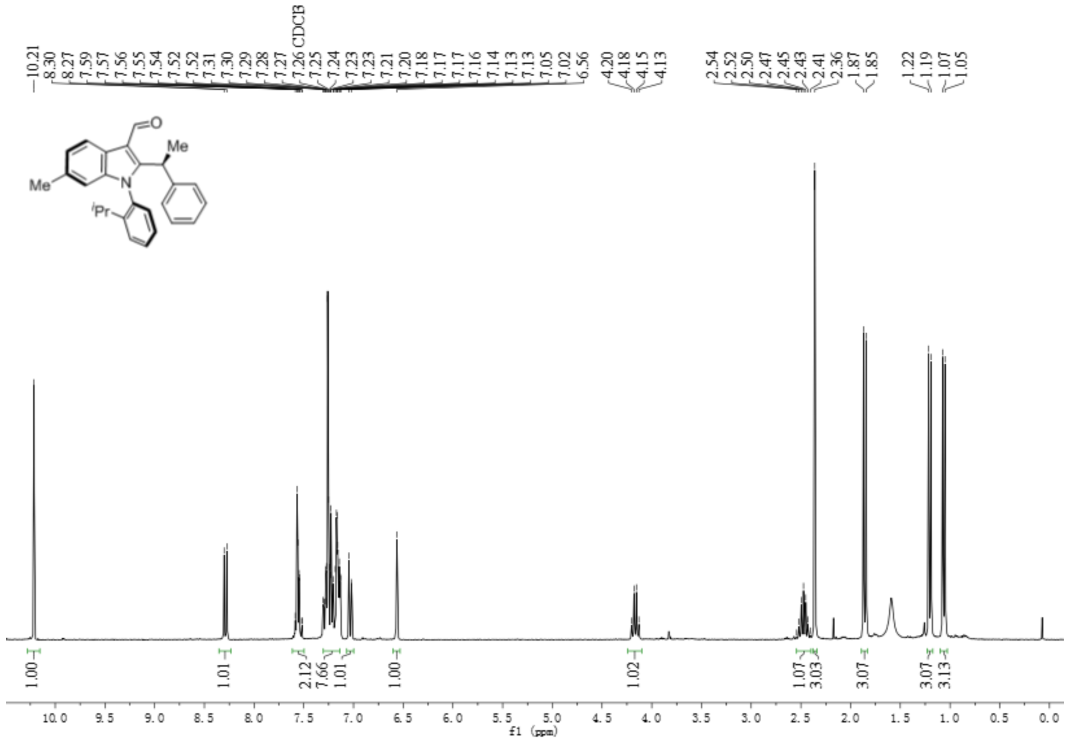

Supplementary Fig. 115. ^{13}C NMR spectrum of **16**

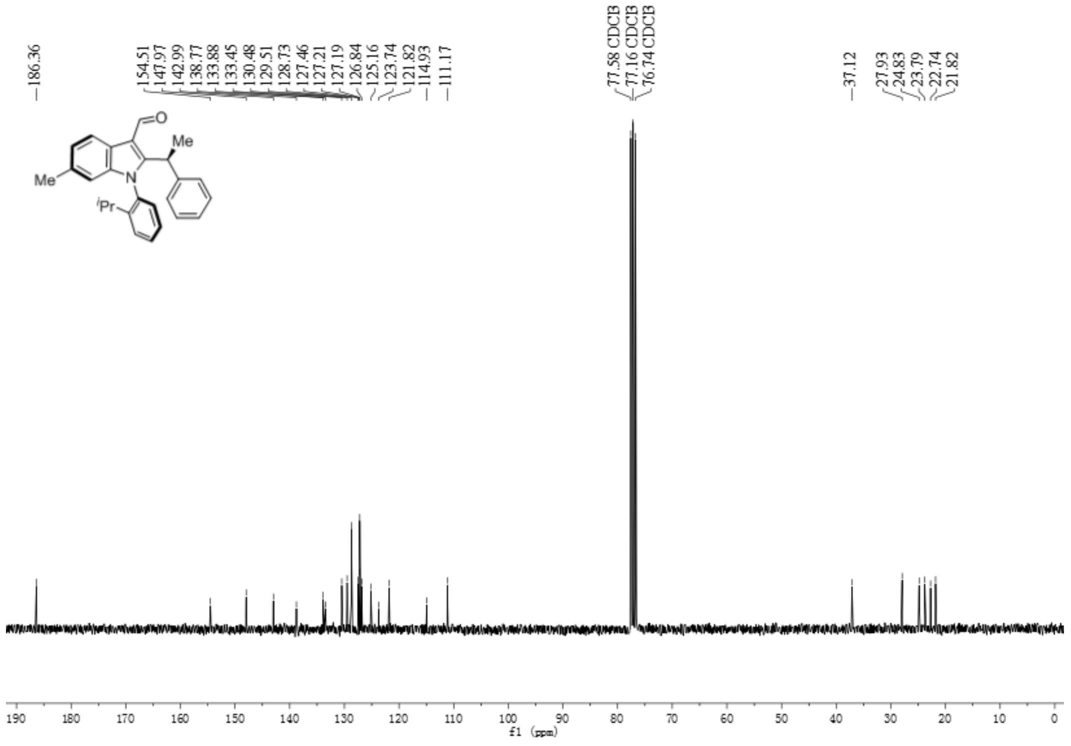

Supplementary Fig. 116. ^{1}H NMR spectrum of **17**

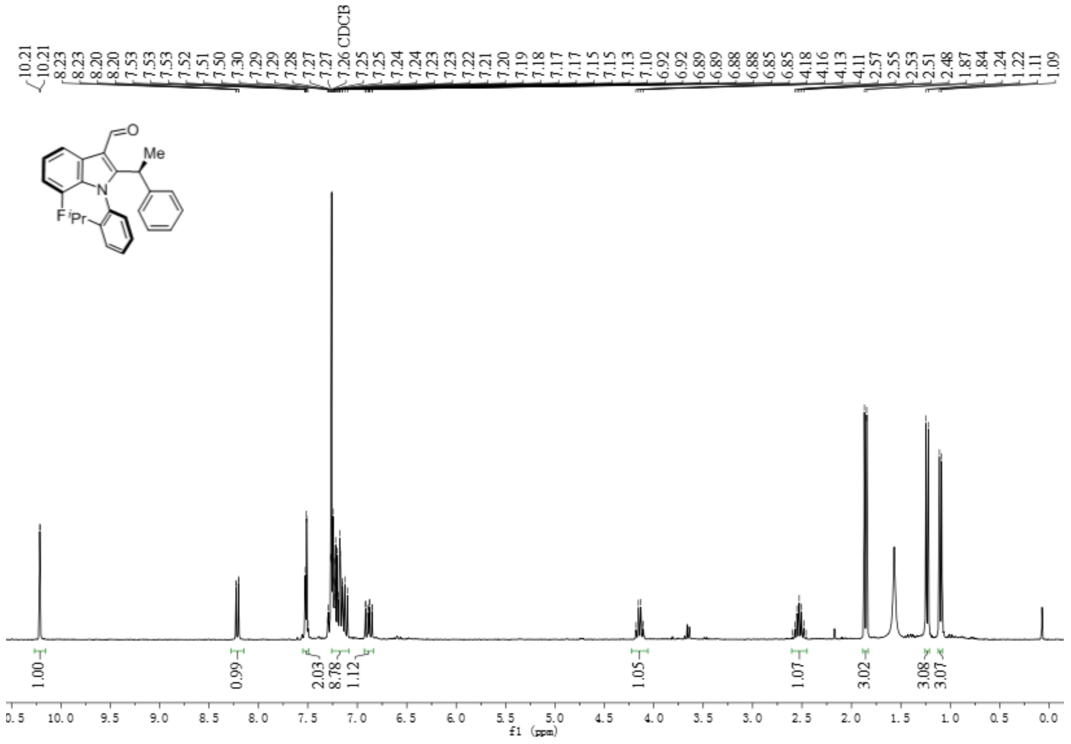

Supplementary Fig. 117. ^{13}C NMR spectrum of **17**

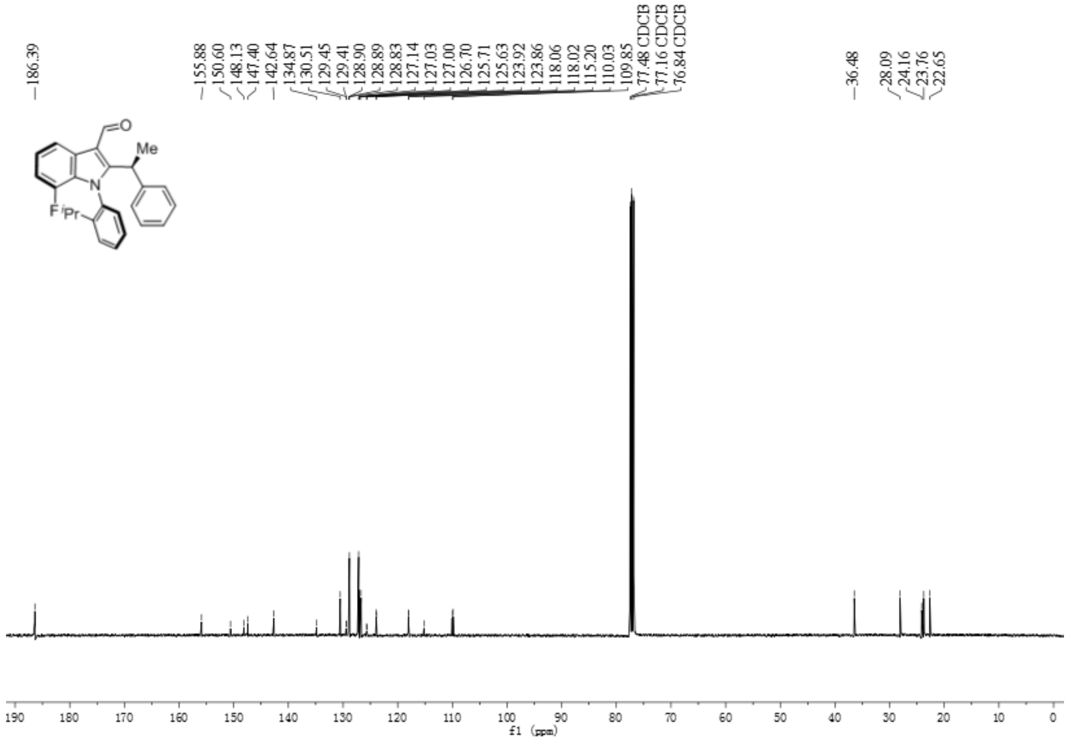

Supplementary Fig. 118. ^{19}F NMR spectrum of **17**

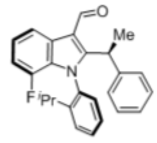

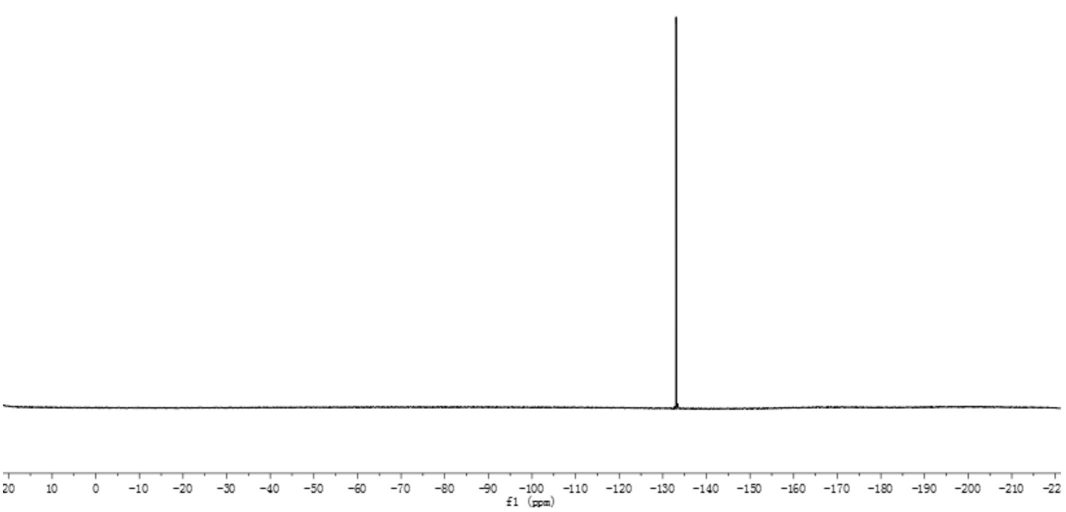

Supplementary Fig. 119. ^{1}H NMR spectrum of **18**

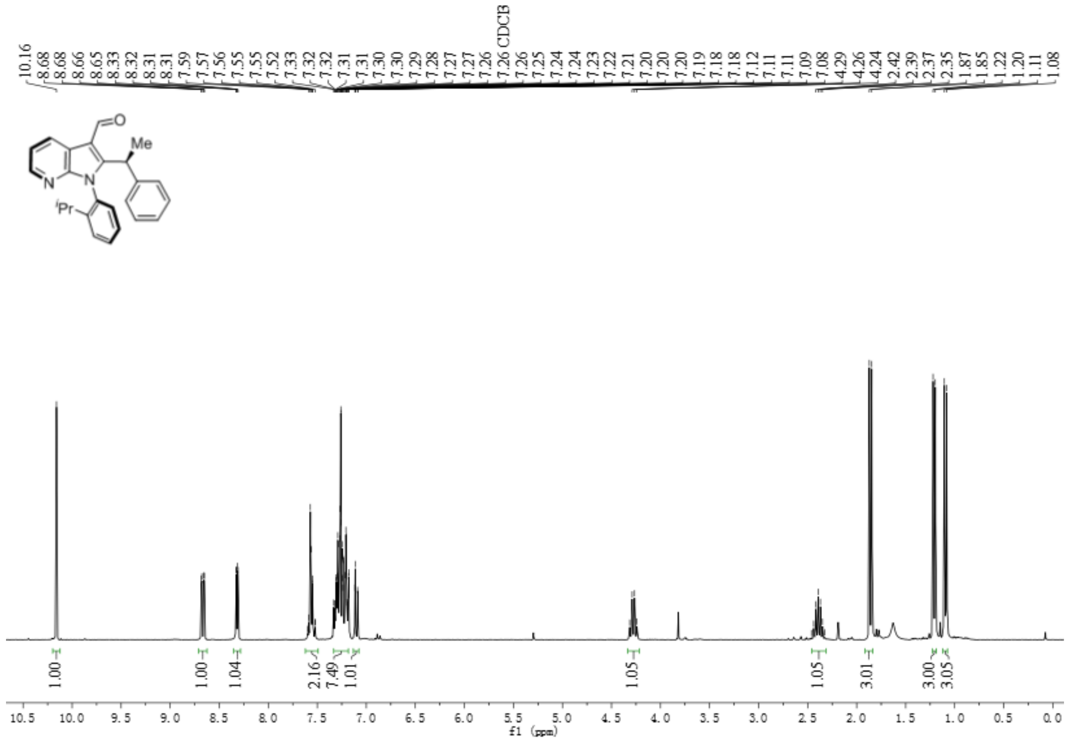

Supplementary Fig. 120. ^{13}C NMR spectrum of **18**

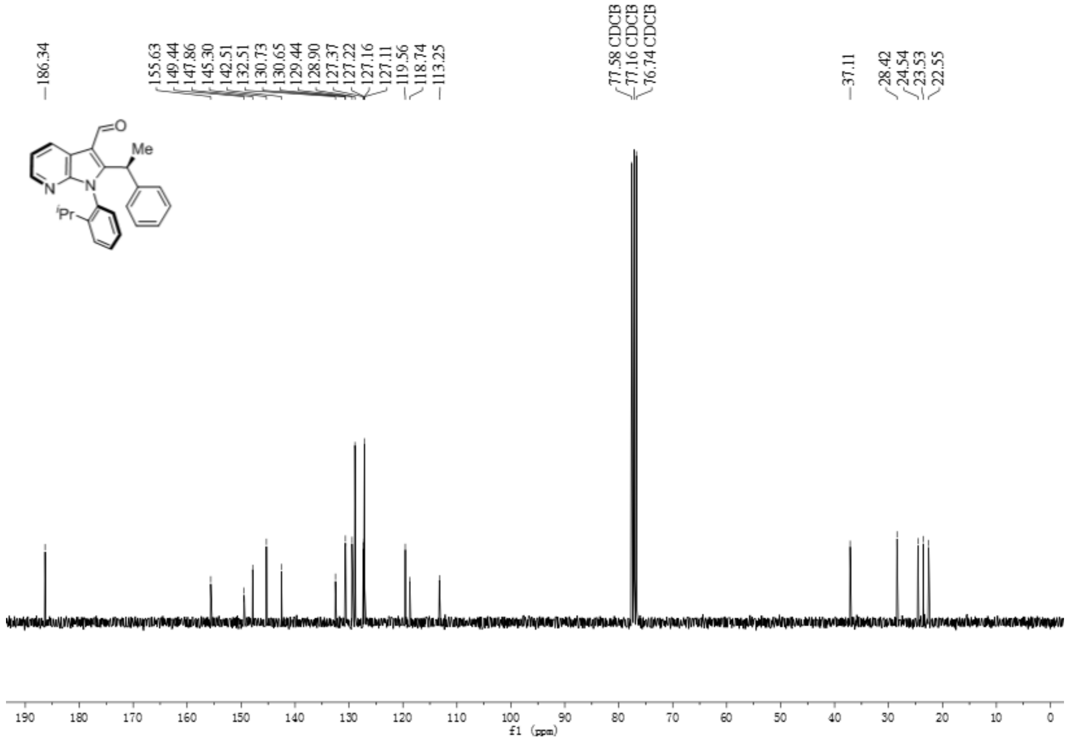

Supplementary Fig. 121. ^{1}H NMR spectrum of **19**

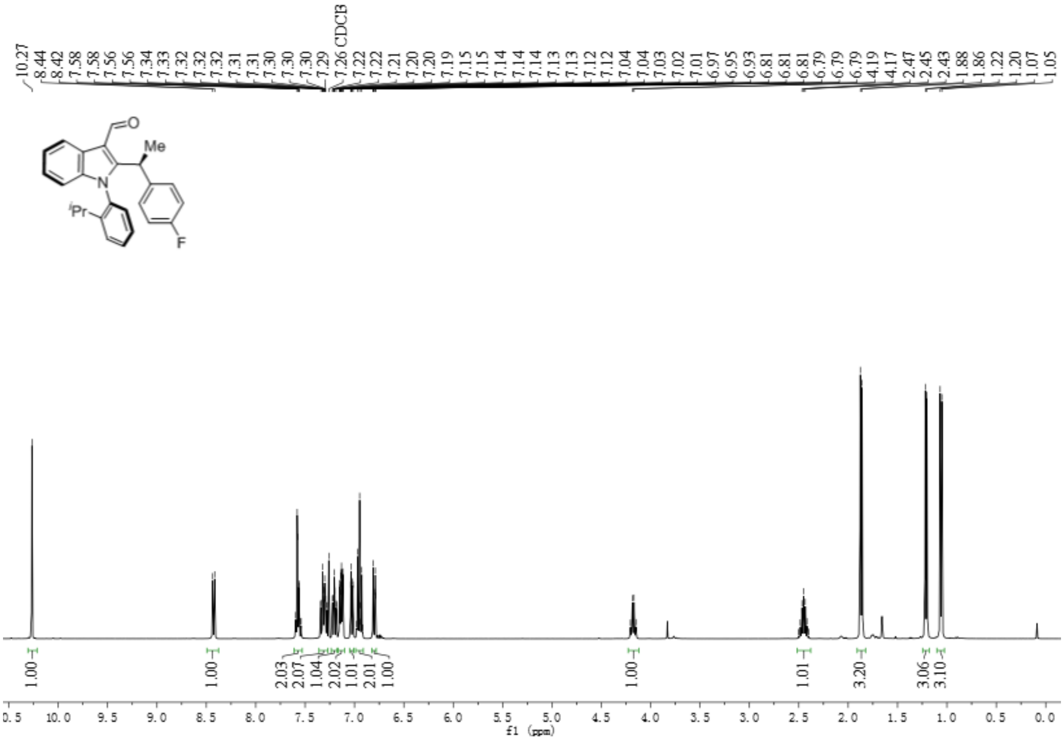

Supplementary Fig. 122. ^{13}C NMR spectrum of **19**

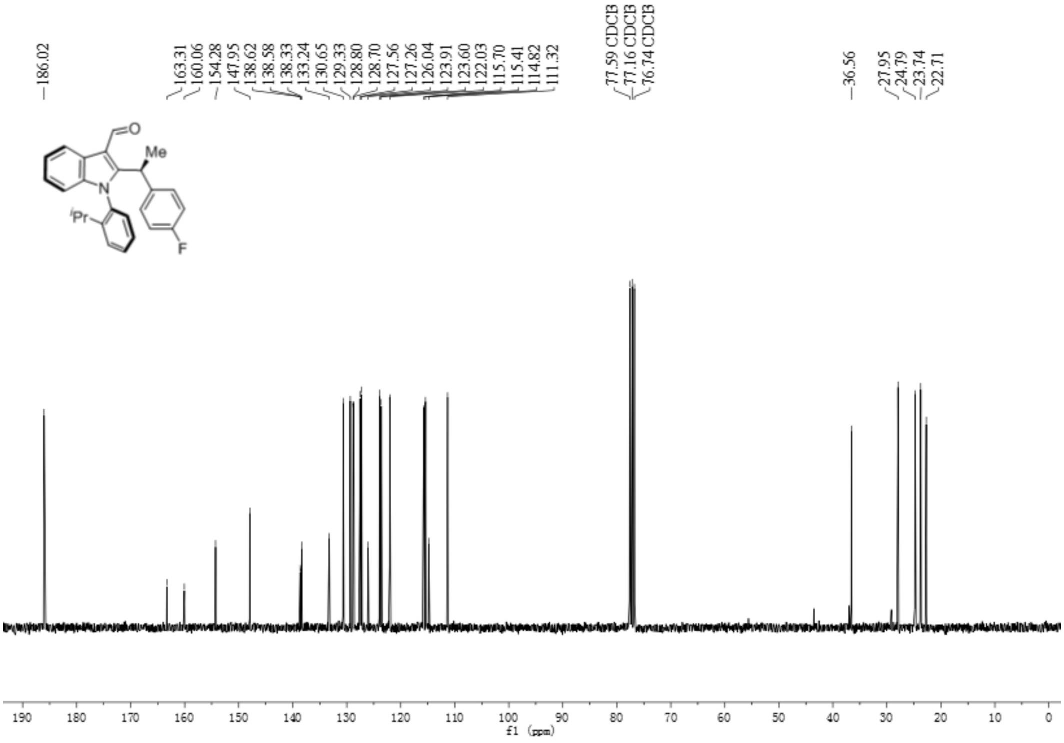

Supplementary Fig. 123. ^{19}F NMR spectrum of **19**

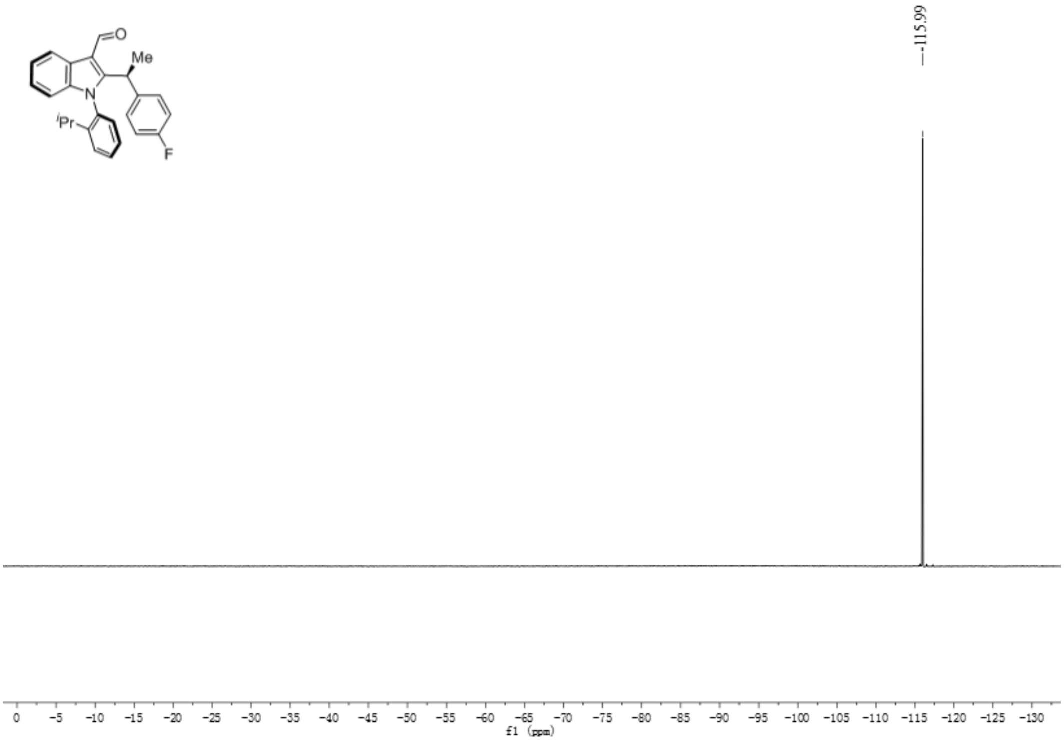

Supplementary Fig. 124. ^{1}H NMR spectrum of **20**

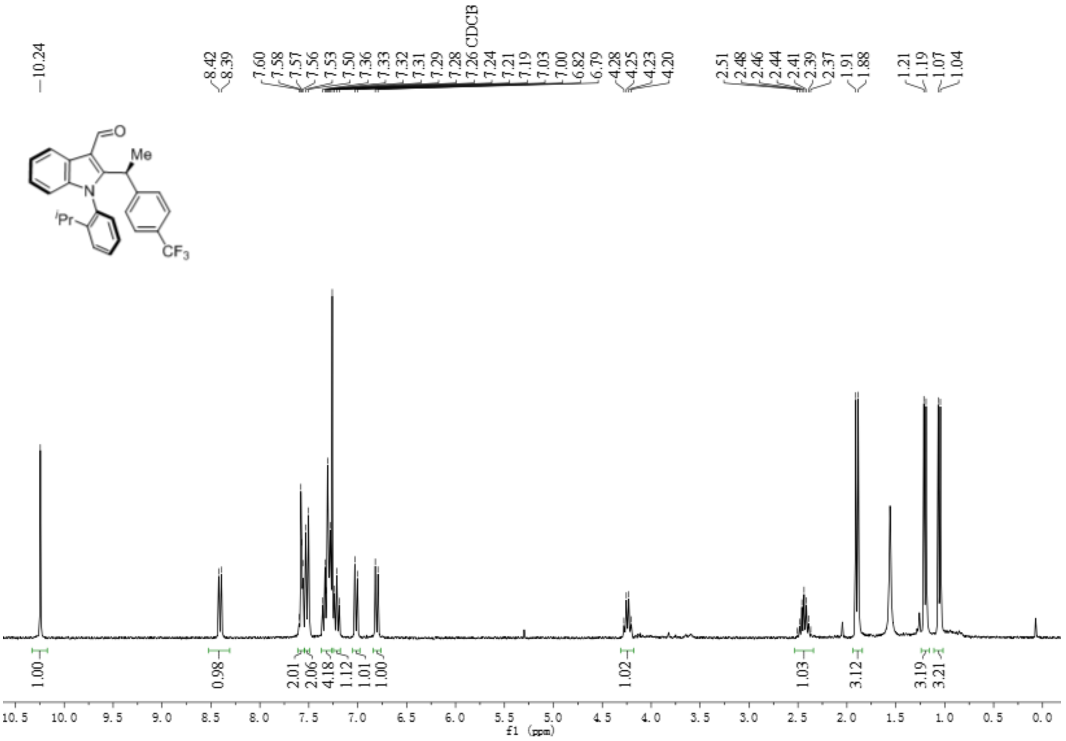

Supplementary Fig. 125. ^{13}C NMR spectrum of **20**

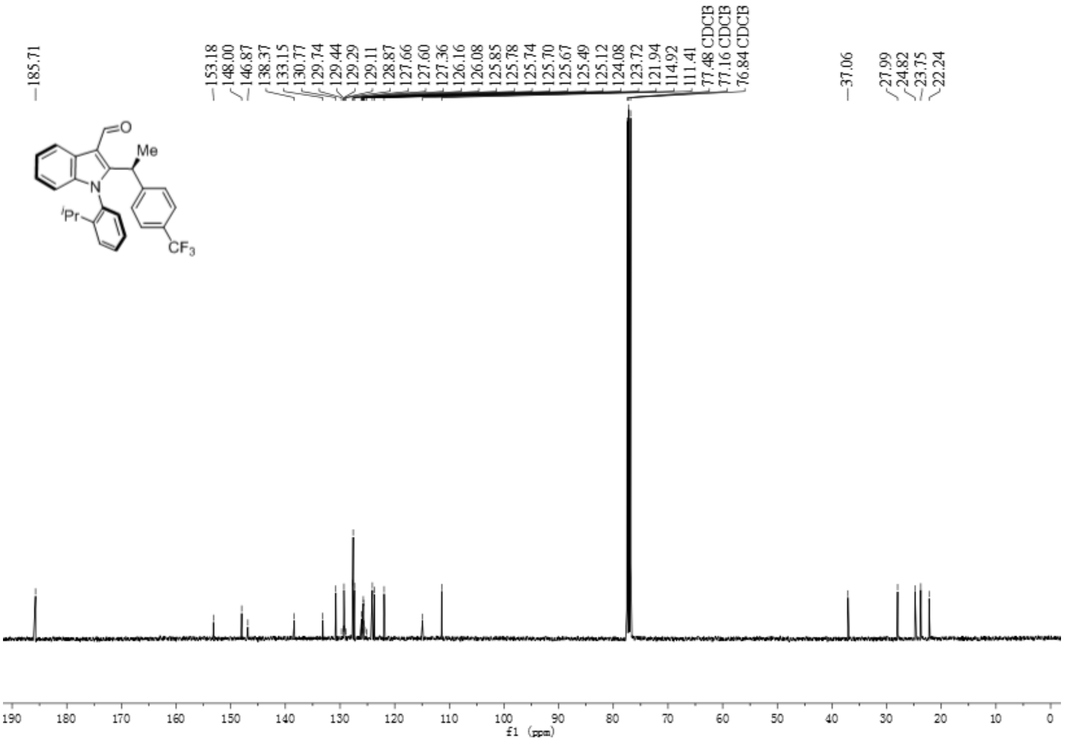

Supplementary Fig. 126. ^{19}F NMR spectrum of **20**

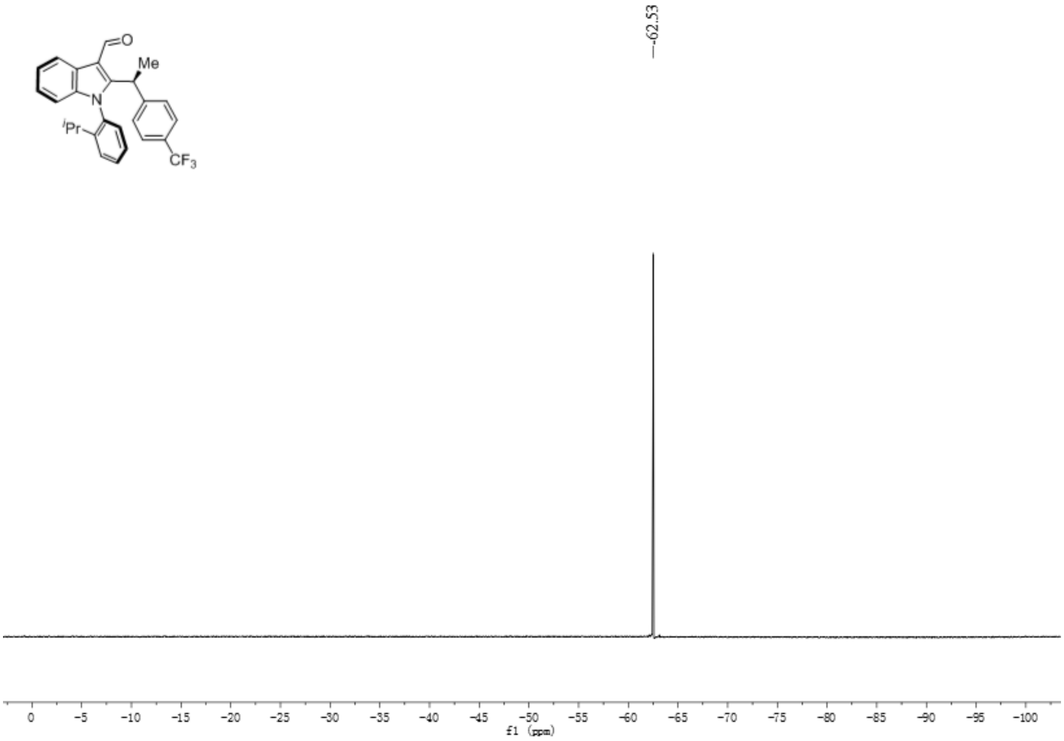

Supplementary Fig. 127. ^{1}H NMR spectrum of **21**

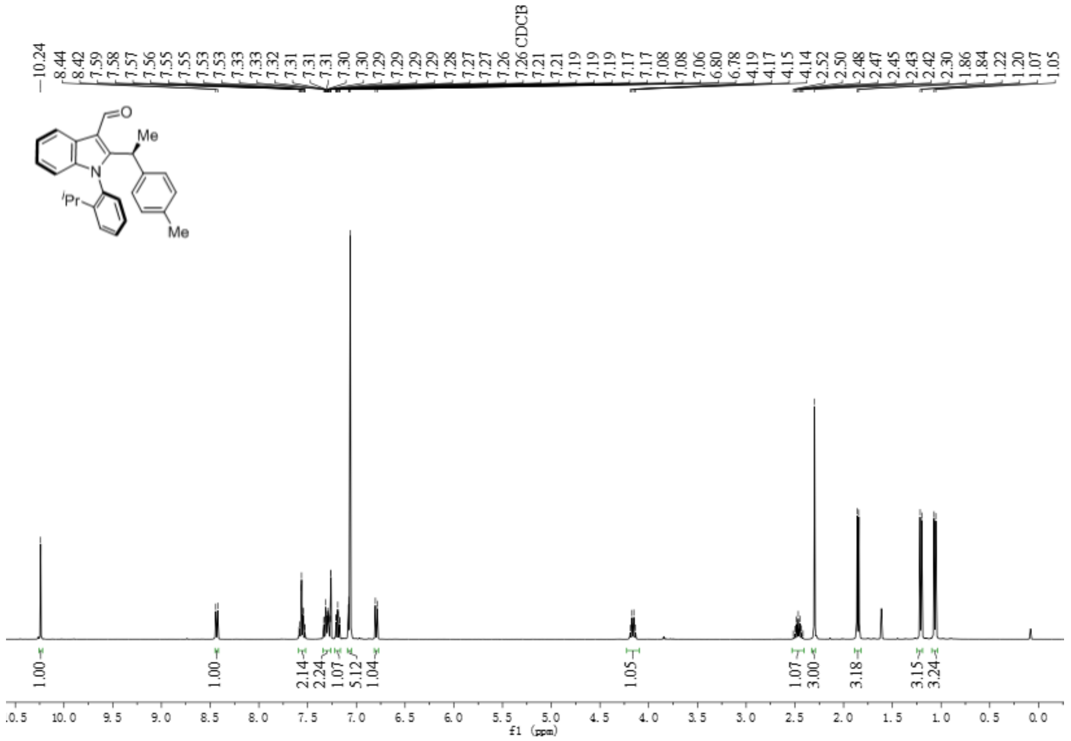

Supplementary Fig. 128. ^{13}C NMR spectrum of **21**

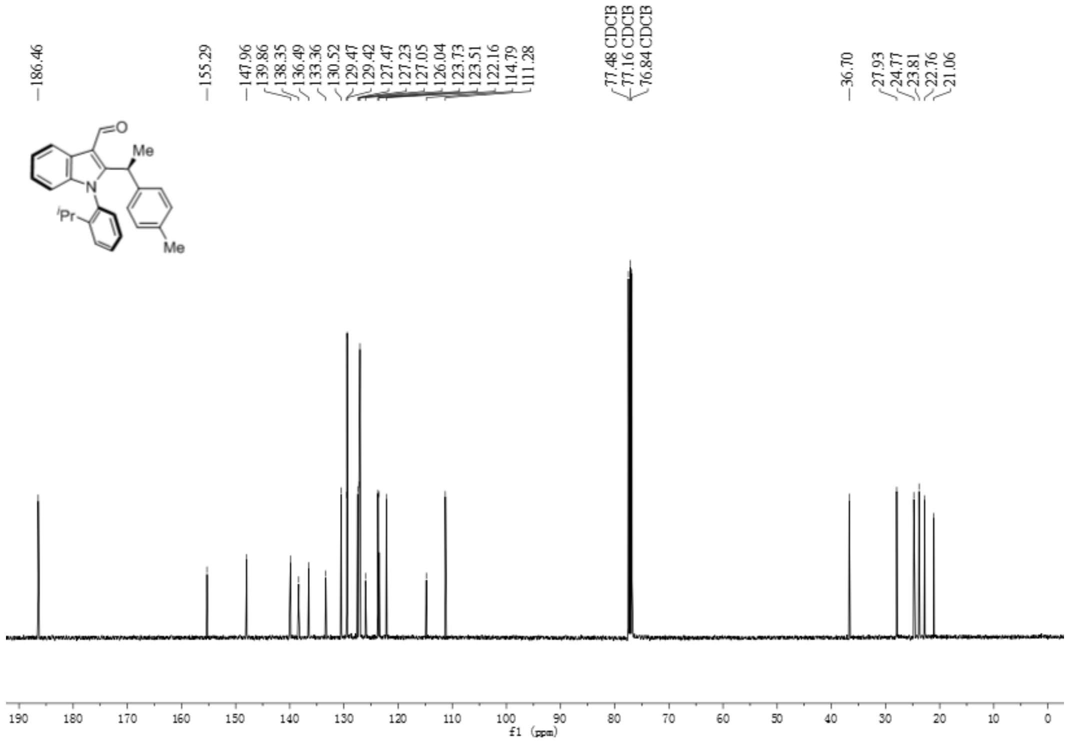

Supplementary Fig. 129. ^{1}H NMR spectrum of **22**

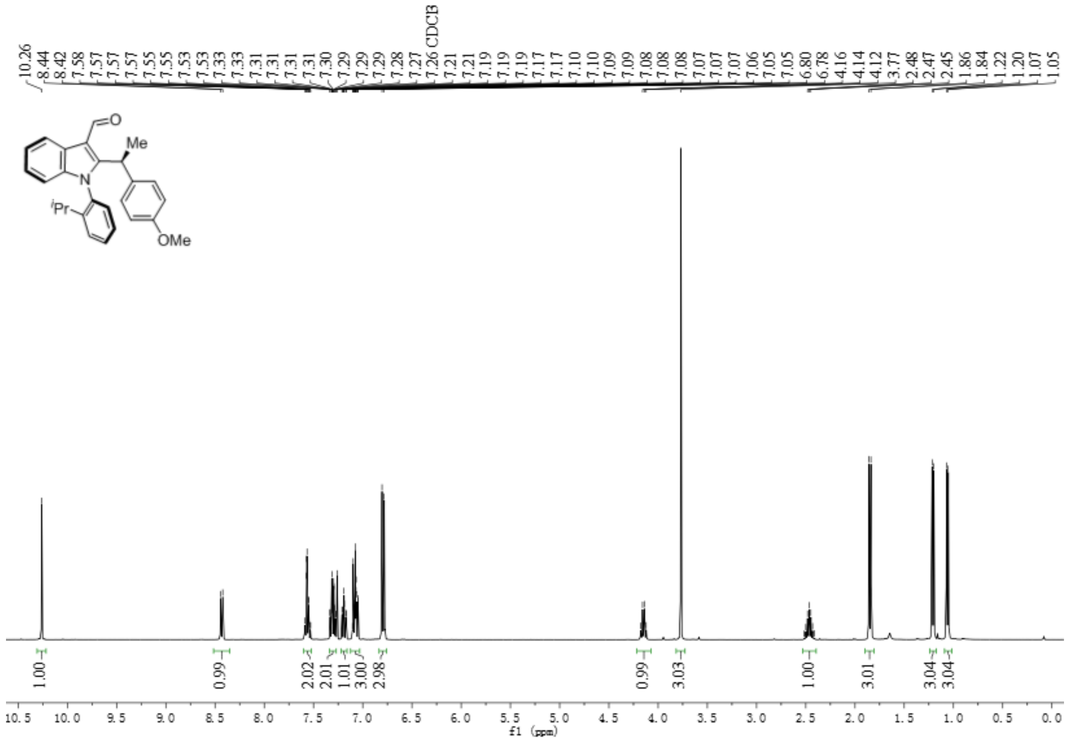

Supplementary Fig. 130. ^{13}C NMR spectrum of **22**

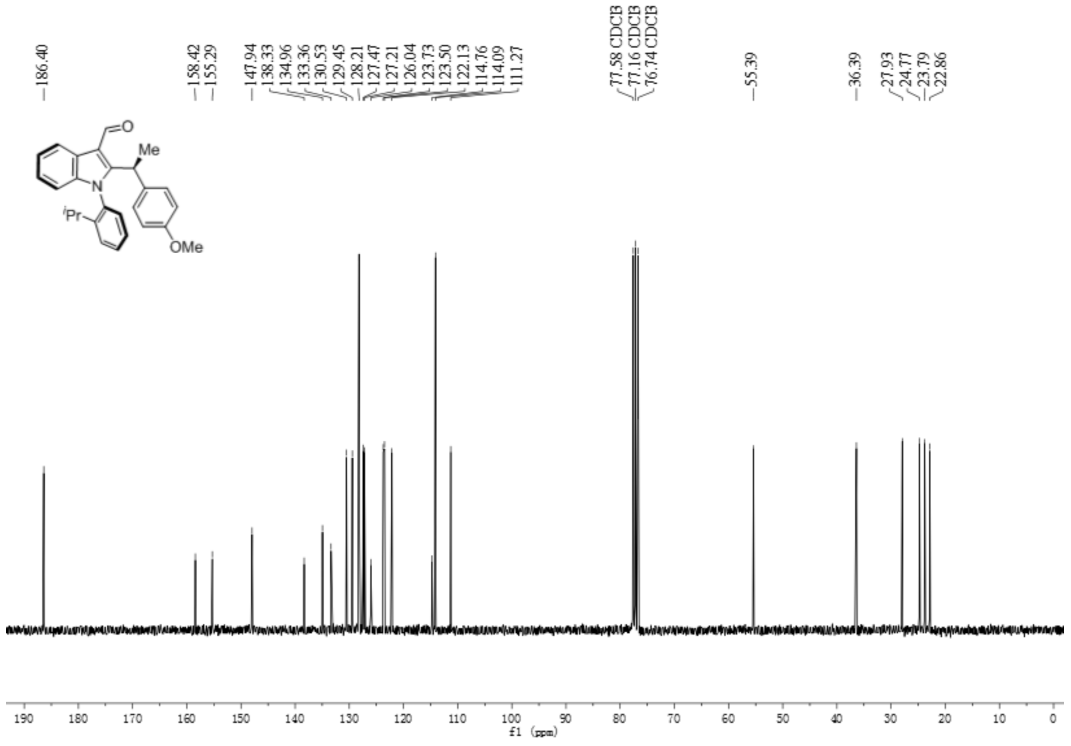

Supplementary Fig. 131. ^{1}H NMR spectrum of **23**

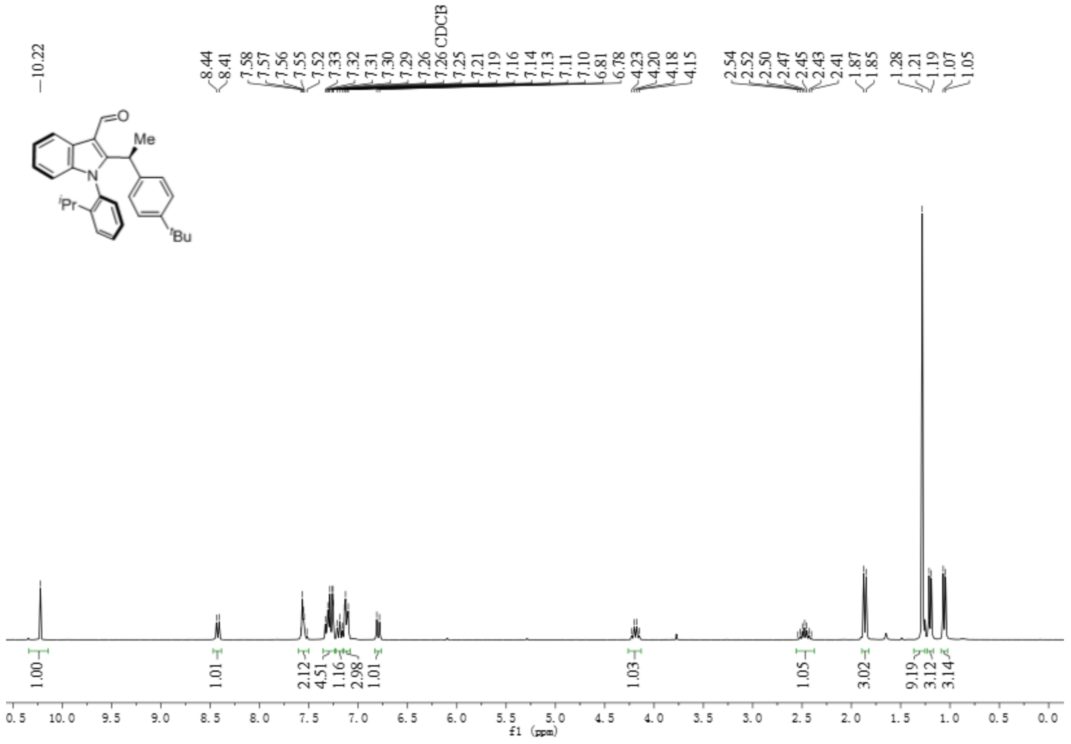

Supplementary Fig. 132. ^{13}C NMR spectrum of **23**

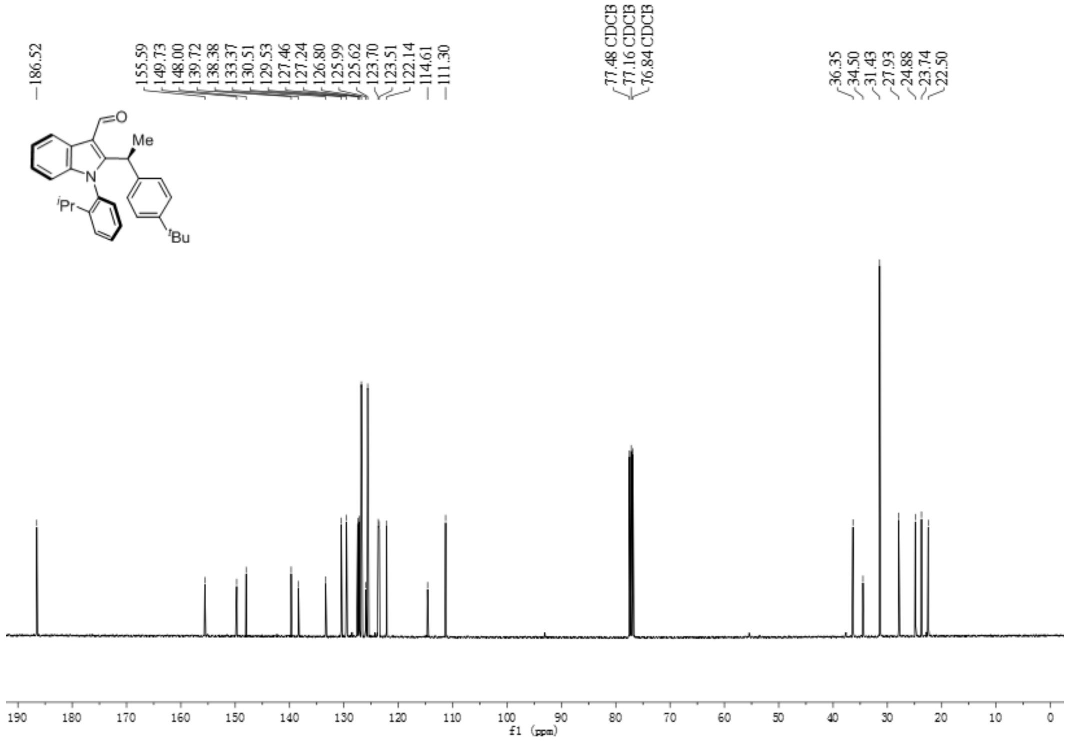

Supplementary Fig. 133. ^{1}H NMR spectrum of **24**

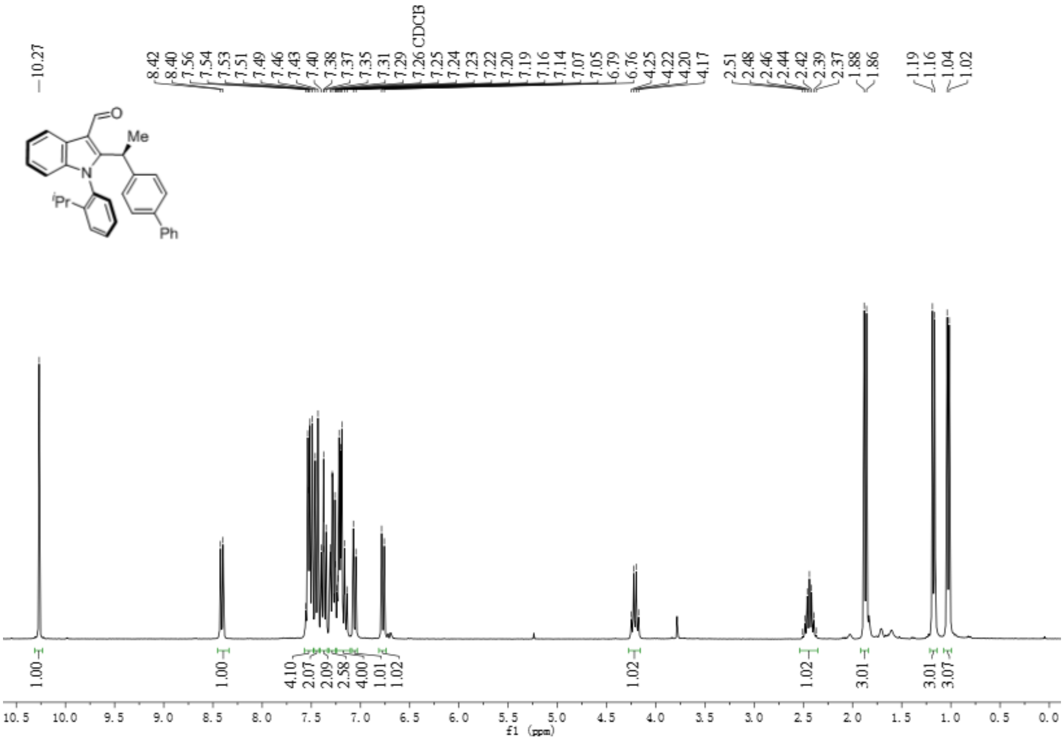

Supplementary Fig. 134. ^{13}C NMR spectrum of **24**

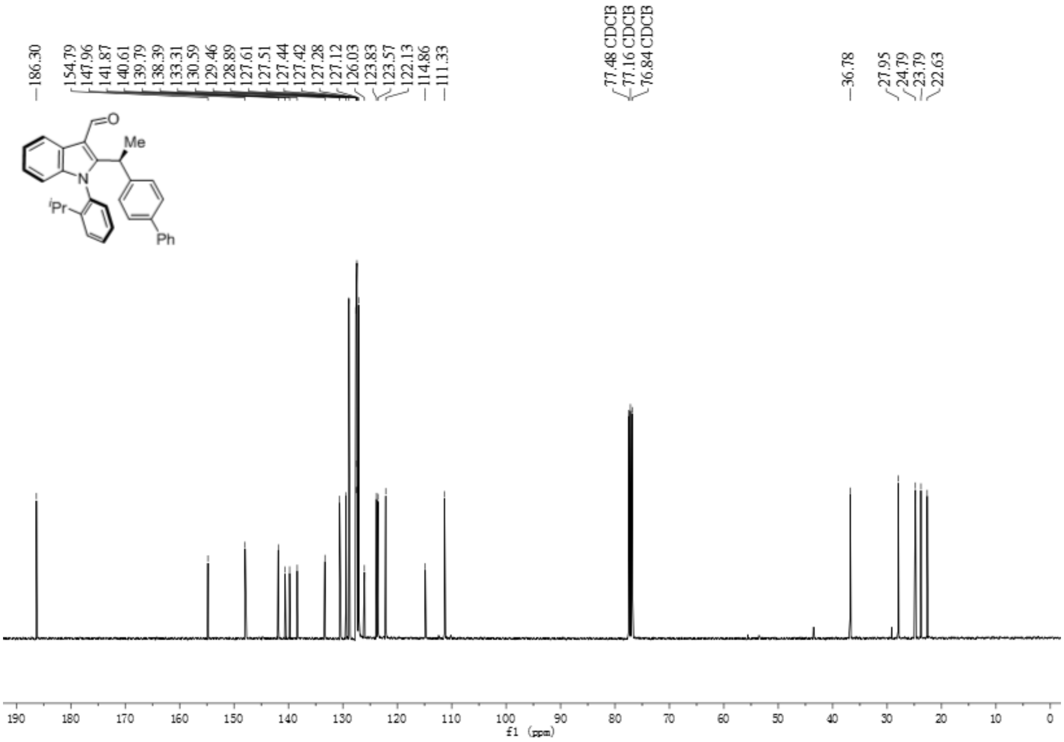

Supplementary Fig. 135. ^{1}H NMR spectrum of **25**

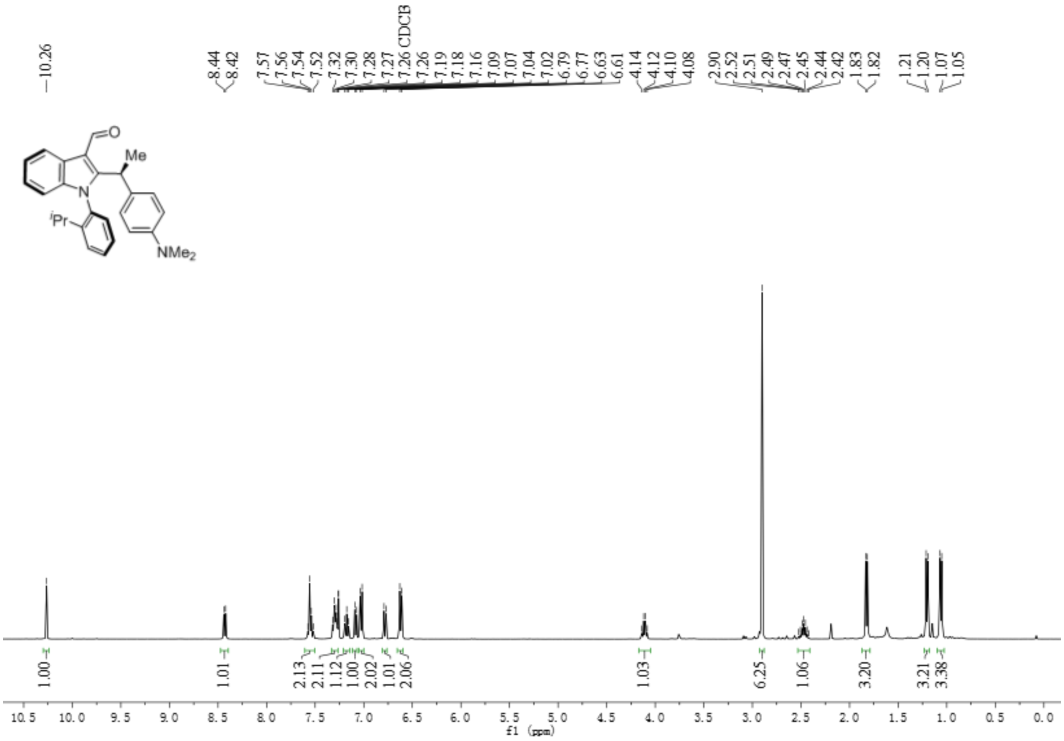

Supplementary Fig. 136. ^{13}C NMR spectrum of **25**

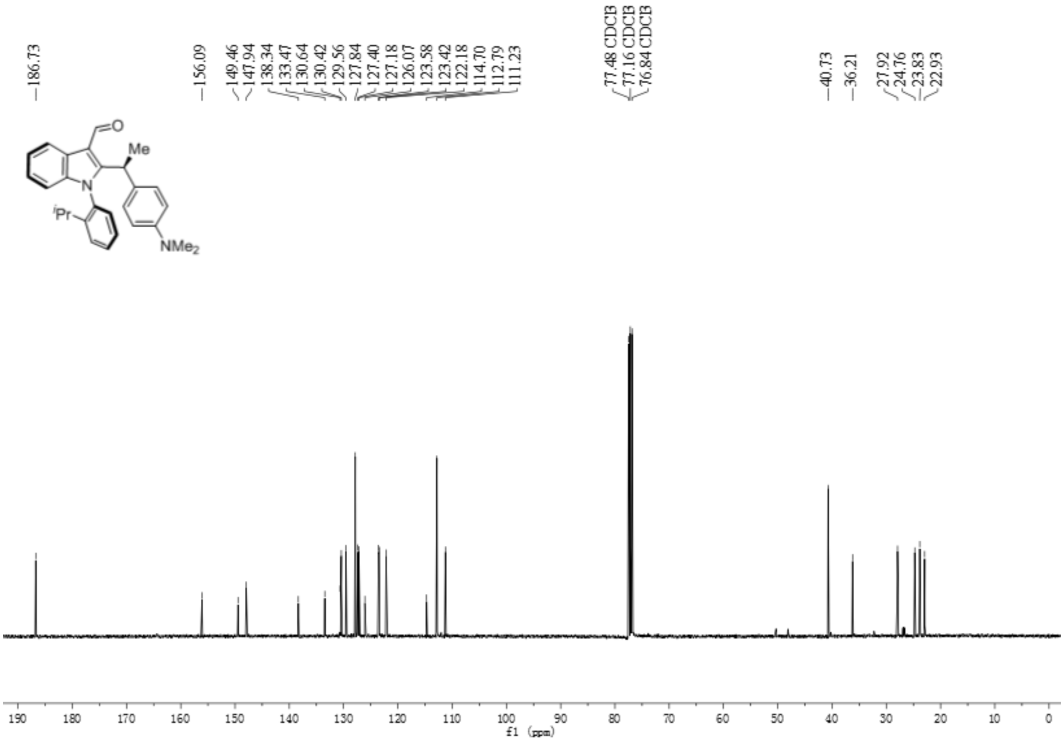

Supplementary Fig. 137. ^{1}H NMR spectrum of **26**

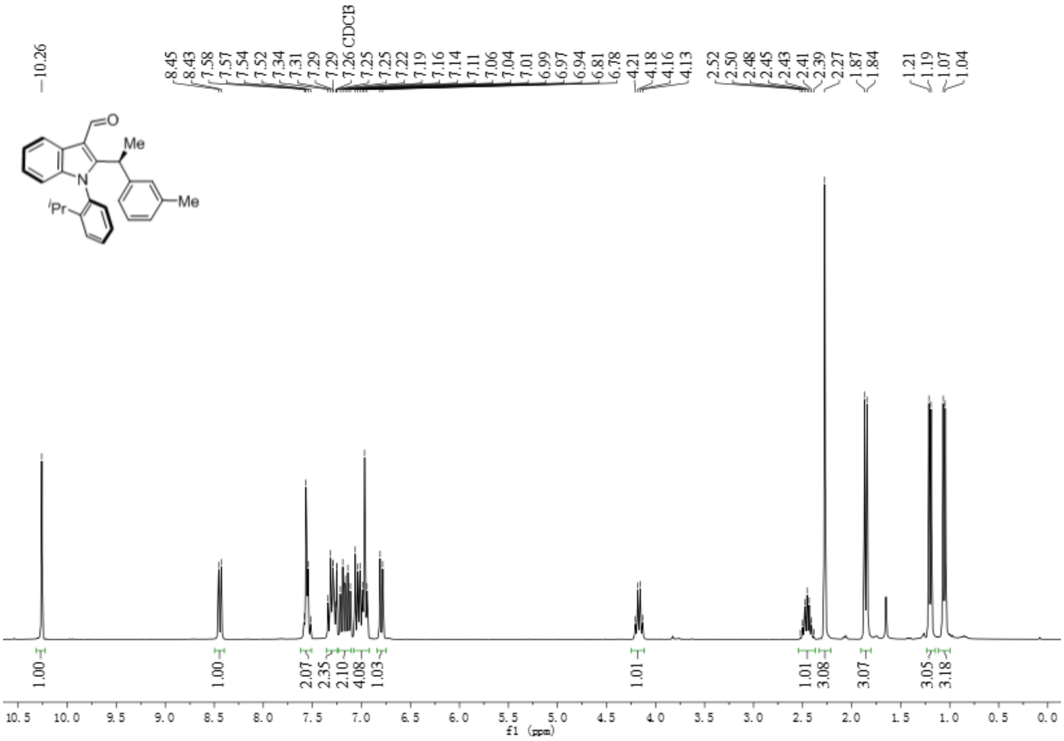

Supplementary Fig. 138. ^{13}C NMR spectrum of **26**

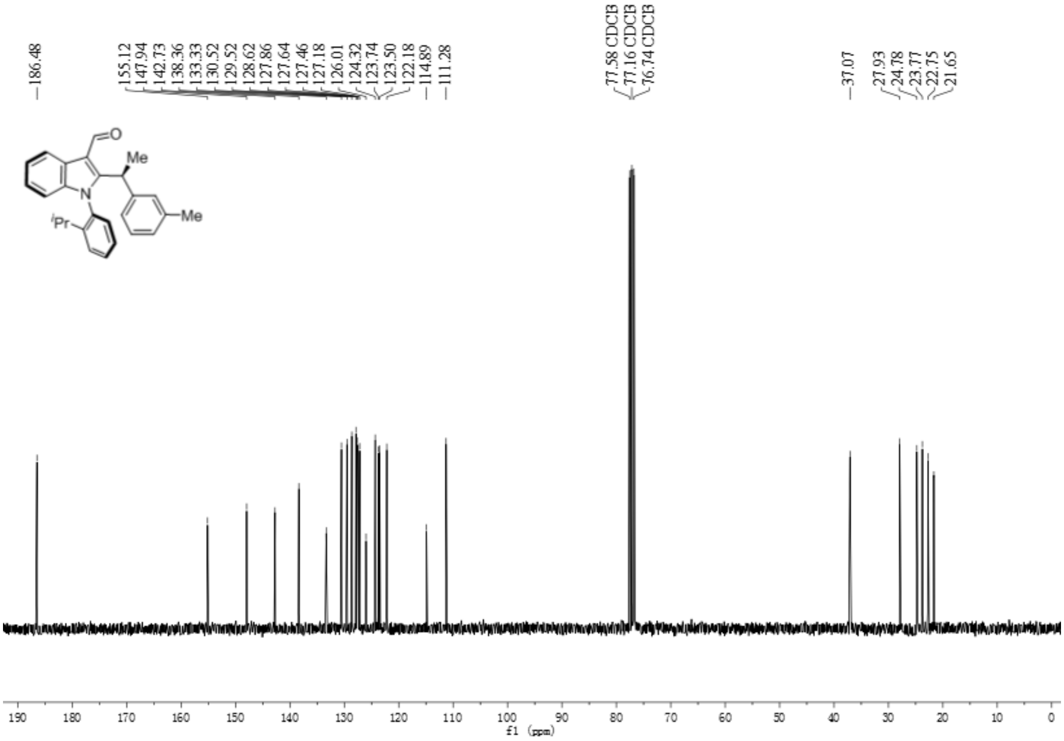

Supplementary Fig. 139. ^{1}H NMR spectrum of **27**

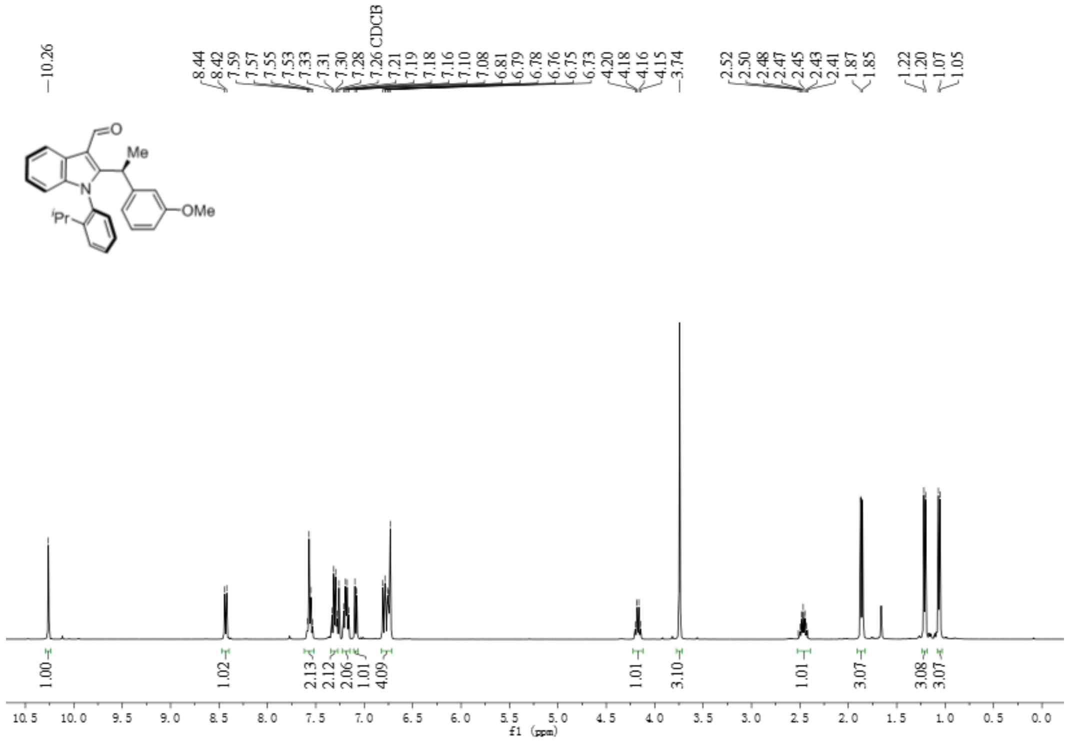

Supplementary Fig. 140. ^{13}C NMR spectrum of **27**

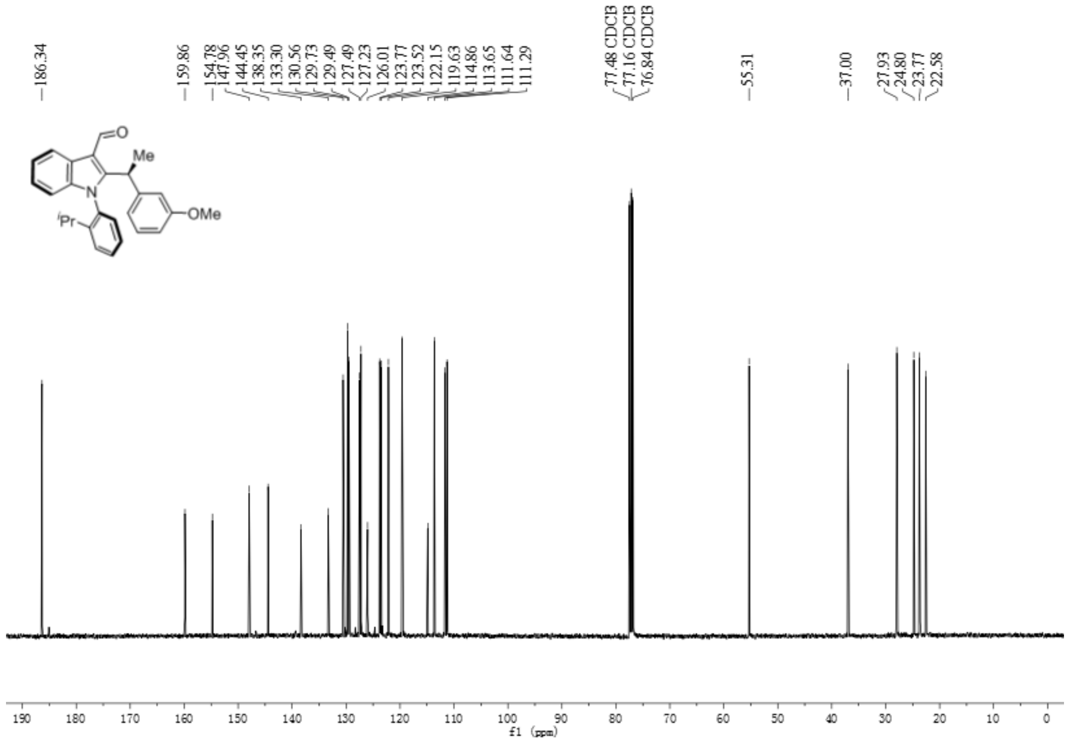

Supplementary Fig. 141. ^{1}H NMR spectrum of **28**

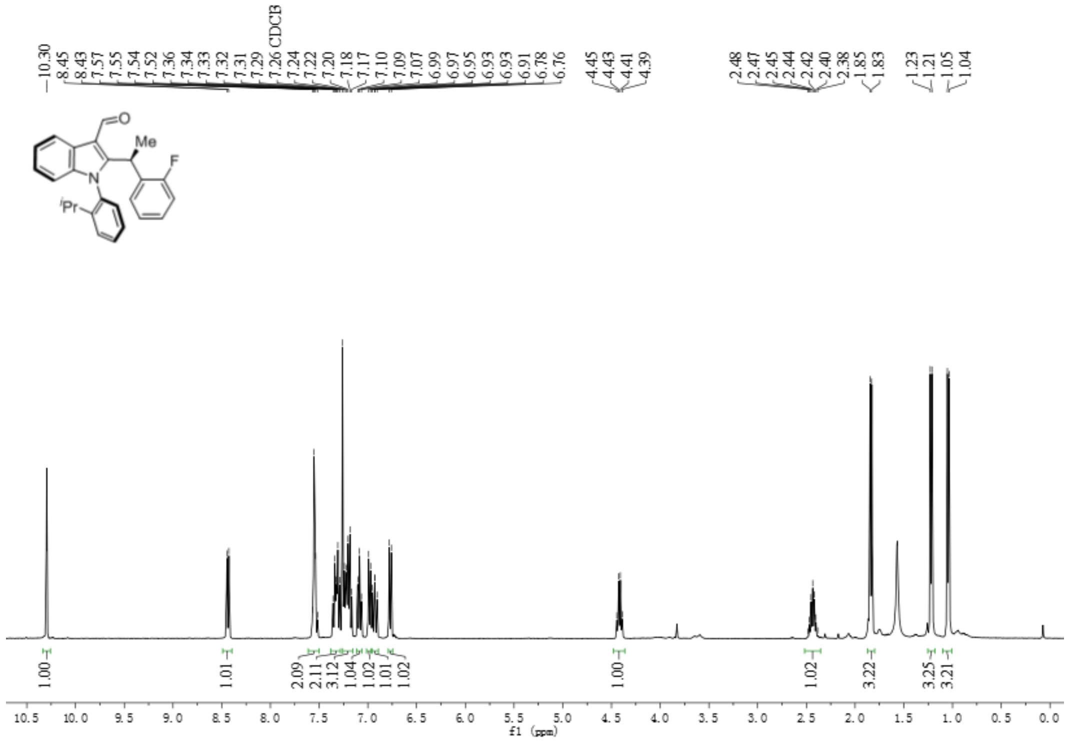

Supplementary Fig. 142. ^{13}C NMR spectrum of **28**

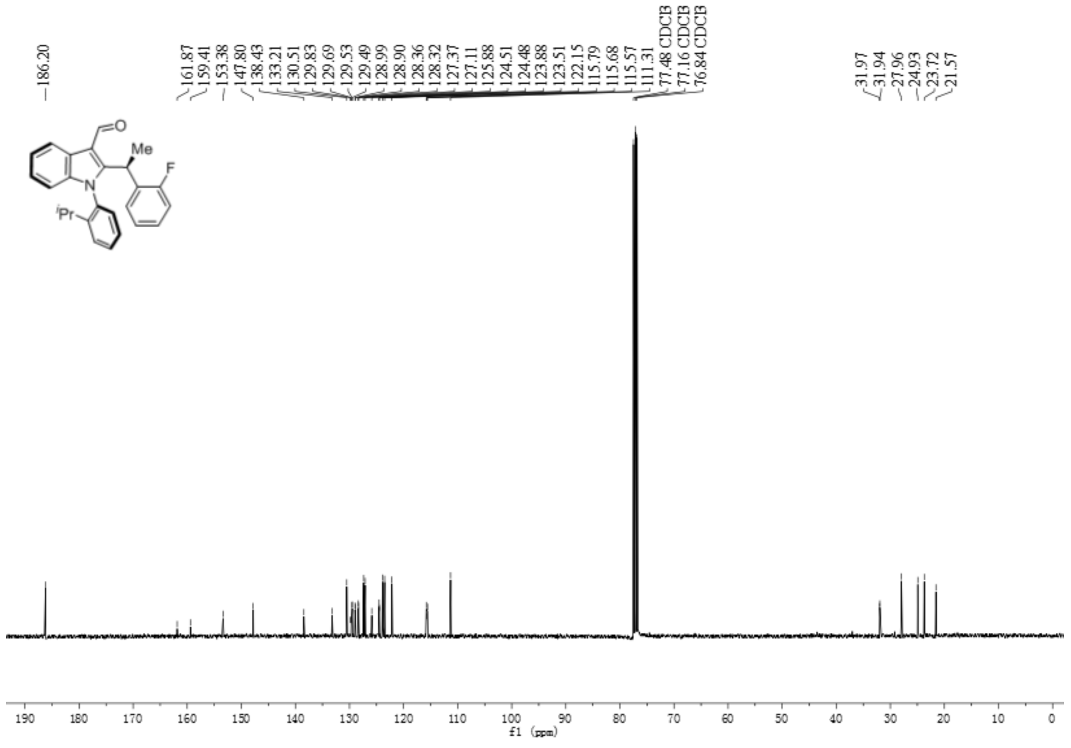

Supplementary Fig. 143. ^{19}F NMR spectrum of **28**

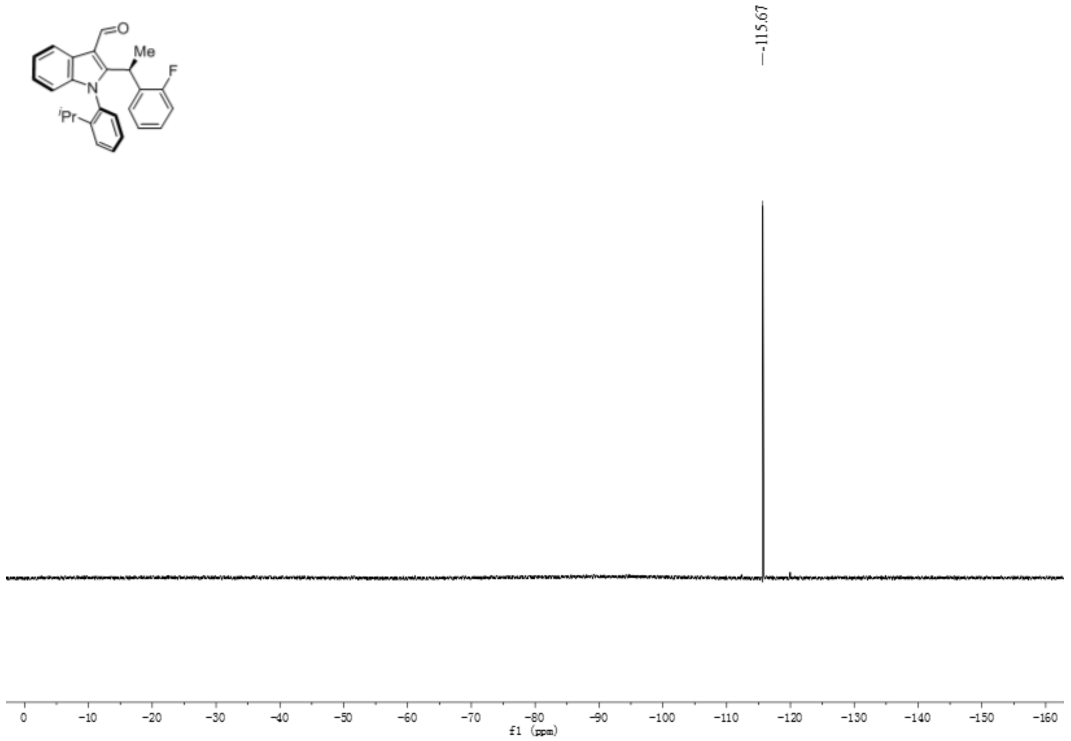

Supplementary Fig. 144. ^{1}H NMR spectrum of **29**

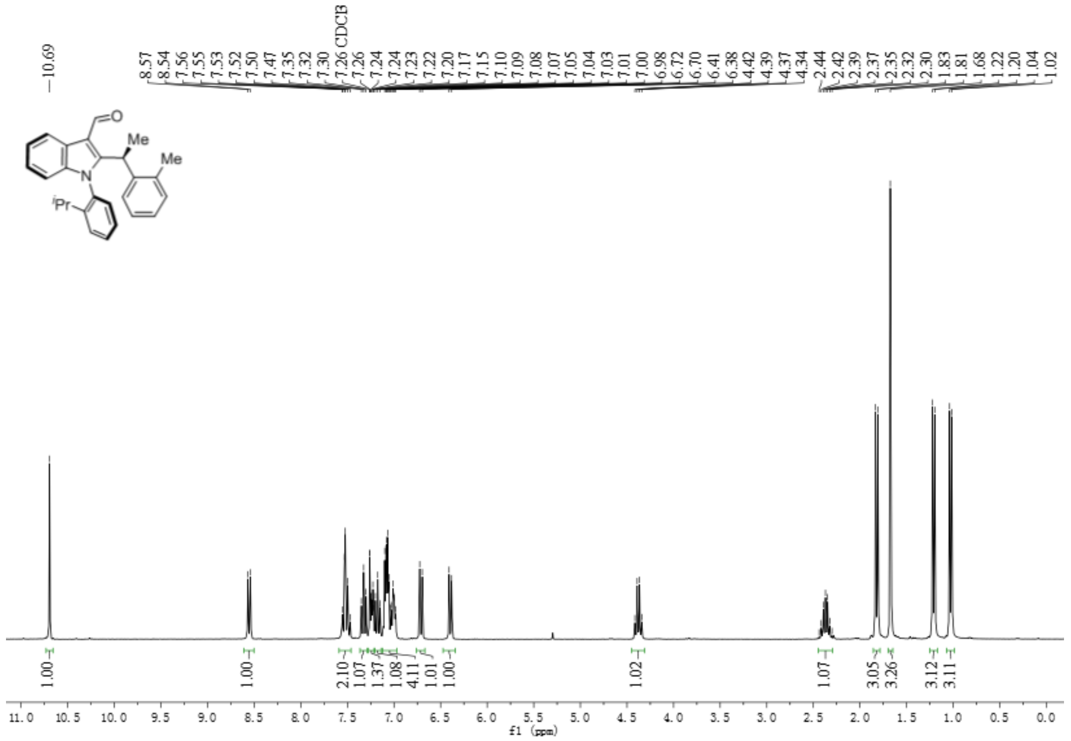

Supplementary Fig. 145. ^{13}C NMR spectrum of **29**

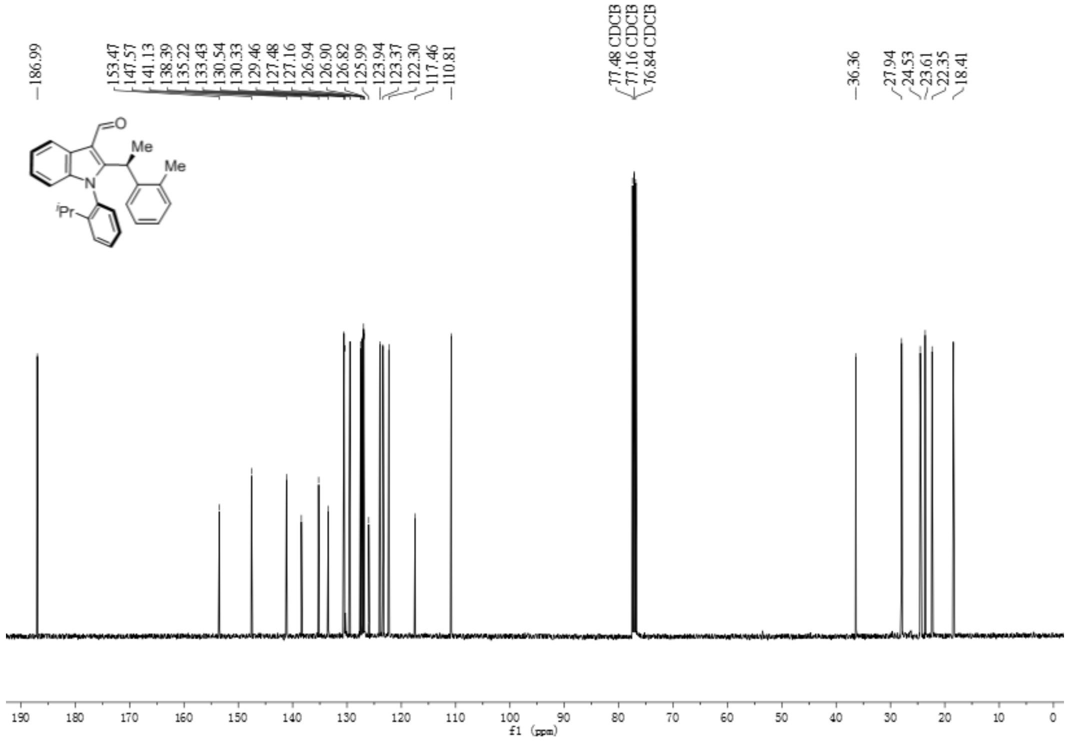

Supplementary Fig. 146. ^{1}H NMR spectrum of **30**

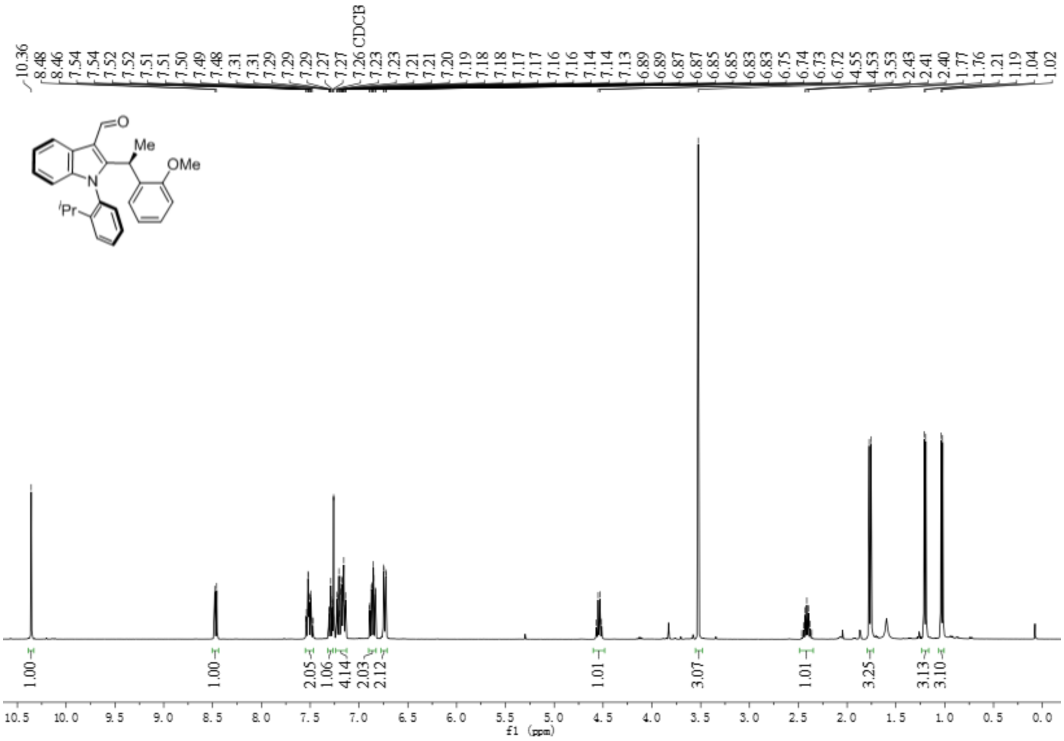

Supplementary Fig. 147. ^{13}C NMR spectrum of **30**

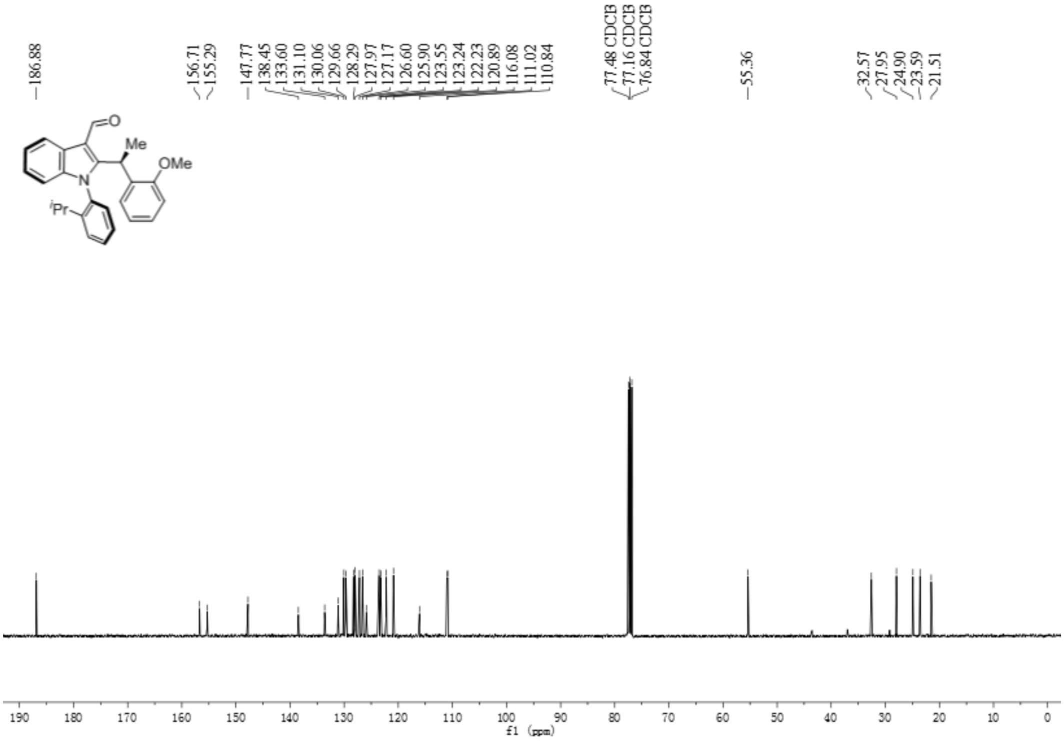

Supplementary Fig. 148. ^{1}H NMR spectrum of **31**

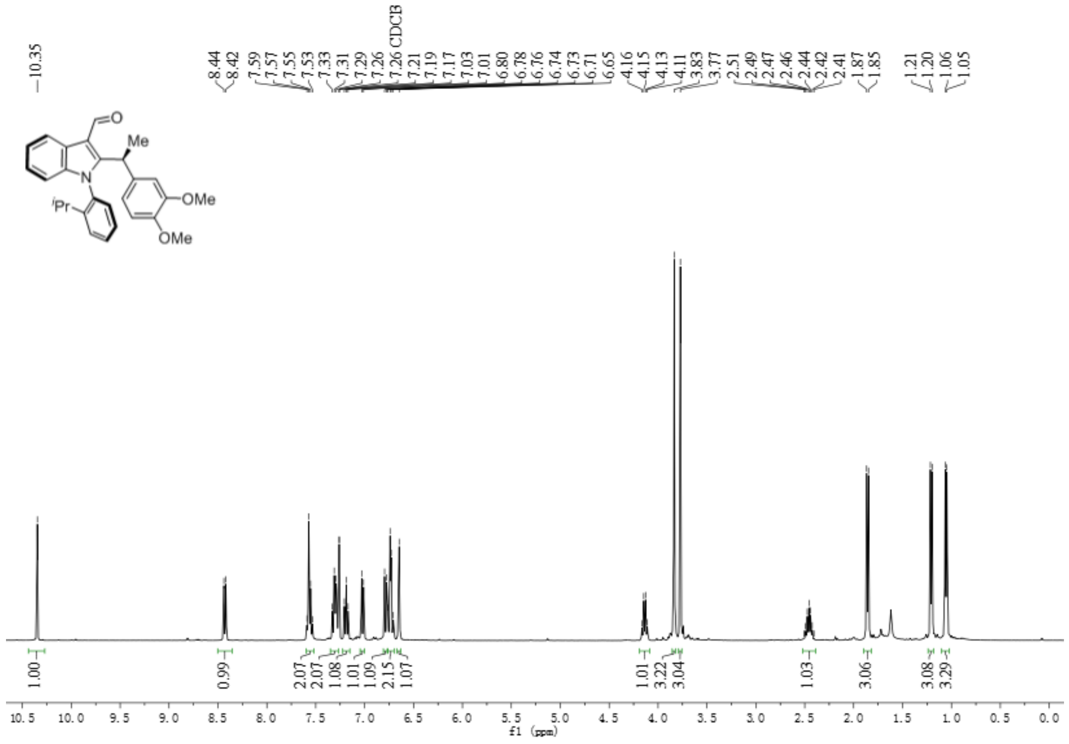

Supplementary Fig. 149. ^{13}C NMR spectrum of **31**

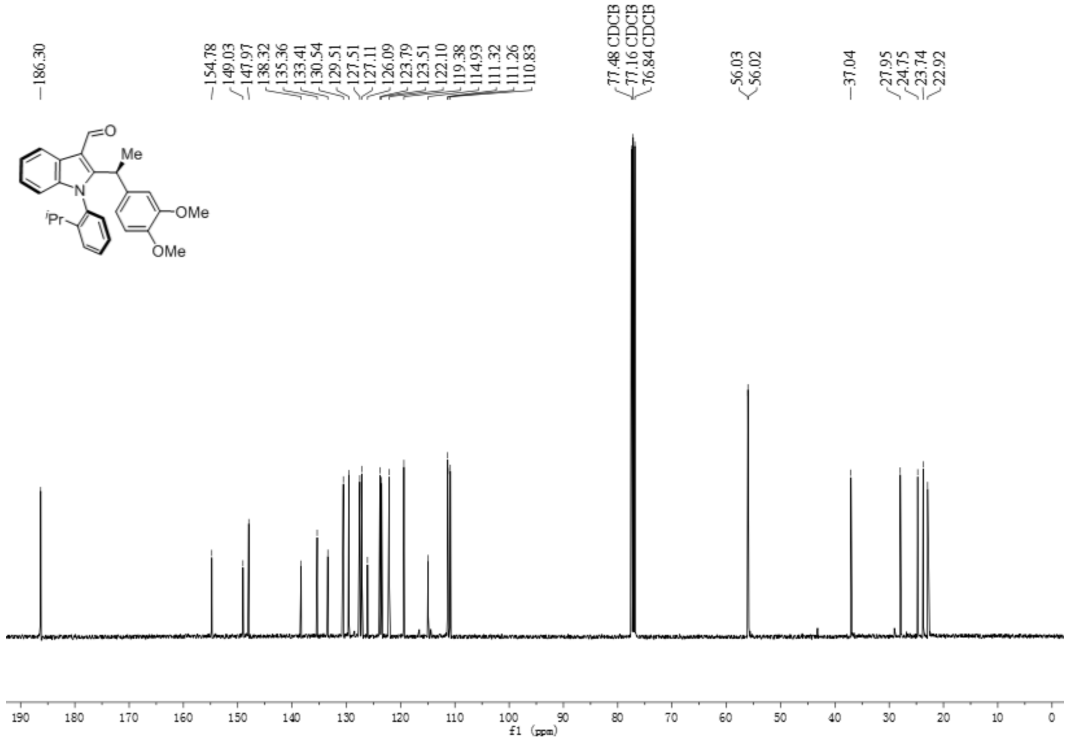

Supplementary Fig. 150. ^{1}H NMR spectrum of **32**

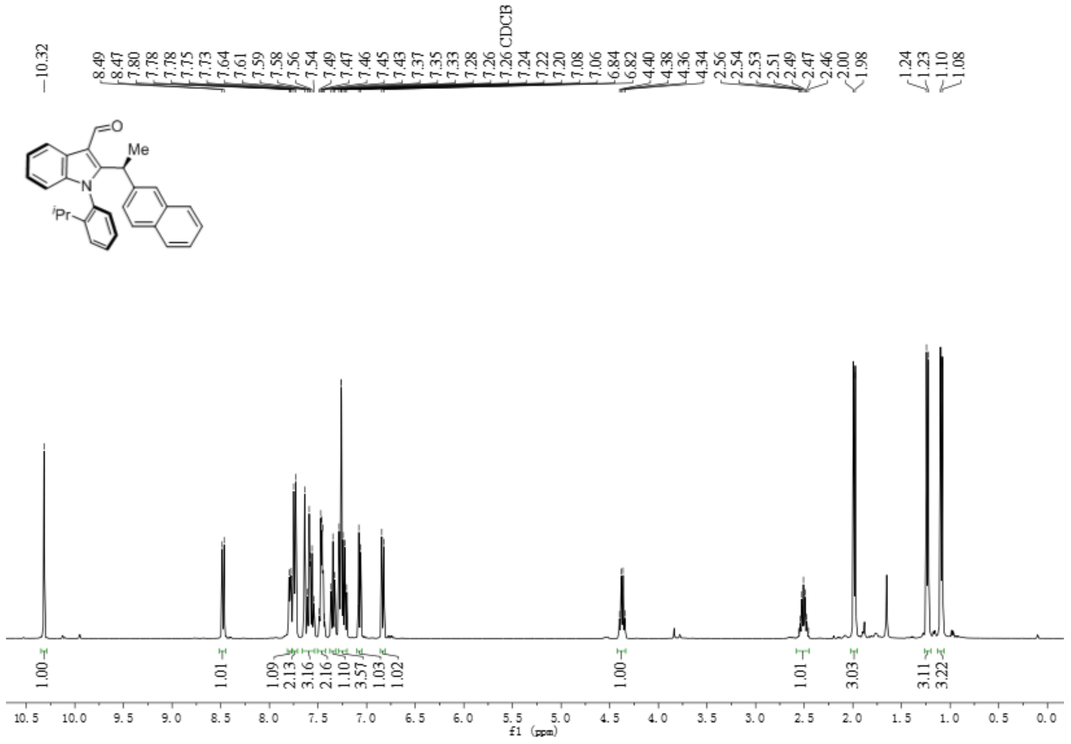

Supplementary Fig. 151. ^{13}C NMR spectrum of **32**

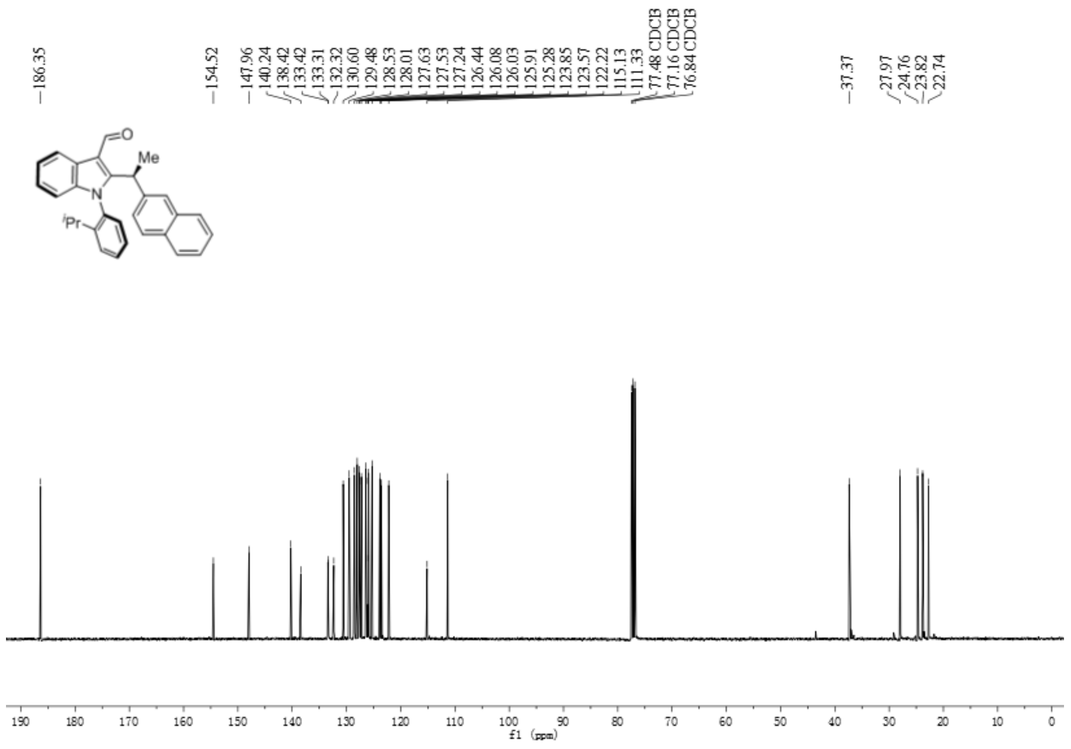

Supplementary Fig. 152. ^{1}H NMR spectrum of **33**

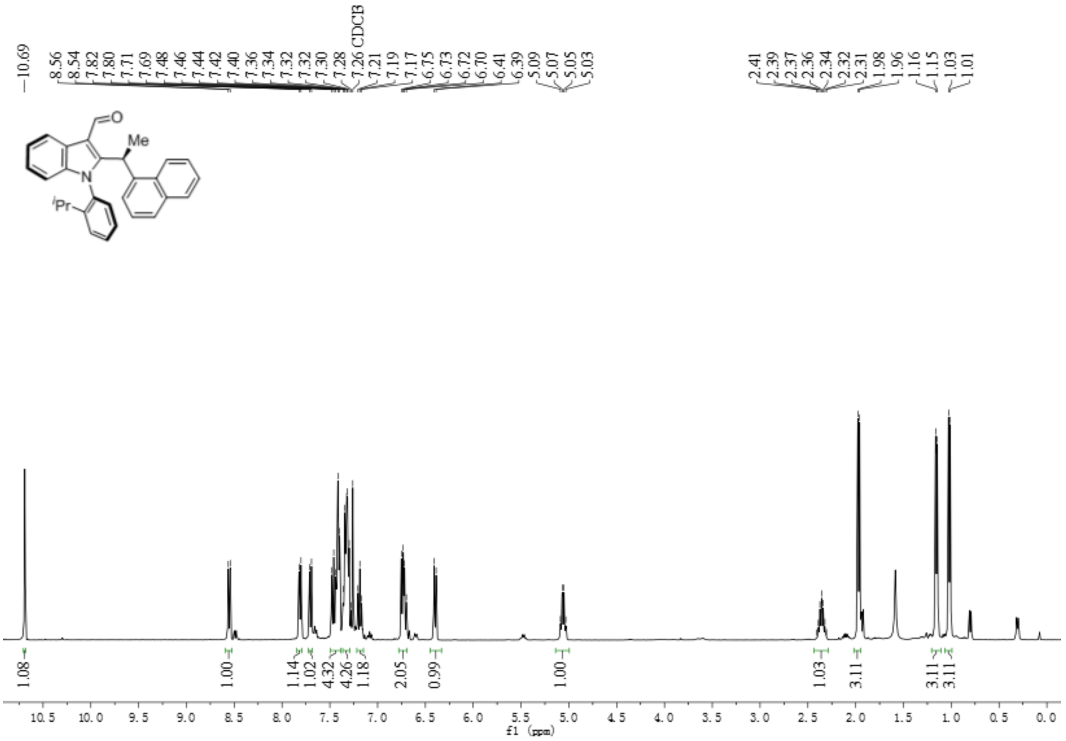

Supplementary Fig. 153. ^{13}C NMR spectrum of **33**

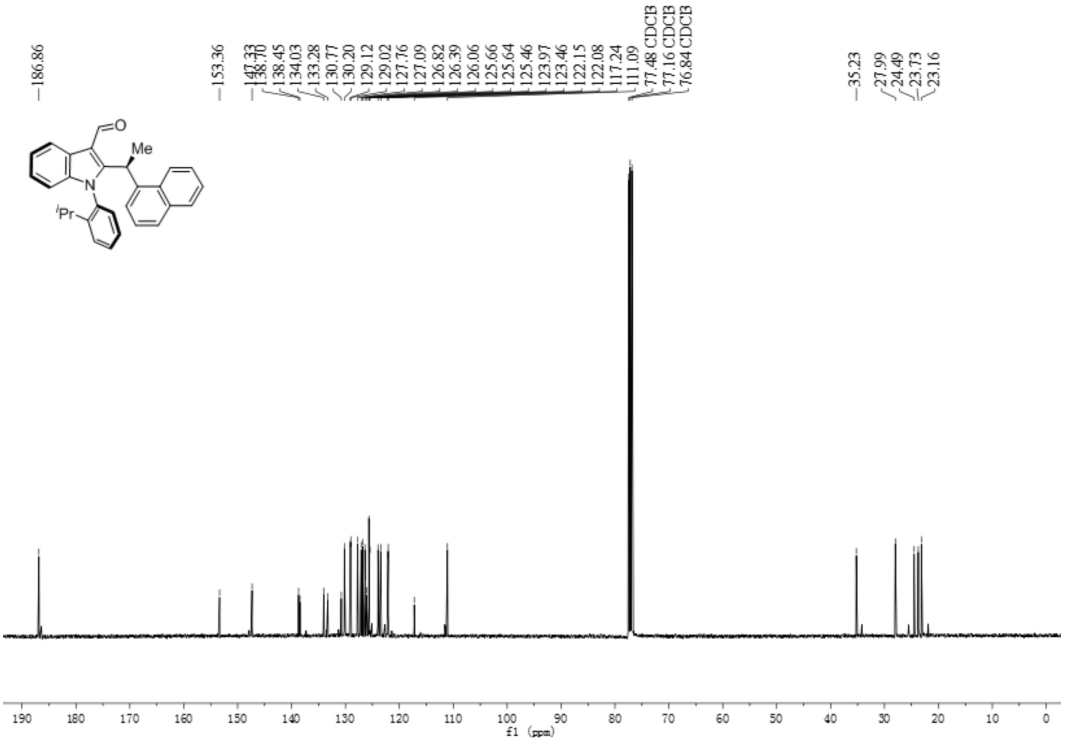

Supplementary Fig. 154. ^{1}H NMR spectrum of **34**

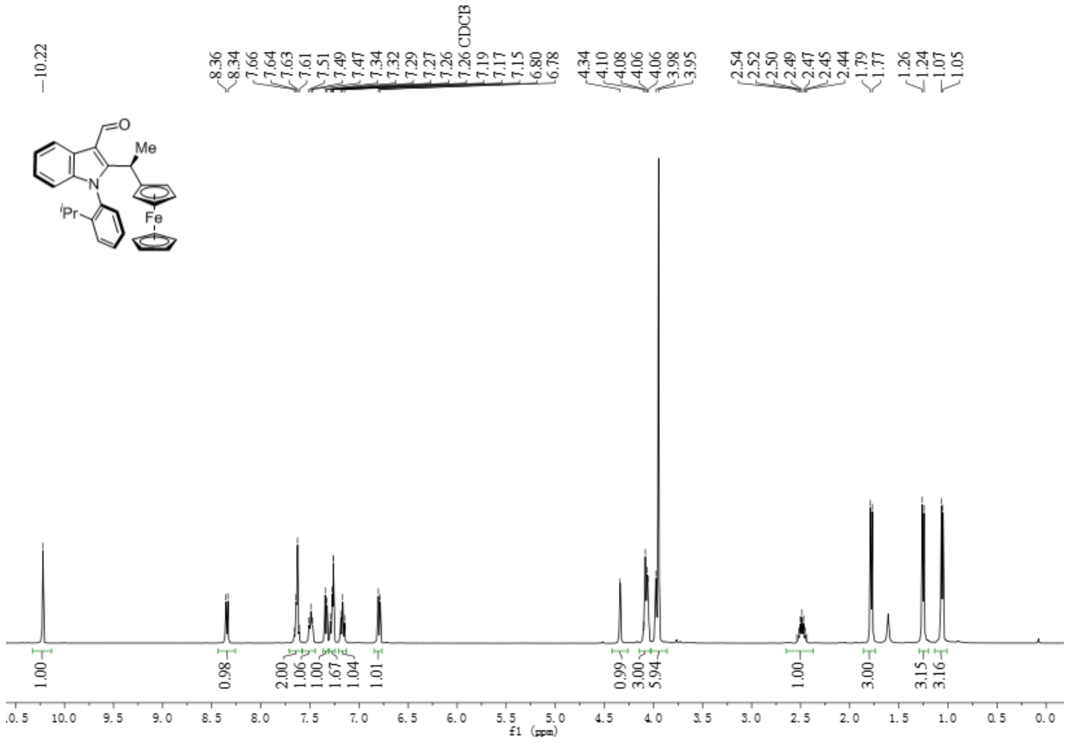

Supplementary Fig. 155. ^{13}C NMR spectrum of **34**

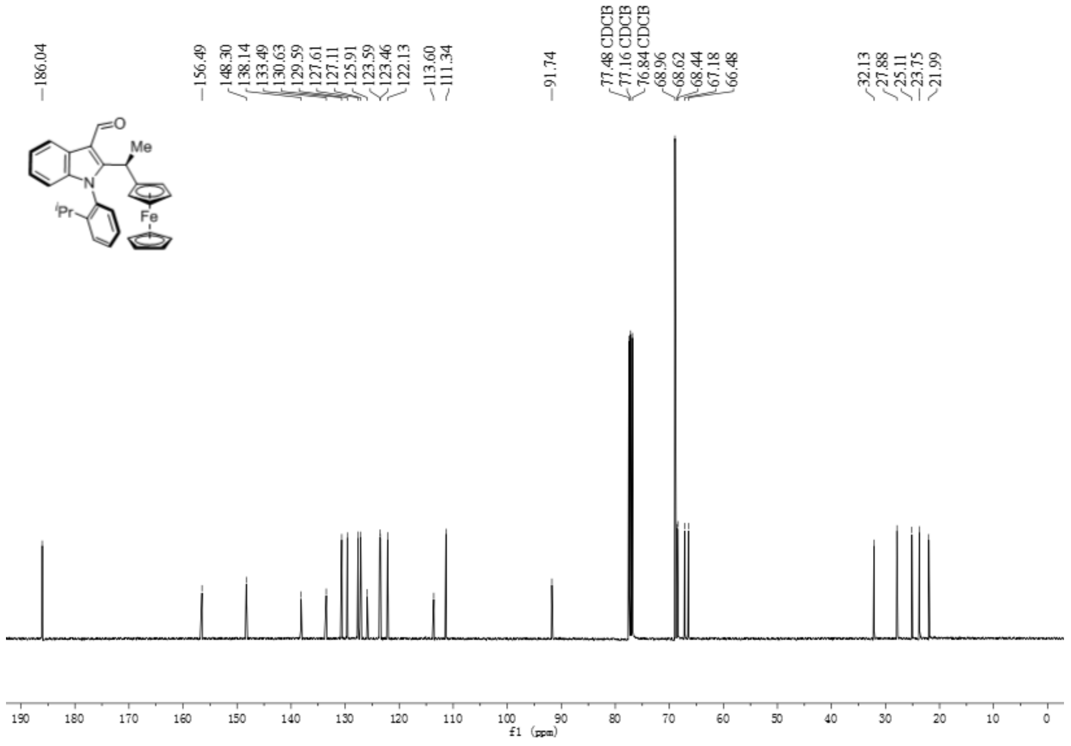

Supplementary Fig. 156. ^{1}H NMR spectrum of **35**

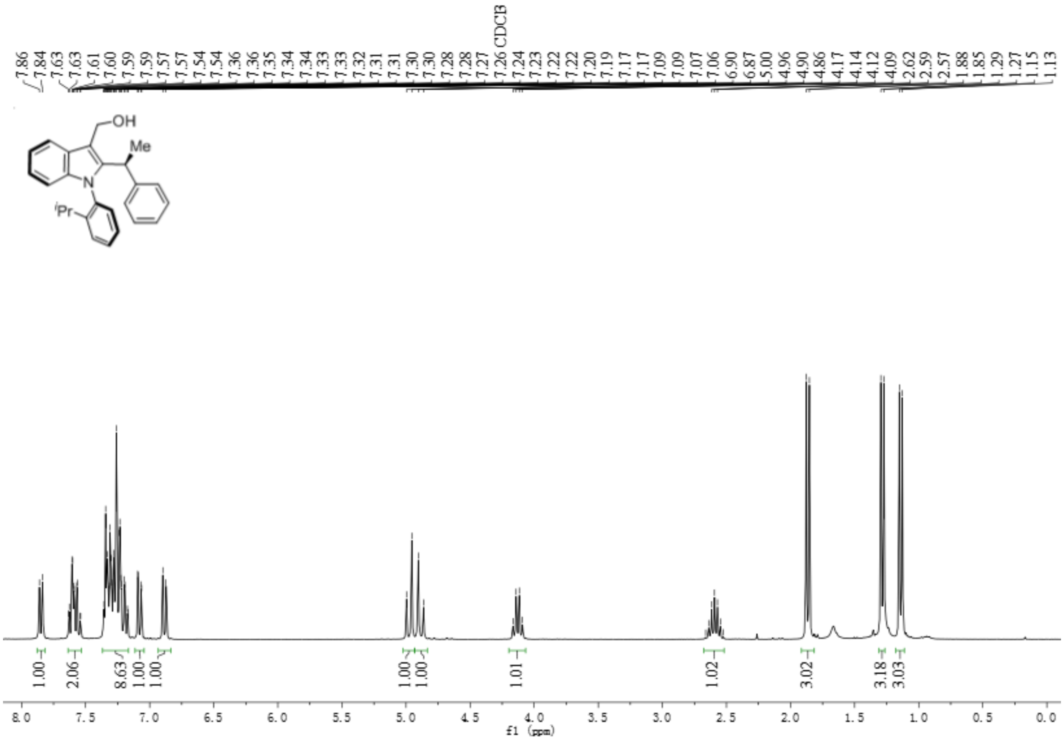

Supplementary Fig. 157. ^{13}C NMR spectrum of **35**

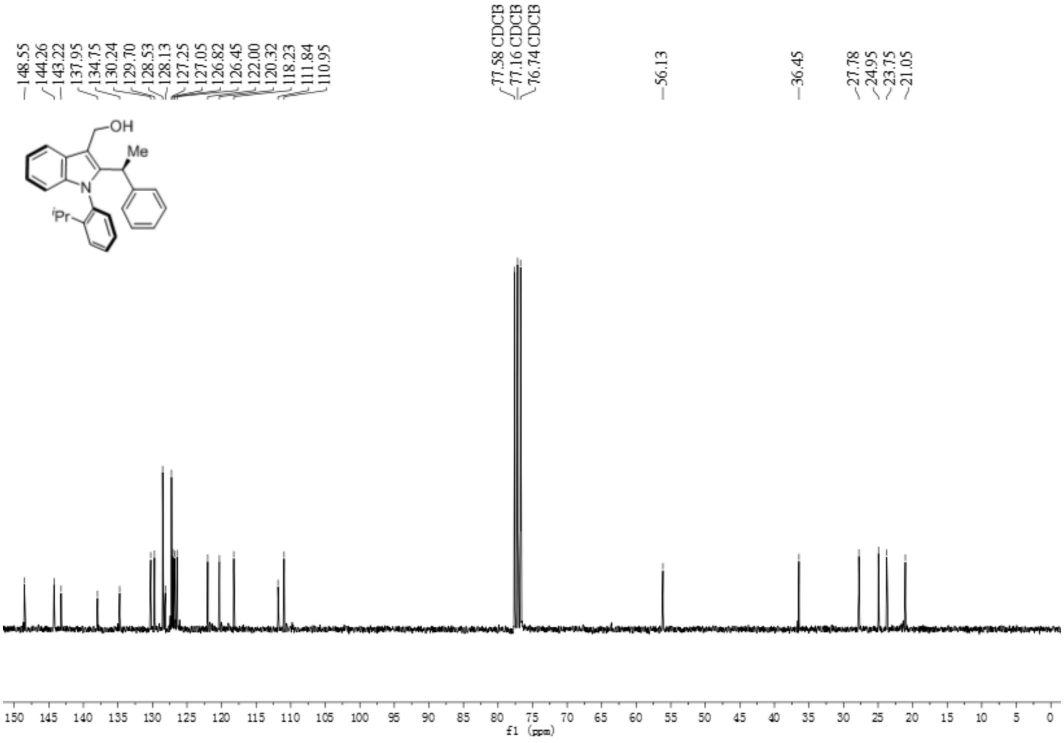

Supplementary Fig. 158. ^{1}H NMR spectrum of **36**

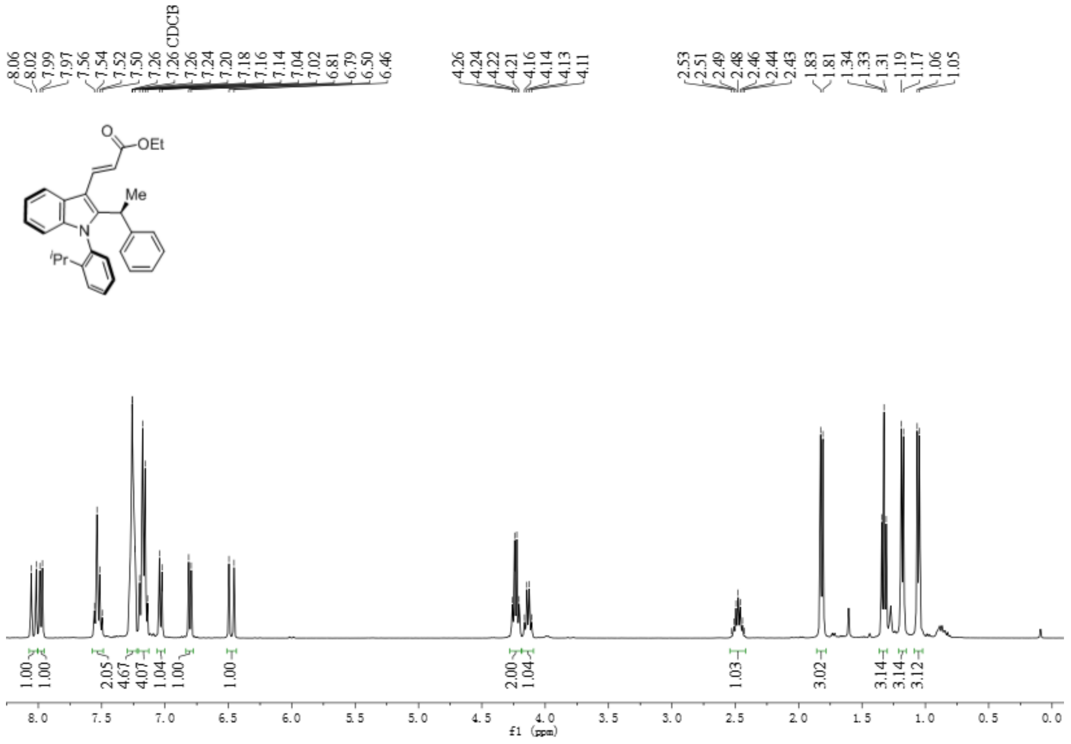

Supplementary Fig. 159. ^{13}C NMR spectrum of **36**

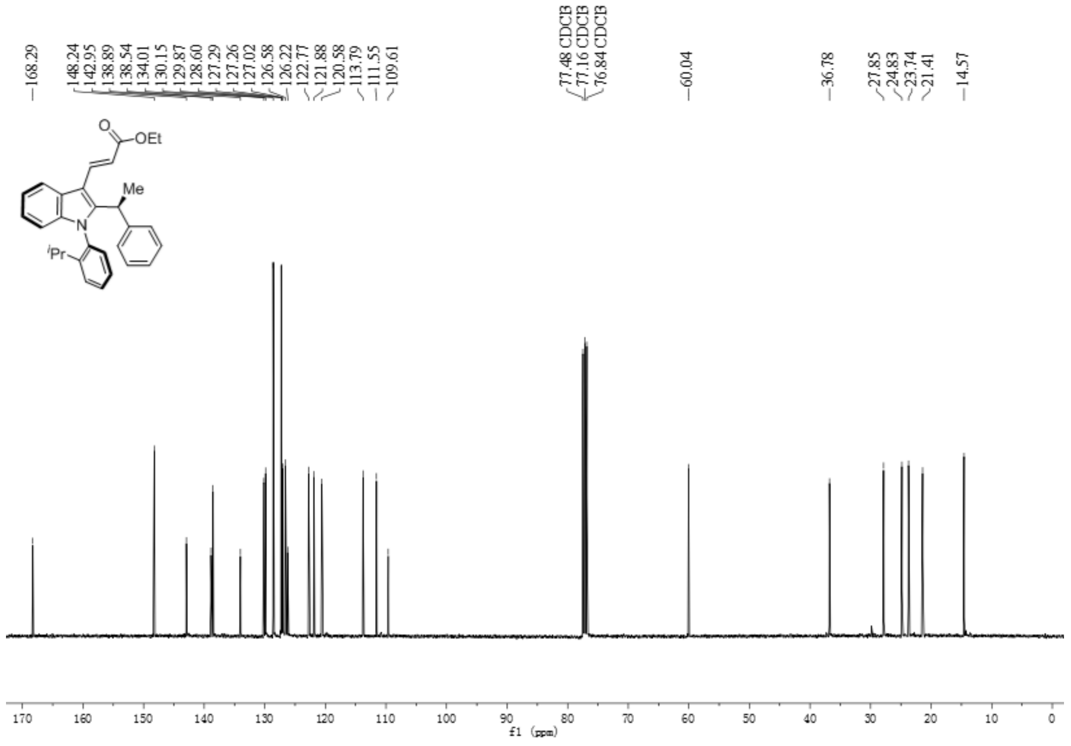

Supplementary Fig. 160. ^{1}H NMR spectrum of **37**

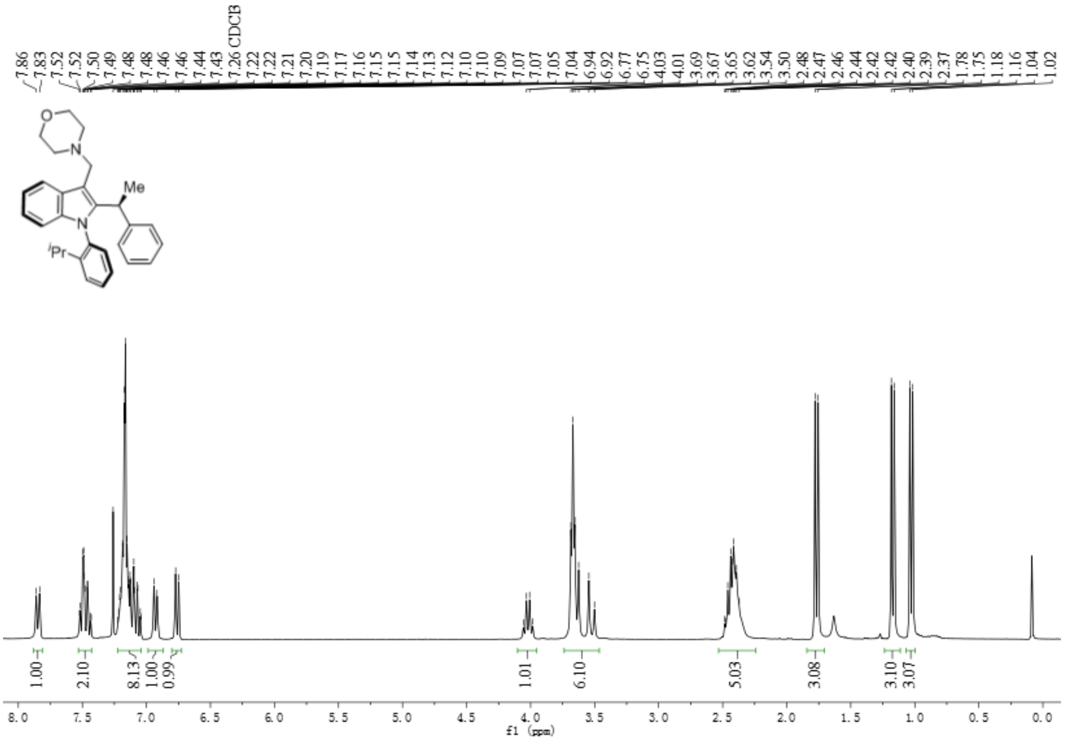

Supplementary Fig. 161. ^{13}C NMR spectrum of **37**

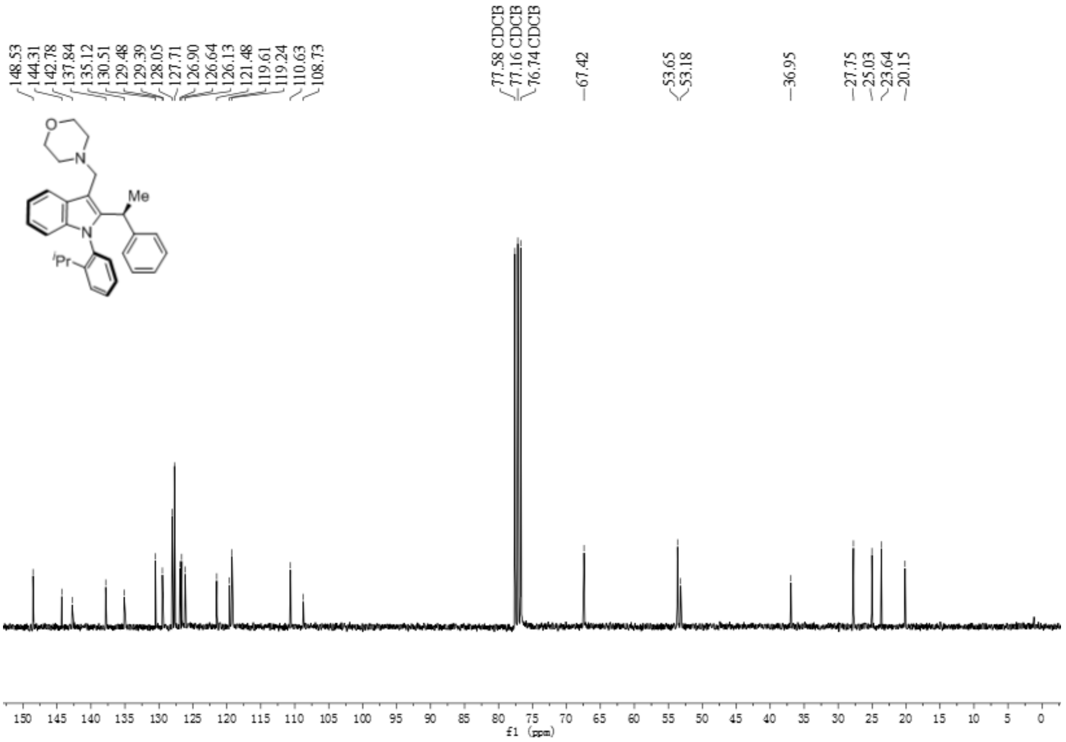

13. References

1. Loup, J. et al. Asymmetric iron-catalyzed C–H alkylation enabled by remote ligand *meta*-substitution. *Angew. Chem. Int. Ed.* **56**, 14197−14201 (2017).
2. Abadie, M.-A. et al. Development of chiral C_2-symmetric *N*-heterocyclic carbene Rh(I) catalysts through control of their steric properties. *Organometallics* **38**, 536−543 (2019).
3. Wang, H. et al. NHC ligands tailored for simultaneous regio- and enantiocontrol in nickel-catalyzed reductive couplings. *J. Am. Chem. Soc.* **139**, 9317−9324 (2017).
4. Jacob, N., Zaid, Y., Oliveira, J. C. A., Ackermann, L. & Wencel-Delord, J. Cobalt-catalyzed enantioselective C−H arylation of indoles. *J. Am. Chem. Soc.* **144**, 798−806 (2022).
5. Guillaneux, D., Zhao, S.-H., Samuel, O., Rainford, D. & Kagan, H. B. Nonlinear effects in asymmetric catalysis. *J. Am. Chem. Soc.* **116**, 9430−9439 (1994).
6. Girard, C. & Kagan, H. B. Nonlinear effects in asymmetric synthesis and stereoselective reactions: ten years of investigation. *Angew. Chem. Int. Ed.* **37**, 2922−2959 (1998).
7. Chen, W. et al. Formal Co(0), Fe(0), and Mn(0) complexes with NHC and styrene ligation. *Chin. Chem. Lett.* **31**, 1342−1344 (2020).
8. Gaussian 16, Revision A.03, Gaussian Inc.: Wallingford CT, 2016. Frisch, M. J., Trucks, G. W., Schlegel, H. B., Scuseria, G. E., Robb, M. A., Cheeseman, J. R., Scalmani, G., Barone, V., Petersson, G. A., Nakatsuji, H., Li, X., Caricato, M., Marenich, A. V., Bloino, J., Janesko, B. G., Gomperts, R., Mennucci, B., Hratchian, H. P., Ortiz, J. V., Izmaylov, A. F., Sonnenberg, J. L., Williams-Young, D., Ding, F., Lipparini, F., Egidi, F., Goings, J., Peng, B., Petrone, A., Henderson, T., Ranasinghe, D., Zakrzewski, V. G., Gao, J., Rega, N., Zheng, G., Liang, W., Hada, M., Ehara, M., Toyota, K., Fukuda, R., Hasegawa, J., Ishida, M., Nakajima, T., Honda, Y., Kitao, O., Nakai, H., Vreven, T., Throssell, K., Montgomery, J. A., Jr. Peralta, J. E., Ogliaro, F., Bearpark, M. J., Heyd, J. J., Brothers, E. N., Kudin, K. N., Staroverov, V. N., Keith, T. A., Kobayashi, R., Normand, J., Raghavachari, K., Rendell, A. P., Burant, J. C., Iyengar, S. S., Tomasi, J., Cossi, M., Millam, J. M., Klene, M., Adamo, C., Cammi, R., Ochterski, J. W., Martin, R. L., Morokuma, K., Farkas, O., Foresman, J. B., Fox. D. J.
9. Becke, A. D. Density-functional thermochemistry. III. The role of exact exchange. *J. Chem. Phys.* **98**, 5648−5652 (1993).
10. Lee C., Yang W. & Parr R. G. Development of the Colle-Salvetti correlation-energy formula into a functional of the electron density. *Phys. Rev. B: Condens. Matter Mater. Phys*. **37**, 785−789

(1988).

11. Grimme S., Ehrlich S. & Goerigk L. Effect of the damping function in dispersion corrected density functional theory. *J. Comp. Chem.* **32**, 1456−1465 (2011).
12. Weigend, F. & Ahlrichs, R. Balanced basis sets of split valence, triple zeta valence and quadruple zeta valence quality for H to Rn: Design and assessment of accuracy. *Phys. Chem. Chem. Phys.* **7**, 3297−305 (2005).
13. Weigend, F. Accurate Coulomb-fitting basis sets for H to Rn. *Phys. Chem. Chem. Phys.* **8**, 1057−1065 (2006).
14. Marenich, A. V., Cramer, C. J. & Truhlar, D. G. Universal solvation model based on solute electron density and on a continuum model of the solvent defined by the bulk dielectric constant and atomic surface tensions. *J. Phys. Chem. B* **113**, 6378−6396 (2009).
15. Legault, C. Y. CYLview, version 1.0b; Université de Sherbrooke, 2009 (http://www.cylview.org).
16. Humphrey, W., Dalke, A. & Schulten, K. VMD - Visual Molecular Dynamics. vol. *Molec. Graphics* **14**, 33−38 (1996).
17. Lu, T., Liu Z. & Chen, Q. Comment on "18 and 12 – Member carbon rings (cyclo[n]carbons) – A density functional study". *Mat. Sci. Eng. B*, **273**, 115425 (2021).
18. Lu, T. & Chen, F. Multiwfn: a multifunctional wavefunction analyzer. *J. Comput. Chem.* **33**, 580−592 (2012).
19. Neese, F. The ORCA program system. *Comput. Mol. Sci.* **2**, 73−78 (2012).
20. Becke, A. D. Density-functional exchange-energy approximation with correct asymptotic behavior. *Phys. Rev. A* **38**, 3098−3100 (1988).
21. Perdew J. P. Density-functional approximation for the correlation energy of the inhomogeneous electron gas. *Phys. Rev. B* **33**, 8822−8824 (1986).
22. Neese, F. Prediction and interpretation of the ^{57}Fe isomer shift in Mössbauer spectra by density functional theory. *Inorg. Chim. Acta* **337**, 181−192 (2002).
23. Sinnecker, S., Slep, L. D., Bill, E. & Neese, F. Performance of nonrelativistic and quasi-relativistic hybrid DFT for the prediction of electric and magnetic hyperfine parameters in ^{57}Fe Mössbauer spectra. *Inorg, Chem.* **44**, 2245–2254 (2005).
24. Bjornsson, R., Neese, F. & DeBeer, S. Revisiting the Mössbauer isomer shifts of the FeMoco cluster of nitrogenase and the cofactor charge. *Inorg. Chem.* **56**, 1470−1477 (2017).
25. McWilliams, S. F., Brennan-Wydra, E., Macleod, K. C. & Holland, P. L. Density functional

calculations for prediction of ^{57}Fe Mössbauer isomer shifts and quadrupole splittings in β-diketiminate complexes. *ACS Omega* **2**, 2594−2606 (2017).

26. *CrysAlisPro*, version 171.41.120a; Rigaku Corporation: Oxford, UK (2021).
27. Sheldrick, G. M. *SHELXT*, version 2018/2; *Acta. Crystallogr.* **A71**, 3-8 (2015).
28. Sheldrick, G. M. *SHELXL*, version 2018/3; *Acta. Crystallogr.* **C71**, 3-8 (2015).
29. Dolomanov, O. V., Bourhis, L. J., Gildea, R. J., Howard, J. A. K. & Puschmann, H. *Olex2*, version 1.3-ac4; *J. Appl. Cryst.* **42**, 339-341 (2009).
