# Supplementary material for: Iron-catalyzed stereoselective C–H alkylation for simultaneous construction of C–N axial and C-central chirality
Source: Nat Commun. 2024 Apr 25;15:3503. doi: 10.1038/s41467-024-47589-7 (PMC11045758; doi:10.1038/s41467-024-47589-7)
Supplement: Supplementary file 3 — Description of Additional Supplementary Files [file 41467_2024_47589_MOESM3_ESM.pdf]

Supplementary Data 1

Cartesian coordinates and atomic coordinates.
