# Supplementary material for: Iron-catalyzed stereoselective C–H alkylation for simultaneous construction of C–N axial and C-central chirality
Source: Nat Commun. 2024 Apr 25;15:3503. doi: 10.1038/s41467-024-47589-7 (PMC11045758; doi:10.1038/s41467-024-47589-7)
Supplement: Supplementary file 4 — Supplementary Data 1 [file 41467_2024_47589_MOESM4_ESM.pdf]

Supplementary Data

Cartesian coordinates and atomic coordinates

Table of Contents

1. Cartesian coordinates of the computed structures..2

2. Atomic coordinates of **neijd09**...125

3. Atomic coordinates of **neijd10**...148

1. Cartesian coordinates of the computed structures

int1(singlet)

C	0.43127400	-2.27148600	-0.33338000
C	-0.98436400	-1.87965400	-0.82955200
H	0.35084700	-2.99782100	0.48635200
H	-1.20172000	-2.36235500	-1.79357300
N	-0.84629300	-0.41760100	-1.01477300
N	0.89160700	-0.98155600	0.19119200
C	-2.02792300	-2.28222400	0.19780000
C	-2.72026900	-3.49027800	0.04758500
C	-2.25618300	-1.49705400	1.33272100
C	-3.62653600	-3.91322700	1.02377900
H	-2.55155300	-4.10394600	-0.84239600
C	-3.16660200	-1.91372600	2.30515200
H	-1.72522100	-0.55562400	1.45801200
C	-3.84953400	-3.12452900	2.15586700
H	-4.16513300	-4.85513800	0.89438500
H	-3.35301300	-1.27646000	3.17076300
H	-4.56666000	-3.44559600	2.91511000
C	1.39536300	-2.79401000	-1.37787000
C	2.14850300	-3.94521100	-1.12356800
C	1.61649200	-2.08501500	-2.57011600
C	3.10447300	-4.39062700	-2.04277800
H	1.99356700	-4.49136700	-0.18927800
C	2.56649200	-2.52973300	-3.48927400
H	1.05637600	-1.16840200	-2.76685400
C	3.31394600	-3.68416200	-3.22812000
H	3.69132600	-5.28643600	-1.82658300
H	2.73392900	-1.96669600	-4.41040200
H	4.06552900	-4.02422000	-3.94422000
C	-1.91847400	0.37083800	-1.46275200
C	-1.82446500	1.77484300	-1.64300700
C	-3.16124900	-0.22952800	-1.71158800
C	-2.96862800	2.49657500	-1.98263700
C	-4.32103300	0.48246700	-2.04412700
H	-3.23703200	-1.30376800	-1.59707100
C	-4.20196200	1.86804800	-2.17019300
H	-2.91236000	3.57617600	-2.10278300
H	-5.06272600	2.48655600	-2.42061500
C	0.24321400	0.08720700	-0.33093800
Fe	0.86739600	1.80329900	-0.17020400
C	2.00599500	-0.89082400	1.05998600
C	3.29256400	-0.70451600	0.56080800

C	1.79888900	-1.05835400	2.44456100
C	4.41459000	-0.64974900	1.39490800
H	3.38837800	-0.59724600	-0.51324400
C	2.90643300	-0.98757000	3.29989700
C	4.18462600	-0.78198100	2.77219100
H	2.78200600	-1.09038200	4.37766600
H	5.02636800	-0.72080200	3.46640200
O	-0.60003500	2.39768400	-1.50069400
O	0.52502200	-1.28559100	2.83525200
C	-0.50248400	3.78391400	-1.82742700
H	-0.83578300	3.95413700	-2.86241100
H	-1.10159700	4.39377200	-1.13291800
H	0.55215300	4.05436800	-1.72467700
C	0.23825100	-1.47434400	4.20193300
H	0.76356800	-2.35675200	4.60839800
H	0.51210200	-0.58800400	4.80037900
H	-0.84556000	-1.62968700	4.26857900
C	-5.63825000	-0.25892700	-2.26087800
H	-5.61702400	-0.64090300	-3.29942400
C	-6.89844100	0.63331600	-2.12746300
C	-5.82323800	-1.48376300	-1.32438600
H	-6.80652600	1.50730200	-2.79082300
C	-7.07559100	1.09871500	-0.66922600
C	-8.14189500	-0.16820900	-2.55391900
H	-4.95114500	-2.14966000	-1.39204500
C	-5.98979600	-1.01435800	0.13050800
C	-7.06890100	-2.27732800	-1.75336700
H	-7.96169100	1.75197600	-0.59485300
H	-6.20852600	1.69569100	-0.34645200
C	-7.23915800	-0.12564000	0.24740600
H	-8.04112400	-0.49131900	-3.60409000
H	-9.03402100	0.47858500	-2.50080800
C	-8.31895300	-1.39133200	-1.64008100
H	-5.09477000	-0.46778500	0.46249600
H	-6.08041500	-1.88981500	0.79238700
H	-6.95167700	-2.63744500	-2.78995000
H	-7.17457900	-3.16910300	-1.11256000
H	-7.35709800	0.20757000	1.29223800
C	-8.48271100	-0.92403900	-0.18258300
H	-9.21067100	-1.96122700	-1.95083000
H	-8.61993200	-1.79587000	0.47972300
H	-9.38760800	-0.29985900	-0.08512200
C	5.82171600	-0.41423600	0.86793800
H	6.15757900	0.53842000	1.31736600

C	5.94393500	-0.25188500	-0.66733500
C	6.83139500	-1.50397300	1.32032400
H	5.22480900	0.50776000	-1.01256000
C	5.69719400	-1.58550700	-1.40225000
C	7.37239900	0.22449500	-0.99345800
H	6.74321700	-1.65615700	2.40785100
C	6.54399700	-2.82953200	0.59384800
C	8.25798200	-1.03190000	0.99116200
H	5.79859200	-1.42560200	-2.48888700
H	4.67854200	-1.96074900	-1.24068800
C	6.70722300	-2.63941200	-0.92478100
H	7.56882300	1.19302000	-0.50370800
H	7.46919800	0.38938900	-2.07969800
C	8.39883600	-0.82361200	-0.52589000
H	5.52122300	-3.17238600	0.82243300
H	7.23577600	-3.60861700	0.95710700
H	8.47655300	-0.09246500	1.52675600
H	8.99019300	-1.78005700	1.34010900
H	6.51014800	-3.59477800	-1.43984400
C	8.13473200	-2.16082900	-1.24094300
H	9.41802900	-0.47240600	-0.75893900
H	8.87217700	-2.91484000	-0.91553700
H	8.25781200	-2.03904000	-2.33069400
C	-0.51534600	2.31816800	1.37388200
C	-0.84499100	1.79262300	2.59220600
H	0.51393700	2.80926800	1.29707100
H	-1.31959000	2.78547900	0.79723600
H	-0.04157400	1.47850500	3.26549900
C	2.27675600	1.70740900	-1.60859600
C	2.77303300	2.51478800	-0.53330700
H	2.71113600	0.71900000	-1.78940800
H	1.96632600	2.19129600	-2.54343200
H	3.36295300	1.99951400	0.23935100
C	-2.20153400	1.55565500	3.06188400
C	-3.33775100	1.70565600	2.22888900
C	-2.42737400	1.12996000	4.39097600
C	-4.62082400	1.46367900	2.71047900
H	-3.21012200	1.98848800	1.18238200
C	-3.71392600	0.88238300	4.86941400
H	-1.56897600	1.00487200	5.05614400
C	-4.82328900	1.04915400	4.03314900
H	-5.47553000	1.58696500	2.04191700
H	-3.85309300	0.55903700	5.90448400
H	-5.83212300	0.85372300	4.40343100

C	2.97814200	3.96998400	-0.56039400
C	3.28175500	4.67095500	0.63070100
C	2.89671000	4.73275900	-1.74716700
C	3.45805000	6.05052900	0.64113500
H	3.36011600	4.10557400	1.56415600
C	3.06186400	6.12026500	-1.73350600
H	2.72746500	4.22259900	-2.69789900
C	3.33767300	6.79438800	-0.54174200
H	3.68777800	6.55754600	1.58249500
H	2.99108900	6.67819400	-2.67174600
H	3.47021900	7.87863100	-0.53269100

int1(triplet)

C	0.86360200	-1.68200300	0.66162700
C	-0.64032000	-1.96392300	0.44597700
H	1.12218800	-1.76083600	1.72531000
H	-0.79246100	-2.96594800	0.02387000
N	-0.96003900	-0.93843500	-0.56681800
N	0.93164900	-0.25803500	0.25065900
C	-1.45048500	-1.81498700	1.72127500
C	-1.97433900	-2.95037600	2.35137900
C	-1.66726200	-0.55249700	2.29418800
C	-2.70792400	-2.83231900	3.53568200
H	-1.81849600	-3.93560600	1.90305600
C	-2.39659100	-0.43628200	3.47810400
H	-1.27467700	0.34438800	1.81421500
C	-2.91990000	-1.57314400	4.10238400
H	-3.12026500	-3.72545600	4.01115900
H	-2.56734500	0.55477600	3.90435100
H	-3.49882400	-1.47678700	5.02390500
C	1.75878000	-2.59443300	-0.14802600
C	2.60423400	-3.49991600	0.50191600
C	1.72516200	-2.57606100	-1.55120200
C	3.41576500	-4.37026600	-0.23141200
H	2.63713100	-3.51521500	1.59436400
C	2.53275100	-3.44671000	-2.28282200
H	1.07047700	-1.87410800	-2.06876300
C	3.38339200	-4.34351600	-1.62688300
H	4.07889200	-5.06422700	0.29010000
H	2.50151500	-3.42408300	-3.37471900
H	4.01964800	-5.01851800	-2.20405800
C	-2.11722600	-1.04216700	-1.37016500
C	-2.08026600	-0.72735400	-2.74617200
C	-3.32236200	-1.46718000	-0.80179600

C	-3.26675100	-0.80155500	-3.48480500
C	-4.52099600	-1.51643000	-1.51805400
H	-3.31515800	-1.71595900	0.25234200
C	-4.46453200	-1.18032300	-2.87739800
H	-3.26505100	-0.55792100	-4.54624100
H	-5.36974100	-1.21456100	-3.48709900
C	-0.11547800	0.13641100	-0.50628400
Fe	-0.49396600	1.89447700	-1.41516300
C	1.99436400	0.56156300	0.70803300
C	3.31573400	0.18558700	0.46871300
C	1.71626500	1.71757700	1.46376000
C	4.40285000	0.91888500	0.96099600
H	3.48110400	-0.69029800	-0.15113200
C	2.79098100	2.48045800	1.93417300
C	4.10601900	2.07892500	1.68719600
H	2.60946600	3.39119700	2.50341500
H	4.91427300	2.70579000	2.06778000
O	-0.88374300	-0.35106000	-3.27533400
O	0.41383600	2.01603300	1.68557200
C	-0.82092000	0.01996900	-4.63814400
H	-1.10582900	-0.81739700	-5.29791200
H	-1.46855100	0.88761700	-4.84697200
H	0.22193000	0.29984800	-4.83182800
C	0.09744600	3.04654800	2.60096600
H	0.58469500	2.87256800	3.57624100
H	0.39605000	4.03426100	2.21397900
H	-0.98959200	3.03241800	2.72073500
C	-5.84598800	-1.89750700	-0.87382600
H	-6.18133100	-2.82328800	-1.37737800
C	-6.95135500	-0.83109900	-1.11125200
C	-5.79599000	-2.20142200	0.64455600
H	-6.99242600	-0.57896100	-2.18220100
C	-6.64693000	0.44004400	-0.29915100
C	-8.30950200	-1.41026800	-0.68006700
H	-5.00628400	-2.94316500	0.84707000
C	-5.53566000	-0.92864000	1.47566500
C	-7.15643500	-2.78495500	1.07240000
H	-7.41036500	1.20703300	-0.51221100
H	-5.67569900	0.86431900	-0.60179400
C	-6.63496000	0.10849300	1.20362000
H	-8.54214300	-2.30999600	-1.27507100
H	-9.10881900	-0.67647400	-0.87951800
C	-8.27463500	-1.75766800	0.81713200
H	-4.55346100	-0.49506500	1.24880700

H	-5.51106800	-1.19386800	2.54526900
H	-7.36325600	-3.71458800	0.51529000
H	-7.12499600	-3.05095600	2.14249100
H	-6.42699100	1.02533000	1.77979700
C	-7.99525900	-0.47556700	1.62214000
H	-9.24456100	-2.18488500	1.12245600
H	-7.99276500	-0.69915700	2.70277000
H	-8.79793700	0.26114200	1.44731500
C	5.84362100	0.54675800	0.63580800
H	6.16208200	1.25136400	-0.15165300
C	6.03390700	-0.87786100	0.06271300
C	6.82617600	0.72366100	1.82316200
H	5.34919500	-1.02829500	-0.78421400
C	5.77478600	-1.93834800	1.14808300
C	7.47799300	-1.02429000	-0.44746600
H	6.71042200	1.72903400	2.25595900
C	6.55238400	-0.33477100	2.90484600
C	8.26937600	0.57390100	1.31056000
H	5.88994300	-2.94540400	0.71427600
H	4.73967300	-1.87083100	1.51347500
C	6.75595500	-1.74116900	2.31467100
H	7.67119500	-0.28480600	-1.24310700
H	7.61328500	-2.02287800	-0.89685100
C	8.46855900	-0.82812300	0.71248200
H	5.52337800	-0.22808000	3.28545100
H	7.23184700	-0.17887800	3.76022600
H	8.48055200	1.34465000	0.54982900
H	8.97889100	0.73664000	2.13986200
H	6.56408000	-2.49991700	3.09225700
C	8.19950100	-1.88379300	1.79996200
H	9.50232500	-0.93132600	0.34182500
H	8.91397400	-1.75973900	2.63195000
H	8.35525400	-2.89674100	1.39052300
C	-2.25330900	2.65066600	-2.15020600
C	-2.52695100	2.12959400	-0.85800900
H	-2.54243900	2.03860900	-3.01226000
H	-2.29881100	3.72601000	-2.34120900
H	-3.01494500	1.15707700	-0.78223500
C	0.41980800	3.27034400	-2.59937400
C	1.39809000	2.74098600	-1.70585500
H	0.04171700	4.28772600	-2.45888600
H	0.43541600	2.94564800	-3.64823300
H	1.64689700	3.36391200	-0.83668100
C	-2.69564600	2.97563200	0.32809400

C	-2.22729700	4.30670100	0.39084800
C	-3.38445600	2.47857500	1.45571000
C	-2.46671700	5.10445600	1.50951100
H	-1.66998400	4.71713900	-0.45316000
C	-3.61856600	3.27481900	2.57684300
H	-3.75633000	1.45340200	1.43331200
C	-3.16594100	4.59932900	2.61188100
H	-2.09981200	6.13444300	1.52346400
H	-4.16938600	2.86083500	3.42617100
H	-3.35495600	5.22803200	3.48501600
C	2.54121700	1.92617000	-2.18611800
C	3.85530400	2.40246200	-2.01693800
C	2.37717500	0.69764800	-2.84985400
C	4.95282700	1.69414700	-2.50666400
H	4.01023200	3.34975300	-1.49495300
C	3.47398700	-0.01938100	-3.33159600
H	1.37204400	0.28847100	-2.94341600
C	4.77106600	0.47411800	-3.16546000
H	5.96027900	2.09745200	-2.37525700
H	3.31630600	-0.97984900	-3.82732300
H	5.63071900	-0.08644900	-3.54023200

int2(singlet)

C	2.53287900	-2.56244600	0.12277800
C	1.19426800	-3.17713600	-0.32966900
H	2.77581300	-2.88827600	1.14227900
H	1.35211800	-3.89611000	-1.14428500
N	0.48062300	-1.99329000	-0.85933500
N	2.19700300	-1.12042100	0.15895500
C	0.40888700	-3.86889100	0.76773800
C	0.18592500	-5.24811900	0.70957900
C	-0.13915900	-3.13519400	1.83134100
C	-0.56764300	-5.89270200	1.69675100
H	0.59495400	-5.82425000	-0.12509500
C	-0.89500300	-3.77414300	2.81373500
H	0.00952200	-2.05758800	1.88805300
C	-1.11174200	-5.15613000	2.75064200
H	-0.73799200	-6.97018900	1.63383900
H	-1.32418700	-3.17926700	3.62299400
H	-1.70942800	-5.65476400	3.51761400
C	3.72788100	-2.80380600	-0.77770900
C	5.01430400	-2.68061300	-0.23358100
C	3.59580300	-3.07216600	-2.14759200
C	6.14807900	-2.82692800	-1.03570100

H	5.12661300	-2.45914400	0.83054000
C	4.72926000	-3.24388800	-2.94566500
H	2.60561300	-3.13021000	-2.60259200
C	6.00799500	-3.11899000	-2.39480400
H	7.14160700	-2.71845500	-0.59533500
H	4.61215300	-3.46468900	-4.00963800
H	6.89238300	-3.24547100	-3.02373400
C	-0.73180200	-2.23295800	-1.56272000
C	-0.71280400	-2.39894700	-2.96164900
C	-1.91028900	-2.48050700	-0.86083400
C	-1.90154600	-2.74922300	-3.61286000
C	-3.10377400	-2.87081500	-1.48360500
H	-1.86895700	-2.38535500	0.21865400
C	-3.07089100	-2.98437600	-2.87884000
H	-1.92603400	-2.85758500	-4.69673200
H	-3.96846900	-3.26940100	-3.42827900
C	1.03462200	-0.80933700	-0.47660400
Fe	0.43302500	0.94480600	-0.57137000
C	2.98770800	-0.24822600	0.95365500
C	3.95778300	0.58292900	0.39401300
C	2.89218200	-0.36514600	2.36068300
C	4.82991400	1.34847400	1.19076400
H	4.04776000	0.59019000	-0.69034300
C	3.69427000	0.44677100	3.16235900
C	4.63419300	1.30028800	2.57535400
H	3.59666900	0.41552500	4.24711800
H	5.23725800	1.91966400	3.23707400
O	0.48401200	-2.22091600	-3.58183700
O	2.02892600	-1.29248000	2.83248100
C	0.52923900	-2.18990400	-4.99353000
H	0.25679800	-3.16618200	-5.43110700
H	-0.13988900	-1.41110900	-5.39706400
H	1.56500100	-1.94606500	-5.26320400
C	2.12630500	-1.71511700	4.17826900
H	3.15338100	-2.04549700	4.41326000
H	1.82147700	-0.92406700	4.87817500
H	1.43366400	-2.56008100	4.28080500
C	-4.31855600	-3.24128900	-0.63419100
H	-4.14292200	-4.28340500	-0.30518800
C	-5.67117400	-3.22158300	-1.38661400
C	-4.47252600	-2.38506400	0.65136700
H	-5.59680600	-3.83413900	-2.29923400
C	-6.05600700	-1.77812700	-1.75355600
C	-6.76604700	-3.82259000	-0.48665000

H	-3.52992800	-2.39088800	1.21874000
C	-4.85049200	-0.94201300	0.27998000
C	-5.57090900	-2.98879100	1.54249600
H	-7.00972300	-1.77697100	-2.30866400
H	-5.30313100	-1.32632300	-2.41208300
C	-6.19076600	-0.93450700	-0.47449500
H	-6.51596100	-4.86579800	-0.22786800
H	-7.72226300	-3.84734400	-1.03623600
C	-6.91304800	-2.98388800	0.79373700
H	-4.07170200	-0.48469800	-0.34213600
H	-4.92437700	-0.32760900	1.19307900
H	-5.30005700	-4.01829100	1.83323900
H	-5.65250100	-2.40172300	2.47334400
H	-6.44981000	0.10021200	-0.74936600
C	-7.28586300	-1.53593900	0.42308200
H	-7.69916000	-3.41857900	1.43392700
H	-7.40000100	-0.93245500	1.34034500
H	-8.25868400	-1.51646100	-0.09759900
C	5.94957400	2.13297700	0.52147700
H	5.46278100	2.85165000	-0.16147100
C	6.85338300	1.22743100	-0.36466400
C	6.87347100	2.94123600	1.46582000
H	6.22148900	0.62476900	-1.03157100
C	7.71017000	0.29969200	0.51241600
C	7.77526200	2.11711100	-1.21498800
H	6.25968600	3.58061700	2.12184200
C	7.77171900	2.02266600	2.32313600
C	7.78949000	3.83576800	0.60713100
H	8.30760600	-0.36681400	-0.13281900
H	7.06123400	-0.34560900	1.12751600
C	8.63627300	1.13587700	1.41400700
H	7.17127100	2.77024500	-1.86086100
H	8.39168400	1.48723500	-1.87933400
C	8.67486200	2.96540500	-0.30402000
H	7.17771500	1.38553200	2.99243600
H	8.41566100	2.64606000	2.96699800
H	7.18068200	4.52263100	-0.00467900
H	8.42011000	4.46059700	1.26219800
H	9.25535200	0.46600900	2.03435600
C	9.54406400	2.02936500	0.55218600
H	9.32079800	3.61507200	-0.91835700
H	10.21705100	2.61718100	1.19979000
H	10.18376900	1.40950400	-0.09938000
C	0.08338900	0.93660900	-2.57983000

C	1.14051200	1.81907900	-2.27135900
H	-0.90528000	1.35209200	-2.77287800
H	0.29496000	0.00286300	-3.09864200
H	0.87538800	2.87660900	-2.11820800
C	2.54509700	1.61722300	-2.65068600
C	3.42363500	2.71876800	-2.66545100
C	3.05847300	0.36420000	-3.04778100
C	4.74475600	2.58798900	-3.09212900
H	3.04658000	3.69797900	-2.35840700
C	4.38319900	0.23294600	-3.46038100
H	2.41168400	-0.51364700	-3.02254200
C	5.23447400	1.34307300	-3.49587100
H	5.39337100	3.46716500	-3.11490800
H	4.75989000	-0.74981500	-3.74594300
H	6.27038300	1.23514200	-3.82360900
C	-3.27388600	1.57425900	-1.81284700
C	-4.14760600	2.69803800	-1.86911400
C	-5.12877700	2.83532300	-2.85804200
C	-5.21013900	1.83475700	-3.82514800
C	-4.33298400	0.72888500	-3.80653800
C	-3.37115700	0.58885300	-2.81001200
C	-2.43402700	1.74184600	-0.64465400
C	-2.80858100	2.94089700	-0.07292600
H	-5.79814100	3.69766300	-2.86844400
H	-5.96184800	1.91169200	-4.61443800
H	-4.41623700	-0.03443100	-4.58389400
H	-2.70685700	-0.27350300	-2.79743000
H	-2.43314200	3.41182600	0.83188200
N	-3.83478900	3.52941400	-0.80419200
C	-4.66195900	4.58789700	-0.32708500
C	-5.77232100	4.28682000	0.48825100
C	-4.35241400	5.90628700	-0.67615900
C	-6.54621000	5.36335300	0.94690800
C	-5.13928900	6.95928000	-0.20914600
H	-3.48649400	6.08654100	-1.31585100
C	-6.23847300	6.68226300	0.60744600
H	-7.40919000	5.16579000	1.58652400
H	-4.89561100	7.98879400	-0.48077400
H	-6.86197300	7.49775900	0.98203100
C	-1.45474000	0.79755000	-0.09222700
H	-1.82878100	-0.23422900	-0.05626700
N	-0.61756200	1.23851700	0.93024400
C	-0.71362200	0.83217800	2.24608700
C	0.23647000	1.30213500	3.18351400

C	-1.75834000	0.02271800	2.73296700
C	0.16206800	0.95176800	4.52096000
H	1.03278800	1.95742800	2.82699100
C	-1.82427300	-0.35283200	4.08149700
H	-2.53938100	-0.31014400	2.04931500
C	-0.85698300	0.10050900	4.98471600
H	0.88897400	1.32922500	5.24371100
H	-2.65003000	-0.98425700	4.41093000
O	-0.81275300	-0.22439500	6.31234200
C	-1.81057500	-1.06818500	6.82588500
H	-1.59296700	-1.20682700	7.89382800
H	-2.81980900	-0.62828900	6.72136600
H	-1.81137200	-2.05785600	6.33075300
C	-6.09602300	2.85378400	0.88501900
C	-7.58011800	2.51557800	0.69658200
C	-5.62972000	2.56482100	2.32096700
H	-5.52644800	2.18973400	0.22038900
H	-7.90352100	2.71167900	-0.33723700
H	-7.75781700	1.45096200	0.91190300
H	-8.22591700	3.09899400	1.37239600
H	-4.54681700	2.72553300	2.42830600
H	-6.14670200	3.21476500	3.04601600
H	-5.84189400	1.51781600	2.59100900

int2(triplet)

C	2.19697700	-2.99003200	-0.03801200
C	0.82390400	-3.28015000	-0.67952500
H	2.36158900	-3.65346400	0.82081100
H	0.93764400	-3.58936200	-1.72786800
N	0.18763400	-1.93389100	-0.65349000
N	2.01426400	-1.60468400	0.46823800
C	0.05326500	-4.35466900	0.05503200
C	-0.26733800	-5.55513200	-0.58822800
C	-0.35307600	-4.15386900	1.38125100
C	-0.99725100	-6.54382400	0.07880200
H	0.03974800	-5.70920100	-1.62621800
C	-1.08161900	-5.13929500	2.04896100
H	-0.12526400	-3.21271100	1.88243100
C	-1.40746600	-6.33537600	1.39794000
H	-1.24991900	-7.47422100	-0.43549500
H	-1.40770200	-4.96159000	3.07495900
H	-1.98441300	-7.10294500	1.91966600
C	3.39826200	-3.05741900	-0.95601600
C	4.63259400	-3.47428100	-0.44082000

C	3.33191400	-2.63087100	-2.29045500
C	5.77807900	-3.47457700	-1.24022400
H	4.69563200	-3.79133400	0.60305000
C	4.47366900	-2.63893200	-3.09422400
H	2.38582000	-2.27664100	-2.70585200
C	5.70012500	-3.05953300	-2.57150800
H	6.73421200	-3.79546900	-0.82097000
H	4.40635600	-2.31349000	-4.13480000
H	6.59374900	-3.05783500	-3.19952100
C	-1.01207300	-1.69817400	-1.37586600
C	-1.13279500	-0.61091500	-2.27551700
C	-2.08224800	-2.58467800	-1.25528200
C	-2.26572500	-0.53275700	-3.08802700
C	-3.23999800	-2.50318500	-2.04371500
H	-2.00582300	-3.37837500	-0.52016600
C	-3.29573000	-1.47522200	-2.98658900
H	-2.35963200	0.27525700	-3.81009500
H	-4.14818200	-1.37742500	-3.65793100
C	0.89248700	-0.99874700	0.03494500
Fe	0.15556200	0.91788200	0.14082800
C	3.00335300	-1.10335400	1.35592400
C	4.02783000	-0.27795800	0.89952400
C	3.03048500	-1.58760300	2.67887300
C	5.07973400	0.13730300	1.72353700
H	3.99058500	0.02531400	-0.13867900
C	4.06056800	-1.16537200	3.52963300
C	5.05808200	-0.31158500	3.05160900
H	4.09321800	-1.49874900	4.56653200
H	5.84341400	0.00436100	3.74230300
O	-0.14129500	0.32292900	-2.27625600
O	2.05747700	-2.46808500	3.02001400
C	-0.25830700	1.42566400	-3.17106300
H	-0.28422100	1.07601700	-4.21564200
H	-1.16012800	2.01485800	-2.94659600
H	0.63222500	2.03978700	-3.01748000
C	2.10418000	-3.09423800	4.28295700
H	3.05950400	-3.62680300	4.43433400
H	1.96443900	-2.36819500	5.10237800
H	1.27922900	-3.81718900	4.29897700
C	-4.30249800	-3.57647800	-1.85979400
H	-3.80159400	-4.52980700	-2.11065900
C	-5.54754900	-3.47378600	-2.77043500
C	-4.78570500	-3.71165300	-0.38925800
H	-5.22626300	-3.35928100	-3.81882000

C	-6.45179800	-2.29156800	-2.36750500
C	-6.35688500	-4.77856100	-2.63280000
H	-3.91501100	-3.75315700	0.28218500
C	-5.66659300	-2.51120500	-0.00775000
C	-5.59519300	-5.01138100	-0.24998600
H	-7.32541900	-2.25387000	-3.04044100
H	-5.92540300	-1.33184300	-2.47489500
C	-6.91020100	-2.46168700	-0.91064800
H	-5.74054000	-5.63917800	-2.94346300
H	-7.23043000	-4.74884800	-3.30586900
C	-6.82232600	-4.96040200	-1.17623900
H	-5.09183500	-1.57719400	-0.10614500
H	-5.97039600	-2.59290800	1.04939000
H	-4.95941400	-5.87780000	-0.49947700
H	-5.91750600	-5.14062200	0.79782200
H	-7.54357800	-1.60671300	-0.62145300
C	-7.70850800	-3.76938800	-0.77069800
H	-7.39580600	-5.89835400	-1.08715500
H	-8.05493200	-3.89354300	0.26973000
H	-8.60916400	-3.73213700	-1.40713100
C	6.18984800	1.05613300	1.23415400
H	6.09565800	1.98657700	1.82442900
C	6.11436000	1.46151200	-0.25892200
C	7.61076000	0.49278000	1.50838000
H	5.11269200	1.85266400	-0.48826600
C	6.43155900	0.27224600	-1.18721100
C	7.15183800	2.56935900	-0.51946300
H	7.68415000	0.17495200	2.56056900
C	7.89029500	-0.71018700	0.59046800
C	8.64854400	1.59731200	1.24405900
H	6.36546900	0.60688700	-2.23504400
H	5.69847600	-0.53684100	-1.07646800
C	7.83656100	-0.26686600	-0.88217900
H	6.93388400	3.44803900	0.11104000
H	7.07850300	2.90141500	-1.56841800
C	8.56906600	2.04591800	-0.22472000
H	7.14693100	-1.50407100	0.77094600
H	8.88189100	-1.13342000	0.82600400
H	8.46493500	2.45410800	1.91454300
H	9.66094800	1.22153000	1.47117100
H	8.04744600	-1.13055900	-1.53501900
C	8.87425700	0.84095900	-1.13263500
H	9.30593100	2.84583700	-0.40852700
H	9.89251600	0.46522400	-0.93145900

H	8.84703400	1.14828700	-2.19198200
C	0.86368500	2.83941500	0.22722300
C	1.93453800	2.00127800	-0.15458400
H	0.75287900	3.15912800	1.26857800
H	0.37568100	3.48654800	-0.50859700
H	2.59006600	1.63227800	0.64162500
C	2.56630300	2.06222600	-1.49722300
C	2.78696500	3.30655900	-2.12039800
C	2.98038600	0.90835500	-2.18972400
C	3.39653200	3.39430500	-3.37434700
H	2.47973200	4.21504500	-1.59832500
C	3.60036900	0.99272900	-3.43527300
H	2.77624100	-0.06916700	-1.75884000
C	3.81210800	2.23666400	-4.03863200
H	3.55564400	4.37454100	-3.83153800
H	3.91325800	0.07728100	-3.94152900
H	4.29325700	2.30262000	-5.01739700
C	-3.08858600	3.21595600	-1.00936200
C	-3.16675600	4.62323900	-0.79547500
C	-3.75394500	5.48755700	-1.72664200
C	-4.30187100	4.92356700	-2.87764500
C	-4.27015100	3.52941700	-3.09227600
C	-3.66915000	2.67497000	-2.16969000
C	-2.38176200	2.64487000	0.12076800
C	-2.10560400	3.70272200	0.96962600
H	-3.77897300	6.56481200	-1.55411500
H	-4.76898700	5.57255800	-3.62230600
H	-4.72639900	3.11634200	-3.99536000
H	-3.65460800	1.59757700	-2.33792700
H	-1.60108200	3.69401600	1.93101100
N	-2.57347800	4.89554700	0.42789700
C	-2.33511800	6.18561800	0.97511300
C	-3.40131300	6.98918200	1.42640400
C	-1.00769700	6.62873100	1.06528100
C	-3.08217800	8.25521100	1.94267300
C	-0.71776100	7.88308800	1.59776100
H	-0.21331500	5.97156600	0.70535100
C	-1.76387100	8.70319400	2.03016600
H	-3.88757200	8.89982100	2.30200000
H	0.31827100	8.22267900	1.66528800
H	-1.55230100	9.69273900	2.44239100
C	-2.01417700	1.25222400	0.29754700
H	-2.60647400	0.52866200	-0.28531200
N	-1.34218000	0.82911800	1.41062400

C	-1.71332800	-0.30578900	2.11261100
C	-0.84403200	-0.81896400	3.10587300
C	-2.96195900	-0.94088700	1.96101000
C	-1.21070800	-1.89875300	3.89512700
H	0.12470400	-0.33692900	3.24608000
C	-3.32550300	-2.04341300	2.74113800
H	-3.67657300	-0.54879900	1.23841400
C	-2.45205000	-2.53072500	3.71629200
H	-0.54811400	-2.27857200	4.67333400
H	-4.30184300	-2.49860700	2.58269200
O	-2.70847500	-3.61284600	4.52041000
C	-3.94805400	-4.26143400	4.37799800
H	-3.96389800	-5.08169600	5.10871800
H	-4.79388000	-3.58071300	4.58523600
H	-4.07857400	-4.68166000	3.36296100
C	-4.83569900	6.48309300	1.44570100
C	-5.82245300	7.47131700	0.81134200
C	-5.24760700	6.12026100	2.88216500
H	-4.87180100	5.55836700	0.85480500
H	-5.53089200	7.72229900	-0.21981000
H	-6.83330500	7.03507200	0.78021200
H	-5.88731500	8.41118700	1.38312800
H	-4.56303800	5.37287700	3.31137100
H	-5.23299100	7.00706100	3.53692100
H	-6.26663400	5.70118900	2.90022700

TS3(triplet)

C	-1.76933200	-2.09360300	1.10398400
C	-0.34261600	-2.31795700	1.68723100
H	-2.10344800	-2.99535500	0.56993500
H	-0.36092000	-2.13105600	2.76997600
N	0.41620600	-1.24737900	1.03625400
N	-1.55423200	-0.99885200	0.12587400
C	0.23440700	-3.69120200	1.43727900
C	0.42866900	-4.57662700	2.50301100
C	0.55889100	-4.10017200	0.13672600
C	0.93379200	-5.86039800	2.27547200
H	0.18544300	-4.25568700	3.51965200
C	1.05487500	-5.38349200	-0.09287900
H	0.42492700	-3.41252000	-0.69805100
C	1.24494900	-6.26649900	0.97564600
H	1.08601100	-6.54262300	3.11538900
H	1.28338100	-5.69105900	-1.11399500
H	1.63722200	-7.27020100	0.79438500

C	-2.74986700	-1.76614000	2.21083100
C	-3.62755900	-2.74468500	2.69082900
C	-2.73251300	-0.50553300	2.82002400
C	-4.48752500	-2.46486700	3.75695900
H	-3.64070300	-3.73315500	2.22324800
C	-3.59611600	-0.22158300	3.87844400
H	-2.03271100	0.24455400	2.45686100
C	-4.47746900	-1.20051700	4.35044300
H	-5.17244700	-3.23535400	4.11864900
H	-3.58190400	0.76948900	4.33938900
H	-5.15554300	-0.97697800	5.17727700
C	1.69836800	-0.86741800	1.51694600
C	1.77279900	-0.09728200	2.69450800
C	2.84810400	-1.17035700	0.80297400
C	3.01712500	0.38304600	3.10344700
C	4.10643500	-0.67658000	1.18207700
H	2.72640000	-1.75748900	-0.10368300
C	4.15923000	0.11077900	2.33694400
H	3.11136300	0.99538200	4.00003500
H	5.10446400	0.54469000	2.66098800
C	-0.28009300	-0.49962000	0.15504600
Fe	0.44250400	1.01474700	-0.75247700
C	-2.58296400	-0.57287400	-0.73737800
C	-3.86134000	-1.13441500	-0.57308200
C	-2.42893900	0.37587000	-1.78446200
C	-4.97648100	-0.80430300	-1.34896200
H	-3.99367200	-1.85801800	0.21933400
C	-3.53004100	0.69811600	-2.57791600
C	-4.78436000	0.13232200	-2.36827800
H	-3.41628200	1.44132200	-3.36318800
H	-5.60719200	0.44635800	-3.00917300
O	0.58822500	0.16050300	3.31754800
O	-1.21308200	0.98294400	-2.02782500
C	0.59485600	0.87531000	4.53351100
H	1.21370700	0.37115800	5.29624500
H	0.95986400	1.90659300	4.39450600
H	-0.44716300	0.90691600	4.87851600
C	-0.94096800	1.48464400	-3.34395100
H	-1.51584000	2.40153000	-3.53319400
H	0.13202200	1.69663900	-3.35219900
H	-1.18637200	0.71845100	-4.09454800
C	-0.99434200	2.29517400	0.96927600
C	-2.31922200	2.51857900	0.94976800
H	-0.49135900	1.91605600	1.85624900

H	-0.35663500	2.64990400	0.13280500
H	-2.90194700	2.25912800	1.83955400
C	-3.10659600	3.09110900	-0.14670600
C	-4.51083800	3.05209200	-0.08563300
C	-2.50606700	3.70008000	-1.26380100
C	-5.29054800	3.59204400	-1.10933300
H	-4.99220000	2.57919900	0.77424500
C	-3.28289400	4.24413500	-2.28323000
H	-1.41984400	3.74513600	-1.32994500
C	-4.68024400	4.18962200	-2.21447900
H	-6.37962300	3.54593900	-1.04404800
H	-2.79536100	4.72613600	-3.13442100
H	-5.28803700	4.61887000	-3.01440300
C	4.28575500	2.40211600	-1.22666800
C	3.89790900	3.39490200	-0.28178500
C	4.81847300	4.27695000	0.27933200
C	6.16347400	4.16176100	-0.10818900
C	6.56410100	3.19476700	-1.03956800
C	5.62971800	2.31555000	-1.60503800
C	3.08974000	1.64980500	-1.53024700
C	1.96603000	2.19220400	-0.76736500
H	4.50133800	5.03046700	1.00267600
H	6.90464100	4.83895200	0.32341200
H	7.61516400	3.12551800	-1.33028300
H	5.94897300	1.56979800	-2.33717400
N	2.51437600	3.30655600	-0.08570900
C	1.79312800	4.11856600	0.82653500
C	0.97077000	5.17255400	0.36845200
C	1.90893100	3.84125500	2.19361600
C	0.25668800	5.89979800	1.33216900
C	1.20241200	4.59144400	3.13416300
H	2.55708400	3.01680200	2.49415400
C	0.36511300	5.61831000	2.69655500
H	-0.39924400	6.71111600	1.01553200
H	1.30173200	4.37460300	4.20089600
H	-0.20437000	6.20986500	3.41758100
C	2.83769300	0.45745300	-2.17179600
H	3.60238400	-0.06513100	-2.75446700
N	1.60486000	-0.07377500	-2.02480900
C	1.37961200	-1.37483300	-2.48980600
C	2.41740200	-2.31411800	-2.67985300
C	0.07138100	-1.82902900	-2.77624000
C	2.17621200	-3.61128200	-3.14210800
H	3.44611700	-2.04353500	-2.44661300

C	-0.17892600	-3.11823000	-3.22860700
H	-0.76425500	-1.15477300	-2.63623200
C	0.87074100	-4.03030100	-3.41860700
H	3.02224400	-4.28935100	-3.25523000
H	-1.19907000	-3.44595800	-3.43822600
O	0.52903200	-5.28466600	-3.83634100
C	1.55849200	-6.19443500	-4.13419900
H	1.07629100	-7.11195500	-4.49787400
H	2.23570700	-5.80770300	-4.91771400
H	2.16455100	-6.44267600	-3.24256900
H	1.66127000	1.26356200	0.21341700
C	0.89876800	5.50418800	-1.11631600
C	2.21295800	6.13682000	-1.61047600
C	-0.27534400	6.41468700	-1.48981600
H	0.78118800	4.54121900	-1.64353800
H	3.07905000	5.48752400	-1.43666300
H	2.15389600	6.34059800	-2.69180400
H	2.39401000	7.09435000	-1.09483100
H	-1.23684700	6.04582700	-1.10748800
H	-0.12744000	7.43611500	-1.10287600
H	-0.35416900	6.49388800	-2.58549200
C	5.30042500	-0.93761500	0.27798700
H	5.10200500	-0.34920900	-0.63422100
C	5.42652400	-2.42377700	-0.15034400
C	6.66997800	-0.47157900	0.82205000
H	4.45828100	-2.78586500	-0.52538300
C	5.85874800	-3.28659500	1.04655100
C	6.47040400	-2.53462900	-1.27375500
H	6.60113700	0.58497600	1.12450000
C	7.11984700	-1.34739000	2.00807400
C	7.71553000	-0.58834300	-0.30233600
H	5.90989300	-4.34609500	0.74252300
H	5.10666200	-3.21905700	1.84966100
C	7.23108500	-2.81444000	1.55810600
H	6.15474800	-1.93040100	-2.14196800
H	6.54179200	-3.58097100	-1.61765500
C	7.83746900	-2.05110000	-0.76164400
H	6.41014600	-1.27270000	2.84595900
H	8.09497800	-0.99009900	2.38129000
H	7.42584700	0.05584200	-1.14699600
H	8.69151600	-0.22260600	0.05942500
H	7.54375300	-3.43941400	2.41163700
C	8.26935900	-2.92766300	0.42759400
H	8.58507800	-2.12564300	-1.56933000

H	9.26073300	-2.60792000	0.79195500
H	8.36806300	-3.97928000	0.10708400
C	-6.32468300	-1.47758100	-1.09841900
H	-6.34784700	-2.38225900	-1.73541900
C	-7.54241600	-0.60513700	-1.49806400
C	-6.53527700	-1.94218200	0.36672300
H	-7.43339100	-0.26936200	-2.54040800
C	-7.63963300	0.61566900	-0.56618300
C	-8.83365700	-1.43472000	-1.38989000
H	-5.69863300	-2.58092000	0.68439100
C	-6.63403500	-0.72347000	1.30100700
C	-7.82979900	-2.76714500	0.46748600
H	-8.48456500	1.25463500	-0.87565200
H	-6.72689000	1.22268000	-0.64775500
C	-7.83360500	0.14603000	0.88602800
H	-8.78325700	-2.30397600	-2.06750000
H	-9.69318200	-0.82375200	-1.71407700
C	-9.03453500	-1.90575300	0.05897100
H	-5.70250000	-0.13738600	1.26651600
H	-6.75056700	-1.06445300	2.34205500
H	-7.76308200	-3.65764100	-0.18061600
H	-7.95429000	-3.13048500	1.50180700
H	-7.90166300	1.02323900	1.55158500
C	-9.12729200	-0.68215600	0.98854000
H	-9.96071400	-2.49997800	0.13259400
H	-9.28483300	-1.01070800	2.03016100
H	-9.99741100	-0.06255900	0.71147100

TS3(triplet)

C	-1.87632000	-2.13013800	1.04492500
C	-0.48811400	-2.43076900	1.68570300
H	-2.20490700	-2.98735800	0.43898600
H	-0.56731200	-2.32747400	2.77655300
N	0.32394800	-1.33467800	1.15992700
N	-1.58143700	-0.98549900	0.14353300
C	0.06668600	-3.80137200	1.36977000
C	-0.08212500	-4.83351400	2.30498800
C	0.70030900	-4.06804300	0.14915200
C	0.38907900	-6.11878200	2.02582100
H	-0.56741400	-4.62456800	3.26259500
C	1.17803600	-5.35056800	-0.12670600
H	0.82519800	-3.27279000	-0.58530800
C	1.02316600	-6.37861400	0.80787500
H	0.26948200	-6.91546500	2.76417200

H	1.67328300	-5.53841700	-1.08017200
H	1.40054600	-7.38056700	0.58922600
C	-2.90159900	-1.84752000	2.12371200
C	-3.85146600	-2.81565700	2.46816900
C	-2.85714700	-0.64839300	2.84535800
C	-4.76175000	-2.58096500	3.50325600
H	-3.88404200	-3.75870000	1.91540700
C	-3.77056600	-0.40757100	3.87189100
H	-2.09387100	0.08689300	2.59823200
C	-4.72876500	-1.37247200	4.20250700
H	-5.50414700	-3.34149800	3.75625800
H	-3.73569300	0.53922200	4.41673500
H	-5.44640500	-1.18214600	5.00384500
C	1.58253800	-1.00188400	1.72757800
C	1.60851500	-0.38558600	2.99347000
C	2.75552700	-1.16857700	1.00310600
C	2.83588700	0.03922300	3.50603000
C	3.99444700	-0.71342600	1.48035100
H	2.66502200	-1.61198500	0.01519900
C	4.00351500	-0.11973800	2.74858900
H	2.89539400	0.52336700	4.48055700
H	4.93749000	0.25267200	3.16966800
C	-0.29409200	-0.53763400	0.26200300
Fe	0.59981800	0.88176000	-0.69112700
C	-2.56020400	-0.46621400	-0.72860100
C	-3.86657500	-0.97849400	-0.63608000
C	-2.33997900	0.54196200	-1.70774600
C	-4.94896200	-0.53757200	-1.40438200
H	-4.05264200	-1.75120000	0.09671400
C	-3.40728500	0.97342200	-2.49398600
C	-4.69209700	0.45854800	-2.34891500
H	-3.24290400	1.76871800	-3.21690600
H	-5.48485100	0.86207900	-2.97696300
O	0.40333200	-0.21283400	3.60442800
O	-1.08920600	1.09863100	-1.90302100
C	0.35470800	0.45298400	4.84635500
H	0.93831500	-0.07922600	5.61781800
H	0.72696900	1.48873500	4.76177700
H	-0.70170400	0.47191400	5.14516700
C	-0.78303100	1.65547100	-3.19103200
H	-1.30183600	2.61447000	-3.32648900
H	0.30112700	1.80314400	-3.19022100
H	-1.07072900	0.94684300	-3.98186500
C	-1.09818000	2.38282100	1.44965400

C	-2.39879600	2.41548200	1.11812500
H	-0.73028000	1.75498100	2.26208600
H	-0.34772600	2.99459700	0.94727600
H	-3.10595000	1.80425200	1.68622200
C	-3.00649900	3.18736000	0.02823200
C	-4.40686900	3.22626900	-0.09185500
C	-2.23900900	3.87109700	-0.93286400
C	-5.02356900	3.92556400	-1.12989700
H	-5.01569100	2.68630300	0.63753100
C	-2.85291200	4.56648400	-1.97231400
H	-1.15125300	3.83128900	-0.87661500
C	-4.24897300	4.59746000	-2.07845600
H	-6.11343400	3.93858000	-1.20300600
H	-2.23799600	5.09254600	-2.70657600
H	-4.72698900	5.14312000	-2.89538700
C	4.40210400	2.22850400	-1.49188900
C	4.08908100	3.28748300	-0.59320600
C	5.05452500	4.19119900	-0.15060300
C	6.36212300	4.04591400	-0.63447800
C	6.68584400	3.02468000	-1.54071300
C	5.71360200	2.11423000	-1.97208400
C	3.18881100	1.45623600	-1.64377900
C	2.13686700	2.06116000	-0.84399600
H	4.79808700	4.98168900	0.55702000
H	7.13759600	4.73913500	-0.29986000
H	7.71023500	2.93617300	-1.91059400
H	5.97877300	1.31704500	-2.67071500
N	2.72633100	3.21574100	-0.28286600
C	2.12597100	3.95324600	0.77552600
C	1.35664800	5.10953100	0.52388000
C	2.30037200	3.47045600	2.07847400
C	0.77178400	5.74578600	1.63071900
C	1.71124900	4.12300400	3.16045500
H	2.89224400	2.56530300	2.22236800
C	0.94087300	5.26425800	2.93090900
H	0.16220700	6.63644700	1.47764900
H	1.85294300	3.74188600	4.17489400
H	0.46537300	5.78522200	3.76546200
C	2.91257200	0.25130600	-2.28573500
H	3.68762800	-0.26458800	-2.86776100
N	1.68949000	-0.26769800	-2.14689300
C	1.39459400	-1.54719400	-2.61945000
C	2.35873500	-2.53639900	-2.90308300
C	0.04000300	-1.91488200	-2.79897100

C	1.99791600	-3.81750000	-3.33657300
H	3.41860800	-2.32048500	-2.76880600
C	-0.32679500	-3.18419600	-3.22064500
H	-0.73107500	-1.18019700	-2.58835300
C	0.64900800	-4.15747400	-3.49187400
H	2.78647000	-4.54488900	-3.53062800
H	-1.37800800	-3.45196100	-3.34371300
O	0.19566000	-5.38500600	-3.87451400
C	1.13509700	-6.38093600	-4.18916400
H	0.56580500	-7.27170500	-4.48758500
H	1.79357400	-6.07942200	-5.02471300
H	1.77082300	-6.64104300	-3.32216000
H	1.73437600	1.28233600	0.33551700
C	1.17661300	5.63390000	-0.89565400
C	2.46331300	6.29055800	-1.42696600
C	0.00823600	6.61427600	-1.04278300
H	0.97647900	4.75107900	-1.52745900
H	3.30945100	5.59418900	-1.44444500
H	2.30629800	6.65771000	-2.45399600
H	2.74125700	7.15284800	-0.79863200
H	-0.92800300	6.22299600	-0.62252700
H	0.22812000	7.57397100	-0.54740700
H	-0.16369500	6.83516200	-2.10778100
C	5.22629200	-0.77399500	0.58753700
H	5.13975400	0.09525800	-0.08640500
C	5.28180100	-2.03963200	-0.30416000
C	6.57919300	-0.64608900	1.32648800
H	4.33112900	-2.15482800	-0.84090600
C	5.53766300	-3.28698700	0.55769300
C	6.40967600	-1.88460500	-1.33695300
H	6.57205900	0.25444000	1.95990900
C	6.84321600	-1.89640100	2.18541500
C	7.70595600	-0.49268100	0.28893300
H	5.54327100	-4.18685100	-0.08108700
H	4.72231400	-3.41819500	1.28744600
C	6.88728700	-3.14286400	1.28324500
H	6.21887900	-1.00297300	-1.97049900
H	6.42918400	-2.76504800	-2.00256600
C	7.75860300	-1.73540700	-0.61504500
H	6.05770100	-2.01542000	2.94868200
H	7.80042700	-1.78391800	2.72301200
H	7.53567400	0.41367600	-0.31357400
H	8.67126100	-0.36232600	0.80755000
H	7.07420200	-4.03823100	1.89987300

C	8.01431200	-2.98582400	0.24580100
H	8.56756000	-1.62192000	-1.35633700
H	8.98876200	-2.89962000	0.75671000
H	8.06553100	-3.88302100	-0.39513200
C	-6.33212500	-1.15479700	-1.20806600
H	-6.35730400	-2.07601900	-1.82072700
C	-7.49984600	-0.25455100	-1.68535000
C	-6.62262800	-1.56997200	0.25877300
H	-7.33334900	0.04593300	-2.73120700
C	-7.60363300	0.99583900	-0.79299200
C	-8.82138500	-1.03988900	-1.61139000
H	-5.82248500	-2.22308300	0.63249800
C	-6.71735300	-0.32021700	1.15067100
C	-7.94655800	-2.34924300	0.32615100
H	-8.41932900	1.64439400	-1.15600800
H	-6.67544000	1.58142900	-0.85133300
C	-7.87072100	0.57408300	0.66245200
H	-8.76988700	-1.93019000	-2.26099500
H	-9.64524700	-0.41212400	-1.99116400
C	-9.10206600	-1.46161500	-0.16011800
H	-5.76489200	0.23259300	1.13184200
H	-6.88328700	-0.62408400	2.19684100
H	-7.88138600	-3.26060500	-0.29252400
H	-8.12762500	-2.67694000	1.36409300
H	-7.93830500	1.47193300	1.29988800
C	-9.19360200	-0.21027900	0.73190200
H	-10.05045500	-2.02253200	-0.11248000
H	-9.40527600	-0.50494400	1.77407100
H	-10.03046900	0.42883200	0.40190700

int4(singlet)

C	1.13664800	-2.51729400	-1.22496700
C	-0.33676400	-2.59200200	-1.67576200
H	1.44600400	-3.45345600	-0.74168700
H	-0.40328800	-2.60742900	-2.77203000
N	-0.85798600	-1.29345700	-1.18837100
N	1.07283100	-1.44512800	-0.19601100
C	-1.12187300	-3.75957900	-1.11692500
C	-2.08400900	-4.39471500	-1.91117100
C	-0.95127900	-4.17998600	0.21070400
C	-2.86989100	-5.42685800	-1.39172000
H	-2.22857400	-4.06427200	-2.94271300
C	-1.72719100	-5.21843700	0.72799400
H	-0.22131500	-3.68321800	0.85366900

C	-2.69268200	-5.84125100	-0.06951800
H	-3.62125900	-5.90846000	-2.02197400
H	-1.58122800	-5.54056600	1.76165000
H	-3.30402400	-6.64846000	0.34034900
C	2.08698800	-2.22519900	-2.36864300
C	3.10155600	-3.13399400	-2.69074600
C	1.95944200	-1.04824000	-3.12047300
C	3.99836700	-2.86304100	-3.72801400
H	3.20746900	-4.05174600	-2.10731700
C	2.85504100	-0.77701300	-4.15560500
H	1.15013800	-0.35271600	-2.90019300
C	3.88214100	-1.67812800	-4.45825300
H	4.79349700	-3.57576100	-3.95859800
H	2.74941000	0.14488200	-4.73313600
H	4.58575300	-1.45928400	-5.26495200
C	-2.08453900	-0.79966100	-1.71254900
C	-2.08156700	0.17773700	-2.73279800
C	-3.29422300	-1.33892500	-1.28693500
C	-3.30376000	0.61254200	-3.24867300
C	-4.53166100	-0.93259300	-1.81257800
H	-3.26773600	-2.10444600	-0.51564500
C	-4.50749400	0.05391300	-2.80175800
H	-3.32875600	1.37919300	-4.02280900
H	-5.43241000	0.40012500	-3.26120900
C	-0.06239300	-0.68206100	-0.27379900
Fe	-0.37660600	1.10642100	0.42685700
C	2.10804900	-1.32904200	0.76204700
C	3.44396100	-1.44541000	0.35118100
C	1.84867700	-1.18070800	2.13945900
C	4.52218000	-1.44106800	1.24054200
H	3.63471900	-1.49642600	-0.71363900
C	2.91495700	-1.19576900	3.04331000
C	4.22978600	-1.33947200	2.60927000
H	2.68425800	-1.10700600	4.10723200
H	5.03050600	-1.33931300	3.34842200
O	-0.87762200	0.62786100	-3.17607600
O	0.57010900	-1.03447300	2.60383300
C	-0.81234500	1.92246400	-3.74114400
H	-1.27247000	1.95882600	-4.74383600
H	-1.29793000	2.66268700	-3.08336400
H	0.25431000	2.16949600	-3.83405400
C	0.23356800	0.29741400	2.92794500
H	0.83243400	0.67451000	3.77695100
H	0.47165900	1.01708300	2.09919200

H	-0.82882900	0.33397000	3.17951700
C	-5.77735300	-1.63647900	-1.29882200
H	-5.57821100	-2.71304800	-1.45184800
C	-7.11205600	-1.33097000	-2.01711200
C	-5.99281400	-1.45021400	0.22828100
H	-6.97278800	-1.42961900	-3.10675300
C	-7.64559400	0.07756900	-1.68412800
C	-8.15356600	-2.36450800	-1.54060100
H	-5.04511300	-1.61962800	0.75456900
C	-6.49411700	-0.02920300	0.52786300
C	-7.03113200	-2.47598500	0.70847300
H	-8.60242300	0.23690200	-2.21047800
H	-6.95848000	0.86187200	-2.03222300
C	-7.84361200	0.21347900	-0.16696600
H	-7.81596500	-3.38394000	-1.79321800
H	-9.10952600	-2.20246100	-2.06717100
C	-8.36577200	-2.24087300	-0.01908500
H	-5.75125700	0.70738600	0.18205000
H	-6.59192800	0.10182900	1.61656700
H	-6.66292600	-3.49875400	0.52054800
H	-7.16870900	-2.38287100	1.79926000
H	-8.20584100	1.22858000	0.06777200
C	-8.87173100	-0.82581800	0.31240200
H	-9.10809600	-2.98779100	0.30964200
H	-9.03131300	-0.72829100	1.40013000
H	-9.84604100	-0.64581900	-0.17371000
C	5.95321900	-1.40156600	0.71916100
H	6.18833000	-0.32995200	0.61270000
C	6.14824300	-2.04740000	-0.67464000
C	7.00700700	-2.00120000	1.68148200
H	5.42238200	-1.63295500	-1.38822500
C	5.97248000	-3.57237000	-0.57927300
C	7.56038900	-1.72528500	-1.19154800
H	6.90090200	-1.54843900	2.67879200
C	6.83086200	-3.52648900	1.77712200
C	8.41650500	-1.67760800	1.15537100
H	6.08273000	-4.02381000	-1.57976500
H	4.95804700	-3.81698200	-0.22627000
C	7.02108000	-4.15468200	0.38468900
H	7.68471900	-0.63288900	-1.27963800
H	7.68950800	-2.14869000	-2.20235900
C	8.61365100	-2.30409300	-0.23414500
H	5.83028700	-3.77075900	2.16925900
H	7.56624900	-3.94541000	2.48506000

H	8.55296300	-0.58436100	1.10195400
H	9.17362900	-2.06538800	1.85814300
H	6.89454500	-5.24825000	0.45248400
C	8.43279700	-3.82975800	-0.13704800
H	9.62557300	-2.07263900	-0.60714500
H	9.19334900	-4.25867300	0.53776500
H	8.58503200	-4.29187600	-1.12762900
C	1.89315200	1.60169900	-0.78695100
C	3.19964600	1.65644500	-1.07419800
H	1.14133800	1.52383100	-1.56490700
H	1.54290400	1.70618700	0.25870700
H	3.48174200	1.61193600	-2.13123500
C	4.32013600	1.82310200	-0.14393500
C	5.60043800	2.10711800	-0.65328100
C	4.16654400	1.73359400	1.24949400
C	6.68439400	2.32371900	0.19891700
H	5.73697300	2.17200400	-1.73576600
C	5.24693100	1.94082700	2.10245700
H	3.19375900	1.47448600	1.66699900
C	6.51130400	2.24445100	1.58416600
H	7.66880800	2.54829400	-0.21863200
H	5.10482400	1.85327200	3.18188100
H	7.35770800	2.40592900	2.25560900
C	-2.71658100	4.40129300	-0.16238000
C	-1.49608200	5.05465500	-0.49549400
C	-1.45910400	6.35742700	-0.98739700
C	-2.68023500	7.01653400	-1.17973300
C	-3.89709300	6.38306800	-0.87849900
C	-3.92497200	5.08160200	-0.36802900
C	-2.33271600	3.10467000	0.34552900
C	-0.91176300	2.97014800	0.30166000
H	-0.51064800	6.84634300	-1.21584700
H	-2.68308800	8.03703100	-1.57012300
H	-4.83581000	6.91848200	-1.04165100
H	-4.87776600	4.60212700	-0.12946800
N	-0.43882000	4.16826300	-0.19606100
C	0.93257600	4.52102600	-0.32530200
C	1.76146000	4.60611500	0.81751500
C	1.43722600	4.78285700	-1.60236000
C	3.11236400	4.91270600	0.60840200
C	2.78228700	5.10496300	-1.78002500
H	0.76054500	4.70446800	-2.45531000
C	3.62357200	5.15560600	-0.66875500
H	3.79083700	4.95658100	1.46013800

H	3.17308400	5.29666100	-2.78209800
H	4.68582900	5.37834100	-0.78997500
C	-3.01176200	1.97562700	0.81063300
H	-4.09896200	1.96575700	0.95163400
N	-2.29283400	0.89454600	1.09834400
C	-2.89264300	-0.10000900	1.89038400
C	-3.92421500	0.20053600	2.80274000
C	-2.46190500	-1.44208900	1.84008800
C	-4.56918400	-0.79125100	3.54659500
H	-4.22918700	1.23725600	2.94803700
C	-3.09087500	-2.43239500	2.58025200
H	-1.62724900	-1.70223600	1.20039900
C	-4.17195500	-2.12765900	3.42166200
H	-5.37152500	-0.50162300	4.22495000
H	-2.77013000	-3.47089900	2.50467000
O	-4.74979500	-3.17360800	4.07428600
C	-5.85890800	-2.91972900	4.90028400
H	-6.18581000	-3.88955500	5.29909600
H	-5.60203400	-2.25489200	5.74546000
H	-6.69451400	-2.46423500	4.33735600
H	-0.81728400	1.36433000	-0.94103200
C	1.17543200	4.41567200	2.21074600
C	0.28020200	5.60974200	2.58778700
C	2.21790400	4.16899300	3.30325800
H	0.52854400	3.52557500	2.15890800
H	-0.52300300	5.76871200	1.85612200
H	-0.18755700	5.44294300	3.57142400
H	0.87658000	6.53521700	2.64584700
H	2.88121400	3.32791000	3.05890200
H	2.85051800	5.05495500	3.47615500
H	1.71278900	3.94115500	4.25499300

int4(triplet)

C	1.98339300	-2.58687900	-1.89967600
C	0.60717400	-2.59122500	-2.58245300
H	2.28452400	-3.59623300	-1.59062900
H	0.70713600	-2.39836000	-3.65550900
N	-0.03316100	-1.41005700	-1.94147300
N	1.70508600	-1.76764900	-0.69076700
C	-1.26611600	-0.89304600	-2.40010200
C	-1.41235600	-0.49614700	-3.74734500
C	-2.33533400	-0.73508700	-1.52257400
C	-2.64092300	0.01445700	-4.17169800
C	-3.56419000	-0.18364000	-1.91742100

H	-2.18120000	-1.03622900	-0.49016200
C	-3.69519700	0.17316300	-3.26370900
H	-2.78151800	0.32574800	-5.20618200
H	-4.62602700	0.60400900	-3.63129100
C	0.58852500	-1.01271000	-0.80127100
Fe	-0.09765300	0.48713900	0.33867700
C	2.72832600	-1.57512000	0.27442300
C	3.64427800	-0.54138700	0.11549400
C	2.84907200	-2.46031300	1.36574000
C	4.64570600	-0.26750700	1.05701500
H	3.53642400	0.07866200	-0.77104300
C	3.83114600	-2.19398600	2.32761800
C	4.69878200	-1.10577900	2.17554900
H	3.93116500	-2.82982900	3.20604000
H	5.43205700	-0.92694100	2.96182600
O	-0.31552100	-0.62101400	-4.54801600
O	2.00733300	-3.52064800	1.39949000
C	-0.38246100	-0.17548600	-5.88331500
H	-1.13154600	-0.73948900	-6.46642200
H	-0.62435800	0.90020600	-5.94019200
H	0.61241700	-0.34407500	-6.31601900
C	2.09019000	-4.41153500	2.49338700
H	3.09356700	-4.86704700	2.56402000
H	1.84941200	-3.90228700	3.44114000
H	1.34331400	-5.19401200	2.32316100
C	0.65570800	1.67871700	3.05566100
C	-0.05572500	2.89553400	3.07656200
C	0.70078400	0.93410200	4.25063100
C	-0.71158600	3.33051000	4.22573800
H	-0.11205800	3.49978600	2.17283100
C	0.04735600	1.36920100	5.40451200
H	1.25350400	-0.00882900	4.25975600
C	-0.67156200	2.56874900	5.39905900
H	-1.26971500	4.26989600	4.20122600
H	0.09995500	0.76671700	6.31585200
H	-1.18912600	2.90972300	6.29886100
C	-3.45368400	2.63413700	1.54331000
C	-3.12390300	3.52681000	0.48204200
C	-3.92424000	4.62197400	0.15693500
C	-5.05547000	4.85868700	0.94297400
C	-5.37559000	4.01453500	2.02263600
C	-4.58642100	2.90377800	2.32706700
C	-2.46854700	1.58168500	1.47446200
C	-1.56625300	1.84986400	0.42179000

H	-3.67872200	5.26195600	-0.69135400
H	-5.70033600	5.71013200	0.71337500
H	-6.26173900	4.22789600	2.62527700
H	-4.85392200	2.24833600	3.15896600
N	-1.95972300	3.04163300	-0.13869700
C	-1.33099900	3.65472200	-1.26212500
C	-0.68798600	4.90390200	-1.15631100
C	-1.34959200	2.95292600	-2.47365500
C	-0.08568900	5.41878600	-2.31730800
C	-0.73461600	3.48055200	-3.60466400
H	-1.85299100	1.99006800	-2.50509800
C	-0.10186200	4.72485000	-3.52660200
H	0.42147300	6.38520100	-2.26562100
H	-0.75468300	2.92187200	-4.54317200
H	0.38193600	5.15517900	-4.40697200
C	-2.28910700	0.35697700	2.16258400
H	-2.96802100	0.05082000	2.97072000
N	-1.33097200	-0.45089600	1.77275200
C	-1.21474200	-1.70849000	2.39739900
C	-1.20946000	-1.85456500	3.79149900
C	-1.16794600	-2.86995900	1.60460200
C	-1.15437400	-3.12024400	4.38858400
H	-1.22980600	-0.96273100	4.41776900
C	-1.14949000	-4.12630100	2.18935800
H	-1.17108100	-2.77972000	0.51919400
C	-1.12626900	-4.26635600	3.58563100
H	-1.13413000	-3.19103700	5.47604200
H	-1.14518900	-5.02724000	1.57678900
O	-1.04359900	-5.54357200	4.05604900
C	-1.04782700	-5.75054500	5.44768300
H	-0.99545700	-6.83604900	5.60677900
H	-0.17858700	-5.27494100	5.93857500
H	-1.96969600	-5.36322100	5.91832500
H	0.28652700	1.18045500	-0.94197400
C	-0.58661500	5.67210000	0.15478600
C	-1.26507000	7.04801500	0.07430500
C	0.87559100	5.82309200	0.60517400
H	-1.11260500	5.08839400	0.92331100
H	-2.32337000	6.96283700	-0.20853100
H	-1.21699900	7.55707900	1.05010900
H	-0.76678400	7.69630100	-0.66473500
H	1.38219100	4.85025600	0.67379300
H	1.44826400	6.44966800	-0.09745900
H	0.92472100	6.29922100	1.59723900

C	-4.64305300	0.06136800	-0.87344400
H	-4.30391200	0.94087500	-0.30167000
C	-4.79176500	-1.10110000	0.14395100
C	-6.04407800	0.39365300	-1.43542500
H	-3.80923400	-1.36003300	0.56124300
C	-5.39939100	-2.33336600	-0.54619300
C	-5.70511200	-0.65081800	1.29541900
H	-5.96493700	1.22229100	-2.15713000
C	-6.66579200	-0.84170600	-2.11431000
C	-6.95085500	0.84536800	-0.27553400
H	-5.46946000	-3.16588300	0.17438400
H	-4.74359400	-2.67296000	-1.36492000
C	-6.79602600	-1.98366500	-1.09142400
H	-5.26583600	0.22214900	1.80081200
H	-5.78403000	-1.45822500	2.04357900
C	-7.09577400	-0.29293200	0.74886000
H	-6.04833200	-1.17323500	-2.96384600
H	-7.65857000	-0.58173600	-2.52036900
H	-6.52486600	1.73915700	0.20870100
H	-7.94233300	1.12845900	-0.66882700
H	-7.23495400	-2.86920600	-1.58160400
C	-7.70493500	-1.53024900	0.06544800
H	-7.74760100	0.03513900	1.57611200
H	-8.71309700	-1.29485200	-0.31729100
H	-7.82135500	-2.34748900	0.79809200
C	5.53392100	0.95158400	0.85639900
H	4.87536100	1.82603100	1.01510900
C	6.09834100	1.06544200	-0.58626700
C	6.71427800	1.08652700	1.84658600
H	5.28440700	0.95728200	-1.31795400
C	7.14534900	-0.03313600	-0.83181500
C	6.74792800	2.44750600	-0.76666100
H	6.33881800	1.00776200	2.87946300
C	7.77799600	0.00122500	1.58908200
C	7.36265700	2.47153600	1.66254600
H	7.50866900	0.02874500	-1.87115600
H	6.68223800	-1.02600300	-0.71656100
C	8.31482500	0.13131100	0.15383100
H	5.99625700	3.24000600	-0.61229200
H	7.11908900	2.55212100	-1.80055400
C	7.90661400	2.61248600	0.23026900
H	7.35670700	-1.00494400	1.73596000
H	8.60153600	0.11329000	2.31492800
H	6.62283200	3.26365100	1.86667700

H	8.18044800	2.59900000	2.39198400
H	9.06834000	-0.65300600	-0.03020700
C	8.95680200	1.51789100	-0.02988000
H	8.36703500	3.60690600	0.10558200
H	9.80706900	1.63549000	0.66366400
H	9.35983300	1.61681300	-1.05251400
C	-0.23987900	-3.83011400	-2.38120800
C	-1.21670900	-4.13702400	-3.33886400
C	-0.12462800	-4.64093100	-1.24258200
C	-2.06593900	-5.23225400	-3.16801400
H	-1.31217400	-3.50527800	-4.22521900
C	-0.96268500	-5.74749000	-1.08153700
H	0.60948500	-4.40245500	-0.47044000
C	-1.93735200	-6.04417900	-2.03825400
H	-2.82538700	-5.45524100	-3.92117800
H	-0.85445800	-6.38548100	-0.20120200
H	-2.59355300	-6.90723400	-1.90370900
C	3.09945200	-1.95664700	-2.70718000
C	4.37876000	-2.52183200	-2.69471400
C	2.89594800	-0.74362800	-3.38647100
C	5.44403000	-1.89322700	-3.34649300
H	4.54654100	-3.45708100	-2.15417600
C	3.96017500	-0.11237500	-4.03143800
H	1.90467500	-0.28753900	-3.40070900
C	5.23783900	-0.68390600	-4.01222100
H	6.43897000	-2.34308900	-3.32186400
H	3.79301400	0.83584700	-4.54825700
H	6.07060100	-0.18410000	-4.51252900
C	1.33946000	1.16139100	1.86339300
C	1.53146500	1.84163200	0.66151400
H	1.94643100	0.27088200	2.04612600
H	2.36775400	1.58562600	0.01559600
H	1.15713500	2.85731200	0.53293900

int4(quintet)

C	-0.31139000	-3.03460800	-0.50077100
C	1.03884200	-3.23646400	0.22522200
H	-0.25899900	-3.41331100	-1.53018100
H	0.89855000	-3.80288500	1.15665200
N	1.41503700	-1.84350700	0.57624400
N	-0.40775600	-1.55015200	-0.56020400
C	2.12009500	-3.90920500	-0.59356900
C	3.05488300	-4.73469000	0.04371600
C	2.24516200	-3.68161900	-1.97200100

C	4.09172500	-5.33003500	-0.67906400
H	2.97055000	-4.90666400	1.11962500
C	3.27240600	-4.28629200	-2.69863300
H	1.54732800	-3.01570100	-2.48425800
C	4.19982500	-5.11115100	-2.05443000
H	4.81413800	-5.96974100	-0.16678700
H	3.35449400	-4.10442000	-3.77262800
H	5.00603000	-5.57904400	-2.62399400
C	-1.50051800	-3.68144400	0.17232000
C	-2.40187500	-4.44328500	-0.57981100
C	-1.73471000	-3.50255100	1.54346800
C	-3.52312900	-5.02035200	0.02208600
H	-2.23045000	-4.57507600	-1.65110100
C	-2.85461000	-4.07824600	2.14473000
H	-1.04176300	-2.89798900	2.13226200
C	-3.75387500	-4.83576400	1.38671600
H	-4.22149200	-5.60633800	-0.57889200
H	-3.03162000	-3.93444700	3.21320900
H	-4.63354100	-5.27824200	1.85963900
C	2.39961900	-1.58698500	1.56970200
C	1.98570300	-1.46243900	2.91204700
C	3.74002100	-1.44738300	1.23502100
C	2.95126500	-1.22428000	3.88987200
C	4.72028000	-1.15982400	2.19957300
H	4.00976400	-1.53866700	0.18517500
C	4.29575100	-1.07962800	3.52879100
H	2.67092000	-1.12725000	4.93792600
H	5.01556900	-0.88930700	4.32413300
C	0.58531900	-0.90936100	0.07771200
Fe	1.01326800	1.12939200	0.31039000
C	-1.41068100	-0.93794700	-1.35603600
C	-2.74273800	-1.00361600	-0.96222600
C	-1.06348500	-0.33679100	-2.58518300
C	-3.78391200	-0.49839900	-1.75414200
H	-2.96132600	-1.45224400	0.00293200
C	-2.08416700	0.22737400	-3.35774800
C	-3.41939000	0.13617800	-2.94545400
H	-1.85246900	0.72858700	-4.29660200
H	-4.17947300	0.58866700	-3.58191100
O	0.65196600	-1.57707400	3.15728500
O	0.24365200	-0.37472100	-2.93671200
C	0.17869700	-1.25565900	4.44942500
H	0.56103000	-1.95851900	5.21020600
H	0.45989800	-0.22672000	4.72925700

H	-0.91489000	-1.32604700	4.40980400
C	0.65679000	0.32631700	-4.09130500
H	0.19632700	-0.09268500	-5.00352500
H	0.40565200	1.39696300	-4.01541000
H	1.74615600	0.21381800	-4.14663400
C	6.16193400	-0.96427300	1.75407300
H	6.50415400	-1.94563900	1.37475600
C	7.15476600	-0.53166200	2.85882800
C	6.28797500	0.03959100	0.57499300
H	7.07887900	-1.22181400	3.71544900
C	6.88249200	0.91397700	3.32421400
C	8.58481000	-0.60279500	2.28960000
H	5.57974800	-0.23176000	-0.21598700
C	5.98742900	1.46836200	1.05483800
C	7.71466400	-0.02641900	0.00861000
H	7.60712300	1.18544400	4.11132400
H	5.88011400	1.00800000	3.76599100
C	7.00585400	1.87736000	2.13283000
H	8.81327800	-1.63404000	1.97040300
H	9.31225000	-0.34052700	3.07668200
C	8.72654000	0.36102100	1.09809600
H	4.95870700	1.53018300	1.44585900
H	6.04139200	2.15753800	0.19657600
H	7.92284300	-1.03830400	-0.37855700
H	7.79825000	0.66597100	-0.84456600
H	6.79736300	2.90642200	2.47002600
C	8.43291900	1.79923500	1.56175200
H	9.75229200	0.30137700	0.69588400
H	8.54096500	2.49697100	0.71342300
H	9.16496400	2.10820800	2.32794000
C	-5.20697800	-0.62028900	-1.23321900
H	-5.24796800	-0.00977600	-0.31503100
C	-5.56733600	-2.06896400	-0.80496100
C	-6.32014300	-0.10127200	-2.16985500
H	-4.77901600	-2.46570700	-0.15071500
C	-5.70763800	-2.96898300	-2.04208000
C	-6.89172200	-2.04838900	-0.02771200
H	-6.07816700	0.92435200	-2.49367000
C	-6.48854000	-1.01603400	-3.39932700
C	-7.64567000	-0.07793700	-1.38436700
H	-5.92622400	-4.00241800	-1.72413000
H	-4.75704900	-2.99831900	-2.60021200
C	-6.83919300	-2.44183000	-2.94267100
H	-6.78777600	-1.43065300	0.87455400

H	-7.14050200	-3.06969400	0.30905800
C	-8.01219000	-1.50137800	-0.92622700
H	-5.57004700	-1.03940800	-4.00437000
H	-7.28840900	-0.61748700	-4.04677700
H	-7.55161300	0.58521600	-0.50885700
H	-8.44863400	0.33355500	-2.01958100
H	-6.94668600	-3.09483300	-3.82521100
C	-8.16191500	-2.41453400	-2.15573400
H	-8.96056800	-1.47370500	-0.36346900
H	-8.97781700	-2.05164600	-2.80438500
H	-8.43461000	-3.43504400	-1.83591900
C	-2.31264200	0.20705200	2.13538200
C	-3.22262100	-0.54651900	2.76878700
H	-1.25427900	-0.06098000	2.14512500
H	-2.57696700	1.10706900	1.57893900
H	-2.88037400	-1.44180600	3.29579100
C	-4.67358800	-0.32021500	2.84455400
C	-5.48387100	-1.24277000	3.53137600
C	-5.29819600	0.79998100	2.26363300
C	-6.86230800	-1.05021100	3.64637400
H	-5.01953600	-2.12354700	3.98264400
C	-6.67222100	0.99561000	2.37866600
H	-4.69411900	1.53169000	1.73259600
C	-7.46374400	0.07276900	3.07302400
H	-7.46980600	-1.78212100	4.18406700
H	-7.13127000	1.87611000	1.92182600
H	-8.54165500	0.22648600	3.16017700
C	0.32758900	4.80138600	-1.61547900
C	-0.65076700	5.03542700	-0.61046600
C	-1.24366800	6.28788100	-0.43186600
C	-0.88151600	7.31161400	-1.31049300
C	0.06017500	7.09024800	-2.33274800
C	0.67401500	5.84677800	-2.48581400
C	0.78113800	3.44320200	-1.40493900
C	0.07046800	2.87349300	-0.32460700
H	-1.95396600	6.46510000	0.37736200
H	-1.33117700	8.30071300	-1.19564200
H	0.32301300	7.90970000	-3.00624700
H	1.42300400	5.69434300	-3.26726200
N	-0.79880400	3.84387200	0.12006500
C	-1.59606800	3.71379500	1.29320400
C	-2.99768100	3.89270100	1.25458100
C	-0.93841000	3.38792600	2.48521700
C	-3.68546100	3.79576500	2.47439300

C	-1.65751600	3.25342400	3.67177900
H	0.14000200	3.22116200	2.45520600
C	-3.03461500	3.47667400	3.66809600
H	-4.76505800	3.94194500	2.49329000
H	-1.13943000	2.98645300	4.59560100
H	-3.61253100	3.38730700	4.59067000
C	1.88988900	2.74948900	-1.96218100
H	2.42900900	3.19492500	-2.81441400
N	2.28748100	1.60688300	-1.46656100
C	3.44443300	0.99492000	-1.97064900
C	4.58675400	1.72438700	-2.34211700
C	3.48969300	-0.40671000	-2.10080000
C	5.72741800	1.09098600	-2.84427100
H	4.60008900	2.80701000	-2.20380300
C	4.62161300	-1.04417000	-2.59190600
H	2.61187100	-0.98184800	-1.81442400
C	5.75401000	-0.30386500	-2.96778900
H	6.59672100	1.69538800	-3.10197500
H	4.65971500	-2.12958500	-2.67916600
O	6.82441300	-1.02114400	-3.40572400
C	7.96353200	-0.32984500	-3.85936900
H	8.67942600	-1.09041700	-4.19873400
H	7.72560600	0.34227500	-4.70402600
H	8.43162500	0.26496400	-3.05517400
H	1.93565000	1.41105100	1.66921200
C	-3.73446300	4.06832600	-0.06918600
C	-5.17770100	4.55550600	0.08176100
C	-3.69008400	2.76292100	-0.88047700
H	-3.20007600	4.82771700	-0.65623100
H	-5.24038500	5.48652600	0.66635300
H	-5.61198100	4.74918000	-0.91104200
H	-5.81655300	3.80284800	0.57253300
H	-2.66506600	2.39762400	-1.02518100
H	-4.26072100	1.96933700	-0.37741700
H	-4.13659100	2.91024900	-1.87606200

TS5(singlet)

C	1.74691900	-3.21165800	-1.82155200
C	0.27869000	-3.29201300	-2.26544300
H	2.06405900	-4.12662400	-1.30667100
H	0.20373100	-3.36246700	-3.35639600
N	-0.21042000	-1.95737200	-1.83299700
N	1.70600800	-2.09071400	-0.83547500
C	-1.52415000	-1.51679600	-2.11177800

C	-1.95812100	-1.40403100	-3.45143900
C	-2.39488800	-1.17900700	-1.07483900
C	-3.26309300	-0.97924200	-3.70020500
C	-3.69738000	-0.69962100	-1.30851800
H	-2.03364700	-1.29344300	-0.04993600
C	-4.11296200	-0.62952900	-2.64105900
H	-3.62792100	-0.88540700	-4.72260500
H	-5.11206200	-0.27189700	-2.88644500
C	0.59731800	-1.32923000	-0.93644000
Fe	-0.23203800	0.43756700	-0.23025700
C	2.89373200	-1.80224700	-0.11427500
C	3.71375400	-0.73883300	-0.48042900
C	3.30867400	-2.68422000	0.90738100
C	4.91916600	-0.45148300	0.18097700
H	3.39309700	-0.13301400	-1.32689800
C	4.50122800	-2.41221600	1.58350300
C	5.28523700	-1.31016100	1.22242900
H	4.83512300	-3.05837100	2.39432700
H	6.21486800	-1.14840800	1.76677000
O	-1.04618600	-1.71319100	-4.41385600
O	2.53103300	-3.77288900	1.13290200
C	-1.39210200	-1.53140700	-5.76892600
H	-2.23457800	-2.18006400	-6.06659300
H	-1.65802700	-0.48090000	-5.97987100
H	-0.50454600	-1.80365500	-6.35479000
C	2.94700600	-4.72843300	2.08752700
H	3.95710100	-5.10970900	1.85782500
H	2.92587300	-4.32024600	3.11032500
H	2.22914800	-5.55602000	2.02980800
C	1.24810500	1.79275500	-0.80092300
C	1.47054200	1.38963300	0.54667200
H	1.94075400	1.43904400	-1.57079800
H	0.88134800	2.80090400	-1.00907100
H	2.19093100	0.58950200	0.70749700
C	1.25363100	2.19584400	1.75576500
C	0.61719300	3.45060300	1.76141100
C	1.70155600	1.68967400	2.99125400
C	0.41891700	4.15782700	2.94478000
H	0.25356900	3.86701600	0.82651700
C	1.50771200	2.39594400	4.17899000
H	2.19412000	0.71437700	3.01240100
C	0.85873700	3.63470500	4.16590400
H	-0.09361700	5.12289300	2.91462400
H	1.86413900	1.97412000	5.12296200

H	0.70087700	4.18821200	5.09465200
C	-3.14076500	2.80841300	1.66455400
C	-3.14082400	3.48060900	0.40956700
C	-4.06862700	4.47385400	0.09837500
C	-4.98971000	4.83989800	1.08522400
C	-4.98112600	4.21681700	2.34629800
C	-4.06940600	3.20088400	2.64136200
C	-2.14443500	1.77064400	1.54913800
C	-1.53806100	1.83447200	0.26634800
H	-4.08363000	4.93574300	-0.88942700
H	-5.72779300	5.61606600	0.86997600
H	-5.70855200	4.52448600	3.10147100
H	-4.08901700	2.70945900	3.61694700
N	-2.13945500	2.89734500	-0.38573000
C	-1.87160100	3.28354900	-1.73235700
C	-1.33625100	4.54776700	-2.05499700
C	-2.14407300	2.34642600	-2.73777000
C	-1.10782100	4.82603800	-3.41389100
C	-1.89536000	2.64239500	-4.07436000
H	-2.55568400	1.38417300	-2.44732600
C	-1.37726700	3.89468500	-4.41574000
H	-0.69259800	5.79800200	-3.69058300
H	-2.11432700	1.89774100	-4.84315900
H	-1.18120900	4.14711400	-5.46075700
C	-1.76173400	0.72518200	2.42551600
H	-2.13514300	0.70490200	3.46210900
N	-0.99205300	-0.23609200	1.98830700
C	-0.50162900	-1.22770800	2.83755800
C	-0.06441900	-0.97127600	4.14792600
C	-0.42244200	-2.55018700	2.35977900
C	0.45145500	-1.99270000	4.95309500
H	-0.08683400	0.05083700	4.52640100
C	0.06246200	-3.57082800	3.16173800
H	-0.76226900	-2.76456500	1.34716000
C	0.52546700	-3.30079700	4.45912000
H	0.80632300	-1.74762400	5.95417200
H	0.10463200	-4.59830100	2.80077800
O	1.05225100	-4.36095600	5.14134700
C	1.48090400	-4.16118700	6.46644400
H	1.83609300	-5.13327200	6.83467400
H	2.30941300	-3.43157000	6.52734000
H	0.65814600	-3.81070300	7.11576700
H	-0.01402600	1.04947400	-1.60348900
C	-0.56910700	-4.37592600	-1.63579100

C	-1.68179100	-4.85779900	-2.33805200
C	-0.33969500	-4.82830700	-0.32826900
C	-2.55774300	-5.77093800	-1.74631600
H	-1.86669600	-4.50325400	-3.35514300
C	-1.21401500	-5.74357400	0.26226200
H	0.51474700	-4.45035400	0.23442300
C	-2.32626200	-6.21460600	-0.44178000
H	-3.42394600	-6.13465100	-2.30413300
H	-1.03177900	-6.08558600	1.28381700
H	-3.01102400	-6.92576800	0.02609400
C	2.73781400	-2.87075200	-2.91329800
C	4.01596800	-3.43916900	-2.89571700
C	2.43478300	-1.90062000	-3.88279400
C	4.98163600	-3.05285200	-3.82982500
H	4.26153200	-4.18176300	-2.13210600
C	3.39865300	-1.51371700	-4.81477600
H	1.44391700	-1.44206000	-3.90231800
C	4.67498500	-2.08746300	-4.79088500
H	5.97662200	-3.50340900	-3.80206400
H	3.15470900	-0.75529700	-5.56288700
H	5.42845200	-1.78010100	-5.52007000
C	-4.53810400	-0.22130200	-0.13679100
H	-4.06720300	0.72037600	0.18885400
C	-4.50007400	-1.17746200	1.08535800
C	-6.01500000	0.09336600	-0.46295600
H	-3.45763200	-1.41628500	1.33697800
C	-5.27184300	-2.46916200	0.77060800
C	-5.14138200	-0.47256500	2.29175800
H	-6.06425500	0.77381600	-1.32837100
C	-6.79933500	-1.19967000	-0.75880700
C	-6.64625600	0.80230800	0.74977600
H	-5.21109800	-3.15687100	1.63116100
H	-4.81243300	-2.98898400	-0.08691200
C	-6.74257200	-2.13132900	0.46413100
H	-4.58169800	0.44316400	2.52917000
H	-5.08667500	-1.12988600	3.17647800
C	-6.60435200	-0.12492700	1.97670200
H	-6.38614000	-1.71882100	-1.63780400
H	-7.84761200	-0.95011800	-0.99705600
H	-6.10603600	1.73845400	0.96338400
H	-7.69017800	1.07435200	0.51744000
H	-7.29834600	-3.05912300	0.24623300
C	-7.37665000	-1.42112400	1.67421500
H	-7.05827800	0.38805400	2.84118300

H	-8.43592100	-1.19315000	1.46377000
H	-7.35905700	-2.08577200	2.55518000
C	5.78115200	0.70000800	-0.31943900
H	6.08114100	0.41445300	-1.34524000
C	7.08492100	0.96466700	0.47042300
C	5.01198500	2.04364000	-0.43902000
H	7.64547200	0.02183000	0.58228300
C	6.79233600	1.57010600	1.85869200
C	7.94605500	1.96346000	-0.32725200
H	4.06522900	1.88050300	-0.97165400
C	4.71258400	2.61377900	0.95613500
C	5.87040500	3.04190600	-1.23382500
H	7.74449800	1.73815500	2.39085700
H	6.19875700	0.88005100	2.47656300
C	6.02879700	2.89483600	1.69880900
H	8.19250800	1.54257900	-1.31696600
H	8.90169000	2.13237100	0.19761400
C	7.19319400	3.29591400	-0.49232900
H	4.09996300	1.90748500	1.53338100
H	4.11346400	3.53422300	0.86306100
H	6.06950600	2.64842600	-2.24554100
H	5.31883800	3.98906800	-1.35885500
H	5.80706000	3.31479600	2.69380600
C	6.88594600	3.88729600	0.89484000
H	7.81474800	4.00143900	-1.06911500
H	6.35254000	4.84694100	0.78578600
H	7.82571500	4.10016000	1.43315100
C	-0.95116100	5.57999600	-1.00362900
C	-1.73406800	6.89258200	-1.15856300
C	0.56175900	5.85755500	-1.02756200
H	-1.20191400	5.16112500	-0.01915200
H	-2.81862700	6.72733200	-1.10199500
H	-1.45923800	7.59868100	-0.35885500
H	-1.51314500	7.37875000	-2.12264100
H	1.14846800	4.93204700	-0.94003200
H	0.86088200	6.35512500	-1.96396900
H	0.84591100	6.51529300	-0.19101400

TS5(triplet)

C	1.97055300	-2.63437700	-1.91065500
C	0.59636700	-2.61602200	-2.59717500
H	2.25444900	-3.64888400	-1.60161800
H	0.70050800	-2.43973700	-3.67281900
N	-0.01880200	-1.41273500	-1.96984600

N	1.69872100	-1.81092300	-0.70346700
C	-1.25443700	-0.89605700	-2.42131800
C	-1.40751700	-0.50495700	-3.76932200
C	-2.31868200	-0.73772300	-1.53772100
C	-2.63726900	0.00665500	-4.18816200
C	-3.55020200	-0.18935000	-1.92871700
H	-2.15784200	-1.03709800	-0.50528300
C	-3.68634200	0.16734600	-3.27454600
H	-2.78316100	0.31724200	-5.22209400
H	-4.61895800	0.59768700	-3.63806800
C	0.59761800	-1.03411400	-0.82107100
Fe	-0.08110300	0.43598800	0.39218800
C	2.72129600	-1.60842200	0.25974600
C	3.61881800	-0.55802300	0.10410500
C	2.85551700	-2.49432200	1.34817300
C	4.62009500	-0.27431900	1.04300000
H	3.49461900	0.06451500	-0.77877900
C	3.83908400	-2.21985600	2.30648100
C	4.69163600	-1.12000600	2.15536900
H	3.95117900	-2.85794200	3.18181300
H	5.42700100	-0.93604300	2.93846000
O	-0.31388900	-0.63373300	-4.57470900
O	2.02403600	-3.56271600	1.38328000
C	-0.39921700	-0.22599000	-5.92124200
H	-1.15457300	-0.80821700	-6.47765400
H	-0.64526500	0.84704400	-6.00639100
H	0.59040100	-0.40421500	-6.36204600
C	2.12132500	-4.45551800	2.47456900
H	3.12732700	-4.90692600	2.53404000
H	1.88771900	-3.94954500	3.42564800
H	1.37610800	-5.24082200	2.31007900
C	1.60000300	1.92397000	0.62825300
C	1.45504000	1.29194400	1.88117800
H	2.50409600	1.72340000	0.05250700
H	1.22461000	2.94095400	0.49739300
H	2.10877300	0.44313900	2.09564300
C	0.71483100	1.80809900	3.03265000
C	-0.05345400	2.99190200	3.00420900
C	0.74532500	1.08979100	4.24730600
C	-0.77047000	3.41861100	4.11902800
H	-0.10994200	3.57332700	2.08590300
C	0.02946400	1.51754700	5.36519900
H	1.33378700	0.16972800	4.29635300
C	-0.74188500	2.68363600	5.30951600

H	-1.37064300	4.32965800	4.05213600
H	0.07278200	0.93431100	6.28962200
H	-1.30847800	3.01768000	6.18184700
C	-3.46893800	2.62258700	1.53385000
C	-3.13489900	3.50678100	0.46648300
C	-3.94184500	4.59034200	0.12083000
C	-5.08201500	4.82836300	0.89390900
C	-5.40467800	3.99531200	1.98099800
C	-4.61018800	2.89353600	2.30449000
C	-2.48126000	1.57107700	1.48268700
C	-1.56985600	1.83230900	0.43422500
H	-3.69512900	5.22147300	-0.73348000
H	-5.73145600	5.67162200	0.64759000
H	-6.29750000	4.20894800	2.57363600
H	-4.88112200	2.24484000	3.14058700
N	-1.96123100	3.01991900	-0.13670400
C	-1.30417000	3.60923500	-1.25550700
C	-0.71262900	4.88706900	-1.18735800
C	-1.21871800	2.83905100	-2.42262500
C	-0.06502400	5.35931600	-2.34240300
C	-0.55501600	3.32353400	-3.54503800
H	-1.68022300	1.85567500	-2.42360000
C	0.01990300	4.59732200	-3.50705800
H	0.40226700	6.34667200	-2.31884000
H	-0.49145300	2.70676500	-4.44418700
H	0.53942700	4.99649500	-4.38170200
C	-2.30845900	0.35470400	2.18932900
H	-2.98074500	0.06862300	3.01024200
N	-1.36044800	-0.47053600	1.80821600
C	-1.22049500	-1.70870300	2.46168900
C	-1.16072900	-1.81655100	3.85758700
C	-1.18236500	-2.88802300	1.69533200
C	-1.06446700	-3.06627900	4.48280900
H	-1.16629600	-0.90730800	4.45934000
C	-1.12121500	-4.12857200	2.30966900
H	-1.22355300	-2.82128800	0.60858800
C	-1.04611900	-4.23191900	3.70776700
H	-1.00222700	-3.10984300	5.57009700
H	-1.12216100	-5.04561000	1.72095300
O	-0.92569300	-5.49534200	4.20667400
C	-0.87370100	-5.66646900	5.60216100
H	-0.79728400	-6.74657300	5.78635200
H	0.00549300	-5.16473500	6.04720300
H	-1.78333300	-5.28162300	6.09799000

H	0.69616300	1.35719500	-0.59490500
C	-0.69257600	5.72631600	0.08378900
C	-1.37893100	7.08753100	-0.10521600
C	0.74323300	5.92197500	0.59839500
H	-1.24980200	5.17789500	0.85584600
H	-2.41729800	6.97613000	-0.44689200
H	-1.39484300	7.64441300	0.84510200
H	-0.84388900	7.70563200	-0.84434700
H	1.25718800	4.96166200	0.74470900
H	1.34378700	6.51799200	-0.10743200
H	0.73702900	6.44936500	1.56533700
C	-4.62857700	0.04816600	-0.88282700
H	-4.29454900	0.92901200	-0.31038000
C	-4.76741800	-1.11702100	0.13292400
C	-6.03267800	0.37053000	-1.44299900
H	-3.78256800	-1.36787100	0.54928500
C	-5.36585000	-2.35301200	-0.55871000
C	-5.68345600	-0.67567900	1.28567600
H	-5.96080000	1.20102900	-2.16332900
C	-6.64530700	-0.86864200	-2.12335600
C	-6.94212600	0.81312600	-0.28173500
H	-5.42901500	-3.18707600	0.16074600
H	-4.70812000	-2.68678100	-1.37835900
C	-6.76562800	-2.01337800	-1.10232700
H	-5.25063800	0.19994800	1.79180200
H	-5.75523900	-1.48474200	2.03276300
C	-7.07731100	-0.32800300	0.74082200
H	-6.02623300	-1.19396100	-2.97411900
H	-7.64051900	-0.61577400	-2.52795600
H	-6.52287700	1.70940100	0.20361100
H	-7.93608800	1.08896300	-0.67395600
H	-7.19800800	-2.90149500	-1.59371400
C	-7.67721000	-1.56901700	0.05597400
H	-7.73105000	-0.00640900	1.56911500
H	-8.68750900	-1.34106000	-0.32566100
H	-7.78652900	-2.38831100	0.78742300
C	5.48935600	0.95999100	0.85108900
H	4.82214700	1.82368400	1.03148000
C	6.03775000	1.10301100	-0.59496700
C	6.67749700	1.09692600	1.83186300
H	5.21816900	0.99426700	-1.32049600
C	7.09677600	0.02202900	-0.86623200
C	6.66696600	2.49624300	-0.76129000
H	6.31307900	0.99877700	2.86694200

C	7.75271600	0.02974900	1.54864800
C	7.30521600	2.49316200	1.66180300
H	7.44894300	0.10307200	-1.90804400
H	6.64771000	-0.97820900	-0.76067100
C	8.27354000	0.18803500	0.11033400
H	5.90646400	3.27634700	-0.58773300
H	7.02632700	2.62109300	-1.79707700
C	7.83316500	2.66220000	0.22665400
H	7.34579600	-0.98384400	1.68488100
H	8.58173900	0.14220100	2.26813300
H	6.55684000	3.27209000	1.88473300
H	8.12824600	2.62115000	2.38518500
H	9.03551400	-0.58331700	-0.09260200
C	8.89517200	1.58561900	-0.05941300
H	8.27906000	3.66439900	0.11202500
H	9.75051600	1.70478600	0.62756100
H	9.28685600	1.70458000	-1.08431500
C	-0.28227000	-3.82866100	-2.37458800
C	-1.26452200	-4.13003200	-3.32828700
C	-0.19641400	-4.61242700	-1.21427800
C	-2.14886700	-5.19283400	-3.13232300
H	-1.33758700	-3.51867700	-4.23092900
C	-1.07096200	-5.68603600	-1.02728800
H	0.54293000	-4.37755700	-0.44595500
C	-2.05134800	-5.97687400	-1.98005300
H	-2.91232700	-5.41154100	-3.88266800
H	-0.98762000	-6.30245200	-0.12921600
H	-2.73623000	-6.81376000	-1.82478500
C	3.09851000	-2.01937200	-2.71362100
C	4.37449500	-2.59146300	-2.67990000
C	2.90995500	-0.81357200	-3.40984800
C	5.45051300	-1.97693500	-3.32720900
H	4.53065100	-3.52121000	-2.12654700
C	3.98504400	-0.19632200	-4.05055900
H	1.92128600	-0.35274500	-3.44209300
C	5.25887600	-0.77484000	-4.01030100
H	6.44250800	-2.43220400	-3.28624500
H	3.82903600	0.74586800	-4.58176400
H	6.09987000	-0.28650700	-4.50812000

TS5(quintet)

C	-1.76081700	3.21422400	-0.79262100
C	-0.63904800	3.16121900	-1.85123600
H	-1.42155700	3.77815100	0.08847400

H	-1.08184900	3.02085200	-2.84748100
N	0.04275500	1.90035500	-1.47096400
N	-1.84925500	1.78015700	-0.41333500
C	1.34010000	1.56219100	-1.93061100
C	1.65798700	1.65372200	-3.30144600
C	2.31100800	1.17061200	-1.01497000
C	2.96168600	1.35479900	-3.70502000
C	3.62599600	0.86804800	-1.40220400
H	2.01393800	1.10000500	0.03010200
C	3.92477700	0.96577200	-2.76486800
H	3.24018400	1.40951600	-4.75675700
H	4.92400700	0.72712400	-3.12723100
C	-0.73671000	1.08989600	-0.73050000
Fe	-0.16849100	-0.88965200	-0.18690000
C	-3.00226100	1.22035100	0.18701900
C	-3.61239800	0.11637000	-0.40397000
C	-3.56010100	1.79006700	1.34979400
C	-4.74193100	-0.51197500	0.14407500
H	-3.16475700	-0.26724900	-1.32165600
C	-4.69541500	1.18886000	1.90322700
C	-5.26484000	0.05514300	1.31488400
H	-5.14484400	1.59510800	2.80826400
H	-6.13998200	-0.37403200	1.79652700
O	0.65339400	2.02510800	-4.13819000
O	-2.95300000	2.89212700	1.84671600
C	0.94068000	2.21181900	-5.50496000
H	1.71265600	2.98690400	-5.65495500
H	1.27633700	1.27432300	-5.98237800
H	0.00590900	2.53812900	-5.97945600
C	-3.46084400	3.47777800	3.02665400
H	-4.49584900	3.83449600	2.88298900
H	-3.43197500	2.76567200	3.86863900
H	-2.80808500	4.32351900	3.26902700
C	-1.60356100	-2.53947300	-0.34589800
C	-1.57144500	-2.12695400	1.00809800
H	-2.51306200	-2.38710900	-0.92406000
H	-1.04486300	-3.42535900	-0.65457300
H	-2.35165200	-1.43951100	1.34505400
C	-0.80219100	-2.76279600	2.07730900
C	0.04446600	-3.87415300	1.87227800
C	-0.88422600	-2.25094800	3.38960400
C	0.79913000	-4.41442200	2.90935700
H	0.13168900	-4.30517000	0.87733100
C	-0.13204700	-2.79461200	4.42991200

H	-1.53530400	-1.39342200	3.57778000
C	0.72687500	-3.87523900	4.19853000
H	1.46374900	-5.25792600	2.70585200
H	-0.21585100	-2.37041900	5.43495900
H	1.32487600	-4.29504800	5.01051900
C	3.41332200	-2.56079400	1.45127500
C	3.47628300	-3.22818700	0.19551900
C	4.52471800	-4.08859900	-0.13638100
C	5.49420300	-4.33886300	0.83680400
C	5.41549300	-3.73802300	2.10862800
C	4.38793100	-2.84830500	2.42070200
C	2.29155400	-1.65618200	1.35827300
C	1.67039300	-1.82545900	0.10712000
H	4.58691700	-4.53853900	-1.12728100
H	6.32480900	-5.01034600	0.60726500
H	6.17963300	-3.96409800	2.85615300
H	4.34630100	-2.37667600	3.40489100
N	2.38376600	-2.79073700	-0.56715600
C	2.10903200	-3.18668100	-1.91016300
C	1.81196600	-4.52185000	-2.25279700
C	2.11447600	-2.17993000	-2.88511900
C	1.56431200	-4.79874800	-3.60837100
C	1.84869700	-2.48013000	-4.21712700
H	2.33361600	-1.16117500	-2.57570500
C	1.58160800	-3.80211500	-4.58319100
H	1.33269500	-5.82504800	-3.90298300
H	1.85667800	-1.68221600	-4.96314100
H	1.37830100	-4.05753100	-5.62607500
C	1.84099600	-0.65422000	2.26944000
H	2.33605900	-0.56757700	3.25185000
N	0.91583700	0.18678100	1.92005800
C	0.49189600	1.19981400	2.78115600
C	0.23675500	1.01740600	4.14974600
C	0.30164100	2.48821500	2.24271100
C	-0.20056300	2.07929500	4.95134800
H	0.35728800	0.02576600	4.58648600
C	-0.09997000	3.55163900	3.03358600
H	0.49385400	2.63480600	1.18242500
C	-0.37276000	3.35394500	4.39633500
H	-0.40773600	1.89338900	6.00526500
H	-0.22995100	4.55004500	2.61120900
O	-0.82849900	4.44422100	5.08030800
C	-1.10079200	4.31215700	6.45421400
H	-1.43441600	5.29728000	6.80726100

H	-1.90022700	3.57289100	6.64733000
H	-0.20290500	4.01236600	7.02443200
H	-0.84297600	-1.46485200	-1.59953400
C	1.67304100	-5.63545600	-1.22280300
C	2.64525000	-6.79790200	-1.47289300
C	0.22734900	-6.15867400	-1.17509100
H	1.90610100	-5.21043000	-0.23680200
H	3.69088100	-6.46133200	-1.48477700
H	2.54547100	-7.55762100	-0.68140000
H	2.43776100	-7.29205300	-2.43582400
H	-0.49499100	-5.34373400	-1.02711100
H	-0.04258900	-6.67271600	-2.11140100
H	0.10239200	-6.87519500	-0.34793200
C	0.25185800	4.37311000	-1.88953200
C	0.06371600	5.32901300	-2.89535200
C	1.25361600	4.58430700	-0.93114600
C	0.85386300	6.48023200	-2.94078800
H	-0.70982700	5.16454500	-3.64955700
C	2.05063400	5.72904600	-0.97964300
H	1.42976200	3.84252200	-0.15246900
C	1.85170000	6.68168000	-1.98363700
H	0.69366900	7.21881700	-3.72991900
H	2.83475000	5.87185600	-0.23248400
H	2.47716600	7.57668600	-2.02251000
C	-3.05781800	3.79683200	-1.28208400
C	-3.54544600	4.98547400	-0.72865300
C	-3.79360300	3.16256600	-2.29366000
C	-4.74535600	5.54002700	-1.18198200
H	-2.98013500	5.47352700	0.06850700
C	-4.99522700	3.71005300	-2.74322600
H	-3.42974000	2.22444700	-2.71906300
C	-5.47345700	4.90233000	-2.18910500
H	-5.11445800	6.47038600	-0.74380200
H	-5.56465300	3.20246600	-3.52541300
H	-6.41544900	5.33041700	-2.54011500
C	4.61466400	0.42171200	-0.33925200
H	4.26370300	-0.56618300	-0.00239700
C	4.61010300	1.34280800	0.91201900
C	6.07614800	0.24968500	-0.80835500
H	3.57693900	1.48721300	1.25937900
C	5.23785800	2.70426300	0.57016600
C	5.41572700	0.67110800	2.03480600
H	6.10085200	-0.39835000	-1.69966200
C	6.72303200	1.61236500	-1.12642000

C	6.87281600	-0.43308300	0.31958600
H	5.19661100	3.36430400	1.45363300
H	4.66227000	3.19931000	-0.23028900
C	6.69894100	2.50364600	0.12669900
H	4.95718300	-0.29224400	2.29738100
H	5.39242800	1.30391700	2.93885000
C	6.86645800	0.45493700	1.57687900
H	6.19535500	2.11950100	-1.94850800
H	7.76333700	1.45745300	-1.46073600
H	6.43404400	-1.41714600	0.54891000
H	7.91066800	-0.60825000	-0.01220400
H	7.15236400	3.48162700	-0.10812100
C	7.49747900	1.82028900	1.25157600
H	7.43886300	-0.04013900	2.37930600
H	8.54836900	1.69044400	0.94011600
H	7.50499200	2.45738200	2.15289400
C	-5.29059200	-1.73545100	-0.56547300
H	-4.61785500	-1.87834600	-1.42639200
C	-6.71178600	-1.59095800	-1.17461000
C	-5.23124100	-3.06509600	0.23747700
H	-6.77476400	-0.62727900	-1.70637200
C	-7.81052800	-1.66628300	-0.09995100
C	-6.91696600	-2.75082300	-2.16872400
H	-4.24258400	-3.15247100	0.71192200
C	-6.33260200	-3.14140100	1.30903800
C	-5.43695900	-4.22245900	-0.75839400
H	-8.79826500	-1.57733500	-0.58395000
H	-7.73463400	-0.82080000	0.59962600
C	-7.71769800	-3.00741500	0.65099600
H	-6.15980500	-2.70044200	-2.96946400
H	-7.90419700	-2.65988900	-2.65323900
C	-6.81796200	-4.09772100	-1.42857200
H	-6.18798600	-2.36960500	2.07889800
H	-6.26000500	-4.11021900	1.83168000
H	-4.63918700	-4.21042100	-1.52014800
H	-5.36050800	-5.18804300	-0.23059100
H	-8.50223500	-3.05221100	1.42504200
C	-7.91345800	-4.16113500	-0.34886000
H	-6.95673300	-4.92342700	-2.14653100
H	-7.87004200	-5.12951500	0.17810500
H	-8.91074200	-4.09289700	-0.81649400

int6(singlet)

C	0.52472300	-3.11062200	-1.14802400

C	-0.90793700	-2.78601500	-1.63450400
H	0.54340300	-4.06526200	-0.60684600
H	-0.95962800	-2.83047400	-2.73145400
N	-1.03140400	-1.36188600	-1.22347300
N	0.78096400	-2.00122300	-0.20831300
C	-1.95035000	-3.72672600	-1.06236800
C	-2.89074300	-4.32768200	-1.90868000
C	-1.98689500	-4.01499800	0.31023100
C	-3.87498500	-5.17631900	-1.39570900
H	-2.86508900	-4.10816000	-2.97917300
C	-2.96508500	-4.87041900	0.82179200
H	-1.25800900	-3.56008800	0.98417700
C	-3.91763200	-5.44604100	-0.02541200
H	-4.60887300	-5.62768500	-2.06738600
H	-2.98292000	-5.09094200	1.89196400
H	-4.68524900	-6.10928500	0.38002900
C	1.55817100	-3.14774700	-2.25401700
C	2.29229400	-4.31165600	-2.50268500
C	1.80545700	-2.00296100	-3.02852500
C	3.26029100	-4.33971100	-3.51199100
H	2.11292600	-5.20114900	-1.89297600
C	2.77637700	-2.02750100	-4.02905100
H	1.24973800	-1.08433300	-2.83225300
C	3.50595300	-3.19668700	-4.27454400
H	3.83327700	-5.25248300	-3.69085900
H	2.97166400	-1.12584400	-4.61398000
H	4.27111200	-3.21161000	-5.05407500
C	-2.12733900	-0.55769400	-1.61825000
C	-1.90444700	0.75765900	-2.05792500
C	-3.44280800	-1.01203100	-1.51850500
C	-2.97670300	1.56045300	-2.43450200
C	-4.54467600	-0.21605200	-1.86366500
H	-3.61718800	-2.00927900	-1.13273000
C	-4.28403100	1.07123900	-2.34515300
H	-2.80602800	2.58359200	-2.76503600
H	-5.09939300	1.72875600	-2.64411500
C	-0.02017400	-0.92431300	-0.40705000
Fe	0.24897500	1.01813000	-0.07098000
C	1.80633100	-2.13620700	0.76988400
C	3.15060000	-2.07091600	0.40508000
C	1.45638900	-2.33296600	2.11944800
C	4.18173300	-2.12737600	1.34633300
H	3.37251000	-1.89687200	-0.64114500
C	2.47791700	-2.44024200	3.07106300

C	3.81149200	-2.32089900	2.68379900
H	2.23875200	-2.57341900	4.12500400
H	4.58210100	-2.34355300	3.45793400
O	-0.60428400	1.19380200	-2.02846800
O	0.13201400	-2.37715500	2.41946100
C	-0.25450300	2.26689300	-2.90668900
H	-0.53078800	1.98915200	-3.93565000
H	-0.75507200	3.19910600	-2.61640400
H	0.82427800	2.39126500	-2.81522200
C	-0.25644400	-2.35124600	3.77858100
H	0.06278200	-3.26519400	4.30973900
H	0.16136400	-1.47012000	4.29385100
H	-1.34999700	-2.28027600	3.78497900
C	-5.94316100	-0.80253700	-1.71248100
H	-6.03025700	-1.58693900	-2.48796400
C	-7.10907200	0.18898100	-1.93920200
C	-6.16778400	-1.50159800	-0.34177200
H	-6.97204300	0.70338200	-2.90419800
C	-7.18867000	1.22419200	-0.79909200
C	-8.43095500	-0.60099700	-1.98371300
H	-5.34506500	-2.20408200	-0.14164700
C	-6.22943600	-0.45768700	0.78468300
C	-7.48835400	-2.28780800	-0.38797500
H	-8.02021600	1.92231600	-0.99611300
H	-6.26981500	1.82704700	-0.74859400
C	-7.40387300	0.50371900	0.54135400
H	-8.40625800	-1.33183300	-2.80999500
H	-9.26837300	0.08730100	-2.18840600
C	-8.65898300	-1.32460400	-0.64496900
H	-5.28087500	0.09744500	0.84216400
H	-6.35367800	-0.96428600	1.75438400
H	-7.44431200	-3.05651200	-1.17864200
H	-7.63739100	-2.81798900	0.56851100
H	-7.44638600	1.24506100	1.35640300
C	-8.72123900	-0.28973800	0.49270500
H	-9.60518800	-1.88995700	-0.68693200
H	-8.89228600	-0.79863600	1.45678200
H	-9.57184800	0.39536500	0.33483700
C	5.63364900	-1.84614600	1.00124300
H	5.88960600	-0.94908900	1.59282300
C	5.91923700	-1.49166400	-0.47641100
C	6.61286600	-2.96897200	1.43204700
H	5.21763800	-0.70800100	-0.80128200
C	5.79675400	-2.72442400	-1.39255800

C	7.35830100	-0.95186400	-0.57280900
H	6.40319500	-3.25689100	2.47476600
C	6.45169700	-4.19675100	0.51966400
C	8.05242400	-2.43532400	1.33284100
H	6.01029900	-2.42944800	-2.43363300
H	4.77627200	-3.12904300	-1.39280000
C	6.77930900	-3.81290500	-0.93463900
H	7.46348900	-0.04998300	0.05354000
H	7.57484000	-0.64979900	-1.61156700
C	8.35742000	-2.03283200	-0.12006400
H	5.42008300	-4.58117200	0.58488400
H	7.12099400	-5.00505300	0.86073000
H	8.17800100	-1.56790500	2.00280800
H	8.76509400	-3.20841200	1.66790300
H	6.67547800	-4.69923700	-1.58342700
C	8.21752000	-3.27289300	-1.02121200
H	9.38534600	-1.63851900	-0.18772600
H	8.93705700	-4.04938000	-0.70880900
H	8.45861000	-3.01190800	-2.06596000
C	2.81966900	1.07683700	0.58844700
C	4.11925900	1.56555100	0.81812600
C	2.02052700	0.82048700	1.72592200
C	4.62150800	1.68763700	2.11637900
H	4.75331500	1.82807600	-0.02991100
C	2.50030900	0.98198400	3.02348300
H	0.99934100	0.39582300	1.62090200
C	3.82372300	1.38759600	3.22547900
H	5.64945200	2.03076300	2.26461600
H	1.85168800	0.76830600	3.87650600
H	4.22088500	1.49107000	4.23773800
C	-1.50802200	4.74815200	-0.39869700
C	-0.23801600	5.26433900	-0.76393400
C	-0.07170300	6.57195800	-1.22327000
C	-1.21545100	7.36552000	-1.35113300
C	-2.48711000	6.86481700	-1.01562600
C	-2.64316000	5.56333300	-0.53599300
C	-1.25012800	3.40459600	0.05707700
C	0.13285600	3.08556200	-0.07028600
H	0.91652200	6.96085100	-1.47161500
H	-1.11844400	8.39038600	-1.71715900
H	-3.36357500	7.50783300	-1.12850100
H	-3.63375200	5.18646300	-0.26858600
N	0.71752400	4.25308900	-0.53034700
C	2.10400800	4.58265100	-0.61670700

C	2.88298800	4.78028100	0.54473100
C	2.63254300	4.81877500	-1.89074700
C	4.21929300	5.16561800	0.34687000
C	3.95862600	5.21405500	-2.05602200
H	1.98023000	4.68289900	-2.75510300
C	4.75716500	5.37596000	-0.92428200
H	4.86279100	5.31431600	1.21275800
H	4.36222700	5.38560200	-3.05633300
H	5.80238900	5.67834800	-1.02550600
C	-2.09786600	2.46336800	0.67246100
H	-3.13195000	2.73901300	0.92950600
N	-1.64167500	1.26891800	0.96900300
C	-2.43369000	0.48690700	1.83122400
C	-3.13393200	1.07500200	2.91191100
C	-2.58291000	-0.89342300	1.65294300
C	-3.99486200	0.33247900	3.71263000
H	-2.98331500	2.13343800	3.13154500
C	-3.45194800	-1.64590200	2.44390100
H	-2.03079200	-1.38773700	0.86683700
C	-4.18462800	-1.03493200	3.46767900
H	-4.54066000	0.79723300	4.53598100
H	-3.56896700	-2.70403900	2.22220500
O	-5.09616500	-1.68050900	4.24868100
C	-5.32234800	-3.04912800	4.01657000
H	-6.09175700	-3.36931500	4.73180600
H	-5.68479400	-3.23839500	2.98949900
H	-4.40915200	-3.65151100	4.17980100
C	2.30420600	4.61196500	1.94778800
C	1.14432800	5.58602300	2.23206400
C	3.34704200	4.77050200	3.05876600
H	1.90529100	3.58589300	1.99849000
H	0.26478700	5.40825000	1.60563200
H	0.82634100	5.48192700	3.28154800
H	1.46558800	6.62946100	2.07985700
H	4.21881100	4.12309100	2.91181700
H	3.69284800	5.81526300	3.13518200
H	2.89765200	4.49821200	4.02548200
C	2.19802900	0.81124700	-0.73464000
H	2.23793800	-0.27077100	-0.88396400
C	2.91539300	1.39720600	-1.93989200
H	2.42733000	1.08462200	-2.87785400
H	2.95961000	2.49210300	-1.93315500
H	3.96277400	1.04174600	-2.02019600

int6(triplet)

C	0.29487000	-3.08325600	-1.04840200
C	-1.09874700	-2.60118500	-1.51221800
H	0.22297300	-4.04594200	-0.52537900
H	-1.16241700	-2.60428800	-2.60917700
N	-1.08790100	-1.18842400	-1.04857300
N	0.66928700	-2.02524300	-0.08133800
C	-2.24934500	-3.41107300	-0.95376100
C	-3.30380900	-3.79864600	-1.78790500
C	-2.28896000	-3.76293500	0.40458800
C	-4.38721000	-4.52074000	-1.28050800
H	-3.28112700	-3.52113800	-2.84470300
C	-3.37215000	-4.48185200	0.91192800
H	-1.47619000	-3.46291200	1.06921100
C	-4.42664900	-4.85971500	0.07335500
H	-5.20432600	-4.81198900	-1.94335000
H	-3.39391500	-4.75410100	1.97009800
H	-5.27471500	-5.41952900	0.47452500
C	1.30565800	-3.19998700	-2.16816100
C	1.99448300	-4.39700300	-2.38740800
C	1.57503000	-2.09307300	-2.98846900
C	2.93881000	-4.49502400	-3.41455400
H	1.79588900	-5.25791800	-1.74359500
C	2.52470300	-2.18678900	-4.00520000
H	1.04314500	-1.15384900	-2.82574600
C	3.20766300	-3.38912700	-4.22284800
H	3.47485300	-5.43365100	-3.57295100
H	2.73588700	-1.31445500	-4.62798100
H	3.95428500	-3.45962500	-5.01716600
C	-2.15970700	-0.31151500	-1.38457100
C	-1.96935800	0.77495300	-2.26495400
C	-3.41239900	-0.52198800	-0.81265800
C	-3.05465400	1.62056100	-2.52135200
C	-4.50589600	0.32099200	-1.04425500
H	-3.50971200	-1.34241400	-0.11158700
C	-4.29513900	1.39554500	-1.91590000
H	-2.93465800	2.48620100	-3.17004700
H	-5.10008500	2.09977100	-2.12850400
C	-0.06542500	-0.89259000	-0.20643300
Fe	0.33659000	1.04578400	0.25123100
C	1.74221700	-2.24316700	0.82817000
C	3.06624000	-2.19991000	0.38680700
C	1.46967500	-2.51425800	2.18311100
C	4.15051500	-2.32364800	1.25979600

H	3.23289000	-1.99301600	-0.66384300
C	2.54402800	-2.68820600	3.06594200
C	3.85563800	-2.57767100	2.60533900
H	2.36601700	-2.88443400	4.12226200
H	4.66916200	-2.65541300	3.33007800
O	-0.72999300	0.95441100	-2.78170200
O	0.16297000	-2.58939100	2.55672900
C	-0.50264900	2.10199700	-3.58307200
H	-1.13549100	2.08803000	-4.48690800
H	-0.69096800	3.02566400	-3.01358400
H	0.55164300	2.07025200	-3.87628200
C	-0.13866900	-2.71777200	3.93088300
H	0.23231300	-3.67348000	4.34049500
H	0.28758500	-1.88450900	4.51471500
H	-1.23046400	-2.68230100	4.01616200
C	-5.78753100	0.10666500	-0.24952300
H	-5.55540400	0.45121800	0.77238300
C	-6.20684800	-1.38209900	-0.12880500
C	-7.01126700	0.91605900	-0.73492900
H	-5.35212300	-1.98412700	0.21004800
C	-6.69563400	-1.90526100	-1.48862400
C	-7.33540800	-1.50643300	0.90863700
H	-6.73928900	1.97998500	-0.82475400
C	-7.51136600	0.38616700	-2.09159500
C	-8.13889100	0.78656400	0.30595600
H	-6.96238700	-2.97164900	-1.40116600
H	-5.88523000	-1.83774600	-2.23242700
C	-7.91676300	-1.09108800	-1.95045600
H	-6.98503600	-1.15576600	1.89239300
H	-7.61560200	-2.56818200	1.02412400
C	-8.55340200	-0.68743600	0.45146100
H	-6.72877000	0.48591500	-2.85993300
H	-8.37347200	0.98581300	-2.43053300
H	-7.79887800	1.18058300	1.27845600
H	-9.00514400	1.39502000	-0.00515300
H	-8.26941800	-1.47116200	-2.92408800
C	-9.04281800	-1.21706900	-0.90832200
H	-9.36169700	-0.77269900	1.19708400
H	-9.92940500	-0.64963500	-1.23983300
H	-9.35370200	-2.27177800	-0.81276400
C	5.58025600	-2.02191400	0.84517300
H	5.85144800	-1.12771600	1.43376100
C	5.78495400	-1.64477300	-0.64008900
C	6.59380400	-3.13652000	1.21124700

H	5.06385300	-0.86068000	-0.91534500
C	5.62280400	-2.86887600	-1.55953300
C	7.21035300	-1.08733800	-0.80407900
H	6.44547100	-3.43891100	2.26042800
C	6.39924700	-4.35629700	0.29471200
C	8.01968400	-2.58380100	1.04249900
H	5.77094600	-2.56334000	-2.60883700
H	4.60752900	-3.28308800	-1.50158400
C	6.64162200	-3.95103500	-1.17036200
H	7.33395900	-0.19232000	-0.17260200
H	7.36765200	-0.76989900	-1.84903200
C	8.24290700	-2.16249400	-0.41980700
H	5.37789700	-4.75591700	0.41131200
H	7.09691700	-5.15914900	0.58876600
H	8.16894000	-1.72132800	1.71375800
H	8.75775400	-3.35161100	1.33182800
H	6.51264800	-4.83094000	-1.82349100
C	8.06722300	-3.39321300	-1.32765800
H	9.26203100	-1.75696200	-0.53703600
H	8.81063200	-4.16576700	-1.06511900
H	8.24830500	-3.11650400	-2.38047700
C	3.22858000	1.11979900	0.48870000
C	4.46472600	1.72863600	0.19899400
C	3.01148900	0.73463700	1.82915000
C	5.44416700	1.89782600	1.17978800
H	4.67066900	2.07530700	-0.81306400
C	3.98610100	0.89572700	2.81105800
H	2.06229000	0.26087800	2.09480200
C	5.22057000	1.47249000	2.49156000
H	6.39132300	2.37377600	0.91328100
H	3.78157600	0.56358300	3.83197900
H	5.98899600	1.60190900	3.25759500
C	-1.17689600	4.65856800	-0.85948700
C	0.15872300	5.11100300	-1.03794500
C	0.44515300	6.35810900	-1.59516400
C	-0.63033300	7.14338700	-2.02220000
C	-1.95710700	6.69799200	-1.88250600
C	-2.23999200	5.46242900	-1.29704400
C	-1.05799300	3.37792800	-0.20394700
C	0.31205900	3.03408200	-0.05198800
H	1.47257800	6.70974600	-1.69320100
H	-0.43377000	8.11939100	-2.47216500
H	-2.77604900	7.33242000	-2.23034000
H	-3.27343000	5.12734300	-1.17763000

N	1.02529400	4.11717200	-0.52901000
C	2.39240800	4.42914900	-0.25046100
C	2.83628400	4.57165600	1.08420000
C	3.24591800	4.69706400	-1.32526100
C	4.16514800	4.97417700	1.27325100
C	4.56190700	5.10392800	-1.10652600
H	2.86224100	4.58373400	-2.34022400
C	5.02048200	5.24170000	0.20273400
H	4.54858300	5.07844400	2.28769900
H	5.22077900	5.30675300	-1.95389400
H	6.04968200	5.55323000	0.39662000
C	-2.00015700	2.55877500	0.44911900
H	-3.06316900	2.83220200	0.45587600
N	-1.58221300	1.47628100	1.07535500
C	-2.48498000	0.79199100	1.90479700
C	-3.58099800	1.43985200	2.52238500
C	-2.34010700	-0.58183700	2.13594700
C	-4.54032800	0.72506600	3.22838800
H	-3.68167200	2.52340600	2.45026300
C	-3.31000900	-1.31044100	2.82899800
H	-1.47264900	-1.09470900	1.73042000
C	-4.43720100	-0.66830000	3.35536600
H	-5.39301200	1.22926500	3.68716100
H	-3.19047300	-2.38795200	2.92095000
O	-5.46132000	-1.30193800	3.99281400
C	-5.44467700	-2.70810700	4.04928600
H	-6.37040400	-3.01517800	4.55372100
H	-5.41829100	-3.15535500	3.03921900
H	-4.58127300	-3.08670600	4.62700600
C	1.89905800	4.33931900	2.26278800
C	0.86011500	5.46947300	2.37600500
C	2.62091500	4.15361000	3.59909600
H	1.35512900	3.40631600	2.04868000
H	0.25875600	5.58075400	1.46478000
H	0.16902300	5.26730600	3.20999100
H	1.35826600	6.43331200	2.57238800
H	3.39008600	3.37218300	3.53826700
H	3.09674500	5.08751700	3.94186200
H	1.89499200	3.85769300	4.37263900
C	2.21064200	0.77693800	-0.55776100
H	2.26885100	-0.30908500	-0.65657800
C	2.47500100	1.32006400	-1.95493200
H	1.77440400	0.86286000	-2.66485800
H	2.35565600	2.40716800	-2.02921700

H	3.49254600	1.07583600	-2.31817800

int6(quinet)

C	-1.62813900	3.24556700	-0.86279000
C	-0.49971200	3.14482400	-1.90813300
H	-1.28230500	3.81866400	0.01004300
H	-0.93282900	3.00936700	-2.90904700
N	0.14221700	1.86959600	-1.50274300
N	-1.76041000	1.82384000	-0.45581600
C	1.45064500	1.52060700	-1.91793500
C	1.81107900	1.60345600	-3.27928300
C	2.39315200	1.12122200	-0.97499800
C	3.12212000	1.28857000	-3.64450600
C	3.71003200	0.78473400	-1.32505900
H	2.07442900	1.07071700	0.06366700
C	4.04966600	0.87862100	-2.67862100
H	3.43135100	1.33877800	-4.68780800
H	5.05405900	0.62092100	-3.01263600
C	-0.65669700	1.09533800	-0.73398100
Fe	-0.10809800	-0.72172400	0.13870200
C	-2.93398800	1.32704600	0.16252300
C	-3.59580100	0.23621900	-0.39551800
C	-3.46733200	1.95461400	1.30688800
C	-4.75972600	-0.31601700	0.16045500
H	-3.16111300	-0.20779200	-1.29183000
C	-4.63757200	1.43204100	1.86749400
C	-5.26347700	0.31471100	1.30688000
H	-5.07124700	1.88859200	2.75602800
H	-6.16442700	-0.05265800	1.79211800
O	0.83429300	1.97664200	-4.14914200
O	-2.80522000	3.03645500	1.78225300
C	1.17501000	2.19886800	-5.49848000
H	1.95046200	2.97861800	-5.59660200
H	1.53156900	1.27506700	-5.98743500
H	0.25886700	2.53588500	-6.00110700
C	-3.27937600	3.66350300	2.95458400
H	-4.29194300	4.07799900	2.80601100
H	-3.29202200	2.96096200	3.80485100
H	-2.57977600	4.47352000	3.18871700
C	-1.83984100	-2.92024100	-0.50910500
C	-1.58640100	-2.08079100	0.73802800
H	-2.41178100	-3.84682400	-0.30014500
H	-0.90478600	-3.23362600	-1.00221500
H	-2.47579300	-1.51492000	1.04392200

C	-0.99032200	-2.76502800	1.89874900
C	-0.18161800	-3.91871700	1.77862300
C	-1.15025800	-2.23538800	3.20107700
C	0.46196000	-4.48024100	2.88087500
H	-0.03039700	-4.36354000	0.79676800
C	-0.51197500	-2.79879500	4.30299900
H	-1.77516800	-1.34687300	3.33061700
C	0.31156100	-3.92337700	4.15395100
H	1.09848200	-5.35808300	2.74016300
H	-0.66227000	-2.36207200	5.29523600
H	0.81705200	-4.36196900	5.01753000
C	3.35305600	-2.75026800	1.37579000
C	3.36672100	-3.35870100	0.08893200
C	4.37749900	-4.23606000	-0.30419600
C	5.35647500	-4.56760300	0.63611000
C	5.32295100	-4.02799400	1.93548100
C	4.33253200	-3.11903700	2.31059500
C	2.27171900	-1.79218100	1.34020800
C	1.63228300	-1.86161200	0.08015100
H	4.40713700	-4.63813800	-1.31660100
H	6.15801600	-5.25529800	0.35672400
H	6.09261400	-4.31548200	2.65573200
H	4.32718300	-2.69272900	3.31615500
N	2.28118200	-2.83589900	-0.63721100
C	1.95298600	-3.15818700	-1.98757600
C	1.61522600	-4.46646500	-2.39418500
C	1.93073300	-2.09528500	-2.90217700
C	1.29319400	-4.65356100	-3.74998600
C	1.58708200	-2.30733700	-4.23330100
H	2.19196700	-1.10303600	-2.54408200
C	1.27460900	-3.60023100	-4.66300400
H	1.02754400	-5.65616800	-4.09298000
H	1.57308100	-1.46479400	-4.92855000
H	1.00990200	-3.78875400	-5.70628700
C	1.85090800	-0.81102200	2.27655900
H	2.37299400	-0.70846700	3.24024300
N	0.89006200	0.02025500	1.95938100
C	0.51807300	1.06164900	2.82114800
C	0.27083300	0.88364900	4.18948900
C	0.38261600	2.35648600	2.28576600
C	-0.10837500	1.96172900	4.99941700
H	0.34641400	-0.11655600	4.61734800
C	0.03713600	3.43401800	3.08442600
H	0.56805200	2.49912500	1.22331000

C	-0.22970900	3.24455800	4.44958000
H	-0.31320700	1.78135100	6.05462600
H	-0.05118200	4.43850300	2.66617700
O	-0.62519100	4.35226900	5.14108700
C	-0.89605400	4.22753100	6.51626500
H	-1.18127200	5.22581900	6.87458000
H	-1.72815600	3.52578400	6.71058800
H	-0.01006100	3.88400100	7.08036600
H	-2.40913800	-2.35391200	-1.26240700
C	1.51374700	-5.64610300	-1.43478400
C	2.46328200	-6.79297800	-1.81417200
C	0.06928000	-6.16837300	-1.35079300
H	1.79618200	-5.29137800	-0.43416300
H	3.50912600	-6.46170500	-1.87153100
H	2.40639300	-7.59939500	-1.06600100
H	2.19343200	-7.22651700	-2.79079200
H	-0.63791000	-5.36851600	-1.09716300
H	-0.25200200	-6.60858400	-2.30850700
H	-0.01012800	-6.95005800	-0.57874600
C	0.44040900	4.31961200	-1.94251900
C	0.41074100	5.20066400	-3.02948100
C	1.34985100	4.55597000	-0.90115200
C	1.26793800	6.30336300	-3.07591800
H	-0.28971300	5.01504800	-3.84733500
C	2.21316300	5.65095100	-0.94864000
H	1.39979700	3.86994700	-0.05497900
C	2.17347300	6.52923700	-2.03644900
H	1.23189700	6.98469000	-3.92937500
H	2.92412600	5.81384900	-0.13536000
H	2.85103600	7.38546100	-2.07493000
C	-2.90444700	3.85501100	-1.37471800
C	-3.35749600	5.07224300	-0.85538000
C	-3.65458200	3.21780200	-2.37359300
C	-4.53702200	5.65200100	-1.33026000
H	-2.78171000	5.56296700	-0.06736700
C	-4.83609200	3.79036100	-2.84455800
H	-3.31862100	2.25736600	-2.77114300
C	-5.27955400	5.01113400	-2.32469200
H	-4.87926300	6.60440000	-0.91841900
H	-5.41744400	3.28009500	-3.61615900
H	-6.20606700	5.45879800	-2.69207900
C	4.65781200	0.29883800	-0.24137200
H	4.29206900	-0.69949800	0.04659600
C	4.61458800	1.17399900	1.04081400

C	6.13403700	0.13770200	-0.66726400
H	3.57199100	1.30885700	1.36256200
C	5.25363900	2.54566600	0.76709700
C	5.38368700	0.46097400	2.16408300
H	6.18756700	-0.48110000	-1.57761300
C	6.78951500	1.50943000	-0.92006800
C	6.89448500	-0.58406100	0.46110500
H	5.18598600	3.17397700	1.67175800
H	4.70300200	3.06989300	-0.03224300
C	6.72727600	2.35789600	0.36143400
H	4.91789800	-0.51196900	2.37524600
H	5.32944200	1.05976800	3.08964700
C	6.84879700	0.26088100	1.74651800
H	6.28518400	2.04293400	-1.74039500
H	7.83939400	1.36591700	-1.22841500
H	6.44811900	-1.57481900	0.64280900
H	7.94198600	-0.74934000	0.15577900
H	7.18820100	3.34256200	0.17419200
C	7.48977000	1.63569800	1.48712000
H	7.39563300	-0.26092300	2.54981200
H	8.54988100	1.51460900	1.20491800
H	7.46918500	2.24222900	2.40905100
C	-5.35800500	-1.53709100	-0.51165800
H	-4.68131600	-1.74504000	-1.35491200
C	-6.76422600	-1.34836700	-1.14261000
C	-5.37030100	-2.83735600	0.33919300
H	-6.77671700	-0.40230000	-1.70868900
C	-7.87679000	-1.33412400	-0.07960100
C	-7.01292000	-2.53229200	-2.09771500
H	-4.39241800	-2.95238000	0.82886600
C	-6.48563800	-2.82496200	1.39896100
C	-5.61870100	-4.01902500	-0.61765500
H	-8.85415300	-1.21596500	-0.57833600
H	-7.76798500	-0.46886600	0.59097700
C	-7.85568100	-2.65045700	0.71919700
H	-6.24518300	-2.54620400	-2.88977000
H	-7.98955300	-2.41323700	-2.59754400
C	-6.98507700	-3.85470900	-1.30889300
H	-6.31389700	-2.03376500	2.14317000
H	-6.46385800	-3.77702000	1.95585400
H	-4.81286400	-4.07042200	-1.36820400
H	-5.59284100	-4.96752400	-0.05501300
H	-8.65009900	-2.63090200	1.48422000
C	-8.09434300	-3.82872800	-0.24221900

H	-7.15411500	-4.69841800	-1.99897100
H	-8.10220500	-4.77868900	0.31891300
H	-9.08221000	-3.73084500	-0.72453100

TS7(singlet)

C	-0.09423200	-2.77735600	1.25503100
C	1.36565900	-2.33054100	1.53540100
H	-0.11490300	-3.81947200	0.90521200
H	1.53776000	-2.26478900	2.62079400
N	1.39778900	-0.97729000	0.95778200
N	-0.44315700	-1.88717200	0.14023300
C	2.39910500	-0.04121000	1.29404800
C	2.10597600	1.07121200	2.11446100
C	3.69012500	-0.20943600	0.80431100
C	3.12081300	1.99368000	2.38373000
C	4.72631500	0.69558000	1.06789500
H	3.86206100	-1.06098100	0.15381300
C	4.40766400	1.80435700	1.86107600
H	2.91511100	2.87469800	2.99028300
H	5.16492900	2.55624100	2.08683100
C	0.30054500	-0.70688300	0.14427800
Fe	-0.16268000	0.68540500	-1.00749900
C	-1.59865300	-2.01176400	-0.65655900
C	-2.87450200	-1.84780200	-0.11387900
C	-1.46124900	-2.31318800	-2.03425600
C	-4.04536600	-1.98942300	-0.87450000
H	-2.93778800	-1.58780000	0.93860700
C	-2.61530600	-2.41253600	-2.81641800
C	-3.88072200	-2.26056500	-2.23689200
H	-2.54357500	-2.62334500	-3.88283600
H	-4.74964600	-2.34729900	-2.88874700
O	0.83369800	1.16941000	2.58256600
O	-0.20314900	-2.49792600	-2.49485500
C	0.52859700	2.16145400	3.54605800
H	1.11766600	2.00913100	4.46767200
H	0.70519800	3.17421100	3.15993200
H	-0.54058600	2.05462300	3.76851300
C	0.01729000	-2.60150600	-3.88576000
H	-0.42548000	-3.52478400	-4.29928700
H	-0.39157800	-1.72893400	-4.42071200
H	1.10548800	-2.62299100	-4.02487600
C	-0.42393600	0.90092300	-2.89018000
C	-1.03661500	2.16780800	-2.30417300
H	0.44224900	1.06213100	-3.54080300

H	-1.16737800	0.22264100	-3.32973900
H	-0.52828600	3.05914200	-2.68269800
C	-2.50380300	2.20010400	-2.60938000
C	-2.98411700	3.08679700	-3.58599700
C	-3.37878500	1.22662500	-2.10576900
C	-4.28420100	2.97211900	-4.08203700
H	-2.31347500	3.85005900	-3.98952700
C	-4.68412200	1.12137000	-2.58586900
H	-3.03199500	0.52031400	-1.35018800
C	-5.13820300	1.98128800	-3.58980300
H	-4.63176900	3.66118500	-4.85571600
H	-5.34478700	0.35464200	-2.18006100
H	-6.15621400	1.88896500	-3.97540600
C	0.39251200	4.20637200	0.66519700
C	-0.96863100	4.09601600	1.04991700
C	-1.46985200	4.78112000	2.15535100
C	-0.61631900	5.66631200	2.82976800
C	0.71509300	5.83555400	2.42019600
C	1.23147500	5.09769400	1.34927700
C	0.60984000	3.18933900	-0.32748300
C	-0.64230600	2.48005600	-0.56737500
H	-2.49212200	4.62103200	2.49697400
H	-0.99494500	6.22269400	3.69023400
H	1.36176800	6.53183200	2.95958600
H	2.28321700	5.18944300	1.06883500
N	-1.60324900	3.12663700	0.24677400
C	-2.88017900	2.63313100	0.66041600
C	-4.06414900	3.35900000	0.40258600
C	-2.90822000	1.46577900	1.43606100
C	-5.25328200	2.85795000	0.95764800
C	-4.10496500	0.98206800	1.95945100
H	-1.96933600	0.94913500	1.63248300
C	-5.28388500	1.69189900	1.72259400
H	-6.18707300	3.39194600	0.77594200
H	-4.10792000	0.07178700	2.56168000
H	-6.23153800	1.34079300	2.13698800
C	1.77186100	2.59263400	-0.80147300
H	2.75684100	3.06141800	-0.70530700
N	1.64467000	1.36974500	-1.31162500
C	2.68487500	0.75423900	-2.01418600
C	3.78540100	1.46505600	-2.52579400
C	2.65851600	-0.64100700	-2.21828900
C	4.85300000	0.81172400	-3.14914300
H	3.81556200	2.55248700	-2.45136600

C	3.71431600	-1.29457100	-2.83758200
H	1.79702200	-1.19761900	-1.85668700
C	4.83456000	-0.58081500	-3.29463100
H	5.68819200	1.40532600	-3.52046100
H	3.70776900	-2.37865200	-2.95829500
O	5.83679500	-1.31245400	-3.85968200
C	6.92417500	-0.63126200	-4.43614900
H	7.58656700	-1.39648000	-4.86280600
H	6.59894100	0.05389200	-5.24030700
H	7.49214200	-0.05039600	-3.68650100
H	-0.32472800	0.27541900	0.61645300
C	-4.09696000	4.60927400	-0.46982200
C	-3.98205200	5.92140100	0.32723900
C	-5.36388600	4.67247200	-1.33559500
H	-3.22697600	4.54280400	-1.13989600
H	-3.00833700	6.03716900	0.81492200
H	-4.12223900	6.77956800	-0.34953800
H	-4.76557500	5.97536200	1.10168800
H	-5.58611100	3.71015400	-1.80836000
H	-6.23986900	4.97945700	-0.74065500
H	-5.23608400	5.42032400	-2.13322000
C	6.07621100	0.48027800	0.39597400
H	5.92953200	0.77018700	-0.65932700
C	6.52948700	-1.00471500	0.38922100
C	7.23643300	1.33701400	0.95104500
H	5.72388400	-1.64137500	-0.00135500
C	6.89312400	-1.45119200	1.81429600
C	7.75386300	-1.15842000	-0.52628900
H	6.94036900	2.39831500	0.96256800
C	7.61448100	0.88357900	2.37358700
C	8.45977400	1.17947700	0.02890800
H	7.18201400	-2.51579200	1.80733200
H	6.01494200	-1.36076500	2.47413400
C	8.05299900	-0.59092100	2.34648700
H	7.48466000	-0.87124500	-1.55494900
H	8.05929300	-2.21809500	-0.56255400
C	8.90925400	-0.29123300	-0.00248300
H	6.76113300	1.00670600	3.05835600
H	8.43235000	1.51545400	2.76067400
H	8.20697200	1.51887100	-0.99033000
H	9.28339300	1.82033500	0.38747000
H	8.31839800	-0.91515500	3.36690400
C	9.27515100	-0.74428400	1.42266400
H	9.78698500	-0.39680300	-0.66260200

H	10.11702400	-0.14312400	1.80731100
H	9.61041700	-1.79564400	1.40915700
C	-5.39683800	-1.75905400	-0.21147400
H	-5.46953500	-0.66999200	-0.03668200
C	-5.51659900	-2.44151900	1.17803800
C	-6.62412200	-2.17454200	-1.05585500
H	-4.65371800	-2.17252400	1.80202000
C	-5.56461200	-3.96901800	1.01403400
C	-6.79854600	-1.95964900	1.87570700
H	-6.56819400	-1.70220700	-2.04998300
C	-6.68920000	-3.70647300	-1.20936300
C	-7.90387300	-1.68545700	-0.35295000
H	-5.61071600	-4.44679800	2.00736300
H	-4.64174500	-4.32603000	0.52850600
C	-6.79540500	-4.36232600	0.17792900
H	-6.76101500	-0.86783000	2.01777200
H	-6.86822300	-2.41340900	2.87910500
C	-8.02595000	-2.34090700	1.03360900
H	-5.79772700	-4.08503300	-1.73201700
H	-7.56299600	-3.97901700	-1.82553600
H	-7.88041900	-0.58711900	-0.25190000
H	-8.78488400	-1.93431600	-0.96849700
H	-6.83256400	-5.45877100	0.06497600
C	-8.07544200	-3.87120300	0.87750500
H	-8.94492700	-1.98759600	1.53071700
H	-8.96252600	-4.16434100	0.29027900
H	-8.17372200	-4.34787200	1.86791700
C	-1.04942200	-2.63601700	2.42327700
C	-1.10689800	-1.44333400	3.16408600
C	-1.97145400	-3.65235100	2.70252700
C	-2.08157700	-1.27301300	4.14895800
H	-0.39521900	-0.64149500	2.96010200
C	-2.94487000	-3.48361200	3.69165400
H	-1.94126400	-4.57836000	2.12220000
C	-3.00746100	-2.28929500	4.41345200
H	-2.12259000	-0.33688400	4.71131400
H	-3.66222100	-4.28325400	3.89010100
H	-3.77293500	-2.15007400	5.18050400
C	2.42829000	-3.23047100	0.93326700
C	3.57435600	-3.54799600	1.67021600
C	2.32093500	-3.68501400	-0.39064500
C	4.60721000	-4.29738700	1.09833600
H	3.66885900	-3.18589000	2.69730100
C	3.34742400	-4.44004800	-0.95886700

H	1.43999600	-3.42668900	-0.98185100
C	4.49704500	-4.74309100	-0.22009700
H	5.50071100	-4.52644200	1.68334300
H	3.25502900	-4.79049700	-1.99001400
H	5.30363500	-5.32423200	-0.67319000

TS7(triplet)

C	0.20196400	-2.99828600	-1.12256000
C	-1.25541200	-2.59389100	-1.47209300
H	0.22644800	-4.00518100	-0.68202300
H	-1.40787400	-2.63045100	-2.56119700
N	-1.29400400	-1.19554900	-1.01956200
N	0.52819200	-2.00447600	-0.08632800
C	-2.31440700	-0.28842000	-1.38950400
C	-2.03009800	0.80348900	-2.24063900
C	-3.59986600	-0.44923300	-0.88556700
C	-3.04824700	1.72140900	-2.51379300
C	-4.63862400	0.45024000	-1.15457400
H	-3.76908100	-1.28537500	-0.21608600
C	-4.32807300	1.54377400	-1.97077100
H	-2.85105300	2.59067900	-3.13989600
H	-5.08667100	2.29369500	-2.19850400
C	-0.25597900	-0.87418000	-0.16251100
Fe	0.01011900	0.72823600	0.81545000
C	1.66499900	-2.07277000	0.74733600
C	2.94676700	-1.90698200	0.22693300
C	1.49219400	-2.29158800	2.13354300
C	4.09830900	-1.96314400	1.02690600
H	3.03074900	-1.70133500	-0.83642300
C	2.62546000	-2.31832800	2.95086400
C	3.90268500	-2.16201700	2.39783700
H	2.52784400	-2.46478300	4.02615000
H	4.75475000	-2.18363200	3.07695000
O	-0.76615400	0.88765300	-2.73152600
O	0.22172300	-2.45654400	2.56889100
C	-0.44516000	1.93308200	-3.63114300
H	-1.02707000	1.84445000	-4.56537200
H	-0.61786200	2.92153100	-3.18372100
H	0.62461900	1.83087200	-3.85262800
C	-0.04396600	-2.43537600	3.95481800
H	0.38253600	-3.31691500	4.46575700
H	0.34784200	-1.51755200	4.42229300
H	-1.13667400	-2.44771900	4.06037200
C	0.40180700	0.93083300	2.73903600

C	0.93094200	2.23210600	2.16141000
H	-0.49598300	1.02675900	3.35813400
H	1.17466100	0.30522100	3.19539100
H	0.38467600	3.09555700	2.55505000
C	2.39746800	2.33095900	2.47061900
C	2.84052900	3.29027700	3.39375300
C	3.30605100	1.35461100	2.03365800
C	4.13838400	3.24515700	3.90820600
H	2.14332900	4.05590100	3.74442000
C	4.60692300	1.31750500	2.53374500
H	2.98216100	0.59439200	1.32286400
C	5.02494900	2.25084000	3.48721700
H	4.45692800	3.99029100	4.64136700
H	5.29346900	0.54517600	2.18475400
H	6.04044500	2.21158200	3.88848100
C	-0.40987800	4.26383600	-0.89024300
C	0.95824500	4.13068400	-1.24023900
C	1.48174600	4.74720100	-2.37856600
C	0.64165500	5.58428400	-3.12231400
C	-0.69936900	5.77870000	-2.74941300
C	-1.23624000	5.11160200	-1.64570100
C	-0.65754500	3.29935300	0.14628000
C	0.57723000	2.58665300	0.42582400
H	2.51026900	4.56687300	-2.68973600
H	1.03489100	6.08364200	-4.01076700
H	-1.33480100	6.43774900	-3.34586500
H	-2.29371500	5.21911800	-1.39358100
N	1.56546900	3.20081400	-0.38063400
C	2.84968000	2.67978700	-0.74197800
C	4.03130100	3.41600500	-0.50227500
C	2.88787100	1.46854700	-1.44634600
C	5.22832000	2.87876800	-1.00473300
C	4.09241400	0.94963900	-1.91461200
H	1.95097800	0.94068300	-1.62187900
C	5.26944100	1.66908000	-1.69782500
H	6.16116000	3.41803300	-0.83540200
H	4.10414900	0.00294600	-2.45834400
H	6.22388000	1.29041100	-2.07013400
C	-1.87846400	2.83253900	0.65778100
H	-2.79225300	3.43558100	0.56494400
N	-1.89725300	1.62502900	1.19024500
C	-2.98097400	1.12368200	1.90806100
C	-4.04031200	1.90927200	2.39538900
C	-3.02184900	-0.26430000	2.17072300

C	-5.12925700	1.33485900	3.05946200
H	-4.02029200	2.99251200	2.26714300
C	-4.09879400	-0.84109700	2.82642900
H	-2.19126300	-0.87885800	1.82353200
C	-5.17389100	-0.04973300	3.26669600
H	-5.93396700	1.98105400	3.40949200
H	-4.14315900	-1.91864700	2.99383200
O	-6.20456400	-0.70776400	3.86921000
C	-7.29906600	0.04131600	4.34014400
H	-8.00064800	-0.67375700	4.79001700
H	-6.99334400	0.77637800	5.10667100
H	-7.81230900	0.57724200	3.52087100
H	0.36277200	0.18038300	-0.68544000
C	4.05641800	4.70954800	0.30657100
C	3.91188600	5.98239300	-0.54760300
C	5.33233900	4.83682400	1.15248200
H	3.19628100	4.66187800	0.99087500
H	2.92697100	6.06954800	-1.01789900
H	4.05997900	6.87147000	0.08618800
H	4.67727600	6.00721200	-1.34141300
H	5.57893000	3.90292400	1.66833100
H	6.19506400	5.13282100	0.53302300
H	5.19813000	5.61924400	1.91516400
C	-5.97368300	0.25420000	-0.44959600
H	-5.80063300	0.56168400	0.59509800
C	-6.43849100	-1.22604700	-0.40812000
C	-7.13957600	1.11272400	-0.98826900
H	-5.62779500	-1.86272500	-0.02855200
C	-6.84120600	-1.69160600	-1.81619300
C	-7.63978400	-1.35607700	0.54191400
H	-6.83411300	2.17084000	-1.02306400
C	-7.55825500	0.64063200	-2.39315100
C	-8.33869300	0.98021800	-0.03113500
H	-7.13717700	-2.75414300	-1.78627500
H	-5.97966500	-1.61710700	-2.49980600
C	-8.00794600	-0.82964000	-2.33141600
H	-7.34353900	-1.06118700	1.56045700
H	-7.95514700	-2.41235600	0.59621500
C	-8.80017200	-0.48567600	0.03431500
H	-6.72190900	0.74574500	-3.10165600
H	-8.38079300	1.27313200	-2.76922400
H	-8.05307700	1.33202200	0.97508400
H	-9.16606300	1.62372600	-0.37621200
H	-8.30287600	-1.16724500	-3.33933100

C	-9.20706900	-0.95678700	-1.37375700
H	-9.66149400	-0.57291000	0.71844600
H	-10.05292100	-0.35368000	-1.74660600
H	-9.55162200	-2.00452400	-1.33495100
C	5.45863600	-1.71321000	0.38925400
H	5.49523900	-0.63123300	0.16527300
C	5.64564100	-2.45290400	-0.96315000
C	6.67513500	-2.04031100	1.28613200
H	4.79231400	-2.24634900	-1.62365800
C	5.74701900	-3.96827100	-0.72676900
C	6.92905200	-1.95314300	-1.64579400
H	6.57102700	-1.52497000	2.25457800
C	6.79257600	-3.55980200	1.51342100
C	7.95600500	-1.53428400	0.59774700
H	5.84182300	-4.48860500	-1.69498900
H	4.82389500	-4.33888400	-0.25221800
C	6.96577200	-4.27398300	0.16203800
H	6.85429900	-0.87106100	-1.83948800
H	7.04633300	-2.44873800	-2.62469900
C	8.14428900	-2.24711700	-0.75247200
H	5.89971600	-3.94739000	2.02706200
H	7.65644200	-3.77015900	2.16688000
H	7.89421900	-0.44342400	0.44481700
H	8.82696900	-1.72011100	1.24886300
H	7.04109000	-5.36182400	0.32734900
C	8.24687400	-3.76557500	-0.52318300
H	9.06371300	-1.88137700	-1.23972000
H	9.12627900	-3.99625100	0.10223000
H	8.39302600	-4.28306100	-1.48687700
C	-2.33425500	-3.41947100	-0.79695400
C	-3.43202600	-3.87746400	-1.53265200
C	-2.30319200	-3.63248600	0.59109400
C	-4.49595400	-4.52679100	-0.89796300
H	-3.46841600	-3.69917300	-2.61060100
C	-3.36329000	-4.28295600	1.22350100
H	-1.45729500	-3.26698500	1.17747400
C	-4.46645800	-4.72576500	0.48346400
H	-5.35403700	-4.86446100	-1.48354900
H	-3.33362000	-4.44078500	2.30463200
H	-5.30077300	-5.22174400	0.98491800
C	1.18616200	-2.93239900	-2.27315700
C	1.26152300	-1.78586000	-3.08295800
C	2.11894200	-3.95905000	-2.46040700
C	2.26556800	-1.67136900	-4.04591200

H	0.54416200	-0.97372500	-2.94851000
C	3.12218700	-3.84566100	-3.42769000
H	2.07516000	-4.84669600	-1.82384300
C	3.20279300	-2.69734800	-4.21847300
H	2.32101200	-0.77119800	-4.66306000
H	3.84917100	-4.65121200	-3.55360200
H	3.99217600	-2.60048100	-4.96757600

int8(singlet)

C	2.32473200	-3.10090200	1.58403800
C	3.20108600	-1.82319000	1.77442600
H	2.89423400	-3.85875800	1.03763900
H	3.54636100	-1.74292400	2.81510000
N	2.21195600	-0.75444100	1.50705600
N	1.25326900	-2.56300700	0.74303300
C	4.40263100	-1.90600200	0.85192900
C	5.68705900	-2.06366700	1.38791300
C	4.23021100	-1.97941000	-0.53586400
C	6.78372700	-2.30063600	0.55378800
H	5.82818600	-2.00677100	2.47095900
C	5.31925600	-2.23688500	-1.36854700
H	3.23543400	-1.86648500	-0.96059300
C	6.59927300	-2.39865100	-0.82748600
H	7.78060300	-2.41703000	0.98569800
H	5.15644800	-2.31954500	-2.44385800
H	7.45140500	-2.59748100	-1.48208000
C	1.82094900	-3.69840100	2.88214900
C	2.56238700	-4.72023500	3.49101700
C	0.65549300	-3.23137000	3.50551800
C	2.15618000	-5.26372900	4.71181400
H	3.46501900	-5.09565900	2.99990800
C	0.24830500	-3.77978400	4.72446700
H	0.05989200	-2.44437400	3.03380600
C	0.99549500	-4.79286600	5.33245600
H	2.74126000	-6.06237300	5.17479400
H	-0.66470500	-3.41253400	5.19989600
H	0.67086100	-5.22015600	6.28449800
C	2.49988000	0.61937600	1.55296800
C	1.47057300	1.58333800	1.64278400
C	3.81960200	1.08237700	1.43525100
C	1.78261100	2.94037800	1.57916500
C	4.14794700	2.43943300	1.34074700
H	4.60719800	0.34258500	1.37134000
C	3.10008000	3.36752900	1.42156200

H	0.97875400	3.67253400	1.64056000
H	3.29803500	4.43874500	1.36568400
C	1.05917000	-1.22209900	0.92056100
Fe	-0.59534200	-0.36471900	0.69878700
C	0.38931100	-3.29556200	-0.09199000
C	-0.97405500	-3.00026700	-0.09289400
C	0.90930500	-4.18398000	-1.06217900
C	-1.87022600	-3.57166600	-1.01559300
H	-1.33872800	-2.33272100	0.70582300
C	0.03975700	-4.73033900	-2.00578800
C	-1.32799300	-4.41977300	-1.98522700
H	0.42158200	-5.38985000	-2.78491900
H	-1.96332900	-4.85915600	-2.75252500
O	0.16747900	1.17438100	1.80209000
O	2.25397200	-4.38486900	-1.04597100
C	-0.64857700	1.88659900	2.73247900
H	-0.02860200	2.23492600	3.57163800
H	-1.13764900	2.74181000	2.24465900
H	-1.40196000	1.17347400	3.08120400
C	2.86611400	-5.08379700	-2.10485600
H	2.53666300	-6.13702800	-2.14583200
H	2.66081400	-4.60298900	-3.07685000
H	3.94632300	-5.04834300	-1.91236000
C	5.58870700	2.90678500	1.15916300
H	5.90275400	3.34023600	2.12735600
C	5.73154000	4.02907700	0.09438700
C	6.60301900	1.78968900	0.80771500
H	5.01986100	4.83931800	0.31196700
C	5.45033800	3.46526200	-1.30965700
C	7.15835400	4.60199700	0.14207500
H	6.52886300	0.97401200	1.54356700
C	6.34380600	1.23320800	-0.60539200
C	8.02905200	2.36773000	0.85736500
H	5.51042400	4.27628600	-2.05512800
H	4.42621800	3.06130200	-1.35595900
C	6.47190000	2.36276000	-1.63909100
H	7.36026100	5.02786800	1.13959300
H	7.25261600	5.42656600	-0.58493800
C	8.17618100	3.49590100	-0.17771300
H	5.34942800	0.77152700	-0.67102300
H	7.06938900	0.43343500	-0.82123000
H	8.24868800	2.75161000	1.86813700
H	8.75887900	1.56648400	0.65157200
H	6.27417200	1.96413900	-2.64964100

C	7.89601000	2.94361700	-1.58706400
H	9.19871000	3.90710900	-0.13656100
H	8.63122400	2.16057300	-1.83987200
H	8.00901000	3.74632200	-2.33591800
C	-3.34741500	-3.23536400	-0.89318800
H	-3.42183400	-2.14035700	-1.00782500
C	-3.90761500	-3.57005400	0.51945800
C	-4.29281900	-3.86296000	-1.94360900
H	-3.23312700	-3.15322700	1.28242100
C	-4.02009900	-5.09218400	0.70278200
C	-5.29539800	-2.93004900	0.67156100
H	-3.90444900	-3.65639800	-2.95518700
C	-4.45086300	-5.38472100	-1.74290700
C	-5.67926000	-3.20540300	-1.79197400
H	-4.37392500	-5.31529600	1.72365000
H	-3.02940600	-5.56436400	0.59544200
C	-4.99803900	-5.67168900	-0.33593500
H	-5.21652700	-1.84024300	0.56726600
H	-5.69064700	-3.13293500	1.68201900
C	-6.24971300	-3.49439400	-0.39131000
H	-3.49511700	-5.91142900	-1.87574800
H	-5.14201100	-5.78117800	-2.50666300
H	-5.60372100	-2.11833400	-1.94742600
H	-6.36321900	-3.59669400	-2.56478500
H	-5.08841500	-6.76168100	-0.19166300
C	-6.38106600	-5.01325200	-0.18585800
H	-7.23719400	-3.01281700	-0.29454900
H	-7.08200100	-5.44119900	-0.92345700
H	-6.79667500	-5.22445900	0.81456600
C	-1.15047000	4.18156900	-0.19901800
C	-2.36631500	4.54211300	0.43597700
C	-2.55982700	5.79369400	1.02764200
C	-1.51698500	6.71608900	0.93948600
C	-0.31140700	6.39009700	0.28513700
C	-0.11520000	5.12946200	-0.27621500
C	-1.28436600	2.79147300	-0.57465700
H	-3.49187000	6.03326800	1.54142600
H	-1.63582900	7.70480200	1.38832500
H	0.48511000	7.13578100	0.22854200
H	0.83446900	4.87747900	-0.75304600
N	-3.22342900	3.44108800	0.37945100
C	-4.52791100	3.46934600	0.96586600
C	-5.63400700	3.95393600	0.24199800
C	-4.65051900	3.05086200	2.29479100

C	-6.86766900	3.98455700	0.90952300
C	-5.88874600	3.08623100	2.93262500
H	-3.76200400	2.68419800	2.81017300
C	-7.00110700	3.55649200	2.23093400
H	-7.75051800	4.34087600	0.37702800
H	-5.98383000	2.74828900	3.96673000
H	-7.98055300	3.58957500	2.71415800
C	-0.25727900	1.99993800	-1.18583100
H	0.43846200	2.55625000	-1.83315900
N	-0.07134000	0.71973300	-1.03428000
C	0.98263600	0.16614000	-1.81405500
C	0.77826600	-1.03454400	-2.51257500
C	2.22473700	0.80595500	-1.92979100
C	1.76686500	-1.54102100	-3.35028300
H	-0.17263900	-1.55740900	-2.40693400
C	3.21814100	0.30784900	-2.77486700
H	2.42960300	1.69726200	-1.33498400
C	2.98756500	-0.86493400	-3.50321700
H	1.60291300	-2.46303200	-3.91008800
H	4.17316700	0.82686100	-2.83079000
O	3.90401000	-1.43277300	-4.34174600
C	5.03555200	-0.67178700	-4.70435300
H	5.57793900	-1.25456400	-5.46086600
H	4.74513000	0.30286100	-5.13511300
H	5.70564200	-0.49141200	-3.84477600
H	-0.91950500	-1.02693100	2.04548600
C	-5.50746500	4.41732400	-1.20141000
C	-5.23358200	5.92944800	-1.28687300
C	-6.73357800	4.05790300	-2.04920400
H	-4.63955900	3.89195800	-1.62896000
H	-4.30474600	6.21209300	-0.77561900
H	-5.14811200	6.24158900	-2.34009100
H	-6.06228200	6.49735600	-0.83233700
H	-7.00828300	3.00207500	-1.93586400
H	-7.60610500	4.67606700	-1.78194400
H	-6.52033200	4.24458200	-3.11331300
C	-2.57377700	2.37722400	-0.22503100
C	-3.09122200	0.98810000	-0.42325800
H	-2.55150600	0.63528500	-1.31620100
C	-4.56623400	0.84443800	-0.78856900
C	-5.56680700	0.62050500	0.16720400
C	-4.93410300	0.81289000	-2.14332800
C	-6.88853100	0.38504700	-0.21406400
H	-5.30083400	0.61489500	1.22318100

C	-6.25108700	0.55755200	-2.53384600
H	-4.16415300	0.96823700	-2.90474900
C	-7.23715700	0.34265900	-1.56723400
H	-7.64813500	0.21628600	0.55298000
H	-6.50652100	0.52212500	-3.59582700
H	-8.26803700	0.13846100	-1.86607200
C	-2.58977900	0.11027400	0.75614300
H	-2.93711800	0.56324800	1.70253300
H	-3.14744900	-0.84123800	0.69535200

int8(triplet)

C	-2.12787200	-3.41406000	-1.40897300
C	-3.11831400	-2.24183000	-1.64198100
H	-2.60377200	-4.18920100	-0.80120500
H	-3.49469500	-2.25269100	-2.67382400
N	-2.21580000	-1.07650200	-1.46895700
N	-1.07496700	-2.73484900	-0.63357400
C	-4.28259200	-2.29785600	-0.67401900
C	-5.57008000	-2.57901700	-1.14540500
C	-4.07933800	-2.12655800	0.70260000
C	-6.64501800	-2.68696700	-0.25703800
H	-5.73488100	-2.70378700	-2.21914800
C	-5.14692600	-2.25010700	1.59153300
H	-3.08609200	-1.89346200	1.08311900
C	-6.43339200	-2.52739000	1.11436900
H	-7.64734000	-2.89603800	-0.63853400
H	-4.96364700	-2.12961000	2.65989200
H	-7.27003700	-2.61488700	1.81186700
C	-1.58834600	-4.02165400	-2.68683000
C	-2.18358000	-5.18333800	-3.19566300
C	-0.53451700	-3.42530800	-3.39462400
C	-1.74357000	-5.74038000	-4.39880900
H	-2.99778500	-5.65768900	-2.64027300
C	-0.09351800	-3.98507700	-4.59582900
H	-0.04859400	-2.52848000	-3.00254700
C	-0.69578900	-5.14059100	-5.10264300
H	-2.21366100	-6.64917800	-4.78264600
H	0.73204200	-3.51600900	-5.13675300
H	-0.34443400	-5.57717200	-6.04073100
C	-2.62864200	0.24774300	-1.77978700
C	-1.77647200	1.14586100	-2.45904600
C	-3.89807900	0.68941100	-1.39566300
C	-2.23379700	2.43477100	-2.74801700
C	-4.36297800	1.98410200	-1.65014500

H	-4.52863300	-0.00681700	-0.85905400
C	-3.50741300	2.84387600	-2.35038000
H	-1.58424400	3.13913700	-3.26430400
H	-3.82378700	3.86087500	-2.59014900
C	-1.05052800	-1.39990500	-0.85253900
Fe	0.52594800	-0.16728900	-0.72193200
C	-0.15482900	-3.42254100	0.19925100
C	1.21253400	-3.18826800	0.09515200
C	-0.64139300	-4.24677800	1.24061000
C	2.13940700	-3.69105800	1.02318000
H	1.53777900	-2.53624000	-0.71180500
C	0.27053400	-4.79547800	2.14433000
C	1.63761000	-4.50347500	2.04462000
H	-0.07914800	-5.42645200	2.96102500
H	2.30448200	-4.91745200	2.80061700
O	-0.52773500	0.70469900	-2.76048600
O	-1.98882500	-4.40512400	1.31838800
C	0.34724100	1.53237600	-3.50962600
H	-0.09790800	1.78874900	-4.48524800
H	0.59566200	2.45140600	-2.95565000
H	1.25794600	0.94110100	-3.65767100
C	-2.54983400	-5.11714900	2.39699600
H	-2.24414200	-6.17820900	2.38848100
H	-2.27025100	-4.67103800	3.36667500
H	-3.63872800	-5.04834700	2.27606200
C	-5.74530600	2.44582000	-1.20716700
H	-6.33879800	2.57841800	-2.13118100
C	-5.72315500	3.82109900	-0.48627200
C	-6.52214200	1.44905200	-0.31005700
H	-5.16242300	4.54756400	-1.09415300
C	-5.05390400	3.68487300	0.89249800
C	-7.16609500	4.32557600	-0.31389300
H	-6.54771700	0.45802200	-0.78932900
C	-5.87719500	1.33059900	1.08556700
C	-7.96657300	1.95591000	-0.14216200
H	-4.99665800	4.67371100	1.37790200
H	-4.01941500	3.32179900	0.77594000
C	-5.85977500	2.70750600	1.76642200
H	-7.64333400	4.44584300	-1.30134100
H	-7.15956900	5.32001400	0.16389000
C	-7.96622200	3.33276900	0.54492800
H	-4.85576500	0.93234400	1.02303700
H	-6.45237400	0.60854700	1.68892900
H	-8.46138600	2.02457500	-1.12589700

H	-8.54405500	1.23291900	0.45850000
H	-5.38532000	2.62529600	2.75912600
C	-7.30205400	3.21815800	1.92847000
H	-9.00315400	3.69087500	0.65867600
H	-7.87617300	2.52623400	2.56843900
H	-7.30559300	4.20040900	2.43142300
C	3.59224500	-3.24212000	0.92534900
H	3.58425800	-2.16907500	1.19178900
C	4.17999100	-3.33415200	-0.50868100
C	4.57578100	-3.93802600	1.89439000
H	3.49132400	-2.85527900	-1.22011700
C	4.38621700	-4.80539300	-0.90362400
C	5.52610500	-2.59130800	-0.54608900
H	4.17759300	-3.88903500	2.92102500
C	4.80393200	-5.40730900	1.48917300
C	5.92306400	-3.19243800	1.85188600
H	4.76759300	-4.86172600	-1.93737600
H	3.42330500	-5.34233300	-0.88579000
C	5.38165900	-5.46822700	0.06516300
H	5.38284000	-1.53234300	-0.28637000
H	5.93590600	-2.61668300	-1.57032500
C	6.51119300	-3.24993100	0.43187000
H	3.86338000	-5.97793400	1.53051200
H	5.49947800	-5.88227300	2.20235800
H	5.78428900	-2.14235600	2.15487500
H	6.62359500	-3.65037500	2.57137200
H	5.53585200	-6.52138000	-0.22472200
C	6.72577100	-4.71836700	0.02459100
H	7.47096900	-2.70742200	0.40929500
H	7.44703600	-5.20190500	0.70611100
H	7.15722000	-4.76978500	-0.98994800
C	0.71530900	4.02349100	-0.65487600
C	1.97323900	4.63756700	-0.89696200
C	2.08551300	5.94630000	-1.37738200
C	0.90474000	6.64066100	-1.64059200
C	-0.35474700	6.04799900	-1.41343000
C	-0.45978300	4.75067000	-0.91630800
C	0.99001700	2.70486700	-0.13208900
H	3.06247900	6.40459900	-1.53712200
H	0.95818300	7.66251000	-2.02337700
H	-1.26162000	6.62101600	-1.62185600
H	-1.43915400	4.30698600	-0.73067500
N	2.97056400	3.73043100	-0.53429800
C	4.35233400	4.08950300	-0.53036800

C	5.04125100	4.30192000	0.68202200
C	4.98434100	4.26035000	-1.76520800
C	6.40122200	4.62889100	0.59146200
C	6.33159000	4.61234800	-1.82693300
H	4.40536800	4.08962600	-2.67483500
C	7.04369800	4.78121600	-0.63932400
H	6.97665000	4.77378700	1.50577700
H	6.82347300	4.73590700	-2.79428900
H	8.10554700	5.03689900	-0.66648800
C	-0.02264400	1.72994900	0.22544300
H	-1.05353200	2.06167500	0.02505800
N	0.16875500	0.68523300	1.05469500
C	-0.85694600	0.24020600	1.88889600
C	-0.74040400	-1.03813900	2.47956000
C	-1.96646600	1.02501600	2.25119700
C	-1.70325700	-1.51729900	3.35481200
H	0.13250400	-1.64413600	2.23850700
C	-2.93908800	0.54756800	3.13631700
H	-2.06770100	2.03884300	1.86273700
C	-2.82004300	-0.73151200	3.68701700
H	-1.60475200	-2.50510600	3.80699500
H	-3.77985200	1.19139500	3.38867600
O	-3.73253100	-1.29191900	4.53939200
C	-4.80023500	-0.49111300	4.98951500
H	-5.38183100	-1.10430500	5.69104300
H	-4.43941600	0.41229900	5.51332500
H	-5.45762200	-0.17585700	4.15841400
H	0.98328500	-1.10375000	-1.86286700
C	4.31894000	4.22495700	2.01953300
C	3.44243400	5.47346100	2.23168400
C	5.25281800	4.01881200	3.21449200
H	3.64427000	3.35680200	1.97844000
H	2.71031200	5.60556700	1.42392300
H	2.88839000	5.39489000	3.18065800
H	4.06796000	6.38007300	2.27548500
H	5.91928500	3.15783100	3.06386300
H	5.87422100	4.90911500	3.40431700
H	4.65973400	3.83847200	4.12436000
C	2.37713700	2.55446000	-0.08188900
C	3.02269500	1.22842700	0.21714600
H	2.59266600	0.91677100	1.18145200
C	4.53095700	1.14847700	0.36400300
C	5.39726000	1.23911800	-0.73694600
C	5.08890900	0.85547100	1.61558200

C	6.77375700	1.08672100	-0.58454000
H	4.98450600	1.42969100	-1.72758700
C	6.46801200	0.68445500	1.77393700
H	4.42741600	0.75547700	2.48020400
C	7.31812500	0.80713200	0.67398500
H	7.42753300	1.17976700	-1.45487700
H	6.87654700	0.45134000	2.76045400
H	8.39653200	0.67789500	0.79219300
C	2.51658900	0.19842900	-0.84073000
H	2.84041600	0.52774500	-1.84276400
H	3.04945300	-0.74789200	-0.65926500

int8(quinet)

C	-1.69903600	-3.39346300	-1.26095900
C	-2.75035800	-2.29605700	-1.58691600
H	-2.16667800	-4.20398900	-0.69121600
H	-2.99050700	-2.29868400	-2.65902600
N	-1.97675700	-1.06400000	-1.27318600
N	-0.76459800	-2.64390700	-0.40323000
C	-4.01967900	-2.47493400	-0.78111500
C	-5.20613400	-2.85641600	-1.41847600
C	-4.01027000	-2.30607400	0.60970000
C	-6.37390600	-3.06207200	-0.67679900
H	-5.22036400	-2.97996100	-2.50491400
C	-5.17203700	-2.51839100	1.35226000
H	-3.09782900	-1.99594000	1.11632700
C	-6.35738200	-2.89509300	0.71014400
H	-7.29737600	-3.34933600	-1.18526400
H	-5.14216200	-2.38067800	2.43443400
H	-7.26936700	-3.05287300	1.29106700
C	-0.98592300	-3.95273300	-2.47453800
C	-1.37449800	-5.19173300	-2.99763300
C	0.04757400	-3.23932700	-3.10096300
C	-0.74814900	-5.71270700	-4.13310300
H	-2.17153400	-5.75647200	-2.50602500
C	0.67621000	-3.76194900	-4.23264900
H	0.37358000	-2.27815200	-2.69702600
C	0.28055500	-4.99861600	-4.75245200
H	-1.05772400	-6.68317400	-4.52845400
H	1.48567000	-3.20210800	-4.70719200
H	0.77835800	-5.40763600	-5.63485300
C	-2.44942300	0.23406900	-1.59771900
C	-1.57212000	1.23954600	-2.04564300
C	-3.80903400	0.53769700	-1.47535800

C	-2.08001600	2.48923300	-2.40037400
C	-4.33345500	1.79607200	-1.78549900
H	-4.46721400	-0.23771600	-1.10697400
C	-3.44190400	2.76147900	-2.27389300
H	-1.40335200	3.26568100	-2.75207200
H	-3.80161500	3.75365000	-2.55233900
C	-0.85153600	-1.31613500	-0.57388800
Fe	0.61327200	0.07985300	-0.11984100
C	0.20195300	-3.29137700	0.40999500
C	1.56087500	-3.07511600	0.21168300
C	-0.23760700	-4.11584200	1.46636500
C	2.53662100	-3.61669200	1.05902000
H	1.83493200	-2.41828600	-0.60760500
C	0.72232600	-4.69606300	2.30140700
C	2.08313300	-4.43627000	2.10059900
H	0.41825500	-5.33548400	3.12976600
H	2.79630200	-4.88583000	2.79301700
O	-0.23582500	0.94492900	-2.07714800
O	-1.57949600	-4.26598400	1.59747100
C	0.61037200	1.65737400	-2.97779500
H	0.15961400	1.67389600	-3.98229100
H	0.79247800	2.68208800	-2.62719700
H	1.55532500	1.10303300	-2.99897800
C	-2.09523600	-5.01454800	2.67554000
H	-1.80082700	-6.07685500	2.61017400
H	-1.76128700	-4.60972000	3.64625900
H	-3.18774600	-4.93153300	2.61105800
C	-5.81661300	2.10586700	-1.62375900
H	-6.23024700	2.18905200	-2.64651200
C	-6.07955600	3.46413800	-0.91879600
C	-6.63994600	1.01904200	-0.88656100
H	-5.49703500	4.25639100	-1.41334400
C	-5.67083000	3.37724800	0.56202000
C	-7.57476700	3.80975400	-1.02409600
H	-6.47248800	0.03893300	-1.35945500
C	-6.25675800	0.94565400	0.60539500
C	-8.13570100	1.36826300	-0.99576700
H	-5.81345100	4.35864000	1.04523300
H	-4.60098000	3.12692600	0.64423000
C	-6.51617300	2.30526300	1.27218200
H	-7.86871000	3.89345400	-2.08432600
H	-7.76370400	4.79224800	-0.55908300
C	-8.41117000	2.72545100	-0.32572500
H	-5.20380300	0.66227600	0.73870900

H	-6.85276600	0.15687900	1.09369200
H	-8.44164500	1.40084800	-2.05544400
H	-8.73472700	0.57821900	-0.51204300
H	-6.22866100	2.25535100	2.33560800
C	-8.00931400	2.65808400	1.15831000
H	-9.48322300	2.97046400	-0.41051000
H	-8.61635200	1.89888500	1.68083900
H	-8.21205900	3.62596100	1.64826500
C	4.00759100	-3.24840700	0.89556100
H	4.19417800	-2.42757100	1.61330700
C	4.37854800	-2.70598000	-0.50888400
C	4.98779100	-4.39779900	1.24131800
H	3.70576900	-1.87933800	-0.77745800
C	4.27867300	-3.82551900	-1.56110300
C	5.81929900	-2.16719500	-0.47830900
H	4.75287600	-4.80198600	2.23789400
C	4.87776100	-5.51678000	0.19040300
C	6.42647900	-3.85311500	1.26514100
H	4.52669000	-3.41945700	-2.55685300
H	3.24887700	-4.21089700	-1.62338300
C	5.24830300	-4.96335000	-1.19736400
H	5.89893200	-1.34413900	0.25114600
H	6.07666200	-1.74525800	-1.46421500
C	6.79694500	-3.29675700	-0.11855900
H	3.85381800	-5.92402600	0.17464500
H	5.55201800	-6.34783500	0.45913800
H	6.51720300	-3.06203800	2.02858400
H	7.12432400	-4.65883800	1.55021500
H	5.17240600	-5.76813700	-1.94771800
C	6.68839900	-4.41990100	-1.16578300
H	7.82777100	-2.90493200	-0.09830500
H	7.39509500	-5.23218000	-0.92342700
H	6.96900400	-4.03619600	-2.16175500
C	0.68709600	4.22448700	-0.60103400
C	2.03822200	4.39104500	-1.00912200
C	2.40103500	5.32057600	-1.98922900
C	1.40070300	6.14106600	-2.51366300
C	0.06611300	6.03025700	-2.07866500
C	-0.29692300	5.07013600	-1.13232900
C	0.64493700	3.07920400	0.29718200
H	3.43101900	5.39478700	-2.33700500
H	1.66095400	6.88217800	-3.27320700
H	-0.69339000	6.69567100	-2.49598500
H	-1.33778600	4.96649400	-0.81929700

N	2.81987300	3.45346800	-0.33827000
C	4.12686900	3.08000600	-0.76403500
C	5.23675900	3.92387500	-0.56303100
C	4.27256100	1.84193100	-1.40915200
C	6.48751300	3.45976300	-1.00132900
C	5.52458400	1.40531200	-1.83149000
H	3.38708800	1.22231300	-1.55323500
C	6.64176400	2.21901700	-1.61915800
H	7.36796300	4.08635600	-0.84171200
H	5.62698400	0.43631200	-2.32402100
H	7.63281000	1.88927800	-1.93987900
C	-0.52152900	2.44555100	0.79627900
H	-1.47809600	2.97319700	0.72094900
N	-0.47569900	1.14048000	1.21467600
C	-1.46017400	0.59490500	2.01882400
C	-1.26414500	-0.70503700	2.55354400
C	-2.65454000	1.25673400	2.38239200
C	-2.20761400	-1.30729800	3.37100700
H	-0.33341400	-1.22853100	2.32849600
C	-3.61642500	0.64215300	3.19193200
H	-2.84473200	2.27165500	2.03618100
C	-3.40883200	-0.64842200	3.68437700
H	-2.03896500	-2.30381700	3.78078500
H	-4.52805000	1.19109000	3.42549200
O	-4.31196000	-1.34776700	4.44740900
C	-5.49548400	-0.69349000	4.83145300
H	-6.06293600	-1.39968300	5.45316500
H	-5.28795100	0.21779000	5.42182700
H	-6.11220300	-0.40944300	3.95785700
H	1.55482300	-0.57468800	-1.18496700
C	5.12391300	5.26756200	0.14076300
C	5.68308800	6.41685800	-0.71106000
C	5.80546000	5.22683700	1.51554800
H	4.05610200	5.46283100	0.31091100
H	5.20979500	6.46253100	-1.70306600
H	5.51006700	7.38232300	-0.20982000
H	6.76981700	6.31433800	-0.86270000
H	5.37429200	4.44345400	2.14884700
H	6.88513200	5.02675100	1.42056200
H	5.68596700	6.19314400	2.03206300
C	1.98638000	2.66703100	0.47743900
C	2.44443500	1.61205000	1.46375500
H	1.64669300	1.63831100	2.21837100
C	3.72634700	1.92482400	2.23232700

C	4.98041300	1.40726900	1.87830300
C	3.63864000	2.69795000	3.40129700
C	6.10703000	1.64698600	2.66905500
H	5.08309100	0.80688000	0.97554900
C	4.75911300	2.93233600	4.20152800
H	2.66738200	3.10736900	3.69272200
C	6.00117500	2.40278200	3.83915000
H	7.07340800	1.23588100	2.36637000
H	4.66133700	3.52858800	5.11227600
H	6.88072200	2.58318400	4.46193200
C	2.37875400	0.20545000	0.86226900
H	3.26351900	-0.04887800	0.27333300
H	2.25862200	-0.55635500	1.65278700

TS9(singlet)

C	-0.82207200	-0.75831200	2.54363900
C	0.59490600	-0.17833000	2.59164800
H	-0.82871000	-1.79828300	2.89921900
H	0.64524500	0.68842500	3.26468700
N	0.77938800	0.28789700	1.19940800
N	-1.10216000	-0.77057700	1.07781400
C	1.67693200	-1.15921300	2.99315500
C	2.58882600	-0.83746300	4.00262100
C	1.81738400	-2.37547100	2.30840800
C	3.62682900	-1.71506100	4.33021600
H	2.49459900	0.11730000	4.52667200
C	2.85857200	-3.24738300	2.62615700
H	1.11272500	-2.62647800	1.51525600
C	3.76660200	-2.92009300	3.63943600
H	4.33742600	-1.44857800	5.11588200
H	2.96817600	-4.18543500	2.07633700
H	4.58514400	-3.60095700	3.88422200
C	-1.87567600	0.01813300	3.30357300
C	-2.94289100	-0.66515100	3.90142700
C	-1.85595200	1.41946200	3.34990800
C	-3.98226800	0.03420700	4.51951200
H	-2.96811800	-1.75658600	3.86158800
C	-2.89060600	2.12058000	3.97290900
H	-1.03394400	1.96672500	2.88485900
C	-3.96040400	1.43128100	4.55331400
H	-4.81124000	-0.51327600	4.97438900
H	-2.86658700	3.21263300	3.99525400
H	-4.77207400	1.98193900	5.03468000
C	1.96968000	1.02971000	0.98465800

C	1.98419200	2.41403500	1.24494900
C	3.15287100	0.37197800	0.67943100
C	3.19395800	3.10489100	1.12940300
C	4.37164800	1.04548100	0.52817500
H	3.09875900	-0.69937100	0.51356700
C	4.36081200	2.42652500	0.75594100
H	3.23330600	4.18081100	1.28914300
H	5.27372300	3.01165400	0.64068400
C	-0.17143100	-0.13178700	0.31128900
Fe	-0.19661700	0.33658200	-1.60007500
C	-2.18692700	-1.61234300	0.72125600
C	-3.49279700	-1.15289300	0.87047700
C	-1.94857900	-2.98177500	0.49018800
C	-4.60261800	-2.00962000	0.82983900
H	-3.61514200	-0.08990500	1.05905300
C	-3.03899100	-3.85322300	0.43880400
C	-4.33775700	-3.36820800	0.61684200
H	-2.89094000	-4.91413400	0.24445300
H	-5.15706100	-4.08662900	0.57837800
O	0.80049900	2.98166800	1.59614900
O	-0.65091500	-3.36160200	0.36474700
C	0.77682500	4.35521500	1.93556500
H	1.38475800	4.55224100	2.83643000
H	1.13060300	4.98576700	1.10699700
H	-0.27327300	4.60186400	2.13965000
C	-0.35774400	-4.72137800	0.11971600
H	-0.81177800	-5.06045100	-0.82457800
H	0.73457700	-4.79121100	0.03768400
H	-0.69954300	-5.36420400	0.94967900
C	-1.66527200	0.56882400	-3.06759600
C	-2.19153100	-0.28648400	-2.06506700
H	-2.07933600	1.57658200	-3.15896600
H	-1.35304600	0.13475200	-4.02559900
H	-3.00245300	0.08841800	-1.43846800
C	-2.22428700	-1.72878300	-2.41043400
C	-3.43103600	-2.44572200	-2.45848100
C	-1.05334500	-2.40232200	-2.80865200
C	-3.46579700	-3.77922100	-2.86623200
H	-4.35417800	-1.94361600	-2.17186500
C	-1.07993000	-3.73498100	-3.21622500
H	-0.09643400	-1.87242000	-2.79955200
C	-2.29135500	-4.43634400	-3.24484900
H	-4.42149400	-4.31010200	-2.88751400
H	-0.14800900	-4.22558600	-3.50927000

H	-2.31925000	-5.48026900	-3.56694500
C	1.56937200	4.04085400	-1.68515300
C	0.23289900	4.48390400	-1.44388400
C	-0.06746500	5.82282500	-1.19637500
C	0.98781100	6.74434500	-1.20125400
C	2.30806400	6.32937600	-1.44230000
C	2.60589600	4.98353000	-1.68039800
C	1.49496200	2.60763300	-1.81400000
C	0.13618400	2.20486300	-1.61675800
H	-1.09019500	6.13677200	-0.98036500
H	0.77882500	7.79926400	-1.00825800
H	3.11316400	7.06812700	-1.43739200
H	3.63796100	4.66479400	-1.84220600
N	-0.61172800	3.36796500	-1.43380700
C	-1.98178000	3.38533500	-1.06164100
C	-2.94343400	3.97671100	-1.90478100
C	-2.35291600	2.78294900	0.14580800
C	-4.28158900	3.95848500	-1.48080600
C	-3.68807200	2.77709400	0.54486900
H	-1.57208600	2.33202000	0.75437800
C	-4.65559000	3.37032700	-0.27112200
H	-5.04412900	4.40438400	-2.12492400
H	-3.96553000	2.32291200	1.49697900
H	-5.70478100	3.36936400	0.03321200
C	2.35308900	1.52952100	-2.04660100
H	3.42721000	1.63705700	-2.22289600
N	1.77697700	0.32943800	-1.98022200
C	2.53612300	-0.83162500	-2.18652800
C	3.66403200	-0.87637800	-3.02314600
C	2.18541900	-2.01367600	-1.49838400
C	4.45569500	-2.02432200	-3.12291100
H	3.93165200	0.00090100	-3.61402200
C	2.96708000	-3.15695800	-1.59363800
H	1.30339100	-2.00944500	-0.85873300
C	4.12302700	-3.17209700	-2.39106800
H	5.32680500	-2.00854100	-3.77754300
H	2.71267500	-4.06178100	-1.03877000
O	4.84552000	-4.32500800	-2.38935400
C	6.01405000	-4.39168500	-3.17090900
H	6.44059200	-5.39177500	-3.01631500
H	5.79818600	-4.25649400	-4.24646200
H	6.75777200	-3.63458000	-2.86242800
H	-0.21843800	1.48537900	-2.68045700
C	-2.55362800	4.57091400	-3.23205700

H	-2.06462500	5.55150000	-3.11472000
H	-3.43393700	4.70546400	-3.87651400
H	-1.82942800	3.92743600	-3.75493200
C	5.57575800	0.25244000	0.04159000
H	5.32748600	-0.05167700	-0.99057800
C	5.80844100	-1.06186600	0.83612500
C	6.91370700	1.02176600	-0.02501400
H	4.86465900	-1.61995300	0.91433400
C	6.33255900	-0.74293700	2.24500700
C	6.83520200	-1.92509600	0.08526700
H	6.76885900	1.96453000	-0.57784300
C	7.45647000	1.32031300	1.38582600
C	7.93943600	0.15477200	-0.78032100
H	6.45469500	-1.67851200	2.81551500
H	5.59480400	-0.13371500	2.79044400
C	7.67488200	0.00160700	2.14593000
H	6.44747800	-2.17586600	-0.91491300
H	6.98376600	-2.87877900	0.62011400
C	8.16827600	-1.16749700	-0.02637400
H	6.75753100	1.95814700	1.94764700
H	8.40651600	1.87630600	1.30617800
H	7.57648600	-0.05125100	-1.80167500
H	8.89196800	0.70275500	-0.87994100
H	8.05463300	0.21907400	3.15857600
C	8.69605300	-0.86488800	1.38741400
H	8.90330000	-1.78211600	-0.57367400
H	9.66557400	-0.34092200	1.32700900
H	8.87305800	-1.80765400	1.93317600
C	-6.00318600	-1.48057000	1.12973200
H	-6.09929500	-1.48825200	2.23286200
C	-7.15533000	-2.34738700	0.56450300
C	-6.23469800	-0.01809900	0.67145800
H	-7.02883000	-3.39244900	0.88677900
C	-7.16890600	-2.27359100	-0.97277500
C	-8.50028200	-1.83331500	1.10852100
H	-5.43916900	0.62475800	1.06955500
C	-6.24345700	0.06058200	-0.86378600
C	-7.58153500	0.48687000	1.21510100
H	-7.97761500	-2.91105100	-1.36892800
H	-6.22513700	-2.66432700	-1.38203300
C	-7.38009600	-0.81548800	-1.41915400
H	-8.51254500	-1.89903800	2.20993000
H	-9.31831500	-2.47465000	0.73884000
C	-8.72534800	-0.37901800	0.66447300

H	-5.27292200	-0.26279700	-1.26773300
H	-6.37767300	1.10686400	-1.18255500
H	-7.57893000	0.45847700	2.31822700
H	-7.72632700	1.54062300	0.92088000
H	-7.38185300	-0.76389400	-2.52065900
C	-8.72707100	-0.30691500	-0.87364600
H	-9.69096100	-0.01603600	1.05489000
H	-8.89962800	0.73228500	-1.20219700
H	-9.55451500	-0.91411700	-1.27918000

TS9(triplet)

C	-0.37852000	-1.45773800	2.97698400
C	1.01460900	-0.82646100	2.83906100
H	-0.29821900	-2.51605400	3.24727500
H	1.22781600	-0.14559300	3.67354300
N	0.85588000	-0.04486300	1.59113300
N	-0.87226100	-1.35830700	1.57661600
C	2.15577000	-1.81601700	2.70360200
C	3.27795200	-1.72347600	3.53204900
C	2.14278200	-2.77770100	1.67960600
C	4.37556700	-2.57063200	3.34419900
H	3.30499300	-0.96457000	4.31841200
C	3.23976300	-3.61523900	1.48337600
H	1.28149800	-2.85599900	1.01678300
C	4.36228000	-3.51304100	2.31473100
H	5.25003500	-2.47752500	3.99185100
H	3.22151800	-4.33912300	0.66527100
H	5.22694800	-4.16065200	2.15216800
C	-1.33522400	-0.76640300	3.92572700
C	-2.33099300	-1.52133600	4.55957300
C	-1.29695300	0.62011100	4.13632200
C	-3.27026900	-0.90891900	5.39318100
H	-2.37144000	-2.60066500	4.39264400
C	-2.22920800	1.23170700	4.97699500
H	-0.54927900	1.22936400	3.62666100
C	-3.21910500	0.47085100	5.60750900
H	-4.04110900	-1.51167100	5.87938300
H	-2.18847700	2.31258500	5.13241700
H	-3.94847300	0.95268900	6.26306200
C	1.84147800	0.92929800	1.27151600
C	1.59859100	2.29511900	1.52776400
C	3.08772800	0.52802300	0.80532800
C	2.60793900	3.21754100	1.23733600
C	4.11483200	1.43289200	0.50955400

H	3.24282400	-0.53170000	0.64101800
C	3.83614000	2.78786300	0.72107000
H	2.43695200	4.28266200	1.38344100
H	4.58226400	3.54747600	0.48643900
C	-0.17191500	-0.46994600	0.81211800
Fe	-0.59444300	0.10296500	-1.03380800
C	-2.05293300	-2.03718100	1.20384200
C	-3.16970000	-1.35105200	0.72837500
C	-2.09770000	-3.44481900	1.30489100
C	-4.33942200	-2.00758800	0.30688100
H	-3.10939600	-0.26538700	0.67325900
C	-3.25269800	-4.11712900	0.90374900
C	-4.34811600	-3.40599900	0.40496500
H	-3.30406400	-5.20421300	0.95285300
H	-5.21479600	-3.98005000	0.08980200
O	0.39043800	2.62653300	2.05336400
O	-0.97217300	-4.06136800	1.75894000
C	0.16958800	3.96269500	2.46688800
H	0.86918900	4.24768400	3.27252700
H	0.26242300	4.67040500	1.63108800
H	-0.86120900	3.99995500	2.84230700
C	-0.89536300	-5.47055700	1.73871700
H	-1.03382600	-5.86409300	0.71685200
H	0.11184700	-5.72674700	2.09201600
H	-1.64368400	-5.92994000	2.40778400
C	-1.57219400	-0.60940600	-2.79971200
C	-1.23201100	-1.73808500	-2.01417100
H	-2.61880300	-0.29277500	-2.82928900
H	-1.02812100	-0.39924400	-3.72463700
H	-2.01116200	-2.16638600	-1.38332800
C	-0.13786700	-2.67179600	-2.34246800
C	0.19271200	-3.70162200	-1.43712500
C	0.58471900	-2.61794900	-3.55023100
C	1.19345800	-4.62878900	-1.72219200
H	-0.34223300	-3.75472900	-0.49119200
C	1.58229500	-3.54874300	-3.83901500
H	0.36405400	-1.83624800	-4.27820800
C	1.89710400	-4.56174700	-2.93034300
H	1.42383700	-5.41417300	-0.99629100
H	2.13018600	-3.47268600	-4.78135300
H	2.68484200	-5.28387800	-3.15649700
C	0.19830900	4.18716100	-1.12410400
C	-1.13323400	4.30894700	-0.63398200
C	-1.67320500	5.52979300	-0.23335100

C	-0.86054600	6.66584200	-0.33366500
C	0.45522500	6.57029000	-0.82050400
C	0.99144000	5.34121700	-1.21575800
C	0.41267900	2.77988700	-1.35288000
C	-0.75370800	2.06918200	-0.94949300
H	-2.68788100	5.59211100	0.16442200
H	-1.25419300	7.63700900	-0.02489100
H	1.06898800	7.47207100	-0.88609100
H	2.02086600	5.27706300	-1.57582800
N	-1.68656900	3.01999100	-0.57447400
C	-2.93453900	2.73792300	0.05009600
C	-4.14887000	3.09520600	-0.57509800
C	-2.91220000	2.13938600	1.31398600
C	-5.32968400	2.84979500	0.14256000
C	-4.10224800	1.89029000	1.99780300
H	-1.94418700	1.88047600	1.74166500
C	-5.31232300	2.25960600	1.40987600
H	-6.29018800	3.11778800	-0.29777400
H	-4.07524500	1.41489500	2.97924800
H	-6.25306900	2.08299600	1.93616500
C	1.52511300	2.05738200	-1.84679200
H	2.44656400	2.59475600	-2.11662600
N	1.46354100	0.75233300	-1.92157400
C	2.56770400	0.01651300	-2.36724100
C	3.45644000	0.46606800	-3.35643000
C	2.82371400	-1.23572800	-1.77379600
C	4.59193300	-0.27498800	-3.70578700
H	3.25853500	1.40724700	-3.87293500
C	3.95473200	-1.96626700	-2.09677900
H	2.11697400	-1.61701700	-1.03963200
C	4.85614100	-1.49039100	-3.06260400
H	5.25814700	0.10968100	-4.47767600
H	4.15817300	-2.92381900	-1.61721500
O	5.95077700	-2.26892600	-3.30075400
C	6.91610400	-1.81192800	-4.21611300
H	7.71222600	-2.56810900	-4.23993700
H	6.49854000	-1.69975500	-5.23335800
H	7.35025500	-0.84412800	-3.90465500
H	-1.15637100	1.05530000	-2.12125300
C	5.40887100	0.89646700	-0.08523800
H	5.14922400	0.54719600	-1.09817300
C	5.96687800	-0.33394000	0.68096800
C	6.54979400	1.92682700	-0.23791000
H	5.17226000	-1.08135500	0.81048500

C	6.49585600	0.09797800	2.05776700
C	7.10847000	-0.96819900	-0.13024800
H	6.17698300	2.81147900	-0.77973800
C	7.10349000	2.34795200	1.13724200
C	7.68470000	1.28646200	-1.05961900
H	6.85472000	-0.78635600	2.61093900
H	5.68124500	0.54035500	2.65439400
C	7.63998800	1.11265400	1.87992300
H	6.73576600	-1.31920100	-1.10343000
H	7.48989300	-1.85608600	0.40339700
C	8.23924400	0.05458700	-0.32229400
H	6.32277300	2.83303700	1.74235800
H	7.91050600	3.08842300	1.00072800
H	7.30401300	0.99195900	-2.05231900
H	8.48913300	2.02364700	-1.22490400
H	8.02479500	1.41579200	2.86842400
C	8.77544500	0.47842700	1.05647200
H	9.05340500	-0.39545300	-0.91573800
H	9.60330500	1.19908500	0.93942700
H	9.18478100	-0.39892700	1.58634700
C	-5.47371000	-1.16266900	-0.24438200
H	-5.20912700	-0.13000600	0.02563000
C	-6.89284400	-1.40184100	0.34299800
C	-5.58213800	-1.18276500	-1.79421500
H	-6.82298600	-1.43010100	1.44290000
C	-7.55129600	-2.69483300	-0.17518200
C	-7.78716100	-0.22219700	-0.09202000
H	-4.57437100	-1.06000400	-2.22111900
C	-6.20161400	-2.49753400	-2.29585000
C	-6.47588700	-0.00528000	-2.22371000
H	-8.57226900	-2.76584000	0.23773000
H	-7.02364600	-3.59075600	0.17787200
C	-7.61764800	-2.66843300	-1.71277500
H	-7.37516000	0.72439200	0.28771600
H	-8.79382700	-0.33713300	0.34489300
C	-7.88424000	-0.17351800	-1.62874100
H	-5.56347500	-3.35335400	-2.02695700
H	-6.25140800	-2.48158400	-3.39780800
H	-6.03349000	0.94080600	-1.87850000
H	-6.53289200	0.04341500	-3.32458100
H	-8.05703300	-3.61113800	-2.07980800
C	-8.49734100	-1.48404300	-2.15070900
H	-8.51630100	0.67918400	-1.92962500
H	-8.57442200	-1.45326100	-3.25105300

H	-9.52126400	-1.60897700	-1.75874100
C	-4.15344400	3.69646600	-1.97651800
C	-5.47879000	4.36148800	-2.35647800
C	-3.75102100	2.65317500	-3.03466600
H	-3.38010600	4.47999300	-1.99181900
H	-5.78610500	5.11779500	-1.61755900
H	-5.38044800	4.86214600	-3.33178100
H	-6.29373500	3.62529900	-2.45009000
H	-2.77847300	2.20084500	-2.80595900
H	-4.49702100	1.84681500	-3.09551000
H	-3.67943900	3.12463700	-4.02746800

TS9(quintet)

C	-0.26046200	-2.19988600	2.86375500
C	0.99436900	-1.30542200	2.95077900
H	-0.00086400	-3.26047200	2.97610200
H	0.98536300	-0.70306200	3.86898000
N	0.79251000	-0.41032800	1.77822200
N	-0.69594800	-1.95305000	1.46058700
C	2.31972400	-2.02869800	2.84293600
C	3.37404000	-1.69392900	3.69851500
C	2.53962100	-2.96664000	1.82104100
C	4.63279600	-2.28231200	3.54251200
H	3.21386900	-0.95005400	4.48317900
C	3.79744600	-3.54754200	1.65956500
H	1.72717800	-3.22726100	1.14087400
C	4.84843700	-3.20624400	2.51872200
H	5.44974700	-2.00363700	4.21140400
H	3.96469800	-4.26243400	0.85044300
H	5.83556700	-3.65362200	2.38218400
C	-1.38053300	-1.83589800	3.81336000
C	-2.18363900	-2.84224400	4.36208800
C	-1.68107800	-0.49254600	4.09225700
C	-3.26805900	-2.52064500	5.18326100
H	-1.96048500	-3.88840800	4.13710700
C	-2.75735100	-0.17227700	4.92161400
H	-1.07901500	0.30732900	3.65449200
C	-3.55545700	-1.18390100	5.46747800
H	-3.88767000	-3.31657900	5.60309300
H	-2.97594700	0.87523700	5.14195200
H	-4.39869000	-0.92891800	6.11374000
C	1.67677500	0.67955700	1.53887800
C	1.30552300	1.98582600	1.91520100
C	2.92120900	0.44439700	0.96958700

C	2.19693800	3.03036600	1.65353300
C	3.83508300	1.47334800	0.71184500
H	3.16195400	-0.57502900	0.68519600
C	3.43472300	2.77036900	1.05245600
H	1.92582400	4.05933100	1.88244400
H	4.08443300	3.62052600	0.84321800
C	-0.11946700	-0.87325700	0.90812900
Fe	-0.59065800	0.07097900	-0.89854600
C	-1.74027500	-2.71768900	0.88401400
C	-3.01567500	-2.18146400	0.70616600
C	-1.47056500	-4.05065900	0.52035400
C	-4.06311200	-2.93523300	0.15309500
H	-3.16900900	-1.15254200	1.02768000
C	-2.50377100	-4.82160400	-0.02111700
C	-3.77079100	-4.26095000	-0.19854400
H	-2.32891200	-5.85417300	-0.32137100
H	-4.55601500	-4.89257800	-0.61739800
O	0.09326300	2.13412200	2.50985500
O	-0.20032600	-4.48464900	0.72075100
C	-0.28872100	3.42211700	2.95981000
H	0.40532100	3.79290000	3.73408900
H	-0.33404500	4.14438400	2.13108600
H	-1.29332400	3.31301700	3.38734200
C	0.16278200	-5.77920400	0.29108700
H	0.02145600	-5.89544200	-0.79650200
H	1.22773500	-5.89579900	0.53069900
H	-0.41293000	-6.55972800	0.81826900
C	-2.02972300	0.53445500	-2.98904300
C	-1.76303600	-0.80789200	-2.57640800
H	-3.04328600	0.91512900	-2.83306200
H	-1.52855400	0.93892700	-3.87487800
H	-2.55046900	-1.33153600	-2.02709700
C	-0.75469000	-1.67999100	-3.17167800
C	-0.68897000	-3.04132200	-2.78441800
C	0.19065300	-1.23226300	-4.12315900
C	0.27561200	-3.89685900	-3.30610200
H	-1.42072600	-3.41675800	-2.06734500
C	1.15422200	-2.09397300	-4.64225600
H	0.18394100	-0.18910500	-4.44024100
C	1.21453300	-3.42930600	-4.23525200
H	0.29568000	-4.94468000	-2.99170900
H	1.88684200	-1.70914400	-5.35570600
H	1.98328100	-4.09537200	-4.63282800
C	0.20509100	4.21799200	-0.90088200

C	-1.10406200	4.39852400	-0.36631200
C	-1.51329900	5.61550700	0.18307100
C	-0.60058500	6.67846500	0.18196700
C	0.68873600	6.52299800	-0.35285500
C	1.09802300	5.29913300	-0.89219400
C	0.30772600	2.82456900	-1.26167400
C	-0.93229500	2.17502300	-0.94060700
H	-2.50639100	5.72811700	0.61956000
H	-0.89811500	7.63977700	0.60768000
H	1.38191600	7.36766300	-0.34061900
H	2.11032500	5.17929800	-1.28551100
N	-1.77495300	3.17120000	-0.42075900
C	-2.99207100	2.90315400	0.25745300
C	-4.19012100	3.55248800	-0.10689200
C	-2.97201000	1.98660600	1.32171500
C	-5.33101900	3.30147200	0.67404300
C	-4.12250300	1.74148800	2.06685600
H	-2.02729000	1.49897200	1.56228300
C	-5.30513000	2.41921500	1.75345000
H	-6.27019600	3.79420100	0.41363400
H	-4.09021900	1.03238500	2.89571800
H	-6.21128000	2.24518000	2.33797300
C	1.38732700	2.05009700	-1.70651700
H	2.35152000	2.52517100	-1.92773500
N	1.26032800	0.72963100	-1.78541900
C	2.34099200	-0.04939400	-2.20582300
C	3.31963500	0.40007900	-3.10923600
C	2.47880100	-1.35803800	-1.69594100
C	4.42019900	-0.39436600	-3.45014200
H	3.21638000	1.38206300	-3.57343300
C	3.57206900	-2.14523800	-2.01739900
H	1.70164300	-1.74426100	-1.03645600
C	4.56607600	-1.66727400	-2.88748500
H	5.15525000	-0.00303300	-4.15334700
H	3.68033500	-3.15088100	-1.60857000
O	5.62018800	-2.50313400	-3.12183200
C	6.63537500	-2.07251500	-3.99378800
H	7.37701600	-2.88138300	-4.04138300
H	6.24982400	-1.87920400	-5.01177300
H	7.13144600	-1.15522600	-3.62581700
H	-1.45295400	1.54563900	-1.95119700
C	5.13269500	1.10932700	0.00892700
H	4.83139800	0.74432300	-0.98599400
C	5.88761600	-0.06188000	0.69523300

C	6.13179500	2.26386800	-0.21823900
H	5.18917900	-0.88827800	0.88412300
C	6.50111900	0.40531200	2.02386800
C	7.00138100	-0.55912400	-0.23955500
H	5.61214700	3.11168100	-0.69472800
C	6.77287500	2.71920100	1.10719700
C	7.24096200	1.76358800	-1.16380600
H	7.00211200	-0.44346600	2.51963300
H	5.70701600	0.75141400	2.70586800
C	7.50994900	1.53760100	1.75993300
H	6.56378300	-0.93656900	-1.17555600
H	7.52848500	-1.40786700	0.22979400
C	7.99099500	0.58374100	-0.51842800
H	6.01093600	3.10641100	1.80037000
H	7.47813400	3.54540300	0.91208900
H	6.79808700	1.44917100	-2.12366300
H	7.94561100	2.58400100	-1.38315200
H	7.95933500	1.86518700	2.71268300
C	8.61616100	1.04017000	0.81191600
H	8.78420200	0.23352200	-1.20100700
H	9.34827900	1.84636200	0.63243000
H	9.16609500	0.20340100	1.27610700
C	-5.48682400	-2.40427100	0.01923800
H	-6.07180400	-2.91922000	0.80477900
C	-6.15859000	-2.74751900	-1.33816700
C	-5.64267800	-0.88580500	0.26084400
H	-6.06308300	-3.82569000	-1.53962200
C	-5.49587900	-1.95225800	-2.47490300
C	-7.65404600	-2.39123400	-1.26722300
H	-5.17587300	-0.61352000	1.21811300
C	-4.99679200	-0.07922800	-0.87897600
C	-7.13901900	-0.53426500	0.32876300
H	-5.95987600	-2.22508400	-3.43776700
H	-4.42868800	-2.21036300	-2.55354500
C	-5.66116700	-0.44490000	-2.21586100
H	-8.14459100	-2.97314800	-0.46807300
H	-8.14548300	-2.66791800	-2.21535200
C	-7.82093500	-0.88585700	-1.00376600
H	-3.91639500	-0.26526200	-0.92581100
H	-5.11663200	0.99379600	-0.67822700
H	-7.62115600	-1.08094500	1.15749500
H	-7.25125900	0.54133700	0.54081200
H	-5.18552800	0.12177500	-3.03242500
C	-7.15751400	-0.09348500	-2.14469300

H	-8.89365400	-0.63461300	-0.95024700
H	-7.28057400	0.98959500	-1.97401100
H	-7.65051600	-0.32736100	-3.10385500
C	-4.28993600	4.39587800	-1.36957800
C	-5.04299500	5.71511500	-1.16262600
C	-4.92582100	3.56205000	-2.49547300
H	-3.26932800	4.64469400	-1.68839600
H	-4.59804000	6.31064900	-0.35104500
H	-5.01132600	6.32036800	-2.08209000
H	-6.10441300	5.54872600	-0.91770600
H	-4.34897700	2.64440300	-2.68018300
H	-5.95487100	3.26351900	-2.23686900
H	-4.96287100	4.13673500	-3.43480200

TS10(singlet)

C	1.00050800	-2.75400300	-0.97858700
C	-0.48252500	-2.58644300	-1.40126800
H	1.15632900	-3.72629700	-0.49027200
H	-0.58314900	-2.64982400	-2.49298900
N	-0.76198900	-1.19672500	-0.96932800
N	1.11609700	-1.67895100	0.03104300
C	-1.43905200	-3.56698600	-0.75407100
C	-2.49622800	-4.10826700	-1.49409600
C	-1.32940900	-3.88851900	0.60799200
C	-3.43434000	-4.94896500	-0.88878400
H	-2.59704200	-3.85215700	-2.55154200
C	-2.26045100	-4.73337600	1.21263200
H	-0.52051000	-3.46018400	1.20334000
C	-3.32010200	-5.26192500	0.46730200
H	-4.25875300	-5.35466500	-1.47837000
H	-2.16098100	-4.97912800	2.27272000
H	-4.05277200	-5.91803300	0.94301300
C	1.97039800	-2.62828500	-2.13766200
C	2.92781800	-3.62391600	-2.36737300
C	1.93295000	-1.50734600	-2.98064600
C	3.85608800	-3.49386300	-3.40427500
H	2.96233300	-4.49959600	-1.71407100
C	2.86060200	-1.37719900	-4.01511300
H	1.18042300	-0.73246600	-2.82373400
C	3.82850200	-2.36501100	-4.22698500
H	4.60468400	-4.27322600	-3.56449900
H	2.83289400	-0.49169800	-4.65410800
H	4.55769400	-2.25546800	-5.03307800
C	-1.90099800	-0.48694800	-1.43316700

C	-1.73796400	0.55358400	-2.36981300
C	-3.16678900	-0.81486300	-0.96234200
C	-2.87590200	1.25006100	-2.78952000
C	-4.31656200	-0.11875500	-1.35502600
H	-3.23520500	-1.60459300	-0.22211300
C	-4.13633900	0.91918900	-2.27906300
H	-2.78549200	2.07800800	-3.49069500
H	-4.99008300	1.50827500	-2.61594400
C	0.11647100	-0.73478000	-0.05607000
Fe	0.16494500	0.71709200	1.10098500
C	2.18688700	-1.52569300	0.93450300
C	3.50690100	-1.73097900	0.52657300
C	1.94915900	-1.11811200	2.26925400
C	4.60553200	-1.53290300	1.36988100
H	3.66609600	-2.01825600	-0.50503400
C	3.03012600	-0.93257200	3.13179900
C	4.33839900	-1.13669900	2.68682400
H	2.86136600	-0.60708500	4.15728100
H	5.15514400	-0.95503300	3.38580200
O	-0.47706600	0.80637900	-2.79725000
O	0.63901000	-0.88291000	2.63584200
C	-0.28125400	1.75832400	-3.82792600
H	-0.76199100	1.42695900	-4.76575800
H	-0.66330100	2.74860200	-3.54615900
H	0.80391700	1.83207200	-3.97290200
C	0.32789400	-0.69227500	4.01223600
H	0.69226900	-1.54229600	4.61020300
H	0.76452900	0.24586700	4.39141700
H	-0.76569200	-0.63353000	4.06351200
C	-0.01656200	3.84358600	2.46299900
C	0.49614900	5.11106300	2.13005400
C	-1.28714300	3.78924600	3.06836100
C	-0.23866300	6.27165000	2.37382900
H	1.47359900	5.18914600	1.65741900
C	-2.02114000	4.94934400	3.31437000
H	-1.70184400	2.81747800	3.34309600
C	-1.50421600	6.20021300	2.96361100
H	0.18037100	7.24208300	2.09482300
H	-3.00519500	4.87553900	3.78554300
H	-2.07940300	7.11000500	3.15149300
C	-0.95999900	3.90306500	-1.08348000
C	0.42276300	4.01407100	-1.40560500
C	0.85358500	4.79903300	-2.46955700
C	-0.10004700	5.54137600	-3.18718300

C	-1.45764000	5.47916400	-2.85085100
C	-1.89491700	4.65324600	-1.80583800
C	-1.06113600	2.86966000	-0.08868700
C	0.27063500	2.36705500	0.22201400
H	1.89888200	4.80997700	-2.76912100
H	0.22742400	6.16575300	-4.02198900
H	-2.18433300	6.06593400	-3.41829600
H	-2.95874000	4.57300700	-1.57096700
N	1.16101900	3.13162600	-0.58627100
C	2.50355700	2.75696500	-0.87197800
C	3.58752000	3.67142300	-0.83028300
C	2.74046900	1.41257300	-1.20204400
C	4.87009600	3.17217700	-1.11008100
C	4.02471100	0.94661300	-1.47033100
H	1.88004200	0.74940500	-1.25204900
C	5.10245400	1.83212100	-1.41950800
H	5.71843900	3.85973200	-1.07393700
H	4.17624600	-0.09762700	-1.74164600
H	6.11674700	1.48463200	-1.63102100
C	-2.09915000	2.17821500	0.49280100
H	-3.15057500	2.44405800	0.35078800
N	-1.76215000	1.08777000	1.21278100
C	-2.72640600	0.39353000	1.92890500
C	-3.92341000	0.99640600	2.39220600
C	-2.56220400	-0.97938000	2.19327800
C	-4.92157400	0.24579400	3.00151700
H	-4.06769100	2.06995600	2.26572200
C	-3.56274900	-1.73828500	2.80416400
H	-1.65382900	-1.46861700	1.84977700
C	-4.76523300	-1.13549600	3.19353300
H	-5.84841700	0.71474200	3.33856400
H	-3.40184500	-2.80733900	2.93370600
O	-5.82328700	-1.80060700	3.74558400
C	-5.74204100	-3.19912700	3.85838500
H	-6.69949500	-3.54045400	4.27454400
H	-5.58487500	-3.68069700	2.87560900
H	-4.92646000	-3.51191800	4.53685900
C	3.44722900	5.14574900	-0.45899200
C	3.98035200	6.07751700	-1.56135000
C	4.16706200	5.48206200	0.85998400
H	2.37803700	5.35718000	-0.32396800
H	3.53756700	5.86277100	-2.54312500
H	3.75811900	7.12761800	-1.31417500
H	5.07370600	5.98607300	-1.66328300

H	3.81217300	4.87097900	1.69979500
H	5.25375100	5.32422800	0.76982000
H	4.00381000	6.53939700	1.12262300
C	0.70444100	2.59599600	2.12835400
H	0.27597700	1.81000600	2.78195800
C	2.21442600	2.59187400	2.28359100
H	2.51687700	2.82089400	3.32132900
H	2.65319000	1.61891000	2.01304100
H	2.68802500	3.32757000	1.63047200
C	-5.64837100	-0.44167700	-0.68705300
H	-5.59919100	0.02423200	0.31254500
C	-5.87666500	-1.95968200	-0.45625700
C	-6.89557800	0.12937500	-1.40022300
H	-5.00560900	-2.39409200	0.05413500
C	-6.09571400	-2.66939100	-1.80239300
C	-7.11220700	-2.16099700	0.43724500
H	-6.76318900	1.20979200	-1.56906600
C	-7.12482400	-0.58618000	-2.74444100
C	-8.12797100	-0.07607500	-0.50025300
H	-6.22729400	-3.75189400	-1.63457800
H	-5.20729300	-2.54812100	-2.44334800
C	-7.33970200	-2.08973900	-2.49891800
H	-6.95500400	-1.68239000	1.41619300
H	-7.25785100	-3.23918500	0.62668400
C	-8.35461500	-1.57694400	-0.25323900
H	-6.26369900	-0.43573100	-3.41409400
H	-8.00584300	-0.15546700	-3.25052800
H	-7.98258400	0.44602200	0.46038900
H	-9.01729300	0.36826800	-0.97912300
H	-7.49752000	-2.60207200	-3.46311100
C	-8.57293200	-2.29028600	-1.59949000
H	-9.23970000	-1.71701100	0.38999600
H	-9.47295100	-1.89209600	-2.09896300
H	-8.74826000	-3.36708800	-1.43169600
C	6.02951500	-1.62143300	0.83195100
H	6.32132600	-0.57805100	0.60663500
C	6.17333800	-2.42570200	-0.48464100
C	7.05607300	-2.17996400	1.85042300
H	5.46689300	-2.04843500	-1.23825200
C	5.91014700	-3.92081200	-0.22998700
C	7.59885300	-2.24962700	-1.03560500
H	6.98379700	-1.62298300	2.79683800
C	6.78304400	-3.67249800	2.10663500
C	8.47914500	-2.00622700	1.29156300

H	5.99155000	-4.47656400	-1.17917200
H	4.88536400	-4.07446900	0.14263300
C	6.92700500	-4.45865800	0.79120800
H	7.79261800	-1.18355900	-1.24364400
H	7.69171700	-2.78594600	-1.99511000
C	8.62371700	-2.78716200	-0.02419000
H	5.77075200	-3.80792100	2.52125400
H	7.49317200	-4.05752400	2.85789000
H	8.69005800	-0.93652300	1.12360700
H	9.21425900	-2.36716300	2.03065900
H	6.73367800	-5.52837300	0.97708600
C	8.35190400	-4.27960800	0.23688700
H	9.64371300	-2.65896600	-0.42316800
H	9.09019800	-4.67838400	0.95336300
H	8.46834200	-4.85443600	-0.69779100

TS10(triplet)

C	1.19109400	-3.07160500	-0.66346500
C	-0.29512500	-3.05896800	-1.12346500
H	1.39468700	-3.94288200	-0.02585200
H	-0.36572900	-3.29108500	-2.19401700
N	-0.64549500	-1.63724300	-0.89719200
N	1.24247200	-1.83385500	0.16351500
C	-1.23710400	-3.95083700	-0.34304700
C	-2.12683400	-4.79576300	-1.01341000
C	-1.31469300	-3.84780500	1.05566700
C	-3.09115500	-5.51987800	-0.30501300
H	-2.08537500	-4.86495300	-2.10332100
C	-2.27589600	-4.56861400	1.76351200
H	-0.64279200	-3.17114700	1.58882600
C	-3.17162600	-5.40290400	1.08361600
H	-3.79144100	-6.16233500	-0.84312600
H	-2.33727700	-4.47039800	2.84986600
H	-3.93449900	-5.95430500	1.63763500
C	2.16354100	-3.04778900	-1.82479300
C	3.07100100	-4.09696800	-2.00984500
C	2.17358600	-1.96422000	-2.71730900
C	3.99783000	-4.05796800	-3.05567200
H	3.07011500	-4.94057700	-1.31493100
C	3.10133100	-1.92498500	-3.75883700
H	1.45822900	-1.14806900	-2.59673500
C	4.02006700	-2.96716400	-3.92774500
H	4.70938400	-4.87715700	-3.18169100
H	3.11204600	-1.06933400	-4.43765700

H	4.75066100	-2.92828000	-4.73895800
C	-1.81405500	-1.01014800	-1.41374200
C	-1.67004300	0.03027000	-2.35497600
C	-3.06892000	-1.36515100	-0.93501300
C	-2.82530300	0.69561700	-2.77659000
C	-4.23488800	-0.70617000	-1.34162500
H	-3.12004900	-2.13989400	-0.17789200
C	-4.07873800	0.32865600	-2.27231900
H	-2.75606900	1.52225400	-3.48232300
H	-4.94753800	0.89212600	-2.61434900
C	0.19483400	-1.01575900	-0.06904600
Fe	-0.12568700	0.82931400	0.78791600
C	2.30890900	-1.48867400	1.02678500
C	3.62817600	-1.77762300	0.66770200
C	2.06786400	-0.82023700	2.24796900
C	4.72528500	-1.42580300	1.45993000
H	3.79146600	-2.25690800	-0.28948000
C	3.14996700	-0.49136900	3.06669400
C	4.45653600	-0.78852100	2.67800500
H	2.97979000	0.02780700	4.00876000
H	5.27345300	-0.48215900	3.33243400
O	-0.41301700	0.31881400	-2.76831100
O	0.77017100	-0.47548900	2.55668000
C	-0.20848200	1.44044100	-3.61210300
H	-0.65851600	1.27676400	-4.60683500
H	-0.61849800	2.35681700	-3.16445600
H	0.87786500	1.55401500	-3.71440500
C	0.43444900	-0.17852000	3.91002800
H	0.87359700	0.77800200	4.23080800
H	-0.65964000	-0.09942100	3.93776600
H	0.77174600	-0.98942500	4.57474600
C	-0.23387900	4.29108000	2.12810900
C	0.41601500	5.47974900	1.70823900
C	-1.62890800	4.37305700	2.37067700
C	-0.29382900	6.65866700	1.50378300
H	1.49007500	5.46784300	1.52907400
C	-2.33244200	5.55989600	2.17243200
H	-2.15277000	3.48624800	2.73169800
C	-1.67691300	6.71050600	1.72286600
H	0.23679900	7.55240400	1.16359300
H	-3.40832400	5.58615400	2.36986200
H	-2.23064500	7.63731000	1.55568500
C	-0.85553900	4.13907700	-1.37818200
C	0.54492700	4.25109500	-1.60410700

C	1.04865200	5.08131400	-2.60143700
C	0.14727000	5.87532100	-3.32709500
C	-1.22848900	5.82383400	-3.06940100
C	-1.73708700	4.94788000	-2.10259500
C	-1.03619200	3.05985000	-0.42860200
C	0.27640600	2.55687800	-0.05928100
H	2.11036800	5.10134000	-2.83363900
H	0.52997400	6.53754800	-4.10761100
H	-1.91102300	6.45874800	-3.63960200
H	-2.81332500	4.88109900	-1.92650800
N	1.21175900	3.32239100	-0.76956200
C	2.55238500	2.87617600	-0.97280600
C	3.68039200	3.72573600	-0.86935800
C	2.73399300	1.51043500	-1.26176700
C	4.94852000	3.14845600	-1.05910900
C	4.00229400	0.96620900	-1.43491600
H	1.84467500	0.88674500	-1.34166500
C	5.12414600	1.79276300	-1.33074300
H	5.83050700	3.78752500	-0.97351800
H	4.10927300	-0.09530600	-1.66141500
H	6.12908500	1.38466500	-1.46463600
C	-2.18390500	2.49838700	0.11438900
H	-3.17320400	2.89726600	-0.13400600
N	-2.06941300	1.41353700	0.91645500
C	-3.14073900	0.88343400	1.61465200
C	-4.34707300	1.56778700	1.86724900
C	-3.03329400	-0.43483400	2.12222000
C	-5.40442900	0.95826800	2.55243400
H	-4.46279600	2.60021600	1.53583200
C	-4.07819700	-1.04445200	2.80106100
H	-2.11121800	-0.98903200	1.93451500
C	-5.28421400	-0.35689500	3.01711700
H	-6.31915400	1.52793100	2.71666300
H	-3.99277100	-2.07209600	3.15998500
O	-6.27122500	-1.04941600	3.66195900
C	-7.50076000	-0.40603000	3.88930500
H	-8.14355600	-1.12681700	4.41241300
H	-7.38363900	0.49412500	4.52011400
H	-7.99150400	-0.11080700	2.94344100
C	3.60208800	5.20604700	-0.50859000
C	4.26062000	6.10064400	-1.57269800
C	4.24821900	5.49599300	0.85813700
H	2.54118500	5.47973600	-0.43899700
H	3.87863700	5.90211200	-2.58379800

H	4.07923600	7.16298500	-1.34519000
H	5.35186400	5.94960900	-1.59826400
H	3.80394700	4.89411300	1.66077700
H	5.32937300	5.28367100	0.83961300
H	4.12019300	6.55808400	1.12143200
C	0.44871900	3.02007000	2.19891500
H	-0.11281900	2.28876700	2.79253700
C	1.93907100	2.95476000	2.38822900
H	2.27080700	3.43928100	3.32856200
H	2.30491000	1.91873000	2.39883800
H	2.47034000	3.45026800	1.56864200
C	-5.55557200	-1.04696300	-0.66433800
H	-5.52993800	-0.52437700	0.30592800
C	-5.72704500	-2.55807800	-0.35724100
C	-6.81774800	-0.55992400	-1.41138600
H	-4.84655100	-2.93283100	0.18293800
C	-5.90549400	-3.34406900	-1.66642800
C	-6.95997700	-2.76323700	0.53848800
H	-6.72575700	0.51510600	-1.63219000
C	-7.00742400	-1.35075000	-2.71871100
C	-8.04652900	-0.76655600	-0.50729200
H	-5.99373200	-4.42164200	-1.44506300
H	-5.01791500	-3.21964300	-2.30823700
C	-7.16601400	-2.84769600	-2.39800600
H	-6.82407300	-2.23473200	1.49441700
H	-7.06581500	-3.83557700	0.77835200
C	-8.21800100	-2.26061100	-0.18642000
H	-6.14596500	-1.19946800	-3.38820800
H	-7.89965500	-0.98173700	-3.25306100
H	-7.92262900	-0.19121800	0.42578900
H	-8.94925900	-0.37921000	-1.00994800
H	-7.29796600	-3.41355500	-3.33572300
C	-8.39851900	-3.04808100	-1.49678700
H	-9.10265000	-2.40270400	0.45740900
H	-9.30822000	-2.70929700	-2.02195200
H	-8.53566100	-4.12065400	-1.27557900
C	6.15393200	-1.60984200	0.96305200
H	6.48097500	-0.60161800	0.64580400
C	6.30492400	-2.54067500	-0.26552500
C	7.14419200	-2.08960700	2.05584800
H	5.61803600	-2.22369800	-1.06502900
C	6.01420400	-4.00264700	0.12211200
C	7.74530000	-2.44114200	-0.79849800
H	7.05781200	-1.44597000	2.94440000

C	6.83645700	-3.54617000	2.44365900
C	8.58224800	-1.99267600	1.51733100
H	6.10877200	-4.64455000	-0.76958500
H	4.98004700	-4.11144500	0.48323200
C	6.99864900	-4.45597600	1.21295800
H	7.96296600	-1.40342300	-1.10328200
H	7.84960400	-3.06896800	-1.69952700
C	8.73965000	-2.89508300	0.28297000
H	5.81124100	-3.62404200	2.84142300
H	7.51945700	-3.86954700	3.24726500
H	8.81720600	-0.94687400	1.25649300
H	9.29461600	-2.29645300	2.30292600
H	6.78280000	-5.50028800	1.49424000
C	8.43811200	-4.35174100	0.67804000
H	9.76951900	-2.82005100	-0.10398700
H	9.15393400	-4.69083700	1.44617900
H	8.56516600	-5.01490500	-0.19470800

TS10(quintet)

C	-0.15752300	-3.27804900	0.35556600
C	1.31854100	-2.98521600	0.74846000
H	-0.20604000	-4.07957900	-0.39331300
H	1.52273100	-3.33844400	1.76818400
N	1.32815100	-1.50235600	0.72724000
N	-0.54378500	-1.98960300	-0.27024400
C	2.33399900	-3.57511800	-0.20817900
C	3.29258400	-4.48412000	0.24939400
C	2.34795500	-3.18213400	-1.55689600
C	4.26752800	-4.98627100	-0.61857100
H	3.29256200	-4.78451300	1.30040600
C	3.32469400	-3.67819200	-2.42003000
H	1.60402600	-2.47430200	-1.92787700
C	4.29098400	-4.57748500	-1.95289100
H	5.01784600	-5.68676800	-0.24555200
H	3.33689000	-3.35726600	-3.46441300
H	5.06087000	-4.95562200	-2.62929100
C	-1.06828200	-3.60556600	1.51913600
C	-1.88586100	-4.73976000	1.48159300
C	-1.15091000	-2.73596600	2.61990000
C	-2.78348100	-5.00464800	2.52079100
H	-1.83596000	-5.41128800	0.62072100
C	-2.04126200	-3.00452100	3.65978600
H	-0.53490200	-1.83372800	2.65333400
C	-2.86405600	-4.13653600	3.61121700

H	-3.42446300	-5.88771000	2.47287400
H	-2.10284100	-2.31982500	4.50872300
H	-3.56749100	-4.33786600	4.42237400
C	2.37634000	-0.69776700	1.25556000
C	2.06964900	0.36243400	2.13531000
C	3.69446900	-0.89885300	0.84726700
C	3.10725600	1.19832000	2.55857000
C	4.74597800	-0.07916400	1.26972400
H	3.88341000	-1.69220000	0.13412100
C	4.41741100	0.97585800	2.13055700
H	2.89660700	2.04321700	3.21201000
H	5.19079100	1.66764100	2.46696300
C	0.28282700	-0.98551000	0.05352400
Fe	0.01150800	1.01468100	-0.12773900
C	-1.66545200	-1.83645300	-1.12319000
C	-2.95418300	-2.05195400	-0.64235900
C	-1.46405000	-1.45024200	-2.46657700
C	-4.08854700	-1.92371200	-1.45615300
H	-3.05888200	-2.29980600	0.40889200
C	-2.58344100	-1.32373000	-3.29295200
C	-3.86660100	-1.56480800	-2.79166400
H	-2.46835800	-1.02327300	-4.33368200
H	-4.70858100	-1.44170700	-3.47288200
O	0.76413300	0.52528300	2.49129500
O	-0.18520400	-1.21357500	-2.85878500
C	0.41772000	1.61472100	3.33761700
H	0.92670700	1.53092700	4.31229700
H	0.65808800	2.57659300	2.86045800
H	-0.66675100	1.55713800	3.48299700
C	0.04781900	-0.60919600	-4.11738700
H	-0.49742100	0.34561100	-4.20525600
H	1.12564300	-0.40845000	-4.16784900
H	-0.24224800	-1.27566800	-4.94788200
C	-1.23603100	3.58079500	-2.22239100
C	-0.00372200	3.92230000	-2.81977800
C	-2.18937400	4.61185400	-2.06490800
C	0.26509800	5.22611800	-3.23478100
H	0.75322500	3.14650900	-2.94938600
C	-1.91599800	5.91546200	-2.47329300
H	-3.14947600	4.39150700	-1.59818100
C	-0.68659900	6.23562200	-3.06065700
H	1.23006900	5.45580800	-3.69513300
H	-2.66999300	6.69367600	-2.32735600
H	-0.47441400	7.25940800	-3.37804600

C	-0.67917100	4.73003500	1.25869900
C	-1.97548300	4.28792000	1.64428100
C	-2.73969600	5.00943000	2.55769200
C	-2.24850800	6.24116700	3.01536900
C	-1.00754600	6.72584900	2.58285900
C	-0.21198100	5.96758000	1.71635000
C	-0.07935800	3.65405200	0.49732900
C	-1.05843500	2.57856500	0.39254100
H	-3.68428100	4.62099700	2.92946700
H	-2.84358600	6.82190900	3.72436000
H	-0.64714600	7.69227300	2.94387900
H	0.77514700	6.32695600	1.41558200
N	-2.21596700	3.02177200	1.05789500
C	-3.27410600	2.14391400	1.42680800
C	-4.64380200	2.50234400	1.33759800
C	-2.92591700	0.84790800	1.85402500
C	-5.59408000	1.54656500	1.73593400
C	-3.89122400	-0.08247900	2.22274900
H	-1.87012100	0.57923700	1.87236000
C	-5.24147100	0.27246000	2.17673300
H	-6.65311500	1.80792000	1.67593200
H	-3.58598900	-1.07559100	2.55595600
H	-6.01318500	-0.43889100	2.47702700
C	1.20223200	3.50286200	-0.02880300
H	1.90156900	4.34652800	-0.02845200
N	1.54860700	2.31009200	-0.54870600
C	2.76939400	2.04035600	-1.14500900
C	3.89191500	2.89230600	-1.12149300
C	2.92330100	0.80048500	-1.81342200
C	5.10131300	2.52590400	-1.72031600
H	3.83660200	3.85507300	-0.61265500
C	4.12245100	0.42763600	-2.40285100
H	2.07079200	0.11844900	-1.84014100
C	5.23090500	1.28831900	-2.36269800
H	5.94276300	3.21676500	-1.66399300
H	4.23061900	-0.54200500	-2.89338300
O	6.38407700	0.83044900	-2.94143600
C	7.49590500	1.68946700	-2.98388000
H	8.29305900	1.15117100	-3.51431100
H	7.27034800	2.62616500	-3.52593100
H	7.85720500	1.94935600	-1.97142900
C	-5.15144200	3.81926900	0.75331100
C	-6.07494000	4.57752200	1.72237900
C	-5.90209700	3.59781000	-0.57353700

H	-4.28258200	4.45544300	0.54000800
H	-5.62377200	4.71522200	2.71443600
H	-6.31594200	5.57390600	1.31981500
H	-7.02661400	4.04064500	1.86467400
H	-5.28218300	3.08935000	-1.32286700
H	-6.80745800	2.98874700	-0.42025300
H	-6.21726400	4.56511100	-0.99641600
C	-1.46527000	2.23235900	-1.70143500
H	-0.76033900	1.51725400	-2.15895600
C	-2.86986600	1.68750400	-1.68892900
H	-3.28321800	1.60070400	-2.71008300
H	-2.91863900	0.69742600	-1.22023800
H	-3.53965000	2.33714500	-1.12117500
C	6.15208900	-0.24762200	0.70720300
H	6.23664800	0.50201400	-0.09693900
C	6.42767400	-1.62994400	0.06540700
C	7.28170300	0.04502400	1.72639200
H	5.64642700	-1.86096600	-0.67334500
C	6.47266700	-2.72511900	1.14566500
C	7.77874000	-1.58724600	-0.66849700
H	7.11328600	1.02513900	2.19825000
C	7.31753200	-1.04882900	2.80714300
C	8.63101200	0.08472200	0.98781000
H	6.64805400	-3.70440400	0.67032000
H	5.50563000	-2.79547900	1.66759300
C	7.59416000	-2.41339700	2.15142400
H	7.73705300	-0.82951600	-1.46592700
H	7.96309500	-2.55954500	-1.15687600
C	8.90680200	-1.27597400	0.32819500
H	6.35849900	-1.07551400	3.34985200
H	8.10201100	-0.81779200	3.54792900
H	8.61700700	0.88039600	0.22338800
H	9.43808500	0.33425800	1.69772800
H	7.62379800	-3.19696000	2.92737100
C	8.94520900	-2.36621400	1.41442000
H	9.87453500	-1.24375600	-0.20042000
H	9.75994600	-2.16015100	2.12984700
H	9.15970400	-3.34729300	0.95677700
C	-5.48500900	-2.08788100	-0.86415800
H	-5.75335500	-1.10226200	-0.44060300
C	-5.55375800	-3.11417700	0.29754300
C	-6.57252100	-2.46650100	-1.90006700
H	-4.80445000	-2.86710300	1.06301100
C	-5.29882200	-4.53374600	-0.23767700

C	-6.94453300	-3.05954900	0.95230000
H	-6.56166000	-1.74536400	-2.73166300
C	-6.32158200	-3.88803400	-2.43675000
C	-7.95910600	-2.41259000	-1.23468500
H	-5.31739400	-5.25367400	0.59786300
H	-4.29633500	-4.59595400	-0.69043600
C	-6.37614700	-4.89651900	-1.27542200
H	-7.13082600	-2.05049200	1.35457300
H	-6.97866500	-3.75881800	1.80513700
C	-8.02563500	-3.42130500	-0.07708200
H	-5.34243000	-3.94301500	-2.93822300
H	-7.08462500	-4.14044300	-3.19259200
H	-8.15823300	-1.39387200	-0.86120900
H	-8.73762700	-2.63960600	-1.98260600
H	-6.19095900	-5.91316900	-1.66093600
C	-7.76511400	-4.83974800	-0.61442000
H	-9.02077900	-3.37953600	0.39643000
H	-8.54359200	-5.11666900	-1.34576900
H	-7.82296800	-5.57186800	0.20931000

TS10(R_a,R)

C	0.86418200	-2.45031200	-1.79207100
C	-0.54934400	-1.98916700	-2.22294500
H	0.87204500	-3.52822000	-1.57439000
H	-0.56848600	-1.73632200	-3.29196800
N	-0.71391400	-0.75862400	-1.41794300
N	1.03311900	-1.69786000	-0.52889000
C	-1.66349000	-2.97357200	-1.93006200
C	-2.64612400	-3.23322600	-2.89056900
C	-1.78236900	-3.55520500	-0.65711300
C	-3.73762100	-4.05428900	-2.59112300
H	-2.56797500	-2.77000600	-3.87753100
C	-2.87375500	-4.37033600	-0.35648800
H	-1.03009300	-3.34700400	0.10583200
C	-3.85799900	-4.61930600	-1.32041500
H	-4.50222700	-4.23920900	-3.34841400
H	-2.96146800	-4.81220200	0.63946000
H	-4.71708700	-5.24976900	-1.07945800
C	1.96610800	-2.13788300	-2.78360700
C	3.03115700	-3.03152500	-2.94957900
C	1.97663200	-0.92513200	-3.49111700
C	4.09511900	-2.72412600	-3.80228500
H	3.03410100	-3.97223600	-2.39299100
C	3.03447300	-0.62201600	-4.34996300

H	1.16444200	-0.20743200	-3.35548600
C	4.09878200	-1.51730900	-4.50584500
H	4.92184000	-3.42891300	-3.91533300
H	3.03444400	0.32668700	-4.89172800
H	4.92810100	-1.27359400	-5.17402600
C	-1.80485700	0.12433600	-1.62944600
C	-1.59361800	1.34433200	-2.30243300
C	-3.07681400	-0.21328300	-1.18229100
C	-2.68174900	2.20522900	-2.47637900
C	-4.18187000	0.63147200	-1.34648500
H	-3.18696700	-1.15334800	-0.65179000
C	-3.94801400	1.84985300	-1.99588200
H	-2.54946600	3.17063600	-2.96188900
H	-4.76136600	2.56255000	-2.13635300
C	0.13623700	-0.68446100	-0.36128600
Fe	0.02652900	0.50521100	1.12446900
C	2.05872800	-1.97012900	0.40366600
C	3.37244600	-1.59555100	0.13925800
C	1.74156700	-2.60046100	1.62781800
C	4.41825200	-1.81790200	1.04767500
H	3.56087800	-1.08052000	-0.79737200
C	2.76412700	-2.80819300	2.55801000
C	4.07779300	-2.41916800	2.26436500
H	2.55392000	-3.27726300	3.51860500
H	4.83984200	-2.59424300	3.02357800
O	-0.33198200	1.59761400	-2.73679200
O	0.44158600	-2.94312700	1.82045300
C	-0.10728900	2.74184100	-3.53849800
H	-0.66226100	2.67215700	-4.49122900
H	-0.38645800	3.66827100	-3.01774700
H	0.97148100	2.76787700	-3.73982100
C	0.07458100	-3.61248200	3.00676200
H	0.55258300	-4.60562300	3.07351500
H	0.33814600	-3.02730000	3.90222200
H	-1.01636200	-3.72846700	2.97186100
C	0.24326100	1.46676900	3.41725600
C	-0.71896900	2.32291700	4.00619800
C	0.23252100	0.09862800	3.78954200
C	-1.67110700	1.82994500	4.88654100
H	-0.71537200	3.38025200	3.73390700
C	-0.74094400	-0.38558900	4.67485800
H	1.01669800	-0.57363300	3.43693600
C	-1.69568600	0.46610300	5.22094800
H	-2.41098200	2.50750000	5.32000500

H	-0.74547500	-1.44323500	4.94699900
H	-2.45855000	0.07883300	5.89901100
C	-0.76160400	4.20949700	-0.18068300
C	0.59622800	4.29849600	-0.58864600
C	1.04752700	5.30629800	-1.43929500
C	0.12200500	6.26490500	-1.87549900
C	-1.22210100	6.19946900	-1.47628200
C	-1.67185200	5.17329900	-0.63727300
C	-0.88502100	2.99882000	0.59440100
C	0.41477300	2.34314000	0.63266200
H	2.08301700	5.32952600	-1.77906300
H	0.45275300	7.06557900	-2.54128100
H	-1.92770700	6.95402200	-1.83258700
H	-2.72534600	5.11377100	-0.35434100
N	1.28660100	3.19321600	-0.07619700
C	2.61875800	2.89001800	-0.46337800
C	3.69944800	3.70105000	-0.05116000
C	2.83117100	1.77010600	-1.27951700
C	4.98378900	3.34229000	-0.48987400
C	4.11822600	1.43479700	-1.69485100
H	1.96280100	1.18153600	-1.57307700
C	5.19970400	2.22663100	-1.29895100
H	5.83832000	3.94905100	-0.18116200
H	4.27340300	0.57011200	-2.34074200
H	6.21107300	1.97938300	-1.62852900
C	-2.04248700	2.35357200	1.05709400
H	-3.01805200	2.85411200	0.99439300
N	-1.93690700	1.10476700	1.47818000
C	-2.99072100	0.39143700	2.02326500
C	-4.12617500	0.98221200	2.62091900
C	-2.93066200	-1.01345600	1.98689100
C	-5.16539200	0.19975000	3.10981300
H	-4.18189300	2.06724400	2.71656400
C	-3.97279900	-1.80522400	2.46825700
H	-2.05028400	-1.48050100	1.54270900
C	-5.10955300	-1.20037100	3.02335400
H	-6.04788100	0.65379400	3.56518000
H	-3.89743700	-2.88890900	2.38106500
O	-6.20192500	-1.88025700	3.48003200
C	-6.20472400	-3.28256300	3.38554900
H	-7.16468700	-3.62607000	3.79408700
H	-6.11998600	-3.62264100	2.33698900
H	-5.38371900	-3.73666800	3.97104600
C	3.52277300	4.88763100	0.88790500

C	4.09343000	6.18708500	0.30094700
C	4.14558900	4.60432200	2.26538900
H	2.44447900	5.03558200	1.03663200
H	3.65192900	6.41865600	-0.67858600
H	3.88406600	7.03431400	0.97298200
H	5.18671600	6.12728300	0.17520600
H	3.71161000	3.70900500	2.73080700
H	5.23355500	4.44643700	2.18616500
H	3.97893600	5.45587100	2.94443100
C	1.18110700	1.98927800	2.39336000
C	-5.51698500	0.22905500	-0.73319600
H	-5.39534400	0.35795100	0.35512600
C	-5.87874500	-1.26297800	-0.96461200
C	-6.72712700	1.08938200	-1.16227200
H	-5.03362500	-1.90037700	-0.67010600
C	-6.21151100	-1.50158100	-2.44601500
C	-7.09516200	-1.63220200	-0.09899700
H	-6.49699500	2.15520100	-1.00315100
C	-7.07524100	0.84333500	-2.64260800
C	-7.94016900	0.71469600	-0.29032700
H	-6.43424800	-2.56958700	-2.60915900
H	-5.33844100	-1.25709300	-3.07246900
C	-7.42100400	-0.64093000	-2.85241800
H	-6.86230900	-1.48695300	0.96662200
H	-7.33535100	-2.70131700	-0.23673700
C	-8.30045000	-0.76639400	-0.49823300
H	-6.23227100	1.12407600	-3.29261100
H	-7.93111900	1.47653300	-2.93360800
H	-7.70948900	0.89989900	0.77207700
H	-8.80032000	1.35423300	-0.55297000
H	-7.66148600	-0.81660900	-3.91473200
C	-8.63430200	-1.01107000	-1.98041000
H	-9.17058700	-1.02663000	0.12788000
H	-9.51032000	-0.40884800	-2.27737600
H	-8.90418600	-2.07000400	-2.13625700
C	5.82009800	-1.33384100	0.70011400
H	5.78750100	-0.23076700	0.77357500
C	6.23703000	-1.67228200	-0.75731200
C	6.93999300	-1.82971900	1.64521400
H	5.45701000	-1.33945500	-1.45581500
C	6.44073500	-3.18822900	-0.91117300
C	7.54688700	-0.94089200	-1.09363500
H	6.67160700	-1.59948700	2.68874500
C	7.16186700	-3.34661800	1.48255200

C	8.24951300	-1.09536800	1.30263400
H	6.69989800	-3.42196500	-1.95765500
H	5.50192300	-3.72050900	-0.68658900
C	7.56205500	-3.66052900	0.03096000
H	7.40163000	0.14754000	-1.00109800
H	7.82679900	-1.14246800	-2.14175200
C	8.66363800	-1.40577900	-0.14598000
H	6.25176300	-3.90691200	1.74539700
H	7.95417500	-3.67923900	2.17465300
H	8.11695100	-0.00809700	1.43342800
H	9.04473600	-1.40925200	1.99981800
H	7.70994900	-4.74745600	-0.08356300
C	8.86863700	-2.92213700	-0.30979300
H	9.60072900	-0.87563200	-0.38500600
H	9.68151800	-3.26878700	0.35101100
H	9.17569600	-3.15156600	-1.34463400
C	2.52910700	1.27751300	2.32499300
H	3.20560600	1.74507600	1.60483000
H	3.01467300	1.29079300	3.31632000
H	2.44189200	0.22250300	2.02672600
H	1.30704500	3.06830500	2.52863900

TS10(S_a,S)

C	0.86552800	-3.22370000	0.09060200
C	-0.54224100	-3.26343400	-0.55617900
H	0.89579800	-3.84735300	0.99470000
H	-0.51871600	-3.80574400	-1.51102500
N	-0.77761100	-1.82163100	-0.81428600
N	0.96488700	-1.79314700	0.47687000
C	-1.66604200	-3.78582300	0.31384700
C	-2.66364200	-4.59425000	-0.24078500
C	-1.80068300	-3.34616300	1.64149500
C	-3.78470600	-4.95996300	0.51048500
H	-2.57322700	-4.92245000	-1.27950200
C	-2.91956300	-3.71145100	2.39073800
H	-1.04630500	-2.69063500	2.08235500
C	-3.91758200	-4.51466100	1.82675500
H	-4.56172100	-5.58142300	0.06056800
H	-3.01739600	-3.36224900	3.42169100
H	-4.79812300	-4.78917900	2.41217600
C	1.99680000	-3.61881600	-0.83422300
C	2.87327500	-4.64938400	-0.48211200
C	2.21369400	-2.91144700	-2.02876500
C	3.95696300	-4.97456700	-1.30435100

H	2.71709200	-5.19303700	0.45335100
C	3.29948200	-3.23012900	-2.84343800
H	1.54020200	-2.10020700	-2.31114700
C	4.17582300	-4.26131700	-2.48371600
H	4.63838100	-5.77682800	-1.01306100
H	3.47063400	-2.66374700	-3.76154100
H	5.02950600	-4.50331600	-3.12101700
C	-1.89805000	-1.39035800	-1.57234800
C	-1.78036200	-1.31073800	-2.97441200
C	-3.10777500	-1.10316300	-0.95097900
C	-2.88272900	-0.87142700	-3.71020000
C	-4.23942700	-0.69841000	-1.67500100
H	-3.15588200	-1.19597500	0.13099700
C	-4.08462400	-0.56297900	-3.05924200
H	-2.82012600	-0.75879600	-4.79207800
H	-4.91441300	-0.21159900	-3.67131300
C	0.03633800	-1.01923300	-0.10868800
Fe	-0.11116300	1.00803000	-0.02250000
C	1.98552600	-1.31782800	1.34242500
C	3.30483900	-1.24566300	0.90067200
C	1.65917200	-0.94691700	2.66326200
C	4.35416500	-0.84934000	1.74239500
H	3.49706700	-1.49266400	-0.13969300
C	2.69063500	-0.53833500	3.51287300
C	4.01185300	-0.50587800	3.05582600
H	2.47601500	-0.23461000	4.53656600
H	4.78130000	-0.17743100	3.75450000
O	-0.57697300	-1.65984700	-3.49916200
O	0.34630600	-0.99659000	3.01032100
C	-0.41690200	-1.65877500	-4.90072200
H	-1.13226300	-2.34292300	-5.38975800
H	-0.54006700	-0.64758700	-5.32352700
H	0.60600500	-2.00644500	-5.09530300
C	-0.04669800	-0.48097700	4.26934100
H	0.36304100	-1.08442800	5.09774100
H	0.27162500	0.56765300	4.38591100
H	-1.14282100	-0.52110300	4.29074900
C	0.54122400	3.53213200	2.30169500
C	0.93926400	4.87580900	2.13832200
C	-0.59767100	3.29141600	3.09478200
C	0.23889500	5.91822500	2.74394900
H	1.79408000	5.10944900	1.50125900
C	-1.30488400	4.33085300	3.69831600
H	-0.95083500	2.26646100	3.21656400

C	-0.88854500	5.65560900	3.52976900
H	0.56925900	6.94870500	2.58827400
H	-2.19032400	4.10246000	4.29726800
H	-1.44044700	6.47421300	3.99834700
C	0.31735800	4.86908700	-1.21278000
C	1.62821200	4.50389800	-1.63584200
C	2.44324600	5.37234700	-2.35628400
C	1.94381400	6.65075100	-2.65037700
C	0.66289200	7.03526200	-2.23076600
C	-0.15517900	6.15092000	-1.51527100
C	-0.23805200	3.71212800	-0.53222200
C	0.80059900	2.68265200	-0.48330500
H	3.43862400	5.06386800	-2.68213500
H	2.56471700	7.35317800	-3.21179100
H	0.29648500	8.03727800	-2.46733400
H	-1.15430600	6.45855700	-1.19737900
N	1.87638200	3.17814800	-1.23140700
C	2.86523600	2.32808700	-1.81599600
C	2.56833400	1.45286800	-2.89217400
C	4.16345300	2.36017000	-1.28812700
C	3.57083900	0.54142100	-3.27079900
C	5.15314300	1.47933000	-1.72144600
H	4.38043000	3.08592100	-0.50671200
C	4.83947500	0.52781800	-2.69125400
H	3.35004100	-0.15791600	-4.08190700
H	6.15524400	1.52912000	-1.29137600
H	5.58532200	-0.20003100	-3.01905400
C	-1.48556100	3.43620300	0.01177200
H	-2.24183800	4.22184500	0.11539700
N	-1.71718300	2.18253000	0.46571100
C	-2.81376500	1.82637300	1.23113600
C	-3.97839700	2.61184800	1.40732700
C	-2.76989700	0.59546900	1.91988100
C	-5.02087700	2.18201600	2.22142300
H	-4.07048900	3.57266300	0.90034300
C	-3.81373900	0.15647700	2.73523800
H	-1.87516800	-0.02331500	1.82071900
C	-4.95239500	0.95556000	2.89971400
H	-5.91599500	2.79389300	2.35214200
H	-3.72602000	-0.81117600	3.22892600
O	-6.03124400	0.62004200	3.67044900
C	-5.98417400	-0.58496200	4.39055200
H	-6.92930900	-0.66134900	4.94501400
H	-5.89105400	-1.46156200	3.72330900

H	-5.14395500	-0.60049900	5.10976500
C	1.35649300	1.53505900	-3.82181400
C	1.55967700	2.70048100	-4.80946100
C	-0.04920000	1.59280200	-3.22416300
H	1.40356300	0.60466300	-4.41213300
H	2.53055700	2.62545500	-5.32297900
H	0.76363700	2.69913500	-5.57211200
H	1.52504800	3.66706900	-4.28566100
H	-0.19595300	0.81453500	-2.46288400
H	-0.28092200	2.56345600	-2.77255700
H	-0.79084800	1.41368300	-4.01892700
C	1.23683800	2.43189000	1.61650300
H	0.86859600	1.46119700	1.98591200
C	2.74402900	2.45780800	1.62733100
H	3.13242600	3.37279300	1.15838000
H	3.13851000	2.42490300	2.65906600
H	3.16002100	1.59951700	1.08552800
C	-5.53077200	-0.42183000	-0.91847100
H	-5.32659600	0.45066300	-0.27250800
C	-5.93042400	-1.59288500	0.02436900
C	-6.75682600	-0.07858200	-1.79662100
H	-5.07154400	-1.86635000	0.65458800
C	-6.37169900	-2.81225700	-0.80147900
C	-7.08942300	-1.14445600	0.92970900
H	-6.49900800	0.74657200	-2.48081900
C	-7.22968700	-1.30402000	-2.60653600
C	-7.90874700	0.37676900	-0.88019900
H	-6.61029200	-3.64942000	-0.12377500
H	-5.54654200	-3.15207800	-1.44857200
C	-7.60313300	-2.44922800	-1.65145000
H	-6.78410800	-0.29111900	1.54934500
H	-7.35679300	-1.96517100	1.61837500
C	-8.30638400	-0.76433500	0.07324200
H	-6.45141400	-1.64786200	-3.30327300
H	-8.10324900	-1.02199900	-3.21900200
H	-7.59868900	1.26065600	-0.29847400
H	-8.77516100	0.67824700	-1.49320100
H	-7.92126200	-3.32804900	-2.23757300
C	-8.75394200	-1.99109600	-0.73921700
H	-9.12912000	-0.42815300	0.72620400
H	-9.64109800	-1.74513000	-1.34788100
H	-9.04782300	-2.80748400	-0.05701500
C	5.77114200	-0.72440400	1.19406000
H	5.81235000	0.25817300	0.69139700

C	6.12078000	-1.79580800	0.12707400
C	6.88383200	-0.73785000	2.27013700
H	5.35418300	-1.80667300	-0.66051800
C	6.20696200	-3.18316900	0.78561500
C	7.47334000	-1.45561200	-0.52070600
H	6.67072100	0.02493600	3.03494500
C	6.97700600	-2.12748600	2.92690500
C	8.23314300	-0.39722300	1.61194400
H	6.42260400	-3.94349300	0.01718700
H	5.23677700	-3.45184900	1.23238200
C	7.31090500	-3.18214600	1.85747900
H	7.41934300	-0.47221900	-1.01612700
H	7.70360800	-2.19724900	-1.30463000
C	8.57938200	-1.44913300	0.54572300
H	6.02821900	-2.38394300	3.42394100
H	7.75692400	-2.11833500	3.70729600
H	8.18641500	0.60545000	1.15392900
H	9.02265400	-0.36218500	2.38159500
H	7.37083100	-4.17800100	2.32792900
C	8.66120900	-2.83821700	1.20346300
H	9.54721500	-1.20068900	0.07892500
H	9.46336700	-2.85158200	1.96109000
H	8.92046400	-3.59895500	0.44720400

TS10(S_a,R)

C	1.05518900	-3.12457100	-0.73282000
C	-0.29387300	-2.96647500	-1.47022900
H	1.04527200	-4.00544900	-0.07634500
H	-0.17921400	-3.16021800	-2.54483800
N	-0.56521000	-1.51805200	-1.26522500
N	1.07321400	-1.89421100	0.10203900
C	-1.45806800	-3.76218100	-0.92063800
C	-2.37047000	-4.36093400	-1.79546900
C	-1.71593700	-3.78608400	0.45974500
C	-3.52733800	-4.97532800	-1.30694200
H	-2.18453900	-4.32591900	-2.87210200
C	-2.87176600	-4.39697300	0.94692700
H	-1.02713000	-3.29926100	1.15340300
C	-3.78214000	-4.99061400	0.06546600
H	-4.23812000	-5.42807700	-2.00166100
H	-3.07220900	-4.39845100	2.02101600
H	-4.69324400	-5.45539100	0.44894500
C	2.27322400	-3.17058900	-1.62923800
C	3.26340900	-4.13664900	-1.42552200

C	2.46323000	-2.19058000	-2.61847600
C	4.43240900	-4.13131700	-2.19275800
H	3.12557400	-4.89219200	-0.64771000
C	3.63251200	-2.18222800	-3.37860500
H	1.70029000	-1.42662200	-2.78443600
C	4.62192100	-3.14991400	-3.16682300
H	5.20008700	-4.88820200	-2.01880500
H	3.77724500	-1.41138100	-4.13828100
H	5.53891800	-3.13588000	-3.76047000
C	-1.70946300	-0.93234400	-1.86593900
C	-1.66155000	-0.57542600	-3.22738500
C	-2.89169000	-0.80931600	-1.14926500
C	-2.80260200	-0.01795000	-3.80782000
C	-4.06584000	-0.30682400	-1.72837000
H	-2.89355600	-1.12938300	-0.10926900
C	-3.97972400	0.12068700	-3.05751200
H	-2.79125400	0.31488700	-4.84530300
H	-4.84309200	0.56863600	-3.54764200
C	0.16911100	-0.97945400	-0.27858900
Fe	-0.01521800	0.99832000	0.31420600
C	2.03585200	-1.70715800	1.12618700
C	3.33900200	-1.34016400	0.80595400
C	1.65738100	-1.90305900	2.47017400
C	4.32588700	-1.16736900	1.78704500
H	3.56219200	-1.15579200	-0.24271800
C	2.62779500	-1.73597300	3.46227800
C	3.93619700	-1.37878800	3.11515300
H	2.37454400	-1.86602200	4.51372100
H	4.65415100	-1.24448900	3.92399500
O	-0.48418500	-0.80589500	-3.86910600
O	0.35718700	-2.21943300	2.69944800
C	-0.40914800	-0.58118500	-5.25979100
H	-1.16096900	-1.17749400	-5.80586300
H	-0.54656000	0.48442500	-5.50888800
H	0.59654700	-0.89323800	-5.57034500
C	-0.10017600	-2.32605100	4.03389300
H	0.39100400	-3.16074300	4.56315300
H	0.06730800	-1.39038200	4.59121700
H	-1.17944000	-2.51411700	3.97607500
C	0.31400800	2.67773200	2.96914500
C	-0.47796200	3.83361000	3.17940300
C	-0.00318100	1.53285600	3.74450700
C	-1.54363900	3.82741800	4.08046400
H	-0.23518300	4.74408000	2.62913300

C	-1.06257900	1.53575600	4.64456000
H	0.58635900	0.62377800	3.61882500
C	-1.85066000	2.68076700	4.81750700
H	-2.14075700	4.73438200	4.21180500
H	-1.29292500	0.63061000	5.21308600
H	-2.69334800	2.67359200	5.51192800
C	-0.19556200	4.98324800	-0.55439400
C	1.12934500	4.85355600	-1.06627200
C	1.77927600	5.90059400	-1.71746300
C	1.09179800	7.11527400	-1.85275200
C	-0.20888300	7.26747000	-1.34979000
C	-0.85792900	6.20821900	-0.70316700
C	-0.54567700	3.69212500	0.01154600
C	0.62041600	2.82505600	-0.09315500
H	2.79298000	5.77540500	-2.10326000
H	1.57960000	7.95437100	-2.35489600
H	-0.72278000	8.22477400	-1.46626100
H	-1.87455400	6.33545700	-0.32251000
N	1.56728900	3.54718700	-0.82492200
C	2.73534400	2.95274700	-1.37971800
C	2.70131800	2.23808200	-2.59845500
C	3.92897900	3.04977300	-0.65017200
C	3.87616300	1.56156600	-2.97444700
C	5.08521800	2.38864400	-1.05994300
H	3.91983200	3.64195400	0.26549700
C	5.04782100	1.61317100	-2.22065000
H	3.86691700	0.98933900	-3.90599900
H	6.00111800	2.46831600	-0.47047100
H	5.93299800	1.06336400	-2.54830600
C	-1.71631100	3.19298800	0.57332100
H	-2.56780100	3.85436700	0.76340500
N	-1.76721200	1.88623700	0.90018100
C	-2.79754800	1.33061600	1.64429100
C	-4.00615000	1.97966900	1.96289200
C	-2.62955900	0.01471500	2.13843800
C	-4.98667000	1.36236100	2.74357900
H	-4.19233300	2.99396700	1.61076900
C	-3.60389500	-0.60842900	2.90411700
H	-1.69531600	-0.50940900	1.92523900
C	-4.79355800	0.06186000	3.22621300
H	-5.90244000	1.91064100	2.96474200
H	-3.46696700	-1.62655000	3.27490100
O	-5.70118600	-0.63284900	3.97640200
C	-6.91329800	-0.00442900	4.30991800

H	-7.48762400	-0.72163800	4.91198200
H	-6.75170800	0.91420100	4.90353500
H	-7.50209000	0.25764400	3.41118500
C	1.56409300	2.24853400	-3.61893200
C	1.79748000	3.40531500	-4.61100500
C	0.12169800	2.28246700	-3.11182200
H	1.68173400	1.30714000	-4.18364500
H	1.69293000	4.37487300	-4.10050000
H	2.80270700	3.36041300	-5.05752300
H	1.05486800	3.37022700	-5.42501200
H	-0.07240500	1.51802300	-2.35077600
H	-0.14932600	3.25474000	-2.68469200
H	-0.56172900	2.09967800	-3.95470500
C	1.36644500	2.68713300	1.95811800
C	-5.31570600	-0.29711600	-0.86574800
H	-5.04782600	0.27318600	0.03902000
C	-5.69890600	-1.72907400	-0.38869700
C	-6.57796100	0.36380300	-1.46271400
H	-4.80478300	-2.22982900	0.00996300
C	-6.26803500	-2.54894800	-1.55735600
C	-6.75475300	-1.62380800	0.72238400
H	-6.32310700	1.37159500	-1.83139900
C	-7.19070700	-0.47345200	-2.60508800
C	-7.62509500	0.48396600	-0.33643100
H	-6.48936500	-3.57393100	-1.21452500
H	-5.51822700	-2.63725400	-2.36093300
C	-7.54642100	-1.87779100	-2.09239400
H	-6.34091600	-1.08321800	1.58285800
H	-7.01902900	-2.63414100	1.08001800
C	-8.00732600	-0.91620100	0.18184400
H	-6.50214300	-0.56112300	-3.45716400
H	-8.09631900	0.03305000	-2.98095700
H	-7.21748200	1.09169000	0.48811500
H	-8.52250400	1.00388300	-0.71315000
H	-7.96223400	-2.47724100	-2.91996300
C	-8.59044700	-1.75442200	-0.96873600
H	-8.75584200	-0.81728200	0.98674300
H	-9.50871100	-1.28376700	-1.36037000
H	-8.87332900	-2.75502700	-0.59889200
C	5.71133400	-0.67622500	1.38475900
H	5.59736500	0.40478200	1.18177600
C	6.24195000	-1.32877100	0.07961300
C	6.79831000	-0.83119900	2.47506400
H	5.49447000	-1.23106400	-0.72073300

C	6.54521400	-2.81658300	0.32331800
C	7.52785700	-0.61246100	-0.36577800
H	6.45297700	-0.36486800	3.41116800
C	7.11435600	-2.32011400	2.71438900
C	8.08177000	-0.11294800	2.01913000
H	6.88779700	-3.28190900	-0.61561300
H	5.62647200	-3.34551000	0.62271500
C	7.62521200	-2.95672600	1.41050000
H	7.31852400	0.45163000	-0.55987300
H	7.88435800	-1.04889700	-1.31452500
C	8.60655000	-0.74596900	0.71992600
H	6.21965800	-2.85705000	3.06566100
H	7.87661900	-2.41399500	3.50649800
H	7.87758300	0.95981400	1.86211800
H	8.84632200	-0.18183000	2.81129600
H	7.84145800	-4.02459600	1.58209300
C	8.90769300	-2.23616600	0.95816800
H	9.52531300	-0.22707300	0.39914600
H	9.69397700	-2.34626300	1.72445900
H	9.29442900	-2.69546700	0.03225200
C	2.49612900	1.69204100	2.07438900
H	3.05393500	1.58923500	1.13625800
H	3.22206900	1.98761400	2.85754800
H	2.14260100	0.69156000	2.34087300
H	1.71972200	3.70235300	1.75303300

1a

C	0.82750700	1.87107300	-0.40305200
C	2.14465300	1.35409900	-0.54952100
C	3.27510800	2.17518900	-0.61751700
C	3.07198500	3.55033900	-0.52975300
C	1.77531900	4.08795900	-0.38207300
C	0.65452400	3.26441400	-0.32032700
C	-0.06783500	0.73222700	-0.37473700
C	0.72838800	-0.39597100	-0.49967200
H	4.27257300	1.74942100	-0.73910100
H	3.93026600	4.22434700	-0.57893300
H	1.65185100	5.17149600	-0.31756600
H	-0.34269200	3.69644900	-0.21008300
H	0.43022800	-1.44125000	-0.51120900
N	2.05010800	-0.03269400	-0.60573600
C	3.15872800	-0.92774100	-0.69351500
C	3.45414300	-1.50650600	-1.93369400
C	3.93394300	-1.20454200	0.44854200

C	4.53285100	-2.37992700	-2.05999800
H	2.82774200	-1.25553900	-2.79178500
C	5.02019400	-2.08007600	0.29155800
C	5.32008600	-2.66241100	-0.94002500
H	4.76129800	-2.83228900	-3.02739800
H	5.64111500	-2.31674300	1.15866200
H	6.17162400	-3.34152900	-1.02606000
C	3.60153600	-0.61429000	1.80968000
C	4.75781600	0.23056400	2.36321500
C	3.18231100	-1.71504200	2.79658800
H	2.73938600	0.05510100	1.68096300
H	5.02469200	1.04156400	1.66930900
H	4.47342700	0.68726500	3.32429600
H	5.65869500	-0.37985200	2.53770600
H	2.33022000	-2.29344200	2.40776200
H	4.00785400	-2.41940400	2.98916300
H	2.88458500	-1.27498500	3.76156300
C	-1.50989000	0.74834000	-0.23966100
H	-1.97026400	1.74907300	-0.12567800
N	-2.21954400	-0.32060100	-0.26159600
C	-3.60131000	-0.27491700	-0.05833300
C	-4.39501500	-1.24317900	-0.69383600
C	-4.24877800	0.66120500	0.77868200
C	-5.78331300	-1.26220600	-0.54987400
H	-3.89463900	-1.98269300	-1.32203700
C	-5.62800400	0.64091800	0.94072800
H	-3.65645700	1.39228800	1.33287500
C	-6.41214300	-0.31371900	0.27100400
H	-6.36160100	-2.02327000	-1.07333300
H	-6.13138500	1.35279700	1.59791000
O	-7.75175100	-0.25155600	0.49031400
C	-8.58696200	-1.19434500	-0.13684800
H	-9.61414300	-0.96270200	0.17529400
H	-8.52512700	-1.13107200	-1.23870700
H	-8.34423300	-2.22824200	0.16984100

2a

C	0.40901700	-1.28472500	0.00001300
C	-0.51601200	-0.22482600	-0.00001000
C	-0.01336600	1.09119000	0.00003300
C	1.35845900	1.33258500	0.00000600
C	2.26548700	0.26589800	-0.00002400
C	1.78436800	-1.04500600	-0.00000700
H	0.03742300	-2.31303700	0.00003600

H	-0.70481300	1.93608400	0.00010100
H	1.72654000	2.36156200	0.00001200
H	3.34089900	0.45862400	-0.00004800
H	2.48247300	-1.88566800	-0.00000700
C	-1.95546700	-0.53291200	0.00000200
C	-2.97528100	0.33741000	-0.00002900
H	-2.19097100	-1.60347100	0.00007700
H	-4.01028100	-0.01206500	0.00001800
H	-2.82450500	1.42028800	-0.00009100

product

C	-0.57126200	-2.16673900	-0.93210700
C	-1.93867500	-1.80086900	-0.96253300
C	-2.93361100	-2.61803100	-1.50874800
C	-2.53247500	-3.84204200	-2.03993500
C	-1.17627300	-4.22930300	-2.02696400
C	-0.19411800	-3.40432100	-1.48383200
C	0.15057900	-1.07615200	-0.29611000
C	-0.80292600	-0.10913700	0.05775600
H	-3.97792900	-2.30167500	-1.51859300
H	-3.27867400	-4.51022500	-2.47583300
H	-0.89252500	-5.19423300	-2.45342300
H	0.85091100	-3.72154100	-1.48864700
C	-0.74548000	1.21017400	0.79320000
H	-1.75588700	1.35244800	1.20276800
C	0.21365200	1.22230800	1.99231000
H	0.11444300	2.17870100	2.52763900
H	-0.03184400	0.40487100	2.68898900
H	1.25259700	1.09772500	1.66580100
N	-2.04816400	-0.55475500	-0.36275700
C	-0.52438900	2.38072700	-0.16034500
C	-1.50395500	3.37592800	-0.28190300
C	0.64975600	2.48887000	-0.92143100
C	-1.32176500	4.45784600	-1.14820600
H	-2.42369200	3.29858900	0.30338500
C	0.83098900	3.56880800	-1.78776500
H	1.42094900	1.72310300	-0.81598900
C	-0.15309100	4.55586300	-1.90676800
H	-2.09659600	5.22442400	-1.23036600
H	1.75055600	3.64067600	-2.37427300
H	-0.00797300	5.39906300	-2.58672900
C	-3.29951100	0.10049600	-0.14987400
C	-4.04981200	-0.17703400	1.00767800
C	-3.75845000	0.99702400	-1.12100600

C	-5.27877600	0.48533000	1.15349300
C	-4.98243600	1.64187200	-0.95301200
H	-3.13570800	1.18573100	-1.99633400
C	-5.74298900	1.38266300	0.19100900
H	-5.88497000	0.29384800	2.04191300
H	-5.33832000	2.34514200	-1.70880300
H	-6.70385200	1.88253800	0.33474800
C	-3.55228400	-1.14684400	2.06683400
C	-4.46331200	-2.37959700	2.16455300
C	-3.38270400	-0.45570700	3.42799800
H	-2.55951300	-1.49947800	1.75243400
H	-4.53584700	-2.89348500	1.19428800
H	-4.06515700	-3.09627100	2.90025600
H	-5.48194900	-2.10290900	2.48192300
H	-2.70674200	0.41022200	3.35520700
H	-4.34672200	-0.09781900	3.82401200
H	-2.95897100	-1.15610600	4.16496200
C	1.59261500	-1.11565100	-0.16677500
H	2.02352600	-2.11422300	-0.37041400
N	2.36335100	-0.13180100	0.12704200
C	3.75486700	-0.28021100	0.14153100
C	4.49691400	0.52802300	1.01695700
C	4.46473600	-1.15919700	-0.70608100
C	5.88832500	0.44108000	1.09116900
H	3.95818700	1.22927700	1.65717000
C	5.84972000	-1.24264300	-0.64945100
H	3.92391000	-1.75990100	-1.44009000
C	6.57708600	-0.45168600	0.25637800
H	6.42316100	1.07831800	1.79498700
H	6.40389300	-1.90987500	-1.31249700
O	7.92640000	-0.60612000	0.23960000
C	8.71058500	0.17452000	1.10929000
H	8.46450700	-0.01744600	2.16964200
H	9.75649900	-0.10838500	0.93018400
H	8.59303600	1.25553500	0.91103700

TS-S1

C	-0.93947000	-2.04126400	2.74022500
C	0.49709200	-1.48050000	2.83681600
H	-0.93880800	-3.13736900	2.77630700
H	0.63425300	-0.91249700	3.76780900
N	0.51548200	-0.53437500	1.69311100
N	-1.34241900	-1.59754100	1.37619600
C	1.58364800	0.38098800	1.50834900

C	1.43990300	1.69651100	1.99107300
C	2.74852000	-0.01013400	0.86179100
C	2.47070600	2.60875000	1.75219800
C	3.79302900	0.89159000	0.61324700
H	2.80177000	-1.03856600	0.51192700
C	3.61583700	2.20726200	1.05678200
H	2.38115800	3.64341100	2.07866500
H	4.37350200	2.96223900	0.84953000
C	-0.52223100	-0.66821000	0.85364200
Fe	-0.54984300	0.32333600	-0.97003000
C	-2.64028500	-1.90612600	0.90794500
C	-3.60830500	-0.91642500	0.78382500
C	-2.96711300	-3.25167600	0.64241000
C	-4.92701900	-1.20580600	0.39506300
H	-3.31334500	0.10619800	1.02150800
C	-4.28245500	-3.56518800	0.29400900
C	-5.24320900	-2.55137300	0.17960400
H	-4.57160400	-4.59569600	0.09085700
H	-6.25909000	-2.84072600	-0.08580500
O	0.28787100	1.97734600	2.64717100
O	-1.95342000	-4.14900300	0.75621300
C	0.11820400	3.26000500	3.21505000
H	0.90071500	3.47234600	3.96469500
H	0.12218500	4.04483800	2.44523000
H	-0.86417200	3.25462600	3.70336100
C	-2.17736900	-5.48672900	0.36523100
H	-2.94042700	-5.97492200	0.99656700
H	-2.48835400	-5.54504300	-0.69137900
H	-1.22046900	-6.00968300	0.48961500
C	-1.82930300	1.43257900	-3.39280800
C	-2.04536100	0.25562300	-2.43916100
H	-2.51151300	1.39112500	-4.26517700
H	-0.80254800	1.47740100	-3.79058400
H	-2.99058700	0.37652000	-1.89014300
C	-2.00829200	-1.08516000	-3.05999300
C	-1.13619200	-1.40221200	-4.12810700
C	-2.82599800	-2.13421400	-2.57933500
C	-1.06465300	-2.69172400	-4.65325600
H	-0.48592800	-0.62579300	-4.53451600
C	-2.75607100	-3.42286800	-3.10708700
H	-3.52900800	-1.92256100	-1.77639100
C	-1.86867800	-3.71892800	-4.14730300
H	-0.36439900	-2.90107800	-5.46668300
H	-3.41446900	-4.19949200	-2.70654400

H	-1.80718600	-4.72844100	-4.56028900
C	2.58324000	2.82439400	-2.17224900
C	1.94089400	3.92867300	-1.52955900
C	2.65427000	5.13750300	-1.49123500
C	3.91227000	5.23960000	-2.09460300
C	4.50860800	4.15663600	-2.75047200
C	3.83699500	2.93995900	-2.78559300
C	1.75350400	1.67693800	-1.95324200
C	0.63838300	2.05166800	-1.20621200
H	2.30306400	6.02071400	-0.98986600
H	4.43345100	6.19825300	-2.03972200
H	5.49078400	4.26354100	-3.21647900
H	4.28588400	2.06942400	-3.26942000
N	0.69265500	3.43390000	-0.98682200
C	-0.43504100	4.03504900	-0.28540900
C	-0.59191900	5.34298200	0.26823400
C	-1.54276700	3.16632500	-0.10654600
C	-1.70873100	5.59290900	1.09371900
C	-2.63956800	3.44723300	0.69239600
H	-1.54673400	2.22696700	-0.64584400
C	-2.71432600	4.67222400	1.34936800
H	-1.78775700	6.57962100	1.54871400
H	-3.43747900	2.70773000	0.77865900
H	-3.55277200	4.92411700	2.00205600
C	2.02145900	0.30781100	-2.24759500
H	2.94497100	0.05453500	-2.78954500
N	1.21988700	-0.63007600	-1.82712500
C	1.56985500	-1.98598400	-1.84925200
C	2.89490900	-2.44125000	-1.96122200
C	0.55553300	-2.94833900	-1.67643600
C	3.20570700	-3.80157800	-1.91461200
H	3.71399000	-1.72852900	-2.05151500
C	0.85554400	-4.30113800	-1.63809000
H	-0.47801600	-2.62367500	-1.57766000
C	2.18235900	-4.74315900	-1.74750600
H	4.25060900	-4.10447300	-1.97610300
H	0.06570300	-5.04237500	-1.51601400
O	2.38495200	-6.08655600	-1.62766900
C	3.69306400	-6.58575600	-1.77643900
H	3.62770000	-7.67783800	-1.68071700
H	4.11651300	-6.33547200	-2.76576500
H	4.37231800	-6.19809100	-0.99498800
H	-2.00305400	2.39399100	-2.88487300
C	0.31856000	6.54325200	0.05393900

C	1.42498100	6.59433500	1.12060200
C	-0.41419500	7.89355200	-0.01176900
H	0.74912100	6.42584400	-0.94148600
H	1.99450300	5.65589000	1.16480400
H	2.13545000	7.41510900	0.92849000
H	0.97529300	6.76322400	2.11251500
H	-1.27569100	7.85248100	-0.69512300
H	-0.77360800	8.23543200	0.97102300
H	0.27957200	8.66466100	-0.38274300
C	-1.91243300	-1.49854500	3.76302100
C	-2.73176400	-2.36700400	4.49014000
C	-2.05441700	-0.11245700	3.93858600
C	-3.68001500	-1.86602800	5.38784800
H	-2.63304500	-3.44594400	4.34400900
C	-3.00184400	0.38741000	4.83192400
H	-1.42450300	0.57384500	3.36787000
C	-3.81765500	-0.48740700	5.55922600
H	-4.31594800	-2.55504000	5.94884700
H	-3.10850100	1.46761100	4.96016700
H	-4.56103500	-0.09289200	6.25616000
C	1.61246000	-2.49216200	2.69295000
C	2.79965400	-2.32590400	3.41581800
C	1.51862900	-3.53732100	1.76149400
C	3.88082400	-3.18853700	3.21613200
H	2.88307200	-1.50217200	4.12942000
C	2.59445500	-4.40514600	1.56720800
H	0.61129300	-3.66298800	1.16828200
C	3.77891600	-4.23003600	2.29096100
H	4.80445600	-3.04282600	3.78048000
H	2.50674000	-5.21495500	0.84183200
H	4.62298600	-4.90582700	2.13251900
C	-5.91959300	-0.05649600	0.32136400
H	-5.90098500	0.40185900	1.32737500
C	-7.39397700	-0.42079700	0.03145200
C	-5.49429700	1.05742400	-0.67459200
H	-7.71067800	-1.23190300	0.70810700
C	-7.60590000	-0.84798500	-1.43563300
C	-8.25503500	0.82938600	0.30328000
H	-4.43229700	1.29887300	-0.51937800
C	-5.69413200	0.59652500	-2.12775700
C	-6.34740300	2.30809600	-0.40661300
H	-8.67013600	-1.09487600	-1.59107500
H	-7.03071000	-1.75375500	-1.67697500
C	-7.17929300	0.28900000	-2.37766000

H	-8.14644300	1.14000300	1.35634800
H	-9.32052200	0.59000800	0.14698200
C	-7.83279600	1.97668000	-0.63389100
H	-5.08529700	-0.29608600	-2.33497800
H	-5.33963800	1.38297900	-2.81413500
H	-6.18815800	2.66017500	0.62738800
H	-6.03325500	3.12633500	-1.07596300
H	-7.31941400	-0.02817100	-3.42418100
C	-8.02976000	1.53928700	-2.09597700
H	-8.44851100	2.86764600	-0.42521200
H	-7.73890100	2.35711400	-2.77698300
H	-9.09513100	1.32394600	-2.28712100
C	4.99205700	0.43258200	-0.20132800
H	4.62839400	0.33637600	-1.24080700
C	5.52687800	-0.96158900	0.22349600
C	6.18184100	1.41822300	-0.24517800
H	4.69903300	-1.68269000	0.27597000
C	6.20342000	-0.86734900	1.60072900
C	6.54578900	-1.45070800	-0.81872000
H	5.82158100	2.41191700	-0.55141000
C	6.87405700	1.49934400	1.12853000
C	7.19802800	0.92262700	-1.28987300
H	6.54694700	-1.86809200	1.91271400
H	5.47303300	-0.53136000	2.35405600
C	7.39133800	0.10794000	1.53169300
H	6.06338900	-1.54153300	-1.80749100
H	6.90572100	-2.45724900	-0.54457300
C	7.72562600	-0.46791100	-0.89603300
H	6.17728000	1.87293800	1.89442300
H	7.71122200	2.21647700	1.07862500
H	6.72285900	0.88150500	-2.28495000
H	8.03473200	1.63777800	-1.36407400
H	7.88028800	0.16751200	2.51865100
C	8.40443000	-0.38573700	0.48325400
H	8.45313500	-0.81630800	-1.64833100
H	9.26764100	0.30029000	0.43959900
H	8.79559500	-1.37690900	0.77075300

2. Atomic coordinates of neijd09

Atomic coordinates (x 10^4) and equivalent isotropic displacement parameters (Å^{2}x 10^3) for **neijd09**. U_{eq} is defined as one third of the trace of the orthogonalized U_{ij} tensor.

	x	y	z	U_{eq}
Fe1	2785(1)	3883(1)	1484(1)	38(1)
C1	2085(5)	3037(5)	411(4)	43(1)
C2	2478(4)	3934(6)	112(3)	44(1)
C3	3447(5)	3948(7)	-173(4)	49(1)
C4	4161(5)	3068(7)	-92(4)	57(2)
C5	5062(6)	3102(8)	-398(5)	66(2)
C6	5281(6)	3986(9)	-781(5)	73(2)
C7	4619(7)	4873(8)	-867(4)	73(3)
C8	3717(6)	4834(7)	-556(4)	59(2)
C9	1770(5)	4490(5)	2133(4)	42(1)
C10	1450(5)	5054(5)	1392(4)	42(1)
C11	422(5)	4890(5)	723(4)	43(1)
C12	-269(4)	4019(6)	703(4)	48(2)
C13	-1249(5)	3924(7)	76(4)	56(2)
C14	-1569(6)	4659(7)	-536(5)	58(2)
C15	-915(6)	5515(7)	-542(5)	61(2)
C16	83(6)	5636(6)	84(4)	53(2)
C17	4444(4)	4159(5)	1850(4)	45(1)
C18	3994(4)	5174(5)	1671(4)	42(1)
C19	3915(4)	5939(5)	2290(4)	45(1)
C20	4215(5)	5734(6)	3149(4)	51(2)
C21	4117(5)	6471(7)	3727(5)	60(2)
C22	3698(6)	7450(7)	3486(7)	72(2)
C23	3382(6)	7688(6)	2635(7)	73(2)
C24	3504(5)	6961(6)	2060(5)	57(2)
C25	2990(5)	2530(5)	2248(4)	44(1)
C26	3687(5)	1651(6)	2053(4)	49(1)
C27	3710(6)	656(6)	2585(5)	56(2)
C28	4084(7)	907(6)	3501(5)	60(2)
C29	3411(6)	1771(7)	3725(5)	60(2)
C30	3370(6)	2756(6)	3187(4)	51(2)
Cl1	9610(1)	7877(1)	4521(1)	49(1)
Mg1	8684(1)	6771(2)	3374(1)	34(1)

O1	7836(3)	5833(3)	2353(2)	38(1)
O2	10109(3)	5870(3)	3491(2)	38(1)
O3	9122(3)	7699(3)	2480(2)	42(1)
O4	7217(3)	7627(3)	3254(2)	42(1)
O5	8146(3)	5671(3)	4119(2)	38(1)
C31	6862(4)	6203(5)	1727(4)	45(1)
C32	6678(5)	5406(6)	1026(4)	54(2)
C33	7140(5)	4403(6)	1471(4)	51(2)
C34	8137(5)	4807(5)	2122(4)	45(1)
C35	10338(5)	4779(5)	3746(4)	47(1)
C36	11470(6)	4775(7)	4371(5)	63(2)
C37	11785(5)	5921(7)	4490(5)	62(2)
C38	11153(4)	6405(5)	3672(4)	45(1)
C39	9417(5)	7247(5)	1779(4)	45(1)
C40	10254(5)	7994(6)	1600(4)	51(2)
C41	10466(6)	8810(6)	2287(4)	58(2)
C42	9414(6)	8788(6)	2529(5)	62(2)
C43	6765(7)	8460(7)	2682(5)	68(2)
C44	6656(8)	9349(7)	3195(6)	74(2)
C45	6289(5)	8844(6)	3886(4)	49(1)
C46	6800(4)	7755(5)	3967(4)	44(1)
C47	8596(5)	5539(6)	5011(4)	48(2)
C48	7957(5)	4650(6)	5253(4)	54(2)
C49	6843(5)	4755(6)	4606(4)	54(2)
C50	7153(5)	5051(5)	3833(4)	45(1)
C51	2947(15)	7178(13)	6675(13)	169(7)
C52	2024(9)	7054(10)	7082(7)	94(3)
C53	2163(10)	6689(9)	7823(8)	103(3)
C54	1285(9)	6675(10)	8191(7)	95(3)
C55	309(9)	6963(17)	7746(8)	156(7)
C56	98(9)	6944(9)	6973(8)	99(3)
C57	1000(8)	6979(13)	6647(6)	118(5)

Bond lengths [Å] and angles [°] for **neijd09**.

Fe(1)-C(1)	2.062(6)
Fe(1)-C(2)	2.219(6)
Fe(1)-C(9)	2.074(5)
Fe(1)-C(10)	2.238(6)
Fe(1)-C(17)	2.068(5)
Fe(1)-C(18)	2.212(5)
Fe(1)-C(25)	2.108(6)
C(1)-H(1A)	1.05(7)
C(1)-H(1B)	1.12(8)
C(1)-C(2)	1.394(9)
C(2)-H(2)	1.03(8)
C(2)-C(3)	1.457(8)
C(3)-C(4)	1.424(11)
C(3)-C(8)	1.386(11)
C(4)-H(4)	0.9500
C(4)-C(5)	1.396(9)
C(5)-H(5)	0.9500
C(5)-C(6)	1.359(14)
C(6)-H(6)	0.9500
C(6)-C(7)	1.389(14)
C(7)-H(7)	0.9500
C(7)-C(8)	1.403(10)
C(8)-H(8)	0.9500
C(9)-H(9A)	1.14(7)
C(9)-H(9B)	1.04(6)
C(9)-C(10)	1.388(9)
C(10)-H(10)	0.95(9)
C(10)-C(11)	1.477(8)
C(11)-C(12)	1.409(9)
C(11)-C(16)	1.400(10)
C(12)-H(12)	0.9500
C(12)-C(13)	1.390(9)
C(13)-H(13)	0.9500
C(13)-C(14)	1.357(11)
C(14)-H(14)	0.9500
C(14)-C(15)	1.372(12)
C(15)-H(15)	0.9500

C(15)-C(16)	1.410(10)
C(16)-H(16)	0.9500
C(17)-H(17A)	0.96(7)
C(17)-H(17B)	1.10(8)
C(17)-C(18)	1.404(9)
C(18)-H(18)	0.91(9)
C(18)-C(19)	1.445(9)
C(19)-C(20)	1.404(10)
C(19)-C(24)	1.406(10)
C(20)-H(20)	0.9500
C(20)-C(21)	1.377(10)
C(21)-H(21)	0.9500
C(21)-C(22)	1.364(12)
C(22)-H(22)	0.9500
C(22)-C(23)	1.399(13)
C(23)-H(23)	0.9500
C(23)-C(24)	1.374(11)
C(24)-H(24)	0.9500
C(25)-H(25)	1.0000
C(25)-C(26)	1.523(9)
C(25)-C(30)	1.534(9)
C(26)-H(26A)	0.9900
C(26)-H(26B)	0.9900
C(26)-C(27)	1.538(10)
C(27)-H(27A)	0.9900
C(27)-H(27B)	0.9900
C(27)-C(28)	1.505(11)
C(28)-H(28A)	0.9900
C(28)-H(28B)	0.9900
C(28)-C(29)	1.507(10)
C(29)-H(29A)	0.9900
C(29)-H(29B)	0.9900
C(29)-C(30)	1.530(10)
C(30)-H(30A)	0.9900
C(30)-H(30B)	0.9900
Cl(1)-Mg(1)	2.396(2)
Mg(1)-O(1)	2.107(4)
Mg(1)-O(2)	2.116(4)

Mg(1)-O(3)	2.105(4)
Mg(1)-O(4)	2.131(4)
Mg(1)-O(5)	2.116(4)
O(1)-C(31)	1.457(7)
O(1)-C(34)	1.439(7)
O(2)-C(35)	1.449(7)
O(2)-C(38)	1.454(6)
O(3)-C(39)	1.453(7)
O(3)-C(42)	1.423(8)
O(4)-C(43)	1.430(8)
O(4)-C(46)	1.455(7)
O(5)-C(47)	1.448(7)
O(5)-C(50)	1.455(7)
C(31)-H(31A)	0.9900
C(31)-H(31B)	0.9900
C(31)-C(32)	1.514(9)
C(32)-H(32A)	0.9900
C(32)-H(32B)	0.9900
C(32)-C(33)	1.504(11)
C(33)-H(33A)	0.9900
C(33)-H(33B)	0.9900
C(33)-C(34)	1.509(8)
C(34)-H(34A)	0.9900
C(34)-H(34B)	0.9900
C(35)-H(35A)	0.9900
C(35)-H(35B)	0.9900
C(35)-C(36)	1.526(9)
C(36)-H(36A)	0.9900
C(36)-H(36B)	0.9900
C(36)-C(37)	1.501(12)
C(37)-H(37A)	0.9900
C(37)-H(37B)	0.9900
C(37)-C(38)	1.506(9)
C(38)-H(38A)	0.9900
C(38)-H(38B)	0.9900
C(39)-H(39A)	0.9900
C(39)-H(39B)	0.9900
C(39)-C(40)	1.525(8)

C(40)-H(40A)	0.9900
C(40)-H(40B)	0.9900
C(40)-C(41)	1.511(10)
C(41)-H(41A)	0.9900
C(41)-H(41B)	0.9900
C(41)-C(42)	1.520(10)
C(42)-H(42A)	0.9900
C(42)-H(42B)	0.9900
C(43)-H(43A)	0.9900
C(43)-H(43B)	0.9900
C(43)-C(44)	1.447(12)
C(44)-H(44A)	0.9900
C(44)-H(44B)	0.9900
C(44)-C(45)	1.514(11)
C(45)-H(45A)	0.9900
C(45)-H(45B)	0.9900
C(45)-C(46)	1.516(10)
C(46)-H(46A)	0.9900
C(46)-H(46B)	0.9900
C(47)-H(47A)	0.9900
C(47)-H(47B)	0.9900
C(47)-C(48)	1.516(10)
C(48)-H(48A)	0.9900
C(48)-H(48B)	0.9900
C(48)-C(49)	1.529(9)
C(49)-H(49A)	0.9900
C(49)-H(49B)	0.9900
C(49)-C(50)	1.508(9)
C(50)-H(50A)	0.9900
C(50)-H(50B)	0.9900
C(51)-H(51A)	0.9800
C(51)-H(51B)	0.9800
C(51)-H(51C)	0.9800
C(51)-C(52)	1.54(2)
C(52)-C(53)	1.289(14)
C(52)-C(57)	1.309(13)
C(53)-H(53)	0.9500
C(53)-C(54)	1.434(14)

C(54)-H(54)	0.9500
C(54)-C(55)	1.311(14)
C(55)-H(55)	0.9500
C(55)-C(56)	1.244(14)
C(56)-H(56)	0.9500
C(56)-C(57)	1.418(14)
C(57)-H(57)	0.9500
C(1)-Fe(1)-C(2)	37.8(3)
C(1)-Fe(1)-C(9)	118.0(2)
C(1)-Fe(1)-C(10)	98.8(2)
C(1)-Fe(1)-C(17)	119.8(2)
C(1)-Fe(1)-C(18)	128.1(2)
C(1)-Fe(1)-C(25)	93.2(3)
C(2)-Fe(1)-C(10)	90.4(2)
C(9)-Fe(1)-C(2)	125.7(2)
C(9)-Fe(1)-C(10)	37.3(2)
C(9)-Fe(1)-C(18)	100.1(2)
C(9)-Fe(1)-C(25)	88.1(2)
C(17)-Fe(1)-C(2)	99.2(2)
C(17)-Fe(1)-C(9)	122.2(3)
C(17)-Fe(1)-C(10)	127.7(2)
C(17)-Fe(1)-C(18)	38.1(2)
C(17)-Fe(1)-C(25)	91.2(3)
C(18)-Fe(1)-C(2)	91.8(2)
C(18)-Fe(1)-C(10)	90.8(2)
C(25)-Fe(1)-C(2)	127.4(3)
C(25)-Fe(1)-C(10)	122.5(2)
C(25)-Fe(1)-C(18)	123.7(2)
Fe(1)-C(1)-H(1A)	116(4)
Fe(1)-C(1)-H(1B)	112(4)
H(1A)-C(1)-H(1B)	109(6)
C(2)-C(1)-Fe(1)	77.3(4)
C(2)-C(1)-H(1A)	121(4)
C(2)-C(1)-H(1B)	117(4)
Fe(1)-C(2)-H(2)	112(4)
C(1)-C(2)-Fe(1)	65.0(3)
C(1)-C(2)-H(2)	119(4)
C(1)-C(2)-C(3)	124.1(7)

C(3)-C(2)-Fe(1)	115.4(4)
C(3)-C(2)-H(2)	111(4)
C(4)-C(3)-C(2)	123.2(7)
C(8)-C(3)-C(2)	120.9(7)
C(8)-C(3)-C(4)	115.8(6)
C(3)-C(4)-H(4)	119.3
C(5)-C(4)-C(3)	121.4(8)
C(5)-C(4)-H(4)	119.3
C(4)-C(5)-H(5)	119.8
C(6)-C(5)-C(4)	120.4(9)
C(6)-C(5)-H(5)	119.8
C(5)-C(6)-H(6)	119.7
C(5)-C(6)-C(7)	120.6(7)
C(7)-C(6)-H(6)	119.7
C(6)-C(7)-H(7)	120.7
C(6)-C(7)-C(8)	118.7(8)
C(8)-C(7)-H(7)	120.7
C(3)-C(8)-C(7)	123.0(9)
C(3)-C(8)-H(8)	118.5
C(7)-C(8)-H(8)	118.5
Fe(1)-C(9)-H(9A)	104(3)
Fe(1)-C(9)-H(9B)	108(3)
H(9A)-C(9)-H(9B)	118(4)
C(10)-C(9)-Fe(1)	77.8(3)
C(10)-C(9)-H(9A)	122(3)
C(10)-C(9)-H(9B)	116(3)
Fe(1)-C(10)-H(10)	113(5)
C(9)-C(10)-Fe(1)	64.9(3)
C(9)-C(10)-H(10)	124(5)
C(9)-C(10)-C(11)	125.0(6)
C(11)-C(10)-Fe(1)	117.2(4)
C(11)-C(10)-H(10)	106(5)
C(12)-C(11)-C(10)	123.1(6)
C(16)-C(11)-C(10)	119.8(6)
C(16)-C(11)-C(12)	117.1(6)
C(11)-C(12)-H(12)	119.6
C(13)-C(12)-C(11)	120.8(7)
C(13)-C(12)-H(12)	119.6

C(12)-C(13)-H(13)	119.3
C(14)-C(13)-C(12)	121.4(7)
C(14)-C(13)-H(13)	119.3
C(13)-C(14)-H(14)	120.1
C(13)-C(14)-C(15)	119.7(7)
C(15)-C(14)-H(14)	120.1
C(14)-C(15)-H(15)	119.8
C(14)-C(15)-C(16)	120.4(7)
C(16)-C(15)-H(15)	119.8
C(11)-C(16)-C(15)	120.7(7)
C(11)-C(16)-H(16)	119.6
C(15)-C(16)-H(16)	119.6
Fe(1)-C(17)-H(17A)	110(4)
Fe(1)-C(17)-H(17B)	105(4)
H(17A)-C(17)-H(17B)	119(6)
C(18)-C(17)-Fe(1)	76.5(3)
C(18)-C(17)-H(17A)	119(5)
C(18)-C(17)-H(17B)	117(4)
Fe(1)-C(18)-H(18)	107(5)
C(17)-C(18)-Fe(1)	65.4(3)
C(17)-C(18)-H(18)	122(5)
C(17)-C(18)-C(19)	124.7(6)
C(19)-C(18)-Fe(1)	113.8(4)
C(19)-C(18)-H(18)	111(5)
C(20)-C(19)-C(18)	123.9(6)
C(20)-C(19)-C(24)	114.9(6)
C(24)-C(19)-C(18)	121.2(6)
C(19)-C(20)-H(20)	118.6
C(21)-C(20)-C(19)	122.9(7)
C(21)-C(20)-H(20)	118.6
C(20)-C(21)-H(21)	119.5
C(22)-C(21)-C(20)	121.0(8)
C(22)-C(21)-H(21)	119.5
C(21)-C(22)-H(22)	121.0
C(21)-C(22)-C(23)	118.0(8)
C(23)-C(22)-H(22)	121.0
C(22)-C(23)-H(23)	119.5
C(24)-C(23)-C(22)	120.9(8)

C(24)-C(23)-H(23)	119.5
C(19)-C(24)-H(24)	118.9
C(23)-C(24)-C(19)	122.2(8)
C(23)-C(24)-H(24)	118.9
Fe(1)-C(25)-H(25)	105.3
C(26)-C(25)-Fe(1)	116.0(4)
C(26)-C(25)-H(25)	105.3
C(26)-C(25)-C(30)	109.0(5)
C(30)-C(25)-Fe(1)	114.8(4)
C(30)-C(25)-H(25)	105.3
C(25)-C(26)-H(26A)	109.0
C(25)-C(26)-H(26B)	109.0
C(25)-C(26)-C(27)	112.7(5)
H(26A)-C(26)-H(26B)	107.8
C(27)-C(26)-H(26A)	109.0
C(27)-C(26)-H(26B)	109.0
C(26)-C(27)-H(27A)	109.3
C(26)-C(27)-H(27B)	109.3
H(27A)-C(27)-H(27B)	108.0
C(28)-C(27)-C(26)	111.5(6)
C(28)-C(27)-H(27A)	109.3
C(28)-C(27)-H(27B)	109.3
C(27)-C(28)-H(28A)	109.2
C(27)-C(28)-H(28B)	109.2
C(27)-C(28)-C(29)	111.8(6)
H(28A)-C(28)-H(28B)	107.9
C(29)-C(28)-H(28A)	109.2
C(29)-C(28)-H(28B)	109.2
C(28)-C(29)-H(29A)	109.3
C(28)-C(29)-H(29B)	109.3
C(28)-C(29)-C(30)	111.5(6)
H(29A)-C(29)-H(29B)	108.0
C(30)-C(29)-H(29A)	109.3
C(30)-C(29)-H(29B)	109.3
C(25)-C(30)-H(30A)	108.9
C(25)-C(30)-H(30B)	108.9
C(29)-C(30)-C(25)	113.6(6)
C(29)-C(30)-H(30A)	108.9

C(29)-C(30)-H(30B)	108.9
H(30A)-C(30)-H(30B)	107.7
O(1)-Mg(1)-Cl(1)	178.34(15)
O(1)-Mg(1)-O(2)	90.08(16)
O(1)-Mg(1)-O(4)	88.58(16)
O(1)-Mg(1)-O(5)	85.93(17)
O(2)-Mg(1)-Cl(1)	91.47(13)
O(2)-Mg(1)-O(4)	177.92(18)
O(2)-Mg(1)-O(5)	90.73(16)
O(3)-Mg(1)-Cl(1)	94.18(14)
O(3)-Mg(1)-O(1)	85.27(17)
O(3)-Mg(1)-O(2)	87.95(16)
O(3)-Mg(1)-O(4)	93.52(17)
O(3)-Mg(1)-O(5)	171.10(18)
O(4)-Mg(1)-Cl(1)	89.89(13)
O(5)-Mg(1)-Cl(1)	94.66(13)
O(5)-Mg(1)-O(4)	87.59(16)
C(31)-O(1)-Mg(1)	122.5(4)
C(34)-O(1)-Mg(1)	127.6(3)
C(34)-O(1)-C(31)	109.7(4)
C(35)-O(2)-Mg(1)	129.5(3)
C(35)-O(2)-C(38)	106.6(4)
C(38)-O(2)-Mg(1)	119.3(4)
C(39)-O(3)-Mg(1)	122.8(4)
C(42)-O(3)-Mg(1)	128.2(4)
C(42)-O(3)-C(39)	107.6(5)
C(43)-O(4)-Mg(1)	127.8(4)
C(43)-O(4)-C(46)	107.1(5)
C(46)-O(4)-Mg(1)	120.4(3)
C(47)-O(5)-Mg(1)	125.8(4)
C(47)-O(5)-C(50)	109.1(4)
C(50)-O(5)-Mg(1)	124.5(3)
O(1)-C(31)-H(31A)	110.8
O(1)-C(31)-H(31B)	110.8
O(1)-C(31)-C(32)	104.7(5)
H(31A)-C(31)-H(31B)	108.9
C(32)-C(31)-H(31A)	110.8
C(32)-C(31)-H(31B)	110.8

C(31)-C(32)-H(32A)	111.2
C(31)-C(32)-H(32B)	111.2
H(32A)-C(32)-H(32B)	109.1
C(33)-C(32)-C(31)	103.0(5)
C(33)-C(32)-H(32A)	111.2
C(33)-C(32)-H(32B)	111.2
C(32)-C(33)-H(33A)	111.4
C(32)-C(33)-H(33B)	111.4
C(32)-C(33)-C(34)	101.7(5)
H(33A)-C(33)-H(33B)	109.3
C(34)-C(33)-H(33A)	111.4
C(34)-C(33)-H(33B)	111.4
O(1)-C(34)-C(33)	105.2(5)
O(1)-C(34)-H(34A)	110.7
O(1)-C(34)-H(34B)	110.7
C(33)-C(34)-H(34A)	110.7
C(33)-C(34)-H(34B)	110.7
H(34A)-C(34)-H(34B)	108.8
O(2)-C(35)-H(35A)	110.5
O(2)-C(35)-H(35B)	110.5
O(2)-C(35)-C(36)	106.0(5)
H(35A)-C(35)-H(35B)	108.7
C(36)-C(35)-H(35A)	110.5
C(36)-C(35)-H(35B)	110.5
C(35)-C(36)-H(36A)	110.8
C(35)-C(36)-H(36B)	110.8
H(36A)-C(36)-H(36B)	108.9
C(37)-C(36)-C(35)	104.7(6)
C(37)-C(36)-H(36A)	110.8
C(37)-C(36)-H(36B)	110.8
C(36)-C(37)-H(37A)	111.2
C(36)-C(37)-H(37B)	111.2
C(36)-C(37)-C(38)	102.6(6)
H(37A)-C(37)-H(37B)	109.2
C(38)-C(37)-H(37A)	111.2
C(38)-C(37)-H(37B)	111.2
O(2)-C(38)-C(37)	102.7(5)
O(2)-C(38)-H(38A)	111.2

O(2)-C(38)-H(38B)	111.2
C(37)-C(38)-H(38A)	111.2
C(37)-C(38)-H(38B)	111.2
H(38A)-C(38)-H(38B)	109.1
O(3)-C(39)-H(39A)	110.5
O(3)-C(39)-H(39B)	110.5
O(3)-C(39)-C(40)	106.2(5)
H(39A)-C(39)-H(39B)	108.7
C(40)-C(39)-H(39A)	110.5
C(40)-C(39)-H(39B)	110.5
C(39)-C(40)-H(40A)	110.8
C(39)-C(40)-H(40B)	110.8
H(40A)-C(40)-H(40B)	108.9
C(41)-C(40)-C(39)	104.8(5)
C(41)-C(40)-H(40A)	110.8
C(41)-C(40)-H(40B)	110.8
C(40)-C(41)-H(41A)	111.3
C(40)-C(41)-H(41B)	111.3
C(40)-C(41)-C(42)	102.3(6)
H(41A)-C(41)-H(41B)	109.2
C(42)-C(41)-H(41A)	111.3
C(42)-C(41)-H(41B)	111.3
O(3)-C(42)-C(41)	104.0(6)
O(3)-C(42)-H(42A)	111.0
O(3)-C(42)-H(42B)	111.0
C(41)-C(42)-H(42A)	111.0
C(41)-C(42)-H(42B)	111.0
H(42A)-C(42)-H(42B)	109.0
O(4)-C(43)-H(43A)	110.7
O(4)-C(43)-H(43B)	110.7
O(4)-C(43)-C(44)	105.3(6)
H(43A)-C(43)-H(43B)	108.8
C(44)-C(43)-H(43A)	110.7
C(44)-C(43)-H(43B)	110.7
C(43)-C(44)-H(44A)	111.1
C(43)-C(44)-H(44B)	111.1
C(43)-C(44)-C(45)	103.5(7)
H(44A)-C(44)-H(44B)	109.0

C(45)-C(44)-H(44A)	111.1
C(45)-C(44)-H(44B)	111.1
C(44)-C(45)-H(45A)	111.1
C(44)-C(45)-H(45B)	111.1
C(44)-C(45)-C(46)	103.2(5)
H(45A)-C(45)-H(45B)	109.1
C(46)-C(45)-H(45A)	111.1
C(46)-C(45)-H(45B)	111.1
O(4)-C(46)-C(45)	106.3(5)
O(4)-C(46)-H(46A)	110.5
O(4)-C(46)-H(46B)	110.5
C(45)-C(46)-H(46A)	110.5
C(45)-C(46)-H(46B)	110.5
H(46A)-C(46)-H(46B)	108.7
O(5)-C(47)-H(47A)	110.5
O(5)-C(47)-H(47B)	110.5
O(5)-C(47)-C(48)	106.1(5)
H(47A)-C(47)-H(47B)	108.7
C(48)-C(47)-H(47A)	110.5
C(48)-C(47)-H(47B)	110.5
C(47)-C(48)-H(48A)	111.3
C(47)-C(48)-H(48B)	111.3
C(47)-C(48)-C(49)	102.3(5)
H(48A)-C(48)-H(48B)	109.2
C(49)-C(48)-H(48A)	111.3
C(49)-C(48)-H(48B)	111.3
C(48)-C(49)-H(49A)	111.4
C(48)-C(49)-H(49B)	111.4
H(49A)-C(49)-H(49B)	109.3
C(50)-C(49)-C(48)	101.9(5)
C(50)-C(49)-H(49A)	111.4
C(50)-C(49)-H(49B)	111.4
O(5)-C(50)-C(49)	106.0(5)
O(5)-C(50)-H(50A)	110.5
O(5)-C(50)-H(50B)	110.5
C(49)-C(50)-H(50A)	110.5
C(49)-C(50)-H(50B)	110.5
H(50A)-C(50)-H(50B)	108.7

H(51A)-C(51)-H(51B)	109.5
H(51A)-C(51)-H(51C)	109.5
H(51B)-C(51)-H(51C)	109.5
C(52)-C(51)-H(51A)	109.5
C(52)-C(51)-H(51B)	109.5
C(52)-C(51)-H(51C)	109.5
C(53)-C(52)-C(51)	123.7(12)
C(53)-C(52)-C(57)	110.3(13)
C(57)-C(52)-C(51)	122.7(12)
C(52)-C(53)-H(53)	119.6
C(52)-C(53)-C(54)	120.9(11)
C(54)-C(53)-H(53)	119.6
C(53)-C(54)-H(54)	120.2
C(55)-C(54)-C(53)	119.5(11)
C(55)-C(54)-H(54)	120.2
C(54)-C(55)-H(55)	121.0
C(56)-C(55)-C(54)	118.0(13)
C(56)-C(55)-H(55)	121.0
C(55)-C(56)-H(56)	121.8
C(55)-C(56)-C(57)	116.5(11)
C(57)-C(56)-H(56)	121.8
C(52)-C(57)-C(56)	126.0(11)
C(52)-C(57)-H(57)	117.0
C(56)-C(57)-H(57)	117.0

Anisotropic displacement parameters ($Å^2 x 10^3$) for **neijd09**.

The anisotropic displacement factor exponent takes the form: $-2\pi^2[h^2 a^{*2}U_{11} + ... + 2 h k a^* b^* U_{12}]$

	U_{11}	U_{22}	U_{33}	U_{23}	U_{13}	U_{12}
Fe1	32(1)	41(1)	45(1)	-2(1)	16(1)	-3(1)
C1	34(3)	50(4)	45(3)	-7(3)	10(2)	-4(2)
C2	38(3)	56(4)	40(3)	-3(3)	13(2)	-8(3)
C3	38(3)	70(4)	41(3)	-9(3)	15(2)	-13(3)
C4	43(3)	80(5)	55(4)	-18(4)	24(3)	-3(3)
C5	42(3)	103(7)	56(4)	-26(4)	20(3)	-10(4)
C6	51(4)	116(8)	59(4)	-32(5)	25(3)	-21(5)
C7	76(5)	110(7)	40(3)	-18(4)	29(3)	-53(5)
C8	53(4)	80(5)	45(3)	-10(3)	17(3)	-21(4)
C9	37(3)	46(3)	49(3)	-11(3)	23(3)	2(2)
C10	34(3)	43(3)	55(3)	-2(3)	22(2)	4(2)
C11	35(3)	50(3)	48(3)	-5(3)	19(2)	2(2)
C12	33(3)	63(4)	53(3)	-4(3)	21(2)	-5(3)
C13	40(3)	75(4)	60(4)	-20(4)	27(3)	-6(3)
C14	42(3)	74(5)	61(4)	-7(4)	18(3)	11(3)
C15	58(4)	72(5)	54(4)	2(3)	16(3)	27(4)
C16	51(3)	51(4)	61(4)	2(3)	23(3)	13(3)
C17	26(2)	60(4)	52(3)	-5(3)	16(2)	-4(2)
C18	31(3)	43(3)	56(4)	2(3)	18(2)	-12(2)
C19	29(2)	47(3)	62(4)	-2(3)	20(3)	-9(2)
C20	34(3)	57(4)	68(4)	-18(3)	22(3)	-10(3)
C21	34(3)	75(5)	75(5)	-31(4)	23(3)	-16(3)
C22	45(4)	65(5)	115(7)	-37(5)	38(4)	-18(3)
C23	47(4)	42(4)	137(8)	-13(4)	39(4)	-13(3)
C24	40(3)	47(4)	88(5)	-4(3)	24(3)	-12(3)
C25	38(3)	42(3)	56(3)	4(3)	18(3)	0(2)
C26	45(3)	52(4)	53(3)	-3(3)	17(3)	0(3)
C27	58(4)	47(4)	66(4)	2(3)	25(3)	6(3)
C28	63(4)	57(4)	67(4)	7(4)	28(4)	12(3)
C29	67(4)	66(5)	56(4)	4(4)	31(3)	8(4)
C30	58(4)	50(4)	50(3)	3(3)	23(3)	8(3)
Cl1	38(1)	59(1)	52(1)	-18(1)	18(1)	-10(1)
Mg1	28(1)	39(1)	36(1)	0(1)	12(1)	0(1)

O1	35(2)	44(2)	35(2)	-5(2)	11(2)	-1(2)
O2	29(2)	40(2)	48(2)	2(2)	17(2)	1(2)
O3	49(2)	39(2)	47(2)	6(2)	25(2)	-1(2)
O4	38(2)	49(2)	41(2)	3(2)	13(2)	10(2)
O5	31(2)	48(2)	39(2)	7(2)	14(2)	1(2)
C31	28(2)	63(4)	39(3)	-2(3)	3(2)	0(2)
C32	43(3)	77(5)	41(3)	-7(3)	13(3)	-5(3)
C33	46(3)	61(4)	53(3)	-18(3)	25(3)	-12(3)
C34	39(3)	45(3)	51(3)	-12(3)	15(2)	-2(2)
C35	39(3)	47(3)	60(4)	11(3)	23(3)	6(3)
C36	45(3)	69(5)	75(5)	29(4)	19(3)	10(3)
C37	39(3)	80(5)	64(4)	15(4)	12(3)	-3(3)
C38	27(2)	49(3)	61(4)	4(3)	17(2)	-4(2)
C39	48(3)	53(4)	39(3)	0(3)	22(3)	-6(3)
C40	49(3)	61(4)	49(3)	2(3)	23(3)	-13(3)
C41	71(4)	50(4)	61(4)	-1(3)	28(3)	-14(4)
C42	78(4)	40(3)	80(5)	4(4)	43(4)	1(3)
C43	71(5)	71(5)	68(5)	21(4)	28(4)	33(4)
C44	74(5)	54(4)	99(6)	14(4)	35(5)	15(4)
C45	37(3)	51(3)	59(3)	-15(3)	13(2)	-3(3)
C46	35(3)	53(4)	50(3)	-1(3)	21(2)	-1(3)
C47	40(3)	68(4)	37(3)	5(3)	13(2)	5(3)
C48	49(3)	63(4)	56(4)	24(3)	24(3)	12(3)
C49	43(3)	68(4)	58(4)	14(3)	24(3)	0(3)
C50	36(3)	55(4)	47(3)	4(3)	16(2)	-12(2)
C51	156(13)	94(10)	290(20)	-16(12)	116(15)	2(9)
C52	83(5)	93(7)	100(6)	-14(5)	17(5)	8(5)
C53	117(8)	79(7)	131(8)	33(7)	61(7)	35(6)
C54	95(6)	104(8)	93(6)	-26(6)	40(5)	6(6)
C55	64(6)	280(20)	124(8)	-67(12)	28(6)	-11(9)
C56	82(6)	91(7)	119(7)	-39(6)	21(5)	8(5)
C57	76(5)	193(14)	72(6)	-40(7)	4(4)	13(7)

Hydrogen coordinates (x 10^4) and isotropic displacement parameters (Å^2 x 10^3) for **neijd09**.

	x	y	z	U(eq)
H1A	2420(60)	2280(60)	370(40)	47(18)
H1B	1180(70)	2960(60)	280(50)	70(20)
H2	1940(60)	4530(60)	-170(50)	60(20)
H4	4022	2445	176	68
H5	5524	2503	-339	79
H6	5892	3998	-992	88
H7	4774	5493	-1131	87
H8	3270	5445	-611	70
H9A	1310(50)	3770(60)	2250(40)	44(16)
H9B	2250(50)	4900(50)	2650(40)	29(14)
H10	1670(70)	5760(70)	1330(50)	70(20)
H12	-64	3491	1123	58
H13	-1703	3332	77	67
H14	-2242	4581	-958	70
H15	-1137	6029	-971	74
H16	530	6231	72	64
H17A	4750(60)	3950(60)	2420(40)	53(19)
H17B	4750(60)	3790(70)	1370(50)	70(20)
H18	3910(70)	5490(70)	1170(50)	70(20)
H20	4499	5056	3341	61
H21	4344	6295	4303	72
H22	3623	7956	3885	86
H23	3078	8361	2451	87
H24	3304	7154	1488	68
H25	2245	2219	2139	53
H26A	4440	1914	2150	59
H26B	3402	1460	1456	59
H27A	2972	343	2442	67
H27B	4206	127	2456	67
H28A	4856	1131	3658	73
H28B	4036	262	3823	73
H29A	3725	1960	4320	72
H29B	2662	1508	3649	72
H30A	4104	3077	3331	61
H30B	2871	3278	3321	61
H31A	6975	6920	1531	54

H31B	6233	6220	1956	54
H32A	5894	5328	731	64
H32B	7067	5615	620	64
H33A	7337	3895	1090	62
H33B	6624	4064	1730	62
H34A	8336	4329	2611	53
H34B	8761	4861	1891	53
H35A	10323	4329	3259	56
H35B	9792	4508	4007	56
H36A	11453	4451	4905	76
H36B	11990	4378	4150	76
H37A	12578	6015	4592	74
H37B	11568	6231	4960	74
H38A	11071	7178	3726	54
H38B	11511	6267	3233	54
H39A	8769	7190	1286	54
H39B	9732	6533	1921	54
H40A	10931	7609	1614	61
H40B	9963	8331	1047	61
H41A	10597	9517	2081	70
H41B	11097	8610	2762	70
H42A	9529	9062	3101	74
H42B	8843	9215	2137	74
H43A	6047	8251	2305	82
H43B	7253	8638	2341	82
H44A	6107	9858	2874	88
H44B	7359	9719	3421	88
H45A	5485	8797	3733	59
H45B	6555	9248	4412	59
H46A	7398	7698	4494	53
H46B	6251	7204	3964	53
H47A	8517	6200	5305	58
H47B	9378	5355	5154	58
H48A	7903	4747	5825	65
H48B	8290	3954	5213	65
H49A	6438	4079	4530	65
H49B	6401	5315	4762	65
H50A	7279	4409	3537	54

H50B	6568	5472	3451	54
H51A	3516	7635	7023	253
H51B	2659	7496	6122	253
H51C	3256	6482	6622	253
H53	2857	6427	8131	124
H54	1410	6458	8754	114
H55	-222	7180	8005	187
H56	-629	6907	6620	119
H57	847	6944	6058	141

Torsion angles [°] for **neijd09**.

Fe1-C1-C2-C3	104.8(5)
Fe1-C2-C3-C4	68.9(7)
Fe1-C2-C3-C8	-112.0(6)
Fe1-C9-C10-C11	106.7(6)
Fe1-C10-C11-C12	66.3(7)
Fe1-C10-C11-C16	-115.7(6)
Fe1-C17-C18-C19	102.8(5)
Fe1-C18-C19-C20	71.3(6)
Fe1-C18-C19-C24	-107.7(6)
Fe1-C25-C26-C27	-174.8(4)
Fe1-C25-C30-C29	174.7(4)
C1-C2-C3-C4	-7.0(9)
C1-C2-C3-C8	172.1(6)
C2-C3-C4-C5	177.8(6)
C2-C3-C8-C7	-177.7(6)
C3-C4-C5-C6	0.3(10)
C4-C3-C8-C7	1.4(9)
C4-C5-C6-C7	0.6(11)
C5-C6-C7-C8	-0.4(11)
C6-C7-C8-C3	-0.6(10)
C8-C3-C4-C5	-1.3(9)
C9-C10-C11-C12	-10.9(9)
C9-C10-C11-C16	167.2(6)
C10-C11-C12-C13	177.4(5)
C10-C11-C16-C15	-177.5(6)
C11-C12-C13-C14	0.3(9)
C12-C11-C16-C15	0.7(9)
C12-C13-C14-C15	0.2(10)
C13-C14-C15-C16	-0.2(10)
C14-C15-C16-C11	-0.2(10)
C16-C11-C12-C13	-0.7(9)
C17-C18-C19-C20	-4.4(9)
C17-C18-C19-C24	176.7(5)
C18-C19-C20-C21	-179.0(5)
C18-C19-C24-C23	177.4(6)
C19-C20-C21-C22	1.1(9)
C20-C19-C24-C23	-1.7(8)

C20-C21-C22-C23	-0.7(10)
C21-C22-C23-C24	-0.9(10)
C22-C23-C24-C19	2.1(10)
C24-C19-C20-C21	0.1(8)
C25-C26-C27-C28	-55.8(8)
C26-C25-C30-C29	-53.2(7)
C26-C27-C28-C29	54.6(8)
C27-C28-C29-C30	-53.6(9)
C28-C29-C30-C25	53.9(8)
C30-C25-C26-C27	53.8(7)
Mg1-O1-C31-C32	167.5(4)
Mg1-O1-C34-C33	167.9(4)
Mg1-O2-C35-C36	-134.1(5)
Mg1-O2-C38-C37	119.5(5)
Mg1-O3-C39-C40	-147.9(4)
Mg1-O3-C42-C41	130.3(5)
Mg1-O4-C43-C44	-122.7(6)
Mg1-O4-C46-C45	144.8(4)
Mg1-O5-C47-C48	-179.0(4)
Mg1-O5-C50-C49	-156.5(4)
O1-C31-C32-C33	29.8(6)
O2-C35-C36-C37	4.8(7)
O3-C39-C40-C41	5.0(7)
O4-C43-C44-C45	-38.9(8)
O5-C47-C48-C49	-30.1(7)
C31-O1-C34-C33	-16.4(6)
C31-C32-C33-C34	-39.1(6)
C32-C33-C34-O1	34.5(6)
C34-O1-C31-C32	-8.4(6)
C35-O2-C38-C37	-38.8(6)
C35-C36-C37-C38	-27.6(7)
C36-C37-C38-O2	40.7(7)
C38-O2-C35-C36	21.2(6)
C39-O3-C42-C41	-36.6(7)
C39-C40-C41-C42	-25.8(7)
C40-C41-C42-O3	38.4(7)
C42-O3-C39-C40	19.8(7)
C43-O4-C46-C45	-12.9(7)

C43-C44-C45-C46	29.9(8)
C44-C45-C46-O4	-10.7(7)
C46-O4-C43-C44	32.7(8)
C47-O5-C50-C49	14.4(7)
C47-C48-C49-C50	37.7(7)
C48-C49-C50-O5	-32.5(7)
C50-O5-C47-C48	10.2(7)
C51-C52-C53-C54	174.8(12)
C51-C52-C57-C56	-174.9(14)
C52-C53-C54-C55	5(2)
C53-C52-C57-C56	25(2)
C53-C54-C55-C56	21(3)
C54-C55-C56-C57	-22(3)
C55-C56-C57-C52	-2(3)
C57-C52-C53-C54	-25.3(18)

3. Atomic coordinates of neijd10

Atomic coordinates (x 10^4) and equivalent isotropic displacement parameters ($Å^2$x 10^3) for **neijd10**. U_{eq} is defined as one third of the trace of the orthogonalized U_{ij} tensor.

	x	y	z	U_{eq}
Fe1	3914(1)	1748(1)	6574(1)	43(1)
N1	3484(2)	503(1)	7611(1)	47(1)
N2	2955(2)	-56(1)	6704(1)	46(1)
C1	2807(2)	2569(2)	6900(2)	52(1)
C2	3536(2)	3072(2)	6586(1)	50(1)
C3	4444(2)	3575(2)	6908(1)	51(1)
C4	4867(2)	3429(2)	7530(1)	54(1)
C5	5701(3)	3927(2)	7822(1)	60(1)
C6	6163(3)	4587(2)	7499(2)	66(1)
C7	5776(3)	4738(2)	6884(2)	67(1)
C8	4926(3)	4245(2)	6587(1)	57(1)
C9	5098(2)	1090(2)	6180(1)	50(1)
C10	5042(2)	1966(2)	5959(1)	50(1)
C11	4498(2)	2238(2)	5344(1)	51(1)
C12	3693(3)	1746(2)	5008(2)	63(1)
C13	3198(3)	2040(3)	4432(2)	75(1)
C14	3498(3)	2831(3)	4183(2)	78(1)
C15	4281(3)	3314(2)	4505(2)	74(1)
C16	4783(3)	3034(2)	5082(2)	63(1)
C17	3413(2)	659(1)	6994(1)	43(1)
C18	3132(3)	-389(2)	7767(1)	54(1)
C19	2613(3)	-718(2)	7138(1)	64(1)
C20	3860(2)	1110(2)	8102(1)	46(1)
C21	3094(2)	1578(2)	8386(1)	50(1)
C22	3464(3)	2175(2)	8857(1)	55(1)
C23	4552(2)	2289(2)	9050(1)	54(1)
C24	5286(2)	1784(2)	8773(1)	52(1)
C25	4961(2)	1186(2)	8295(1)	48(1)
C26	1906(2)	1455(2)	8199(2)	60(1)
C27	4917(3)	2954(2)	9548(2)	72(1)
C28	5786(3)	653(2)	8007(2)	60(1)
C29	2718(2)	-192(2)	6045(1)	45(1)
C30	1806(2)	185(2)	5716(1)	48(1)

C31	1599(2)	26(2)	5076(1)	51(1)
C32	2261(2)	-516(2)	4766(1)	55(1)
C33	3146(2)	-904(2)	5113(1)	54(1)
C34	3384(2)	-760(2)	5751(1)	49(1)
C35	1057(2)	756(2)	6037(2)	67(1)
C36	2035(3)	-668(3)	4072(2)	83(1)
C37	4361(3)	-1192(2)	6112(1)	61(1)
O1	1859(2)	8334(2)	2537(2)	74(1)
C38	2416(4)	7542(3)	2656(3)	76(1)
C39	1589(4)	6809(3)	2558(3)	79(1)
C40	569(4)	7286(4)	2768(3)	82(1)
C41	752(4)	8173(3)	2527(3)	73(1)
C42	2198(6)	6464(5)	4350(4)	127(3)
C43	2435(6)	5813(5)	4812(3)	113(2)
C44	1731(7)	5096(7)	4938(3)	129(3)
C45	1844(6)	4482(5)	5409(4)	107(3)
C46	1055(6)	3863(4)	5561(3)	121(3)
O1'	348(12)	6734(11)	2768(7)	107(4)
C39'	978(17)	7271(15)	2423(8)	101(4)
C40'	1977(14)	7508(15)	2840(10)	95(5)
C41'	1804(16)	7080(15)	3463(10)	126(9)
C42'	618(17)	7017(18)	3407(8)	134(9)
O2'	2628(16)	4905(15)	5656(9)	161(7)
C43'	3051(18)	5472(17)	5208(10)	127(5)
C44'	2430(20)	5280(20)	4593(9)	142(5)
C45'	1280(20)	5080(30)	4773(12)	145(5)
C46'	1511(17)	4810(20)	5438(13)	120(5)

Bond lengths [Å] and angles [°] for **neijd10**.

Fe(1)-C(1)	2.046(3)
Fe(1)-C(2)	2.067(2)
Fe(1)-C(9)	2.050(3)
Fe(1)-C(10)	2.072(3)
Fe(1)-C(17)	2.020(2)
N(1)-C(17)	1.341(3)
N(1)-C(18)	1.477(3)
N(1)-C(20)	1.438(3)
N(2)-C(17)	1.346(3)
N(2)-C(19)	1.470(3)
N(2)-C(29)	1.429(3)
C(1)-H(1A)	1.02(3)
C(1)-H(1B)	0.99(4)
C(1)-C(2)	1.419(4)
C(2)-H(2)	0.96(3)
C(2)-C(3)	1.470(4)
C(3)-C(4)	1.396(4)
C(3)-C(8)	1.405(4)
C(4)-H(4)	0.9500
C(4)-C(5)	1.377(4)
C(5)-H(5)	0.9500
C(5)-C(6)	1.386(4)
C(6)-H(6)	0.9500
C(6)-C(7)	1.372(5)
C(7)-H(7)	0.9500
C(7)-C(8)	1.389(4)
C(8)-H(8)	0.9500
C(9)-H(9A)	0.97(3)
C(9)-H(9B)	1.11(4)
C(9)-C(10)	1.413(4)
C(10)-H(10)	0.96(4)
C(10)-C(11)	1.472(4)
C(11)-C(12)	1.384(4)
C(11)-C(16)	1.398(4)
C(12)-H(12)	0.9500
C(12)-C(13)	1.390(5)
C(13)-H(13)	0.9500

C(13)-C(14)	1.386(6)
C(14)-H(14)	0.9500
C(14)-C(15)	1.345(6)
C(15)-H(15)	0.9500
C(15)-C(16)	1.388(5)
C(16)-H(16)	0.9500
C(18)-H(18A)	0.9900
C(18)-H(18B)	0.9900
C(18)-C(19)	1.513(4)
C(19)-H(19A)	0.9900
C(19)-H(19B)	0.9900
C(20)-C(21)	1.391(4)
C(20)-C(25)	1.392(4)
C(21)-C(22)	1.396(4)
C(21)-C(26)	1.501(4)
C(22)-H(22)	0.9500
C(22)-C(23)	1.382(4)
C(23)-C(24)	1.384(4)
C(23)-C(27)	1.503(4)
C(24)-H(24)	0.9500
C(24)-C(25)	1.396(4)
C(25)-C(28)	1.501(4)
C(26)-H(26A)	0.9800
C(26)-H(26B)	0.9800
C(26)-H(26C)	0.9800
C(27)-H(27A)	0.9800
C(27)-H(27B)	0.9800
C(27)-H(27C)	0.9800
C(28)-H(28A)	0.9800
C(28)-H(28B)	0.9800
C(28)-H(28C)	0.9800
C(29)-C(30)	1.389(4)
C(29)-C(34)	1.400(4)
C(30)-C(31)	1.392(4)
C(30)-C(35)	1.503(4)
C(31)-H(31)	0.9500
C(31)-C(32)	1.392(4)
C(32)-C(33)	1.387(4)

C(32)-C(36)	1.504(4)
C(33)-H(33)	0.9500
C(33)-C(34)	1.387(4)
C(34)-C(37)	1.515(4)
C(35)-H(35A)	0.9800
C(35)-H(35B)	0.9800
C(35)-H(35C)	0.9800
C(36)-H(36A)	0.9800
C(36)-H(36B)	0.9800
C(36)-H(36C)	0.9800
C(37)-H(37A)	0.9800
C(37)-H(37B)	0.9800
C(37)-H(37C)	0.9800
O(1)-C(38)	1.398(6)
O(1)-C(41)	1.402(5)
C(38)-H(38A)	0.9900
C(38)-H(38B)	0.9900
C(38)-C(39)	1.517(7)
C(39)-H(39A)	0.9900
C(39)-H(39B)	0.9900
C(39)-C(40)	1.578(8)
C(40)-H(40A)	0.9900
C(40)-H(40B)	0.9900
C(40)-C(41)	1.472(7)
C(41)-H(41A)	0.9900
C(41)-H(41B)	0.9900
C(42)-H(42A)	0.9800
C(42)-H(42B)	0.9800
C(42)-H(42C)	0.9800
C(42)-C(43)	1.408(9)
C(43)-H(43A)	0.9900
C(43)-H(43B)	0.9900
C(43)-C(44)	1.445(10)
C(44)-H(44A)	0.9900
C(44)-H(44B)	0.9900
C(44)-C(45)	1.372(9)
C(45)-H(45A)	0.9900
C(45)-H(45B)	0.9900

C(45)-C(46)	1.427(9)
C(46)-H(46A)	0.9800
C(46)-H(46B)	0.9800
C(46)-H(46C)	0.9800
O(1')-C(39')	1.406(14)
O(1')-C(42')	1.442(15)
C(39')-H(39C)	0.9900
C(39')-H(39D)	0.9900
C(39')-C(40')	1.491(15)
C(40')-H(40C)	0.9900
C(40')-H(40D)	0.9900
C(40')-C(41')	1.528(18)
C(41')-H(41C)	0.9900
C(41')-H(41D)	0.9900
C(41')-C(42')	1.474(15)
C(42')-H(42D)	0.9900
C(42')-H(42E)	0.9900
O(2')-C(43')	1.440(15)
O(2')-C(46')	1.423(15)
C(43')-H(43C)	0.9900
C(43')-H(43D)	0.9900
C(43')-C(44')	1.483(16)
C(44')-H(44C)	0.9900
C(44')-H(44D)	0.9900
C(44')-C(45')	1.550(19)
C(45')-H(45C)	0.9900
C(45')-H(45D)	0.9900
C(45')-C(46')	1.485(16)
C(46')-H(46D)	0.9900
C(46')-H(46E)	0.9900
C(1)-Fe(1)-C(2)	40.35(11)
C(1)-Fe(1)-C(9)	171.32(11)
C(1)-Fe(1)-C(10)	131.26(11)
C(2)-Fe(1)-C(10)	91.48(11)
C(9)-Fe(1)-C(2)	131.04(11)
C(9)-Fe(1)-C(10)	40.07(10)
C(17)-Fe(1)-C(1)	95.08(10)
C(17)-Fe(1)-C(2)	135.10(10)

C(17)-Fe(1)-C(9)	93.60(10)
C(17)-Fe(1)-C(10)	133.42(10)
C(17)-N(1)-C(18)	113.65(19)
C(17)-N(1)-C(20)	126.24(19)
C(20)-N(1)-C(18)	120.11(19)
C(17)-N(2)-C(19)	113.5(2)
C(17)-N(2)-C(29)	127.05(19)
C(29)-N(2)-C(19)	119.3(2)
Fe(1)-C(1)-H(1A)	107.0(18)
Fe(1)-C(1)-H(1B)	112(2)
H(1A)-C(1)-H(1B)	124(3)
C(2)-C(1)-Fe(1)	70.62(15)
C(2)-C(1)-H(1A)	112.2(18)
C(2)-C(1)-H(1B)	117(2)
Fe(1)-C(2)-H(2)	108.2(15)
C(1)-C(2)-Fe(1)	69.03(14)
C(1)-C(2)-H(2)	116.1(17)
C(1)-C(2)-C(3)	123.9(3)
C(3)-C(2)-Fe(1)	110.35(19)
C(3)-C(2)-H(2)	116.5(16)
C(4)-C(3)-C(2)	123.3(2)
C(4)-C(3)-C(8)	116.9(3)
C(8)-C(3)-C(2)	119.8(3)
C(3)-C(4)-H(4)	119.1
C(5)-C(4)-C(3)	121.9(3)
C(5)-C(4)-H(4)	119.1
C(4)-C(5)-H(5)	119.8
C(4)-C(5)-C(6)	120.4(3)
C(6)-C(5)-H(5)	119.8
C(5)-C(6)-H(6)	120.5
C(7)-C(6)-C(5)	119.0(3)
C(7)-C(6)-H(6)	120.5
C(6)-C(7)-H(7)	119.5
C(6)-C(7)-C(8)	121.0(3)
C(8)-C(7)-H(7)	119.5
C(3)-C(8)-H(8)	119.6
C(7)-C(8)-C(3)	120.8(3)
C(7)-C(8)-H(8)	119.6

Fe(1)-C(9)-H(9A)	109.1(18)
Fe(1)-C(9)-H(9B)	115(2)
H(9A)-C(9)-H(9B)	111(3)
C(10)-C(9)-Fe(1)	70.80(15)
C(10)-C(9)-H(9A)	117.1(18)
C(10)-C(9)-H(9B)	126(2)
Fe(1)-C(10)-H(10)	105(2)
C(9)-C(10)-Fe(1)	69.12(14)
C(9)-C(10)-H(10)	115(2)
C(9)-C(10)-C(11)	124.5(3)
C(11)-C(10)-Fe(1)	110.16(19)
C(11)-C(10)-H(10)	118(2)
C(12)-C(11)-C(10)	123.0(3)
C(12)-C(11)-C(16)	117.5(3)
C(16)-C(11)-C(10)	119.5(3)
C(11)-C(12)-H(12)	119.7
C(11)-C(12)-C(13)	120.5(3)
C(13)-C(12)-H(12)	119.7
C(12)-C(13)-H(13)	119.6
C(14)-C(13)-C(12)	120.7(4)
C(14)-C(13)-H(13)	119.6
C(13)-C(14)-H(14)	120.4
C(15)-C(14)-C(13)	119.2(3)
C(15)-C(14)-H(14)	120.4
C(14)-C(15)-H(15)	119.5
C(14)-C(15)-C(16)	121.0(3)
C(16)-C(15)-H(15)	119.5
C(11)-C(16)-H(16)	119.5
C(15)-C(16)-C(11)	121.0(3)
C(15)-C(16)-H(16)	119.5
N(1)-C(17)-Fe(1)	126.86(17)
N(1)-C(17)-N(2)	106.86(19)
N(2)-C(17)-Fe(1)	126.28(17)
N(1)-C(18)-H(18A)	111.4
N(1)-C(18)-H(18B)	111.4
N(1)-C(18)-C(19)	101.9(2)
H(18A)-C(18)-H(18B)	109.2
C(19)-C(18)-H(18A)	111.4

C(19)-C(18)-H(18B)	111.4
N(2)-C(19)-C(18)	102.5(2)
N(2)-C(19)-H(19A)	111.3
N(2)-C(19)-H(19B)	111.3
C(18)-C(19)-H(19A)	111.3
C(18)-C(19)-H(19B)	111.3
H(19A)-C(19)-H(19B)	109.2
C(21)-C(20)-N(1)	118.1(2)
C(21)-C(20)-C(25)	122.2(2)
C(25)-C(20)-N(1)	119.7(2)
C(20)-C(21)-C(22)	117.8(3)
C(20)-C(21)-C(26)	121.9(2)
C(22)-C(21)-C(26)	120.4(3)
C(21)-C(22)-H(22)	119.1
C(23)-C(22)-C(21)	121.8(3)
C(23)-C(22)-H(22)	119.1
C(22)-C(23)-C(24)	118.6(2)
C(22)-C(23)-C(27)	120.1(3)
C(24)-C(23)-C(27)	121.4(3)
C(23)-C(24)-H(24)	119.0
C(23)-C(24)-C(25)	122.0(3)
C(25)-C(24)-H(24)	119.0
C(20)-C(25)-C(24)	117.6(2)
C(20)-C(25)-C(28)	122.3(2)
C(24)-C(25)-C(28)	120.2(3)
C(21)-C(26)-H(26A)	109.5
C(21)-C(26)-H(26B)	109.5
C(21)-C(26)-H(26C)	109.5
H(26A)-C(26)-H(26B)	109.5
H(26A)-C(26)-H(26C)	109.5
H(26B)-C(26)-H(26C)	109.5
C(23)-C(27)-H(27A)	109.5
C(23)-C(27)-H(27B)	109.5
C(23)-C(27)-H(27C)	109.5
H(27A)-C(27)-H(27B)	109.5
H(27A)-C(27)-H(27C)	109.5
H(27B)-C(27)-H(27C)	109.5
C(25)-C(28)-H(28A)	109.5

C(25)-C(28)-H(28B)	109.5
C(25)-C(28)-H(28C)	109.5
H(28A)-C(28)-H(28B)	109.5
H(28A)-C(28)-H(28C)	109.5
H(28B)-C(28)-H(28C)	109.5
C(30)-C(29)-N(2)	120.3(2)
C(30)-C(29)-C(34)	121.2(2)
C(34)-C(29)-N(2)	118.3(2)
C(29)-C(30)-C(31)	118.4(2)
C(29)-C(30)-C(35)	121.4(2)
C(31)-C(30)-C(35)	120.2(3)
C(30)-C(31)-H(31)	119.1
C(30)-C(31)-C(32)	121.8(2)
C(32)-C(31)-H(31)	119.1
C(31)-C(32)-C(36)	121.0(3)
C(33)-C(32)-C(31)	118.3(2)
C(33)-C(32)-C(36)	120.7(3)
C(32)-C(33)-H(33)	119.1
C(34)-C(33)-C(32)	121.7(2)
C(34)-C(33)-H(33)	119.1
C(29)-C(34)-C(37)	121.4(2)
C(33)-C(34)-C(29)	118.5(2)
C(33)-C(34)-C(37)	120.1(2)
C(30)-C(35)-H(35A)	109.5
C(30)-C(35)-H(35B)	109.5
C(30)-C(35)-H(35C)	109.5
H(35A)-C(35)-H(35B)	109.5
H(35A)-C(35)-H(35C)	109.5
H(35B)-C(35)-H(35C)	109.5
C(32)-C(36)-H(36A)	109.5
C(32)-C(36)-H(36B)	109.5
C(32)-C(36)-H(36C)	109.5
H(36A)-C(36)-H(36B)	109.5
H(36A)-C(36)-H(36C)	109.5
H(36B)-C(36)-H(36C)	109.5
C(34)-C(37)-H(37A)	109.5
C(34)-C(37)-H(37B)	109.5
C(34)-C(37)-H(37C)	109.5

H(37A)-C(37)-H(37B)	109.5
H(37A)-C(37)-H(37C)	109.5
H(37B)-C(37)-H(37C)	109.5
C(38)-O(1)-C(41)	108.7(4)
O(1)-C(38)-H(38A)	110.3
O(1)-C(38)-H(38B)	110.3
O(1)-C(38)-C(39)	107.0(4)
H(38A)-C(38)-H(38B)	108.6
C(39)-C(38)-H(38A)	110.3
C(39)-C(38)-H(38B)	110.3
C(38)-C(39)-H(39A)	111.7
C(38)-C(39)-H(39B)	111.7
C(38)-C(39)-C(40)	100.4(4)
H(39A)-C(39)-H(39B)	109.5
C(40)-C(39)-H(39A)	111.7
C(40)-C(39)-H(39B)	111.7
C(39)-C(40)-H(40A)	112.0
C(39)-C(40)-H(40B)	112.0
H(40A)-C(40)-H(40B)	109.7
C(41)-C(40)-C(39)	98.9(4)
C(41)-C(40)-H(40A)	112.0
C(41)-C(40)-H(40B)	112.0
O(1)-C(41)-C(40)	110.4(4)
O(1)-C(41)-H(41A)	109.6
O(1)-C(41)-H(41B)	109.6
C(40)-C(41)-H(41A)	109.6
C(40)-C(41)-H(41B)	109.6
H(41A)-C(41)-H(41B)	108.1
H(42A)-C(42)-H(42B)	109.5
H(42A)-C(42)-H(42C)	109.5
H(42B)-C(42)-H(42C)	109.5
C(43)-C(42)-H(42A)	109.5
C(43)-C(42)-H(42B)	109.5
C(43)-C(42)-H(42C)	109.5
C(42)-C(43)-H(43A)	105.9
C(42)-C(43)-H(43B)	105.9
C(42)-C(43)-C(44)	125.6(7)
H(43A)-C(43)-H(43B)	106.2

C(44)-C(43)-H(43A)	105.9
C(44)-C(43)-H(43B)	105.9
C(43)-C(44)-H(44A)	104.8
C(43)-C(44)-H(44B)	104.8
H(44A)-C(44)-H(44B)	105.8
C(45)-C(44)-C(43)	129.9(6)
C(45)-C(44)-H(44A)	104.8
C(45)-C(44)-H(44B)	104.8
C(44)-C(45)-H(45A)	105.5
C(44)-C(45)-H(45B)	105.5
C(44)-C(45)-C(46)	127.2(7)
H(45A)-C(45)-H(45B)	106.1
C(46)-C(45)-H(45A)	105.5
C(46)-C(45)-H(45B)	105.5
C(45)-C(46)-H(46A)	109.5
C(45)-C(46)-H(46B)	109.5
C(45)-C(46)-H(46C)	109.5
H(46A)-C(46)-H(46B)	109.5
H(46A)-C(46)-H(46C)	109.5
H(46B)-C(46)-H(46C)	109.5
C(39')-O(1')-C(42')	104.4(14)
O(1')-C(39')-H(39C)	110.2
O(1')-C(39')-H(39D)	110.2
O(1')-C(39')-C(40')	107.6(12)
H(39C)-C(39')-H(39D)	108.5
C(40')-C(39')-H(39C)	110.2
C(40')-C(39')-H(39D)	110.2
C(39')-C(40')-H(40C)	111.1
C(39')-C(40')-H(40D)	111.1
C(39')-C(40')-C(41')	103.3(11)
H(40C)-C(40')-H(40D)	109.1
C(41')-C(40')-H(40C)	111.1
C(41')-C(40')-H(40D)	111.1
C(40')-C(41')-H(41C)	111.4
C(40')-C(41')-H(41D)	111.4
H(41C)-C(41')-H(41D)	109.3
C(42')-C(41')-C(40')	101.7(12)
C(42')-C(41')-H(41C)	111.4

C(42')-C(41')-H(41D)	111.4
O(1')-C(42')-C(41')	102.4(13)
O(1')-C(42')-H(42D)	111.3
O(1')-C(42')-H(42E)	111.3
C(41')-C(42')-H(42D)	111.3
C(41')-C(42')-H(42E)	111.3
H(42D)-C(42')-H(42E)	109.2
C(46')-O(2')-C(43')	105.4(15)
O(2')-C(43')-H(43C)	110.5
O(2')-C(43')-H(43D)	110.5
O(2')-C(43')-C(44')	106.3(15)
H(43C)-C(43')-H(43D)	108.7
C(44')-C(43')-H(43C)	110.5
C(44')-C(43')-H(43D)	110.5
C(43')-C(44')-H(44C)	111.3
C(43')-C(44')-H(44D)	111.3
C(43')-C(44')-C(45')	102.4(13)
H(44C)-C(44')-H(44D)	109.2
C(45')-C(44')-H(44C)	111.3
C(45')-C(44')-H(44D)	111.3
C(44')-C(45')-H(45C)	111.2
C(44')-C(45')-H(45D)	111.2
H(45C)-C(45')-H(45D)	109.1
C(46')-C(45')-C(44')	102.7(13)
C(46')-C(45')-H(45C)	111.2
C(46')-C(45')-H(45D)	111.2
O(2')-C(46')-C(45')	110.7(14)
O(2')-C(46')-H(46D)	109.5
O(2')-C(46')-H(46E)	109.5
C(45')-C(46')-H(46D)	109.5
C(45')-C(46')-H(46E)	109.5
H(46D)-C(46')-H(46E)	108.1

Anisotropic displacement parameters ($Å^2$x 10^3) for **neijd10**.

The anisotropic displacement factor exponent takes the form: $-2\pi^2$[h^2 $a^{*2}U_{11}$ + ... + 2 h k a* b* U_{12}]

	U_{11}	U_{22}	U_{33}	U_{23}	U_{13}	U_{12}
Fe1	56(1)	30(1)	45(1)	2(1)	14(1)	-1(1)
N1	69(1)	32(1)	41(1)	1(1)	11(1)	-9(1)
N2	66(1)	33(1)	41(1)	0(1)	12(1)	-7(1)
C1	62(2)	34(1)	62(2)	-3(1)	18(1)	5(1)
C2	64(2)	30(1)	56(2)	0(1)	15(1)	4(1)
C3	68(2)	32(1)	54(1)	-1(1)	18(1)	2(1)
C4	70(2)	38(1)	55(2)	2(1)	16(1)	-1(1)
C5	78(2)	47(1)	56(2)	-3(1)	11(1)	-3(1)
C6	80(2)	52(2)	65(2)	-7(1)	11(2)	-17(1)
C7	93(2)	46(1)	64(2)	2(1)	20(2)	-15(2)
C8	82(2)	39(1)	53(1)	1(1)	15(1)	-9(1)
C9	57(2)	44(1)	54(1)	4(1)	21(1)	7(1)
C10	57(2)	45(1)	50(1)	1(1)	18(1)	-2(1)
C11	59(2)	50(1)	47(1)	5(1)	17(1)	8(1)
C12	62(2)	69(2)	59(2)	5(1)	15(1)	-4(1)
C13	60(2)	109(3)	57(2)	2(2)	4(1)	4(2)
C14	82(2)	94(3)	58(2)	21(2)	16(2)	31(2)
C15	96(3)	66(2)	62(2)	18(2)	19(2)	20(2)
C16	84(2)	48(1)	60(2)	9(1)	21(2)	6(1)
C17	53(1)	32(1)	44(1)	-1(1)	15(1)	2(1)
C18	83(2)	36(1)	45(1)	2(1)	14(1)	-9(1)
C19	104(2)	43(1)	47(1)	-1(1)	16(1)	-24(1)
C20	63(2)	31(1)	44(1)	0(1)	10(1)	-2(1)
C21	62(2)	37(1)	51(1)	0(1)	11(1)	-2(1)
C22	74(2)	39(1)	54(1)	-3(1)	17(1)	4(1)
C23	72(2)	38(1)	51(1)	-1(1)	7(1)	-5(1)
C24	64(2)	39(1)	53(1)	3(1)	6(1)	-4(1)
C25	63(2)	36(1)	46(1)	3(1)	10(1)	-2(1)
C26	63(2)	55(2)	65(2)	-3(1)	17(1)	-1(1)
C27	94(2)	55(2)	68(2)	-18(2)	6(2)	-8(2)
C28	64(2)	54(2)	63(2)	-1(1)	12(1)	5(1)
C29	57(1)	36(1)	42(1)	0(1)	11(1)	-2(1)
C30	52(1)	38(1)	54(1)	-4(1)	11(1)	-4(1)

C31	51(1)	50(1)	53(1)	4(1)	5(1)	1(1)
C32	57(2)	64(2)	45(1)	3(1)	9(1)	4(1)
C33	61(2)	54(1)	48(1)	-4(1)	12(1)	6(1)
C34	60(2)	41(1)	46(1)	1(1)	6(1)	3(1)
C35	55(2)	68(2)	79(2)	-25(2)	8(1)	2(1)
C36	70(2)	127(3)	50(2)	-6(2)	7(2)	20(2)
C37	81(2)	44(1)	56(2)	0(1)	3(1)	14(1)
O1	63(2)	56(2)	101(2)	-8(1)	8(2)	0(1)
C38	73(3)	57(2)	99(4)	-1(2)	10(3)	5(2)
C39	82(3)	61(3)	93(3)	4(2)	3(3)	0(2)
C40	81(3)	73(3)	87(3)	17(3)	-3(3)	-14(3)
C41	62(2)	69(3)	90(3)	-9(2)	12(2)	-10(2)
C42	118(5)	115(5)	140(6)	58(5)	-18(4)	-25(4)
C43	127(5)	122(6)	89(4)	22(4)	4(4)	-34(5)
C44	140(6)	139(5)	93(4)	44(4)	-49(4)	-57(5)
C45	103(5)	119(7)	95(4)	30(4)	-9(4)	-7(4)
C46	155(6)	100(4)	95(4)	29(3)	-39(4)	-49(4)
O1'	109(7)	110(8)	99(7)	15(7)	-9(6)	-46(6)
C39'	101(7)	94(7)	105(7)	4(7)	-1(6)	-7(6)
C40'	94(9)	74(7)	121(10)	-21(8)	25(8)	-10(8)
C41'	125(19)	88(14)	150(20)	-2(15)	-27(17)	20(13)
C42'	140(20)	119(19)	140(20)	-10(17)	-10(17)	10(17)
O2'	154(12)	172(18)	153(12)	0(11)	0(10)	-56(13)
C43'	130(9)	148(10)	103(9)	14(8)	9(7)	6(9)
C44'	153(9)	147(12)	121(8)	21(9)	-1(8)	-16(10)
C45'	163(9)	145(10)	120(9)	31(9)	-18(8)	-44(10)
C46'	130(9)	119(12)	105(8)	17(9)	-8(8)	7(9)

Hydrogen coordinates (x 10^4) and isotropic displacement parameters (Å^2 x 10^3) for **neijd10**.

	x	y	z	U(eq)
H1A	2090(30)	2470(20)	6629(15)	60(9)
H1B	2890(30)	2600(20)	7361(17)	74(10)
H2	3280(20)	3249(16)	6168(14)	40(7)
H4	4570	2972	7758	64
H5	5961	3818	8249	72
H6	6739	4931	7700	79
H7	6094	5184	6657	80
H8	4667	4363	6162	69
H9A	5680(20)	950(20)	6503(14)	53(8)
H9B	4810(30)	500(30)	5905(19)	93(12)
H10	5560(30)	2360(20)	6176(16)	66(9)
H12	3477	1203	5173	75
H13	2647	1694	4207	90
H14	3156	3030	3790	93
H15	4493	3854	4335	88
H16	5328	3390	5303	75
H18A	2606	-369	8076	65
H18B	3752	-762	7933	65
H19A	2883	-1311	7045	77
H19B	1818	-738	7121	77
H22	2954	2512	9050	66
H24	6034	1846	8912	63
H26A	1758	1438	7741	91
H26B	1511	1946	8360	91
H26C	1672	902	8374	91
H27A	4721	3546	9394	109
H27B	5702	2916	9653	109
H27C	4566	2833	9923	109
H28A	5586	29	8008	90
H28B	6495	733	8250	90
H28C	5815	848	7576	90
H31	989	294	4844	62
H33	3602	-1277	4907	65
H35A	802	429	6383	101
H35B	438	925	5736	101
H35C	1441	1285	6200	101

H36A	2689	-549	3876	124
H36B	1455	-274	3896	124
H36C	1813	-1281	3993	124
H37A	4737	-765	6402	91
H37B	4850	-1397	5819	91
H37C	4128	-1694	6349	91
H38A	2779	7532	3092	91
H38B	2970	7472	2367	91
H39A	1803	6292	2824	95
H39B	1464	6623	2114	95
H40A	-115	7026	2568	98
H40B	576	7283	3229	98
H41A	393	8225	2092	88
H41B	432	8617	2786	88
H42A	2808	6873	4360	191
H42B	2073	6185	3937	191
H42C	1550	6787	4431	191
H43A	2601	6132	5213	136
H43B	3119	5537	4723	136
H44A	1024	5377	4970	155
H44B	1636	4748	4546	155
H45A	2056	4818	5798	129
H45B	2482	4127	5336	129
H46A	1411	3393	5820	182
H46B	683	3610	5175	182
H46C	530	4161	5792	182
H39C	573	7810	2281	121
H39D	1166	6952	2050	121
H40C	2053	8154	2885	114
H40D	2627	7268	2679	114
H41C	2145	6492	3508	151
H41D	2090	7453	3823	151
H42D	383	6580	3705	160
H42E	287	7594	3481	160
H43C	3829	5354	5195	153
H43D	2963	6097	5321	153
H44C	2725	4758	4393	170
H44D	2419	5786	4306	170

H45C	825	5615	4730	175
H45D	927	4606	4511	175
H46D	1079	5169	5698	143
H46E	1298	4184	5479	143

Torsion angles [°] for **neijd10**.

Fe1-C1-C2-C3	-101.0(2)
Fe1-C2-C3-C4	-59.7(3)
Fe1-C2-C3-C8	120.6(2)
Fe1-C9-C10-C11	-100.7(3)
Fe1-C10-C11-C12	-55.4(3)
Fe1-C10-C11-C16	123.5(2)
N1-C18-C19-N2	11.9(3)
N1-C20-C21-C22	-179.1(2)
N1-C20-C21-C26	0.7(4)
N1-C20-C25-C24	180.0(2)
N1-C20-C25-C28	0.1(4)
N2-C29-C30-C31	-179.2(2)
N2-C29-C30-C35	1.1(4)
N2-C29-C34-C33	179.0(2)
N2-C29-C34-C37	-2.6(4)
C1-C2-C3-C4	18.1(4)
C1-C2-C3-C8	-161.5(3)
C2-C3-C4-C5	-178.3(3)
C2-C3-C8-C7	179.1(3)
C3-C4-C5-C6	-1.2(5)
C4-C3-C8-C7	-0.5(4)
C4-C5-C6-C7	0.1(5)
C5-C6-C7-C8	0.7(5)
C6-C7-C8-C3	-0.5(5)
C8-C3-C4-C5	1.4(4)
C9-C10-C11-C12	22.6(4)
C9-C10-C11-C16	-158.5(3)
C10-C11-C12-C13	179.2(3)
C10-C11-C16-C15	-179.5(3)
C11-C12-C13-C14	-0.1(5)
C12-C11-C16-C15	-0.6(4)
C12-C13-C14-C15	0.2(5)
C13-C14-C15-C16	-0.5(5)
C14-C15-C16-C11	0.7(5)
C16-C11-C12-C13	0.3(4)
C17-N1-C18-C19	-10.7(3)
C17-N1-C20-C21	98.1(3)

C17-N1-C20-C25	-84.6(3)
C17-N2-C19-C18	-10.9(3)
C17-N2-C29-C30	-79.5(3)
C17-N2-C29-C34	104.7(3)
C18-N1-C17-Fe1	-174.89(19)
C18-N1-C17-N2	4.3(3)
C18-N1-C20-C21	-82.0(3)
C18-N1-C20-C25	95.3(3)
C19-N2-C17-Fe1	-176.2(2)
C19-N2-C17-N1	4.6(3)
C19-N2-C29-C30	95.8(3)
C19-N2-C29-C34	-80.0(3)
C20-N1-C17-Fe1	5.0(4)
C20-N1-C17-N2	-175.8(2)
C20-N1-C18-C19	169.4(3)
C20-C21-C22-C23	-1.7(4)
C21-C20-C25-C24	-2.8(4)
C21-C20-C25-C28	177.3(2)
C21-C22-C23-C24	-1.0(4)
C21-C22-C23-C27	178.6(3)
C22-C23-C24-C25	1.8(4)
C23-C24-C25-C20	0.0(4)
C23-C24-C25-C28	179.9(2)
C25-C20-C21-C22	3.6(4)
C25-C20-C21-C26	-176.6(2)
C26-C21-C22-C23	178.5(3)
C27-C23-C24-C25	-177.8(3)
C29-N2-C17-Fe1	-0.7(4)
C29-N2-C17-N1	-179.9(2)
C29-N2-C19-C18	173.2(2)
C29-C30-C31-C32	1.8(4)
C30-C29-C34-C33	3.3(4)
C30-C29-C34-C37	-178.3(2)
C30-C31-C32-C33	0.1(4)
C30-C31-C32-C36	-178.9(3)
C31-C32-C33-C34	-0.3(4)
C32-C33-C34-C29	-1.3(4)
C32-C33-C34-C37	-179.8(3)

C34-C29-C30-C31	-3.5(4)
C34-C29-C30-C35	176.7(3)
C35-C30-C31-C32	-178.4(3)
C36-C32-C33-C34	178.7(3)
O1-C38-C39-C40	-32.2(6)
C38-O1-C41-C40	11.6(6)
C38-C39-C40-C41	36.3(5)
C39-C40-C41-O1	-30.6(5)
C41-O1-C38-C39	14.4(6)
C42-C43-C44-C45	-173.5(11)
C43-C44-C45-C46	170.7(10)
O1'-C39'-C40'-C41'	-3(3)
C39'-O1'-C42'-C41'	-45(2)
C39'-C40'-C41'-C42'	-24(2)
C40'-C41'-C42'-O1'	42(2)
C42'-O1'-C39'-C40'	29(3)
O2'-C43'-C44'-C45'	35(3)
C43'-O2'-C46'-C45'	16(4)
C43'-C44'-C45'-C46'	-24(4)
C44'-C45'-C46'-O2'	5(4)
C46'-O2'-C43'-C44'	-33(3)
